# Vicinal stereocenters *via* asymmetric allylic alkylation and Cope rearrangement: a straightforward route to functionally and stereochemically rich heterocycles†

**DOI:** 10.1039/d2sc07021a

**Published:** 2023-02-14

**Authors:** Aleksandra Nilova, Michael D. Mannchen, Abdias N. Noel, Evgeniya Semenova, Alexander J. Grenning

**Affiliations:** a Department of Chemistry, University of Florida PO Box 117200 Gainesville 32611 FL USA grenning@ufl.edu

## Abstract

An asymmetric allylic alkylation/Cope rearrangement (AAA/[3,3]) capable of stereoselectively constructing vicinal stereocenters has been developed. Strategically integrated 4-methylation on the 3,3-dicyano-1,5-diene controls stereoselectivity and drives Cope rearrangement equilibrium in the forward direction. The AAA/[3,3] sequence rapidly converts abundant achiral and racemic starting materials into valuable (hetero)cycloalkane building blocks bearing significant functional and stereochemical complexity, highlighting the value of (hetero)cyclohexylidenemalononitriles as launching points for complex heterocycle synthesis. On this line, the resulting alkylidenemalononitrile moiety can be readily converted into amides *via* Hayashi–Lear amidation to ultimately yield amido-piperidines, tropanes, and related scaffolds with 3–5 stereocenters and drug-like functionality.

## Abstract 2

Functionally and stereochemically rich saturated heterocycles are accessed stereoselectively from abundant resources. This work showcases the sequenced value of asymmetric allylic alkylation, Cope rearrangement, and Hayashi–Lear oxidative amidation.

## Graphical abstract

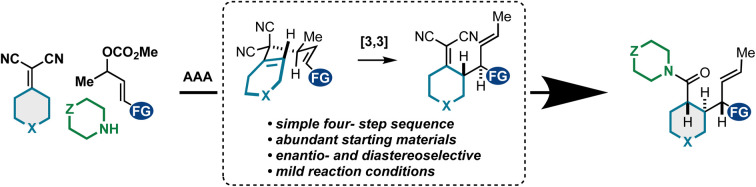

## Introduction

**Fig. 1:**
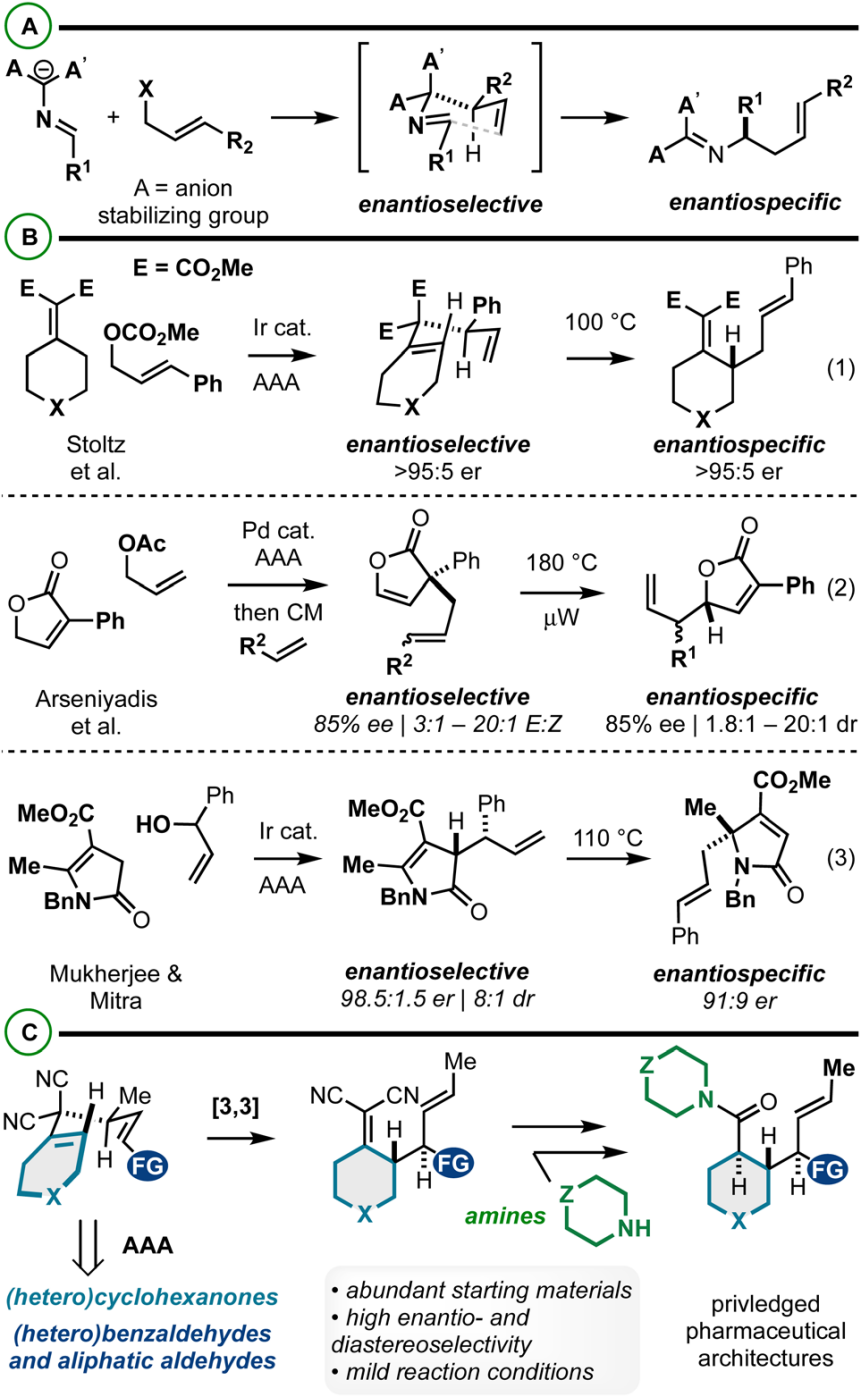
(A and B) Summary of AAA/2-aza-Cope rearrangement (A) and AAA/Cope rearrangement (B). (C) This report: an enantio- and diastereoselective AAA/Cope rearrangement for constructing functionally- and stereochemically dense pharmaceutical architectures.

**Scheme 1:**
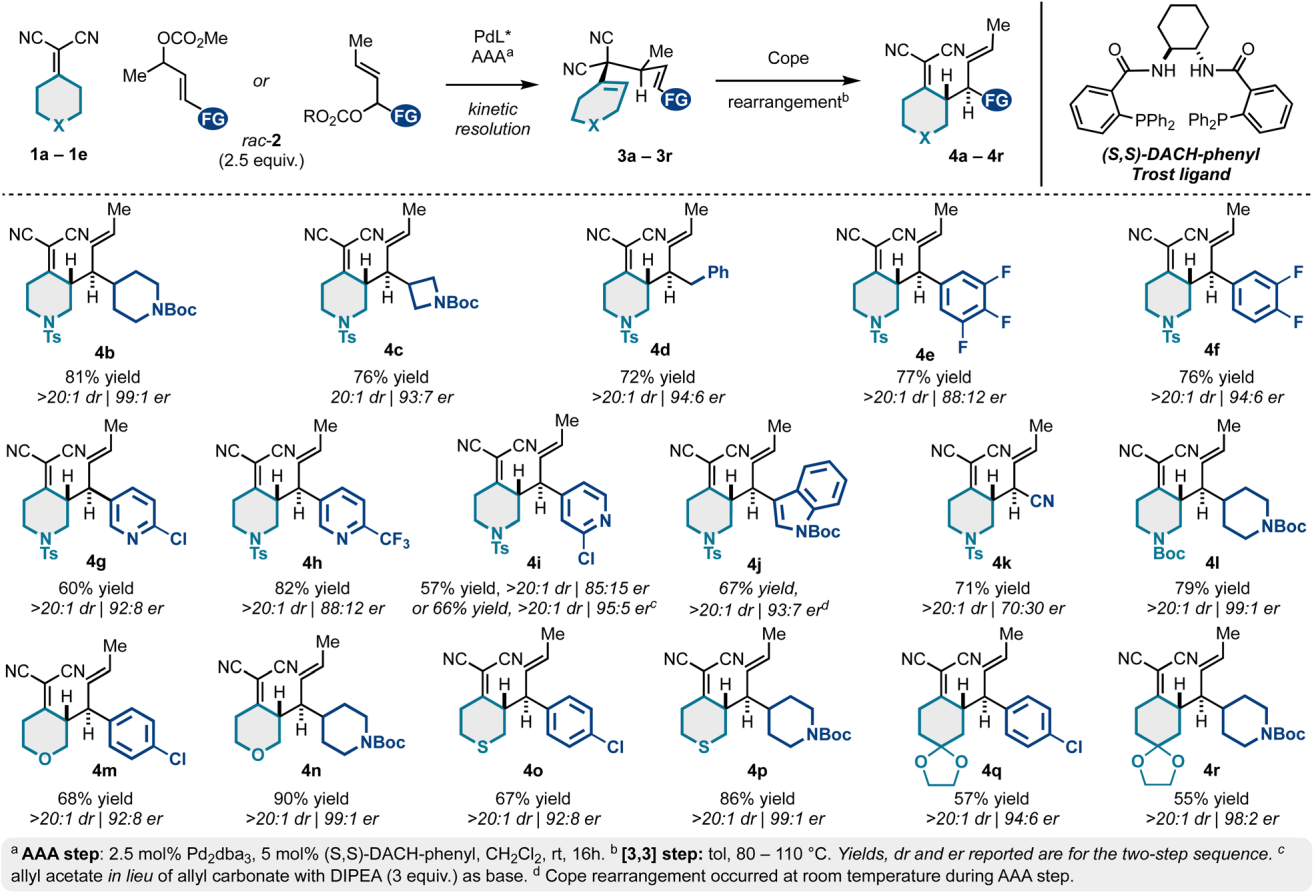
Scope of the Pd-catalyzed kinetic resolution/Cope rearrangement sequence.

Iterative asymmetric allylic alkylation (AAA) and sigmatropic rearrangement is an effective strategy to relay functional groups and stereochemistry about a molecular building block. AAA/2-aza-Cope rearrangement has received significant attention in the synthesis of α-chiral amines asymmetrically *via* catalysis (Fig. 1A).^1–14^ With respect to AAA/Cope rearrangements, there are relatively few reports. Stoltz *et al.* in 2016 reported the iridium-catalyzed AAA/Cope rearrangement between alkylidenemalonates and cinnamyl carbonates (Fig. 1B, eqn (1)).^15^ The initial stereochemistry-generating AAA is highly enantioselective (>90% in most cases), and thus the final Cope rearrangement products too are highly enantioenriched *via* enantiospecific Cope rearrangement which occurs at 100 °C. More recently, Arseniyadis *et al.*^16^ and Mukherjee and Mitra^17^ described AAA/Cope rearrangements to yield substituted butenolides (Fig. 1B, eqn (2)) and butyrolactams (Fig. 1B, eqn (3)), respectively. The former method includes an intermediate cross-metathesis step that yields Cope rearrangement substrates of varying stereochemical purity, which is reflected in the Cope rearrangement products. Noted here is also the kinetic challenge of promoting Cope rearrangements that yield vicinal stereocenters (180 °C heating reported). Similarly, in the latter case, enantio- (98.5 : 1.5 er) and diastereomeric- (8 : 1 dr) mixtures (from AAA reaction) ultimately yield α-3°-amine-containing products with diminished enantiopurity due to stereodivergent Cope rearrangement mechanisms. Despite the value of these methods, they illustrate the effects of incomplete stereocontrol in the AAA/sigmatropic rearrangement sequence as well as the energetic challenges associated with this approach to vicinal stereocenter construction.^18–21^ Further, the aforementioned AAA/2-aza-Cope rearrangements (Scheme 1A) have yet to bear reports on diastereoselectivity about the [3,3] step, potentially due to these stereochemical issues or [3,3] energetic challenges.

Herein we report an enantio- and diastereoselective AAA/Cope rearrangement sequence between alkylidenemalononitriles and 1,3-disubstituted allylic electrophiles to yield functionally and stereochemically complex building blocks (Fig. 1C). Strategically integrated 4-methylation on the 3,3-dicyano-1,5-diene both controls stereoselectivity and drives Cope rearrangement equilibrium in the forward direction thus favoring the construction of the vicinal stereocenters at various temperatures (rt – 110 °C). The products contain two different alkene functional groups: the alkylidenemalononitrile can be mildly converted to amides diastereoselectively by NaBH_4_ reduction and Hayashi oxidative amidation/esterification.^22,23^ To maximize the impact of our work, we focused on the convergent coupling of functionalized starting materials, thus showcasing the tolerance of the sequence to structural modification and the potential value of the sequence for accessing functionally and stereochemically rich pharmaceutical leads. Therefore, this work yields complementary access to functionally- and structurally-complex piperidines, cyclohexanes, and related saturated heterocycles.^24^ Recent state of the art approaches to complex piperidines include C–H functionalization,^25–28^ from pyridine derivatives by hydrogenation or nucleophilic dearomatization,^29–36^ and other methods.^37–40^ A standout route to related cyclohexanes was described by Baran whereby Diels–Alder cycloaddition, desymmetrization, and decarboxylative coupling chemistry are impactfully harnessed.^41^

## Results and discussion

To begin our work, we examined a model asymmetric allylic alkylation (AAA)/Cope rearrangement yielding vicinally stereogenic product 4a*via* 4-methyl-3,3-dicyano-1,5-dienes 3a enantioselectively from alkylidenemalononitrile 1a and racemic allylic electrophiles *rac*-2a (Table 1). While this class of 1,5-diene has not yet been accessed enantioselectively, our previous work established the fundamental Cope rearrangement reactivity of these substrates: 4-methyl-3,3-dicyano-1,5-dienes have favourable energetic profiles for rearrangement to vicinally stereogenic products whereas analogous des-methyl Cope substrates do not.^18,21^ Based on our optimization studies (Table 1), it was found that the coupling of 1 equivalent of alkylidenemalononitrile 1a and 2.5 equivalents of racemic allylic carbonate *rac*-2a catalyzed by a Pd/(S,S)-DACH-phenyl Trost ligand complex facilitated full conversion to the desired product in good enantiomeric ratio (Table 1, entry 1). Using equimolar amounts of the starting materials resulted in full conversion but insignificant 53 : 47 er (Table 1, entry 2). These findings suggest the reaction proceeds by a Pd-catalyzed kinetic resolution^42–45^ rather than a dynamic kinetic asymmetric transformation (DYKAT).^46–49^ Further increasing the equivalents of the electrophilic component did not improve the enantiomeric ratio (Table 1, entry 3), neither did switching to the Pd/naphthyl-Trost complex (Table 1, entry 4). However, we did see some impact with respect to the leaving group, as the allyl acetate (Table 1, entries 6–7) resulted in a slightly increased enantiomeric ratio compared to the methyl carbonate. Additionally, the residual allylcarbonate (2a) was found to be highly enantioenriched (92 : 8 er) (Table 1). Considering 2.5 equivalents of *rac*-2a were initially used, the 92 : 8 er was somewhat unexpected. This potentially suggests that *in situ* generated reagents (*e.g.*, carbonate anion, hydrogen methyl carbonate, or methanol) could be acting as a nucleophile or base to further consume the reactive enantiomer of 2a. Additional experimentation is required to better understand this enantioselective transformation.

**Table 1:** Optimization of enantioselective 1,5-diene synthesis to 4a. 2a is also enantioenriched *via* the kinetic resolution

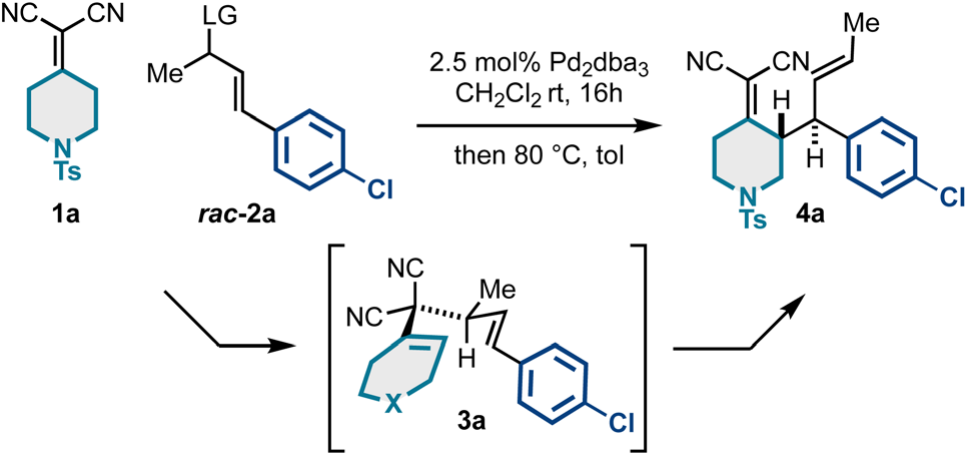						
Entry	Equiv. *rac*-2a	LG	Base	Ligand (5 mol%)	% Conv. (yield)	er
1^a^	2.5	OCO_2_Me	—	(S,S)-DACH-phenyl	100% (67%)	94 : 6
2	1	OCO_2_Me	—	(S,S)-DACH-phenyl	98%	53 : 47
3	5	OCO_2_Me	—	(S,S)-DACH-phenyl	100%	88 : 12
4	2.5	OCO_2_Me	—	(S,S)-DACH-naphthyl	100%	83 : 17
5	2.5	OBoc	—	(S,S)-DACH-phenyl	100%	93 : 7
6	2.5	OAc	DIPEA	(S,S)-DACH-phenyl	100% (55%)	95 : 5
7	2.5	OAc	K_3_PO_4_	(S,S)-DACH-phenyl	100% (74%)	97 : 3

a The excess electrophile 2a was isolated in 40% yield (of a maximum 50%; kinetic resolution) and 92 : 8 er.

We next examined the scope of a tandem catalytic-asymmetric 3,3-dicyano-1,5-diene synthesis/Cope rearrangement for preparing enantioenriched building blocks 4 diastereoselectively. As evidenced in Scheme 1, the sequence is extremely tolerant to structural modifications and is generally high yielding and stereoselective (up to 99 : 1 er). This is due to the mild conditions for both the catalytic-asymmetric allylic alkylation and the Cope rearrangement. This approach allows access to *N*-tosyl-piperidine alkylidenemalononitriles bearing *N*-Boc-piperidyl (4b), *N*-Boc-azetidinyl (4c), benzyl (4d), 4-chlorophenyl (4a; Table 1), fluorophenyl (4e–4f), differentially substituted pyridyl groups (4g–4i), and indole (4j).

A decrease in er was observed for several (hetero)aromatic examples (*e.g.*, 4a, 4e, 4h, 4i), yet this could be improved by exchanging the allyl carbonate for the corresponding allyl acetate electrophiles (*e.g.*, 4a, 4i, and 4j). The incorporation of a nitrile functional group (4k) was not particularly selective under conditions of asymmetric allylic alkylation *via* kinetic resolution. However, allylic alkylation of crotyl cyanohydrin can be achieved in high er *via* stereospecific allylic alkylation.^19,50^ The *N*-tosyl protecting group common to products 4a–4k can be exchanged for a more easily removable *N*-Boc group (4l) without notable changes to yield or er. Similarly, we examined various (hetero)cyclohexylidenemalononitriles 1c–1e. On this line, substituted tetrahydropyran (1c to 4m–4n), thiopyran (1d to 4o–4p), and ketal-protected cyclohexanone (1e to 4q–4r) analogs were accessed in high er.

**Scheme 2:**
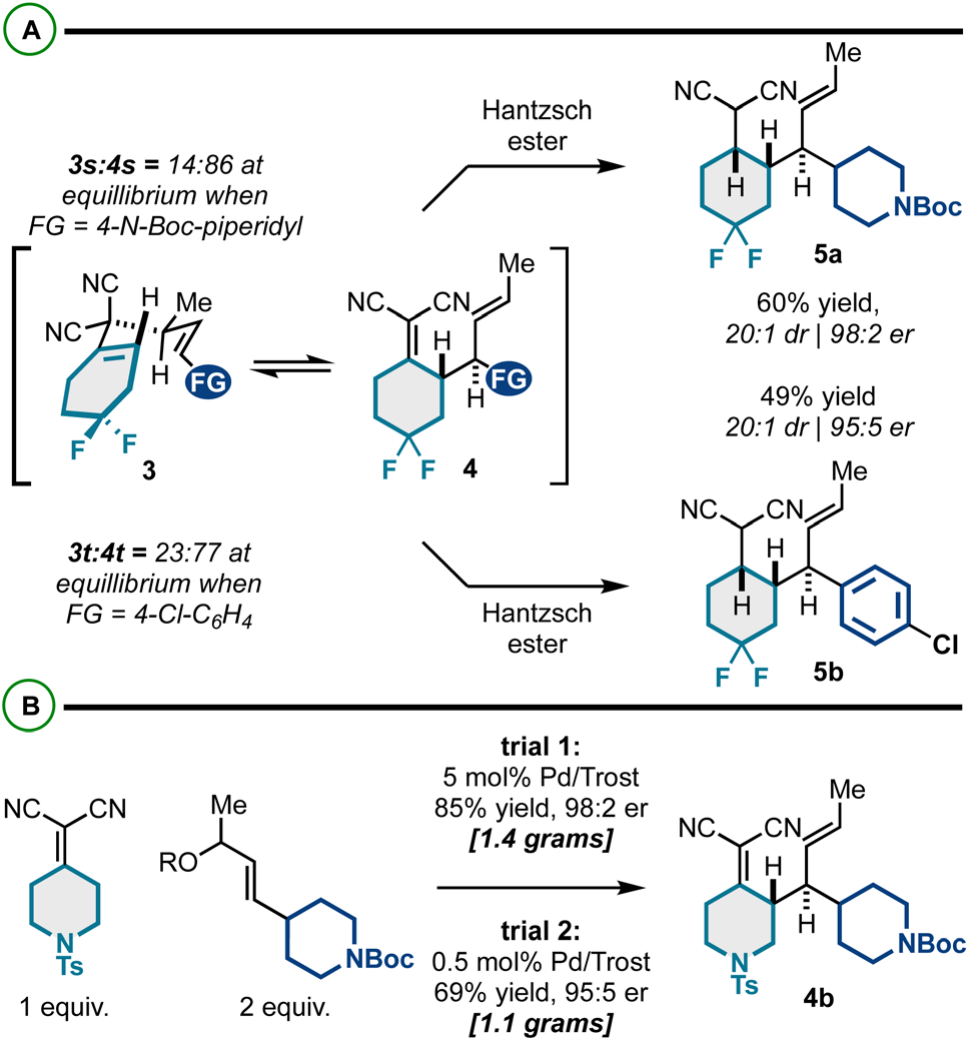
(A) Application of the reductive Cope rearrangement for substrates with unfavorable energetic profiles. (B) Scalability of Pd-catalyzed kinetic resolution/Cope rearrangement.

We also targeted difluorocyclohexyl products (Scheme 2A). Interestingly, these substrates had poor thermodynamic profiles for Cope rearrangement, and the respective Cope isomers 3 and 4 were inseparable by column chromatography. To yield the targeted building blocks, we employed our previously reported “reductive Cope rearrangement” to drive forward the [3,3] process.^19,51,52^ As such, promoting Cope rearrangement in the presence of Hantzsch ester resulted in full conversion and good yields and stereoselectivity for products 5a and 5b.

Importantly, the enantioselective synthesis of products 4 is scalable; as shown in Scheme 2B, we efficiently prepared gram quantities of a representative building block 4b. The catalyst loading could also be reduced tenfold, though the efficiency decreased slightly (69% yield, 95 : 5 er). Overall, these results suggest that large quantities of these functionally and stereochemically dense building blocks can be efficiently generated for diverse downstream modification.

**Scheme 3:**
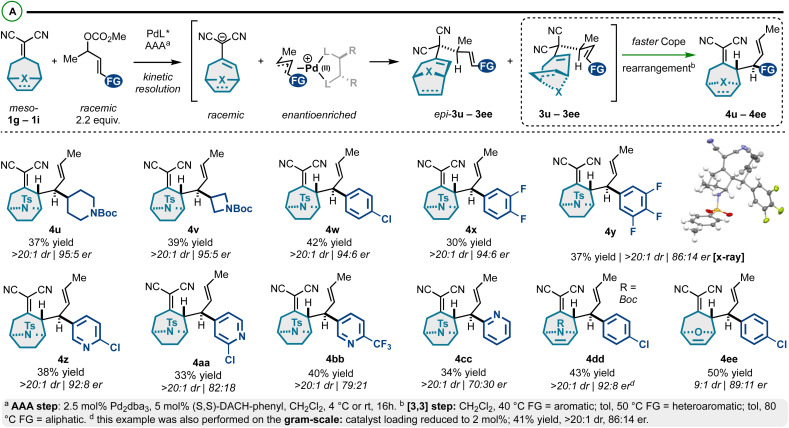
(A) *Meso*-bicyclicalkylidenemalononitriles react *via* Pd-catalyzed AAA and Cope rearrangement kinetic resolution.

In addition to the exploration of achiral alkylidenemalononitriles 1a–1f (Schemes 1 and 2), prochiral alkylidenemalononitriles 1g–1i were also investigated (Scheme 3).^53,54^ For these substrates, we observed a high degree of enantioselectivity with respect to asymmetric allylic alkylation, but an ∼50 : 50 mixture of 1,5-diene diastereomers: the chiral-racemic carbanions generated by deprotonation of 1g–1i couple at equivalent rates with the chiral-nonracemic Pd-π-allyl intermediates, thus yielding enantioenriched diastereomers epi-3u–3ee and 3u–3ee (Scheme 3A). These diastereomers were resolved through a Cope rearrangement to yield products 4u–4ee (maximum theoretical yield of 50%). The prepared compounds exhibit a high degree of structural diversity. Specifically, we accessed *N*-tosyl-tropinones bearing *N*-Boc-piperidyl (4u), *N*-Boc-azetidyl (4v), 4-chlorophenyl (4w), fluoroaromatic (4x and 4y), and differentially substituted pyridyl groups (4z–4cc). The structure of the products was confirmed by an X-ray crystallographic analysis of enantiopure compound 4y. While the stereoselectivity in this series was generally good (86 : 14 er–95 : 5 er), some modestly selective examples were observed in the pyridyl series (*e.g.*, 4cc). In addition, we assessed the reactivity of aza- and oxo-[3.2.1]cyclooctene-based alkylidenemalononitriles. The corresponding products (4dd and 4ee) were isolated in high yields and good er. Finally, this reaction could be performed on the gram scale (Scheme 3B), though a slightly diminished enantiomeric ratio was observed (98 : 2 *vs.* 86 : 14).

**Scheme 4:**
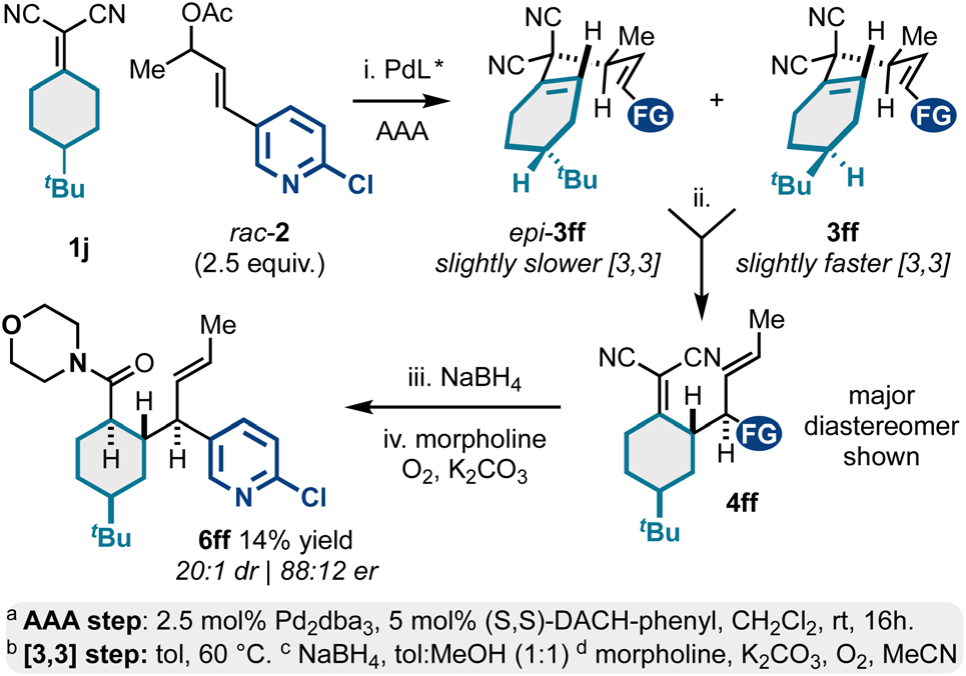
*Meso*-4-substituted cyclohexylidenemalononitriles as reactants for Pd-catalyzed AAA and Cope rearrangement.

In addition to *meso*-bicyclic alkylidenemalononitriles 1g–1i (*e.g.*, tropane derivatives; Scheme 3), we also examined *meso*-4-substituted cyclohexylidenemalononitrile 1j (Scheme 4). This substrate displayed some practical challenges but could still be converted to valuable, enantioenriched polysubstituted and functionalized cyclohexanes. Like the chemistry described in Scheme 3, the AAA-step here yielded two diastereomeric 1,5-dienes, *epi*-3ff and 3ff. In contrast, the [3,3] kinetic profiles for both diastereomers were similar resulting in a major product and minor product diastereomeric mixture *via* [3,3]. For the case examined, the Cope starting material 3ff and Cope product 4ff diastereomers were inseparable *via* silica gel chromatography. Thus, to access characterizable products, we opted to chemically separate the isomers *via* NaBH_4_ reduction and oxidative amidation with morpholine. This was effective and yielded product 6ff as a single diastereomer (88 : 12 er) in 14% overall yield from 1j. This sequence also showcases a key challenge in need of addressing in future studies: there are currently no methods to desymmetrize *meso*-alkylidenemalononitriles. Such a development would address the stereoselectivity challenges present in Schemes 3 and 4.

**Fig. 2:**
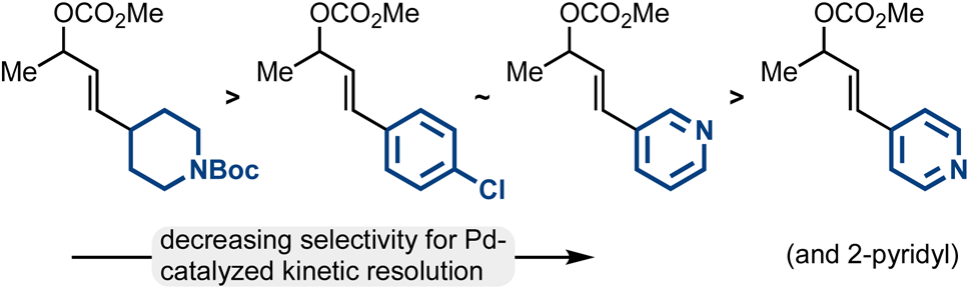
Trends in Pd-catalyzed kinetic resolution efficiency.

While the Pd-catalyzed kinetic resolution of substrates like *rac*-2 has been disclosed in the past, previous reports had limited examples of diverse electrophiles.^42–45^ Our work thoroughly establishes the types of allylic electrophiles that are highly efficient (the methyl/aliphatic allylic electrophiles; 93 : 7 er–99 : 1 er), reasonably efficient (the methyl/aryl allylic electrophiles including 3-pyridyl; 86 : 14 er–93 : 7 er), and modestly efficient (methyl/2- and 4-pyridyl allylic electrophiles; 70 : 30 er–85 : 15 er) (Fig. 2).

**Scheme 5:**
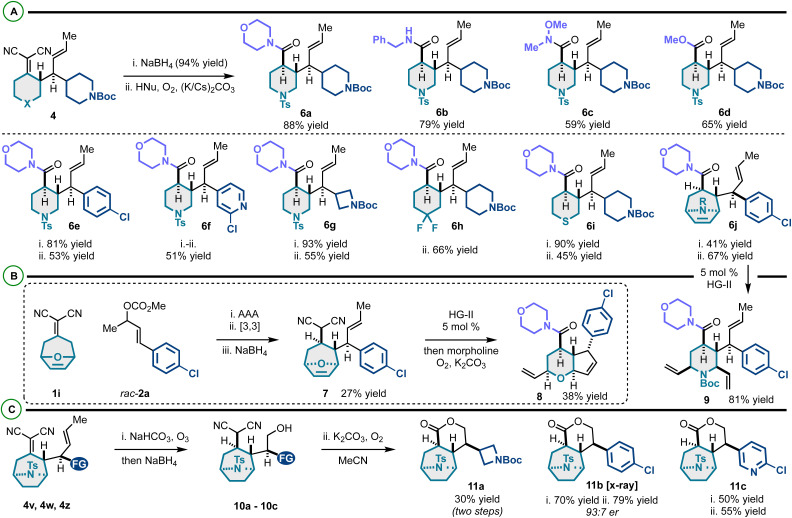
(A) Functional group interconversion to complex (hetero)cycloalkanes. (B) Telescoped sequence and examples of olefin metathesis for functional group interconversion. (C) Alkene ozonolysis and intramolecular oxidative esterification to complex lactones.

This efficient enantioselective coupling of alkylidenemalononitriles and racemic allylic electrophiles yields diverse vicinally stereogenic building blocks with orthogonal alkene functional handles. We envisaged that the alkylidenemalononitrile moiety could be converted to amides/esters *via* NaBH_4_ reduction and Hayashi oxidative amidation or esterification,^22,23^ and the neutral alkene could be utilized in olefin metathesis^55^ and ozonolysis^56^ as summarized in Scheme 4. On this line, the alkylidenemalononitrile can be consistently converted to carboxylate derivatives (Scheme 5A). This transformation gives access to amide and ester products derived from 2° amines (6a), 1° amines (6b), methoxymethyl amine (yielding a Weinreb amide) (6c), and methanol (6d). High compatibility of this protocol was confirmed through the presence and persistence of various functional groups on products 6e–6j.

**Scheme 6:**
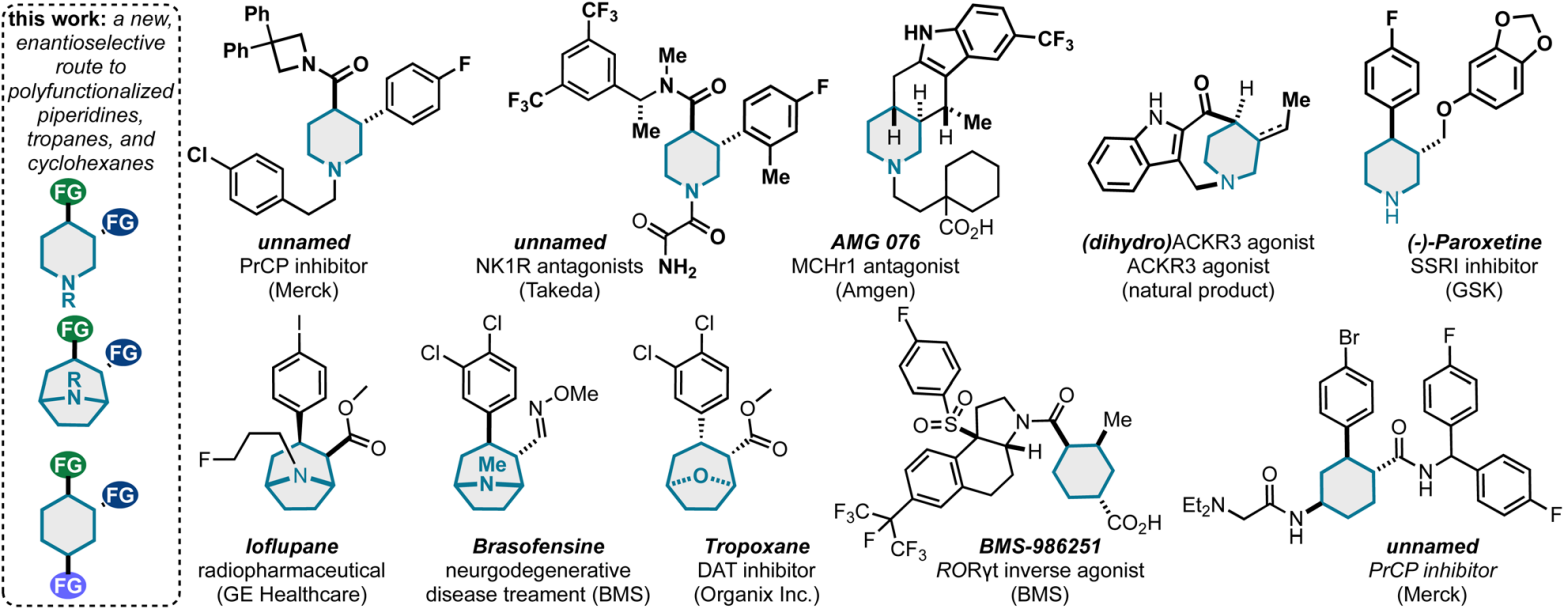
This method can uniquely populate piperidine, tropane, and cyclohexane pharmaceutical space.

We examined the metathesis reactivity of 6j, which resulted in ring-opening/cross-metathesis^57,58^ to 2,3,4,6-tetrasubstituted *N*-Boc-piperidine 9 (Scheme 5B). Under no circumstance was the formation of a product analogous to 8 observed. We speculate that the *N*-Boc group is either sterically blocking the initiation of ring-closing metathesis (RCM) or chelating the necessary ruthylidene to an unreactive conformer preventing RCM. Compound 7 was prepared *via* a telescoped asymmetric allylic alkylation/Cope rearrangement/NaBH_4_ reduction sequence. This scaffold, by a tandem ring-rearrangement metathesis (RRM)^59–61^/oxidative amidation, yielded highly substituted pyran-fused cyclopentene 8. Altogether, a rigid-bicyclic framework 8 decorated with various functional groups (alkenes, chloroarene, morpholine–amide) can be prepared efficiently from the alkylidenemalononitrile and the racemic allylic electrophile. The alkylidenemalononitrile moiety of tropanes 4v, 4w and 4z was surprisingly resistant to NaBH_4_ reduction. However, it was found that treatment of these scaffolds with ozone followed by NaBH_4_ yields products 10a–10c. It is proposed that the neutral alkene undergoes chemoselective ozonolysis, and upon NaBH_4_ reduction, the alcohol is generated which directs and allows the otherwise sterically blocked alkylidenemalononitrile reduction. Finally, it was found that substrates 10a–10c undergo intramolecular oxidative esterification to 11a–11c. Notably, while Hayashi has reported intermolecular conversion of malononitriles into amides and esters, this work establishes the first report of an intramolecular variant of this effective chemistry (Scheme 6).

## Conclusions

In conclusion, we have developed a simple strategy to convert abundant alkylidenemalononitriles and allylic electrophiles into stereochemically and functionally rich (hetero)cycloalkanes. This work relied on the development of an enantio- and diastereoselective AAA/[3,3] sequence, ultimately yielding a broad range of *trans*-3,4-disubstituted piperidines, *trans*-3,4-disubstituted tropanes, and a 1,2,4-trisubstituted cyclohexane. As shown in Scheme 5, heterocycles and carbocycles with these patterns are common to marketed drugs and drug leads/medicinal chemistry campaigns. The possibilities for this work are many fold and can be grouped into two categories: (1) it is anticipated that the synthetic chemistry findings, both the successes and challenges uncovered, will serve as key insight and inspiration for future method development related AAA/[3,3] transformations. Representative areas of future study should include dynamic kinetic asymmetric transformation based approaches to reactive 1,5-dienes, stereoselective deprotonation of *meso*-alkylidenemalononitriles, and access to wholly unique 1,5-dienes for enantioselective sigmatropic rearrangement. (2) It is anticipated that this chemistry as described can be utilized to access important molecules for therapeutic discovery. For example, designed analogs of the marketed drugs and lead molecules in Scheme 5 are within reach by this method. Further, because the described chemistry is both convergent and divergent from abundant starting materials, we believe this chemistry has potential to generate novel and complex sp3-rich libraries for hit-to-lead screening efforts. Continued fundamental and applied studies of AAA/[3,3] will be reported in due course.

## Data availability

The datasets supporting this article have been uploaded as part of the ESI.†

## Author contributions

AN and MDM were the primary researchers responsible for data collection and management. They also cowrote the manuscript. ANN and ES were secondary contributors to the research project. AJG conceptualized the project, oversaw the research, supervised the co-authors, and wrote the manuscript.

## Conflicts of interest

There are no conflicts to declare.

## Supplementary Material

SC-014-D2SC07021A-s001

SC-014-D2SC07021A-s002
