# Supplementary material for: Vicinal stereocenters via asymmetric allylic alkylation and Cope rearrangement: a straightforward route to functionally and stereochemically rich heterocycles
Source: Chem Sci. 2023 Feb 14;14(10):2755–62. doi: 10.1039/d2sc07021a (PMC9993902; doi:10.1039/d2sc07021a)
Supplement: SC-014-D2SC07021A-s001 [file SC-014-D2SC07021A-s001.pdf]

Electronic Supplementary Material (ESI) for Chemical Science.
This journal is © The Royal Society of Chemistry 2023

Supplementary Information

Vicinal Stereocenters via Asymmetric Allylic Alkylation and Cope rearrangement; A Straightforward Route to Functionally and Stereochemically Rich Heterocycles

Aleksandra Nilova,† Michael D. Mannchen,† Abdias N. Noel, Evgeniya Semenova, and Alexander J. Grenning*

Table of Contents

General considerations 3
Experimental procedures S3
Synthesis of alkylidenemalononitriles S3
Synthesis of allylic electrophiles S5
General procedure A. Synthesis of methyl carbonate protected allylic alcohols. S5
General procedure B. Synthesis of acetate protected allylic electrophiles. S5
Synthesis of electrophile ***rac*-2a-Boc**. S6
Synthesis of electrophile ***rac*-2i-Ac**. S6
Asymmetric allylic alkylation followed by Cope rearrangement S12
Reaction optimization S12
General procedure C S12
Reductive Cope rearrangement S27
General procedure D S27
Reactivity of *meso*-4-*tert*-butylcyclohexyllidenemalononitrile S29
Conjugate reduction of alkylidenemalononitriles **4** S29
General procedure E S29
Conjugate reduction of alkylidenemalononitrile **4dd**. S31
Oxidative amidation and esterification of malononitriles S32
General procedure F S32
Ring-opening metathesis: synthesis of compound **9** S36
2-pot protocol toward compound **8** S37
Telescoped sequence allylic alkylation/Cope rearrangement/reduction. S37
Ring rearrangement metathesis/oxidative amidation sequence. S38
Synthesis of lactones S38
General procedure G. Alkene ozonolysis followed by reduction. S38
X-ray Crystallography data S41
Crystal structure data for compound **4y** S42
Crystal structure data for compound **11b**. S51
References S59
NMR spectra S60
HPLC data S141

General considerations

Commercially available reagents and solvents were used without further purification. Allylic alkylation reactions were performed in flame-dried or oven-dried glassware under an argon or nitrogen atmosphere using dry, deoxygenated solvents. Solvents were dried by passage through an activated alumina column under argon. Alkylidenemalononitriles **1b – 1f**, **1h**, and **1i**, (*S,S*)-DACH-phenyl Trost ligand, and allylic electrophile ***rac*-2l** were prepared according to literature procedures.[1–8]

Reaction progress was monitored by thin-layer chromatography (TLC) and visualized by UV irradiation and $KMnO_4$ stain. Crude materials were purified by flash column chromatography on silica gel. ^1H, ^{13}C {^1H}, ^{19}F {^1H} NMR spectra were obtained at 298 K, unless otherwise stated, in $CDCl_3$, DMSO-*d*$_6$, or CD_3CN on 400 MHz, 500 MHz, or 600 MHz spectrometer and referenced to residual solvent peaks.[9] The chemical shifts are reported in ppm. The following notation is used: br – broad signal, s – singlet, d – doublet, t – triplet, q – quartet, p – pentet, m – multiplet, dd – doublet of doublets, dt – doublet of triplets, dq – doublet of quartet, dp – doublet of pentet, and ddd – doublet of doublet of doublets. Analytical chiral HPLC was performed utilizing Chiralpak AD-H, IA, or ID columns (4.6 mm x 150 mm) at 30 °C. HRMS data were obtained by electron spray ionization (ESI) with an ion trap mass analyzer or direct analysis in real time (DART).

Experimental procedures

Synthesis of alkylidenemalononitriles

Alkylidenemalononitriles **1b – 1f**, **1h**, and **1i** were prepared according to literature procedures.[1–6]

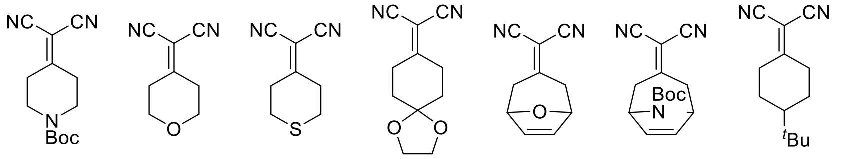

1b **1c** **1d** **1e** **1h** **1i** **1j**

Compound 1a

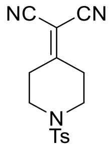

To a solution of 1-tosylpiperidin-4-one[10] (1.0 equiv., 7.37 g, 29 mmol) in toluene (100 mL, 0.3 M) malononitrile (1.2 equiv., 1.31 g, 35 mmol), ammonium acetate (1.3 equiv., 2.92 g, 38 mmol), and glacial acetic acid (5.0 equiv., 8.3 mL, 145 mmol) were added. The reaction vessel was equipped with a Dean-Stark apparatus and heated at reflux for 3 hours. The reaction mixture was concentrated by rotary evaporation. Crude material was dissolved in EtOAc and washed with $NaHCO_3$ (sat. aq.) to quench any remaining acetic acid. The aqueous layer was extracted twice with EtOAc. The combined organics were washed with brine, dried with anhydrous Na_2SO_4, and concentrated. Title compound was purified by column chromatography on silica gel with 1%-3% MeOH/DCM in hexanes as an eluent and obtained as a light-yellow solid in 55% yield (4.84 g, 16 mmol).

R*f* = 0.25 in 20% EtOAc in hexanes.

^{1}H NMR (400 MHz, CDCl$_3$) δ 7.64 (d, *J* = 8.3 Hz, 2H), 7.34 (d, *J* = 8.0 Hz, 2H), 3.25 (dd, *J* = 5.8 Hz, 4H), 2.84 (dd, *J* = 5.8 Hz, 4H), 2.43 (s, 3H).

^{13}C NMR (101 MHz, CDCl$_3$) δ 177.1, 144.6, 133.0, 130.2, 127.6, 110.9, 85.5, 46.0, 33.4, 21.7.

HRMS (ESI-TOF): [M-H]$^-$ Calculated for $C_{15}H_{14}N_3O_2S^-$ 300.0812; found 300.0811.

Compound 1f

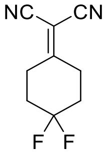

To a solution of 4,4-difluorocyclohexan-4-one (1 equiv., 1.00 g, 7.46 mmol) in toluene (75 mL, 1.0 M) malononitrile (1.1 equiv., 0.541 g, 8.20 mmol), ammonium acetate (0.5 equiv., 0.287 g, 3.73 mmol), and glacial acetic acid (1 equiv., 0.43 mL, 7.46 mmol) were added. The reaction vessel was equipped with a Dean-Stark apparatus and heated at reflux for 4 hours. The reaction mixture was concentrated by rotary evaporation. Crude material was dissolved in EtOAc and washed with 50 mL $NaHCO_3$ (sat. aq.) to quench any remaining acetic acid. The aqueous layer was extracted twice with 50 mL EtOAc. The combined organics were washed with brine, dried with anhydrous Na_2SO_4, and concentrated. Title compound was purified by silica gel column chromatography and obtained in 69% yield (0.943 g, 5.17 mmol) as a white solid.

$\mathbf{R}_{\boldsymbol{f}}$ = 0.52 in 20% EtOAc in hexanes.

^{1}H NMR (400 MHz, $CDCl_3$) δ 2.89 (dd, *J* = 8.0 Hz, 4H), 2.19 (tt, *J* = 12.9, 6.9 Hz, 4H).

^{13}C NMR (101 MHz, $CDCl_3$) δ 177.8, 120.7 (t, *J* = 242.2 Hz), 111.1, 85.8, 33.2 (t, *J* = 25.8 Hz), 30.1 (t, *J* = 5.5 Hz).

^{19}F NMR (377 MHz, $CDCl_3$) δ -100.0.

HRMS (ESI$^-$): [M – H]$^-$ calculated for $C_9H_7F_2N_2^-$ 181.0583; found 181.0588.

Compound 1g

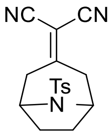

N-Ts protection of 8-azabicyclo[3.2.1]octan-3-one was performed according to the modified literature procedure.[11] To a suspension of 8-azabicyclo[3.2.1]octan-3-one hydrochloride (1.0 equiv., 1.62 g, 10.0 mmol) in DCM (20 mL), N,N-diisopropylethylamine (2.0 equiv., 3.5 mL, 20.0 mmol) was added at stirring followed by slow addition of *p*-toluenesulfonyl chloride (1.01 equiv., 1.93 g, 10.1 mmol). The reaction mixture was stirred at room temperature for 3 hours. The organic layer was washed with saturated aqueous solution of NH_4Cl (2×) and brine and dried over anhydrous Na_2SO_4. Solvent was evaporated under reduced pressure, and the solid residue was washed with Et_2O and dried by suction filtration providing a white solid (2.51 g, 9.0 mmol) that was used without further purification to synthesize compound **1g** according to the modified literature procedure.[6] The obtained ketone (1 equiv., 2.51 g, 9.0 mmol), malononitrile (1 equiv., 0.59 g, 9.0 mmol), ammonium acetate (0.5 equiv., 0.35 g, 4.5 mmol) and acetic acid (1 equiv., 0.51 mL, 9 mmol) were dissolved in toluene (9 mL) in a round-bottom flask equipped with a stir bar, Dean-Stark apparatus, and a condenser. The reaction mixture was refluxed for 3 hours, removed from heat, and the solvent was evaporated under reduced pressure. The contents of the flask were transferred to a fritted funnel with EtOAc, washed with water (3×) and EtOAc leaving behind a grey to brown solid. EtOAc/water mixture was transferred to a separatory funnel; the organic layer was separated and washed with saturated aqueous solution of $NaHCO_3$ and brine. The organic layer was dried over anhydrous Na_2SO_4, filtered, and concentrated under reduced pressure to

provide a solid residue. The collected solids were combined and recrystallized from EtOH to provide the desired product in 78% yield (2.54 g, 7.8 mmol) as a pale-yellow solid.

R*f* = 0.31 in 30% EtOAc in hexanes.

^{1}H NMR (600 MHz, $CDCl_3$) δ 7.75 (d, *J* = 8.3 Hz, 2H), 7.32 (d, *J* = 8.3 Hz, 2H), 4.48 – 4.42 (m, 1H), 2.98 – 2.88 (m, 4H), 2.44 (s, 3H), 1.72 – 1.66 (m, 2H), 1.50 – 1.42 (m, 2H).

^{13}C NMR (101 MHz, $CDCl_3$) δ 177.3, 144.5, 136.4, 130.1, 127.3, 110.9, 88.6, 56.2, 41.7, 29.0, 21.7.

HRMS (ESI$^+$): $[M + Na]^+$ calculated for $C_{17}H_{17}N_3O_2SNa^+$ 350.0934; found 350.0942.

Synthesis of allylic electrophiles

Compound **4l** was prepared according to the literature procedure and the spectral data is consistent with the previously reported.[8]

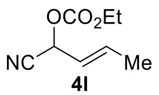

4l

General procedure A. Synthesis of methyl carbonate protected allylic alcohols.

<u>Wittig reaction/reduction sequence</u>

To a solution of (hetero)aryl aldehyde (1 equiv.) in DCM (0.5 M) or alkyl aldehyde (1 equiv.) in toluene (0.67 M) (acetylmethylene)triphenylphosphorane (1.1 equiv.) was added at stirring and the reaction mixture was allowed to react overnight at room temperature or at 100 °C correspondingly. Methanol (0.33 M) was added, and the solution was cooled to 0 °C in an ice-water bath. $NaBH_4$ (1.0 equiv.) was added in one portion, and the reaction flask was raised from the ice-water bath. After 1 hour the reaction was quenched with saturated aqueous solution of ammonium chloride. The biphasic mixture was transferred to a separatory funnel, organic layer was separated, and aqueous layer was extracted with EtOAc (2×). Combined organic layers were washed with brine, dried over Na_2SO_4, and filtered. Solvent was evaporated under reduced pressure to provide crude allylic alcohols that were used without further purification unless otherwise stated.

<u>Methyl carbonate protection of allylic alcohols</u>

Crude allylic alcohol was dissolved in DCM (0.5 M), pyridine (3 equiv.) was added at stirring, and the flask was placed on an ice-water bath. Methyl chloroformate (3 equiv.) was added dropwise via addition funnel and the mixture was stirred overnight. The contents of the flask were transferred to a separatory funnel, washed with saturated aqueous solution of NH_4Cl (2×), and brine. The combined organic phases were dried over anhydrous Na_2SO_4, filtered, and concentrated under reduced pressure. The crude product was further purified by flash column chromatography on silica gel using EtOAc in hexanes as an eluent to provide the desired product.

General procedure B. Synthesis of acetate protected allylic electrophiles.

Allylic alcohols were prepared according to Wittig-reaction/reduction sequence described in General procedure A and purified via column chromatography on silica gel with EtOAc in hexanes as eluent.

<u>Acetate protection of allylic alcohols</u>[12]

An allylic alcohol (1 equiv.) was dissolved in dry DCM (0.5 M) in a flame-dried Schlenk flask and cooled in an ice-water bath. DMAP (0.1 equiv.) and Et_3N (2 equiv.) were added, and the reaction was stirred for 20 minutes. Acetic anhydride (2 equiv.) was added dropwise, and the reaction was allowed to stir warming to room temperature. Upon

completion per TLC, the reaction was quenched with DI water (2 x reaction volume) and transferred to a separatory funnel. The aqueous layer was extracted three times with DCM, washed with brine, dried with sodium sulfate, filtered, and concentrated under reduced pressure. The crude material was purified using silica gel column chromatography.

Synthesis of electrophile *rac*-2a-Boc.

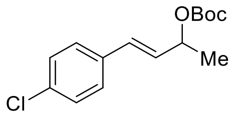

Prepared according to the literature procedure.[13] Alcohol **SI-1** (1 equiv., 0.91 mg, 5.0 mmol) was dissolved in THF (10 mL, 0.2 M) in a flame-dried Schlenk flask under a nitrogen atmosphere. The reaction was cooled in an ice-water bath, and *n*BuLi (1.6 M in THF, 1.1 equiv., 3.43 ml, 5.5 mmol) was added slowly. The mixture was stirred for 10 minutes, and Boc_2O (1.2 equiv., 1.38 mL, 6 mmol) was added dropwise. Upon completion per TLC, the reaction was quenched with sat. aq. NH_4Cl (25 mL), and the aqueous layer was extracted with EtOAc (2 x 25 mL). The combined organic layers were washed with brine, dried with $NaSO_4$, and concentrated under reduced pressure. The crude material was purified with silica gel column chromatography using EtOAc in hexanes as an eluent. The desired compound was obtained in 92% yield (1.30 g, 4.6 mmol) as a colorless oil.

R_f = 0.80 in 20% EtOAc in hexanes.

^{1}H NMR (400 MHz, $CDCl_3$) δ 7.32 – 7.26 (m, 4H), 6.57 (d, *J* = 16.0 Hz, 1H), 6.17 (dd, *J* = 16.0, 6.9 Hz, 1H), 5.43 – 5.23 (m, 1H), 1.49 (s, 9H), 1.44 (d, *J* = 6.5 Hz, 3H).

^{13}C NMR (101 MHz, $CDCl_3$) δ 152.9, 134.9, 133.7, 130.6, 129.3, 128.8, 127.9, 82.2, 73.9, 27.9, 20.5.

HRMS (DART, 300 °C): $[M + NH_4]^+$ calculated for $C_{15}H_{23}ClNO_3^+$ 300.1361; found 300.1355.

Synthesis of electrophile *rac*-2i-Ac.

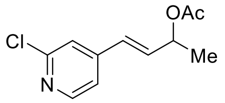

To a solution of 2-chloroisonicotinaldehyde (1 equiv., 2.48 g, 17.2 mmol) in DCM (0.5 M, 34 mL) (acetylmethylene)triphenylphosphorane (1.1 equiv., 5.85 g, 18.9 mmol) was added at stirring and the reaction mixture was left to react overnight at room temperature. Methanol (0.33 M, 53 mL) was added, and the solution was cooled to 0 °C in an ice-water bath. $NaBH_4$ (3.0 equiv.,1.95 g, 51.6 mmol) was added in one portion, and the reaction flask was warmed to room temperature. After 1 hour the reaction was quenched with saturated aqueous solution of ammonium chloride. The biphasic mixture was transferred to a separatory funnel, organic layer was separated, and aqueous layer was extracted with EtOAc (2×). The combined organic layers were washed with brine, dried with anhydrous Na_2SO_4, and concentrated under reduced pressure. The crude alcohol was dissolved in DCM (0.33 M, 53 mL) and cooled in an ice-water bath at stirring. Pyridine (2.5 equiv., 3.47 g, 43.0 mmol) was added followed by dropwise addition of acetyl chloride (1.4 equiv. 1.93 g, 24.1 mmol). The reaction was allowed to warm to room temperature and upon completion per TLC analysis the reaction was quenched with DI water (100 mL). The organic layer was separated, and the aqueous layer was extracted with DCM (50 mL). The combined organic layers were washed with brine, dried with anhydrous Na_2SO_4, and concentrated under reduced pressure. The compound was purified using silica gel column chromatography and obtained in 89% yield (3.52 g, 15.6 mmol) as a light-yellow oil.

R_f = 0.33 in 20% EtOAc in hexanes.

^{1}H NMR (400 MHz, CDCl$_3$) δ 8.31 (d, *J* = 5.2 Hz, 1H), 7.28 (s, 1H), 7.16 (dd, *J* = 5.2, 1.3 Hz, 1H), 6.47 (d, *J* = 6.5 Hz, 1H), 6.39 (ddd, *J* = 24.3, 16.0, 5.8 Hz, 1H), 5.51 (p, *J* = 6.4 Hz, 1H), 2.09 (d, *J* = 1.4, 3H), 1.41 (d, *J* = 6.6, 3H).

^{13}C NMR (101 MHz, CDCl$_3$) δ 170.3, 152.3, 150.0, 147.1, 135.5, 127.6, 121.6, 119.9, 70.1, 21.4, 20.2.

HRMS (EST-TOF): [M+H]$^+$ Calculated for $C_{11}H_{13}ClNO_2^+$ 226.0629. Found 226.0639.

Compound SI-1

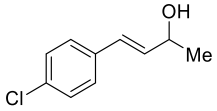

Prepared according to the Wittig reaction (DCM, room temperature)/reduction sequence of the General procedure A from 4-chlorobenzaldehyde on 20 mmol scale and obtained in 86% yield (3.15 g, 17 mmol) as a white solid. The spectral data is consistent with the previously reported.[14]

R$_f$ = 0.30 in 20% EtOAc in Hexanes

^{1}H NMR (600 MHz, CDCl$_3$) δ 7.33 – 7.27 (m, 4H), 6.53 (d, *J* = 15.9 Hz, 1H), 6.24 (dd, *J* = 15.9, 6.3 Hz, 1H), 4.49 (pd, *J* = 6.3, 1.0 Hz, 1H), 1.62 (br, 1H), 1.37 (d, *J* = 6.3 Hz, 3H).

^{13}C NMR (101 MHz, CDCl$_3$) δ 135.3, 134.3, 133.4, 128.9, 128.4, 127.8, 68.9, 23.5.

Compound SI-2

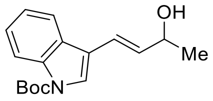

Prepared according to the Wittig reaction (toluene, 100 °C)/reduction sequence of General procedure A from *N*-Boc indole-3-carboxaldehyde[15] on 9.4 mmol scale and obtained in 67% yield (1.81 g, 6.3 mmol) as a yellow oil.

R$_f$ = 0.21 in 20% EtOAc in hexanes.

^{1}H NMR (400 MHz, CDCl$_3$) δ 8.18 (d, *J* = 8.3 Hz, 1H), 7.79 – 7.72 (m, 1H), 7.58 (s, 1H), 7.34 (ddd, *J* = 8.3, 7.2, 1.3 Hz, 1H), 7.26 (ddd, *J* = 8.3, 6.9, 1.1 Hz, 1H), 6.64 (dd, *J* = 16.2, 1.1 Hz, 1H), 6.35 (dd, *J* = 16.1, 6.3 Hz, 1H), 4.50 (pd, *J* = 6.3, 1.2 Hz, 1H), 2.67 (s, 1H), 1.67 (s, 9H), 1.42 (d, *J* = 6.4 Hz, 3H).

^{13}C NMR (101 MHz, CDCl$_3$) δ 149.6, 135.9, 134.1, 128.7, 124.6, 123.7, 122.8, 120.4, 119.9, 118.2, 115.3, 83.8, 69.1, 28.1, 23.5.

HRMS (ESI$^+$): [M – OH]$^+$ calculated for $C_{17}H_{20}NO_2^+$ 270.1489; found 270.1497.

Compound *rac*-2a

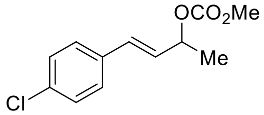

Prepared according to the General procedure A on 20 mmol scale using 5 equiv. of methyl chloroformate and obtained in 84% yield (4.04 g, 17 mmol) as a pale-yellow oil.

R$_f$ = 0.52 in 20% EtOAc in hexanes.

^{1}H NMR (600 MHz, CDCl$_3$) δ 7.33 – 7.26 (m, 4H), 6.60 (d, *J* = 16.0 Hz, 1H), 6.17 (dd, *J* = 16.0, 6.9 Hz, 1H), 5.40 – 5.32 (m, 1H), 3.79 (s, 3H), 1.46 (d, *J* = 6.4 Hz, 3H).

^{13}C NMR (101 MHz, CDCl$_3$) δ 155.3, 134.8, 133.9, 131.1, 128.9(1), 128.8(9), 128.0, 75.2, 54.8, 20.6.

HRMS (DART$^+$): [M-OCO$_2$Me]$^+$ calculated for $C_{10}H_{10}Cl^+$ 165.0466; found 165.0471.

Compound *rac*-2a-Ac

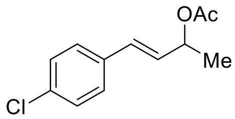

Prepared according to the General procedure B on 2.4 mmol scale and obtained in 85% yield (0.46 g, 2.0 mmol) as a colorless oil. The spectral data is consistent with the previously reported.[12]

R*f* = 0.63 in 20% EtOAc in hexanes.

^{1}H NMR (400 MHz, CDCl$_3$) δ 7.27 – 7.16 (m, 6H), 6.48 (d, *J* = 16.0 Hz, 1H), 6.09 (dd, *J* = 16.0, 6.7 Hz, 1H), 5.44 (p, *J* = 6.5 Hz, 1H), 2.00 (s, 3H), 1.33 (d, *J* = 6.5 Hz, 3H).

^{13}C NMR (101 MHz, CDCl$_3$) δ 170.4, 135.0, 133.7, 130.4, 129.6, 128.9, 127.9, 70.9, 21.5, 20.4.

HRMS (DART, 300 °C): [M+NH$_4$] calculated for $C_{12}H_{17}ClNO_2$ 242.0942; found 242.0932.

Compound *rac*-2b

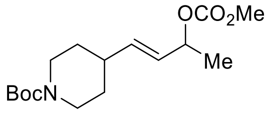

Prepared according to the General procedure A on 5.0 mmol scale and obtained in 90% yield (1.40 g, 4.5 mmol) as a colorless oil.

R*f* = 0.36 in 30% EtOAc in hexanes.

^{1}H NMR (400 MHz, CDCl$_3$) δ 5.69 (dd, *J* = 15.6, 6.4 Hz, 1H), 5.47 (ddd, *J* = 15.6, 6.9, 1.3 Hz, 1H), 5.15 (dq, appears as p, *J* = 6.5 Hz, 1H), 4.07 (br, 2H), 3.76 (s, 3H), 2.80 – 2.61 (m, 2H), 2.16 – 2.04 (m, 1H), 1.68 – 1.61 (m, 2H), 1.44 (s, 9H), 1.34 (d, *J* = 6.5 Hz, 3H), 1.29 – 1.19 (m, 2H).

^{13}C NMR (101 MHz, CDCl$_3$) δ 155.1, 154.9, 137.5, 127.7, 79.4, 75.3, 54.6, 43.7, 38.5, 31.4, 28.5, 20.5.

HRMS (ESI$^+$): [M+Na]$^+$ calculated for $C_{16}H_{27}NO_5Na^+$ 336.1781; found 336.1789.

Compound *rac*-2c

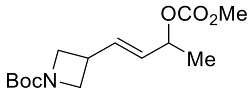

Prepared according to the General procedure A on 1.45 mmol scale using 2 equiv. of pyridine and 2 equiv. of methyl chloroformate and obtained in 57% yield (0.240 g, 0.84 mmol) as a colorless oil.

R*f* = 0.43 in 30% EtOAc in hexanes.

^{1}H NMR (600 MHz, CDCl$_3$) δ 5.91 (ddd, *J* = 15.5, 8.1, 1.1 Hz, 1H), 5.55 (ddd, *J* = 15.5, 6.5, 1.1 Hz, 1H), 5.18 (ddq, appears as dp, *J* = 6.5, 1.1 Hz, 1H), 4.07 (ddd, appears as dt, *J* = 8.5, 1.7 Hz, 2H), 3.77 (s, 3H), 3.76 – 3.68 (m, 2H), 3.22 – 3.12 (m, 1H), 1.43 (s, 9H), 1.36 (d, *J* = 6.5 Hz, 3H).

^{13}C NMR (101 MHz, CDCl$_3$) δ 156.4, 155.2, 133.5, 130.5, 79.6, 74.7, 54.7, 54.6, 54.5, 31.0, 28.5, 20.4.

HRMS (ESI$^+$): $[M + Na]^+$ calculated for $C_{14}H_{26}NO_5Na^+$ 308.1468; found 308.1476.

Compound *rac*-2d

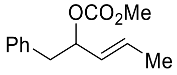

Prepared from the corresponding allylic alcohol[16] (335 mg, 2.06 mmol) using General procedure A on a 2.06 mmol and obtained in 37% yield (169 mg, 0.77 mmol) as a colorless oil.

R$_f$ = 0.44 in 10% EtOAc in hexanes.

^{1}H NMR (400 MHz, CDCl$_3$) δ 7.33 – 7.27 (m, 2H), 7.24 – 7.17 (m, 3H), 5.80 – 5.67 (m, 1H), 5.47 (ddd, *J* = 15.3, 7.6, 1.6 Hz, 1H), 5.22 (q, *J* = 7.1 Hz, 1H), 3.72 (s, 3H), 3.00 (dd, *J* = 13.8, 7.4 Hz, 1H), 2.88 (dd, *J* = 13.8, 6.2 Hz, 1H), 1.67 (d, *J* = 6.5, 1.5 Hz, 3H).

^{13}C NMR (101 MHz, CDCl$_3$) δ 155.2, 137.0, 130.7, 129.7, 128.4, 128.4, 126.7, 79.7, 54.6, 41.3, 17.8.

HRMS (DART$^+$): $[M+NH_4]^+$ Calculated for $C_{13}H_{20}NO_3$ 243.0992. Found 243.0990.

Compound *rac*-2e

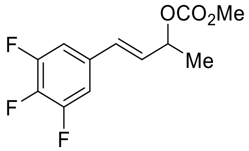

Prepared according to the General procedure A on 2 mmol scale using 3 equiv. of methyl chloroformate and obtained in 81% yield (0.42 g, 1.6 mmol) as a colorless oil.

R$_f$ = 0.52 in 20% EtOAc in hexanes.

^{1}H NMR (600 MHz, CDCl$_3$) δ 7.02 – 6.94 (m, 2H), 6.49 (d, *J* = 15.9 Hz, 1H), 6.12 (dd, *J* = 15.9, 6.6 Hz, 1H), 5.34 (ddq, appears as dp, *J* = 6.6, 1.2 Hz, 1H), 3.79 (s, 3H), 1.46 (d, *J* = 6.6 Hz, 3H).

^{13}C NMR (101 MHz, CDCl$_3$) δ 155.2, 151.5 (dd, *J* = 249.5, 4.2 Hz), 151.4 (dd, *J* = 249.6, 4.1 Hz), 139.5 (dt, *J* = 252.7, 15.6 Hz), 132.6 (dt, *J* = 7.8, 4.7 Hz), 130.7 (d, *J* = 2.6 Hz), 129.3 (q, *J* = 2.4 Hz), 110.8 – 110.4 (m), 74.5, 54.9, 20.4.

^{19}F NMR (377 MHz, CDCl$_3$) δ -134.4 (d, *J* = 20.3 Hz), -160.8 (t, *J* = 20.3 Hz).

HRMS (DART$^+$): $[M-OCO_2Me]^+$ calculated for $C_{10}H_9F_2^+$ 185.0573; found 185.0594.

Compound *rac*-2f

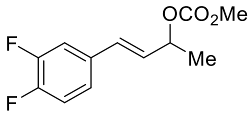

Prepared according to the General procedure A on 5.0 mmol scale and obtained in 65% yield (0.790 g, 3.2 mmol) as a colorless oil.

R*f* = 0.69 in 30% EtOAc in hexanes.

^{1}H NMR (400 MHz, CDCl$_3$) δ 7.23 – 7.16 (m, 1H), 7.13 – 7.03 (m, 2H), 6.55 (d, *J* = 15.9 Hz, 1H), 6.11 (dd, *J* = 15.9, 6.8 Hz, 1H), 5.40 – 5.30 (m, 1H), 3.79 (s, 3H), 1.46 (d, *J* = 6.5 Hz, 3H).

^{13}C NMR (101 MHz, CDCl$_3$) δ 155.2, 150.6 (dd, *J* = 246.9, 11.8 Hz), 150.2 (dd, *J* = 248.9, 12.2 Hz), 133.5 (dd, *J* = 6.0, 4.0 Hz), 130.3 (dd, *J* = 1.9 Hz), 129.4 (d, *J* = 2.5 Hz), 123.1 (dd, *J* = 6.2, 3.5 Hz), 117.5 (d, *J* = 17.3 Hz), 115.1 (d, *J* = 17.6 Hz), 74.9, 54.8, 20.5.

^{19}F NMR (565 MHz, CDCl$_3$) δ -137.7 (d, *J* = 20.7 Hz), -138.3 (d, *J* = 20.7 Hz).

HRMS (DART$^+$): [M-OCO$_2$Me]$^+$ calculated for $C_{10}H_9F_2^+$ 167.0667; found 167.0663.

Compound *rac*-2g

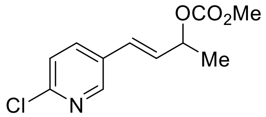

Prepared according to the General procedure A on 10 mmol scale using 5 equiv. of pyridine and 10 equiv. of methyl chloroformate and obtained in 81% yield (1.95 g, 8.1 mmol) as a colorless oil.

R*f* = 0.47 in 30% EtOAc in hexanes.

^{1}H NMR (400 MHz, CDCl$_3$) δ 8.35 (d, *J* = 2.5 Hz, 1H), 7.66 (dd, *J* = 8.5, 2.5 Hz, 1H), 7.27 (d, overlaps with solvent peak at 7.26, *J* = 8.5 Hz, 1H), 6.59 (d, *J* = 16.1 Hz, 1H), 6.24 (dd, *J* = 16.1, 6.5 Hz, 1H), 5.36 (ddq, appears as dp, *J* = 6.5, 1.2 Hz, 1H), 3.79 (s, 3H), 1.47 (d, *J* = 6.5 Hz, 3H).

^{13}C NMR (101 MHz, CDCl$_3$) δ 155.1, 152.3, 150.0, 146.9, 134.6, 128.1, 121.6, 119.9, 74.1, 54.9, 20.3.

HRMS (ESI$^+$): [M + H]$^+$ calculated for $C_{11}H_{13}ClNO_3^+$ 242.0579; found 242.0579.

Compound *rac*-2h

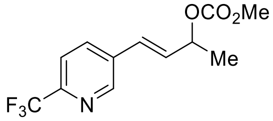

Prepared according to the General procedure A on 5.7 mmol scale using 5 equiv. of pyridine and 10 equiv. of methyl chloroformate and obtained in 52% yield (0.82 g, 3.0 mmol) as a colorless oil.

R*f* = 0.68 in 40% EtOAc in hexanes.

^{1}H NMR (400 MHz, CDCl$_3$) δ 8.70 (d, *J* = 2.2 Hz, 1H), 7.85 (dd, *J* = 8.2, 2.2 Hz, 1H), 7.64 (d, *J* = 8.2 Hz, 1H), 6.68 (d, *J* = 16.1 Hz, 1H), 6.38 (dd, *J* = 16.1, 6.5 Hz, 1H), 5.40 (ddq, appears as dp, *J* = 6.5, 1.3 Hz, 1H), 3.80 (s, 3H), 1.49 (d, *J* = 6.5 Hz, 3H).

^{13}C NMR (101 MHz, CDCl$_3$) δ 155.1, 148.6, 147.3 (q, *J* = 34.8 Hz), 134.8, 134.4, 133.3, 127.0, 121.6 (q, *J* = 273.9 Hz), 120.5 (q, *J* = 2.8 Hz), 74.4, 54.9, 20.4.

^{19}F NMR (377 MHz, CDCl$_3$) δ -67.8.

HRMS (ESI$^+$): $[M+H]^+$ calculated for $C_{12}H_{13}F_3NO_3^+$ 276.0842; found 276.0852.

Compound *rac*-2i

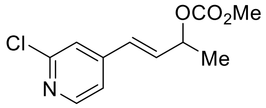

Prepared according to the General procedure A on 7.0 mmol scale using 5 equiv. of pyridine and 10 equiv. of methyl chloroformate and obtained in 65% yield (1.10 g, 4.5 mmol) as a colorless oil.

R*f* = 0.58 in 40% EtOAc in hexanes.

^{1}H NMR (600 MHz, $CDCl_3$) δ 8.31 (dd, *J* = 5.2, 0.7 Hz, 1H), 7.30 – 7.26 (m, 1H), 7.16 (dd, *J* = 5.2, 1.5 Hz, 1H), 6.54 (dt, *J* = 16.0, 0.8 Hz, 1H), 6.42 (dd, *J* = 16.0, 6.5 Hz, 1H), 5.37 (ddq, appears as dp, *J* = 6.5, 1.2 Hz, 1H), 3.80 (s, 3H), 1.47 (d, *J* = 6.5 Hz, 3H).

^{13}C NMR (101 MHz, $CDCl_3$) δ 155.1, 152.3, 150.0, 146.9, 134.6, 128.1, 121.6, 119.9, 74.1, 54.9, 20.3.

HRMS (ESI$^+$): $[M+H]^+$ calculated for $C_{11}H_{13}ClNO_3^+$ 242.0579; found 242.0589.

Compound *rac*-2j

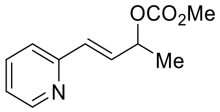

Prepared according to the General procedure A on 11.2 mmol scale using 5 equiv. of pyridine and 10 equiv. of methyl chloroformate and obtained in 42% yield (0.97 g, 4.7 mmol) as a colorless oil.

R*f* = 0.39 in 40% EtOAc in hexanes.

^{1}H NMR (400 MHz, $CDCl_3$) δ 8.61 – 8.50 (m, 1H), 7.63 (ddd, appears as dt, *J* = 7.6, 1.9 Hz, 1H), 7.28 (m, overlaps with solvent peak at 7.26, 1H), 7.14 (ddd, *J* = 7.6, 4.8, 1.2 Hz, 1H), 6.80 – 6.65 (m, 2H), 5.47 – 5.37 (m, 1H), 3.78 (s, 3H), 1.48 (d, *J* = 6.5 Hz, 3H).

^{13}C NMR (101 MHz, $CDCl_3$) δ 155.2, 154.7, 149.7, 136.7, 132.7, 131.2, 122.7, 122.3, 74.7, 54.8, 20.5.

HRMS (ESI$^+$): $[M + H]^+$ calculated for $C_{11}H_{14}NO_3^+$ 208.0968; found 208.0976.

Compound *rac*-2k-Ac

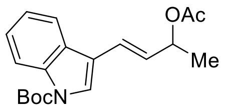

Prepared according to the General procedure B on a 3.1 mmol scale and obtained in 77% yield (0.80 g, 2.4 mmol) as a yellow oil.

R*f* = 0.70 in 20% EtOAc in hexanes.

^{1}H NMR (400 MHz, $CDCl_3$) δ 8.17 (d, *J* = 8.2 Hz, 1H), 7.77 (dt, *J* = 7.8, 1.0 Hz, 1H), 7.63 (s, 1H), 7.34 (ddd, *J* = 8.4, 7.2, 1.4 Hz, 1H), 7.29 (dd, *J* = 7.7, 1.2 Hz, 1H), 6.72 (dt, *J* = 16.1, 0.9 Hz, 1H), 6.28 (dd, *J* = 16.1, 6.9 Hz, 1H), 5.55 (pd, *J* = 6.5, 1.2 Hz, 1H), 2.09 (s, 3H), 1.67 (s, 9H), 1.45 (d, *J* = 6.5 Hz, 3H).

^{13}C NMR (101 MHz, CDCl$_3$) δ 170.5, 149.6, 136.1, 129.2, 128.6, 124.8, 124.5, 123.3, 123.0, 120.0, 117.9, 115.5, 84.0, 71.6, 28.3, 21.6, 20.6.

HRMS (DART, 330 °C): $[M+NH_4]^+$ calculated for $C_{19}H_{27}N_2O^+$ 347.1965; found 347.1967.

Asymmetric allylic alkylation followed by Cope rearrangement

Reaction optimization

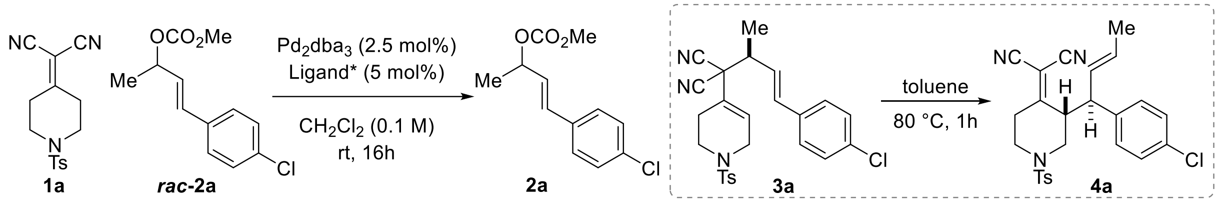

To an oven-dried 10 mL Schlenk flask equipped with a magnetic stir bar $Pd_2(dba)_3$ (2.5 mol%, 0.0046 g, 0.005 mmol), (*S,S*)-DACH-phenyl Trost ligand (5 mol%, 0.0069 g, 0.01 mmol), and alkylidenemalononitrile **1a** (1 equiv., 0.060 g, 0.2 mmol) were added. The flask was sealed with a rubber septum, and the contents were placed under N_2 atmosphere. Anhydrous DCM (2 mL) was added via syringe, and the solution was stirred for 15 minutes (solution turned from purple to yellow-brown). An allylic electrophile ***rac*-2a** (2.5 equiv., 0.120 g, 0.5 mmol) was added via syringe, and the contents were left to react overnight (16 hours) at room temperature. The resulting mixture was concentrated under reduced pressure, and unreacted compound **2a** was isolated by silica gel column chromatography (0.048 g, 0.2 mmol; **HPLC**: Chiralpak AD-H, *i*-PrOH:hexanes = 2:98, flow rate = 1 mL/min, UV = 254 nm, t_R1 = 3.72 min (major) and t_R2 = 4.69 min (minor), er = 92:8).

Compound **3a** was dissolved in toluene (2 mL), and the solution was heated at stirring at 80 °C for 1 hour. The Cope rearrangement product **4a** was isolated by silica gel column chromatography (see details below).

Table S1. Specific reaction conditions for the General procedure C.

	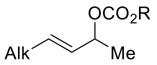 **rac-2b - 2d, 2l**	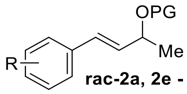 **rac-2a, 2e - 2f**	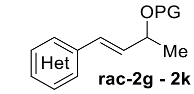 **rac-2g - 2k**
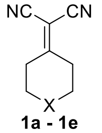 **1a - 1e**	**[AAA]** DCM (0.5 M), r.t.<br>**[3,3]** toluene (0.1 M), 110 °C, 4 hr	**[AAA]** DCM (0.1 M), r.t.<br>**[3,3]** toluene (0.1 M), 80 °C, 1 hr	**[AAA]** DCM (0.1 M), r.t.<br>**[3,3]** toluene (0.1 M), 80 °C, 1 hr
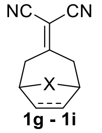 **1g - 1i**	**[AAA]** DCM (0.5 M), r.t.<br>**[3,3]** toluene (0.1 M), 80 °C, 1 hr	**[AAA]** DCM (0.1 M), 4 °C<br>**[3,3]** DCM (0.1 M), 40 °C, 1 hr	**[AAA]** DCM (0.1 M), 4 °C<br>**[3,3]** toluene (0.1 M), 50 °C, 1 hr

General procedure C.

Specific reaction conditions are outlined in Table S1.

Asymmetric allylic alkylation [AAA]: To an oven-dried Schlenk flask equipped with a stir bar, $Pd_2(dba)_3$ (2.5 mol%), (*S,S*)-DACH-phenyl Trost ligand (5 mol%), and alkylidenemalononitrile **1** (1 equiv.) were added, and the contents were placed under N_2 atmosphere. Anhydrous DCM (0.5 M or 0.1 M) was added via syringe, and the solution was stirred for 15 minutes (solution turned from purple to yellow-brown). An allylic electrophile **rac-2** (2.5 equiv. or 2.2

equiv. with bicyclic nucleophiles) was added via syringe, and the contents were left to react overnight (16 – 18 hours) at an indicated temperature. For substrates **1a – 1e** the reaction mixture was filtered through a silica plug and concentrated under reduced pressure.

Cope rearrangement [3,3]: The solvent switch to toluene (0.1 M) was performed where applicable and the mixture was heated at stirring at indicated temperature for indicated time. The desired product was isolated by silica gel column chromatography. Diastereomeric ratio (dr) was determined based on ^{1}H NMR and found to be more than 20:1 unless otherwise stated.

The racemic compounds were prepared under the same reaction conditions using corresponding allylic electrophiles (1-1.5 equiv.) and $Pd(PPh_3)_4$ as a catalyst without additional use of ligand.

Note: absolute stereochemistry of compounds **4** was assigned via analogy to **4y** (determined by X-ray crystallography).

Compound 4a

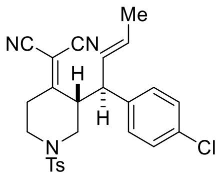

Prepared according to the General procedure C on 0.20 mmol scale from **1a** and ***rac*-2a** and obtained in 67% yield (62.8 mg, 0.13 mmol, er = 94:6) as a white solid; from **1a** and ***rac*-2a-Ac** with addition of K_3PO_4 (3 equiv., 127.4 mg, 0.6 mmol) and obtained in 74% yield (69.2 mg, 0.15 mmol, er = 95:5); from **1a** and ***rac*-2a-Ac** with addition of Et*i*Pr_2N (3 equiv., 69 mg, 0.6 mmol) and obtained in 55% yield (51.6 mg, 0.11 mmol, er = 97:3).

R*f* = 0.28 in 20% EtOAc in hexanes.

^{1}H NMR (400 MHz, $CDCl_3$) δ 7.53 (d, *J* = 8.3 Hz, 2H), 7.37 (d, *J* = 8.5 Hz, 2H), 7.33 – 7.27 (m, 4H), 5.63 – 5.46 (m, 2H), 4.17 – 4.07 (m, 1H), 3.86 – 3.77 (m, 1H), 3.57 (d, *J* = 12.1 Hz, 1H), 3.18 (d, *J* = 10.7 Hz, 1H), 2.98 (d, *J* = 14.9 Hz, 1H), 2.74 (ddd, *J* = 14.8, 12.4, 6.3 Hz, 1H), 2.41 (s, 3H), 2.36 – 2.21 (m, 1H), 2.15 (dd, *J* = 12.1, 3.2 Hz, 1H), 1.65 (d, *J* = 4.9 Hz, 3H).

^{13}C NMR (101 MHz, $CDCl_3$) δ 179.3, 144.6, 139.3, 133.5, 132.1, 130.1, 129.7, 129.5, 129.1, 128.9, 127.7, 111.3, 110.8, 86.7, 49.9, 48.8, 47.8, 46.7, 30.9, 21.6, 17.9.

HRMS (ESI-TOF): $[M+Na]^+$ calculated for $C_{25}H_{24}ClNaN_3O_2S^+$ 488.1170; found 488.1187.

HPLC: (Chiralpak AD-H, *i*-PrOH:hexanes = 5:95, flow rate = 0.5 mL/min, UV = 254 nm) t_R1 = 13.59 min (minor) and t_R2 = 15.50 min (major), er = 94:6.

Compound 4b

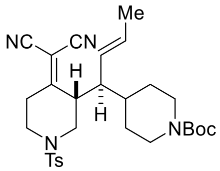

Prepared according to the General procedure C using **1a** and ***rac*-2b** on a 0.2 mmol scale and obtained in 81% yield (86.8 mg, 0.161 mmol, er = 99:1) as a white solid; on a 3 mmol scale the compound was obtained in 85% yield (1.38

g, 2.56 mmol, er = 98.3:1.7); on a 3 mmol scale with 0.5 mol% ligand and 0.25 mol% Pd_2dba_3 loading, the compound was obtained in 69% yield (1.12 g, 2.08, er = 95:5).

R*f* = 0.30 in 30% EtOAc in hexanes.

^{1}H NMR (400 MHz, CD_3CN) δ 7.66 (d, *J* = 8.2 Hz, 2H), 7.42 (d, *J* = 8.2 Hz, 2H), 5.45 (dq, *J* = 15.2, 6.4 Hz, 1H), 5.14 – 5.02 (m, 1H), 4.09 (t, *J* = 16.4 Hz, 2H), 4.01 – 3.89 (m, 2H), 3.01 (d, *J* = 10.6 Hz, 1H), 2.81 (d, *J* = 14.9 Hz, 1H), 2.72 – 2.56 (m, 3H), 2.54 – 2.35 (m, 6H), 1.85 – 1.74 (m, 1H), 1.66 – 1.54 (m, 4H), 1.41 (s, 9H), 1.33 – 1.04 (m, 3H).

^{13}C NMR (101 MHz, CD_3CN) δ 181.9, 155.3, 145.4, 134.3, 130.9, 128.5, 127.2, 112.5, 112.2, 86.2, 79.6, 48.4, 47.8, 46.6, 44.3, 36.9, 31.2, 31.7, 28.6, 26.4, 21.6, 17.9.

HRMS (ESI-TOF): $[M+Na]^+$ calculated for $C_{29}H_{38}NaN_4O_4S^+$ 561.2506; found 561.2533.

HPLC (Chiralpak ID, *i*-PrOH:hexanes = 10:90, flow rate = 0.4 mL/min, UV = 254 nm) t_R1 = 47.97 min (minor) and t_R2 = 51.49 min (major), er = 99:1.

Compound 4c

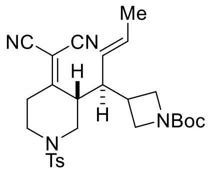

Prepared according to the General procedure C using **1a** and ***rac*-2c** and obtained in 76% yield (77.6 mg, 0.152 mmol) as a white solid.

R*f* = 0.20 in 30% EtOAc in hexanes.

^{1}H NMR (400 MHz, CD_3CN) δ 7.63 (d, *J* = 8.2 Hz, 2H), 7.42 (d, *J* = 8.2 Hz, 2H), 5.65 – 5.55 (m, 1H), 5.20 – 5.11 (m, 1H), 4.01 – 3.89 (m, 2H), 3.78 (ddd, *J* = 21.3, 17.9, 10.7 Hz, 3H), 3.66 – 3.58 (m, 1H), 2.88 – 2.65 (m, 5H), 2.42 (s, 3H), 2.38 – 2.29 (m, 2H), 1.66 (dd, *J* = 6.4, 1.4 Hz, 3H), 1.40 (s, 9H).

^{13}C NMR (101 MHz, CD_3CN) δ 180.8, 157.2, 145.5, 133.4, 131.8, 130.9, 126.6, 126.8, 112.5, 112.2, 86.5, 79.6, 53.5, 48.3, 47.1, 46.6, 46.3, 31.6, 31.2, 28.6, 21.5, 18.0.

HRMS (ESI-TOF): $[M+Na]^+$ calculated for $C_{27}H_{34}NaN_4O_4S^+$ 533.2193; found 533.2215.

HPLC: (Chiralpak ID, *i*-PrOH:hexanes = 20:80, flow rate = 1 mL/min, UV = 254 nm) t_R1 = 10.25 (minor) and t_R2 = 18.16 min (major), er = 93:7.

Compound 4d

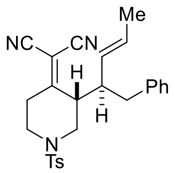

Prepared according to the General procedure C using **1a** and ***rac*-2d** on a 0.2 mmol scale and obtained in 72% yield (64.2 mg, 0.144 mmol) as a white solid.

R*f* = 0.38 in 20% EtOAc in hexanes.

^{1}H NMR (400 MHz, CDCl$_3$) δ 7.63 (d, *J* = 8.1 Hz, 2H), 7.32 (d, *J* = 8.1 Hz, 2H), 7.23 (d, *J* = 6.6 Hz, 2H), 7.15 (t, *J* = 8.6 Hz, 3H), 5.11 – 4.96 (m, 2H), 4.24 (d, *J* = 12.5 Hz, 1H), 4.10 – 4.00 (m, 1H), 3.18 (dd, *J* = 12.9, 2.5 Hz, 1H), 2.95 – 2.71 (m, 3H), 2.66 – 2.54 (m, 1H), 2.41 (s, 4H), 2.38 – 2.29 (m, 2H), 1.49 (d, *J* = 4.6 Hz, 3H).

^{13}C NMR (101 MHz, CDCl$_3$) δ 180.4, 144.5, 138.6, 132.9, 130.1, 129.8, 129.6, 128.5, 128.2, 127.5, 126.3, 111.2, 111.0, 85.7, 47.6, 47.0, 46.4, 45.9, 39.3, 31.1, 21.6, 17.7.

HRMS (ESI-TOF): $[M+Na]^+$ calculated for $C_{26}H_{27}NaN_3O_2S^+$ 468.1716; found 468.1732.

HPLC: (Chiralpak ID, *i*-PrOH:hexanes = 10:90, flow rate = 1 mL/min, UV = 254 nm) t_R1 = 8.58 min (major) and t_R2 = 11.48 min (major), er = 94:6.

Compound 4e

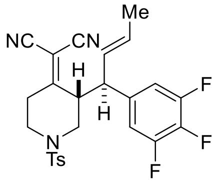

Prepared according to the General procedure C ([AAA] was carried at 4 °C) using **1a** and ***rac*-2e** on a 0.20 mmol scale and obtained in 77% yield (75.0 mg, 0.15 mmol) as a white solid.

R*f* = 0.41 in 20% EtOAc in hexanes.

^{1}H NMR (400 MHz, CDCl$_3$) δ 7.53 (d, *J* = 8.2 Hz, 2H), 7.30 (d, *J* = 8.2 Hz, 2H), 7.06 – 6.95 (m, 2H), 5.64 – 5.49 (m, 1H), 5.49 – 5.38 (m, 1H), 4.16 – 4.10 (m, 1H), 3.78 (t, *J* = 10.3 Hz, 1H), 3.55 (d, *J* = 12.3 Hz, 1H), 3.15 (d, *J* = 10.8 Hz, 1H), 2.99 (d, *J* = 15.0 Hz, 1H), 2.72 (ddd, *J* = 14.9, 12.3, 6.3 Hz, 1H), 2.41 (s, 3H), 2.36 – 2.27 (m, 1H), 2.20 (dd, *J* = 12.3, 3.1 Hz, 1H), 1.66 (dd, *J* = 6.4, 1.2 Hz, 3H).

^{13}C NMR (101 MHz, CDCl$_3$) δ 178.4, 151.5 (ddd, *J* = 251.5, 9.8, 3.8 Hz), 144.8, 139.3 (dt, *J* = 251.8, 15.3 Hz), 136.9 (q, *J* = 6.6 Hz), 132.1, 130.1, 130.0, 128.0, 127.6, 112.7 – 112.3 (m), 111.2, 110.6, 87.0, 49.6, 48.3, 47.7, 46.5, 30.8, 21.6, 17.9.

^{19}F NMR (377 MHz, CDCl$_3$) δ -132.7 (d, *J* = 20.6 Hz), -161.4 (t, *J* = 20.6 Hz).

HRMS (ESI-TOF): $[M+Na]^+$ calculated for $C_{28}H_{22}NaN_2O_6S^+$ 508.1277; found 508.1277.

HPLC: (Chiralpak AD-H, *i*-PrOH:hexanes = 2:98, flow rate = 0.8 mL/min, UV = 254 nm) t_R1 = 12.71 min (major) and t_R2 = 14.29 min (minor), er = 88.3:11.7.

Compound 4f

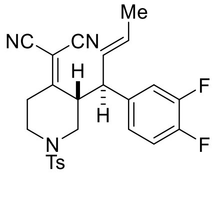

Prepared according to the General procedure C ([AAA] was carried at 4 °C) using **1a** and ***rac*-2f** on a 0.20 mmol scale and obtained in 76% yield (70.8 mg, 0.15 mmol) as a white solid.

R*f* = 0.37 in 20% EtOAc in hexanes.

^{1}H NMR (400 MHz, CDCl$_3$) δ 7.53 (d, *J* = 8.3 Hz, 2H), 7.30 (d, *J* = 8.0 Hz, 2H), 7.25 – 7.08 (m, 3H), 5.63 – 5.42 (m, 2H), 4.16 – 4.09 (m, 1H), 3.80 (t, *J* = 10.1 Hz, 1H), 3.60 – 3.54 (m, 1H), 3.16 (d, *J* = 10.7 Hz, 1H), 2.98 (d, *J* = 15.0 Hz, 1H), 2.79 – 2.68 (m, 1H), 2.41 (s, 3H), 2.30 (td, *J* = 12.0, 3.0 Hz, 1H), 2.18 (dd, *J* = 12.2, 3.2 Hz, 1H), 1.66 (dd, *J* = 6.2, 1.0 Hz, 3H).

^{13}C NMR (101 MHz, CDCl$_3$) δ 178.9, 150.7 (dd, *J* = 249.7, 12.6 Hz), 149.8 (dd, *J* = 248.6, 12.5), 144.7, 137.7 (t, *J* = 5.4, 3.8 Hz), 132.1, 130.1, 129.4, 128.6, 127.7, 124.7 (dd, *J* = 6.2, 3.6 Hz), 118.0 (d, *J* = 17.1), 117.0 (d, *J* = 17.4 Hz), 111.2, 110.7, 86.9, 49.6, 48.7, 47.7, 46.6, 30.9, 21.6, 17.9.

^{19}F NMR (377 MHz, CDCl$_3$) δ -136.0 (d, *J* = 21.2), -139.0 (d, *J* = 21.2 Hz).

HRMS (ESI-TOF): $[M+Na]^+$ calculated $C_{25}H_{23}F_2NaN_3O_2S^+$ 490.1371; found 490.1381.

HPLC: (Chiralpak ID, *i*-PrOH:hexanes = 10:90, flow rate = 1 mL/min, UV = 254 nm) t_R1 = 5.82 min (minor) and t_R2 = 6.98 min (major), er = 93.7:6.3.

Compound 4g

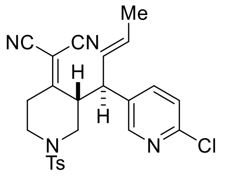

Prepared according to the General procedure C ([AAA] was carried at 4 °C) using **1a** and ***rac*-2g** on a 0.2 mmol scale and obtained in 69% yield (65.0 mg, 0.139 mmol) as a white solid.

R*f* = 0.26 in 30% EtOAc in hexanes.

^{1}H NMR (400 MHz, CDCl$_3$) δ 8.40 (d, *J* = 2.4 Hz, 1H), 7.66 (dd, *J* = 8.2, 2.5 Hz, 1H), 7.51 (d, *J* = 8.2 Hz, 2H), 7.35 (d, *J* = 8.2 Hz, 1H), 7.29 (d, *J* = 8.2 Hz, 2H), 5.67 – 5.54 (m, 1H), 5.54 – 5.43 (m, 1H), 4.18 – 4.07 (m, 1H), 3.88 (t, *J* = 10.3 Hz, 1H), 3.52 (d, *J* = 12.3 Hz, 1H), 3.20 (d, *J* = 10.8 Hz, 1H), 3.00 (d, *J* = 14.9 Hz, 1H), 2.81 – 2.68 (m, 1H), 2.40 (s, 3H), 2.38 – 2.27 (m, 1H), 2.20 (dd, *J* = 12.3, 3.1 Hz, 1H), 1.66 (d, *J* = 6.2 Hz, 3H).

^{13}C NMR (101 MHz, CDCl$_3$) δ 178.4, 150.7, 149.6, 144.7, 138.9, 135.4, 132.1, 130.3, 130.1, 127.9, 127.6, 124.8, 111.1, 110.6, 87.1, 48.2, 47.6, 47.2, 46.5, 30.8, 21.6, 17.9.

HRMS (ESI-TOF): $[M+Na]^+$ calculated for $C_{24}H_{23}ClNaN_4O_2S^+$ 489.1122; found 489.1132.

HPLC: (Chiralpak AD-H, *i*-PrOH:hexanes = 20:80, flow rate = 1 mL/min, UV = 254 nm) t_R1 = 4.10 min (minor) and t_R2 = 6.12 min (major), er = 92.1:7.9.

Compound 4h

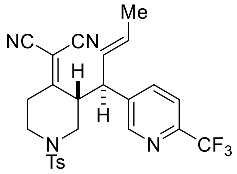

Prepared according to the General procedure C using **1a** and ***rac*-2h** on a 0.20 mmol scale and obtained in 82% yield (82.0 mg, 0.16 mmol) as a light-yellow solid.

R*f* = 0.19 in 30% EtOAc in hexanes.

^{1}H NMR (400 MHz, CDCl$_3$) δ 8.75 (d, *J* = 1.9, 1H), 7.90 (dd, *J* = 8.1, 1.9 Hz, 1H), 7.72 (d, *J* = 8.1 Hz, 1H), 7.51 (d, *J* = 8.3 Hz, 2H), 7.29 (d, *J* = 8.3 Hz, 2H), 5.65 (dq, *J* = 14.9, 6.2 Hz, 1H), 5.54 (ddd, *J* = 15.0, 9.8, 1.3 Hz, 1H), 4.19 – 4.12 (m, 1H), 3.99 (t, *J* = 10.3 Hz, 1H), 3.50 (d, *J* = 12.4 Hz, 1H), 3.28 (d, *J* = 10.8 Hz, 1H), 3.02 (d, *J* = 15.0 Hz, 1H), 2.76 (ddd, *J* = 14.9, 12.3, 6.3 Hz, 1H), 2.43 – 2.30 (m, 4H), 2.23 (dd, *J* = 12.4, 3.1 Hz, 1H), 1.68 (dd, *J* = 6.2, 1.2 Hz, 3H).

^{13}C NMR (101 MHz, CDCl$_3$) δ 178.1, 149.9, 147.5 (q, *J* = 34.9 Hz), 144.8, 139.8, 137.7, 132.1, 130.9, 130.2, 127.5 (d, *J* = 6.1 Hz), 122.9, 121.58 (q, *J* = 274.1 Hz) 120.9 (q, *J* = 2.6), 120.2, 111.1, 110.6, 87.3, 48.1, 47.8, 46.5, 30.8, 21.6, 17.9.

^{19}F NMR (377 MHz, CDCl$_3$) δ -67.8.

HRMS (ESI-TOF): [M+Na]$^+$ calculated for $C_{25}H_{23}F_3NaN_4O_2S^+$ 523.1386; found 523.1410.

HPLC: (Chiralpak AD-H, *i*-PrOH:hexanes = 10:90, flow rate = 1 mL/min, UV = 254 nm) t_R1 = 5.88 min (minor) and t_R2 = 9.13 min (major), er = 88.3:11.7.

Compound 4i

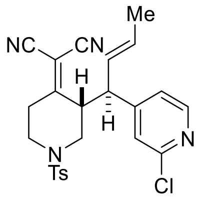

Prepared according to the General procedure C from **1a** and ***rac*-2i** on a 0.4 mmol scale and obtained in 73% yield (156.8 mg, 0.29 mmol, er = 85:15) as a white solid; from 1a and *rac*-2i-Ac on 0.20 mmol scale with addition of Et*i*Pr$_2$N (3 equiv., 69 mg, 0.6 mmol) and obtained in 66% yield (76.2 mg, 0.132 mmol, er = 95:5).

R*f* = 0.27 in 30% EtOAc in hexanes.

^{1}H NMR (400 MHz, CDCl$_3$) δ 8.40 (d, *J* = 5.1 Hz, 1H), 7.52 (d, *J* = 8.2 Hz, 2H), 7.34 – 7.27 (m, 3H), 7.24 (d, *J* = 5.1 Hz, 1H), 5.68 – 5.57 (m, 1H), 5.51 – 5.41 (m, 1H), 4.17 – 4.08 (m, 1H), 3.85 (t, *J* = 10.4 Hz, 1H), 3.51 (d, *J* = 12.4 Hz, 1H), 3.22 (d, *J* = 10.8 Hz, 1H), 3.00 (d, *J* = 14.9 Hz, 1H), 2.78 – 2.67 (m, 1H), 2.40 (s, 3H), 2.38 – 2.26 (m, 1H), 2.21 (dd, *J* = 12.4, 3.0 Hz, 1H), 1.67 (d, *J* = 6.3 Hz, 3H).

^{13}C NMR (101 MHz, CDCl$_3$) δ 178.0, 152.8, 152.5, 150.6, 144.8, 132.1, 131.0, 130.2, 127.6, 127.1, 123.8, 122.5, 111.1, 110.6, 87.3, 49.4, 47.7(1), 47.6(8), 46.5, 30.8, 21.6, 18.0.

HRMS (ESI-TOF): [M+Na]$^+$ Calculated for $C_{24}H_{23}ClNaN_4O_2S^+$ 489.1122; found 489.1130.

HPLC: (Chiralpak ID, *i*-PrOH:hexanes = 10:90, flow rate = 1 mL/min, UV = 254 nm) t_R1 = 13.71 min (minor) and t_R2 = 11.48 min (major), er = 85:15.

Compound 4j

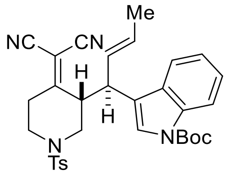

Prepared according to the General procedure C from **1a** and ***rac*-2k-Ac** on 0.20 mmol scale with addition of K_3PO_4 (3 equiv., 127.4 mg, 0.6 mmol) and obtained in 67% yield (76.2 mg, 0.134 mmol) as a light-yellow solid. *Note*: [3,3]-Cope rearrangement occurred at room temperature and was complete upon completion of the [AAA].

R_f = 0.33 in 20% EtOAc in hexanes.

^{1}H NMR (400 MHz, CDCl$_3$) δ 8.25 (d, *J* = 8.1 Hz, 1H), 7.77 (d, *J* = 7.7 Hz, 1H), 7.71 (s, 1H), 7.51 (d, *J* = 8.3 Hz, 2H), 7.36 (td, *J* = 9.2, 8.2, 0.9 Hz, 1H), 7.32 – 7.23 (m, 3H), 5.81 (dd, *J* = 14.8, 10.8 Hz, 1H), 5.68 – 5.55 (m, 1H), 4.17 – 4.03 (m, 2H), 3.73 (d, *J* = 12.0 Hz, 1H), 3.55 (d, *J* = 10.9 Hz, 1H), 3.01 (d, *J* = 14.9 Hz, 1H), 2.81 (ddd, *J* = 14.3, 12.1, 6.0 Hz, 1H), 2.39 (s, 3H), 2.30 (td, *J* = 11.9, 3.0 Hz, 1H), 2.16 (dd, *J* = 12.0, 3.2 Hz, 1H), 1.70 – 1.62 (m, 12H).

^{13}C NMR (101 MHz, CDCl$_3$) δ 179.9, 149.5, 144.3, 136.4, 132.2, 129.9, 129.2, 127.8, 127.7, 127.6, 125.5, 122.8, 119.9, 118.2, 115.7, 111.5, 110.9, 86.6, 83.8, 48.2, 46.7, 46.1, 43.0, 30.8, 28.2, 21.5, 17.8.

HRMS (ESI-TOF): $[M+NH_4]^+$ calculated for $C_{32}H_{38}N_5O_4S^+$ 588.2639; found 588.2636.

HPLC: (Chiralpak AD-H, *i*-PrOH:hexanes = 2.5:97.5, flow rate = 0.5 mL/min, UV = 254 nm) t_R1 = 8.58 min (major) and t_R2 = 11.48 min (major), er = 93.4:6.6.

Compound 4k

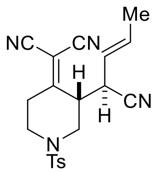

Prepared according to the General procedure C using **1a** and ***rac*-2l** on a 0.4 mmol scale with the following modifications: upon completion of AAA the alkylation product was purified using silica gel column chromatography (R_f = 0.41 in 40% EtOAc in hexanes), dissolved in toluene (0.1 M) and heated at 110 °C for 4 hours to provide the desired product in 71% yield (108 mg, 0.284 mmol) as a colorless oil.

Note: compound 4k was unstable on silica gel and alumina, and purification attempts by column chromatography led to epimerization/racemization.

R_f = 0.32 in 30% EtOAc in hexanes.

^{1}H NMR (400 MHz, CDCl$_3$) δ 7.66 (d, *J* = 8.3 Hz, 2H), 7.36 (d, *J* = 8.0 Hz, 2H), 5.92 (dq, *J* = 13.1, 6.6 Hz, 1H), 5.24 (ddd, *J* = 15.2, 8.4, 1.7 Hz, 1H), 4.29 – 4.21 (m, 1H), 4.13 (ddt, *J* = 9.2, 4.2, 2.1 Hz, 1H), 3.88 – 3.78 (m, 1H), 3.30 (d, *J* = 10.7 Hz, 1H), 2.99 (d, *J* = 15.2 Hz, 1H), 2.71 – 2.57 (m, 1H), 2.54 (dd, *J* = 12.9, 3.0 Hz, 1H), 2.46 – 2.36 (m, 4H), 1.76 (dd, *J* = 6.6, 1.4 Hz, 3H).

^{13}C NMR (101 MHz, CDCl$_3$) δ 174.4, 144.9, 134.9, 132.5, 130.3, 127.7, 120.0, 117.6, 110.4(3), 110.3(6), 88.8, 48.6, 46.4, 45.5, 35.7, 30.7, 21.7, 17.9.

HRMS (ESI-TOF): $[M+Na]^+$ calculated for $C_{20}H_{20}NaN_4O_2S^+$ 403.1199; found 403.1206.

HPLC: (Chiralpak AD-H, *i*-PrOH:hexanes = 10:90, flow rate = 1 mL/min, UV = 254 nm) t_R1 = 11.95 min (major) and t_R2 = 22.02 min (major), er = 70.5:29.5.

Compound 4l

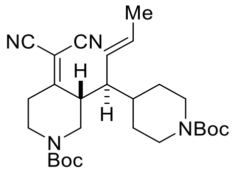

Prepared according to the General procedure C using **1b** and ***rac*-2b** on a 0.20 mmol scale and obtained in 79% yield (77.2 mg, 0.16 mmol) as a light-yellow oil.

R*f* = 0.17 in 20% EtOAc in hexanes.

^{1}H NMR (500 MHz, DMSO-*d₆*, 353 K) δ 5.40 (dq, *J* = 13.0, 6.3 Hz, 1H), 5.10 – 5.01 (m, 1H), 4.35 – 4.20 (m, 2H), 4.10 – 3.96 (m, 2H), 3.01 – 2.84 (m, 3H), 2.73 – 2.61 (m, 3H), 2.44 (ddd, *J* = 14.5, 11.9, 6.6 Hz, 1H), 2.16 – 2.08 (m, 1H), 1.97 – 1.87 (m, 1H), 1.68 – 1.59 (m, 4H), 1.45 (s, 9H), 1.39 (s, 9H), 1.28 – 1.14 (m, 2H), 1.07 (qd, *J* = 12.4, 4.1 Hz, 1H).

^{13}C NMR (126 MHz, DMSO-d_6, 353 K) δ 183.1, 153.5, 153.3, 128.5, 126.2, 111.3, 111.1, 83.4, 79.3, 78.1, 46.9, 44.03, 43.96, 42.7, 35.9, 30.8, 30.3, 27.7(2), 27.6(6), 25.2, 16.8.

HRMS (ESI-TOF): $[M+Na]^+$ calculated for $C_{27}H_{40}NaN_4O_4^+$ 507.2942; found 507.2934.

HPLC: (Chiralpak AD-H, *i*-PrOH:hexanes = 5:95, flow rate = 0.5 mL/min, UV = 254 nm) t_R1 = 6.82 min (major) and t_R2 = 8.42 min (minor), er = 99:1.

Compound 4m

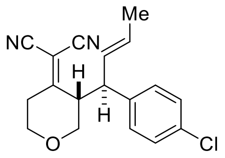

Prepared according to the General procedure C from **1c** and ***rac*-2a** on a 0.4 mmol scale and obtained in 68% yield (85.1 mg, 0.272 mmol) as a white solid.

R*f* = 0.40 in 20% EtOAc in hexanes.

^{1}H NMR (400 MHz, $CDCl_3$) 7.33 (d, *J* = 8.4 Hz, 2H), 7.20 (d, *J* = 8.4 Hz, 2H), 5.60 – 5.44 (m, 2H), 4.28 (dd, *J* = 11.2, 6.8 Hz, 1H), 3.78 – 3.64 (m, 2H), 3.46 (td, *J* = 11.6, 2.6 Hz, 1H), 3.27 (dd, *J* = 11.9, 2.5 Hz, 1H), 3.09 (d, *J* = 10.9 Hz, 1H), 2.88 (d, *J* = 14.6 Hz, 1H), 2.78 – 2.65 (m, 1H), 1.66 (d, *J* = 4.8 Hz, 3H).

^{13}C NMR (101 MHz, $CDCl_3$) δ 180.7, 139.6, 133.3, 129.7, 129.5, 129.4, 128.6, 111.6, 111.2, 85.5, 68.8, 68.3, 50.0, 49.8, 32.3, 17.9.

HRMS (ESI-TOF): $[M-H]^-$ calculated for $C_{18}H_{16}ClN_2O^-$ 311.0957; found 311.0970.

HPLC: (Chiralpak AD-H, *i*-PrOH:hexanes = 5:95, flow rate = 1 mL/min, UV = 254 nm) t_R1 = 3.07 min (minor) and t_R2 = 3.53 min (major), er = 91.9:8.1.

Compound 4n

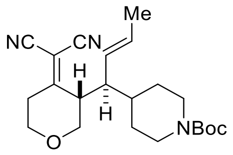

Prepared according to the General procedure C using **1c** and ***rac*-2b** on a 0.2 mmol scale and obtained in 90% yield (69.4 mg, 0.18 mmol) as a white solid.

R*f* = 0.33 in 30% EtOAc in hexanes.

^{1}H NMR (400 MHz, CDCl$_3$) δ 5.46 – 5.33 (m, 1H), 5.06 (dd, *J* = 14.2, 11.4 Hz, 1H), 4.18 (dd, *J* = 11.6, 5.4 Hz, 4H), 3.47 – 3.33 (m, 2H), 2.91 (d, *J* = 10.5 Hz, 1H), 2.80 – 2.43 (m, 5H), 1.77 – 1.62 (m, 4H), 1.56 (d, *J* = 13.5 Hz, 1H), 1.42 (s, 11H), 1.29 – 1.14 (m, 1H).

^{13}C NMR (101 MHz, CDCl$_3$) δ 181.8, 154.8, 130.1, 126.3, 111.4, 111.2, 84.6, 79.5, 68.6, 68.1, 47.6, 45.2, 44.1, 37.1, 32.5, 31.2, 28.5, 26.0, 17.9.

HRMS (ESI-TOF): [M+Na]$^+$ calculated for $C_{22}H_{31}NaN_3O_3^+$ 408.2258; found 408.2261.

HPLC: (Chiralpak IA, *i*-PrOH:hexanes = 2.5:97.5, flow rate = 0.25 mL/min, UV = 254 nm) t_R1 = 23.75 min (minor) and t_R2 = 26.70 min (major), er = 99:1.

Compound 4o

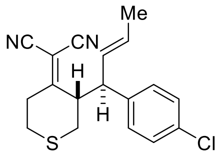

Prepared according to the General procedure C from **1d** and ***rac*-2a** on a 0.2 mmol scale and obtained in 67% yield (44 mg, 0.13 mmol) as a white solid.

R*f* = 0.54 in 20% EtOAc in hexanes.

^{1}H NMR (400 MHz, CDCl3) δ 7.33 (d, *J* = 8.5 Hz, 2H), 7.22 (d, *J* = 8.5 Hz, 2H), 5.60 – 5.46 (m, 2H), 4.20 – 4.09 (m, 1H), 3.44 (dt, *J* = 10.9, 3.1 Hz, 1H), 3.29 – 3.20 (m, 1H), 2.96 – 2.86 (m, 1H), 2.86 – 2.66 (m, 3H), 2.35 (dt, *J* = 14.3, 2.7 Hz, 1H), 1.65 (d, *J* = 4.9 Hz, 3H).

^{13}C NMR (101 MHz, CDCl$_3$) δ 182.7, 139.4, 133.3, 129.5, 129.4, 129.2, 128.8, 111.5, 111.2, 86.2, 49.5, 48.3, 33.2, 32.7, 30.8, 18.0.

HRMS (ESI-TOF): [M-H]$^-$ calculated for $C_{18}H_{16}ClN_2S^-$ 327.0728; found 327.0729.

HPLC: (Chiralpak AD-H, *i*-PrOH:hexanes = 1:99 flow rate = 0.3 mL/min, UV = 254 nm) t_R1 = 14.88 min (minor) and t_R2 = 16.59 min (major), er = 92:8.

Compound 4p

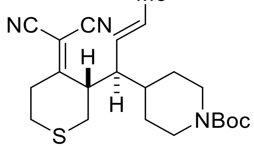

Prepared according to the General procedure C using **1d** and ***rac*-2b** on a 0.2 mmol scale and obtained in 86% yield (68.8 mg, 0.17 mmol) as a light-yellow solid.

R*f* = 0.42 in 20% EtOAc in hexanes.

¹H NMR (400 MHz, CDCl₃) δ 5.43 (dq, *J* = 15.0, 6.4 Hz, 1H), 5.12 – 4.99 (m, 1H), 4.31 – 4.02 (m, 3H), 3.27 (d, *J* = 10.5 Hz, 1H), 3.12 (dt, *J* = 13.9, 2.7 Hz, 1H), 2.95 – 2.51 (m, 8H), 1.69 – 1.50 (m, 5H), 1.45 – 1.30 (m, 10H), 1.29 – 1.15 (m, 1H).

^{13}C NMR (101 MHz, CD_3CN) δ 185.5, 155.3, 130.6, 127.6, 112.7, 112.5, 85.6, 79.6, 48.1, 46.8, 44.6, 36.3, 33.6, 32.7, 32.0, 30.8, 28.6, 26.2, 18.0.

HRMS (ESI-TOF): $[M+Na]^+$ calculated for $C_{22}H_{31}NaN_3O_2S^+$ 424.2029; found 424.2048.

HPLC: (Chiralpak IA, *i*-PrOH:hexanes = 2.5:97.5, flow rate = 0.25 mL/min, UV = 254 nm) t_R1 = 21.96 min (minor) and t_R2 = 23.46 min (major), er = 98.8:1.2.

Compound 4q

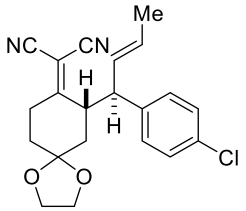

Prepared according to the General procedure C from **1e** and ***rac*-2a** on a 0.2 mmol scale and obtained in 57% yield (42.0 mg, 0.114 mmol) as a white solid.

R_f = 0.28 in 20% EtOAc in hexanes.

^{1}H NMR (400 MHz, $CDCl_3$) δ 7.25 (d, *J* = 8.5 Hz, 2H), 7.11 (d, *J* = 8.5 Hz, 2H), 5.48 – 5.30 (m, 2H), 4.06 – 3.64 (m, 5H), 3.34 – 3.25 (m, 1H), 2.96 – 2.98 (m, 1H), 2.65 (td, *J* = 14.4, 5.2 Hz, 1H), 2.05 – 1.93 (m, 1H), 1.67 (td, *J* = 14.0, 4.4 Hz, 1H), 1.62 – 1.47 (m, 5H).

^{13}C NMR (101 MHz, $CDCl_3$) δ 184.0, 140.6, 132.8, 131.1, 129.6, 129.2, 127.9, 112.0, 111.6, 106.7, 85.6, 65.2, 64.4, 51.3, 47.9, 36.1, 35.4, 29.1, 17.9.

HRMS (ESI-TOF): $[M-H]^-$ calculated for $C_{21}H_{20}ClN_2O_2^-$ 367.1219; found 367.1235.

HPLC: (Chiralpak IA, *i*-PrOH:hexanes = 5:95, flow rate = 0.5 mL/min, UV = 254 nm) t_R1 = 6.63 min (major) and t_R2 = 7.34 min (minor), er = 93.6:6.4.

Compound 4r

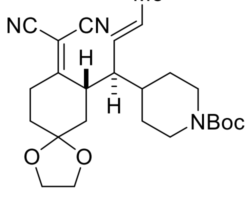

Prepared according to the General procedure C using **1e** and ***rac*-2b** and on a 0.2 mmol scale and obtained in 55% yield (49.0 mg, 0.11 mmol) as a white solid.

R_f = 0.17 in 30% EtOAc in hexanes.

^{1}H NMR (400 MHz, CD_3CN) δ 5.37 (dq, *J* = 15.0, 6.4 Hz, 1H), 5.15 – 5.03 (m, 1H), 4.14 – 3.82 (m, 6H), 3.09 (dd, *J* = 11.1, 5.7 Hz, 1H), 2.84 – 2.52 (m, 5H), 1.99 (ddd, *J* = 10.3, 5.4, 2.5 Hz, 1H), 1.91 – 1.80 (m, 1H), 1.82 – 1.67 (m, 2H), 1.63 (dd, *J* = 6.4, 1.6 Hz, 3H), 1.55 (d, *J* = 14.1 Hz, 1H), 1.41 (s, 10H), 1.29 – 1.02 (m, 3H).

^{13}C NMR (101 MHz, CD_3CN) δ 186.9, 155.4, 130.0, 128.8, 113.1, 112.9, 107.5, 85.0, 79.6, 65.9, 65.0, 49.9, 44.4, 37.1, 36.1, 35.3, 32.1, 30.0, 28.6, 26.2, 17.9.

HRMS (ESI-TOF): $[M+Na]^+$ calculated for $C_{25}H_{35}NaN_3O_4^+$ 464.2520; found 464.2535.

HPLC: (Chiralpak AD-H, *i*-PrOH:hexanes = 5:95, flow rate = 1 mL/min, UV = 254 nm) t_R1 = 4.73 min (minor) and t_R2 = 11.48 min (major), er = 98.5:1.5.

Compound 4u

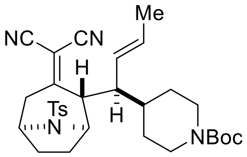

Prepared according to the General procedure C from **1g** and ***rac*-2b** on a 0.40 mmol scale and obtained in 37% yield (0.084 g, 0.15 mmol) as a white solid.

R_f = 0.38 in 30% EtOAc in hexanes.

^{1}H NMR (400 MHz, CD_3CN) δ 7.78 (d, *J* = 8.2 Hz, 2H), 7.40 (d, *J* = 8.2 Hz, 2H), 5.36 (dq, *J* = 15.2, 6.3 Hz, 1H), 5.17 – 5.04 (m, 1H), 4.49 (br, 1H), 4.37 (d, *J* = 7.4 Hz, 1H), 4.06 (m, 2H), 2.89 (d, *J* = 10.0 Hz, 1H), 2.77 (dd, *J* = 15.2, 1.6 Hz, 1H), 2.71 – 2.46 (m, 3H), 2.44 – 2.33 (m, 4H), 1.80 (br, 1H), 1.68 – 1.50 (m, 5H), 1.47 – 1.32 (m, 12H), 1.17 – 1.01 (m, 3H).

^{13}C NMR (101 MHz, CD_3CN) δ 181.8, 155.4, 145.6, 137.5, 131.0, 130.6, 128.4, 127.5, 112.8, 112.3, 89.6, 79.6, 59.2, 58.6, 52.8, 50.3, 44.6 (br), 41.4, 37.0, 32.0, 29.3, 28.9, 28.6, 26.6, 21.6, 17.8.

HRMS (ESI$^-$): $[M-H]^-$ calculated for $C_{31}H_{39}N_4O_4S^-$ 563.2698; found 563.2709.

HPLC (Chiralpak AD-H, *i*-PrOH:hexanes = 6:94, flow rate = 1 mL/min, UV = 254 nm) t_R1 = 8.49 min (major) and t_R2 = 9.94 min (minor), er = 95:5.

Compound 4v

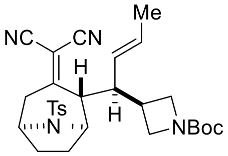

Prepared according to the General procedure C from **1g** and ***rac*-2c** on a 0.20 mmol scale and obtained in 39% yield (0.042 g, 0.08 mmol) as a white solid.

R_f = 0.29 in 30% EtOAc in hexanes.

^{1}H NMR (400 MHz, $CDCl_3$) δ 7.72 (d, *J* = 8.3 Hz, 2H), 7.32 (d, *J* = 8.3 Hz, 2H), 5.51 (dq, *J* = 15.2, 6.3 Hz, 1H), 5.31 (dd, *J* = 15.2, 8.1 Hz, 1H), 4.46 (br, 1H), 4.24 (d, *J* = 7.4 Hz, 1H), 3.90 (m, 2H), 3.73 (dd, *J* = 8.9, 5.9 Hz, 1H), 3.65 (dd, *J* = 8.4,

5.6 Hz, 1H), 3.09 – 2.96 (m, 1H), 2.81 (dd, J = 15.1, 2.7 Hz, 1H), 2.72 – 2.56 (m, 3H), 2.44 (s, 3H), 1.71 (dd, J = 6.3, 1.6 Hz, 3H), 1.60 (m, 1H), 1.50 – 1.39 (m, 10H), 1.38 – 1.23 (m, 2H).

^{13}C NMR (101 MHz, CDCl$_3$) δ 179.3, 156.3, 144.8, 136.2, 131.9, 130.2, 127.4, 125.0, 111.3, 110.9, 89.4, 79.6, 58.5, 57.4, 53.5, 51.9, 50.1, 46.7, 40.8, 29.4, 28.8, 28.5, 28.2, 21.7, 18.1.

HRMS (ESI$^-$): [M - H]$^-$ calculated for $C_{29}H_{35}N_4O_4S^-$ 535.2384; found 535.2395.

HPLC (Chiralpak AD-H, *i*-PrOH:hexanes = 10:90, flow rate = 1 mL/min, UV = 254 nm) t_R1 = 7.79 min (minor) and t_R2 = 17.76 min (major), er = 95:5.

Compound 4w

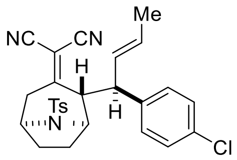

Prepared according to the General procedure C from **1g** and ***rac*-2a** on a 1.0 mmol scale and obtained in 42% yield (0.209 g, 0.42 mmol) as a white solid.

R*f* = 0.32 in 20% EtOAc in hexanes.

^{1}H NMR (400 MHz, CDCl$_3$) δ 7.66 (d, J = 8.2 Hz, 2H), 7.35 (s, 4H), 7.28 (d, J = 8.2 Hz, 2H), 5.56 – 5.43 (m, 2H), 4.52 (br, 1H), 3.99 – 3.90 (m, 1H), 3.89 – 3.78 (m, 1H), 3.11 (d, J = 9.6 Hz, 1H), 2.90 (dd, J = 15.2, 1.6 Hz, 1H), 2.74 (dd, J = 15.2, 3.8 Hz, 1H), 2.43 (s, 3H), 1.63 (d, J = 4.8 Hz, 3H), 1.47 – 1.15 (m, 4H).

^{13}C NMR (101 MHz, CDCl$_3$) δ 179.5, 144.7, 139.9, 136.1, 133.2, 130.2, 129.9, 129.6, 129.3, 128.5, 127.5, 111.7, 110.9, 90.0, 58.5, 57.5(0), 57.4(8), 51.3, 41.1, 28.9, 27.8, 21.7, 17.8.

HRMS (ESI$^-$): [M - H]$^-$ calculated for $C_{27}H_{25}ClN_3O_2S^-$ 490.1361; found 490.1368.

HPLC (Chiralpak AD-H, *i*-PrOH:hexanes = 4:96, flow rate = 1 mL/min, UV = 254 nm) t_R1 = 7.25 min (minor) and t_R2 = 10.51 min (major), er = 94:6.

Compound 4x

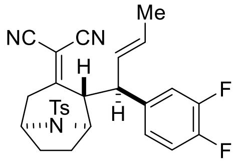

Prepared according to the General procedure C from **1g** and ***rac*-2f** on a 0.40 mmol scale and obtained in 30% yield (0.060 g, 0.12 mmol) as a white solid.

R*f* = 0.50 in 30% EtOAc in hexanes.

^{1}H NMR (400 MHz, CDCl$_3$) δ 7.66 (d, J = 8.2 Hz, 2H), 7.29 (d, J = 8.2 Hz, 2H), 7.20 – 7.10 (m, 3H), 5.60 – 5.37 (m, 2H), 4.54 (br, 1H), 3.94 (br, 1H), 3.80 (dd, appears as t, J = 9.5 Hz, 1H), 3.08 (d, J = 11.5 Hz, 1H), 2.91 (dd, J = 15.7, 1.9 Hz, 1H), 2.73 (dd, J = 15.3, 3.9 Hz, 1H), 2.43 (s, 3H), 1.64 (d, J = 4.9 Hz, 3H), 1.45 – 1.20 (m, 4H).

^{13}C NMR (101 MHz, $CDCl_3$) δ 179.2, 150.5 (dd, *J* = 248.7, 12.6 Hz), 149.6 (dd, *J* = 248.7, 12.6 Hz), 144.8, 138.3 (dd, appears as t, *J* = 4.4 Hz), 136.0, 130.2, 129.4, 128.8, 127.5, 124.5 (dd, *J* = 6.2, 3.4 Hz), 117.9 (d, *J* = 17.3 Hz), 117.4 (d, *J* = 17.3 Hz), 111.7, 110.9, 90.2, 58.5, 57.5, 57.3, 51.1, 41.1, 28.9, 27.9, 21.7, 17.8.

^{19}F NMR (377 MHz, $CDCl_3$) δ -136.3 (d, *J* = 21.3 Hz), -139.6 (d, *J* = 21.3 Hz).

HRMS (DART$^+$): $[M + H]^+$ calculated for $C_{27}H_{26}F_2N_3O_2S^+$ 494.1708; found 494.1693. Calculated for $C_{27}H_{29}F_2N_4O_2S$ $[M + NH_4]^+$ 511.1974; found 511.1960.

HPLC (Chiralpak ID, *i*-PrOH:hexanes = 5:95, flow rate = 0.2 mL/min, UV = 280 nm) t_R1 = 43.70 min (minor) and t_R2 = 59.03 min (major), er = 94:6.

Compound 4y

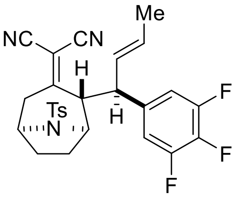

Prepared according to the General procedure C from **1g** and ***rac*-2e** on a 0.40 mmol scale and obtained in 37% yield (0.063 g, 0.12 mmol) as a white solid.

R*f* = 0.27 in 20% EtOAc in hexanes

^{1}H NMR (600 MHz, $CDCl_3$) δ 7.67 (d, *J* = 8.2 Hz, 2H), 7.30 (d, *J* = 8.2 Hz, 2H), 7.06 – 6.96 (m, 2H), 5.50 (dq, *J* = 15.2, 6.4 Hz, 1H), 5.40 (ddd, *J* = 15.2, 10.1, 1.7 Hz, 1H), 4.56 – 4.51 (m, 1H), 3.94 – 3.90 (m, 1H), 3.77 (dd, appears as t, *J* = 10.1 Hz, 1H), 3.05 (d, *J* = 10.1 Hz, 1H), 2.94 – 2.87 (m, 1H), 2.72 (dd, *J* = 15.3, 3.9 Hz, 1H), 2.44 (s, 3H), 1.64 (d, *J* = 6.4 Hz, 3H), 1.45 – 1.32 (m, 3H), 1.29 – 1.24 (m, 1H).

^{13}C NMR (151 MHz, $CDCl_3$) δ 178.6, 151.5 (dd, *J* = 251.0, 3.8 Hz), 151.4 (dd, *J* = 251.0, 3.8 Hz), 144.9, 138.9 (dt, *J* = 251.9, 15.1 Hz), 137.6 (q, *J* = 6.6 Hz), 135.9, 130.3, 129.4, 128.7, 127.5, 112.8 – 112.4 (m), 111.6, 110.8, 90.4, 58.5, 57.5, 56.9, 51.1, 41.1, 28.9, 27.9, 21.7, 17.8.

^{19}F NMR (377 MHz, $CDCl_3$) δ -132.96 (d, *J* = 20.6 Hz), -161.93 (t, *J* = 20.7 Hz).

HRMS (ESI$^-$): $[M - H]^-$ calculated for $C_{27}H_{23}F_3N_3O_2S^-$ 510.1475; found 510.1469.

HPLC (Chiralpak ID, *i*-PrOH:hexanes = 10:90, flow rate = 1 mL/min, UV = 254 nm) t_R1 = 4.82 min (minor) and t_R2 = 6.07 min (major), er = 86:14.

Compound 4z

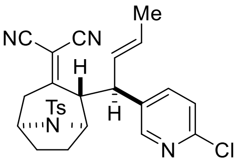

Prepared according to the General procedure C from **1g** and ***rac*-2g** on a 0.40 mmol scale and obtained in 38% yield (0.075 g, 0.15 mmol) as a white solid.

R*f* = 0.39 in 30% EtOAc in hexanes

^{1}**H NMR (400 MHz, CDCl$_3$)** δ 8.46 (d, *J* = 2.6 Hz, 1H), 7.72 (dd, *J* = 8.2, 2.6 Hz, 1H), 7.64 (d, *J* = 8.2 Hz, 2H), 7.35 (d, *J* = 8.2 Hz, 1H), 7.28 (d, *J* = 8.2 Hz, 2H), 5.61 – 5.40 (m, 2H), 4.53 (br, 1H), 4.01 – 3.79 (m, 2H), 3.10 (d, *J* = 10.4 Hz, 1H), 2.92 (dd, *J* = 15.3, 1.6 Hz, 1H), 2.75 (dd, *J* = 15.3, 3.8 Hz, 1H), 2.42 (s, 3H), 1.64 (d, *J* = 4.9 Hz, 3H), 1.44 – 1.28 (m, 3H), 1.27 – 1.18 (m, 1H).

^{13}**C NMR (101 MHz, CDCl$_3$)** δ 178.6, 150.6, 149.9, 144.9, 139.0, 135.9, 135.9, 130.2, 129.7, 128.7, 127.4, 124.7, 111.6, 110.8, 90.4, 58.4, 57.5, 56.9, 48.7, 41.1, 28.8, 27.9, 21.7, 17.8.

HRMS (ESI$^+$): [M + H]$^+$ calculated for $C_{26}H_{26}ClN_4O_2S^+$ 493.1460; found 493.1464. [M + Na]$^+$ Calculated for $C_{27}H_{25}ClN_4O_2SNa^+$ 515.1279; found 515.1263.

HPLC (Chiralpak ID, *i*-PrOH: hexanes = 20:80, flow rate = 1 mL/min, UV = 254 nm) t_R1 = 9.70 min (minor) and t_R2 = 15.23 min (major), er = 92:8.

Compound 4aa

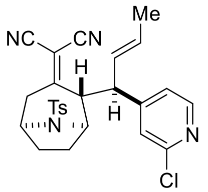

Prepared according to the General procedure C from **1g** and ***rac*-2i** on a 0.20 mmol scale and obtained in 33% yield (0.032 g, 0.07 mmol) as a white solid.

R*f* = 0.32 in 30% EtOAc in hexanes

^{1}**H NMR (400 MHz, CDCl$_3$)** δ 8.39 (d, *J* = 5.1 Hz, 1H), 7.65 (d, *J* = 8.3 Hz, 2H), 7.38 – 7.26 (m, 4H), 5.55 (dq, *J* = 15.0, 6.3 Hz, 1H), 5.43 (ddd, *J* = 15.0, 9.9, 1.5 Hz, 1H), 4.54 (br, 1H), 3.96 – 3.78 (m, 2H), 3.12 (d, *J* = 10.4 Hz, 1H), 2.92 (dd, *J* = 15.3, 1.6 Hz, 1H), 2.73 (dd, *J* = 15.3, 3.9 Hz, 1H), 2.43 (s, 3H), 1.65 (dd, *J* = 6.3, 1.5 Hz, 3H), 1.44 – 1.20 (m, 4H).

^{13}**C NMR (101 MHz, CDCl$_3$)** δ 178.2, 153.4, 152.3, 150.5, 144.9, 135.9, 130.4, 130.3, 127.9, 127.4, 124.1, 122.5, 111.6, 110.7, 90.6, 58.5, 57.5, 56.2, 51.0, 41.1, 28.8, 27.9, 21.8, 17.9.

HRMS (ESI$^+$): [M + H]$^+$ calculated for $C_{26}H_{26}ClN_4O_2S^+$ 493.1460; found 493.1467. [M + Na]$^+$ calculated for $C_{27}H_{25}ClN_4O_2SNa^+$ 515.1279; found 515.1273.

HPLC (Chiralpak ID, *i*-PrOH:hexanes = 20:80, flow rate = 1 mL/min, UV = 254 nm) t_R1 = 6.89 min (minor) and t_R2 = 9.01 min (major), er = 82:18.

Compound 4bb

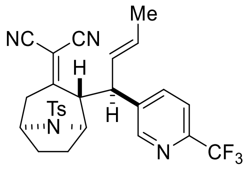

Prepared according to the General procedure C from **1g** and ***rac*-2h** on a 0.40 mmol scale and obtained in 40% yield (0.085 g, 0.16 mmol) as a white solid.

R*f* = 0.37 in 30% EtOAc in hexanes.

^{1}H NMR (400 MHz, CDCl$_3$) δ 8.81 (d, *J* = 2.2 Hz, 1H), 7.96 (dd, *J* = 8.1, 2.2 Hz, 1H), 7.72 (d, *J* = 8.1 Hz, 1H), 7.63 (d, *J* = 8.3 Hz, 2H), 7.32 – 7.24 (m, overlapped with solvent residual peak at 7.26, 2H), 5.66 – 5.44 (m, 2H), 4.54 (br, 1H), 4.02 (dd, appears as t, *J* = 9.9 Hz, 1H), 3.87 (d, *J* = 4.9 Hz, 1H), 3.18 (d, *J* = 10.3 Hz, 1H), 2.94 (dd, *J* = 15.3, 2.1 Hz, 1H), 2.77 (dd, *J* = 15.3, 3.9 Hz, 1H), 2.42 (s, 3H), 1.65 (d, *J* = 6.0 Hz, 3H), 1.43 – 1.18 (m, 4H).

^{13}C NMR (101 MHz, CDCl$_3$) δ 178.3, 150.1, 147.3 (q, *J* = 34.8 Hz), 144.9, 140.3, 137.8, 135.8, 130.3, 128.3, 127.4, 121.7 (q, *J* = 273.6 Hz), 120.8 (q, *J* = 2.5 Hz), 111.6, 110.7, 90.6, 58.4, 57.5, 56.6, 49.3, 41.1, 28.8, 27.8, 21.7, 17.8.

^{19}F NMR (377 MHz, CDCl$_3$) δ -67.7.

HRMS (ESI$^-$): [M - H]$^-$ calculated for $C_{27}H_{24}F_3N_4O_2S^-$ 525.1578; found 525.1595.

HPLC (Chiralpak ID, *i*-PrOH:hexanes = 20:80, flow rate = 1 mL/min, UV = 254 nm) t_R1 = 5.18 min (minor) and t_R2 = 7.16 min (major), er = 79:21.

Compound 4cc

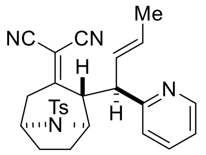

Prepared according to the General procedure C from **1g** and ***rac*-2j** on a 0.40 mmol scale and obtained in 34% yield (0.065 g, 0.14 mmol) as a white solid.

R*f* = 0.34 in 30% EtOAc in hexanes.

^{1}H NMR (400 MHz, CDCl$_3$) δ 8.60 (d, *J* = 4.9 Hz, 1H), 7.63 (ddd, appears as dt, *J* = 7.7, 1.5 Hz, 2H), 7.58 (d, *J* = 8.2 Hz, 2H), 7.25 – 7.21 (m, 3H), 7.17 (ddd, *J* = 7.6, 4.9, 1.5 Hz, 1H), 5.60 – 5.41 (m, 2H), 4.50 (br, 1H), 3.99 – 3.85 (m, 3H), 2.90 (dd, *J* = 15.1, 2.8 Hz, 1H), 2.79 (dd, *J* = 15.1, 3.8 Hz, 1H), 2.41 (s, 3H), 1.63 (d, *J* = 4.6 Hz, 3H), 1.51 – 1.29 (m, 4H).

^{13}C NMR (101 MHz, CDCl$_3$) δ 179.7, 159.4, 149.7, 144.4, 136.7, 136.3, 130.5, 130.1, 128.2, 127.4, 125.3, 122.1, 111.4, 89.9, 58.9, 57.6, 54.1, 53.2, 40.9, 29.0, 27.9, 21.7, 17.9.

HRMS (ESI$^+$): [M + H]$^+$ calculated for $C_{26}H_{27}N_4O_2S^+$ 459.1849; found 459.1865. [M + Na]$^+$ calculated for $C_{26}H_{26}N_4O_2SNa^+$ 481.1669; found 481.1669.

HPLC (Chiralpak ID, *i*-PrOH:hexanes = 20:80, flow rate = 1 mL/min, UV = 254 nm) t_R1 = 8.10 min (minor) and t_R2 = 10.20 min (major), er = 70:30.

Compound 4dd

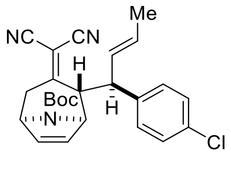

Prepared according to the General procedure C from **1h** and ***rac*-2a** on a 1.0 mmol scale and obtained in 43% yield (0.188 g, 0.43 mmol, er = 92:8) as a white solid; on 7.4 mmol using 1 mol% Pd_2dba_3 and 2 mol% (*S,S*)-DACH-phenyl Trost ligand and obtained in 41% yield (1.33 g, 3.1 mmol, er = 86:14).

R*f* = 0.45 in 20% EtOAc in hexanes.

^{1}H NMR (400 MHz, DMSO-d_6, 354 K) δ 7.43 (d, *J* = 8.5 Hz, 2H), 7.35 (d, *J* = 8.5 Hz, 2H), 6.30 – 6.22 (m, 2H), 5.54 – 5.42 (m, 2H), 4.96 – 4.90 (m, 1H), 4.41 – 4.37 (m, 1H), 3.77 – 3.68 (m, 1H), 3.34 (dd, *J* = 10.5, 2.1 Hz, 1H), 2.91 (d, *J* = 3.0 Hz, 2H), 1.59 (d, *J* = 4.8 Hz, 3H), 1.32 (s, 9H).

^{13}C NMR (101 MHz, DMSO-d_6, 354 K) δ 181.1, 139.7, 134.8, 133.7, 131.7, 131.2, 129.4, 128.3, 126.6, 111.5, 110.9, 89.2, 79.6, 59.1, 57.7, 50.7, 50.2, 35.2, 27.4, 16.7.

HRMS (ESI$^-$): [M - H]$^-$ calculated for $C_{25}H_{25}ClN_3O_2^-$ 434.1641; found 434.1646.

HPLC (Chiralpak IA, *i*-PrOH:hexanes = 2:98, flow rate = 0.4 mL/min, UV = 254 nm) t_R1 = 9.44 min (major) and t_R2 = 11.18 min (minor), er = 92:8.

Compound 4ee

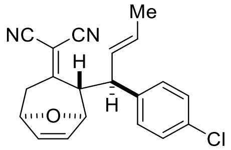

Prepared according to the General procedure C ([AAA] was conducted at room temperature, 3 hours) from **1h** and ***rac*-2a** on a 0.4 mmol scale and obtained in 50% yield (0.068 g, 0.2 mmol, 9:1 to 20:1 dr) as a white solid.

R_f = 0.41 in 20% EtOAc in hexanes.

^{1}H NMR (600 MHz, $CDCl_3$) δ 7.35 (d, *J* = 8.4 Hz, 2H), 7.25 (d, *J* = 8.4 Hz, 2H), 6.21 (dd, *J* = 6.1, 1.8 Hz, 1H), 6.12 (dd, *J* = 6.1, 1.9 Hz, 1H), 5.71 – 5.62 (m, 1H), 5.51 – 5.42 (m, 1H), 5.04 – 4.96 (m, 1H), 4.34 (s, 1H), 3.82 (dd, appears as t, *J* = 10.6 Hz, 1H), 3.04 (d, *J* = 10.6 Hz, 1H), 2.89 – 2.77 (m, 2H), 1.67 (dd, *J* = 6.4, 1.6 Hz, 3H).

^{13}C NMR (101 MHz, $CDCl_3$) δ 179.6, 139.6, 133.6, 133.3, 132.9, 131.0, 129.5, 129.4, 128.4, 111.8, 111.2, 90.3, 78.9, 78.5, 52.5, 51.2, 35.8, 17.8.

HRMS (ESI$^-$): [M - H]$^-$ calculated for $C_{20}H_{16}ClN_2O^-$ 335.0957; found 335.0966.

HPLC (Chiralpak AD-H, *i*-PrOH:hexanes = 1:99, flow rate = 1 mL/min, UV = 254 nm) t_R1 = 6.51 min (minor) and t_R2 = 9.23 (major), er = 89:11.

Reductive Cope rearrangement

General procedure D.

To an oven-dried 1-dram vial equipped with a stir bar $Pd_2(dba)_3$ (2.5 mol%), (*S,S*)-DACH phenyl Trost ligand (5 mol%) and alkylidenemalononitrile **1f** (1 equiv., 60.3 mg, 0.40 mmol) were added, and the contents were placed under N_2 atmosphere. Anhydrous DCM (0.5 M, 8 mL) was added via syringe, and the solution was stirred for 15 minutes (solution turned from purple to yellow-brown). An allylic electrophile ***rac*-2** (2.5 equiv.) was added via syringe, and the reaction was stirred at room temperature overnight (16 hours). Solvent was evaporated, and the crude material and Hantzsch ester (3 equiv., 304 mg, 1.2 mmol) were dissolved in toluene (0.1 M, 4 mL) and heated at the indicated temperature for the indicated time. The reaction mixture was concentrated under reduced pressure, redissolved in ethanol (0.05 M, 8 mL), and KOH (8.9 equiv., 606 mg, 10.8 mmol) was added. This mixture was heated at 75 °C for 1 hour to hydrolyze the pyridine byproduct, diluted with EtOAc (20 mL) and washed with saturated $NaHCO_3$ (2 × 15 mL) followed by brine, dried with anhydrous Na_2SO_4, filtered, and concentrated under reduced pressure. The desired compound was isolated using silica gel column chromatography with EtOAc in hexanes as an eluent.

Compound 5a

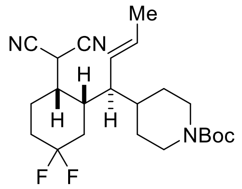

Prepared according to the General procedure D (reduction with Hantzsch ester occurred at 110 °C after 8 hours) and obtained in 60% yield (101.0 mg, 0.24 mmol) as a white solid.

R*f* = 0.32 in 20% EtOAc in hexanes.

^{1}H NMR (400 MHz, $CDCl_3$) δ 5.65 – 5.50 (m, 1H), 5.18 (dd, *J* = 14.8, 10.5 Hz, 1H), 4.25 – 4.02 (m, 3H), 2.73 – 2.54 (m, 2H), 2.38 – 1.84 (m, 9H), 1.70 (dd, *J* = 24.6, 6.4 Hz, 4H), 1.44 (s, 12H), 1.18 – 1.03 (m, 1H).

^{13}C NMR (101 MHz, $CDCl_3$) δ 154.7, 130.9, 129.8, 128.7, 128.0, 122.6 (t, *J* = 244.3, 238.4 Hz) 114.0, 112.5, 79.5, 48.5, 43.9, 36.5, 36.3, 36.1 (d, *J* = 8.4 Hz), 31.9 (ddd, *J* = 332.5, 25.1, 24.1 Hz), 31.0, 28.4, 25.1, 24.6 (d, *J* = 8.2 Hz), 21.3, 18.1.

^{19}F NMR (377 MHz, $CDCl_3$) δ = -89.8 (d, *J* = 240.9 Hz), -98.6 (d, *J* = 17.1 Hz).

HRMS (ESI-TOF): $[M-H]^-$ calculated for $C_{23}H_{32}F_2N_3O_2^-$ 420.2468; found 420.2477.

HPLC: (based on alkylidenemalonitrile **4s**, which was prepared using 0) (Chiralpak AD-H, *i*-PrOH:hexanes = 2.5:97.5, flow rate = 1 mL/min, UV = 254 nm) t_R1 = 5.71 min (major) and t_R2 = 6.77 min (minor), er = 98.5:1.5.

Compound 5b

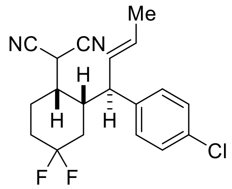

Prepared according to the General procedure D (reduction with Hantzsch ester occurred at 80 °C after 4 hours) and obtained in 49% yield (68.6 mg, 0.20 mmol) as a white solid.

R*f* = 0.34 in 10% EtOAc in hexanes.

^{1}H NMR (400 MHz, $CDCl_3$) δ 7.32 (d, *J* = 8.2 Hz, 2H), 7.09 (d, *J* = 8.2 Hz, 2H), 5.77 – 5.55 (m, 2H), 4.24 (d, *J* = 6.6 Hz, 1H), 3.24 (t, *J* = 10.3 Hz, 1H), 2.38 – 2.20 (m, 2H), 2.26 (d, *J* = 6.1 Hz, 2H), 2.18 – 1.83 (m, 3H), 1.74 – 1.59 (m, 4H).

^{13}C NMR (101 MHz, $CDCl_3$) δ 140.5, 132.9, 132.0, 129.5, 129.0, 128.7, 122.1 (t, *J* = 241.7, 240.9 Hz), 113.7, 112.5 (d, *J* = 160.5 Hz), 50.6, 40.1 (t, *J* = 5.8, 4.4 Hz), 37.3, 33.8 (t, *J* = 24.8 Hz), 30.4 (t, *J* = 25.5 Hz), 24.3 (t, *J* = 6.4, 5.5 Hz), 22.6, 18.1.

^{19}F NMR (377 MHz, $CDCl_3$) δ -90.2 (d, *J* = 242.0 Hz).

HRMS (ESI-TOF): $[M-H]^-$ calculated for $C_{19}H_{18}ClF_2N_2^-$ 347.1132; found 347.1142.

HPLC: (based on alkylidenemalonitrile **4t**, which was prepared using 0) (Chiralpak AD-H, *i*-PrOH:hexanes = 1:99, flow rate = 0.5 mL/min, UV = 254 nm) t_R1 = 8.52 min (minor) and t_R2 = 10.01 min (major), er = 95.5:4.5.

Reactivity of *meso*-4-*tert*-butylcyclohexyllidenemalononitrile

Compound 6ff

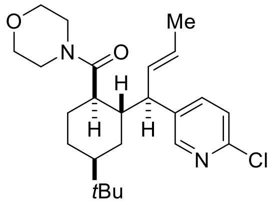

To an oven dried Schlenk flask equipped with $Pd_2(dba)_3$ (0.005 g, 2.5 mol%), (*S,S*)-DACH phenyl Trost ligand (0.007 g, 5 mol%), alkylidenemalononitrile **1g** (1 equiv., 0.040 g, 0.2 mmol), and a stir bar under N_2 atmosphere, anhydrous DCM (2 mL, 0.1 M) was added via syringe, and the contents were left at stirring. After 15 minutes electrophile ***rac*-2g** (2.5 equiv., 0.121 g, 0.5 mmol) was added via syringe, and the reaction mixture was stirred at room temperature overnight. The contents of the flask were transferred to a 20-dram vial, solvent was removed under reduced pressure, the residue was redissolved in toluene and heated at 60 °C for 2 hours. Purification on column chromatography afforded a mixture of α-alkylated adduct iso-**3ff** and Cope rearrangement product **4ff** (0.035 g, calc. 1:2 [AAA]:[3,3], 0.06 mmol of [3,3]). To the resulted mixture in a 20-dram vial toluene (1 mL) and methanol (1mL) were added, the vial was placed on an ice-water bath, and $NaBH_4$ (2 equiv., 0.005 g, 0.12 mmol) was added in one portion. The mixture was stirred at 0 °C for 30 min., quenched with water, diluted with saturated aqueous NH_4Cl, and extracted with EtOAc (2x). The combined organic layers were washed with brine, dried over anhydrous Na_2SO_4, filtered, and concentrated under reduced pressure. The crude residue was placed in a conical vial followed by the addition of K_2CO_3 (2 equiv., 0.017 g, 0.12 mmol), and the vial was sealed with rubber septum. Under N_2 atmosphere, MeCN (1 mL) was added via syringe, and the reaction mixture was saturated with O_2 and kept under O_2 atmosphere (balloon). Morpholine (2 equiv., 0.010 mL, 0.12 mmol) was added via syringe at stirring, and the reaction was allowed to proceed overnight at room temperature. The reaction mixture was filtered through a celite plug, the solvent was evaporated under reduced pressure, and the crude residue was purified by silica gel column chromatography using EtOAc in hexanes as an eluent to provide the desired product in overall 14% yield (0.012 g, 0.03 mmol) as a colorless oil.

$\mathbf{R}_f$ = 0.30 in 40% EtOAc in hexanes.

^{1}H NMR (400 MHz, CD_3CN) δ 8.24 (d, *J* = 2.5 Hz, 1H), 7.63 (dd, *J* = 8.2, 2.5 Hz, 1H), 7.34 (d, *J* = 8.2 Hz, 1H), 5.62 (dq, *J* = 15.1, 6.1 Hz, 1H), 5.56 – 5.47 (m, 1H), 3.71 – 3.38 (m, 9H), 2.94 – 2.86 (m, 1H), 2.24 – 2.17 (m, 1H), 1.75 – 1.65 (m, 2H), 1.63 (dd, *J* = 6.1, 1.3 Hz, 3H), 1.59 – 1.46 (m, 2H), 1.45 – 1.32 (m, 1H), 1.25 – 1.12 (m, 1H), 1.06 – 0.96 (m, 1H), 0.62 (s, 9H).

^{13}C NMR (151 MHz, CD_3CN) δ 175.4, 150.4, 149.5, 140.0, 139.4, 134.3, 128.6, 125.1, 67.5, 48.6, 47.2, 42.7, 41.1, 40.6, 36.5, 32.9, 27.3, 25.3, 24.5, 23.8, 17.9.

HRMS (ESI$^+$): $[M + H]^+$ calculated for $C_{24}H_{36}ClN_2O_2^+$ 419.2460; found 419.2456.

HPLC: (Chiralpak IA, *i*-PrOH:hexanes = 10:90, flow rate = 1.0 mL/min, UV = 254 nm) t_R1 = 5.21 min (major) and t_R2 = 16.79 min (minor), er = 88:12.

Conjugate reduction of alkylidenemalononitriles 4

General procedure E.

An alkylidenemalononitrile **4** (1 equiv.) was dissolved in MeOH (0.1 M) and THF (0.1 M) and cooled to 0 °C in an ice-water bath. $NaBH_4$ (3 equiv.) was added, and the reaction was allowed to stir warming to room temperature. Upon

completion per TLC (1-2 hours) the reaction was quenched with water until the solution became opaque (approx. 0.05 M) and transferred to a separatory funnel. The mixture was extracted with dichloromethane (3 x 10 mL). The combined organic layers were washed with brine, dried with anhydrous Na_2SO_4, filtered, and concentrated under reduced pressure. The desired product was isolated by silica gel column chromatography with EtOAc in hexanes as an eluent unless otherwise stated.

Compound SI-3

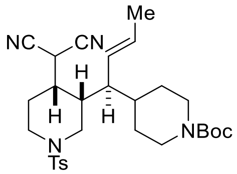

Prepared according to the General procedure E from **4b** on a 0.20 mmol scale and after aqueous work-up obtained in 94% yield (102 mg, 0.19 mmol) as a white solid without further purification.

R*f* = 0.21 in 20% EtOAc in hexanes

^{1}H NMR (400 MHz, CD_3CN) δ 7.65 (d, *J* = 8.3 Hz, 2H), 7.42 (d, *J* = 8.0 Hz, 2H), 5.57 (dq, *J* = 15.5, 6.4 Hz, 1H), 5.29 (ddd, *J* = 15.4, 10.2, 1.7 Hz, 1H), 4.18 (d, *J* = 10.7 Hz, 1H), 4.07 (t, *J* = 14.7 Hz, 2H), 3.79 – 3.63 (m, 2H), 2.76 – 2.56 (m, 2H), 2.43 (s, 4H), 2.35 (ddd, *J* = 13.5, 9.6, 3.2 Hz, 2H), 2.21 (tt, *J* = 10.7, 3.8 Hz, 1H), 2..17 – 2.10 (m, 1H, overlapped with residual water peak), 1.98 – 1.95 m, 1H, overlapped with residual NMR solvent peak), 1.92 – 1.79 (m, 2H), 1.69 (dd, *J* = 6.4, 1.6 Hz, 3H), 1.55 (d, *J* = 14.7 Hz, 1H), 1.41 (s, 9H), 1.31 – 1.15 (m, 2H), 1.07 (qd, *J* = 12.7, 4.2 Hz, 1H).

^{13}C NMR (101 MHz, CD_3CN) δ 155.4, 145.2, 133.7, 131.4, 130.7, 128.7, 114.5, 113.9, 79.6, 49.7, 46.5, 44.6, 41.9, 36.8, 36.7, 31.7, 28.6, 27.8, 26.5, 25.6, 21.5, 18.1.

HRMS (ESI-TOF): $[M+Na]^+$ calculated for $C_{29}H_{40}N_4NaO_4S$ 563.2662; found 563.2676.

Compound SI-4

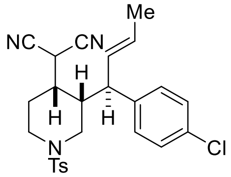

Prepared according to the General procedure E from **4a** on a 0.2 mmol scale and obtained in 81% yield (76.2 mg, 0.163 mmol) as a white solid.

R*f* = 0.34 in 20% EtOAc in hexanes.

^{1}H NMR (400 MHz, $CDCl_3$) δ 7.51 (d, *J* = 8.0 Hz, 2H), 7.34 (d, *J* = 8.3 Hz, 2H), 7.28 (d, *J* = 8.0 Hz, 2 H), 7.20 (d, *J* = 8.3 Hz 2H), 5.78 – 5.65 (m, 2H), 4.33 (d, *J* = 11.5 Hz, 1H), 3.97 (d, *J* = 11.8 Hz, 1H), 3.88 – 3.79 (m, 1H), 3.45 – 3.36 (m, 1H), 2.41 (m, 4H), 2.36 – 2.26 (m, 1H), 2.21 – 2.09 (m, 1H), 2.06 (dd, *J* = 12.0, 2.7 Hz, 1H), 2.01 – 1.91 (m, 2H), 1.69 (d, *J* = 4.3 Hz, 3H).

^{13}C NMR (101 MHz, $CDCl_3$) δ 144.2, 140.8, 133.0, 132.1, 132.0, 129.9, 129.4, 129.3, 129.2, 127.8, 112.6, 112.0, 49.8, 47.5, 46.5, 39.8, 27.3, 24.5, 21.7, 18.2.

HRMS (DART-TOF, 470 °C): $[M+NH_4]^+$ calculated for $C_{25}H_{30}ClN_4O_2S$ 485.1773; found 485.1794.

Compound SI-5

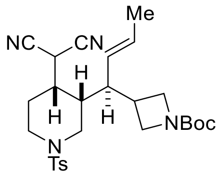

Prepared according to the General procedure E from **4c** on a 0.11 mmol scale and obtained in 93% yield (53.6 mg, 0.10 mmol) as a white solid.

$\mathbf{R}_f$ = 0.2 in 30% EtOAc in hexanes.

^{1}H NMR (400 MHz, CD_3CN) δ 7.63 (d, *J* = 8.3 Hz, 2H), 7.42 (d, *J* = 8.0 Hz, 2H), 5.74 (dq, *J* = 15.2, 6.4 Hz, 1H), 5.44 – 5.32 (m, 1H), 4.17 (d, *J* = 10.7 Hz, 1H), 3.89 (t, *J* = 8.0 Hz, 1H), 3.79 (t, *J* = 8.4 Hz, 1H), 3.63 (q, *J* = 8.0 Hz, 3H), 3.52 – 3.43 (m, 2H), 2.96 (dt, *J* = 12.6, 6.5 Hz, 1H), 2.57 – 2.39 (m, 4H), 2.31 (d, *J* = 12.1 Hz, 1H), 2.25 – 2.13 (m, 1H), 2.07 – 1.97 (m, 1H), 1.94 – 1.80 (m, 2H), 1.73 (dd, *J* = 6.4, 1.5 Hz, 3H), 1.40 (s, 9H).

^{13}C NMR (101 MHz, CD_3CN) δ 157.2, 145.3, 133.1, 132.4, 130.8, 130.2, 128.8, 114.4, 113.9, 79.5, 49.7, 46.5, 42.3, 41.5, 38.7, 30.7, 28.6, 27.6, 25.8, 21.5, 18.3.

HRMS (ESI-TOF): $[M+Na]^+$ calculated for $C_{27}H_{36}NaN_4O_4S^+$ 535.2349; found 535.2366.

Compound SI-6

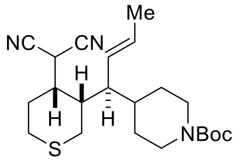

Prepared according to the General procedure E from **4p** on a 0.15 mmol scale and obtained in 91% yield (54.6 mg, 0.14 mmol) as a white solid.

$\mathbf{R}_f$ = 0.32 in 20% EtOAc in hexanes.

^{1}H NMR (400 MHz, CD_3CN) δ 5.59 (dq, *J* = 15.4, 6.4 Hz, 1H), 5.31 (ddq, *J* = 15.4, 10.4, 1.7 Hz, 1H), 4.19 (d, *J* = 10.2 Hz, 1H), 4.10 – 3.96 (m, 2H), 2.90 – 2.58 (m, 7H), 2.39 – 2.21 (m, 2H), 2.03 (ddd, *J* = 10.3, 7.1, 4.5 Hz, 2H), 1.77 – 1.60 (m, 4H), 1.58 – 1.48 (m, 1H), 1.40 (s, 9H), 1.31 – 1.18 (m, 2H), 1.08 (qd, *J* = 12.6, 4.3 Hz, 1H).

^{13}C NMR (101 MHz, CD_3CN) δ 185.5, 155.3, 130.6, 127.6, 112.7, 112.5, 85.6, 79.6, 48.1, 44.6, 36.3, 33.6, 32.7, 32.0, 30.8, 28.6, 26.2, 18.0.

HRMS (DART-TOF, 400 °C): $[M+NH_4]^+$ calculated for $C_{22}H_{37}N_4O_2S^+$ 424.2029; found 424.2048.

Conjugate reduction of alkylidenemalononitrile 4dd.

Compound SI-7

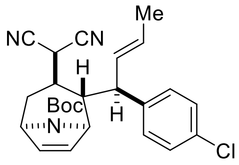

The solution of **4dd** (1 equiv., 0.218 g, 0.5 mmol) in DMPU (2.5 mL) and MeOH (2.5 mL) was cooled to 0 °C in an ice-water bath. $NaBH_4$ (0.057 g, 3 equiv.) was slowly added at stirring and the reaction vessel was removed from the ice-water bath. The reaction mixture was stirred for 1 hour, then slowly quenched with water, diluted with 2 M HCl solution and brine, and extracted with EtOAc (5x). The combined organic layers were washed with brine, dried over anhydrous Na_2SO_4, filtered, and concentrated under reduced pressure. The crude residue was purified by silica gel column chromatography using EtOAc in hexanes as eluent to provide the desired product in 52% yield (0.114 g, 0.26 mmol) as a white solid.

R*f* = 0.28 in 20% EtOAc in hexanes.

^{1}H NMR (500 MHz, DMSO-*d*$_6$, 354 K) δ 7.37 (d, *J* = 8.2 Hz, 2H), 7.27 (d, *J* = 8.2 Hz, 2H), 6.29 – 6.24 (m, 1H), 6.22 – 6.17 (m, 1H), 5.66 – 5.56 (m, 1H), 5.51 (dd, *J* = 15.5, 9.5 Hz, 1H), 4.74 (d, *J* = 10.3 Hz, 1H), 4.64 (s, 1H), 4.27 (s, 1H), 3.44 (dd, appears as t, *J* = 10.2 Hz, 1H), 2.56 – 2.50 (m, 1H), 2.24 – 2.15 (m, 1H), 1.98 (d, *J* = 10.8 Hz, 1H), 1.71 – 1.59 (m, 4H), 1.30 (s, 9H).

^{13}C NMR (126 MHz, DMSO-*d*$_6$, 354 K) δ 141.5, 134.9, 134.6, 133.2, 130.6, 129.3, 128.1, 127.2, 113.5, 113.3, 78.9, 58.4, 56.6, 50.8, 48.2, 40.2, 32.8, 30.6, 27.5, 25.4, 16.9.

HRMS (ESI$^-$): $[M - H]^-$ calculated for $C_{25}H_{27}ClN_3O_2^-$ 436.1797; found 436.1784.

Oxidative amidation and esterification of malononitriles

General procedure F.

Performed according to the modified literature procedure.[17,] An alkyl malononitrile (1 equiv.) was dissolved in MeCN (0.05 M) and DMSO (0.05 M), and K_2CO_3 (3 equiv., ground to fine powder) and a nucleophile (3 equiv.) were added. The reaction vessel was sealed, and the reaction was stirred vigorously overnight under continuous flow of O_2 (balloon) through the solution. Upon completion the reaction was diluted with EtOAc (2 × reaction volume), washed with saturated aqueous NH_4Cl (2 × 20 mL) and brine (20 mL), dried with anhydrous Na_2SO_4, filtered, and concentrated under reduced pressure. The crude residue was purified by silica gel column chromatography using EtOAc in hexanes as an eluent.

Compound 6a

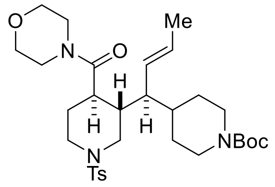

Prepared according to the General procedure F from **SI-3** and morpholine on a 0.19 mmol scale and obtained in 88% yield (98.2 mg, 0.17 mmol) as a white solid.

R*f* = 0.40 in 75% EtOAc in hexanes.

^{1}H NMR (400 MHz, CD_3CN) δ 7.65 (d, *J* = 8.3 Hz, 2H), 7.41 (d, *J* = 8.2 Hz, 2H), 5.33 (dq, *J* = 15.2, 6.3 Hz, 1H), 5.08 (ddq, *J* = 15.2, 10.2, 1.6 Hz, 1H), 4.07 – 3.98 (m, 2H), 3.65 – 3.20 (m, 10H), 2.71 – 2.32 (m, 8H), 2.16 – 2.03 (m, 1H), 1.80 – 1.63 (m, 2H), 1.64 – 1.46 (m, 7H), 1.41 (s, 9H), 1.05 (qd, *J* = 12.6, 4.1 Hz, 1H), 0.92 (qd, *J* = 12.2, 4.2 Hz, 1H).

^{13}C NMR (101 MHz, CD_3CN) δ 174.2, 155.4, 144.9, 134.3, 131.1, 130.7, 129.2, 128.7, 79.5, 67.4, 67.3, 55.3, 51.5, 49.7, 46.5, 45.9, 44.9, 42.7, 39.2, 37.8, 32.1, 29.1, 28.7, 28.6, 21.5, 18.3.

HRMS (ESI-TOF): $[M+Na]^+$ calculated for $C_{31}H_{47}NaN_3O_6S^+$ 612.3078; found 612.3102.

Compound 6b

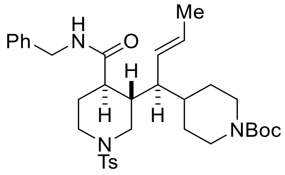

Prepared according to the General procedure F from **SI-3** and benzylamine on a 0.10 mmol scale and obtained in 79% yield (48.0 mg, 0.08 mmol) as a white solid.

R*f* = 0.39 in 50% EtOAc in hexanes.

^{1}H NMR (400 MHz, $CDCl_3$) δ 7.62 (d, *J* = 8.2 Hz, 2H), 7.35 – 7.23 (m, 1H), 7.20 (d, *J* = 6.5 Hz, 2H), 5.76 (t, *J* = 5.4 Hz, 1H), 5.37 – 5.23 (m, 1H), 5.11 (dd, *J* = 14.5, 10.7 Hz, 1H), 4.39 (dd, *J* = 14.6, 5.8 Hz, 1H), 4.29 (dd, *J* = 14.6, 5.3 Hz, 1H), 4.05 (d, *J* = 10.7 Hz, 2H), 3.37 – 3.24 (m, 2H), 2.77 – 2.48 (m, 4H), 2.43 (s, 3H), 2.16 – 2.02 (m, 3H), 1.91 – 1.74 (m, 3H), 1.65 – 1.38 (m, 14H), 1.18 – 1.04 (m, 1H), 1.03 – 0.89 (m, 1H).

^{13}C NMR (101 MHz, $CDCl_3$) δ 175.0, 155.4, 144.9, 140.4, 134.2, 130.9, 130.7, 129.3(9), 129.3(6), 128.7, 128.6, 127.9, 79.5, 51.6, 49.8, 46.1, 44.7, 43.6, 37.5, 37.2, 31.8, 30.3, 29.3, 28.6, 21.5, 18.1.

HRMS (ESI-TOF): $[M+Na]^+$ Calculated for $C_{34}H_{47}NaN_3O_5S^+$ 632.3129. Found 632.3156.

Compound 6c

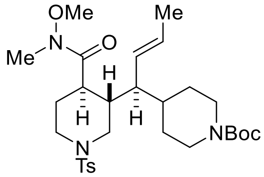

Prepared according to the General procedure F from **SI-3** and *N*,*O*-dimethylhydroxylamine hydrochloride on a 0.05 mmol scale and obtained in 53% yield (15.0 mg, 0.027 mmol) as a white solid.

R*f* = 0.38 in 50% EtOAc in hexanes.

^{1}H NMR (600 MHz, CD_3CN) δ 7.65 (d, *J* = 8.1 Hz, 2H), 7.41 (d, *J* = 8.1 Hz, 2H), 5.35 (dq, *J* = 15.0, 6.4 Hz, 1H), 5.20 – 5.12 (m, 1H), 4.02 (br, 2H), 3.60 (s, 3H), 3.42 – 3.34 (m, 1H), 3.27 – 3.22 (m, 1H), 3.04 (s, 3H), 2.74 – 2.50 (m, 5H), 2.43 (s, 3H), 2.05 – 1.98 (m, 1H), 1.85 – 1.77 (m, 2H), 1.63 – 1.55 (m, 4H), 1.55 – 1.46 (m, 3H), 1.41 (s, 9H), 1.07 (qd, *J* = 12.8, 4.2 Hz, 1H), 0.98 – 0.90 (m, 1H).

^{f13}C NMR (151 MHz, CD_3CN) δ 176.8, 155.4, 144.9, 134.3, 131.1, 130.7, 129.4, 128.7, 79.5, 62.1, 50.8, 49.0, 45.8, 38.6, 37.3, 37.2, 32.7, 32.1, 28.8, 28.6, 27.8, 21.5, 18.2.

HRMS (ESI-TOF): $[M+Na]^+$ calculated for $C_{29}H_{45}NaN_3O_6S^+$ 586.2921; found 586.2948.

Compound 6d

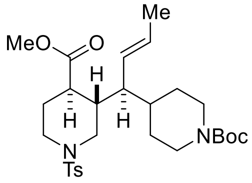

Prepared according to the General procedure F from **SI-3** and methanol on a 0.10 mmol scale and obtained in 65% yield, (34.8 mg, 0.06 mmol) as a colorless oil.

R*f* = 0.57 in 50% EtOAc in hexanes.

^{1}H NMR (400 MHz, CDCl$_3$) δ 7.62 (d, *J* = 8.2 Hz, 2H), 7.30 (d, *J* = 8.2 Hz, 2H), 5.37 – 5.23 (m, 1H), 5.11 (dd, *J* = 14.5, 10.7 Hz, 1H), 4.39 (dd, *J* = 14.6, 5.8 Hz, 1H), 4.29 (dd, *J* = 14.6, 5.3 Hz, 1H), 4.05 (d, *J* = 10.7 Hz, 3H), 3.31 (t, *J* = 12.1 Hz, 2H), 2.78 – 2.64 (m, 3H), 2.56 (dd, *J* = 31.8, 12.6 Hz, 3H), 2.43 (s, 3H), 2.17 – 2.01 (m, 1H), 1.91 – 1.74 (m, 2H), 1.59 (d, *J* = 5.6 Hz, 3H), 1.49 (dd, *J* = 23.2, 9.2 Hz, 3H), 1.44 (s, 9H), 1.17 – 1.04 (m, 1H), 1.04 – 0.90 (m, 1H).

^{13}C NMR (101 MHz, CDCl$_3$) δ 175.2, 155.0, 143.7, 133.4, 129.8, 129.5, 129.1, 127.7, 79.3, 51.9, 48.8, 47.2, 44.5, 45.2, 41.9, 36.3, 36.0, 31.4, 28.6, 27.4, 25.3, 21.6, 18.1.

HRMS (ESI-TOF): [M+Na]$^+$ calculated for $C_{28}H_{42}NaN_2O_6S^+$ 557.2656; found 557.2682.

Compound 6e

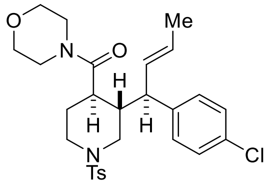

Prepared according to the General procedure F from **SI-4** and morpholine on a 0.16 mmol scale and obtained in 90% yield (75.8 mg, 0.15 mmol) as a white solid.

R*f* = 0.20 in 50% EtOAc in hexanes.

^{1}H NMR (400 MHz, CDCl$_3$) δ 7.51 (d, *J* = 8.3 Hz, 2H), 7.29 – 7.19 (m, 4H), 7.03 (d, *J* = 8.5 Hz, 2H), 5.56 – 5.38 (m, 2H), 3.84 – 3.14 (m, 11H), 2.58 – 2.35 (m, 5H), 2.34 – 2.26 (m, 1H), 2.21 (dd, *J* = 11.9, 9.1 Hz, 1H), 1.79 – 1.68 (m, 2H), 1.60 (d, *J* = 5.3 Hz, 3H).

^{13}C NMR (101 MHz, CDCl$_3$) δ 172.9, 143.7, 140.6, 133.2, 132.5, 132.4, 129.7, 129.5, 128.8, 127.7, 126.8, 66.9, 66.5, 51.1, 47.2, 45.8, 44.9, 41.7, 41.0, 39.2, 27.6, 21.6, 18.1.

HRMS (ESI-TOF): [M+Na]$^+$ calculated for $C_{27}H_{33}ClNaN_2O_4S^+$ 539.1742; found 539.1747.

Compound 6f

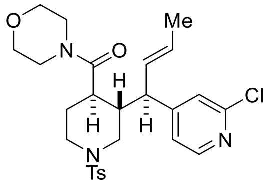

Prepared according to the sequence of the General procedure E (used after aqueous work-up) from **4i** on a 0.213 mmol scale followed by the General procedure F with addition of morpholine and obtained in 51% yield (56.4 mg, 0.107 mmol) as a white solid.

R*f* = 0.40 in 70% EtOAc in hexanes.

^{1}H NMR (400 MHz, CDCl$_3$) δ 8.30 (d, *J* = 5.1 Hz, 1H), 7.55 (d, *J* = 8.0 Hz, 2H), 7.29 (d, *J* = 8.0 Hz, 2H), 7.07 (d, *J* = 1.5 Hz, 1H), 7.02 (dd, *J* = 5.2 Hz, 1.5, 1H), 5.63 – 5.43 (m, 2H), 3.83 – 3.52 (m, 5H), 3.49 – 3.27 (m, 6H), 2.69 – 2.58 (m, 1H), 2.52 – 2.39 (m, 4H), 2.36 – 2.23 (m, 2H), 1.84 – 1.70 (m, 2H), 1.66 (dd, *J* = 6.0, 1.1 Hz, 3H).

^{13}C NMR (101 MHz, CDCl$_3$) δ 172.5, 154.6, 152.0, 150.0, 144.0, 133.2, 130.5, 129.9, 128.9, 127.8, 124.1, 122.6, 67.0, 66.6, 50.7, 46.9, 45.9, 44.9, 42.2, 40.6, 38.9, 27.3, 21.7, 18.2.

HRMS (ESI-TOF): [M+H]$^+$ calculated for $C_{26}H_{33}ClN_3O_4S^+$ 518.1875; found 518.1900.

Compound 6g

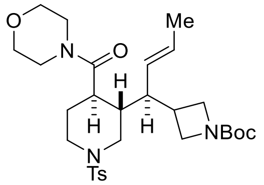

Prepared according to the General procedure F from **SI-5** on a 0.11 mmol scale and obtained in 55% yield (32.4 mg, 0.06 mmol) as a white solid.

R*f* = 0.16 in 50% EtOAc in hexanes.

^{1}H NMR (400 MHz, CD$_3$CN) δ 7.67 (d, *J* = 8.2 Hz, 2H), 7.44 (d, *J* = 8.2 Hz, 2H), 5.47 (dq, *J* = 15.0, 6.4 Hz, 1H), 5.23 – 5.12 (m, 1H), 3.81 (t, *J* = 8.2 Hz, 1H), 3.72 (t, *J* = 7.8 Hz, 1H), 3.66 – 3.50 (m, 8H), 3.48 – 3.34 (m, 4H), 2.53 – 2.38 (m, 5H), 2.31 (td, *J* = 11.7, 2.8 Hz, 1H), 2.16 (d, *J* = 11.5 Hz, 2H), 2.08 – 2.01 (m, 1H), 1.80 – 1.71 (m, 1H), 1.65 (dd, *J* = 6.4, 1.4 Hz, 3H), 1.59 – 1.46 (m, 1H), 1.42 (s, 9H).

^{13}C NMR (101 MHz, CD$_3$CN) δ 173.54, 157.19, 144.96, 134.14, 130.70, 129.71, 129.54, 128.73, 79.41, 67.36, 49.47, 49.25, 46.56, 46.19, 42.77, 39.91, 39.27, 31.04, 29.58, 28.58, 25.62, 21.53, 18.34.

HRMS (ESI-TOF): [M+Na]$^+$ calculated for $C_{29}H_{43}NaN_3O_6S^+$ 584.2765; found 584.2790.

Compound 6h

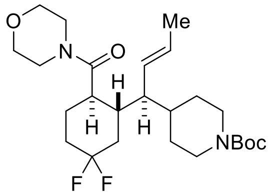

Prepared according to the General procedure F from **5a** and morpholine on a 0.12 mmol scale and obtained in 66% yield (37.4 mg, 0.08 mmol) as a white solid.

R*f* = 0.43 in 50% EtOAc in hexanes.

^{1}H NMR (400 MHz, CD$_3$CN) δ 5.35 (dq, *J* = 15.0, 6.3 Hz, 1H), 5.19 – 5.07 (m, 1H), 4.00 (d, *J* = 12.5 Hz, 2H), 3.74 – 3.38 (m, 7H), 3.29 (ddd, *J* = 12.7, 7.7, 2.9 Hz, 1H), 2.68 – 2.46 (m, 3H), 2.41 – 2.28 (m, 1H), 2.14 – 1.98 (m, 2H), 1.84 – 1.52 (m, 10H), 1.49 – 1.35 (m, 10H), 1.00 (qd, *J* = 12.6, 4.4 Hz, 1H), 0.88 (qd, *J* = 12.7, 4.3 Hz, 1H).

^{13}C NMR (101 MHz, CD$_3$CN) δ 174.5(3), 174.5(1), 155.4, 131.6, 128.8, 125.0 (t, *J* = 240.6 Hz), 79.5, 67.5, 67.4, 54.3, 46.7, 42.8, 40.5, 37.6, 37.5 (d, *J* = 9.0 Hz), 36.1 (ddd, *J* = 555.6, 23.4 Hz, 23.0 Hz), 32.1, 30.1, 28.6, 27.7 (d, *J* = 9.8 Hz), 18.4.

^{19}F NMR (377 MHz, CDCl$_3$) δ -90.4 (d, *J* = 237.0 Hz), -100.0 (d, *J* = 250.7 Hz).

HRMS (ESI-TOF): [M+Na]$^+$ calculated for $C_{25}H_{40}F_2NaN_2O_4$ 493.2848; found 493.2869.

Compound 6i

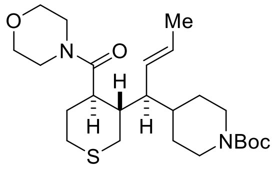

Prepared according to the General procedure F from **SI-6** and morpholine on a 0.13 mmol scale and obtained in 45% yield (27.4 mg, 0.06 mmol) as a colorless oil.

R$_f$ = 0.19 in 50% EtOAc in hexanes.

^{1}H NMR (400 MHz, CD$_3$CN) δ 5.39 (dq, *J* = 15.0, 6.3 Hz, 1H), 5.23 – 5.11 (m, 1H), 4.00 (d, *J* = 12.4 Hz, 2H), 3.69 – 3.27 (m, 8H), 2.73 – 2.44 (m, 7H), 2.21 – 2.08 (m, 1H), 2.03 – 1.95 (m, 1H), 1.85 (dq, *J* = 12.8, 6.9, 6.3 Hz, 1H), 1.75 – 1.61 (m, 4H), 1.63 – 1.42 (m, 3H), 1.40 (s, 9H), 1.06 (qd, *J* = 12.6, 4.2, 1H), 0.92 (qd, *J* = 12.6, 4.2 Hz, 1H).

^{13}C NMR (101 MHz, CD$_3$CN) δ 174.9, 155.4, 131.7, 128.8, 79.5, 67.4, 53.5, 46.7, 45.2, 42.7, 41.2, 40.2, 37.7, 32.4, 31.5, 31.3, 29.6, 28.6, 27.1, 18.3.

HRMS (ESI-TOF): [M+Na]$^+$ calculated for $C_{24}H_{40}NaN_2O_4S$ 475.2601; found 475.2607.

Compound 6j

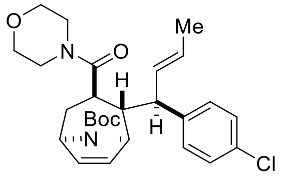

Prepared according to the General procedure F on 0.15 mmol scale from **SI-7** and morpholine (2 equiv.) with the following modifications: the reaction was performed in MeCN (0.1 M) using K_2CO_3 (2 equiv.); upon completion the reaction mixture was filtered through a celite plug and purified by silica gel column chromatography. The desired product was isolated in 71% yield (0.052 g, 0.11 mmol) as a white solid.

R$_f$ = 0.08 in 40% EtOAc in hexanes

^{1}H NMR (600 MHz, DMSO-*d$_6$*, 353 K) δ 7.36 (d, *J* = 8.4 Hz, 2H), 7.23 (d, *J* = 8.4 Hz, 2H), 6.09 (dd, *J* = 5.8, 2.3 Hz, 1H), 5.99 (dd, *J* = 5.8, 2.5 Hz, 1H), 5.47 (dq, *J* = 14.8, 6.3 Hz, 1H), 5.29 – 5.20 (m, 1H), 4.50 (d, *J* = 6.7 Hz, 1H), 4.16 (d, *J* = 1.5 Hz, 1H), 3.59 – 3.50 (m, 4H), 3.49 – 3.37 (m, 2H), 3.36 – 3.25 (m, 3H), 2.85 – 2.78 (m, 1H), 2.59 (dd, *J* = 11.0, 6.2 Hz, 1H), 2.20 – 2.11 (m, 1H), 1.55 (dd, *J* = 6.3, 1.6 Hz, 3H), 1.41 (s, 9H), 1.30 – 1.24 (m, 1H).

^{13}C NMR (151 MHz, DMSO- *d$_6$*, 353 K) δ 173.0, 153.3, 142.2, 134.6, 133.3, 130.4, 129.3, 128.1, 125.2, 78.6, 65.8, 58.3, 55.4, 53.7, 43.5, 38.8, 37.5, 27.7, 26.3, 17.1.

HRMS (DART$^+$): [M + H]$^+$ calculated for $C_{27}H_{36}ClN_2O_4^+$ 487.2358; found 487.2337.

Ring-opening metathesis: synthesis of compound 9

Compound 9

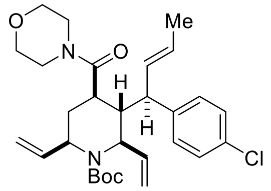

To an oven dried Schlenk flask containing a solution of **6j** (1 equiv., 0.043 g, 0.09 mmol) in dry DCM (4.5 mL, 0.02 M), Hoveyda-Grubbs II catalyst (0.003 g, 5 mol%) was added under a flow of N_2. Ethylene gas (balloon) was bubbled through a solution for 30 minutes, and the reaction proceeded at room temperature overnight under ethylene atmosphere. The reaction mixture was concentrated under reduced pressure and purified by column chromatography on silica gel to provide the desired product in 85% yield (0.035 g, 0.08 mmol) as a colorless oil.

R*f* = 0.34 in 40% EtOAc in hexanes.

^{1}H NMR (500 MHz, DMSO-*d6*, 373 K) δ 7.33 (d, *J* = 8.4 Hz, 2H), 7.18 (d, *J* = 8.4 Hz, 2H), 5.87 – 5.72 (m, 2H), 5.55 – 5.43 (m, 1H), 5.32 (dd, *J* = 14.4, 10.1 Hz, 1H), 5.08 – 4.90 (m, 4H), 4.51 (s, 1H), 4.39 – 4.30 (m, 1H), 3.63 – 3.36 (m, 8H), 3.20 – 3.13 (m, 1H), 3.04 (dd, appears as t, *J* = 10.1 Hz, 1H), 2.57 – 2.50 (m, overlaps with solvent peak at 2.50, 1H), 1.81 – 1.73 (m, 1H), 1.68 – 1.60 (m, 1H), 1.57 (d, *J* = 5.6 Hz, 3H), 1.40 (s, 9H).

^{13}C NMR (126 MHz, DMSO-*d6*, 373 K) δ 172.1, 154.1, 142.0, 141.5, 139.8, 132.6, 130.4, 129.2, 127.8, 125.2, 113.8, 113.7, 78.5, 65.6, 54.1, 53.8, 53.0, 43.2, 38.3, 29.4, 27.6, 17.0.

HRMS (ESI$^+$): $[M + H]^+$ calculated for $C_{29}H_{40}ClN_2O_4^+$ 515.2671; found 515.2689.

2-pot protocol toward compound 8

Telescoped sequence allylic alkylation/Cope rearrangement/reduction.

Compound 7

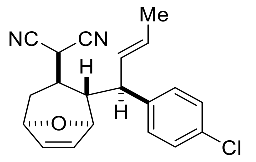

To an oven dried Schlenk flask equipped with $Pd_2(dba)_3$ (0.009 g, 1 mol%), (*S,S*)- DACH phenyl Trost ligand (0.014 g, 2 mol%), and a stir bar under N_2 atmosphere, anhydrous DCM (5 mL, 0.2 M) was added via syringe, and the contents were left at stirring for 15 minutes. The septum was removed, and an alkylidene malononitrile **1g** (1 equiv., 0.172 g, 1.0 mmol) was added under a flow of N_2. The flask was sealed with a rubber septum, and electrophile **rac-2a** (2.2 equiv., 0.529 g, 2.2 mmol) was added via syringe. The reaction mixture was stirred at room temperature for 3 hours, then heated to 40 °C, and left at this temperature for 30 minutes. The reaction vessel was cooled to room temperature and placed on an ice-water bath followed by addition of MeOH (2.5 mL) and DMPU (2.5 mL). After slow addition of $NaBH_4$ (0.057 g, 1.5 equiv. relative to **1g**) at 0 °C the reaction vessel was removed from the ice-water bath and left at room temperature for 3 hours. The reaction mixture was slowly quenched with water, diluted with 2 M HCl, and extracted with EtOAc (3x). The combined organic layers were washed with brine, dried over anhydrous Na_2SO_4, filtered, and concentrated under reduced pressure. The product was isolated by column chromatography on silica gel using EtOAc in hexanes as an eluent in 27% yield (0.091 g, 0.27 mmol) as a white solid.

R*f* = 0.33 in 20% EtOAc in hexanes.

^{1}H NMR (600 MHz, CDCl$_3$) δ 7.31 (d, *J* = 8.4 Hz, 2H), 7.18 (d, *J* = 8.4 Hz, 2H), 6.31 (dd, *J* = 6.0, 1.8 Hz, 1H), 6.16 (dd, *J* = 6.0, 2.0 Hz, 1H), 5.70 – 5.58 (m, 2H), 4.78 – 4.74 (m, 1H), 4.28 (d, *J* = 12.1 Hz, 1H), 4.23 (s, 1H), 3.62 (dd, *J* = 11.2, 8.9 Hz, 1H), 2.71 (dd, *J* = 12.1, 8.2 Hz, 1H), 2.34 – 2.26 (m, 1H), 1.73 – 1.66 (m, 5H).

^{13}C NMR (151 MHz, CDCl$_3$) δ 141.3, 134.8, 134.4, 132.6, 132.5, 129.7, 129.3, 129.1, 112.7, 112.5, 77.9, 77.4, 50.8, 40.5, 33.4, 30.9, 24.9, 18.0.

HRMS (DART$^+$): [M + NH$_4$]$^+$ calculated for $C_{20}H_{23}ClN_3O^+$ 356.1524; found 356.1528.

Ring rearrangement metathesis/oxidative amidation sequence.

Compound 8

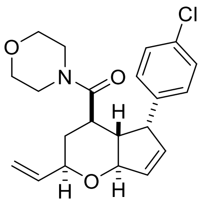

To an oven dried Schlenk flask containing a solution of **7** (1 equiv., 0.049 g, 0.14 mmol) in dry DCM (7 mL, 0.02 M), Hoveyda-Grubbs II catalyst (0.004 g, 5 mol%) was added under a flow of N$_2$, and the flask was sealed with a rubber septum. Ethylene gas (balloon) was bubbled through the solution for 2.5 hours at room temperature until the completion of the reaction based on TLC. The reaction mixture was concentrated, K_2CO_3 (2 equiv., 0.039 g, 0.28 mmol) was added to the reaction vessel followed by MeCN (1.4 mL, 0.1 M), and the reaction mixture was saturated with O$_2$ and kept under O$_2$ atmosphere (balloon). Morpholine (2 equiv., 0.024 mL, 0.28 mmol) was added via syringe at stirring, and the reaction was allowed to proceed overnight at room temperature. Upon completion the reaction mixture was filtered through a celite plug, the solvent was evaporated under reduced pressure, and the crude residue was purified by silica gel column chromatography using EtOAc in hexanes as an eluent to provide the desired product in 38% yield (0.020 g, 0.05 mmol) as a colorless oil.

R$_f$ = 0.19 in 50% EtOAc in hexanes.

^{1}H NMR (400 MHz, CDCl$_3$) δ 7.24 (d, overlaps with solvent peak at 7.26, *J* = 8.4 Hz, 2H), 6.95 (d, *J* = 8.4 Hz, 2H), 6.34 – 6.26 (m, 1H), 6.10 – 6.02 (m, 1H), 5.91 – 5.78 (m, 1H), 5.26 (d, *J* = 17.3 Hz, 1H), 5.15 (d, *J* = 10.6 Hz, 1H), 4.33 – 4.25 (m, 1H), 4.16 – 4.05 (m, 1H), 3.97 – 3.87 (m, 1H), 3.70 – 3.54 (m, 4H), 3.53 – 3.43 (m, 1H), 3.41 – 3.31 (m, 1H), 3.20 – 3.09 (m, 1H), 3.06 – 2.94 (m, 1H), 2.65 – 2.54 (m, 1H), 2.29 – 2.17 (m, 1H), 1.68 – 1.62 (m, 1H), 1.60 – 1.49 (m, 1H).

^{13}C NMR (151 MHz, CDCl$_3$) δ 171.9, 137.5, 137.2, 135.2, 134.7, 132.6, 129.6, 128.6, 116.7, 84.8, 79.1, 67.0, 66.5, 51.1, 49.1, 45.6, 42.1, 39.2, 35.8.

HRMS (ESI$^+$): [M + H]$^+$ calculated for $C_{21}H_{25}ClNO_3^+$ 374.1517; found 374.1527.

Synthesis of lactones

General procedure G. Alkene ozonolysis followed by reduction.

Prepared according to the modified literature procedure.[9] To an oven dried Schlenk flask containing **4** (1 equiv.) and NaHCO$_3$ (0.25 equiv.) under N$_2$ atmosphere, dry MeOH (0.1 M) and dry DCM (0.02 M) were added. The reaction mixture was cooled to -78 °C in a dry ice/acetone bath, and O$_2$ was bubbled through the solution for 2 minutes followed by ozone for up to 5 minutes until the color change from colorless to blue was observed. Then N$_2$ was bubbled through the solution, and NaBH$_4$ (3 equiv.) was added in one portion. The reaction mixture was removed from the dry ice/acetone bath and left at stirring at room temperature. After 1 hour the reaction was slowly

quenched with saturated aqueous solution of NH_4Cl, the organic layer was separated, and the aqueous layer was extracted with DCM (2x). The combined organic layers were washed with brine and dried over anhydrous Na_2SO_4. Solvent was evaporated under reduced pressure and the crude residue was purified by column chromatography with EtOAc in hexanes as an eluent.

Compound 10b

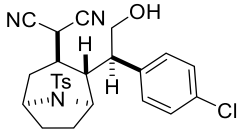

Prepared according to the General procedure G from **4w** on 0.14 mmol scale and isolated in 70% yield (0.047 g, 0.10 mmol) as a white solid.

R*f* = 0.42 in 40% EtOAc in hexanes.

^{1}H NMR (400 MHz, $CDCl_3$) δ 7.54 (d, *J* = 8.2 Hz, 2H), 7.41 – 7.28 (m, 4H), 7.24 (d, overlaps with solvent peak at 7.26, *J* = 8.2 Hz, 2H), 4.61 (d, *J* = 5.8 Hz, 1H), 4.34 – 4.26 (m, 1H), 3.89 (dd, *J* = 11.9, 3.6 Hz, 1H), 3.85 – 3.74 (m, 2H), 3.09 (ddd, appears as dt, *J* = 10.5, 3.6 Hz, 1H), 2.84 – 2.72 (m, 1H), 2.61 – 2.49 (m, 1H), 2.41 (s, 3H), 2.01 (dd, *J* = 10.5, 5.8 Hz, 1H), 1.90 (br, 1H), 1.60 – 1.47 (m, 2H), 1.46 – 1.33 (m, 2H), 1.28 – 1.19 (m, 1H).

^{13}C NMR (101 MHz, $CDCl_3$) δ 144.2, 139.9, 136.4, 133.4, 130.4, 130.1, 129.2, 127.3, 112.7, 112.6, 63.8, 57.5, 54.7, 50.2, 48.4, 33.7, 33.6, 30.9, 29.0, 28.4, 21.7.

HRMS (ESI$^+$): $[M + H]^+$ calculated for $C_{25}H_{27}ClN_3O_3S^+$ 484.1456; found 484.1461.

Compound 10c

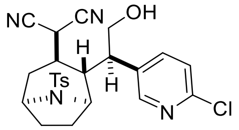

Prepared according to the General procedure G from **4z** on 0.10 mmol scale and isolated in 50% yield (0.024 g, 0.05 mmol) as a white solid.

R*f* = 0.26 in 40% EtOAc in hexanes.

^{1}H NMR (400 MHz, $CDCl_3$) δ 8.44 (d, *J* = 2.4 Hz, 1H), 7.79 (dd, *J* = 8.3, 2.4 Hz, 1H), 7.53 (d, *J* = 8.2 Hz, 2H), 7.34 (d, *J* = 8.3 Hz, 1H), 7.24 (d, *J* = 8.2 Hz, 2H), 4.77 (d, *J* = 5.6 Hz, 1H), 4.38 – 4.23 (m, 1H), 3.97 (dd, *J* = 12.0, 3.2 Hz, 1H), 3.82 (dd, *J* = 12.0, 3.2 Hz, 1H), 3.68 (d, *J* = 8.0 Hz, 1H), 3.58 – 3.03 (m, 2H), 2.90 – 2.72 (m, 1H), 2.64 – 2.50 (m, 1H), 2.40 (s, 3H), 2.12 – 1.98 (m, 1H), 1.63 – 1.48 (m, 2H), 1.48 – 1.35 (m, 2H), 1.22 – 1.06 (m, 1H).

^{13}C NMR (101 MHz, $CDCl_3$) δ 150.2, 149.8, 144.4, 140.6, 136.7, 136.2, 130.2, 127.2, 124.6, 112.7, 112.6, 62.6, 57.4, 54.7, 47.9(2), 47.8(7), 33.7, 33.5, 30.9, 28.8, 28.4, 21.7.

HRMS (ESI$^+$): $[M + H]^+$ calculated for $C_{24}H_{26}ClN_4O_3S^+$ 485.1409; found 485.1420.

Compound 11a

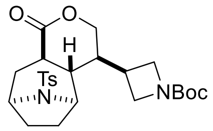

Prepared according to the sequence of the General procedure G from **4v** followed by the General procedure F on 0.1 mmol scale with the following modifications: upon completion of ozonolysis the reaction was quenched with Me_2S (0.022 mL, 0.3 mmol, 3 equiv.) to provide the corresponding aldehyde. Purification by column chromatography resulted in a contaminated sample (0.031 g) that was dissolved in MeOH (0.6 mL) and DCM (3 mL). The solution was cooled to 0 °C in an ice-water bath, $NaBH_4$ (5 equiv.,0.011 g, 0.3 mmol) was added in one portion, and the flask was removed from the bath. After 1 hour the reaction mixture was quenched with water, diluted with 2 M HCl, and extracted with EtOAc (2x). The combined organic layers were washed with brine, dried over Na_2SO_4, filtered, and solvent was evaporated under reduced pressure. The crude residue (0.025 g) was used without further purification to prepare the desired compound according to the General procedure F with the following modifications: the reaction was performed in MeCN (0.1 M) using K_2CO_3 (2 equiv., 0.011 g, 0.08 mmol) without an addition of nucleophile; upon completion the reaction mixture was filtered through a celite plug and purified by preparative TLC on silica gel. Compound **11a** was isolated in 30% yield (over 3 steps, 0.014 g, 0.03 mmol) as a white solid.

Note: the absolute stereochemistry was assigned via analogy to **11b**.

R*f* = 0.47 in 60% EtOAc in hexanes.

^{1}H NMR (400 MHz, CD_3CN) δ 7.74 (d, *J* = 8.2 Hz, 2H), 7.36 (d, *J* = 8.2 Hz, 2H), 4.33 (dd, *J* = 12.5, 6.2 Hz, 1H), 4.30 – 4.22 (m, 1H), 4.08 (dd, *J* = 12.5, 1.8 Hz, 1H), 4.05 – 4.00 (m, 1H), 3.98 – 3.85 (m, 2H), 3.66 – 3.51 (m, 2H), 2.77 – 2.65 (m, 1H), 2.62 – 2.42 (m, 2H), 2.41 (s, 3H), 2.25 – 2.16 (m, 1H), 1.61 – 1.42 (m, 4H), 1.41 (s, 9H), 1.37 – 1.31 (m, 1H), 1.22 – 1.11 (m, 1H).

^{13}C NMR (101 MHz, CD_3CN) δ 176.0, 157.1, 145.0, 138.5, 130.8, 128.0, 79.7, 67.4, 61.6, 54.8, 52.9 (br), 47.8, 44.0, 35.6, 33.4, 32.3, 32.2, 32.1, 28.6, 21.5.

HRMS (ESI$^+$): $[M + Na]^+$ calculated for $C_{25}H_{34}N_2O_6SNa^+$ 513.2030; found 513.2043.

Compound 11b

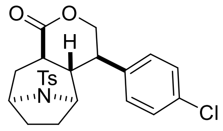

Prepared according to the General procedure F from **10b** on 0.10 mmol scale without an addition of nucleophile with the following modifications: the reaction was performed in MeCN (0.1 M) using K_2CO_3 (2 equiv.); upon completion the reaction mixture was filtered through a celite plug and purified by silica gel column chromatography. The desired product was isolated in 79% yield (0.035 g, 0.08 mmol) as a white solid.

Note: the absolute stereochemistry was assigned based on the crystal structure of **11b**.

R*f* = 0.25 in 40% EtOAc in hexanes.

^{1}H NMR (400 MHz, $CDCl_3$) δ 7.66 (d, *J* = 8.2 Hz, 2H), 7.35 (d, *J* = 8.4 Hz, 2H), 7.27 (d, overlaps with solvent peak at 7.26, *J* = 8.2 Hz, 2H), 7.17 (d, *J* = 8.4 Hz, 2H), 4.55 (dd, *J* = 12.2, 7.2 Hz, 1H), 4.36 – 4.27 (m, 1H), 4.24 (dd, *J* = 12.2, 6.5 Hz, 1H), 3.82 (d, *J* = 6.3 Hz, 1H), 3.25 – 3.14 (m, 1H), 2.81 – 2.69 (m, 1H), 2.64 – 2.51 (m, 1H), 2.41 (s, 3H), 1.76 – 1.65 (m, 1H), 1.62 – 1.44 (m, 2H), 1.47 – 1.33 (m, 2H), 1.26 – 1.18 (m, 1H).

^{13}C NMR (151 MHz, $CDCl_3$) δ 174.2, 143.9, 138.7, 137.3, 133.8, 130.0, 129.6, 129.4, 127.2, 72.7, 57.6, 54.0, 50.4, 46.5, 37.3, 32.9, 31.8, 21.7.

HRMS (ESI⁻): $[M - H]^-$ calculated for $C_{23}H_{23}ClNO_4S^-$ 444.1042; found 444.1045.

Compound 11c

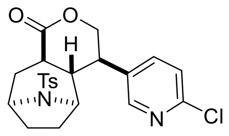

Prepared according to the General procedure F from **10c** on 0.05 mmol scale without an addition of nucleophile with the following modifications: the reaction was performed in MeCN (0.5 mL, 0.1 M) using K_2CO_3 (2 equiv., 0.014 g, 0.1 mmol); upon completion the reaction mixture was filtered through a celite plug and purified by silica gel column chromatography. The desired product was isolated in 55% yield (0.012 g, 0.03 mmol) as a white solid.

Note: the absolute stereochemistry was assigned via analogy to **11b**.

R_f = 0.24 in 40% EtOAc in hexanes.

^{1}H NMR (400 MHz, $CDCl_3$) δ 8.30 (d, *J* = 2.6 Hz, 1H), 7.67 (d, *J* = 8.2 Hz, 2H), 7.59 (dd, *J* = 8.2, 2.6 Hz, 1H), 7.38 (d, *J* = 8.2 Hz, 1H), 7.28 (d, *J* = 8.2 Hz, 2H), 4.61 (dd, *J* = 12.4, 7.3 Hz, 1H), 4.38 – 4.29 (m, 1H), 4.26 (dd, *J* = 12.4, 5.3 Hz, 1H), 3.82 (d, *J* = 5.6 Hz, 1H), 3.34 – 3.23 (m, 1H), 2.83 – 2.70 (m, 1H), 2.71 – 2.59 (m, 1H), 2.42 (s, 3H), 1.71 – 1.63 (m, 1H), 1.56 – 1.49 (m, 2H), 1.47 – 1.41 (m, 1H), 1.41 – 1.35 (m, 1H), 1.25 – 1.18 (m, 1H).

^{13}C NMR (101 MHz, $CDCl_3$) δ 174.1, 151.4, 149.2, 144.1, 138.1, 137.1, 135.5, 130.1, 127.1, 125.3, 71.7, 57.7, 54.0, 50.9, 43.8, 37.0, 32.5, 31.8, 31.7, 21.7.

HRMS (ESI⁺): $[M + H]^+$ calculated for $C_{22}H_{24}ClN_2O_4S^+$ 447.1140; found 447.1150.

X-ray Crystallography data

Crystals of **4y** and **11b** were obtained by slow evaporation of *i*PrOH/hexanes/EtOAc solution at room temperature.

X-Ray Intensity data were collected by the Center for X-Ray Crystallography of the University of Florida on a Bruker Dual micro source D8 Venture diffractometer and PHOTON III detector running APEX3 software package of programs and using MoKα radiation (λ = 0.71073 Å). The data frames were integrated, and multi-scan scaling was applied in APEX3. Intrinsic phasing structure solution provided all of the non-H atoms. The structure was refined using full-matrix least-squares refinement.[18] The non-H atoms were refined with anisotropic displacement parameters and all of the H atoms were calculated in idealized positions and refined riding on their parent atoms.

Crystal structure data for compound 4y.

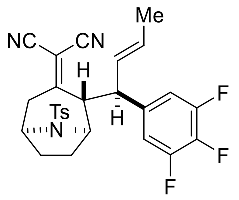

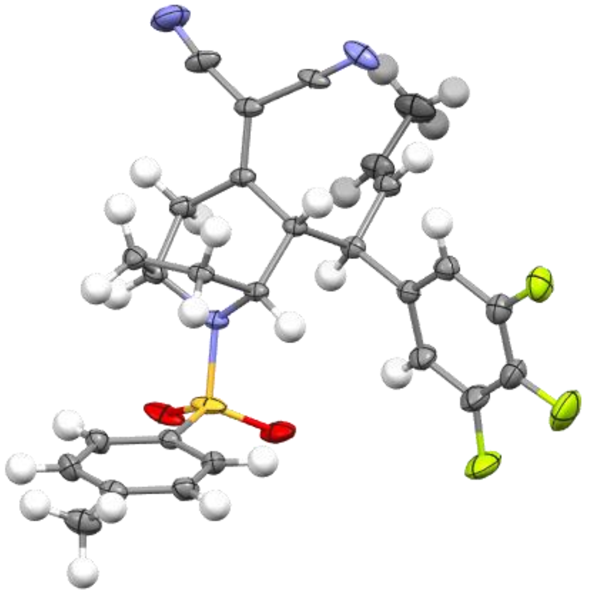

The molecule has three disordered groups: SO_2, CN and the F atoms. Two parts are refined in each case with their site occupation parameters fixed after being fully refined. In the final cycle of refinement, 8307 reflections (of which 6880 are observed with $I > 2\sigma(I)$) were used to refine 393 parameters and the resulting R_1, wR_2 and S (goodness of fit) were 3.70%, 10.09% and 1.043, respectively. The refinement was carried out by minimizing the wR_2 function using F^2 rather than F values. R_1 is calculated to provide a reference to the conventional R value but its function is not minimized.

Table S2. Crystal data and structure refinement for **4y**.

CCDC	2193762	
Empirical formula	$C_{27} H_{24} F_3 N_3 O_2 S$	
Formula weight	511.55	
Temperature	173(2) K	
Wavelength	0.71073 Å	
Crystal system	Orthorhombic	
Space group	P 21 21 21	
Unit cell dimensions	a = 7.4828(3) Å	α= 90°.
	b = 18.3087(7) Å	β= 90°.
	c = 18.4701(7) Å	γ = 90°.
Volume	2530.41(17) $Å^3$	
Z	4	
Density (calculated)	1.343 Mg/m^3	
Absorption coefficient	0.180 mm^{-1}	
F(000)	1064	
Crystal size	0.352 x 0.198 x 0.128 mm^3	
Theta range for data collection	2.205 to 32.461°.	
Index ranges	$-10\leq h\leq 6$, $-27\leq k\leq 26$, $-26\leq l\leq 27$	
Reflections collected	43297	
Independent reflections	8307 [R(int) = 0.0301]	
Completeness to theta = 25.242°	99.9 %	
Absorption correction	multi-scan	
Refinement method	Full-matrix least-squares on F^2	

Data / restraints / parameters	8307 / 804 / 393
Goodness-of-fit on F^2	1.043
Final R indices [I>2sigma(I)]	R1 = 0.0370, wR2 = 0.1009 [6880]
R indices (all data)	R1 = 0.0491, wR2 = 0.1096
Absolute structure parameter	-0.004(15)
Extinction coefficient	n/a
Largest diff. peak and hole	0.303 and -0.146 e.Å^{-3}

$R1 = \sum(||F_O| - |F_C||) / \sum|F_O|$ $wR2 = [\sum[w(F_O^2 - F_C^2)^2] / \sum[w(F_O^2)^2]]^{1/2}$

$S = [\sum[w(F_O^2 - F_C^2)^2] / (n-p)]^{1/2}$ $w= 1/[\sigma^2(F_O^2)+(m*p)^2+n*p]$, $p = [max(F_O^2,0)+ 2* F_C^2]/3$, m & n are constants.

Table S3. Atomic coordinates (x 10^4) and equivalent isotropic displacement parameters (Å^{2}x 10^3) for **4y**. U(eq) is defined as one third of the trace of the orthogonalized U^{ij} tensor.

	x	y	z	U(eq)
F1	7820(30)	3303(13)	1471(11)	76(2)
F2	4510(30)	2854(7)	1204(9)	127(5)
F3	1654(16)	3547(10)	1837(10)	118(5)
F1'	7760(40)	3199(15)	1596(14)	93(5)
F2'	4370(20)	2810(6)	1273(9)	84(3)
F3'	1676(19)	3702(12)	1663(12)	98(4)
N1	4243(2)	5055(1)	4424(1)	39(1)
C1	5034(3)	5536(1)	4983(1)	44(1)
C2	5871(3)	6164(1)	4558(1)	46(1)
C3	7217(2)	5886(1)	4023(1)	38(1)
C4	6792(2)	5189(1)	3615(1)	36(1)
C5	5800(2)	4657(1)	4126(1)	36(1)
C6	6923(2)	4463(1)	4798(1)	41(1)
C7	6465(3)	5060(1)	5355(1)	47(1)
C8	8753(3)	6255(1)	3915(1)	46(1)
C9	9270(30)	6832(7)	4289(12)	61(3)
N2	9730(20)	7371(8)	4522(11)	97(5)
C10	10030(20)	5972(12)	3322(11)	50(3)
N3	11010(30)	5790(12)	2885(11)	70(4)
C9'	9050(20)	6991(6)	4298(10)	49(2)
N2'	9300(20)	7512(7)	4617(9)	71(2)
C10'	10060(30)	6087(13)	3453(11)	47(2)
N3'	11150(30)	5954(12)	3044(11)	66(3)
C11	5647(2)	5351(1)	2922(1)	40(1)
C12	5349(2)	4665(1)	2480(1)	42(1)
C13	3608(1)	4450(1)	2328(1)	58(1)
C14	3296(2)	3836(1)	1904(1)	74(1)
C15	4726(2)	3437(1)	1631(1)	70(1)
C16	6468(2)	3652(1)	1783(1)	60(1)
C17	6779(1)	4266(1)	2207(1)	50(1)
C18	6455(3)	5933(1)	2442(1)	48(1)
C19	5821(4)	6592(1)	2367(2)	61(1)
C20	6549(5)	7154(2)	1855(2)	86(1)
C21	2715(2)	3893(1)	5144(1)	38(1)
C22	2673(2)	3984(1)	5891(1)	49(1)
C23	3030(2)	3395(1)	6344(1)	54(1)
C24	3430(2)	2716(1)	6049(1)	48(1)

C25	3472(2)	2625(1)	5302(1)	51(1)
C26	3115(2)	3214(1)	4850(1)	48(1)
C27	3764(4)	2063(2)	6542(2)	72(1)
S3	2370(5)	4599(2)	4552(2)	58(1)
O1	1212(11)	5119(6)	4869(8)	69(3)
O2	1940(20)	4263(8)	3876(7)	76(3)
S3'	2325(5)	4672(2)	4572(2)	37(1)
O1'	1247(15)	5180(5)	4998(8)	58(2)
O2'	1704(17)	4400(7)	3890(5)	47(1)

Table S4. Bond lengths [Å] and angles [°] for **4y**.

F1-C16	1.325(11)
F2-C15	1.337(9)
F3-C14	1.344(10)
F1'-C16	1.322(14)
F2'-C15	1.350(10)
F3'-C14	1.314(11)
N1-C5	1.480(2)
N1-C1	1.481(2)
N1-S3'	1.621(4)
N1-S3	1.649(4)
C1-C2	1.527(3)
C1-C7	1.543(3)
C1-H1A	1.0000
C2-C3	1.500(3)
C2-H2A	0.9900
C2-H2B	0.9900
C3-C8	1.348(3)
C3-C4	1.515(2)
C4-C5	1.547(2)
C4-C11	1.569(2)
C4-H4A	1.0000
C5-C6	1.540(2)
C5-H5A	1.0000
C6-C7	1.539(3)
C6-H6A	0.9900
C6-H6B	0.9900
C7-H7A	0.9900
C7-H7B	0.9900
C8-C9	1.321(15)
C8-C10'	1.333(17)
C8-C9'	1.537(11)
C8-C10	1.545(14)

C9-N2	1.131(12)
C10-N3	1.137(12)
C9'-N2'	1.137(11)
C10'-N3'	1.140(13)
C11-C18	1.512(3)
C11-C12	1.515(2)
C11-H11A	1.0000
C12-C13	1.3900
C12-C17	1.3900
C13-C14	1.3900
C13-H13A	0.9500
C14-C15	1.3900
C15-C16	1.3900
C16-C17	1.3900
C17-H17A	0.9500
C18-C19	1.304(3)
C18-H18A	0.9500
C19-C20	1.500(3)
C19-H19A	0.9500
C20-H20A	0.9800
C20-H20B	0.9800
C20-H20C	0.9800
C21-C22	1.3900
C21-C26	1.3900
C21-S3	1.712(5)
C21-S3'	1.799(4)
C22-C23	1.3900
C22-H22A	0.9500
C23-C24	1.3900
C23-H23A	0.9500
C24-C25	1.3900
C24-C27	1.523(2)
C25-C26	1.3900
C25-H25A	0.9500
C26-H26A	0.9500
C27-H27A	0.9800
C27-H27B	0.9800
C27-H27C	0.9800
S3-O1	1.415(9)
S3-O2	1.429(11)
S3'-O2'	1.432(10)
S3'-O1'	1.461(9)
C5-N1-C1	103.71(13)

C5-N1-S3'	123.15(17)
C1-N1-S3'	119.53(16)
C5-N1-S3	118.22(19)
C1-N1-S3	122.70(19)
N1-C1-C2	104.66(15)
N1-C1-C7	104.64(15)
C2-C1-C7	111.69(16)
N1-C1-H1A	111.8
C2-C1-H1A	111.8
C7-C1-H1A	111.8
C3-C2-C1	111.01(15)
C3-C2-H2A	109.4
C1-C2-H2A	109.4
C3-C2-H2B	109.4
C1-C2-H2B	109.4
H2A-C2-H2B	108.0
C8-C3-C2	119.99(17)
C8-C3-C4	121.82(18)
C2-C3-C4	118.18(15)
C3-C4-C5	109.13(14)
C3-C4-C11	111.21(13)
C5-C4-C11	110.81(14)
C3-C4-H4A	108.5
C5-C4-H4A	108.5
C11-C4-H4A	108.5
N1-C5-C6	104.14(14)
N1-C5-C4	107.09(13)
C6-C5-C4	112.04(13)
N1-C5-H5A	111.1
C6-C5-H5A	111.1
C4-C5-H5A	111.1
C7-C6-C5	104.69(14)
C7-C6-H6A	110.8
C5-C6-H6A	110.8
C7-C6-H6B	110.8
C5-C6-H6B	110.8
H6A-C6-H6B	108.9
C6-C7-C1	104.93(15)
C6-C7-H7A	110.8
C1-C7-H7A	110.8
C6-C7-H7B	110.8
C1-C7-H7B	110.8
H7A-C7-H7B	108.8

C9-C8-C3	125.0(11)
C10'-C8-C3	127.2(9)
C10'-C8-C9'	113.0(12)
C3-C8-C9'	119.6(8)
C9-C8-C10	117.2(13)
C3-C8-C10	117.8(8)
N2-C9-C8	171(2)
N3-C10-C8	177(2)
N2'-C9'-C8	176.0(17)
N3'-C10'-C8	178(3)
C18-C11-C12	109.11(15)
C18-C11-C4	113.15(15)
C12-C11-C4	111.36(13)
C18-C11-H11A	107.7
C12-C11-H11A	107.7
C4-C11-H11A	107.7
C13-C12-C17	120.0
C13-C12-C11	118.79(10)
C17-C12-C11	121.18(10)
C12-C13-C14	120.0
C12-C13-H13A	120.0
C14-C13-H13A	120.0
F3'-C14-C15	119.3(12)
F3-C14-C15	117.7(9)
F3'-C14-C13	119.8(11)
F3-C14-C13	121.5(9)
C15-C14-C13	120.0
F2-C15-C16	117.2(8)
F2'-C15-C16	121.5(7)
F2-C15-C14	122.7(8)
F2'-C15-C14	118.3(7)
C16-C15-C14	120.0
F1'-C16-C15	117.3(17)
F1-C16-C15	119.3(14)
F1'-C16-C17	122.1(17)
F1-C16-C17	120.4(14)
C15-C16-C17	120.0
C16-C17-C12	120.0
C16-C17-H17A	120.0
C12-C17-H17A	120.0
C19-C18-C11	124.7(2)
C19-C18-H18A	117.7
C11-C18-H18A	117.7

C18-C19-C20	124.7(3)
C18-C19-H19A	117.7
C20-C19-H19A	117.7
C19-C20-H20A	109.5
C19-C20-H20B	109.5
H20A-C20-H20B	109.5
C19-C20-H20C	109.5
H20A-C20-H20C	109.5
H20B-C20-H20C	109.5
C22-C21-C26	120.0
C22-C21-S3	122.74(16)
C26-C21-S3	117.22(16)
C22-C21-S3'	119.01(12)
C26-C21-S3'	120.95(12)
C21-C22-C23	120.0
C21-C22-H22A	120.0
C23-C22-H22A	120.0
C24-C23-C22	120.0
C24-C23-H23A	120.0
C22-C23-H23A	120.0
C23-C24-C25	120.0
C23-C24-C27	120.27(15)
C25-C24-C27	119.70(15)
C24-C25-C26	120.0
C24-C25-H25A	120.0
C26-C25-H25A	120.0
C25-C26-C21	120.0
C25-C26-H26A	120.0
C21-C26-H26A	120.0
C24-C27-H27A	109.5
C24-C27-H27B	109.5
H27A-C27-H27B	109.5
C24-C27-H27C	109.5
H27A-C27-H27C	109.5
H27B-C27-H27C	109.5
O1-S3-O2	120.7(8)
O1-S3-N1	103.8(5)
O2-S3-N1	106.6(7)
O1-S3-C21	109.7(6)
O2-S3-C21	105.6(6)
N1-S3-C21	110.2(3)
O2'-S3'-O1'	121.1(8)
O2'-S3'-N1	106.8(6)

O1'-S3'-N1	107.8(5)
O2'-S3'-C21	107.1(5)
O1'-S3'-C21	106.1(6)
N1-S3'-C21	107.4(2)

Table S5. Anisotropic displacement parameters ($Å^2$x 10^3) for **4y**. The anisotropic displacement factor exponent takes the form: $-2\pi^2[h^2 a^{*2}U^{11} + ... + 2 h k a^* b^* U^{12}]$

	**U11**	**U22**	**U33**	**U23**	**U13**	**U12**
F1	78(4)	81(5)	69(3)	-21(3)	7(3)	16(3)
F2	148(10)	125(7)	107(6)	-71(6)	-9(6)	-22(6)
F3	64(4)	162(12)	129(9)	-68(7)	1(4)	-47(6)
F1'	88(7)	85(8)	106(13)	-43(8)	-6(7)	25(6)
F2'	88(5)	68(4)	97(6)	-27(4)	-16(4)	-17(4)
F3'	73(5)	109(5)	111(8)	-29(5)	-46(6)	-14(4)
N1	25(1)	42(1)	50(1)	7(1)	0(1)	-2(1)
C1	36(1)	47(1)	49(1)	-1(1)	3(1)	-1(1)
C2	42(1)	37(1)	59(1)	-3(1)	3(1)	-2(1)
C3	31(1)	36(1)	47(1)	6(1)	-5(1)	-2(1)
C4	27(1)	35(1)	44(1)	4(1)	-1(1)	1(1)
C5	28(1)	35(1)	44(1)	4(1)	-3(1)	-2(1)
C6	30(1)	45(1)	47(1)	9(1)	-4(1)	0(1)
C7	42(1)	53(1)	45(1)	4(1)	-5(1)	-6(1)
C8	31(1)	47(1)	58(1)	15(1)	-9(1)	-7(1)
C9	56(6)	37(5)	88(5)	17(4)	-19(4)	-12(4)
N2	101(10)	69(7)	121(8)	-7(5)	-8(7)	-40(6)
C10	22(3)	56(6)	71(8)	23(5)	3(5)	-2(3)
N3	43(5)	79(8)	87(9)	33(5)	22(6)	17(5)
C9'	42(4)	32(4)	73(4)	12(4)	-20(3)	-11(4)
N2'	73(6)	50(4)	89(5)	-8(3)	-24(5)	-17(3)
C10'	35(3)	53(6)	55(5)	16(3)	-8(3)	-4(3)
N3'	41(3)	82(9)	74(6)	25(4)	6(3)	7(4)
C11	34(1)	42(1)	44(1)	7(1)	-2(1)	2(1)
C12	39(1)	46(1)	40(1)	6(1)	-3(1)	-2(1)
C13	42(1)	75(1)	58(1)	-9(1)	-9(1)	-3(1)
C14	57(2)	91(2)	74(2)	-20(1)	-14(1)	-16(1)
C15	82(2)	70(2)	59(1)	-16(1)	-10(1)	-11(1)
C16	64(2)	62(1)	55(1)	-7(1)	-1(1)	5(1)
C17	46(1)	55(1)	49(1)	2(1)	0(1)	-1(1)
C18	44(1)	50(1)	50(1)	14(1)	-5(1)	-5(1)
C19	50(1)	57(1)	76(2)	23(1)	-2(1)	2(1)
C20	75(2)	75(2)	107(2)	48(2)	-2(2)	-5(2)

C21	29(1)	44(1)	43(1)	4(1)	2(1)	-6(1)
C22	52(1)	47(1)	47(1)	-2(1)	13(1)	0(1)
C23	62(1)	62(1)	39(1)	4(1)	6(1)	-4(1)
C24	37(1)	51(1)	57(1)	12(1)	2(1)	-6(1)
C25	52(1)	40(1)	62(1)	-3(1)	9(1)	-6(1)
C26	52(1)	48(1)	44(1)	-4(1)	5(1)	-12(1)
C27	62(2)	64(1)	90(2)	32(1)	1(1)	-4(1)
S3	23(1)	68(2)	83(2)	32(1)	-12(1)	-13(1)
O1	15(2)	83(6)	108(6)	46(4)	8(3)	10(3)
O2	57(6)	95(7)	76(4)	33(4)	-36(4)	-38(5)
S3'	28(1)	39(1)	45(1)	8(1)	1(1)	1(1)
O1'	46(4)	43(2)	85(4)	4(3)	15(3)	7(2)
O2'	32(2)	58(3)	51(2)	21(2)	-13(2)	-10(2)

Table S6. Hydrogen coordinates (x 10^4) and isotropic displacement parameters (Å^{2}x 10 ^{3}) for **4y**.

	**x**	**y**	**z**	**U(eq)**
H1A	4115	5713	5335	53
H2A	4923	6436	4299	55
H2B	6461	6505	4898	55
H4A	7941	4958	3462	43
H5A	5415	4207	3863	43
H6A	6599	3974	4985	49
H6B	8214	4469	4682	49
H7A	7536	5353	5475	56
H7B	5991	4841	5806	56
H11A	4451	5530	3086	48
H13A	2630	4723	2514	70
H17A	7970	4413	2311	60
H18A	7499	5810	2176	58
H19A	4826	6727	2658	73
H20A	5565	7371	1578	128
H20B	7394	6921	1521	128
H20C	7164	7536	2130	128
H22A	2400	4448	6093	58
H23A	3001	3457	6854	65
H25A	3746	2161	5101	61
H26A	3144	3152	4339	58
H27A	4724	1762	6338	108
H27B	2670	1771	6580	108
H27C	4114	2235	7023	108

Crystal structure data for compound 11b.

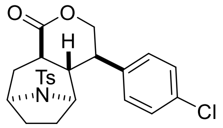

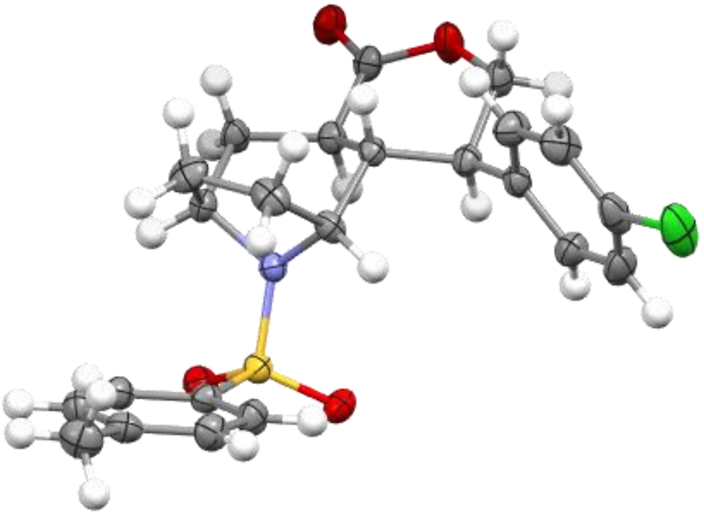

The stereochemistry is decided by anomalous dispersion and the value of the Flack x parameter: 0.01(2). In the final cycle of refinement, 6791 reflections (of which 6055 are observed with I > 2σ(I)) were used to refine 248 parameters and the resulting R_1, wR_2 and S (goodness of fit) were 3.45%, 8.18% and 1.074, respectively. The refinement was carried out by minimizing the wR_2 function using F^2 rather than F values. R_1 is calculated to provide a reference to the conventional R value but its function is not minimized.

Table S7. Crystal data and structure refinement for **11b.**

CCDC	2193763	
Empirical formula	$C_{23} H_{24} Cl N O_4 S$	
Formula weight	445.94	
Temperature	100(2) K	
Wavelength	0.71073 Å	
Crystal system	Monoclinic	
Space group	$P2_1$	
Unit cell dimensions	a = 11.1508(4) Å	α = 90°.
	b = 6.3344(2) Å	β = 99.4170(10)°.
	c = 15.0399(5) Å	γ = 90°.
Volume	1048.01(6) $Å^3$	
Z	2	
Density (calculated)	1.413 Mg/m^3	
Absorption coefficient	0.313 mm^{-1}	
F(000)	468	
Theta range for data collection	1.851 to 32.747°.	
Index ranges	-16≤h≤16, -9≤k≤9, -22≤l≤21	
Reflections collected	22288	
Independent reflections	6791 [R(int) = 0.0447]	

Completeness to theta = 25.242°	99.9 %
Absorption correction	multi-scan
Refinement method	Full-matrix least-squares on F^2
Data / restraints / parameters	6791 / 1 / 248
Goodness-of-fit on F^2	1.074
Final R indices [I>2sigma(I)]	R1 = 0.0345, wR2 = 0.0818 [6055]
R indices (all data)	R1 = 0.0426, wR2 = 0.0849
Absolute structure parameter	0.01(2)
Extinction coefficient	n/a
Largest diff. peak and hole	0.386 and -0.313 e.Å^{-3}

$R1 = \sum(||F_o| - |F_c||) / \sum|F_o|$ $wR2 = [\sum[w(F_o^2 - F_c^2)^2] / \sum[w(F_o^2)^2]]^{1/2}$

$S = [\sum[w(F_o^2 - F_c^2)^2] / (n-p)]^{1/2}$ $w= 1/[\sigma^2(F_o^2)+(m*p)^2+n*p]$, $p = [\max(F_o^2,0)+ 2* F_c^2]/3$, m & n are constants.

Table S8. Atomic coordinates (x 104) and equivalent isotropic displacement parameters (Å^{2}x 10^3) for **11b**. U(eq) is defined as one third of the trace of the orthogonalized U^{ij} tensor.

	x	y	z	U(eq)
Cl1	2756(1)	4243(1)	-604(1)	36(1)
S3	9450(1)	1896(1)	2994(1)	16(1)
O1	10385(1)	1106(2)	3685(1)	21(1)
O2	8833(1)	514(2)	2312(1)	21(1)
O3	4704(1)	1267(3)	4652(1)	23(1)
O4	6058(1)	1637(2)	5871(1)	22(1)
N1	8403(1)	2860(3)	3516(1)	15(1)
C1	8694(2)	4242(3)	4322(1)	17(1)
C2	7863(2)	3490(3)	4986(1)	18(1)
C3	6782(2)	2242(3)	4478(1)	16(1)
C4	6243(2)	3253(3)	3576(1)	15(1)
C5	7268(2)	3785(3)	3024(1)	16(1)
C6	7536(2)	6167(3)	3044(1)	22(1)
C7	8389(2)	6489(3)	3954(1)	24(1)
C8	5839(2)	1752(3)	5059(1)	17(1)
C9	4275(2)	1556(3)	3690(1)	20(1)
C10	5271(2)	1739(3)	3105(1)	17(1)
C11	4693(1)	2407(2)	2159(1)	18(1)
C12	4659(1)	1005(2)	1444(1)	22(1)
C13	4084(1)	1578(2)	587(1)	27(1)
C14	3544(1)	3553(2)	445(1)	26(1)
C15	3577(1)	4955(2)	1160(1)	27(1)
C16	4152(1)	4382(2)	2016(1)	23(1)
C17	10084(1)	3960(2)	2441(1)	16(1)
C18	11029(1)	5147(2)	2912(1)	19(1)
C19	11451(1)	6919(2)	2514(1)	21(1)
C20	10929(1)	7504(2)	1646(1)	20(1)

C21	9985(1)	6316(2)	1175(1)	22(1)
C22	9562(1)	4544(2)	1573(1)	21(1)
C23	11329(2)	9501(4)	1226(2)	28(1)

Table S9. Bond lengths [Å] and angles [°] for **11b.**

Cl1-C14	1.7321
S3-O2	1.4352(14)
S3-O1	1.4366(14)
S3-N1	1.6284(17)
S3-C17	1.7590(10)
O3-C8	1.349(2)
O3-C9	1.458(2)
O4-C8	1.208(2)
N1-C5	1.479(2)
N1-C1	1.487(2)
C1-C7	1.545(3)
C1-C2	1.545(3)
C1-H1	1.0000
C2-C3	1.536(2)
C2-H2A	0.9900
C2-H2AB	0.9900
C3-C8	1.505(3)
C3-C4	1.533(2)
C3-H3	1.0000
C4-C10	1.531(3)
C4-C5	1.556(3)
C4-H4	1.0000
C5-C6	1.537(3)
C5-H5	1.0000
C6-C7	1.548(3)
C6-H6A	0.9900
C6-H6AB	0.9900
C7-H7A	0.9900
C7-H7AB	0.9900
C9-C10	1.530(3)
C9-H9A	0.9900
C9-H9AB	0.9900
C10-C11	1.5238(19)
C10-H10	1.0000
C11-C12	1.3900
C11-C16	1.3900
C12-C13	1.3900
C12-H12	0.9500
C13-C14	1.3900

C13-H13	0.9500
C14-C15	1.3900
C15-C16	1.3900
C15-H15	0.9500
C16-H16	0.9500
C17-C18	1.3900
C17-C22	1.3900
C18-C19	1.3900
C18-H18	0.9500
C19-C20	1.3900
C19-H19	0.9500
C20-C21	1.3900
C20-C23	1.513(2)
C21-C22	1.3900
C21-H21	0.9500
C22-H22	0.9500
C23-H23A	0.9800
C23-H23B	0.9800
C23-H23C	0.9800
O2-S3-O1	120.73(9)
O2-S3-N1	106.01(8)
O1-S3-N1	105.92(8)
O2-S3-C17	107.35(8)
O1-S3-C17	107.49(8)
N1-S3-C17	108.95(8)
C8-O3-C9	123.51(15)
C5-N1-C1	103.28(14)
C5-N1-S3	121.94(12)
C1-N1-S3	122.38(12)
N1-C1-C7	104.17(15)
N1-C1-C2	105.75(15)
C7-C1-C2	113.28(17)
N1-C1-H1	111.1
C7-C1-H1	111.1
C2-C1-H1	111.1
C3-C2-C1	109.98(14)
C3-C2-H2A	109.7
C1-C2-H2A	109.7
C3-C2-H2AB	109.7
C1-C2-H2AB	109.7
H2A-C2-H2AB	108.2
C8-C3-C4	112.93(15)
C8-C3-C2	112.56(15)
C4-C3-C2	112.82(15)

C8-C3-H3	105.9
C4-C3-H3	105.9
C2-C3-H3	105.9
C10-C4-C3	107.05(15)
C10-C4-C5	114.58(15)
C3-C4-C5	110.31(14)
C10-C4-H4	108.2
C3-C4-H4	108.2
C5-C4-H4	108.2
N1-C5-C6	103.38(15)
N1-C5-C4	106.85(14)
C6-C5-C4	111.09(16)
N1-C5-H5	111.7
C6-C5-H5	111.7
C4-C5-H5	111.7
C5-C6-C7	103.65(15)
C5-C6-H6A	111.0
C7-C6-H6A	111.0
C5-C6-H6AB	111.0
C7-C6-H6AB	111.0
H6A-C6-H6AB	109.0
C1-C7-C6	105.25(15)
C1-C7-H7A	110.7
C6-C7-H7A	110.7
C1-C7-H7AB	110.7
C6-C7-H7AB	110.7
H7A-C7-H7AB	108.8
O4-C8-O3	117.67(17)
O4-C8-C3	123.64(17)
O3-C8-C3	118.52(15)
O3-C9-C10	115.42(15)
O3-C9-H9A	108.4
C10-C9-H9A	108.4
O3-C9-H9AB	108.4
C10-C9-H9AB	108.4
H9A-C9-H9AB	107.5
C11-C10-C9	108.84(14)
C11-C10-C4	114.65(15)
C9-C10-C4	107.90(15)
C11-C10-H10	108.4
C9-C10-H10	108.4
C4-C10-H10	108.4
C12-C11-C16	120.0
C12-C11-C10	119.93(11)

C16-C11-C10	120.01(11)
C11-C12-C13	120.0
C11-C12-H12	120.0
C13-C12-H12	120.0
C14-C13-C12	120.0
C14-C13-H13	120.0
C12-C13-H13	120.0
C15-C14-C13	120.0
C15-C14-Cl1	119.26(8)
C13-C14-Cl1	120.65(8)
C14-C15-C16	120.0
C14-C15-H15	120.0
C16-C15-H15	120.0
C15-C16-C11	120.0
C15-C16-H16	120.0
C11-C16-H16	120.0
C18-C17-C22	120.0
C18-C17-S3	119.42(7)
C22-C17-S3	120.23(7)
C19-C18-C17	120.0
C19-C18-H18	120.0
C17-C18-H18	120.0
C18-C19-C20	120.0
C18-C19-H19	120.0
C20-C19-H19	120.0
C21-C20-C19	120.0
C21-C20-C23	119.36(12)
C19-C20-C23	120.54(12)
C20-C21-C22	120.0
C20-C21-H21	120.0
C22-C21-H21	120.0
C21-C22-C17	120.0
C21-C22-H22	120.0
C17-C22-H22	120.0
C20-C23-H23A	109.5
C20-C23-H23B	109.5
H23A-C23-H23B	109.5
C20-C23-H23C	109.5
H23A-C23-H23C	109.5
H23B-C23-H23C	109.5

Table S10. Anisotropic displacement parameters (Å2x 103) for lova3. The anisotropic displacement factor exponent takes the form: $-2\pi^2[h^2 a^{*2}U^{11} + ... + 2 h k a^* b^* U^{12}]$.

	U^{11}	U^{22}	U^{33}	U^{23}	U^{13}	U^{12}
Cl1	26(1)	63(1)	17(1)	10(1)	-5(1)	-8(1)
S3	16(1)	15(1)	15(1)	0(1)	1(1)	2(1)
O1	18(1)	23(1)	21(1)	2(1)	0(1)	4(1)
O2	24(1)	19(1)	19(1)	-5(1)	2(1)	-1(1)
O3	20(1)	32(1)	16(1)	2(1)	0(1)	-9(1)
O4	24(1)	24(1)	16(1)	2(1)	1(1)	-5(1)
N1	14(1)	17(1)	14(1)	0(1)	0(1)	2(1)
C1	17(1)	18(1)	16(1)	-3(1)	0(1)	-2(1)
C2	16(1)	22(1)	13(1)	1(1)	1(1)	-2(1)
C3	16(1)	16(1)	15(1)	2(1)	0(1)	0(1)
C4	14(1)	15(1)	15(1)	0(1)	0(1)	1(1)
C5	14(1)	20(1)	15(1)	4(1)	2(1)	2(1)
C6	20(1)	19(1)	28(1)	7(1)	4(1)	3(1)
C7	32(1)	15(1)	25(1)	0(1)	6(1)	0(1)
C8	19(1)	15(1)	17(1)	1(1)	1(1)	-2(1)
C9	18(1)	24(1)	16(1)	0(1)	-2(1)	-4(1)
C10	17(1)	17(1)	16(1)	-2(1)	-1(1)	0(1)
C11	14(1)	25(1)	15(1)	-1(1)	0(1)	-1(1)
C12	22(1)	26(1)	19(1)	-4(1)	3(1)	-3(1)
C13	25(1)	39(1)	16(1)	-6(1)	1(1)	-6(1)
C14	16(1)	45(1)	15(1)	4(1)	-1(1)	-4(1)
C15	24(1)	35(1)	20(1)	5(1)	-1(1)	7(1)
C16	23(1)	29(1)	17(1)	-1(1)	-1(1)	6(1)
C17	17(1)	18(1)	14(1)	0(1)	2(1)	1(1)
C18	16(1)	25(1)	15(1)	-1(1)	0(1)	2(1)
C19	18(1)	24(1)	20(1)	-1(1)	2(1)	-2(1)
C20	20(1)	22(1)	20(1)	1(1)	6(1)	2(1)
C21	21(1)	27(1)	16(1)	3(1)	0(1)	2(1)
C22	19(1)	25(1)	16(1)	0(1)	-2(1)	-1(1)
C23	31(1)	28(1)	28(1)	6(1)	9(1)	0(1)

Table S11. Hydrogen coordinates (x 10^4) and isotropic displacement parameters (Å^{2}x 10^3) for **11b.**

	x	y	z	U(eq)
H1	9572	4123	4596	21
H2A	8333	2584	5456	21
H2AB	7561	4725	5287	21
H3	7119	848	4327	19
H4	5835	4601	3702	18

H5	7077	3237	2393	20
H6A	6780	6997	3020	27
H6AB	7943	6584	2532	27
H7A	9137	7246	3865	28
H7AB	7976	7310	4376	28
H9A	3770	2849	3608	24
H9AB	3744	350	3470	24
H10	5649	318	3069	20
H12	5029	-345	1541	27
H13	4061	619	99	32
H15	3208	6304	1063	32
H16	4175	5341	2505	28
H18	11385	4748	3505	23
H19	12097	7731	2836	25
H21	9628	6716	582	26
H22	8917	3732	1251	25
H23A	11606	9156	658	42
H23B	11996	10156	1640	42
H23C	10643	10486	1109	42

References

1 M. S. Abaee and S. Cheraghi, *Turkish J. Chem.*, 2014, **38**, 650–660.

2 E. Fereyduni, O. Lahtigui, J. N. Sanders, B. M. Tomiczek, M. D. Mannchen, R. A. Yu, K. N. Houk and A. J. Grenning, *J. Org. Chem.*, 2021, **86**, 2632–2643.

3 O. Lahtigui, F. Emmetiere, W. Zhang, L. Jirmo, S. Toledo-Roy, J. C. Hershberger, J. M. Macho and A. J. Grenning, *Angew. Chem. Int. Ed.*, 2016, **55**, 15792–15796.

4 S. K. Scott, J. N. Sanders, K. E. White, R. A. Yu, K. N. Houk and A. J. Grenning, *J. Am. Chem. Soc.*, 2018, **140**, 16134–16139.

5 B. Föhlisch, E. Gehrlach and R. Herter, *Angew. Chem. Int. Ed.*, 1982, **21**, 137–137.

6 E. Semenova, O. Lahtigui, S. K. Scott, M. Albritton, K. A. Abboud, I. Ghiviriga, A. E. Roitberg and A. J. Grenning, *Chem. Commun.*, 2020, **56**, 11779–11782.

7 B. M. Trost, D. L. Van Vranken and C. Bingel, *J. Am. Chem. Soc.*, 1992, **114**, 9327–9343.

8 D. R. Deardorff, C. M. Taniguchi, S. A. Tafti, H. Y. Kim, So Young Choi, K. J. Downey and T. V. Nguyen, *J. Org. Chem.*, 2001, **66**, 7191–7194.

9 G. R. Fulmer, A. J. M. Miller, N. H. Sherden, H. E. Gottlieb, A. Nudelman, B. M. Stoltz, J. E. Bercaw and K. I. Goldberg, *Organometallics*, 2010, **29**, 2176–2179.

10 G. L. Ellis, R. Amewu, S. Sabbani, P. A. Stocks, A. Shone, D. Stanford, P. Gibbons, J. Davies, L. Vivas, S. Charnaud, E. Bongard, C. Hall, K. Rimmer, S. Lozanom, M. Jesús, D. Gargallo, S. A. Ward and P. M. O'Neill, *J. Med. Chem.*, 2008, **51**, 2170–2177.

11 H. Kubas, U. Meyer, M. Hechenberger, K.-U. Klein, P. Plitt, R. Zemribo, H. W. Spexgoor, S. G. A. van Assema and U. Abel, *Bioorg. Med. Chem. Lett.*, 2013, **23**, 6370–6376.

12 H. Le, A. Batten and J. P. Morken, *Org. Lett.*, 2014, **16**, 2096–2099.

13 C. Li, J. Xing, J. Zhao, P. Huynh, W. Zhang, P. Jiang and Y. J. Zhang, *Org. Lett.*, 2012, **14**, 390–393.

14 S. Akai, R. Hanada, N. Fujiwara, Y. Kita and M. Egi, *Org. Lett.*, 2010, **12**, 4900–4903.

15 M. Tarbe, J. J. Miles, E. S. J. Edwards, K. M. Miles, A. K. Sewell, B. M. Baker and S. Quideau, *ChemMedChem*, 2020, **15**, 799–807.

16 G. Barker, D. G. Johnson, P. C. Young, S. A. Macgregor and A. L. Lee, *Chem. - A Eur. J.*, 2015, **21**, 13748–13757.

17 J. Li, M. J. Lear and Y. Hayashi, *Angew. Chem. Int. Ed.*, 2016, **55**, 9060–9064.

18 G. M. Sheldrick, *Acta Crystallogr. Sect. C Struct. Chem.*, 2015, **71**, 3–8.

NMR spectra

^{1}H NMR (400 MHz, $CDCl_3$) of **1a**

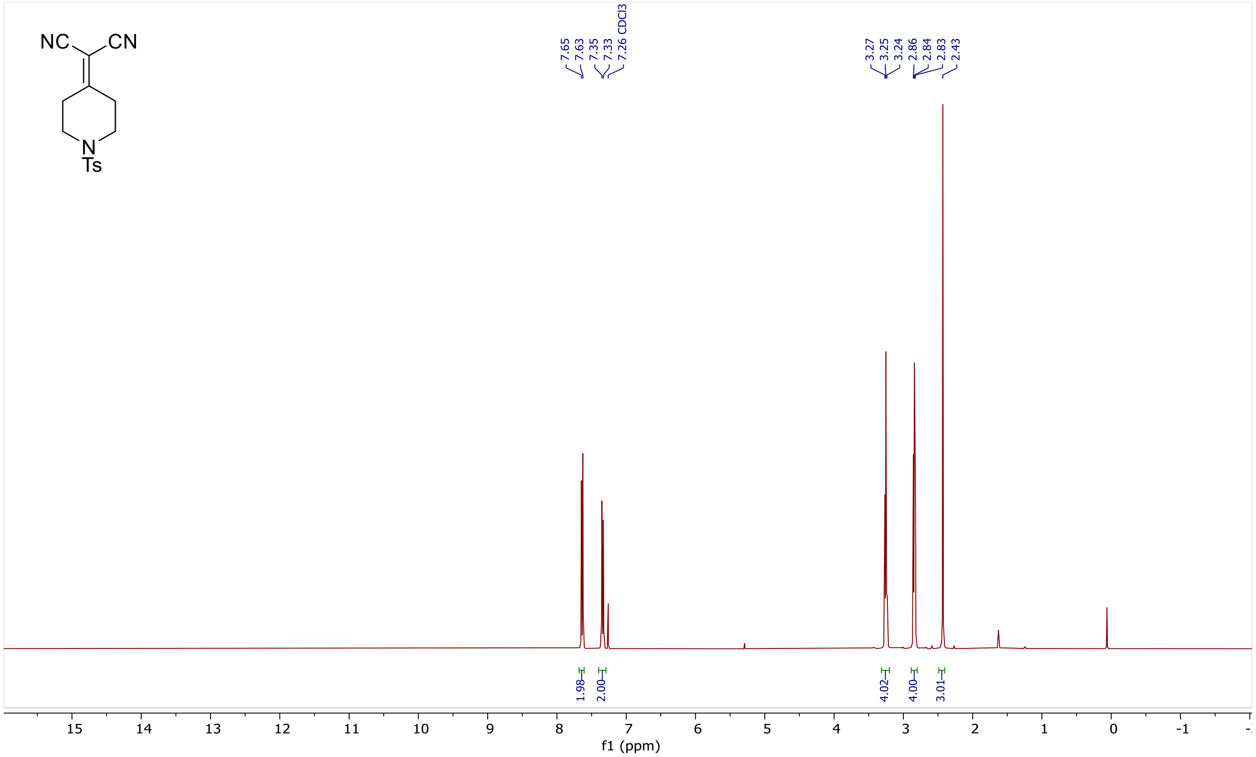

^{13}C NMR (101 MHz, $CDCl_3$) of 1a

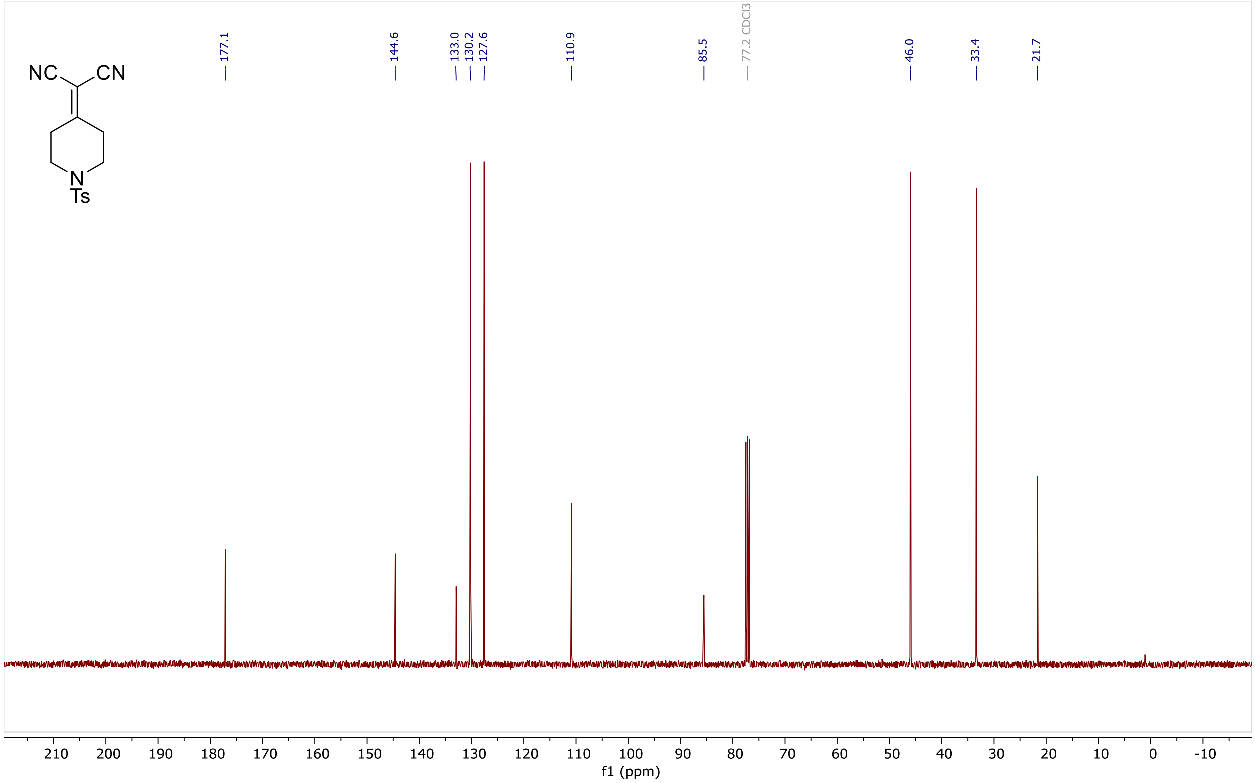

^{1}H NMR (400 MHz, $CDCl_3$) of 1f

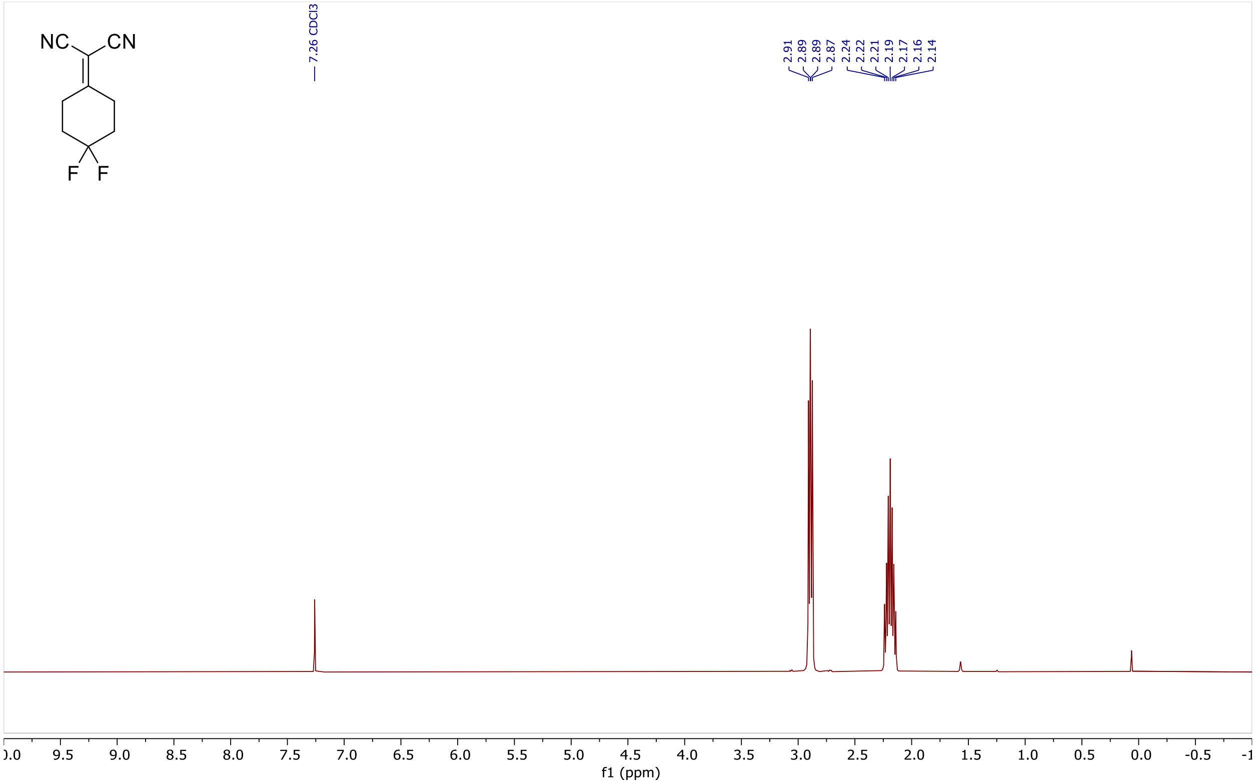

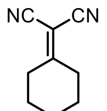

^{13}C NMR (101 MHz, $CDCl_3$) of 1f

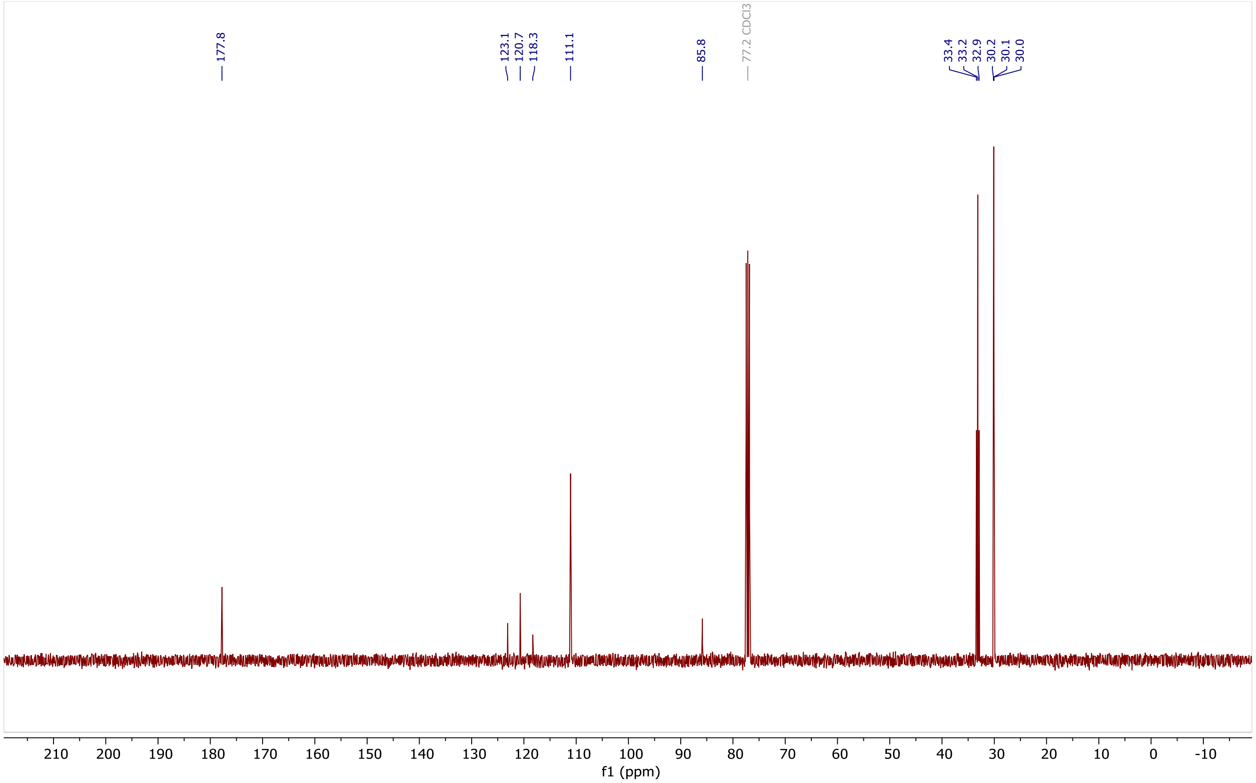

^{19}F NMR (377 MHz, $CDCl_3$) of 1f

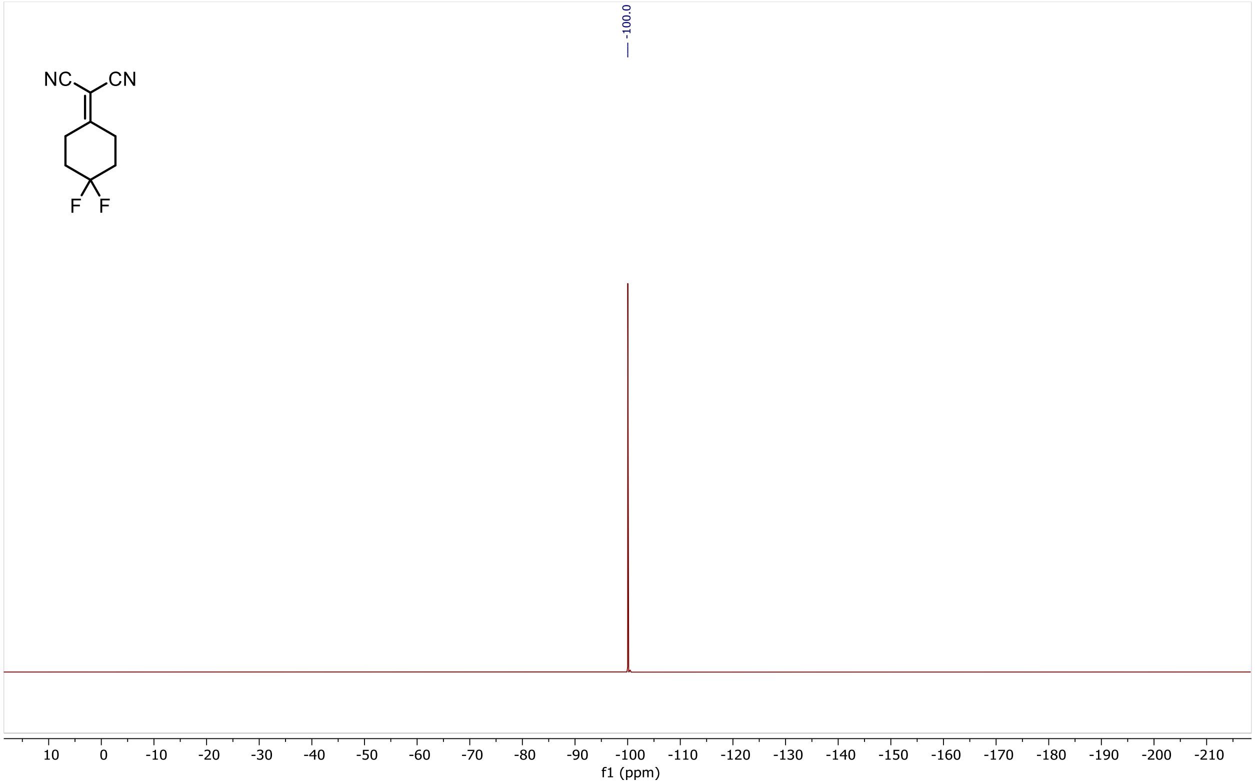

^{1}H NMR (600 MHz, $CDCl_3$) of 1g

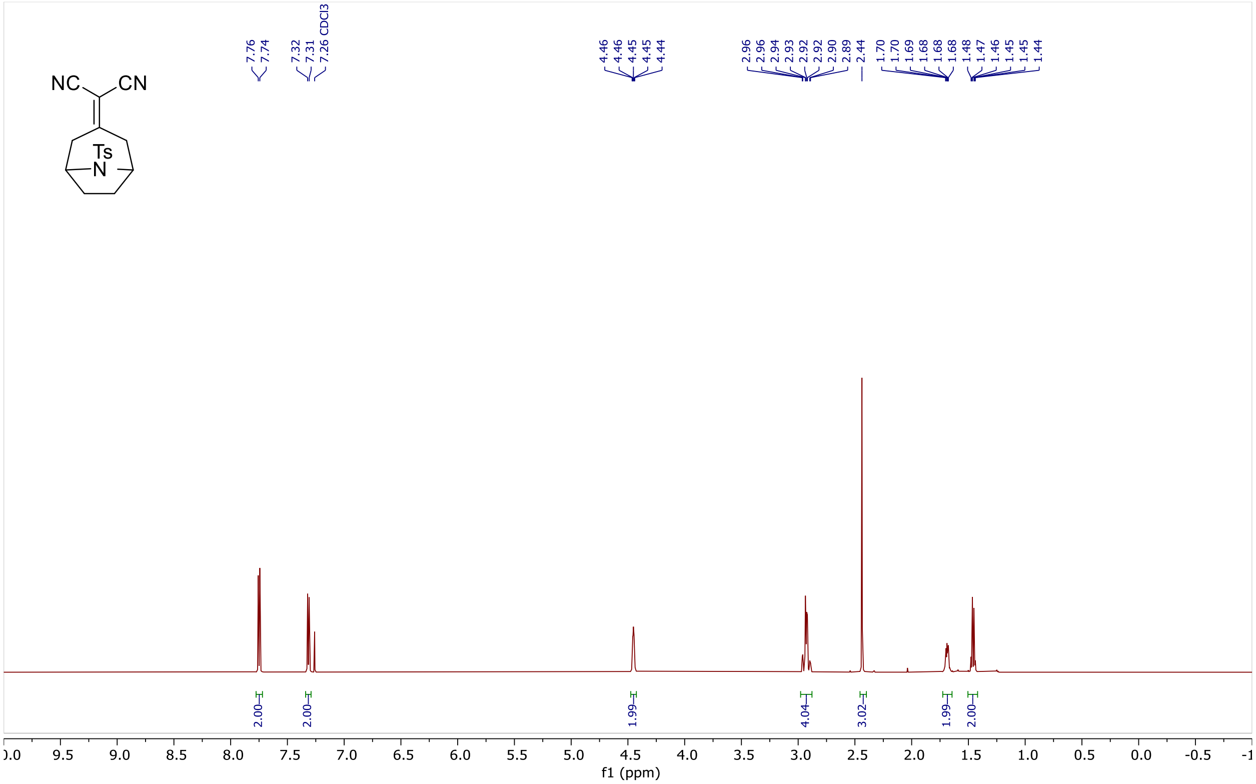

^{13}C NMR (101 MHz, $CDCl_3$) of 1g

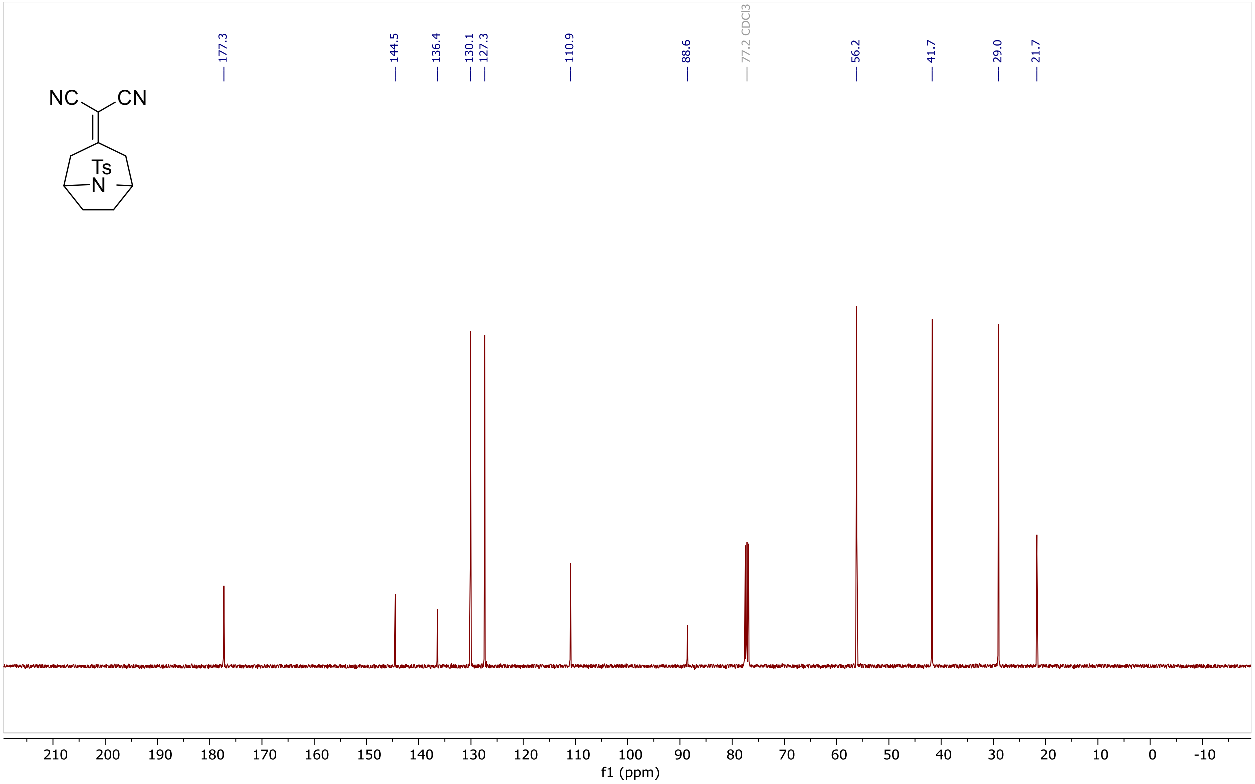

^{1}H NMR (600 MHz, $CDCl_3$) of SI-1

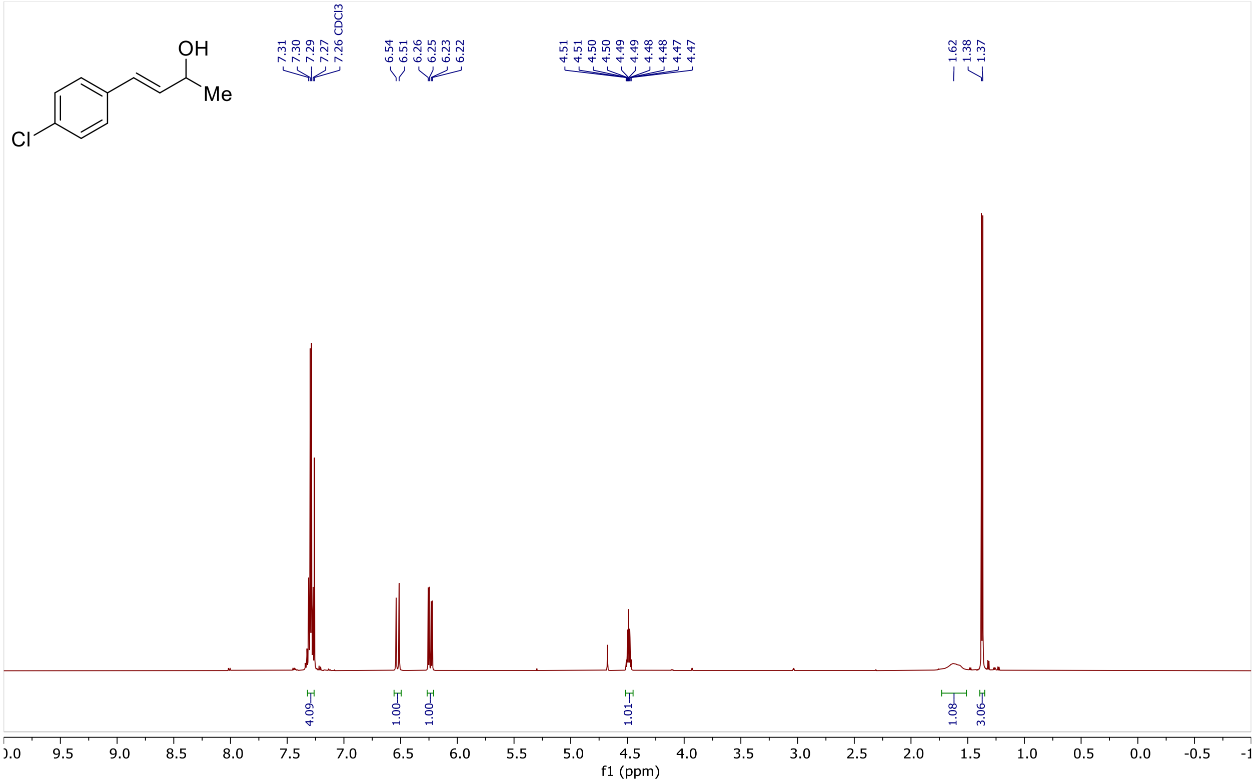

^{13}C NMR (101 MHz, $CDCl_3$) of SI-1

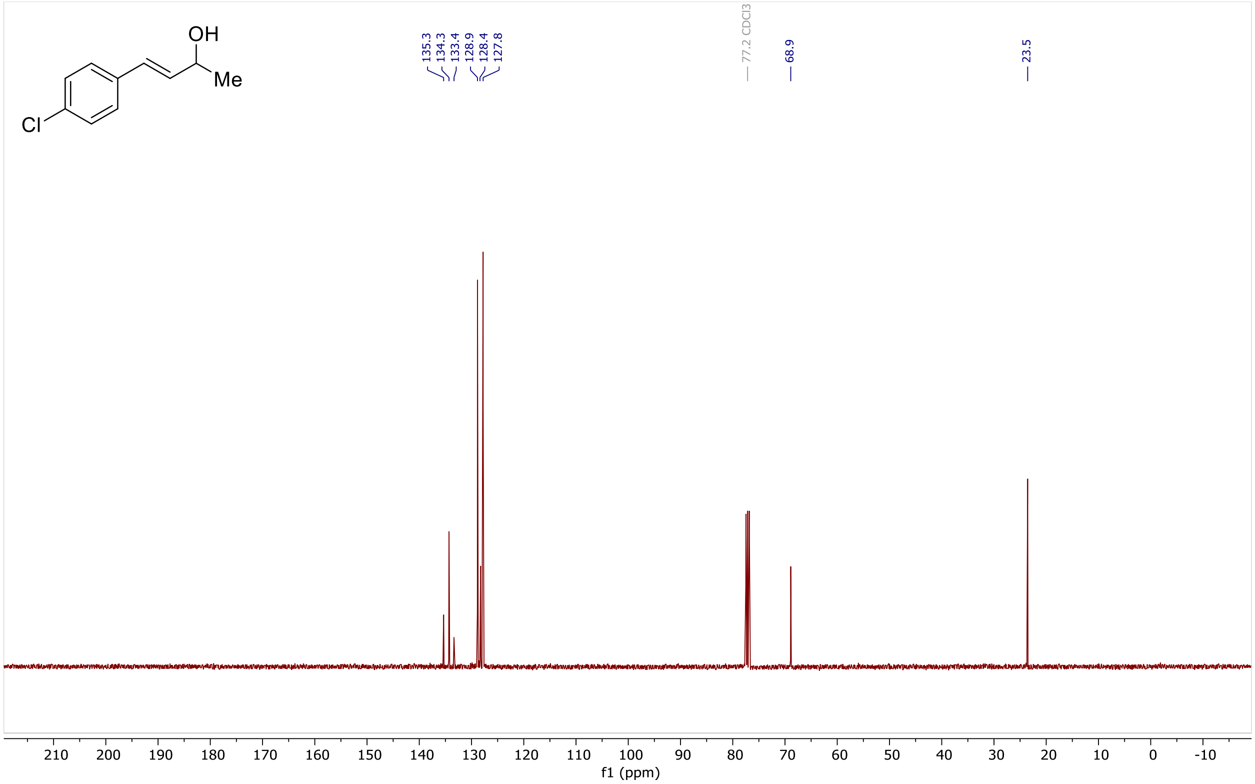

^{1}H NMR (400 MHz, $CDCl_3$) of **SI-2**

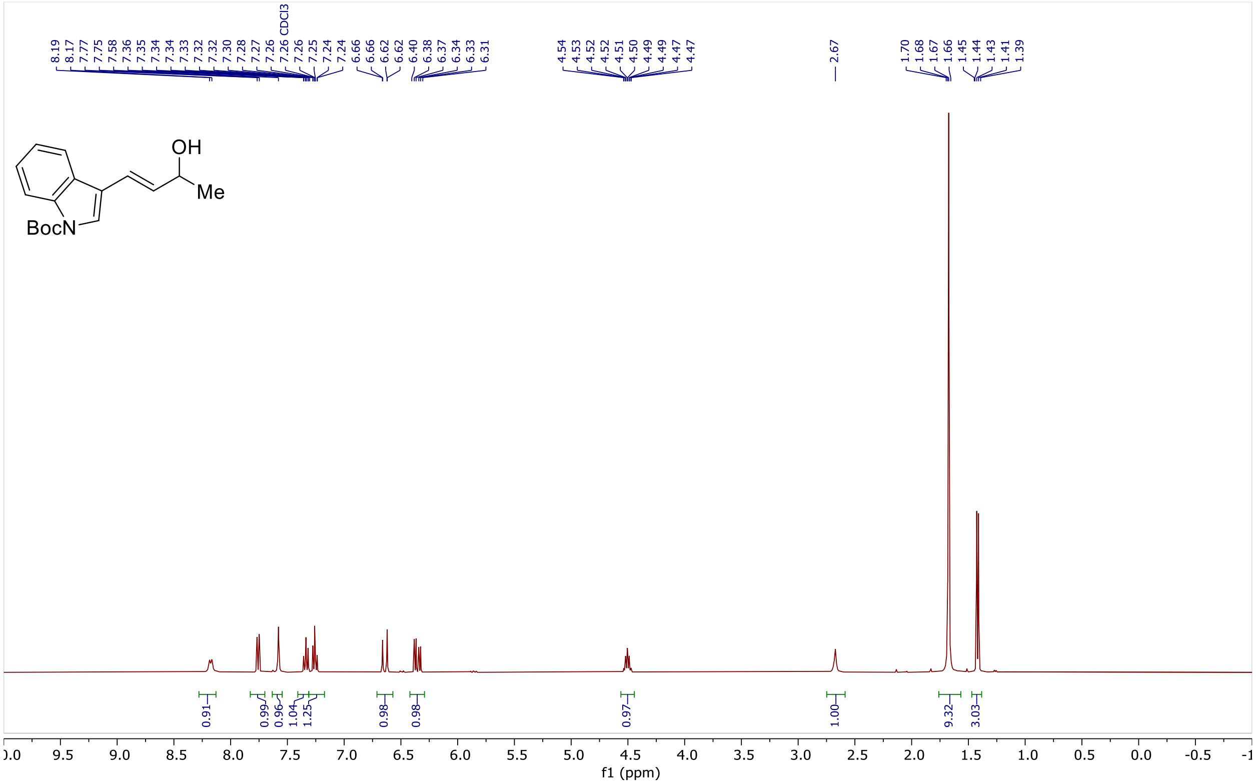

^{13}C NMR (101 MHz, $CDCl_3$) SI-2

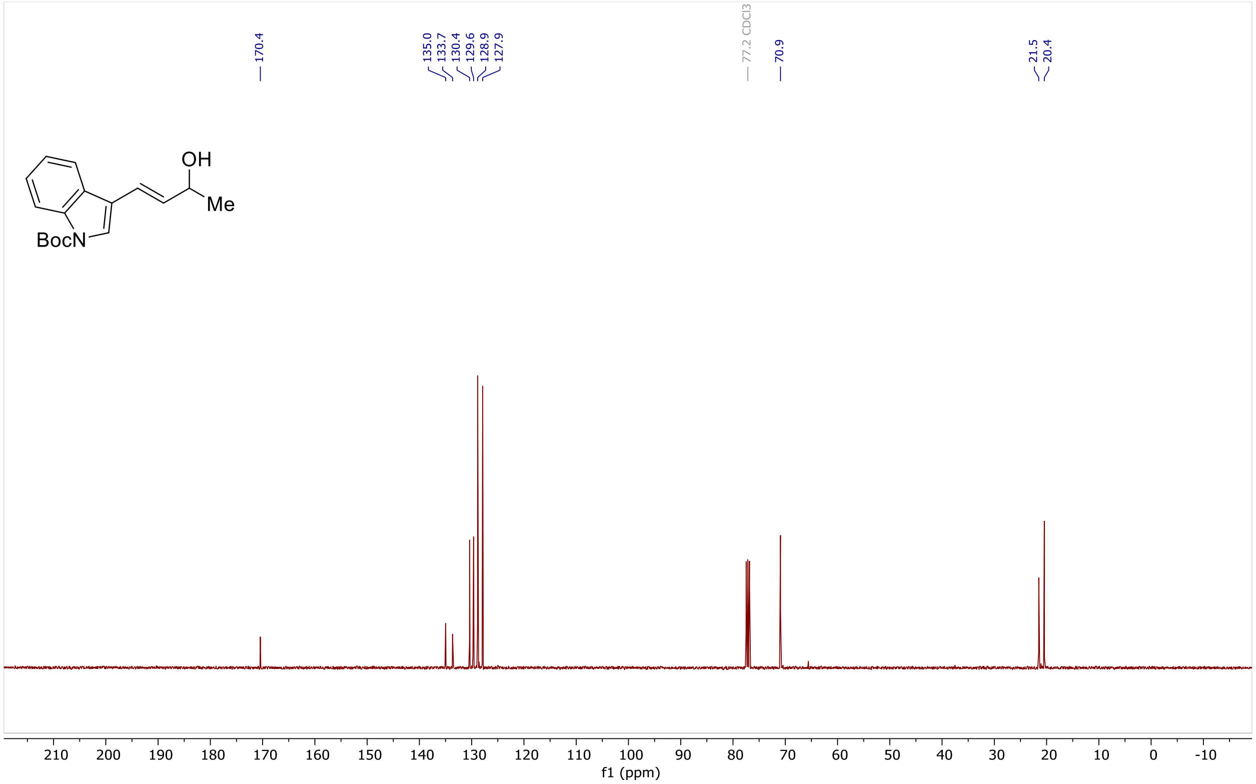

^{1}H NMR (600 MHz, $CDCl_3$) of *rac*-2a

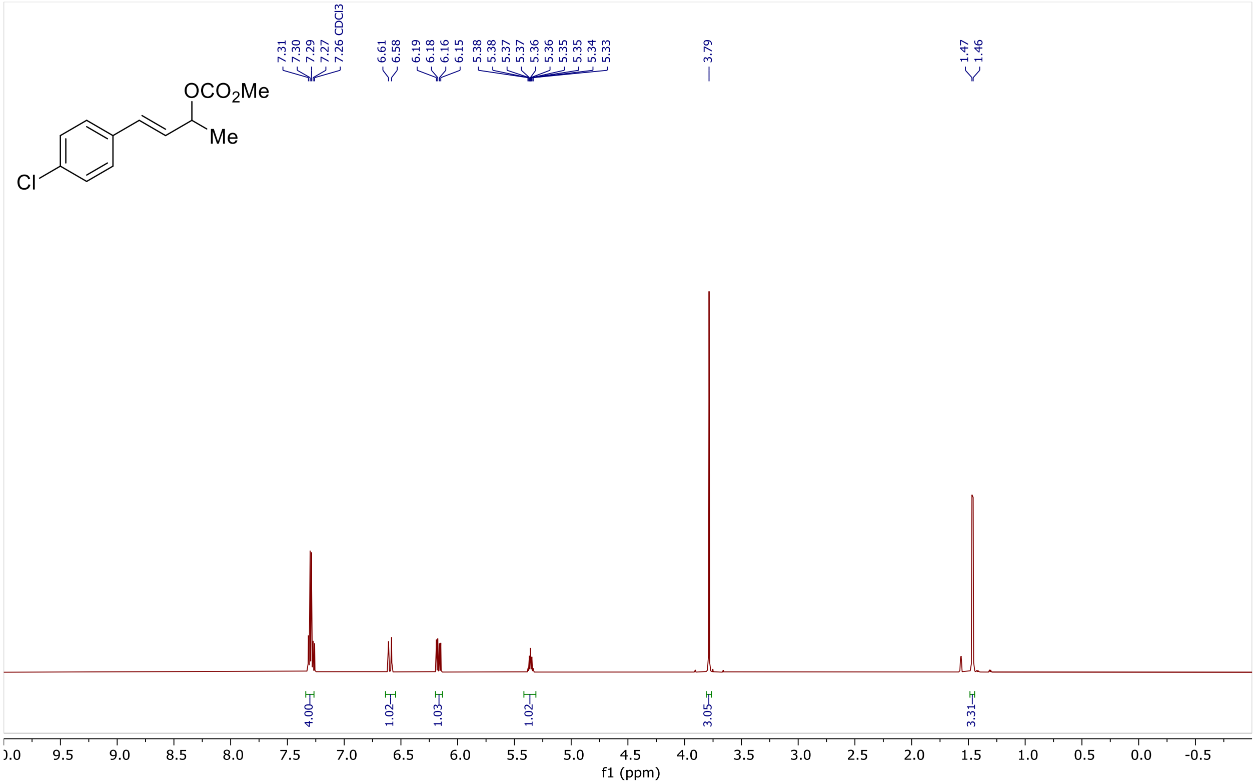

^{13}C NMR (101 MHz, $CDCl_3$) of *rac*-2a

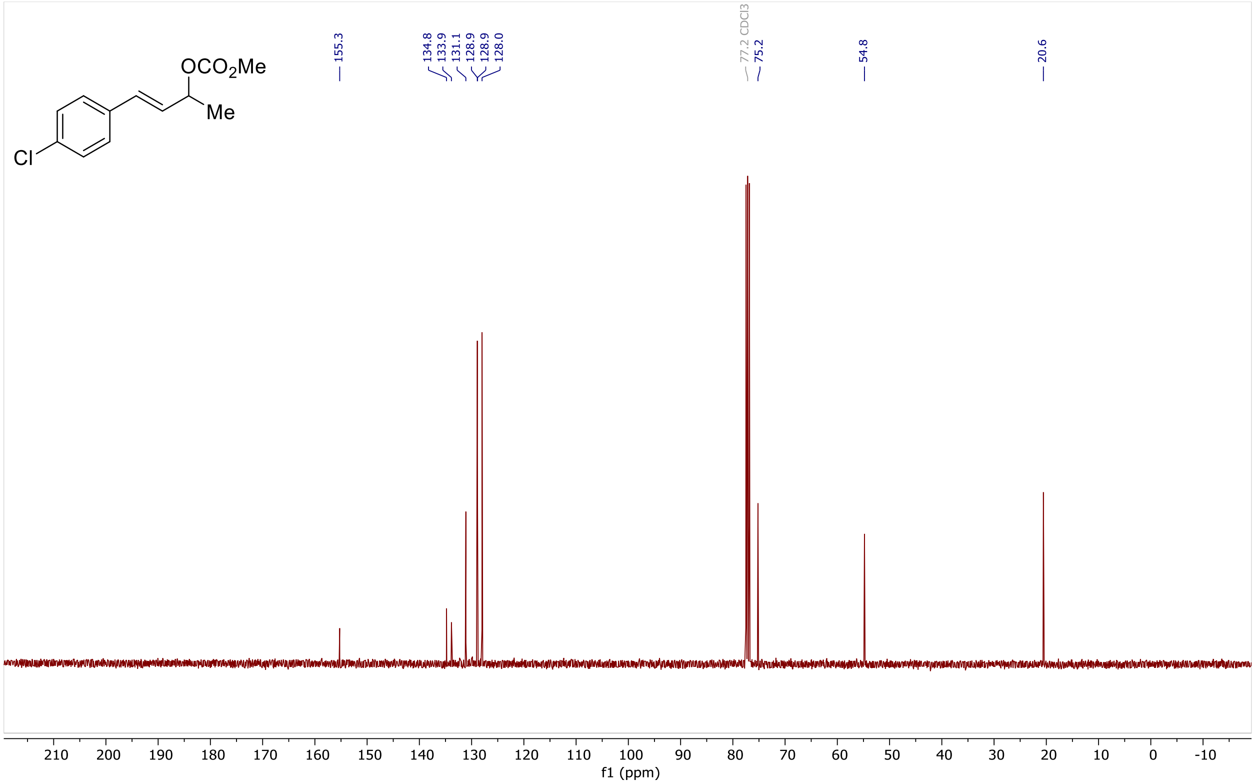

^{1}H NMR (400 MHz, CDCl$_3$) of *rac*-2a-Boc

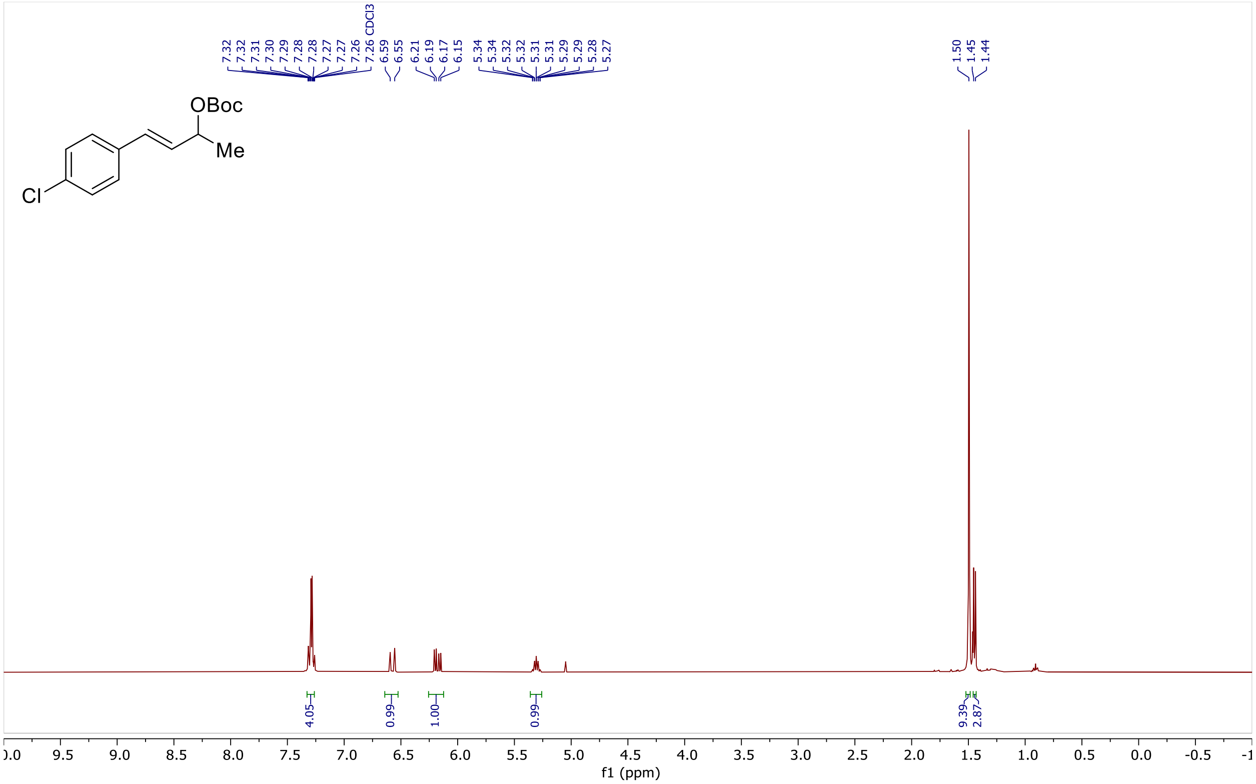

^{13}C NMR (101 MHz, CDCl$_3$) of *rac*-2a-Boc

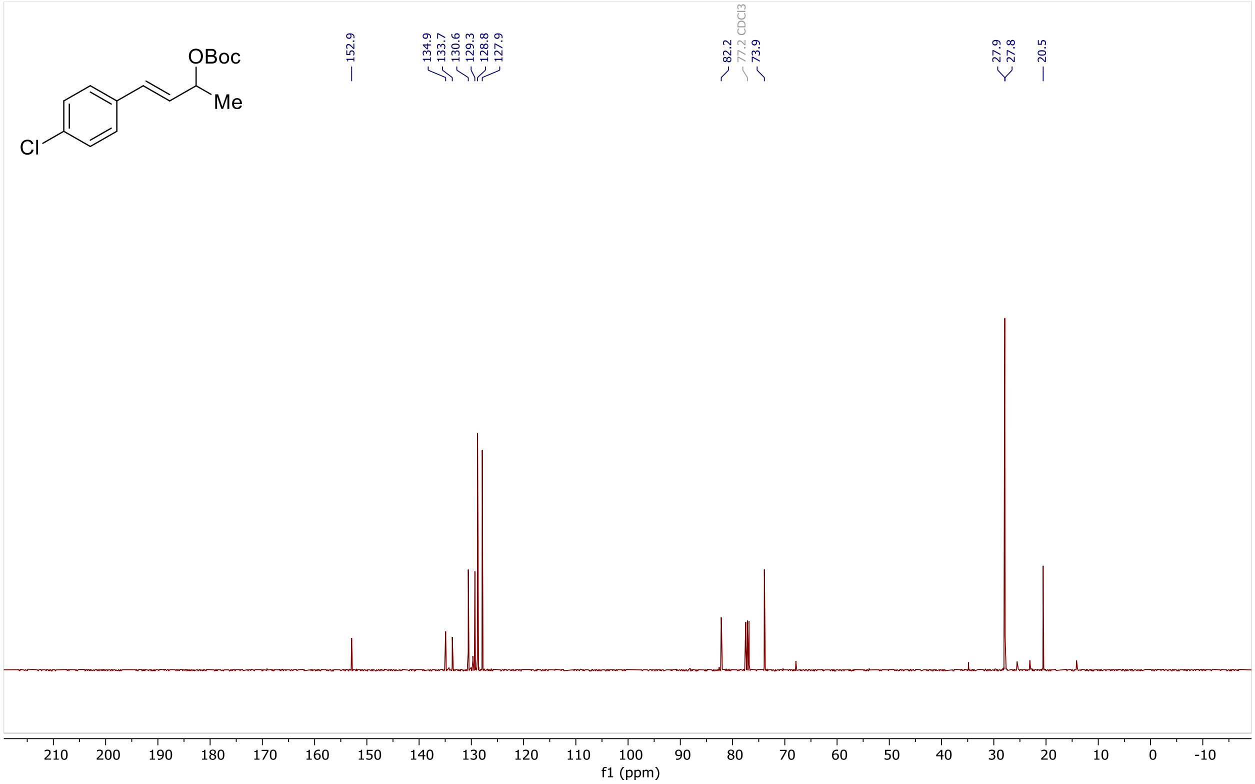

^{1}H NMR (400 MHz, CDCl$_3$) of *rac*-2a-Ac

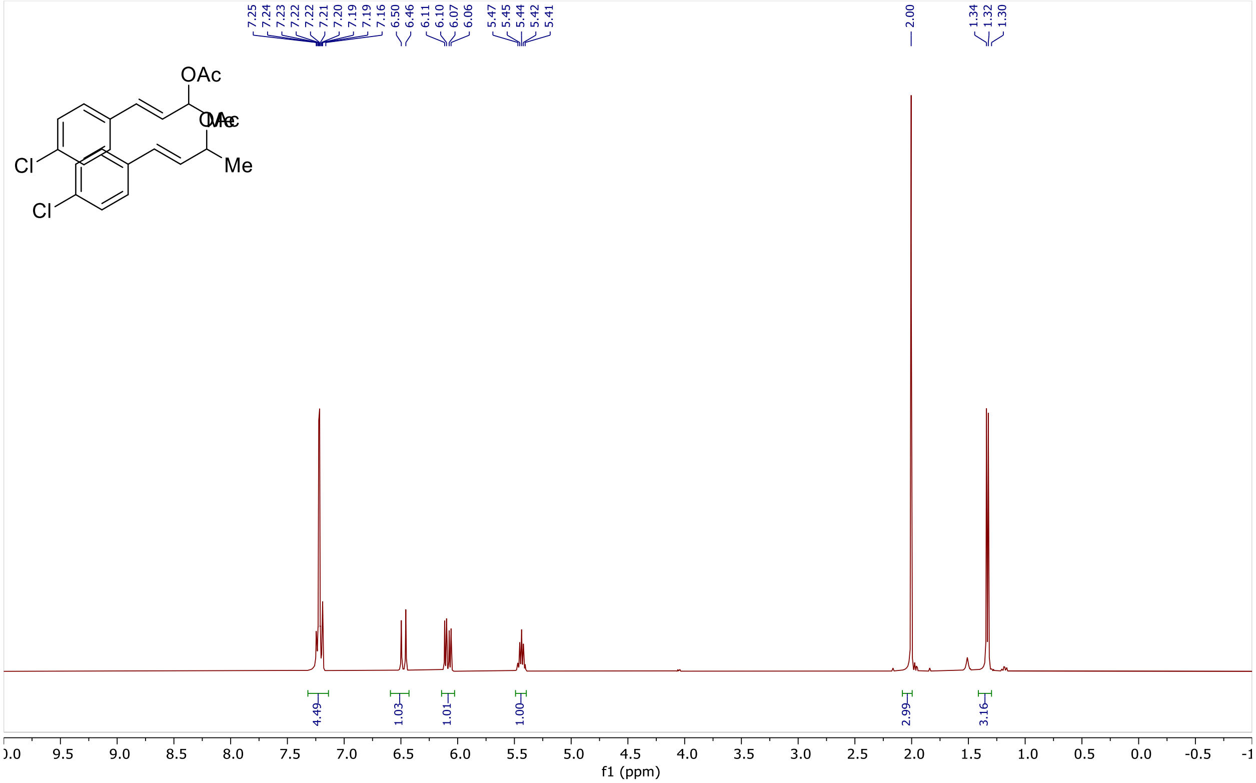

^{13}C NMR (101 MHz, CDCl$_3$) of *rac*-2a-Ac

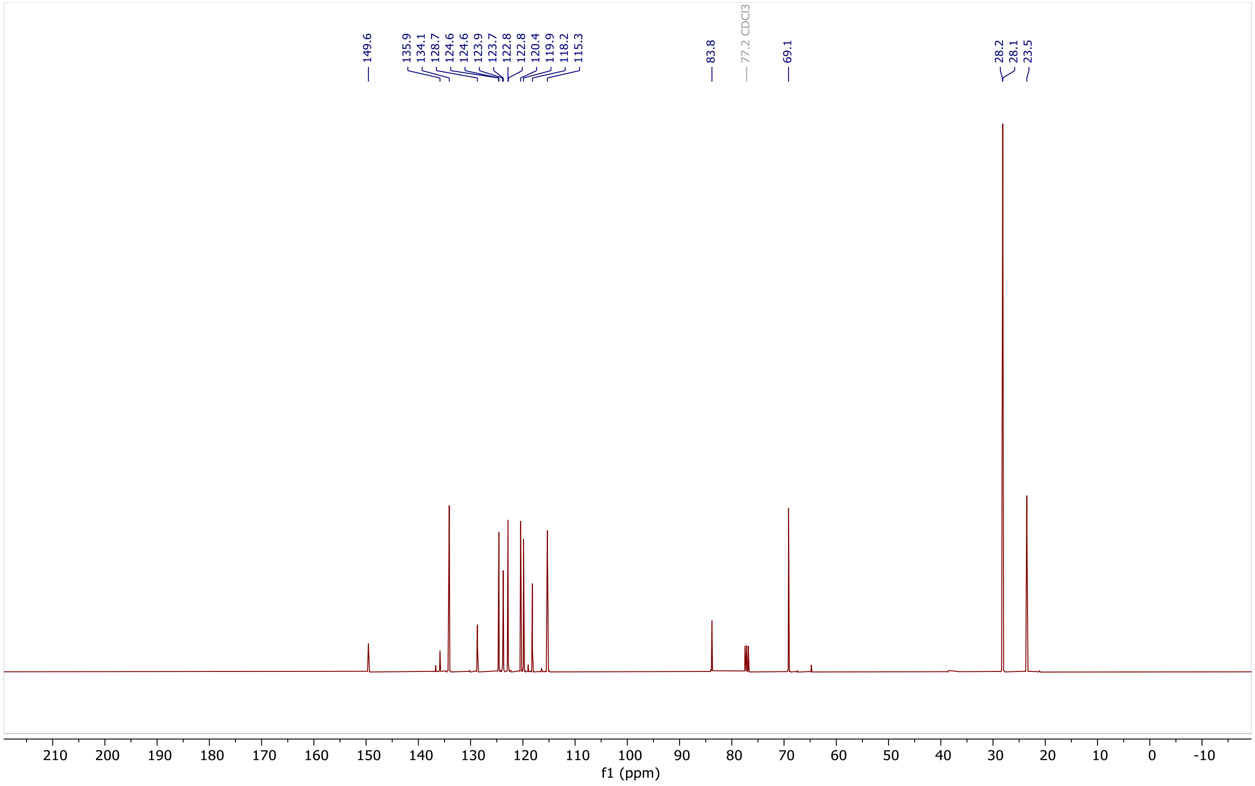

^{1}H NMR (400 MHz, CDCl₃) of *rac*-2b

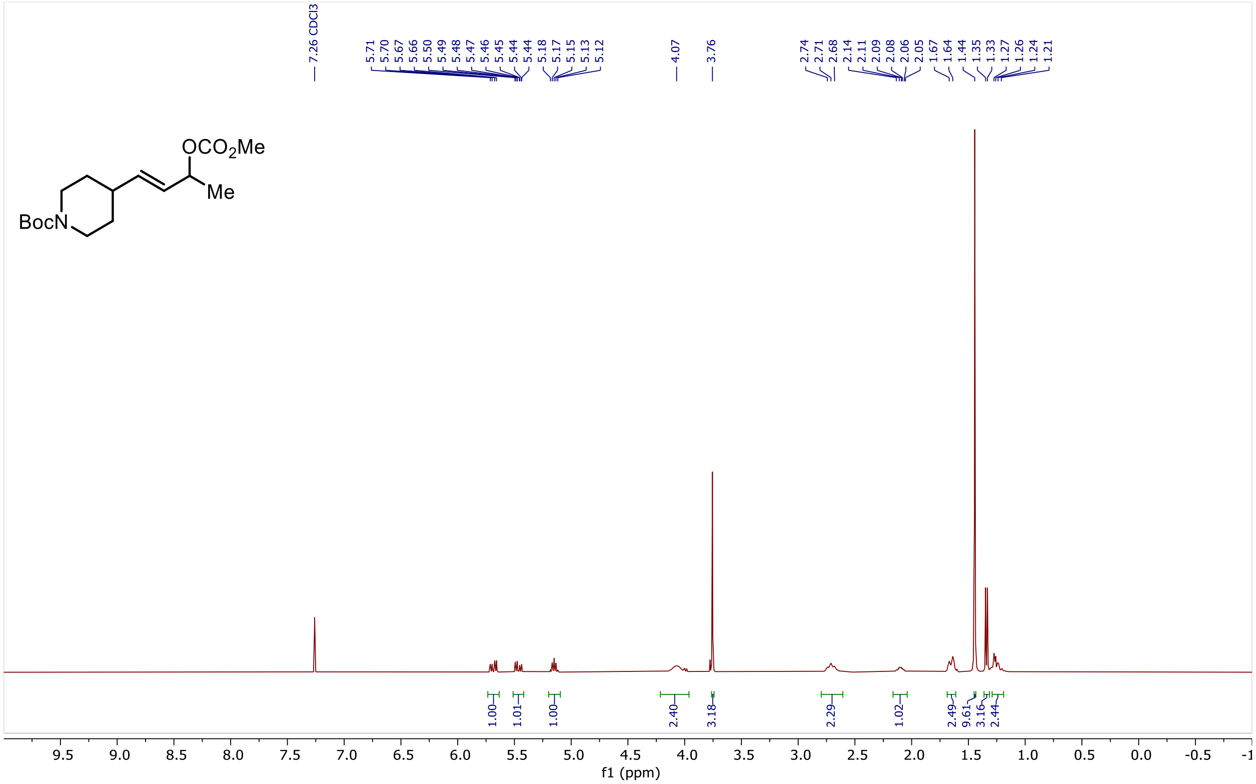

^{13}C NMR (101 MHz, CDCl₃) of *rac*-2b

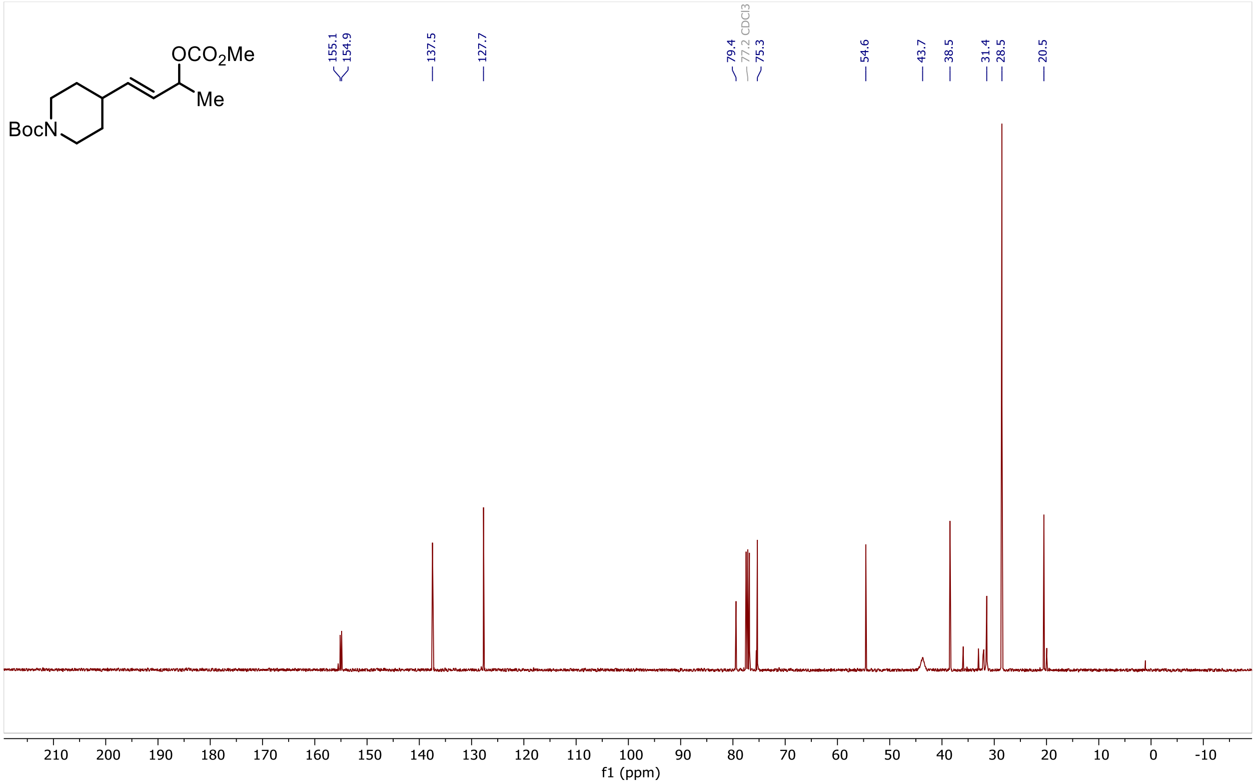

^{1}H NMR (600 MHz, CDCl$_3$) of ***rac*-2c**

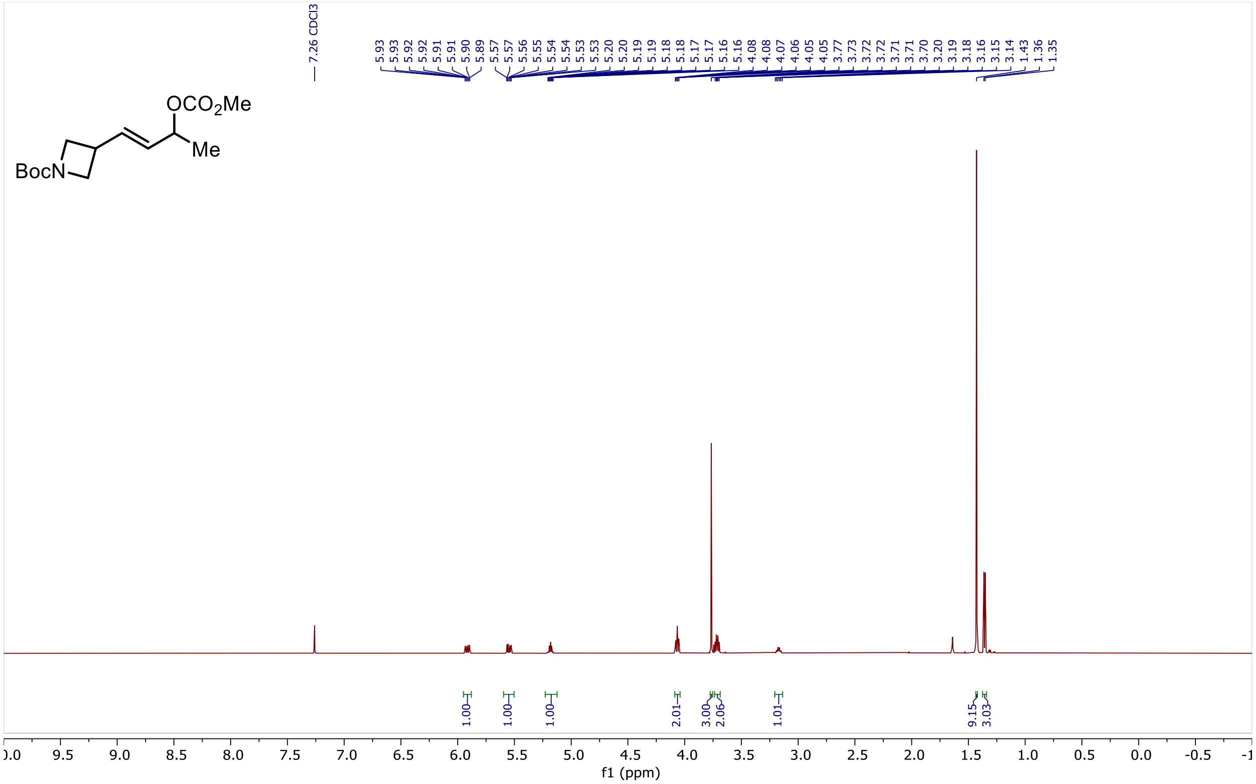

^{13}C NMR (101 MHz, CDCl$_3$) of ***rac*-2c**

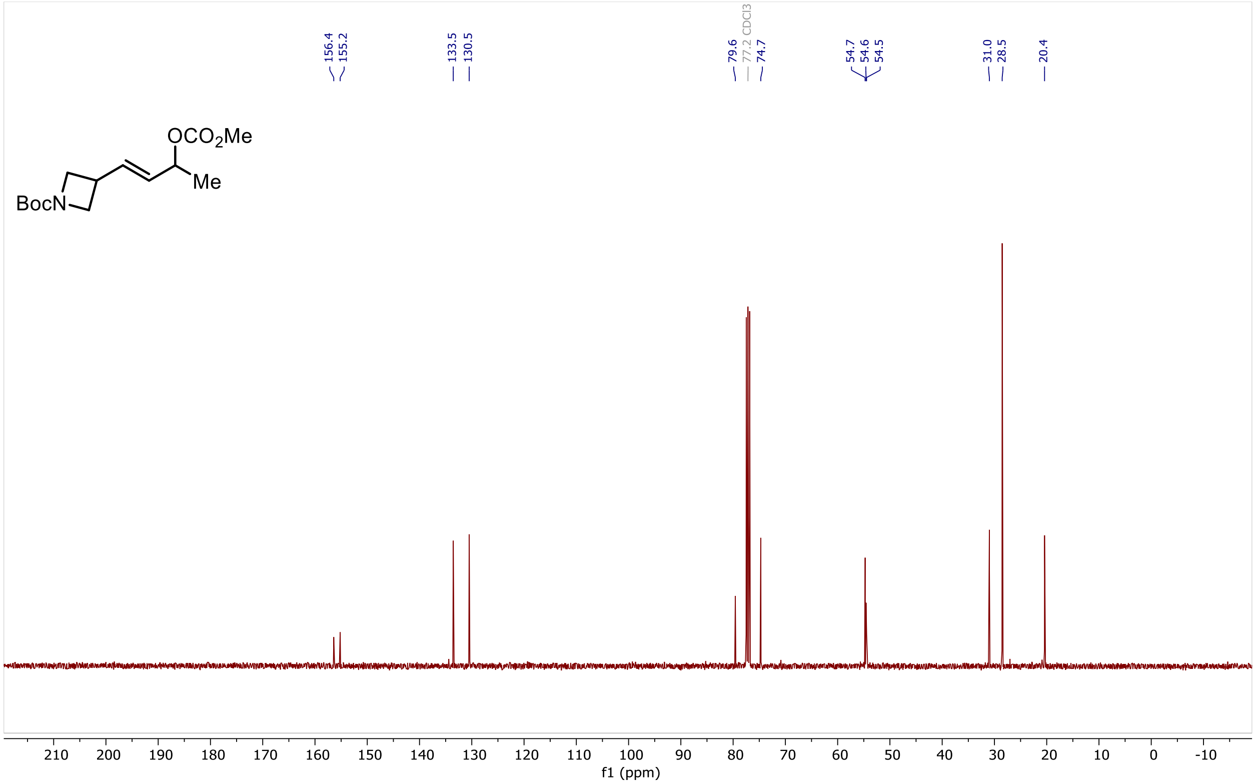

^{1}H NMR (400 MHz, CDCl$_3$) *rac*-2d

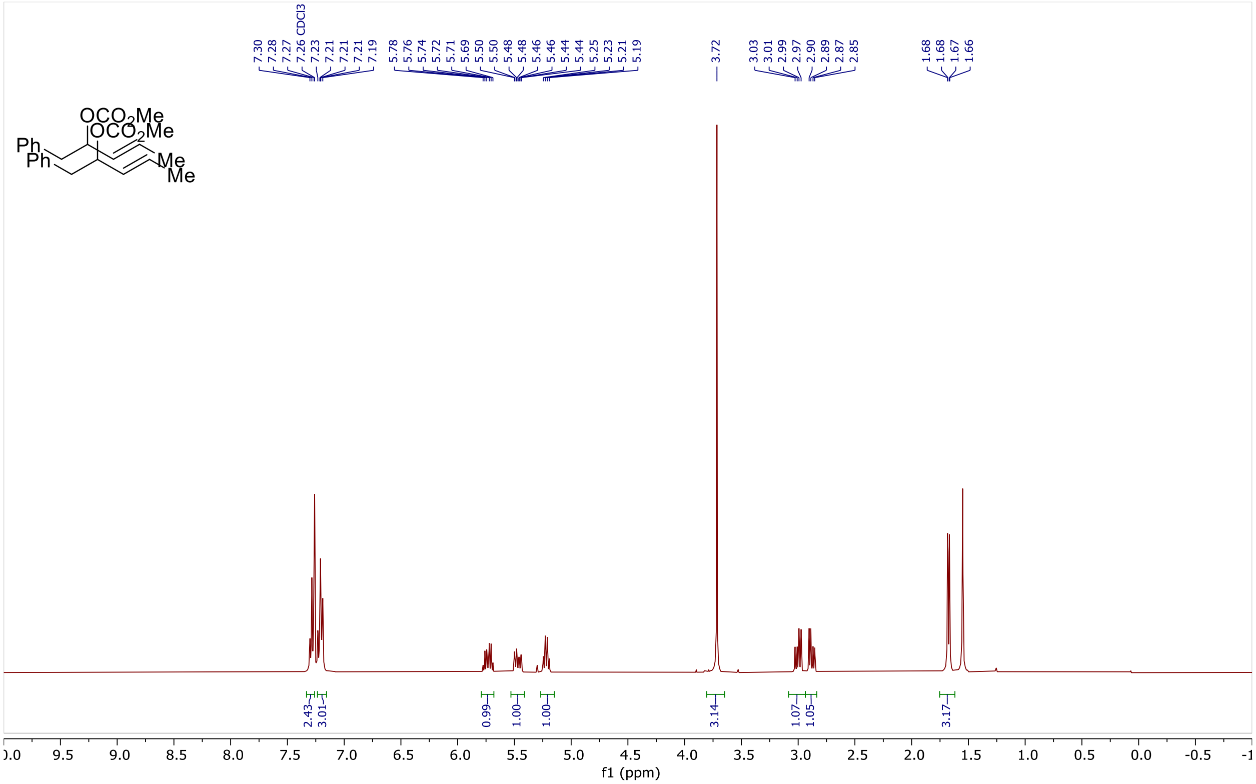

^{13}C NMR (101 MHz, CDCl$_3$) *rac*-2d

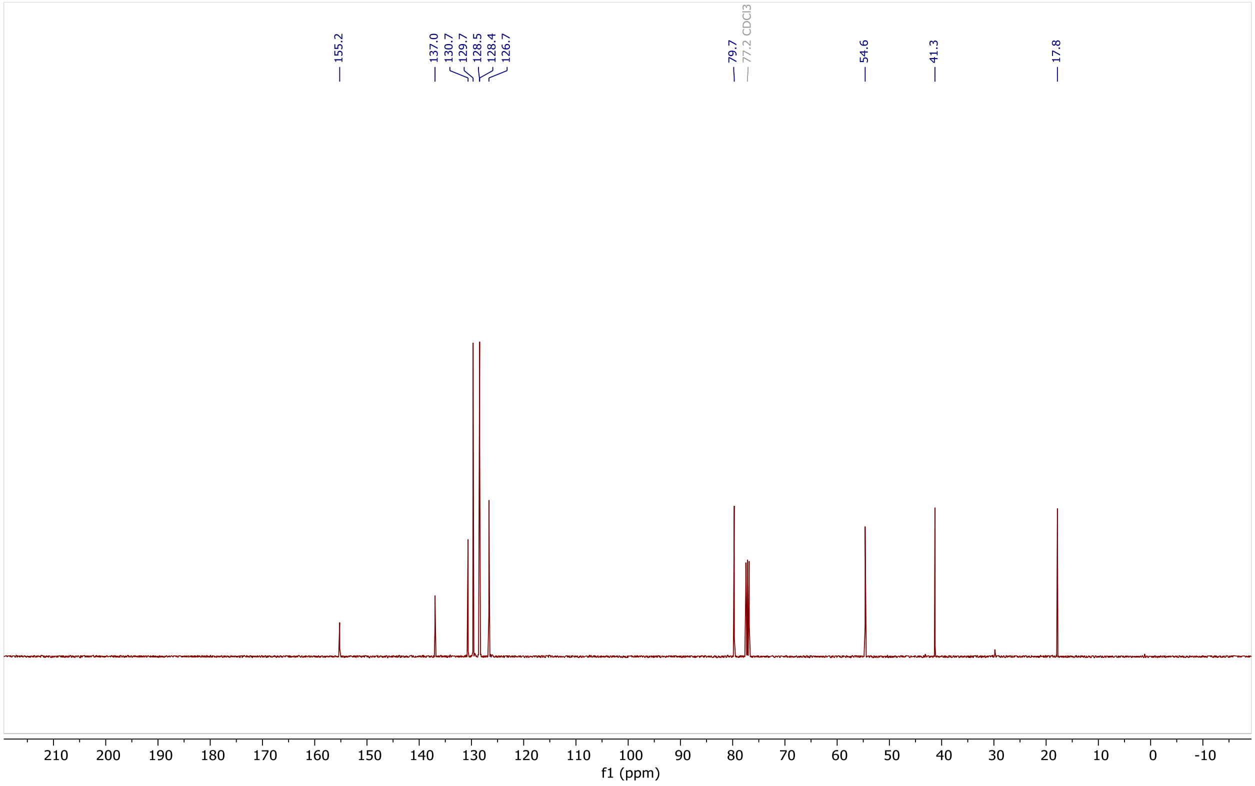

^{1}H NMR (600 MHz, CDCl$_3$) of ***rac*-2e**

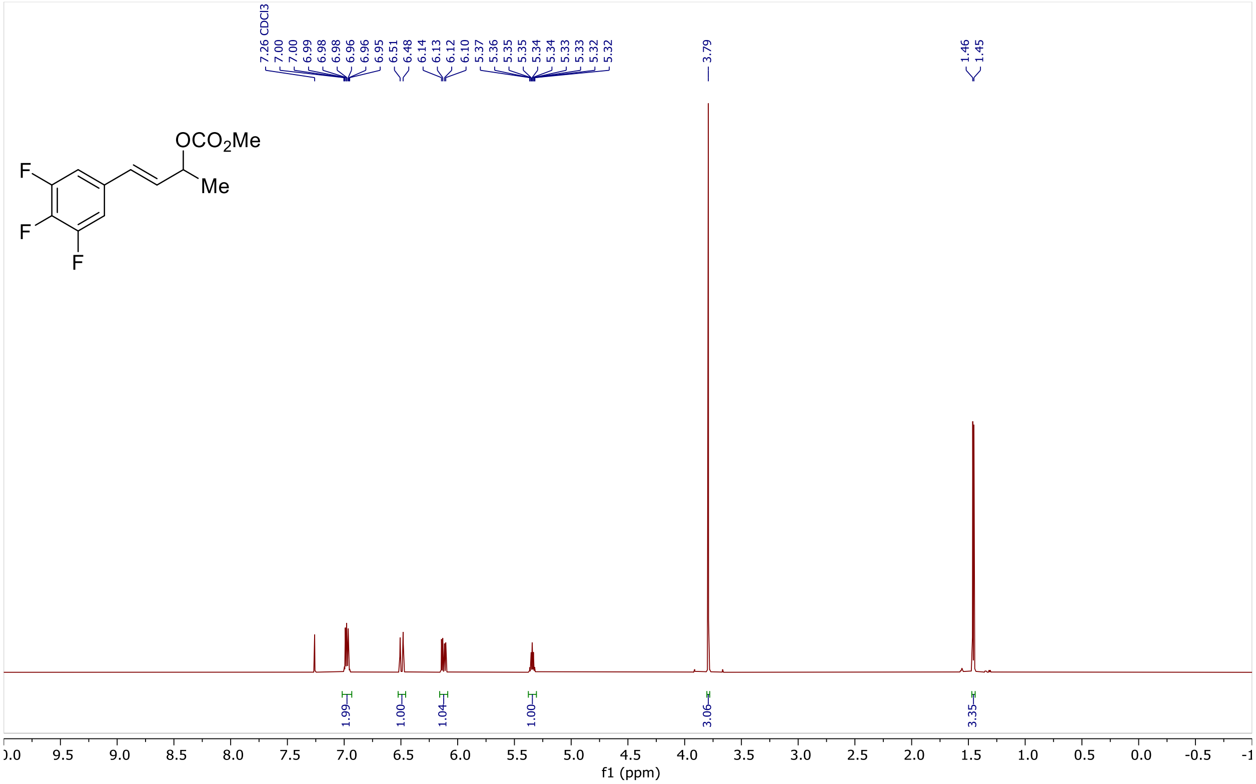

^{13}C NMR (101 MHz, CDCl$_3$) of ***rac*-2e**

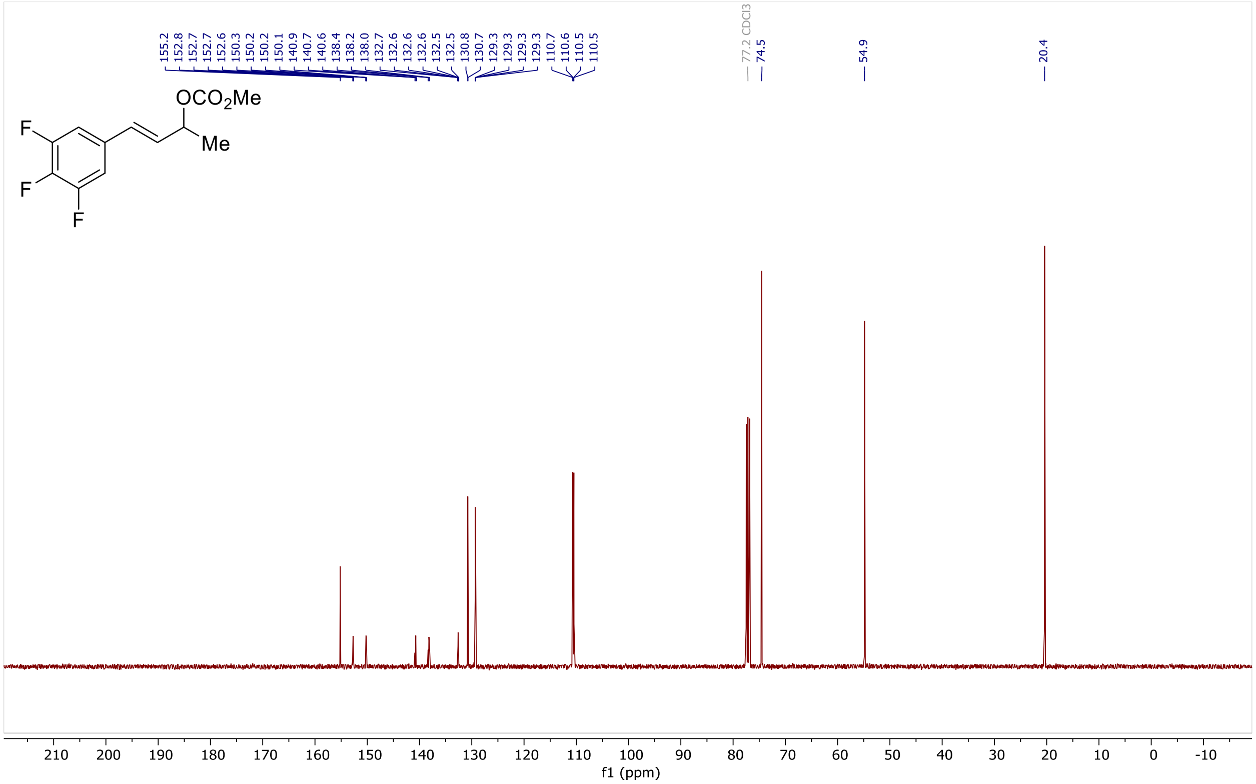

^{19}F NMR (377 MHz, CDCl$_3$) of ***rac*-2e**

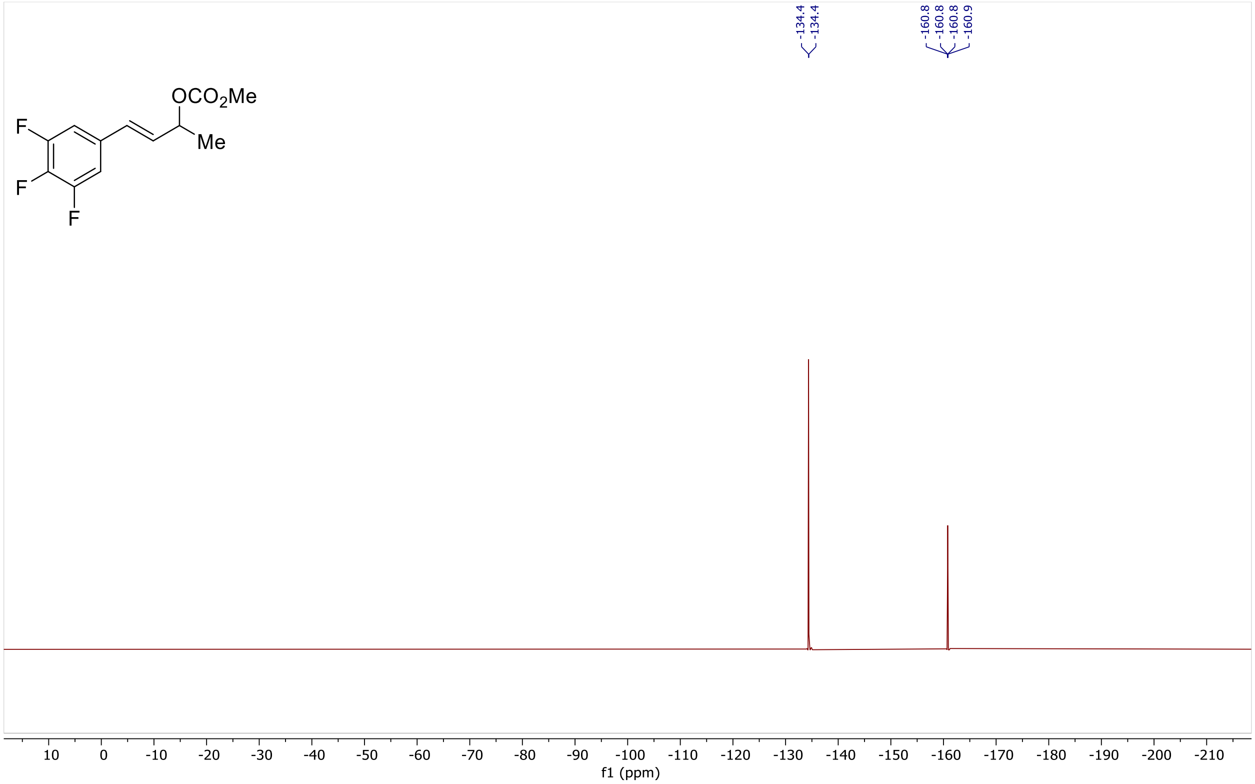

^{1}H NMR (400 MHz, CDCl$_3$) of ***rac*-2f**

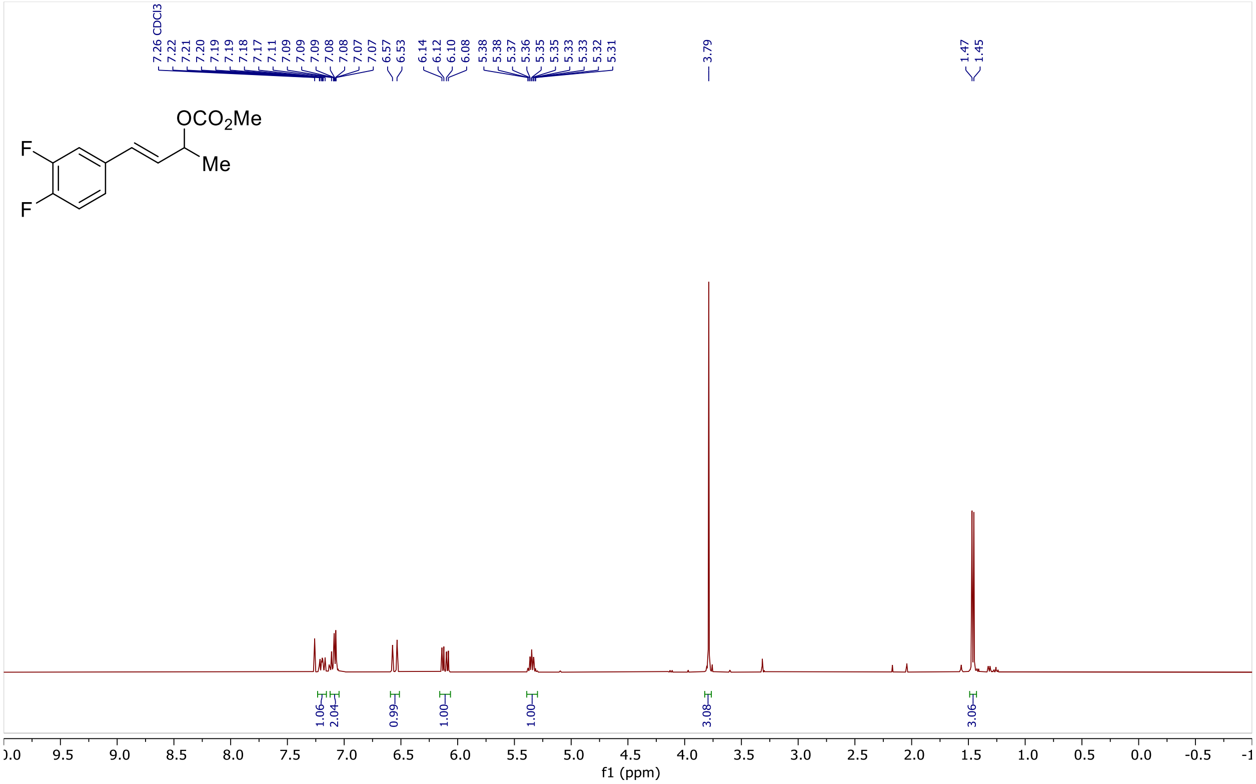

^{13}C NMR (101 MHz, $CDCl_3$) of *rac*-2f

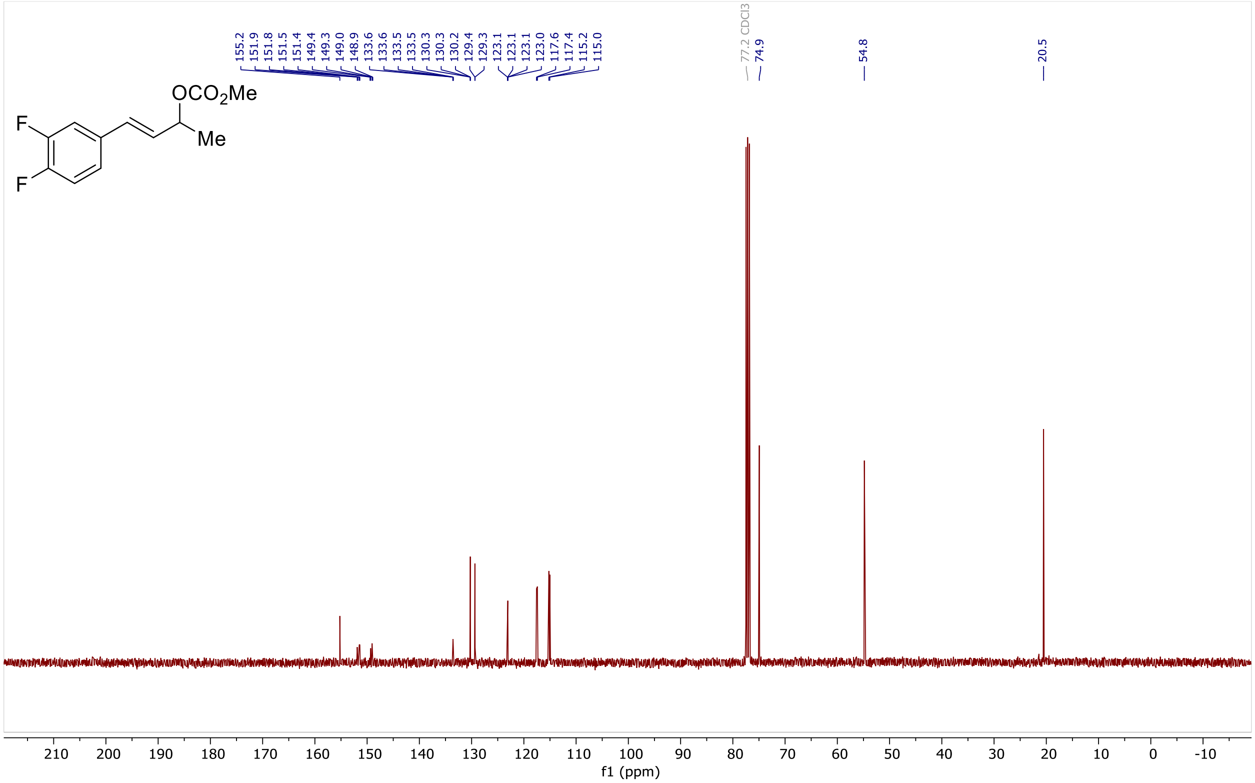

^{19}F NMR (565 MHz, $CDCl_3$) of *rac*-2f

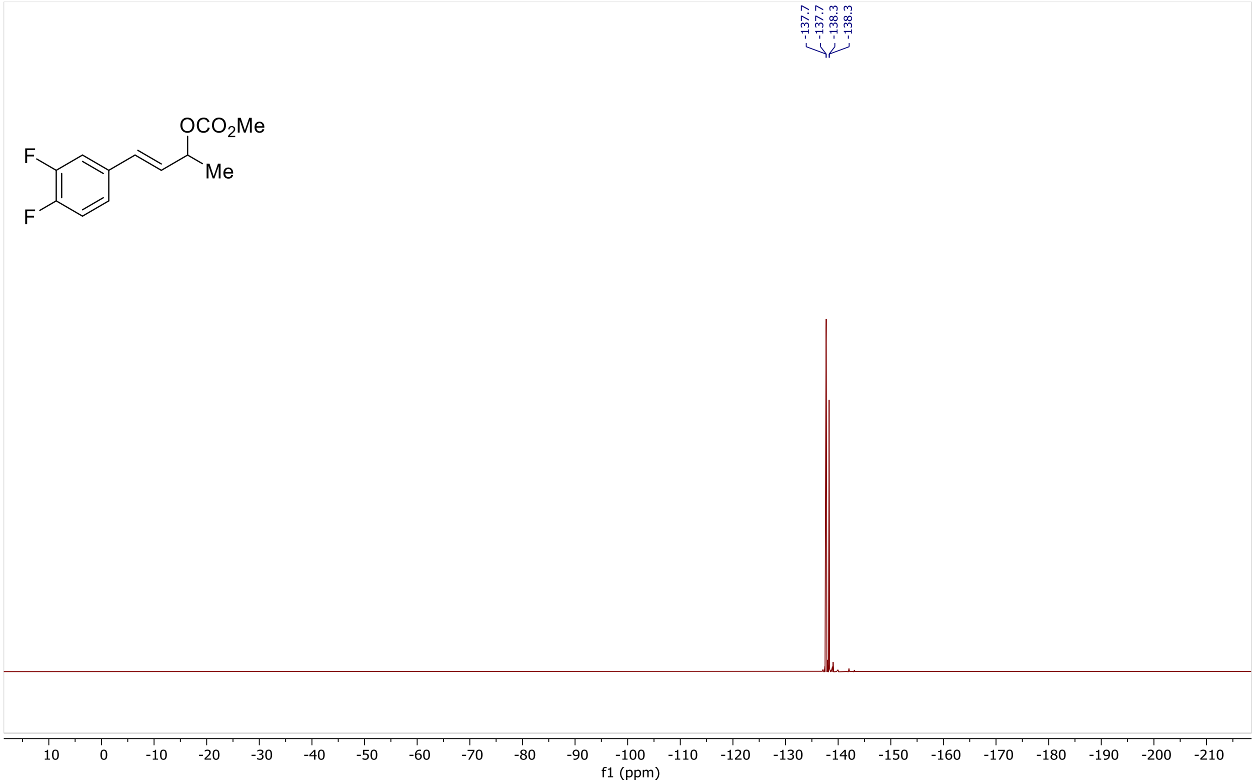

^{1}H NMR (400 MHz, $CDCl_3$) of *rac*-2g

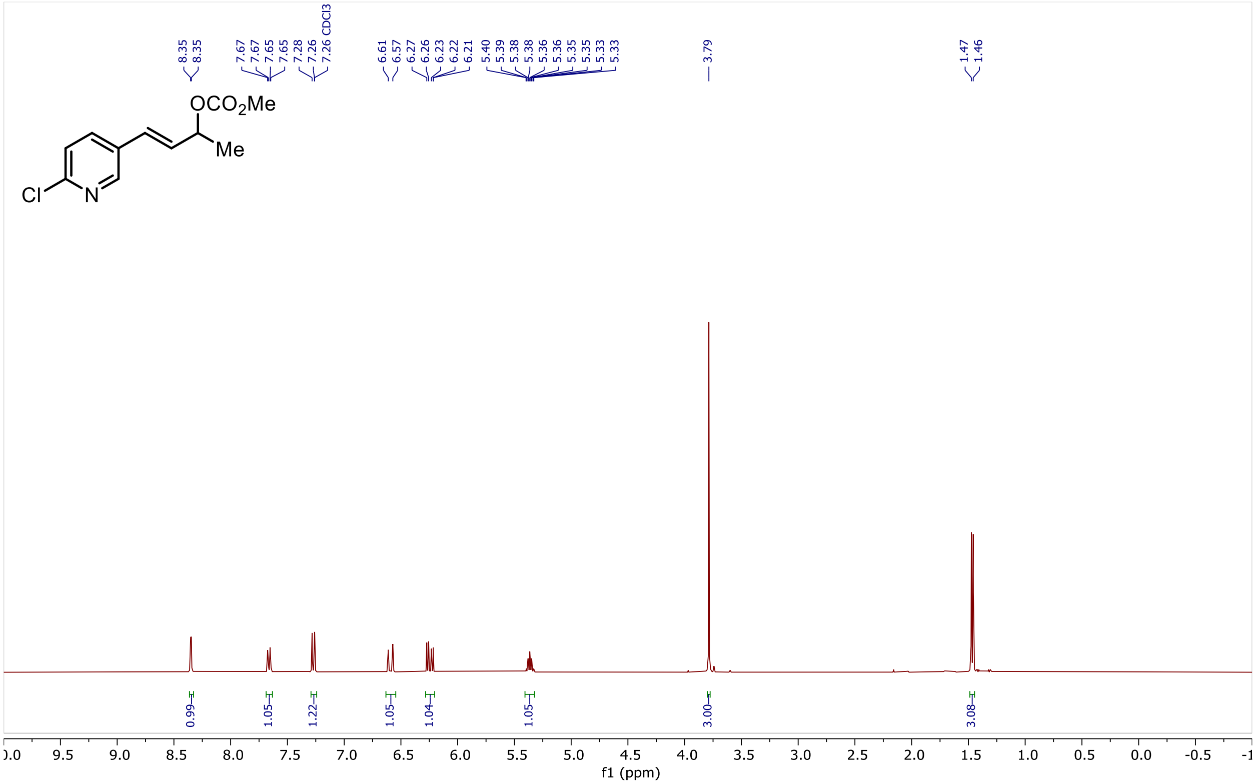

^{13}C NMR (101 MHz, $CDCl_3$) of *rac*-2g

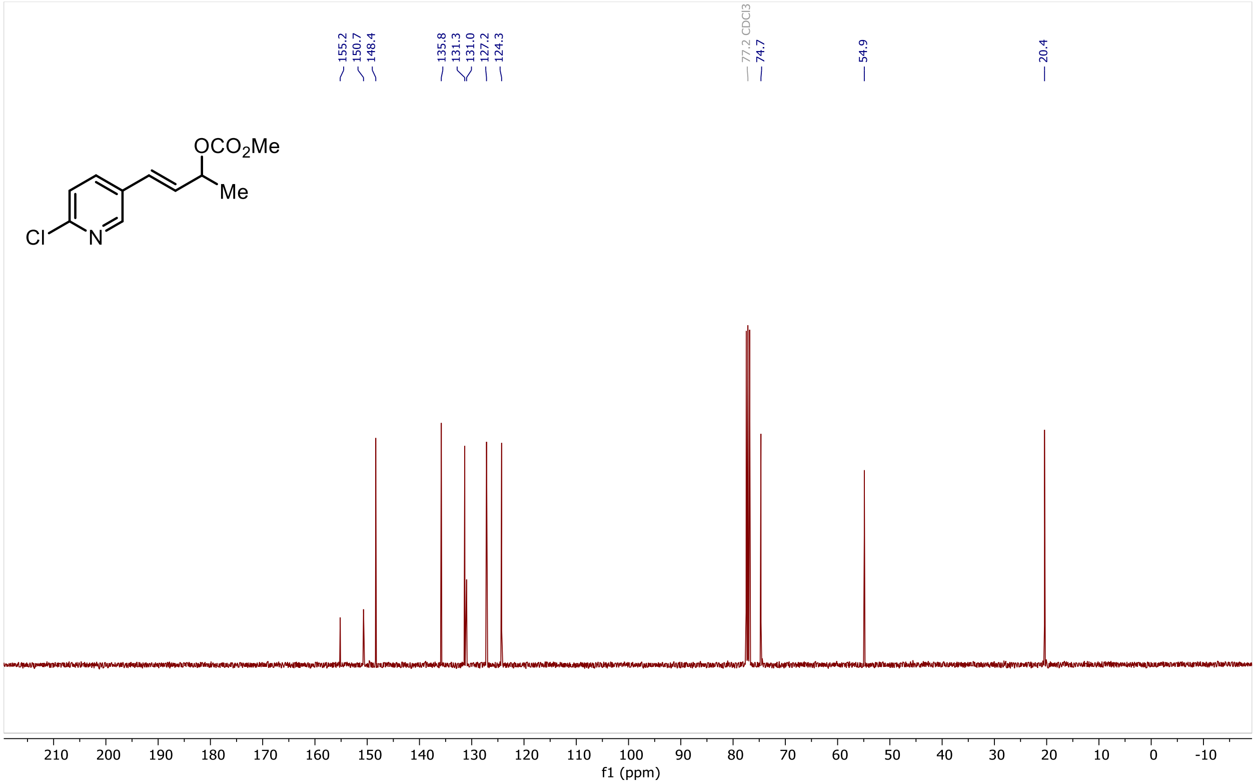

^{1}H NMR (400 MHz, $CDCl_3$) of *rac*-2h

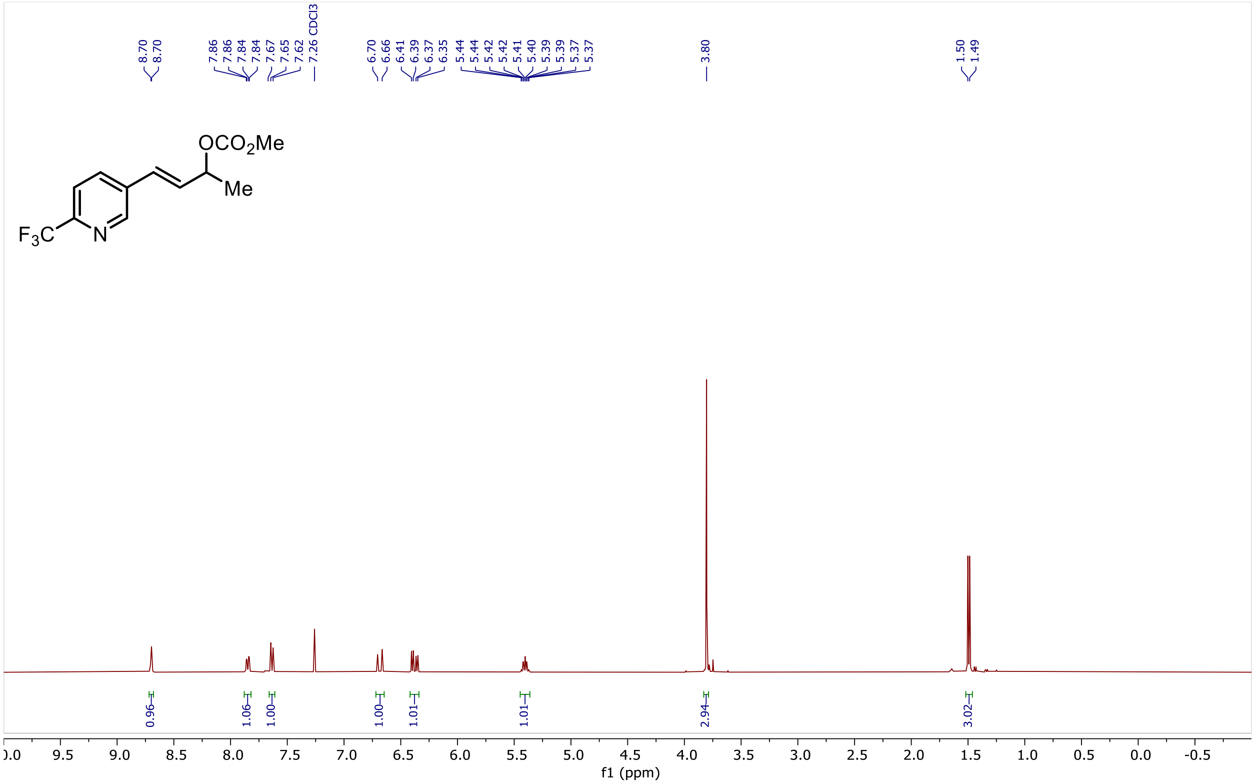

^{13}C NMR (101 MHz, $CDCl_3$) of *rac*-2h

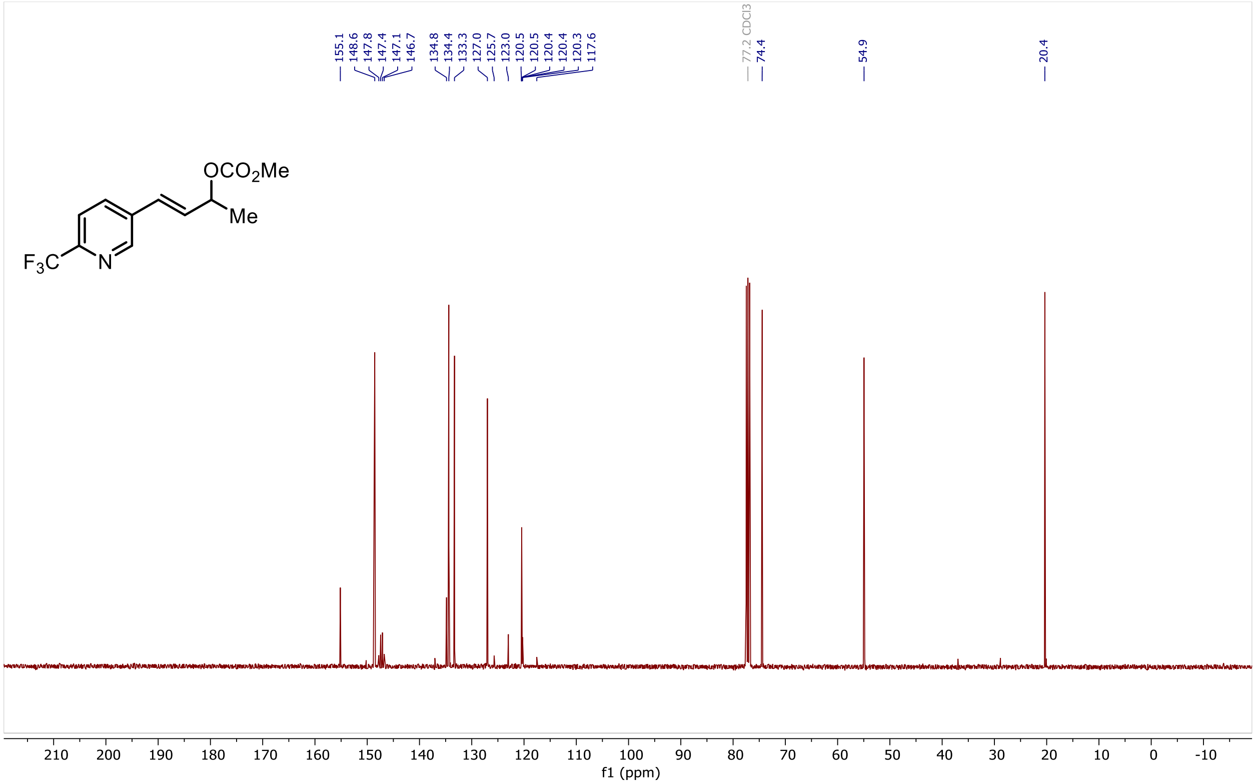

^{19}F NMR (377 MHz, CDCl₃) of *rac*-2h

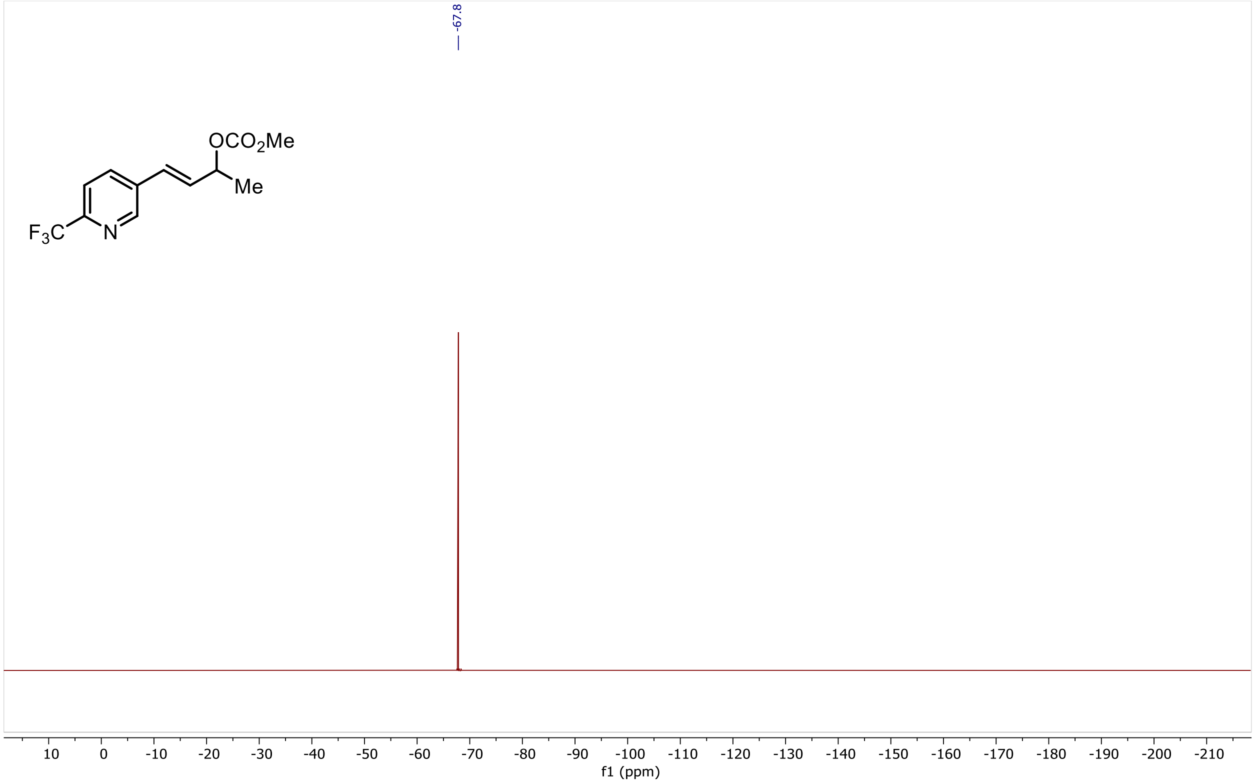

^{1}H NMR (600 MHz, CDCl₃) of *rac*-2i

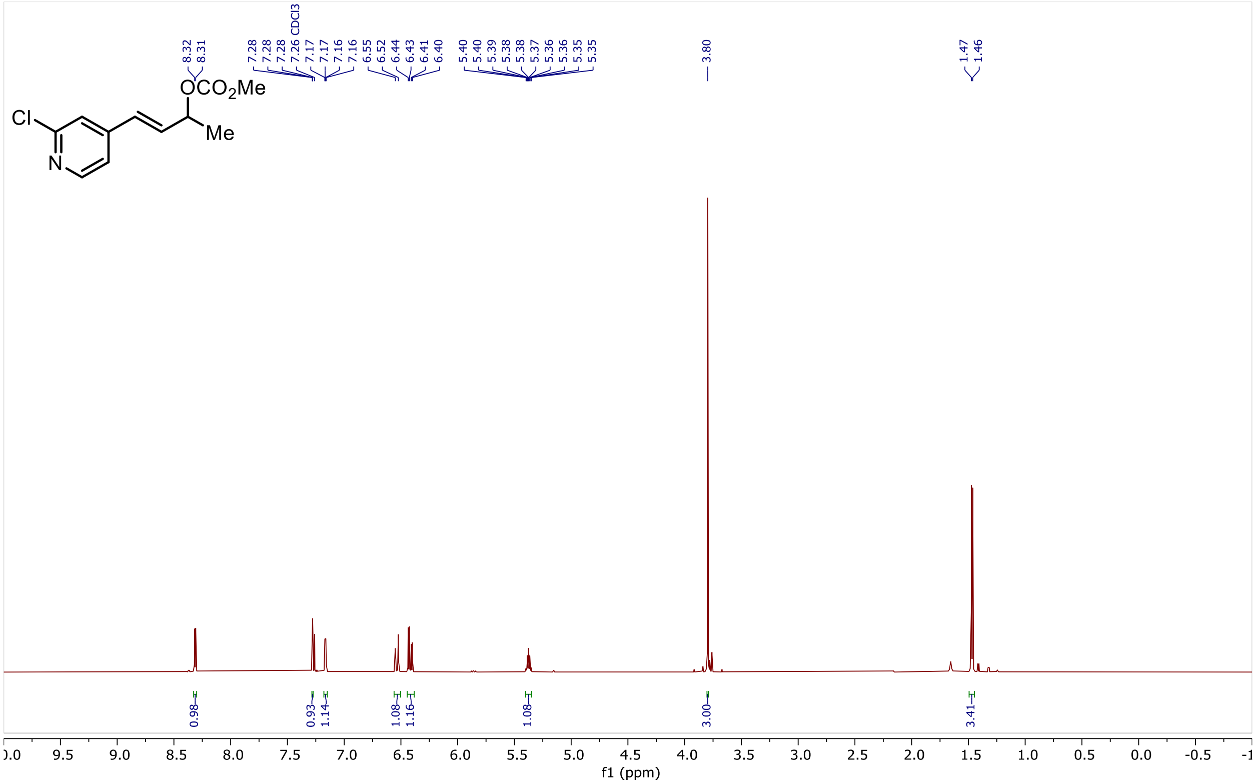

^{13}C NMR (101 MHz, $CDCl_3$) of *rac*-2i

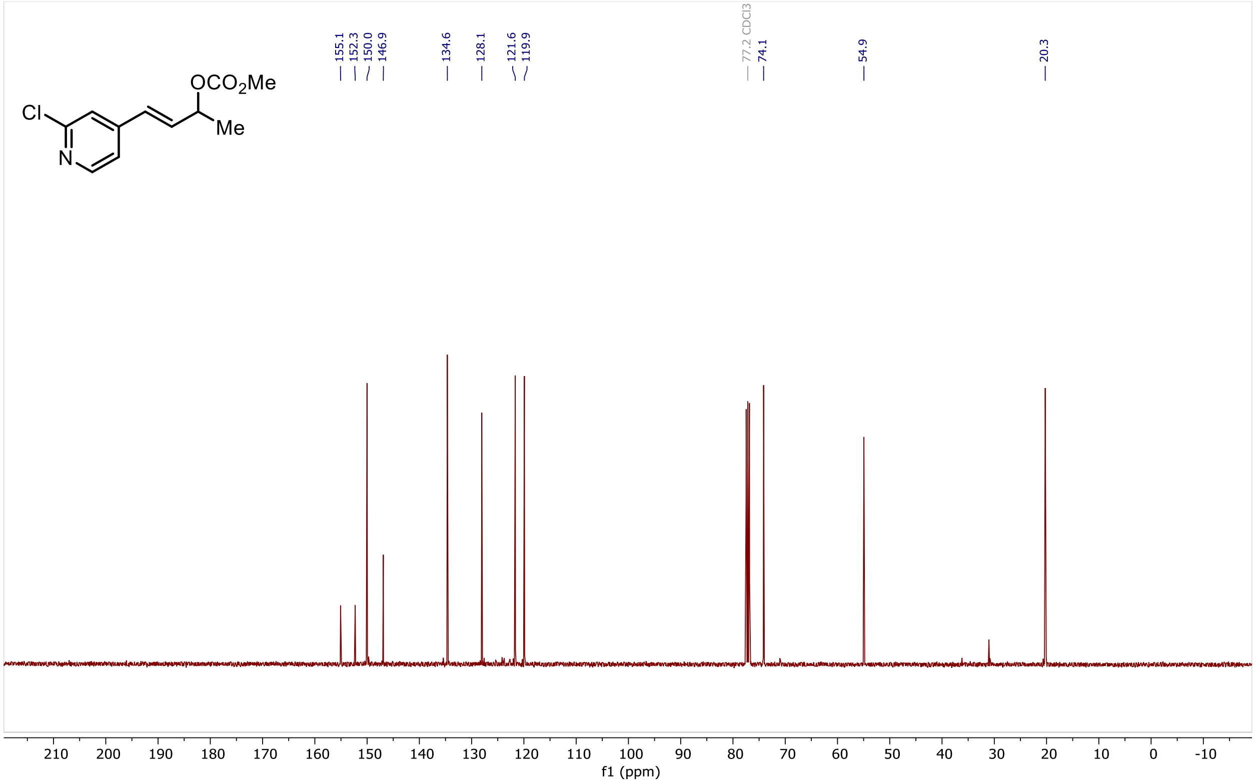

^{1}H NMR (400 MHz, $CDCl_3$) *rac*-2i-Ac

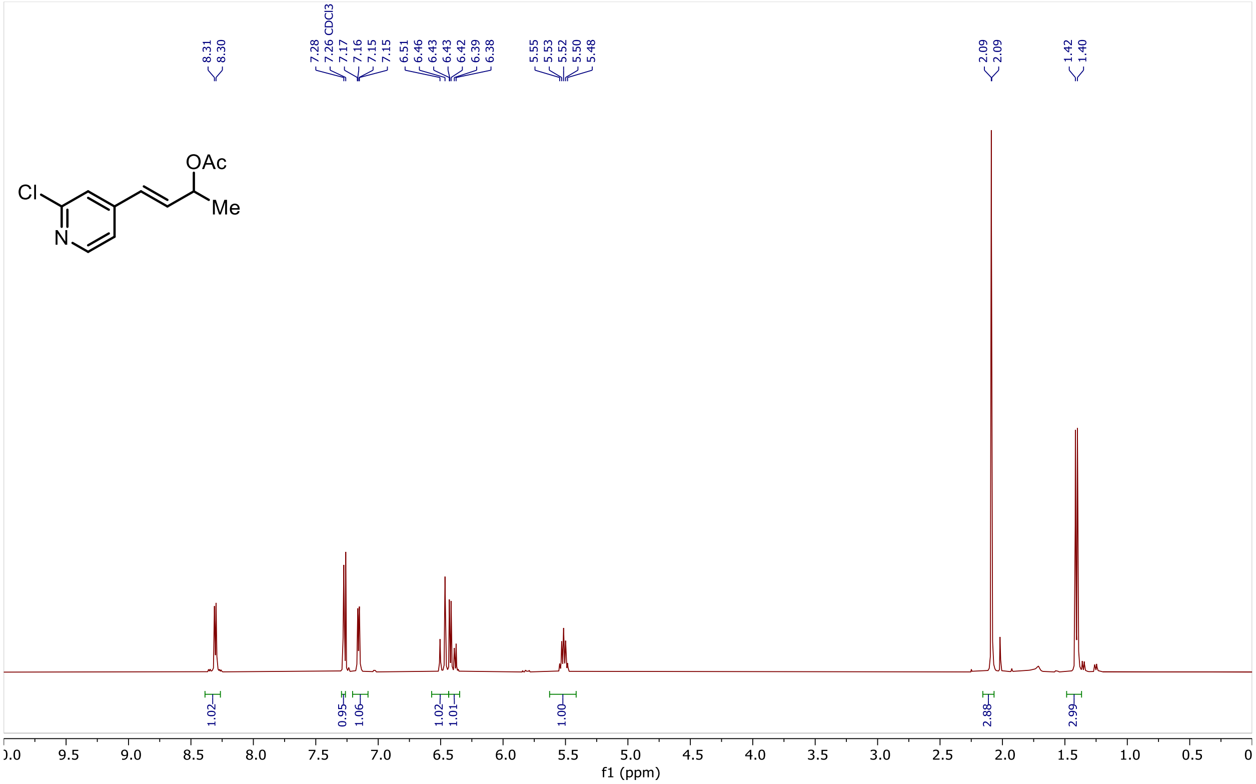

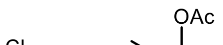

^{13}C NMR (101 MHz, $CDCl_3$) *rac*-2i-Ac

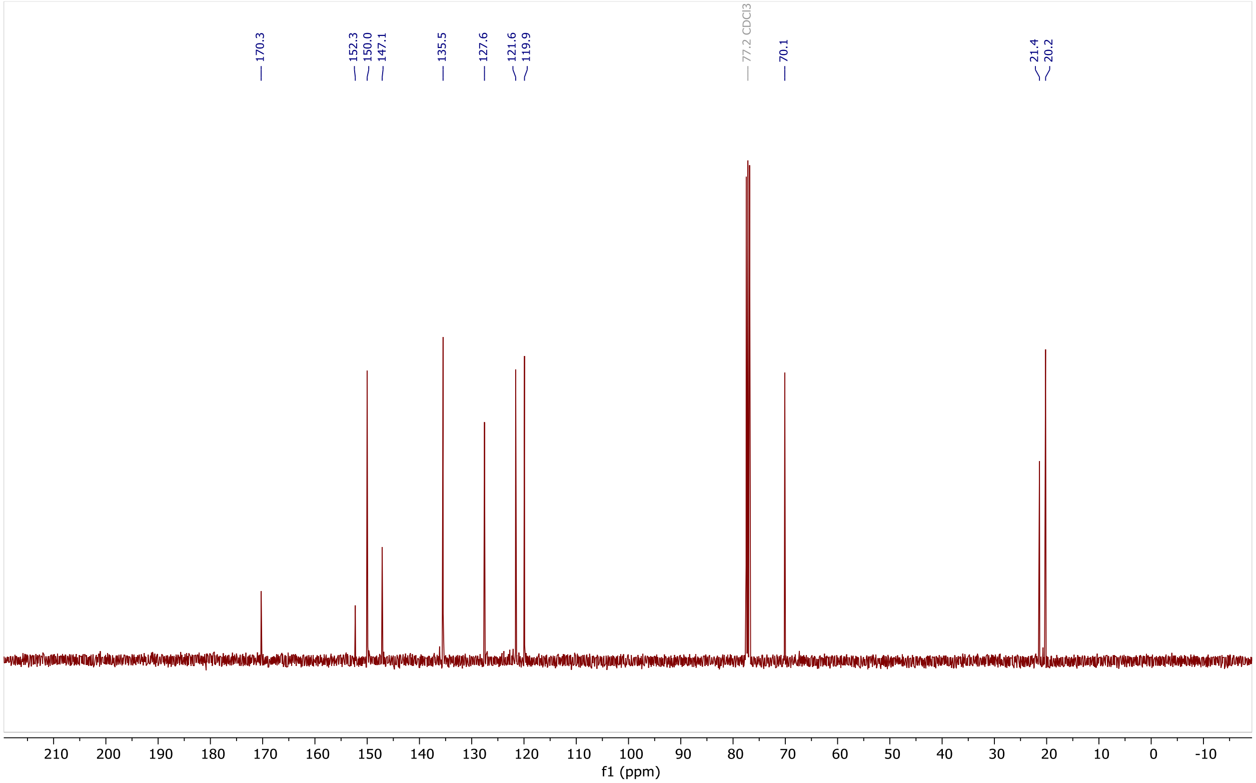

^{1}H NMR (400 MHz, $CDCl_3$) of *rac*-2j

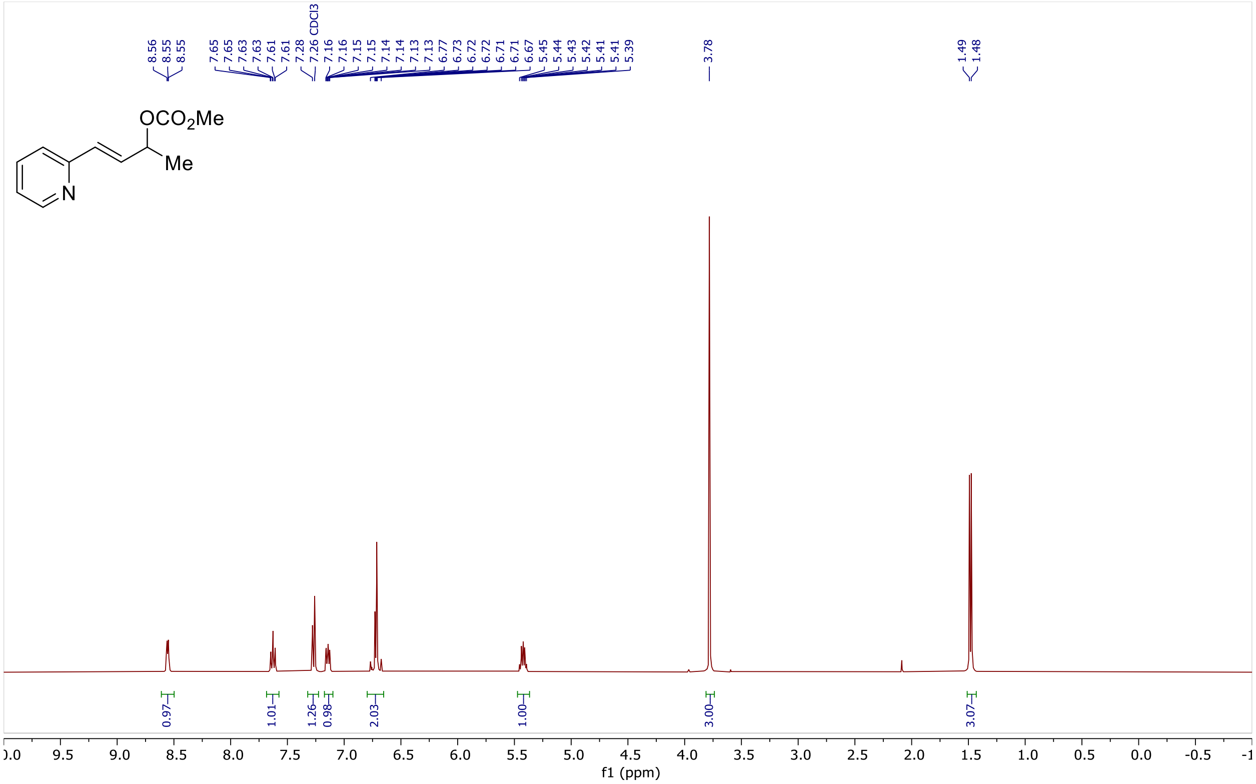

^{13}C NMR (101 MHz, $CDCl_3$) of *rac*-**2j**

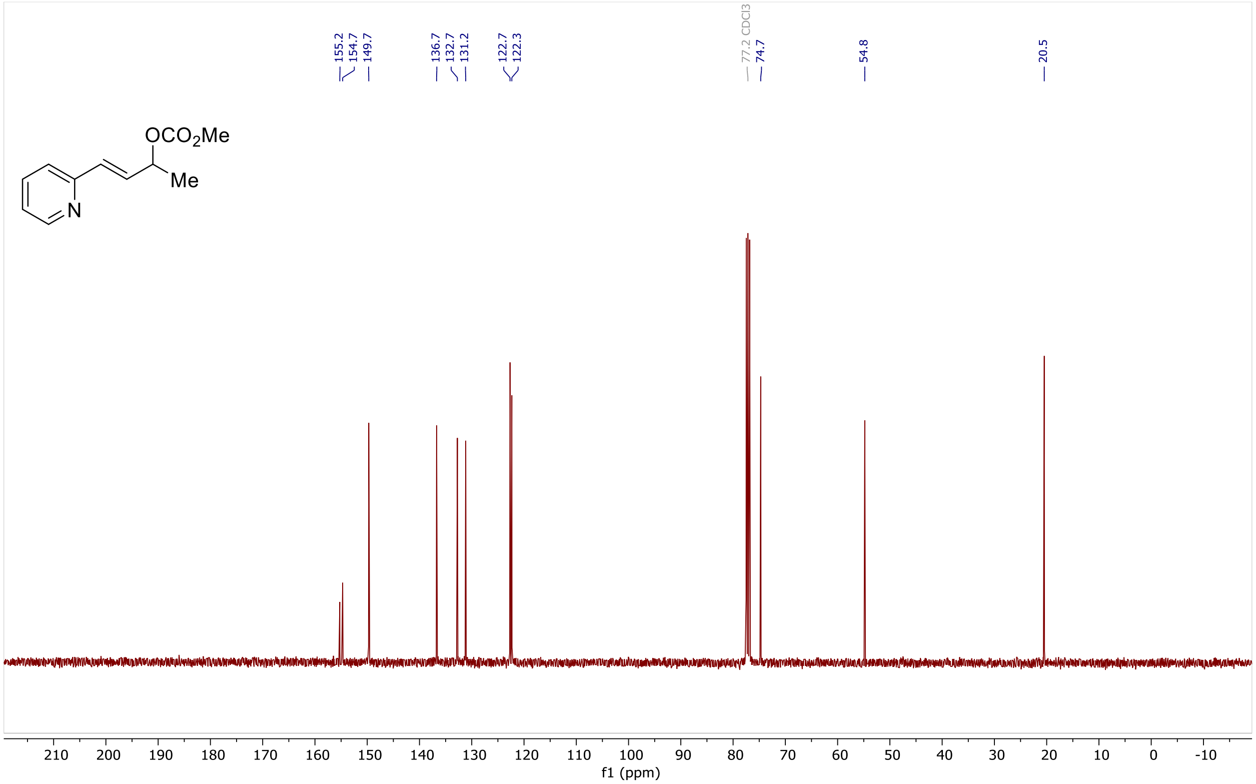

^{1}H NMR (400 MHz, $CDCl_3$) of *rac*-**2k-Ac**

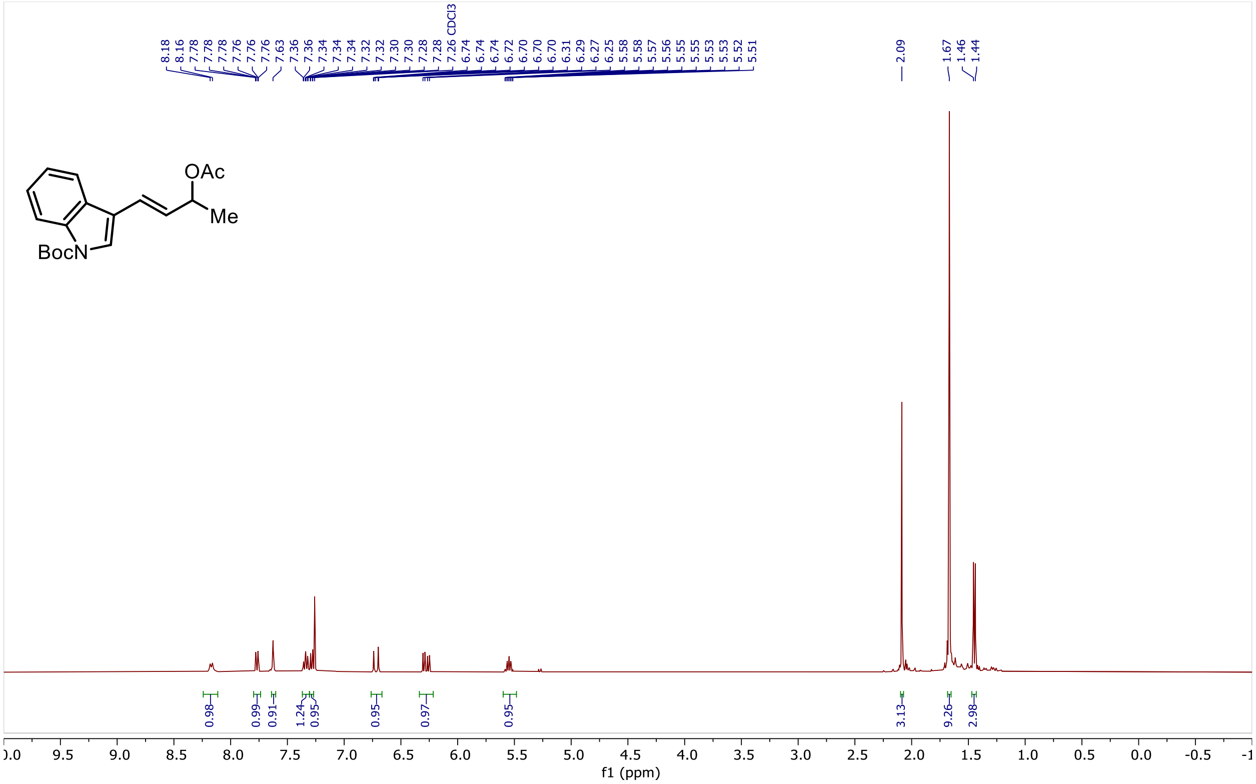

^{13}C NMR (101 MHz, CDCl$_3$) of ***rac*-2k-Ac**

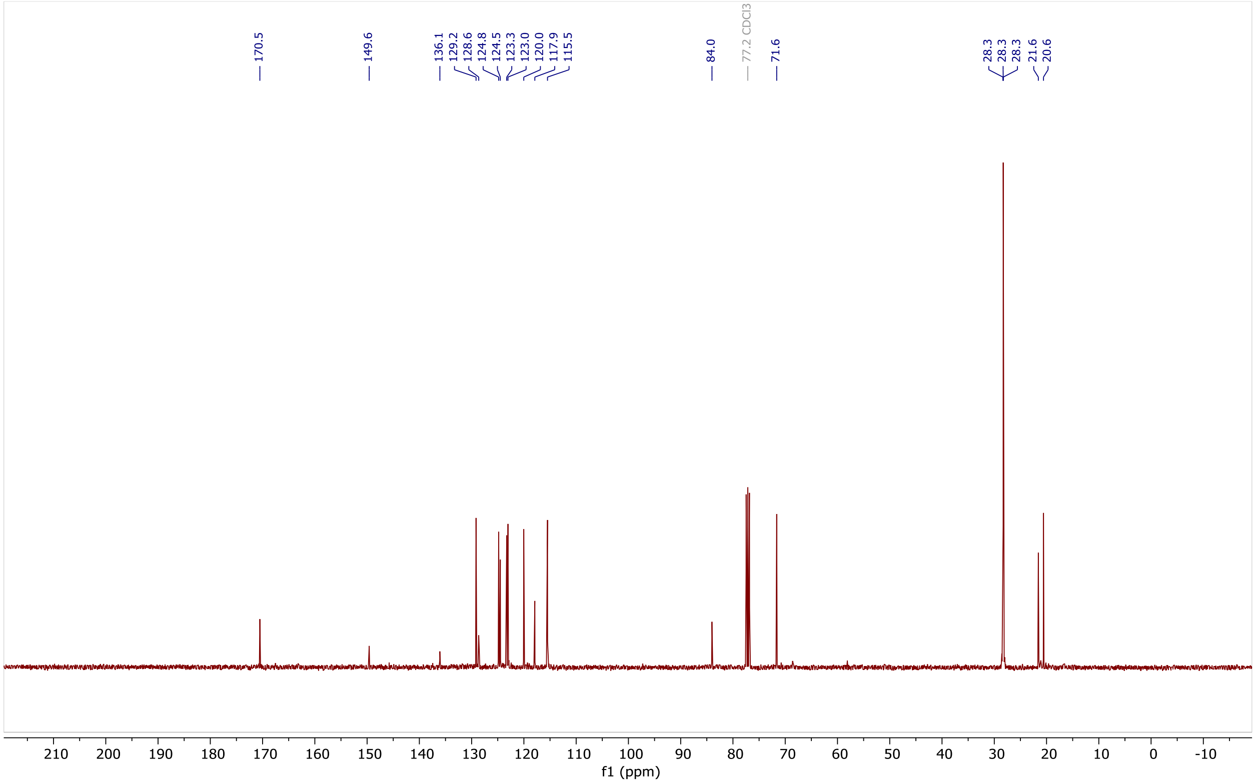

^{1}H NMR (400 MHz, CDCl$_3$) of **4a**

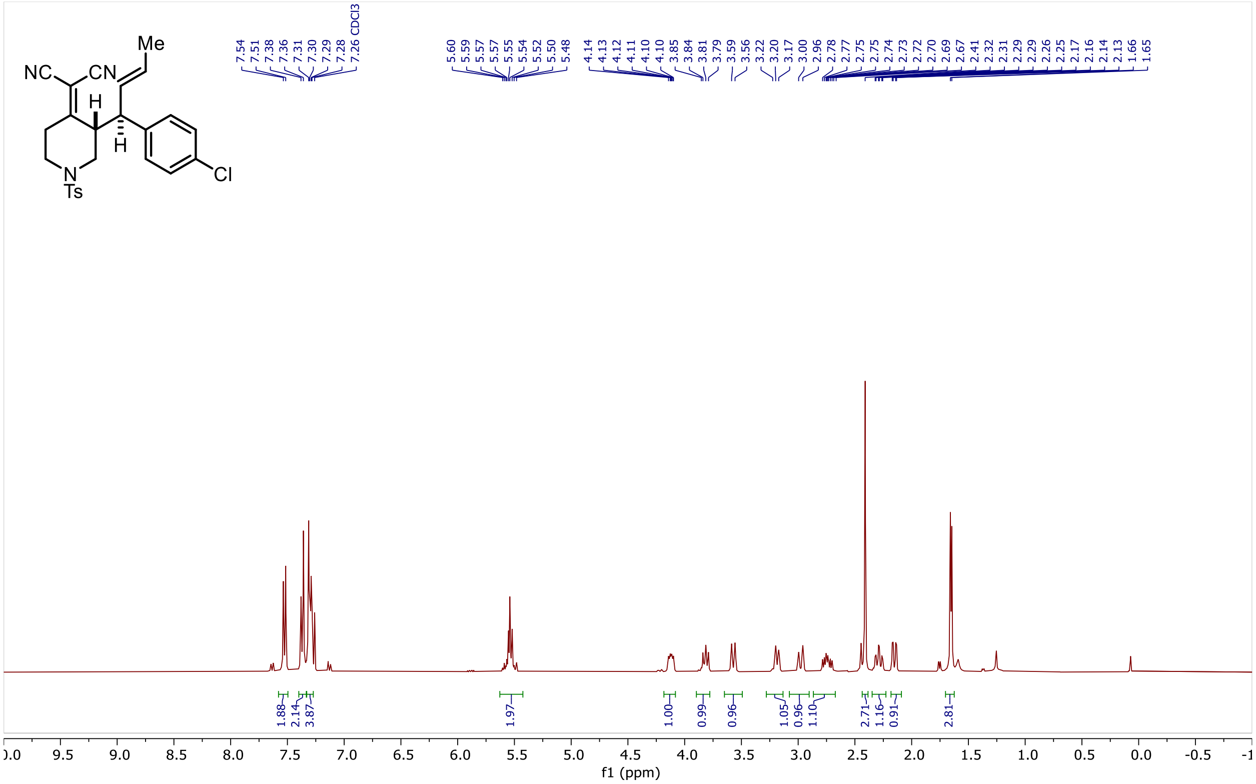

^{13}C NMR (101 MHz, CDCl$_3$) of 4a

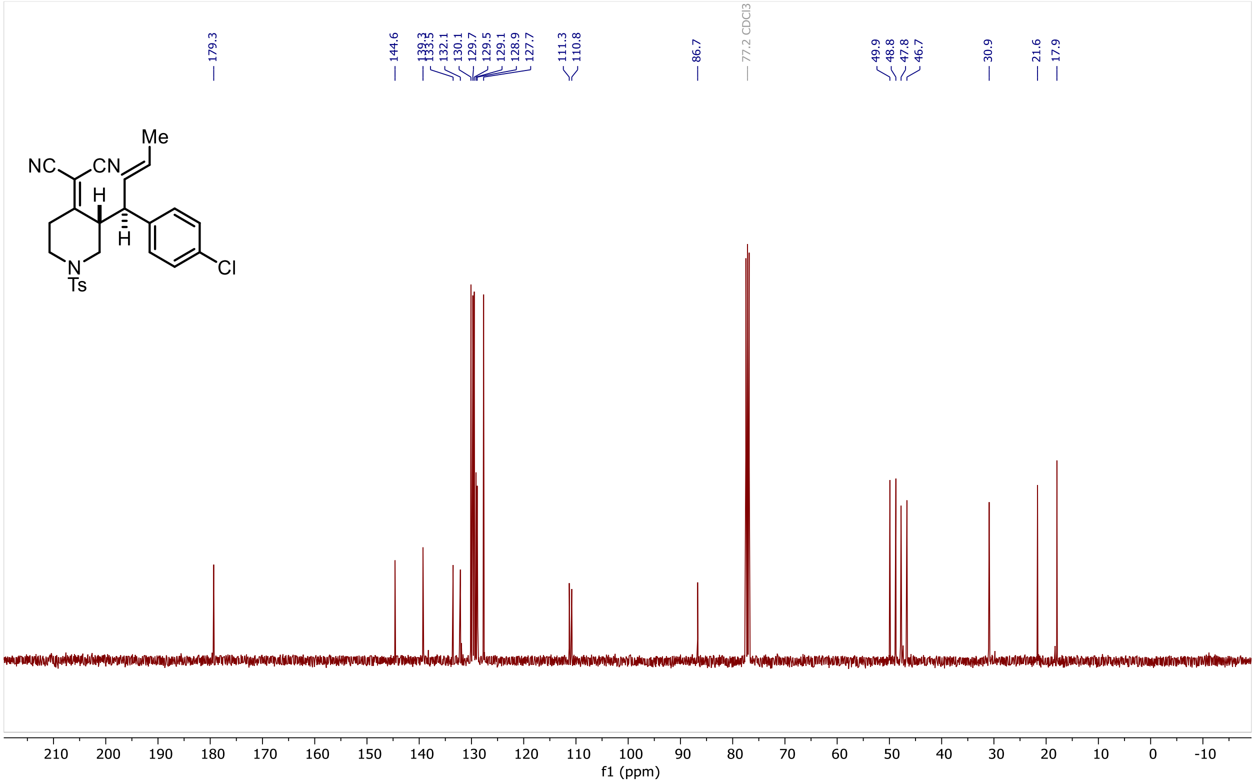

^{1}H NMR (400 MHz, CD$_3$CN) of 4b

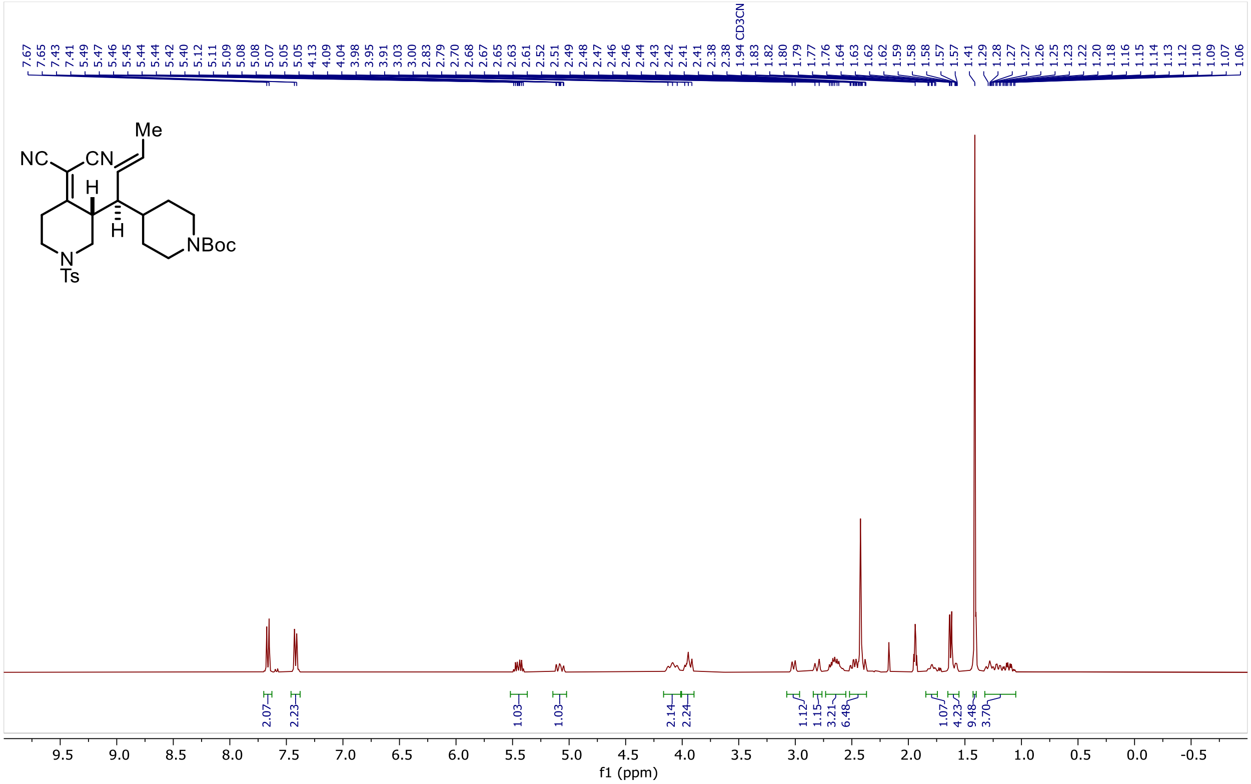

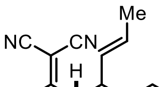

^{13}C NMR (101 MHz, CD$_3$CN) of 4b

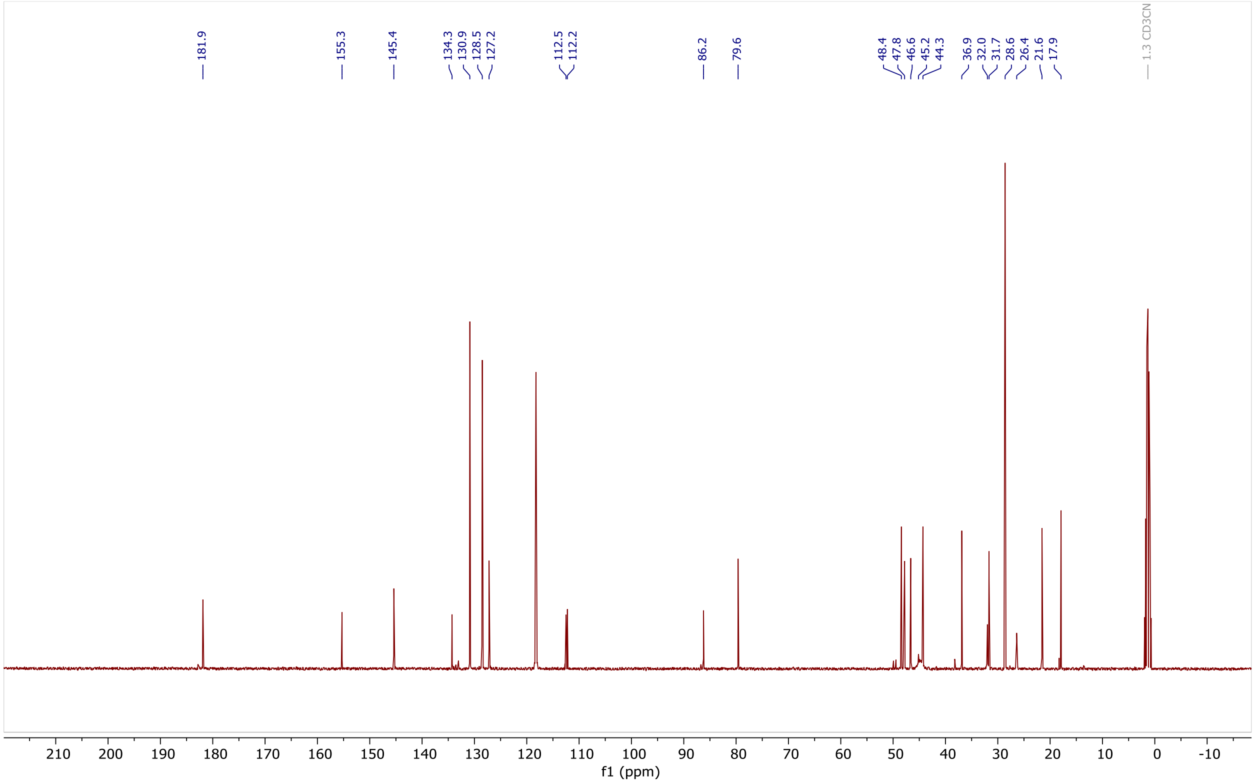

^{1}H NMR (400 MHz, CD$_3$CN) of 4c

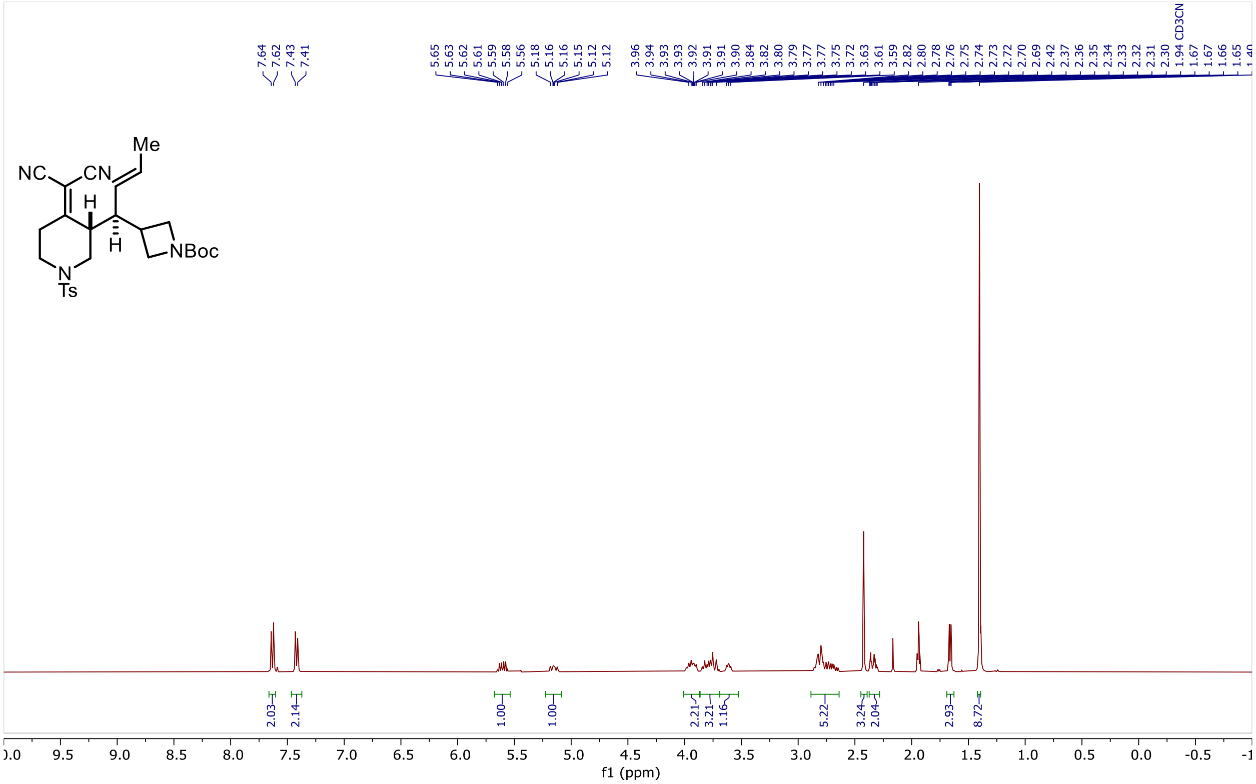

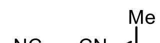

^{13}C NMR (101 MHz, CD_3CN) of 4c

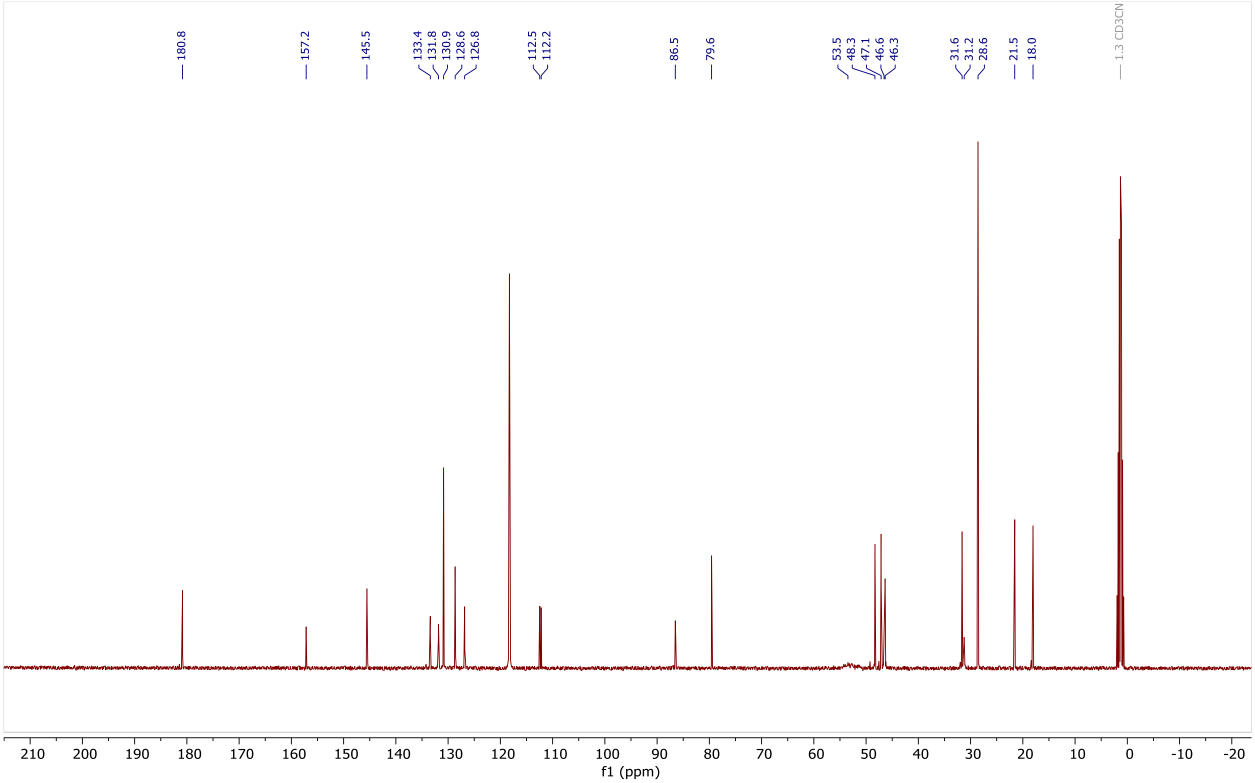

^{1}H NMR (400 MHz, $CDCl_3$) of 4d

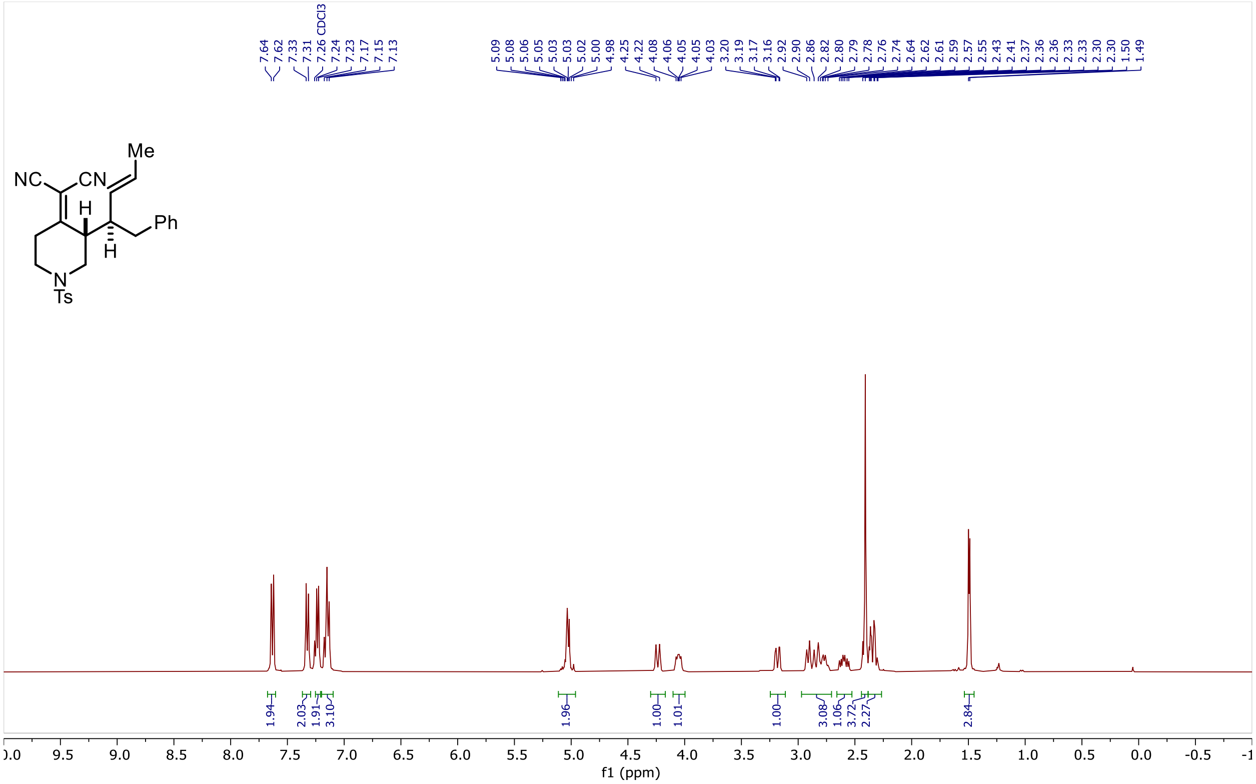

^{13}C NMR (101 MHz, CDCl$_3$) of **4d**

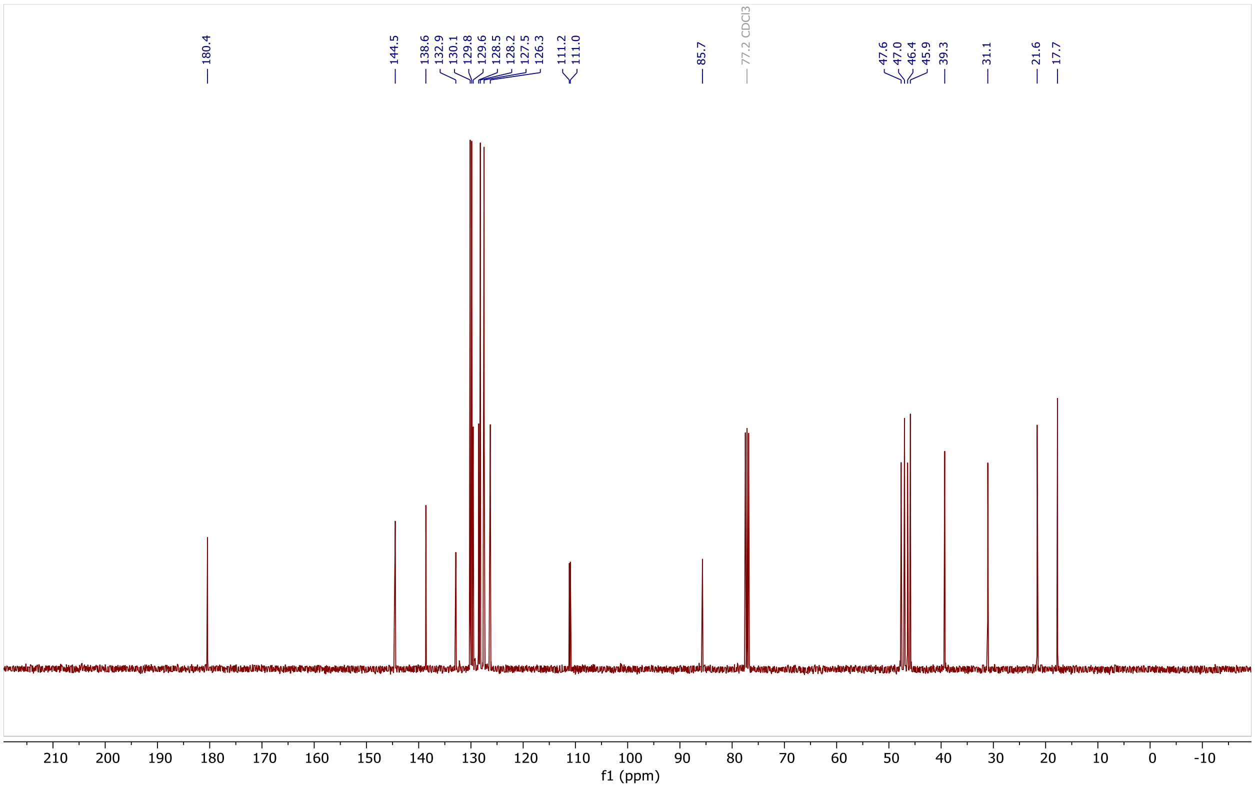

^{1}H NMR (400 MHz, CDCl$_3$) of **4e**

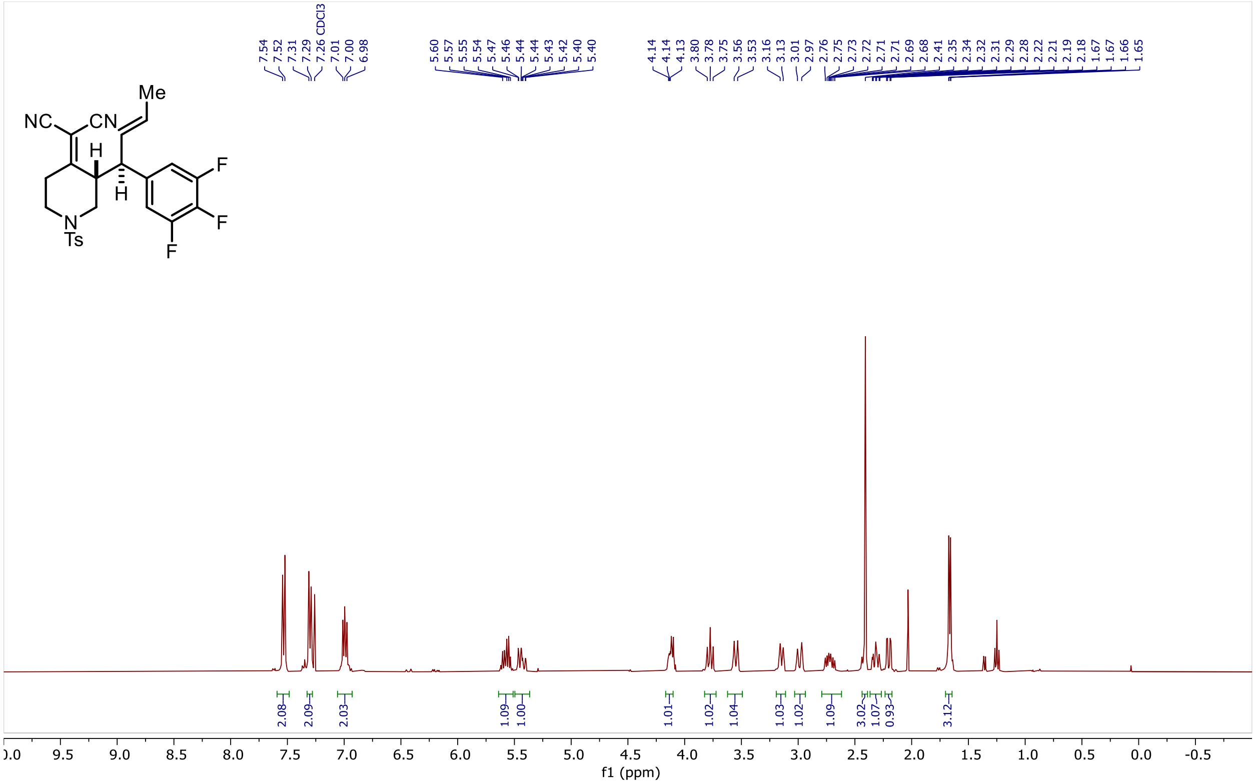

^{13}C NMR (101 MHz, $CDCl_3$) of 4e

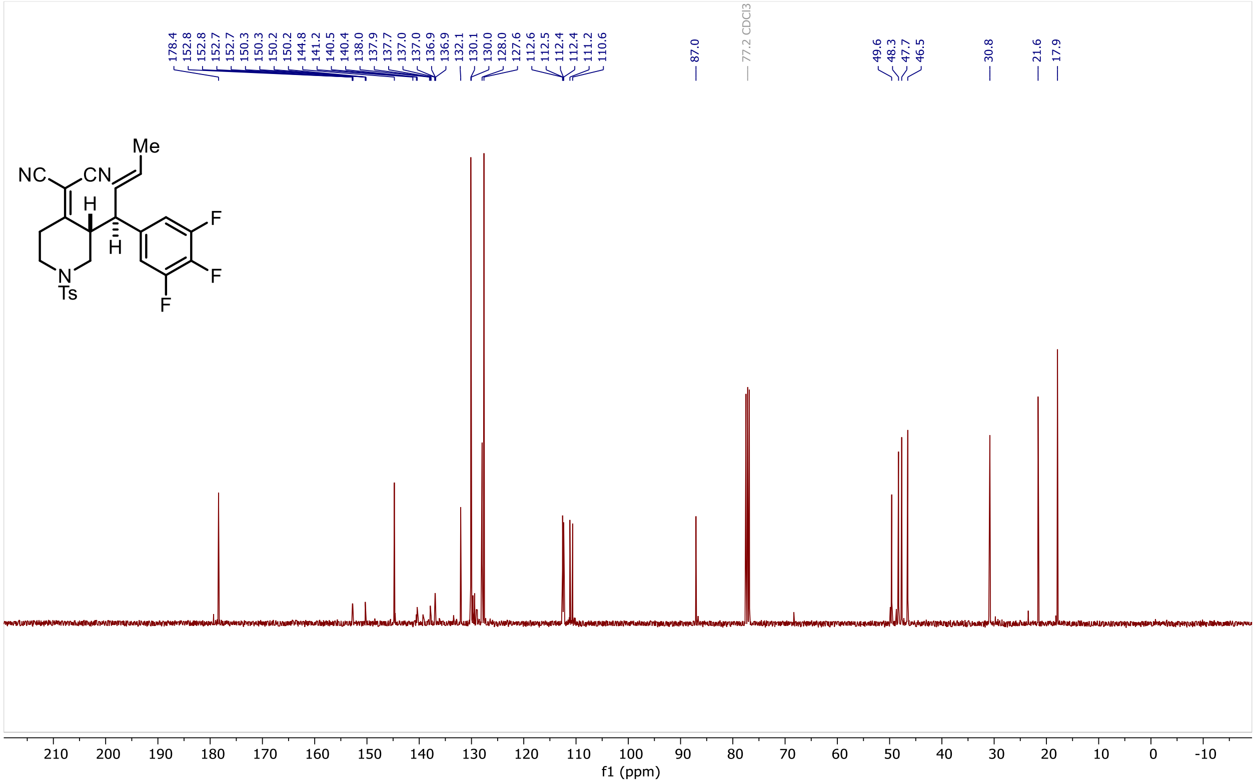

^{19}F NMR (377 MHz, $CDCl_3$) of 4e

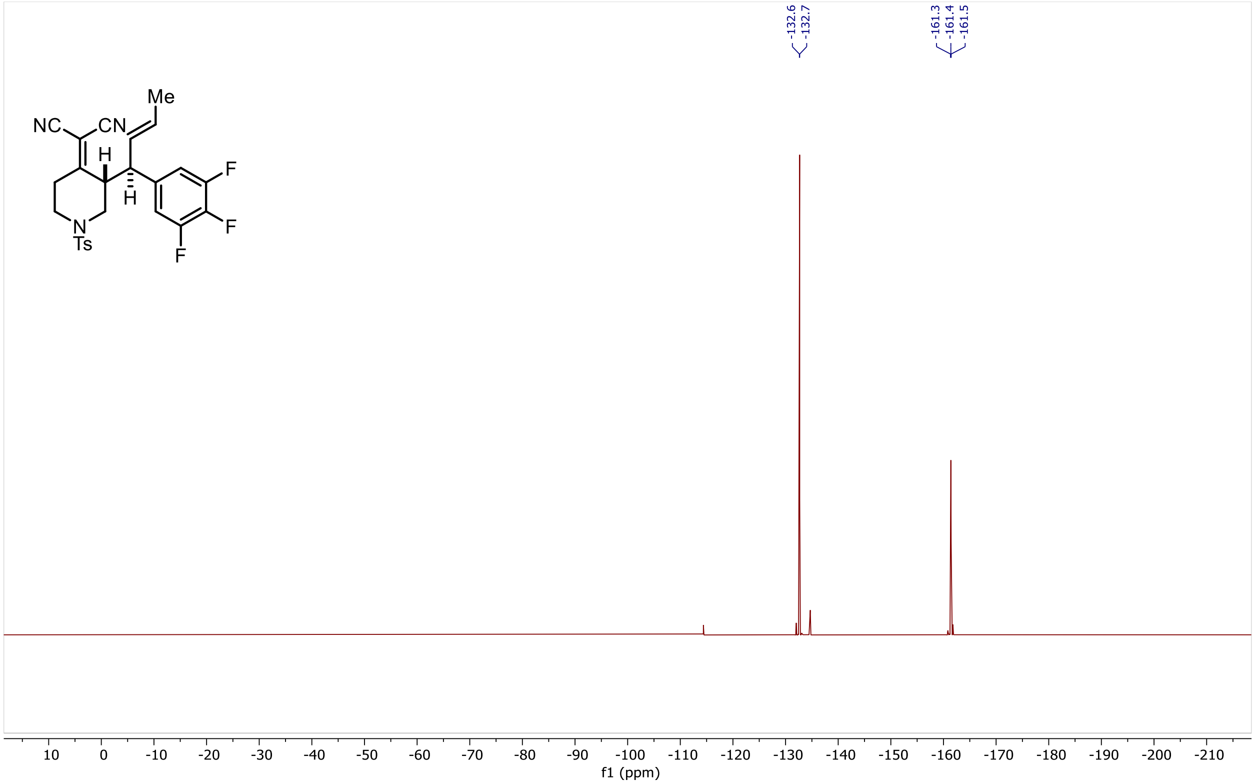

^{1}H NMR (400 MHz, CDCl$_3$) of 4f

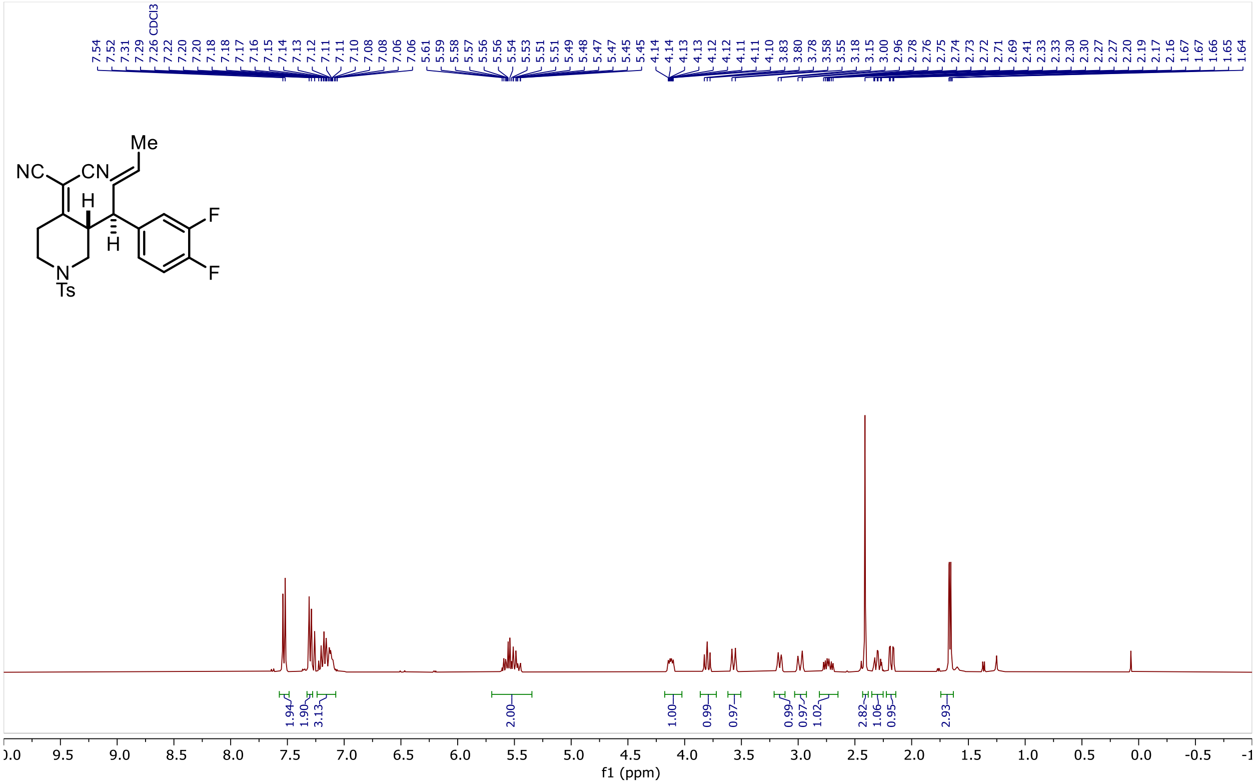

^{13}C NMR (101 MHz, CDCl$_3$) of 4f

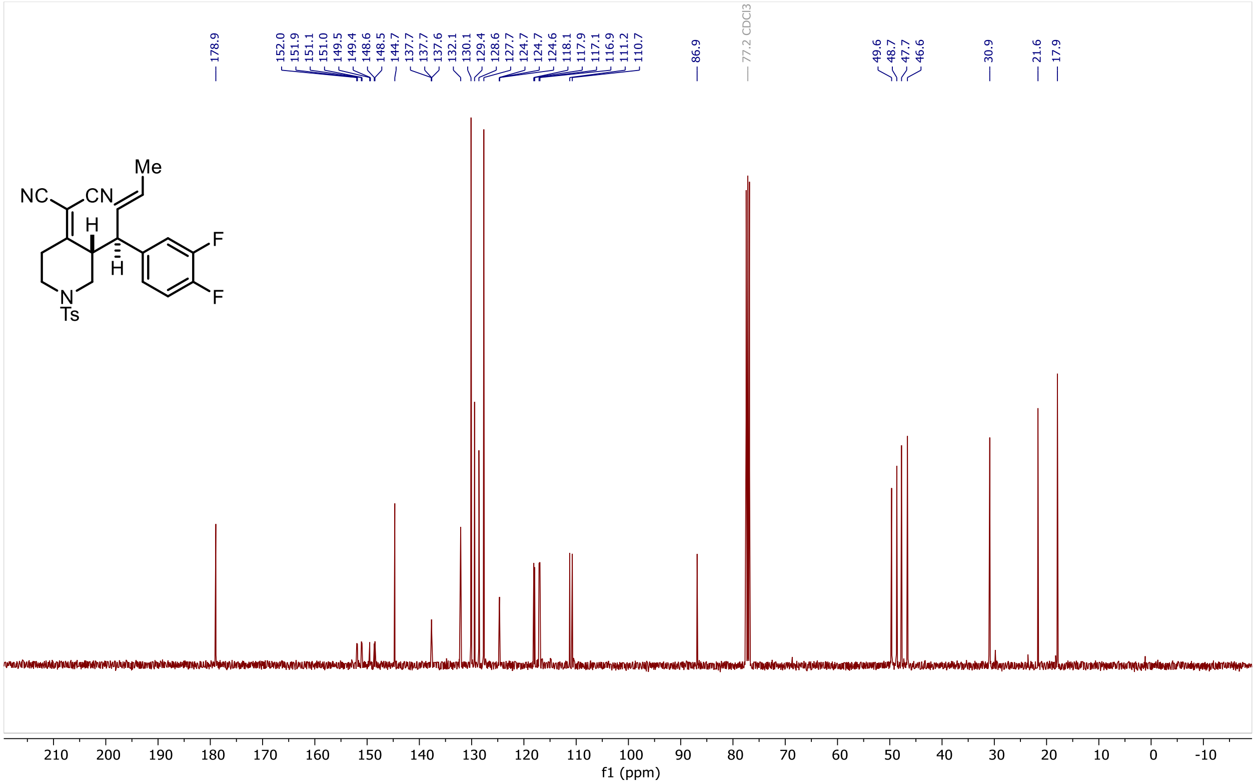

^{19}F NMR (377 MHz, CDCl$_3$) of 4f

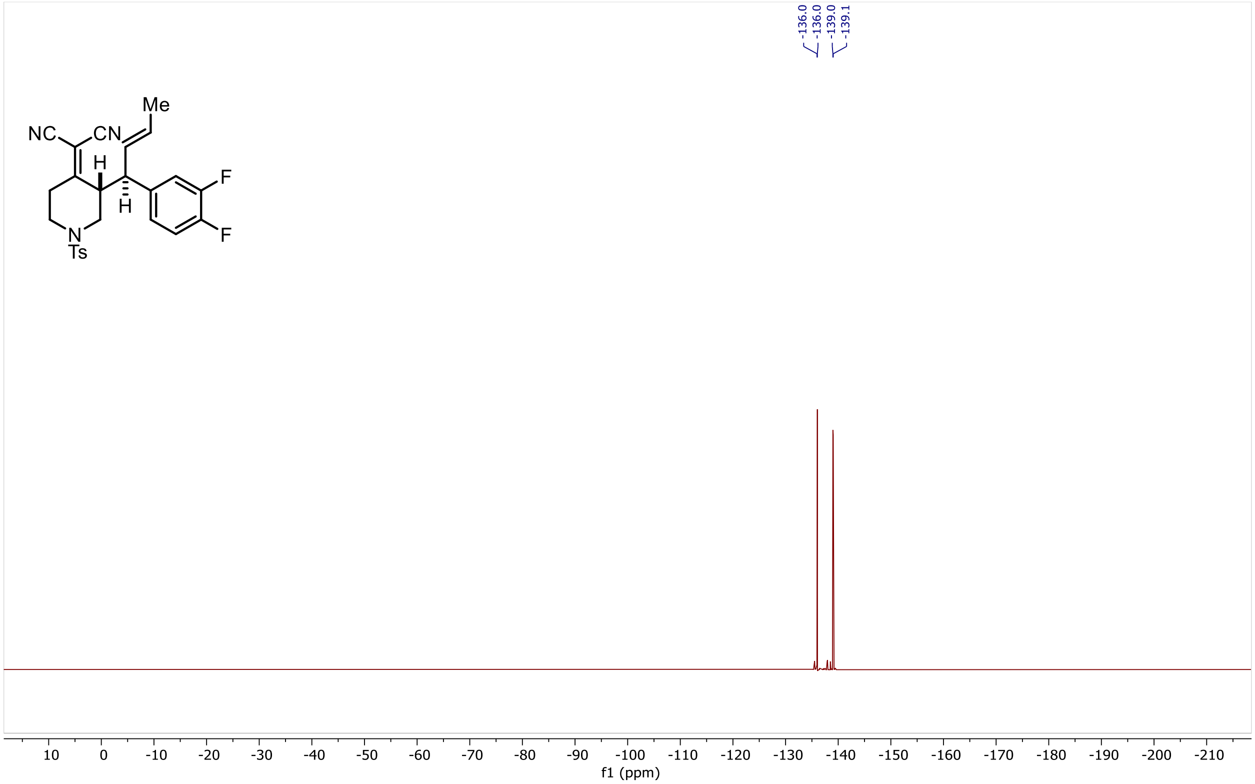

^{1}H NMR (400 MHz, CDCl$_3$) of 4g

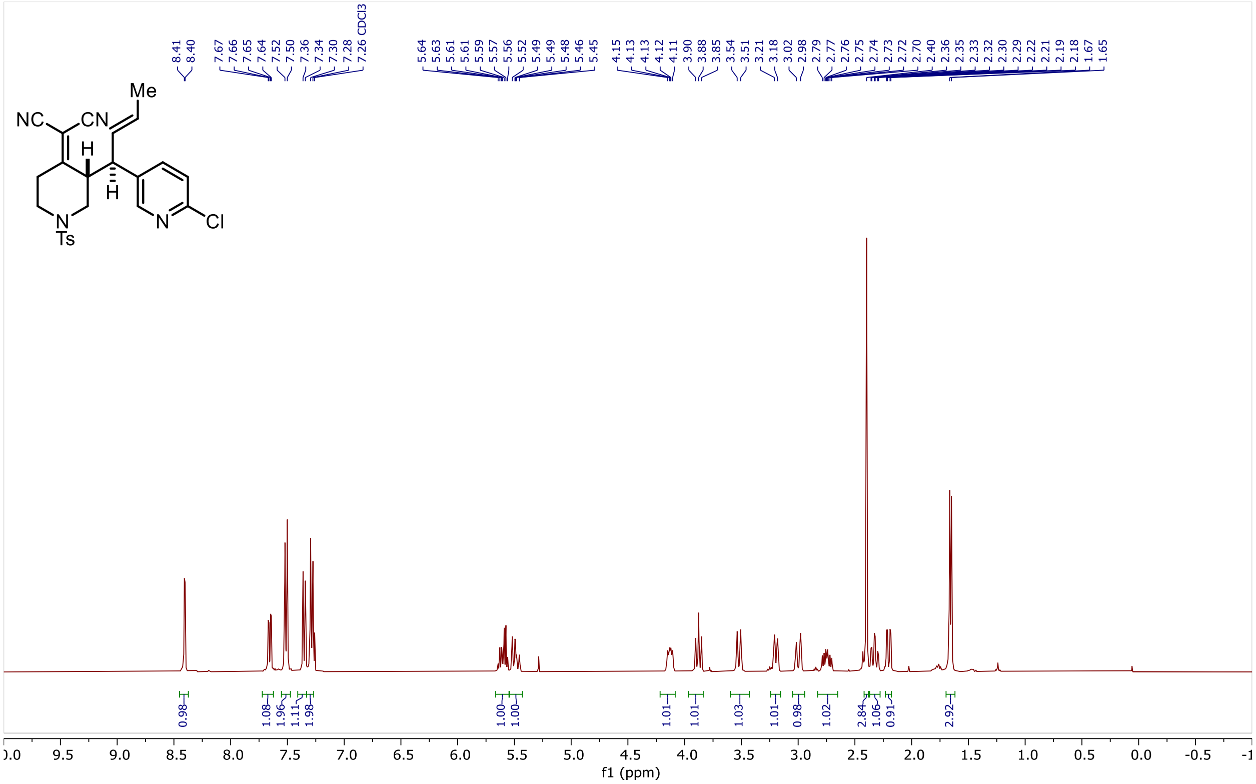

^{13}C NMR (101 MHz, CDCl$_3$) of 4g

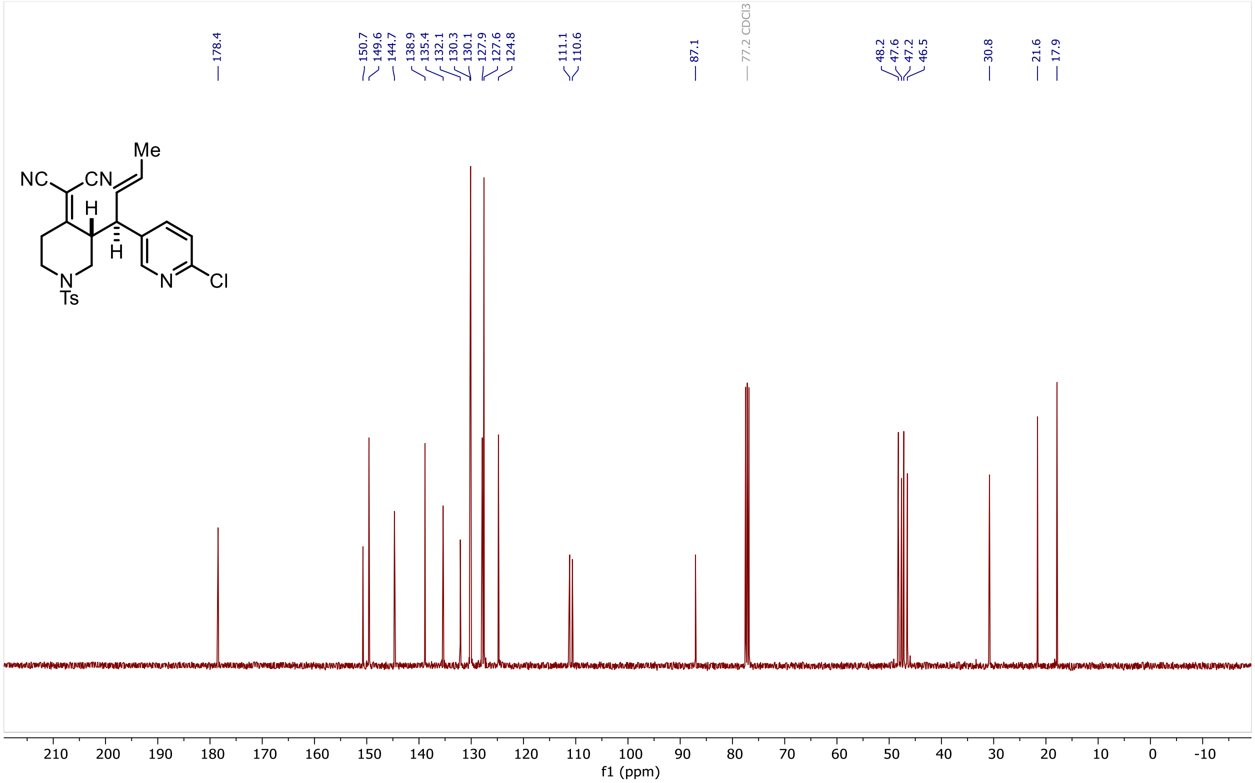

^{1}H NMR (400 MHz, CDCl$_3$) of 4h

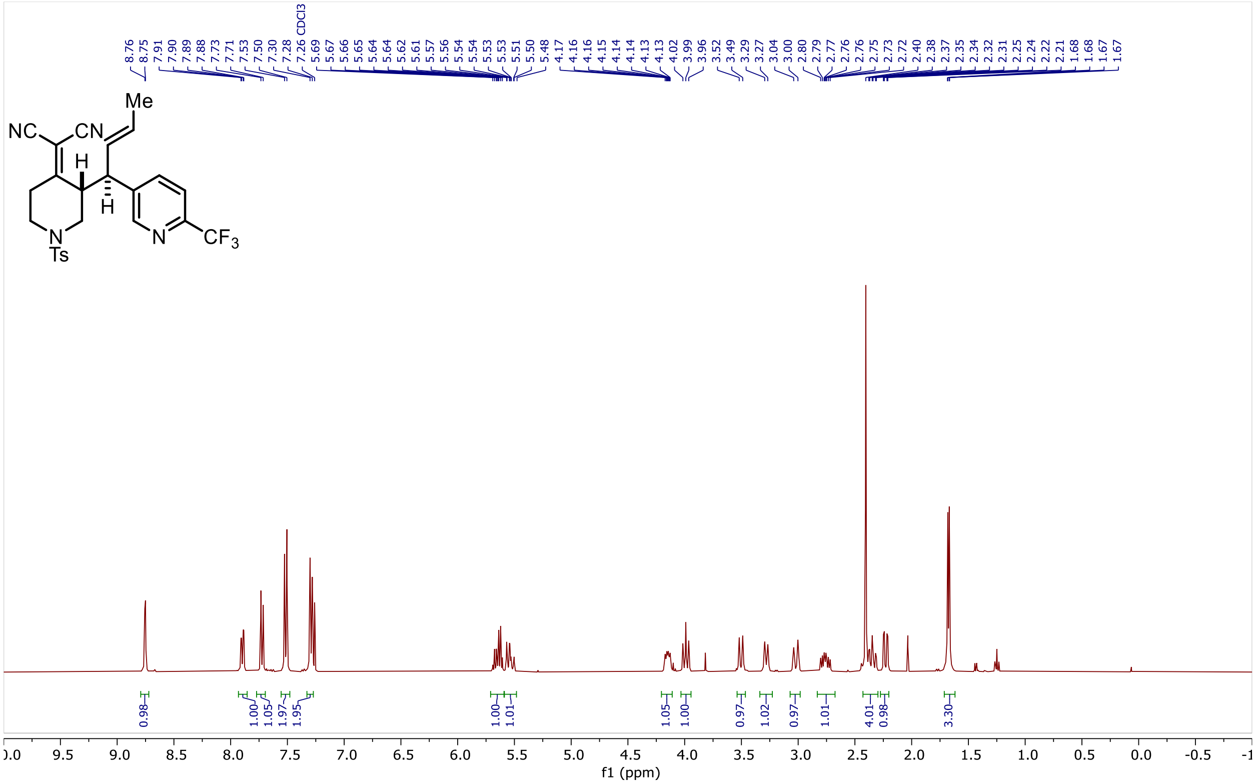

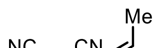

^{13}C NMR (101 MHz, CDCl$_3$) of **4h**

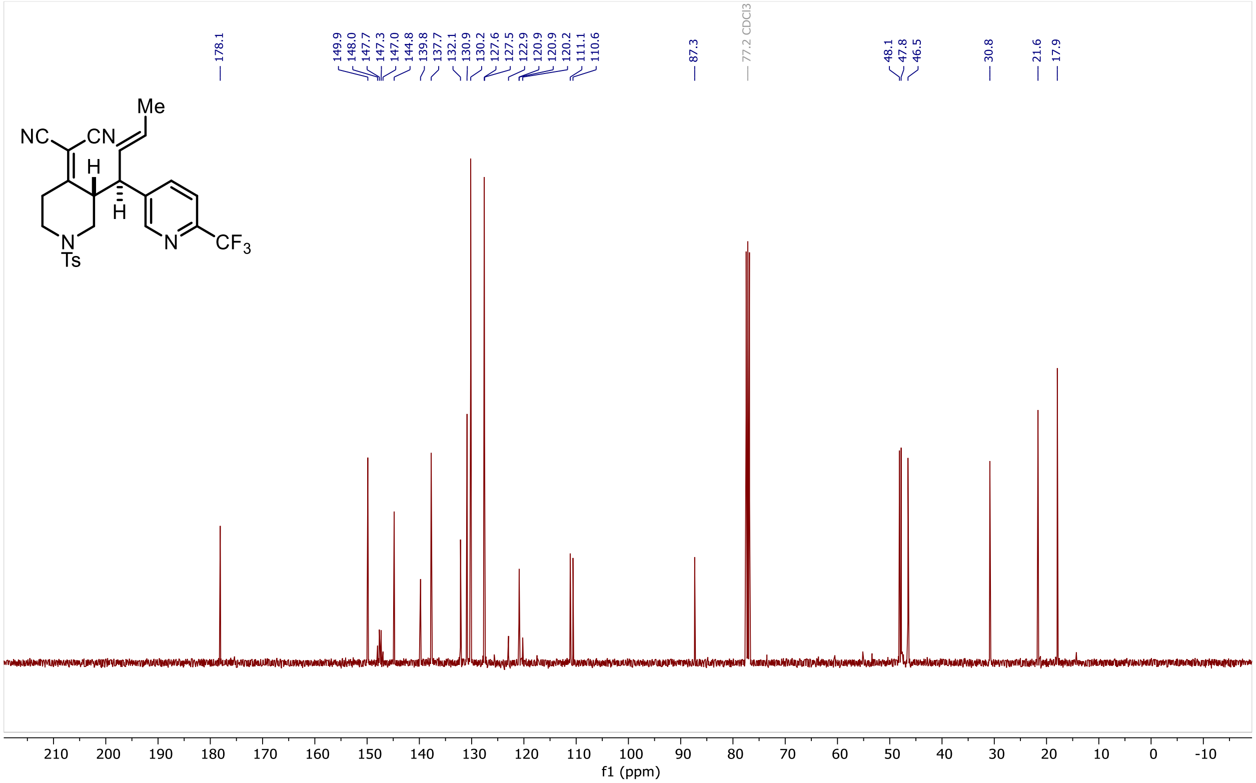

^{19}F NMR (377 MHz, CDCl$_3$) of **4h**

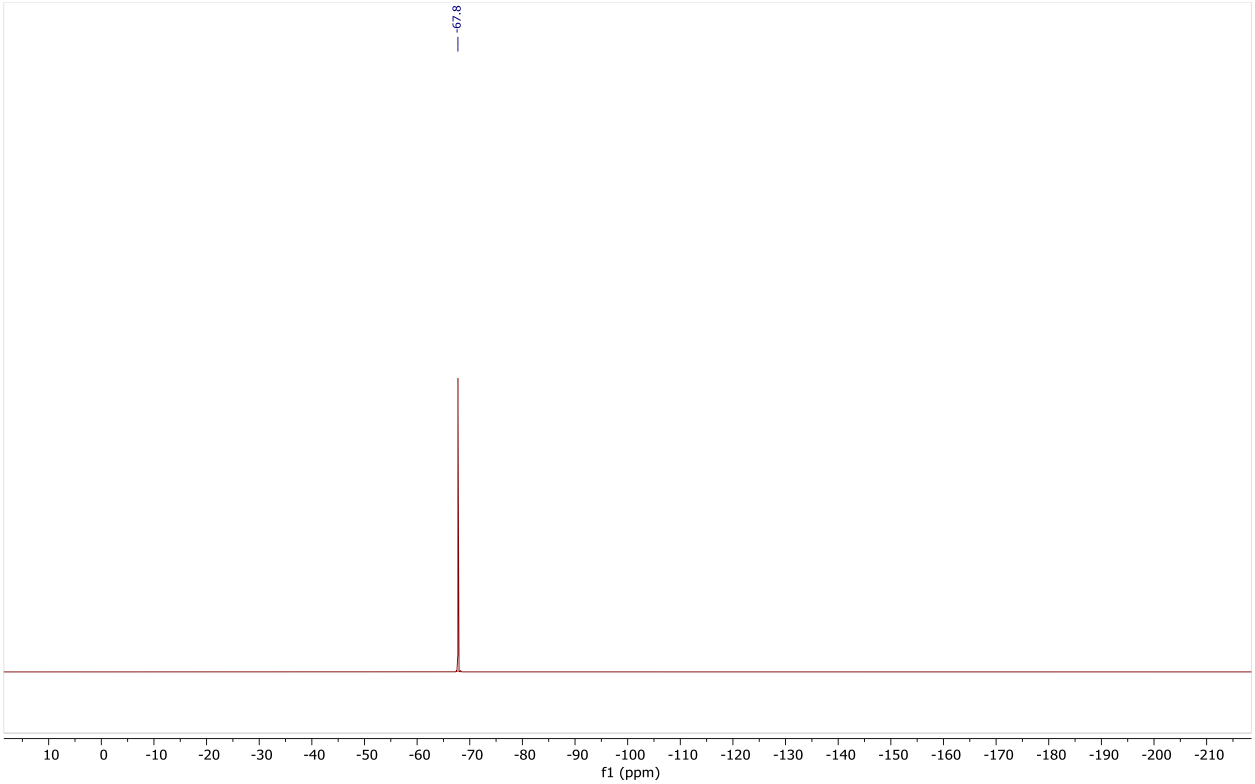

^{1}H NMR (400 MHz, CDCl₃) of 4i

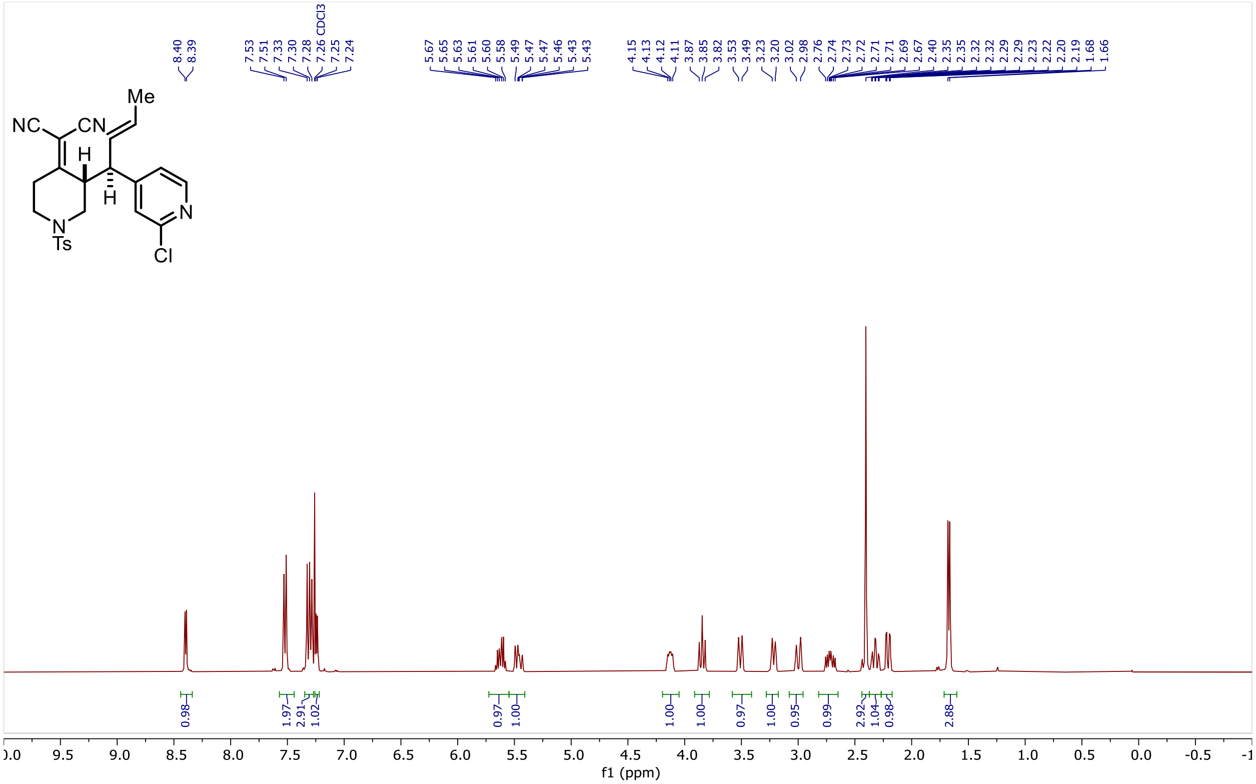

^{13}C NMR (101 MHz, CDCl₃) of 4i

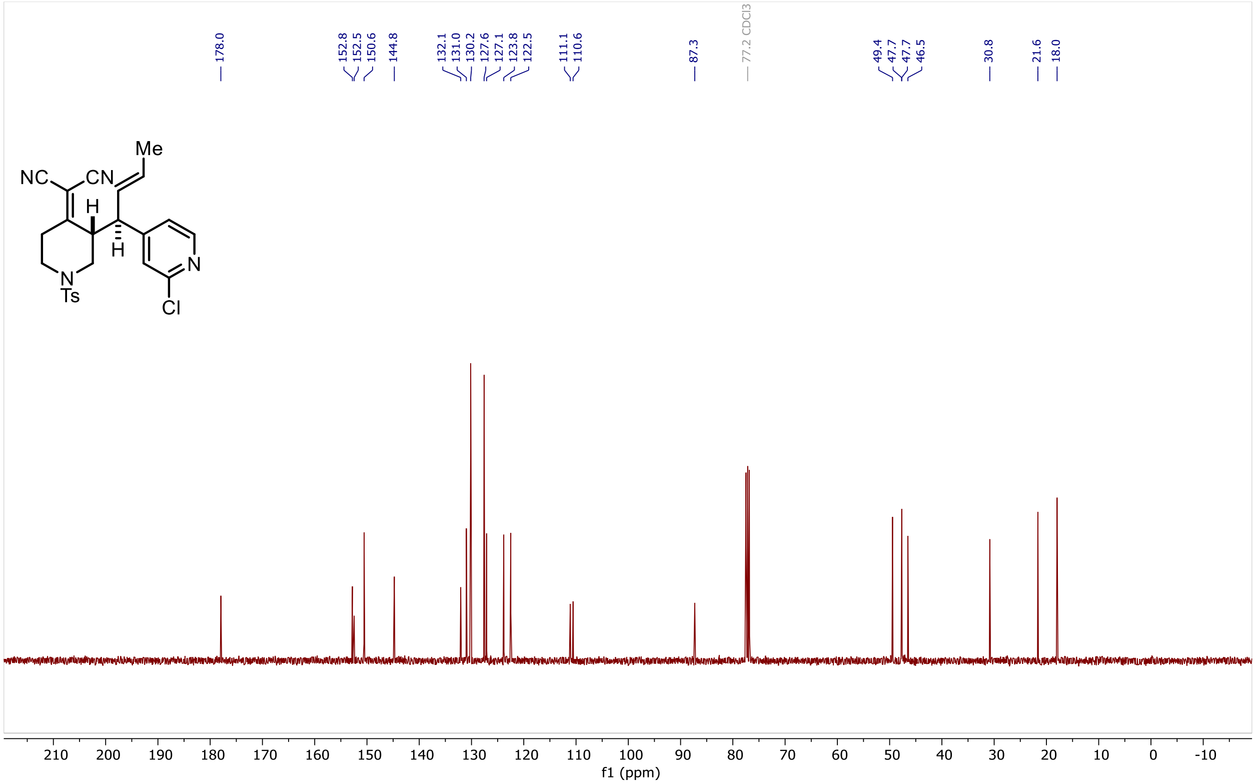

^{1}H NMR (400 MHz, CDCl$_3$) of **4j**

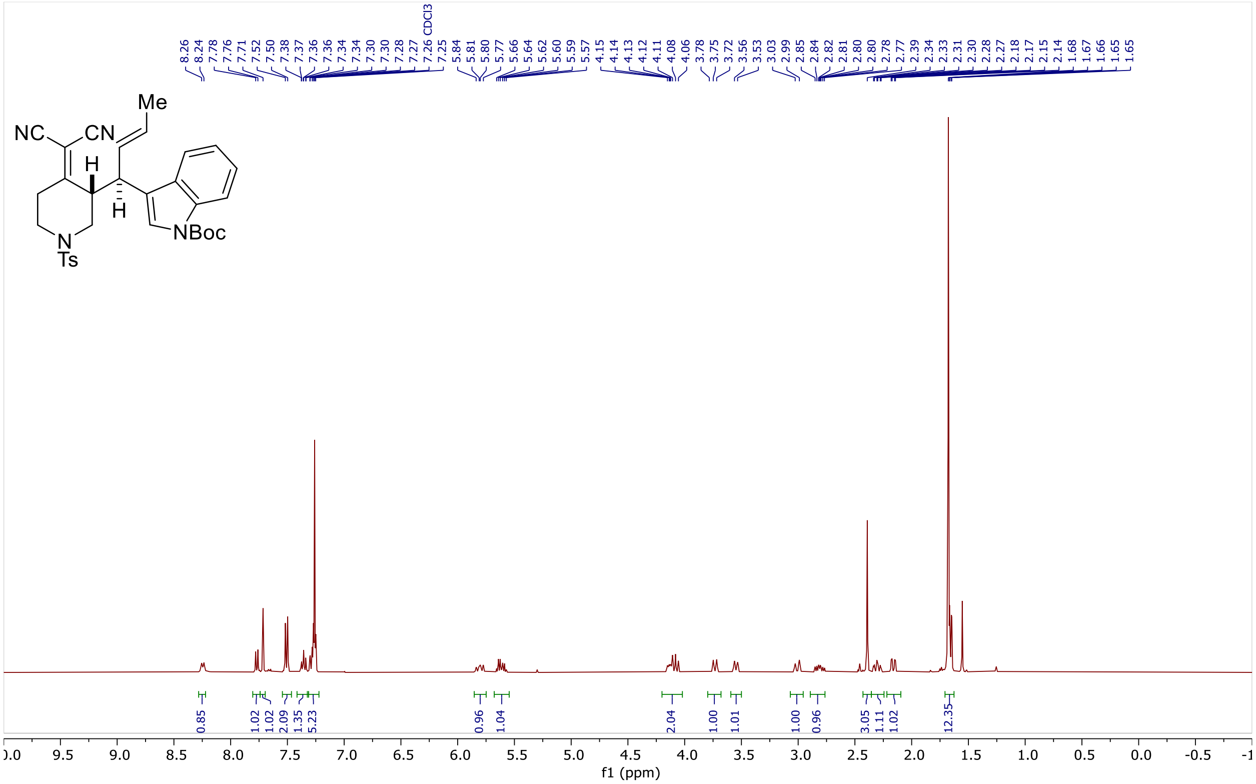

^{13}C NMR (101 MHz, CDCl$_3$) of **4j**

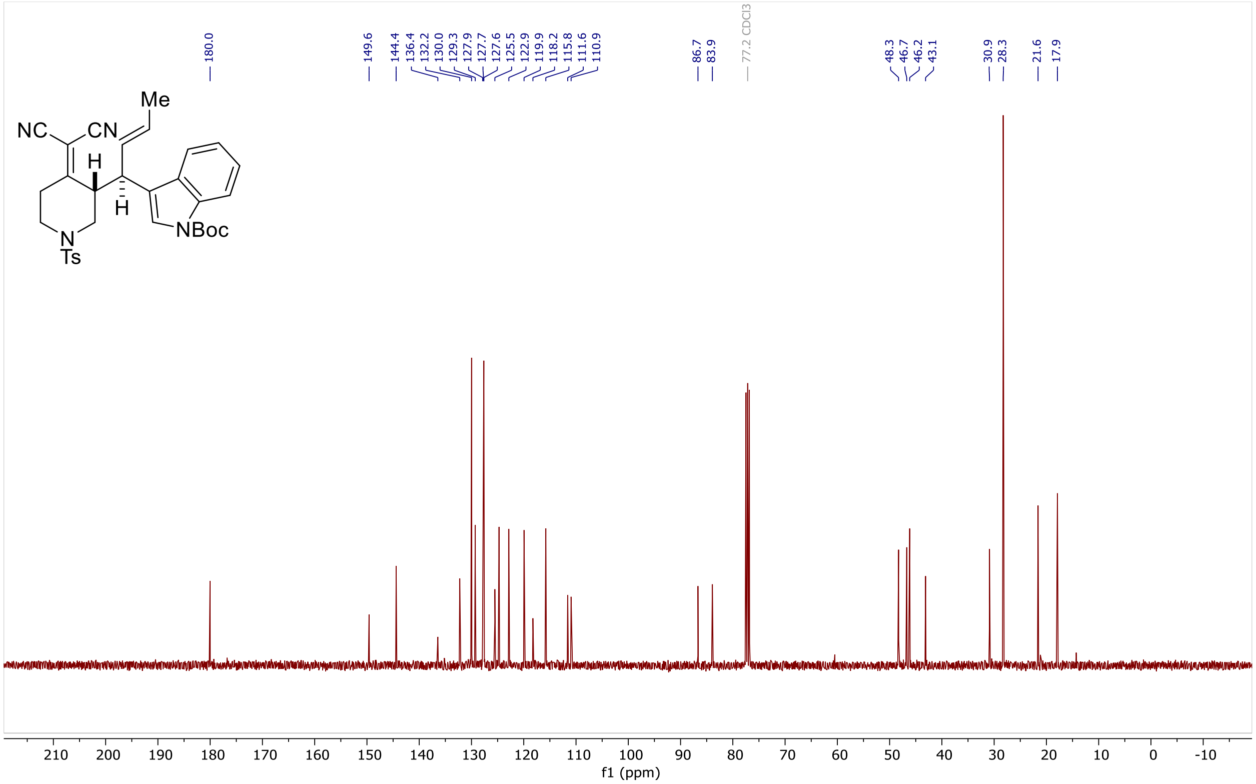

^{1}H NMR (400 MHz, CDCl$_3$) of 4k

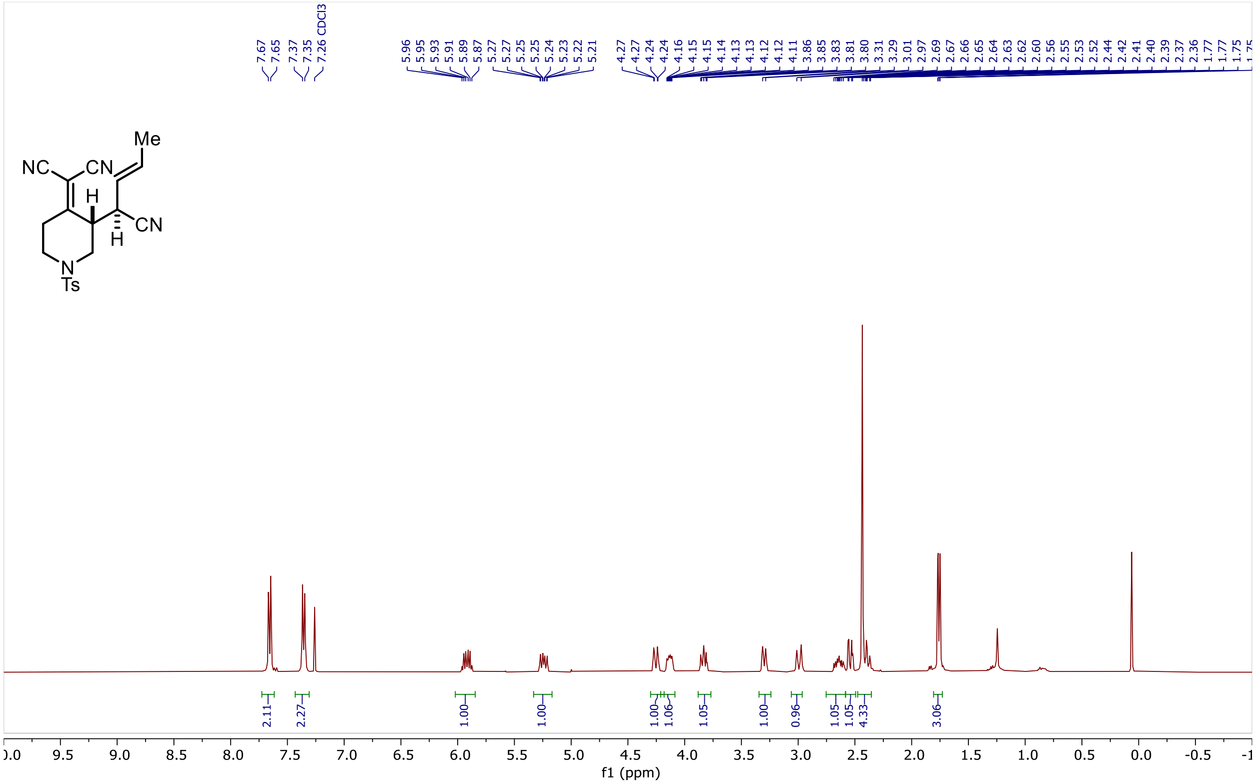

^{13}C NMR (101 MHz, CDCl$_3$) of 4k

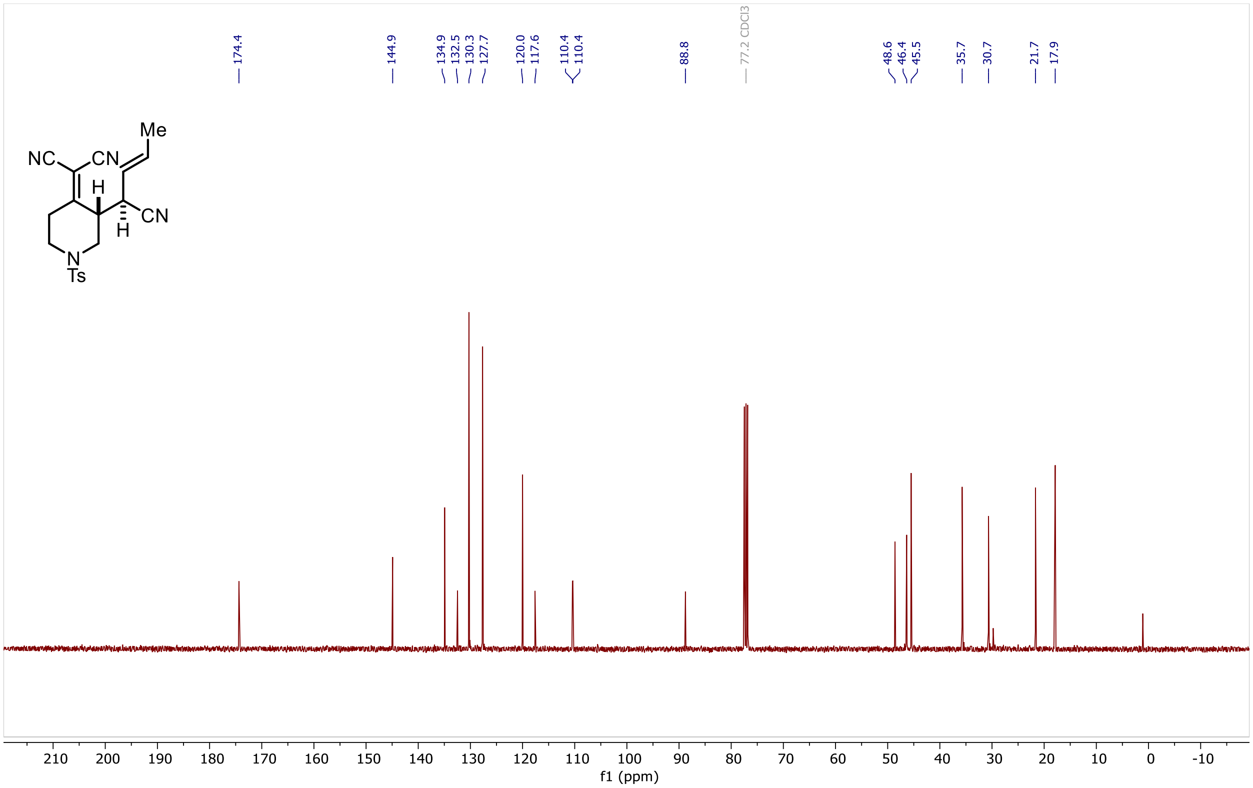

^{1}H NMR (500 MHz, DMSO-*d_6*, 353 K) of 4l

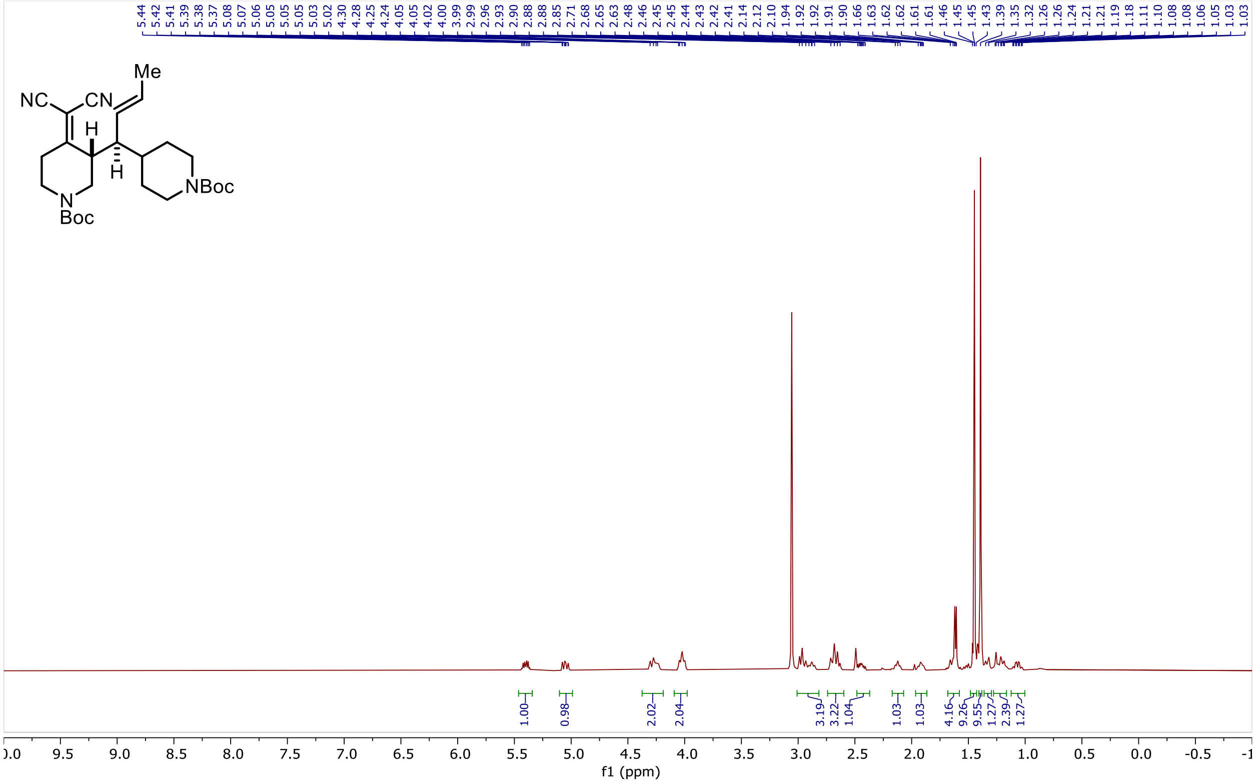

^{13}C NMR (126 MHz, DMSO-*d_6*, 353 K) of 4l

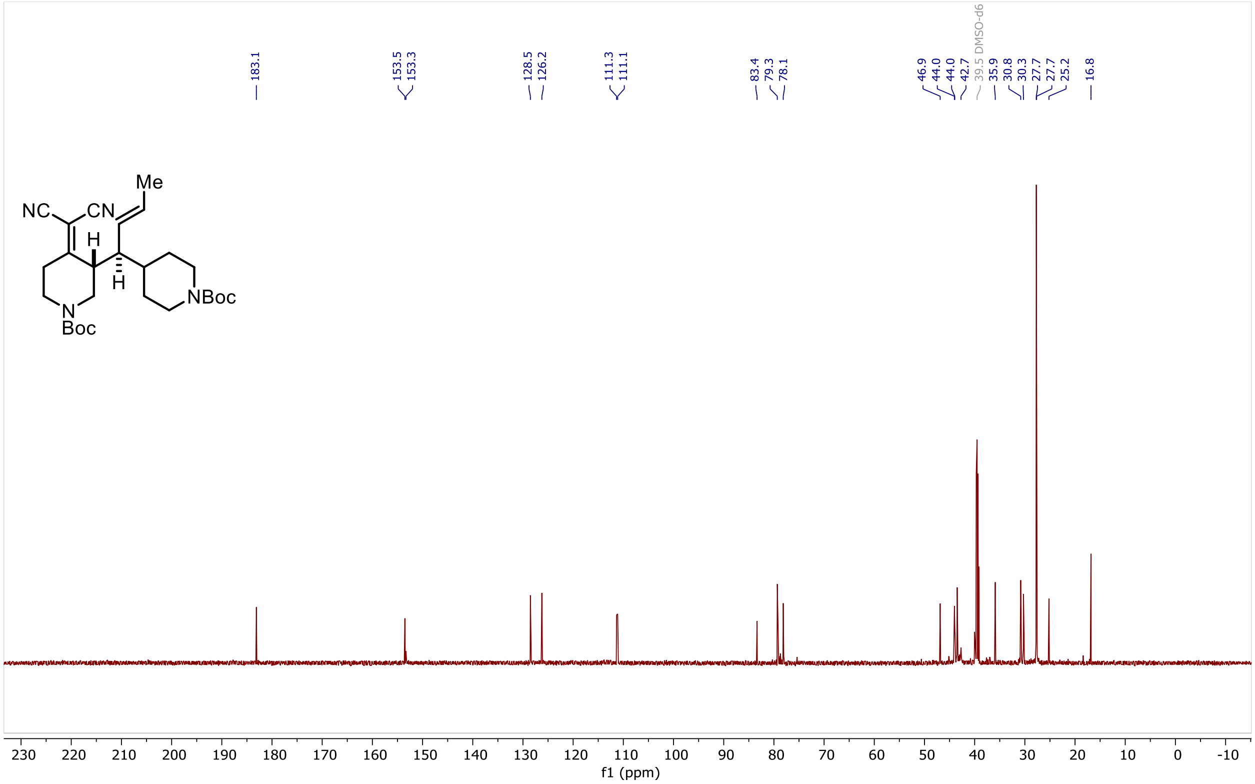

^{1}H NMR (400 MHz, CDCl$_3$) of **4m**

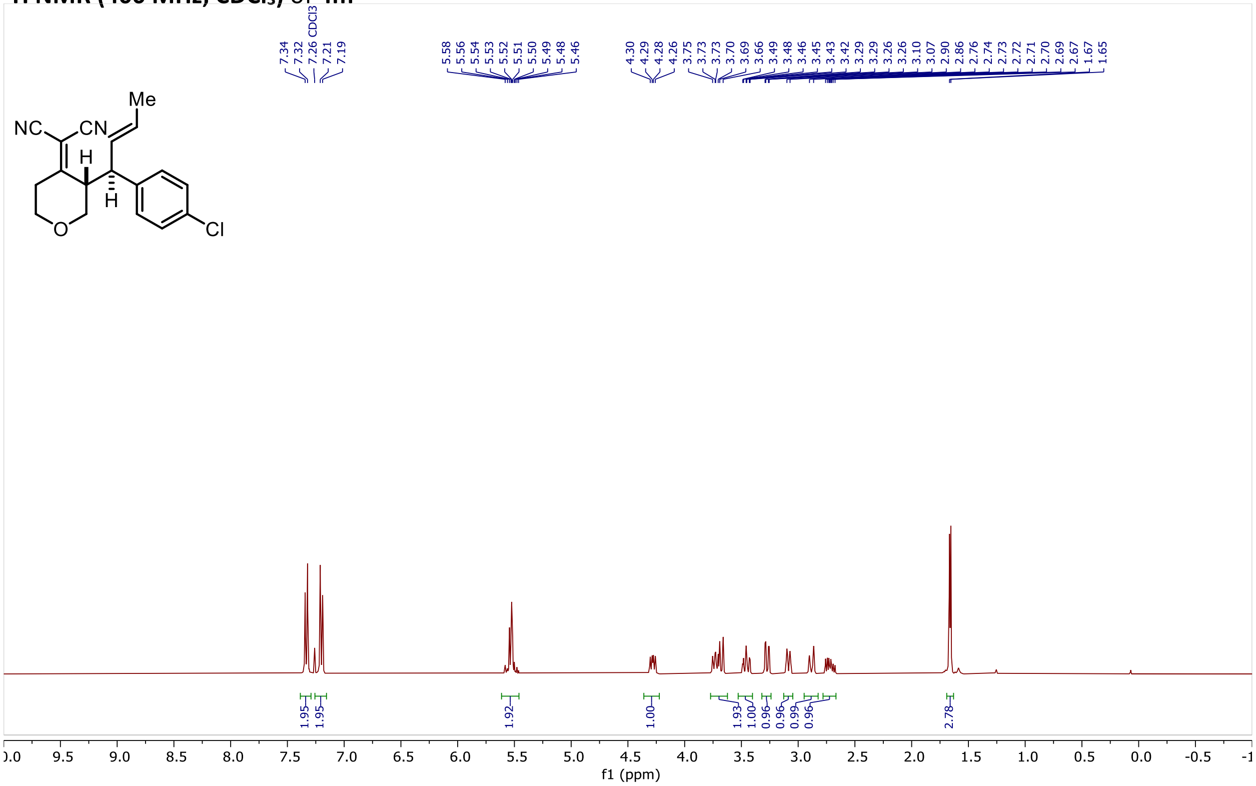

^{13}C NMR (101 MHz, CDCl$_3$) of **4m**

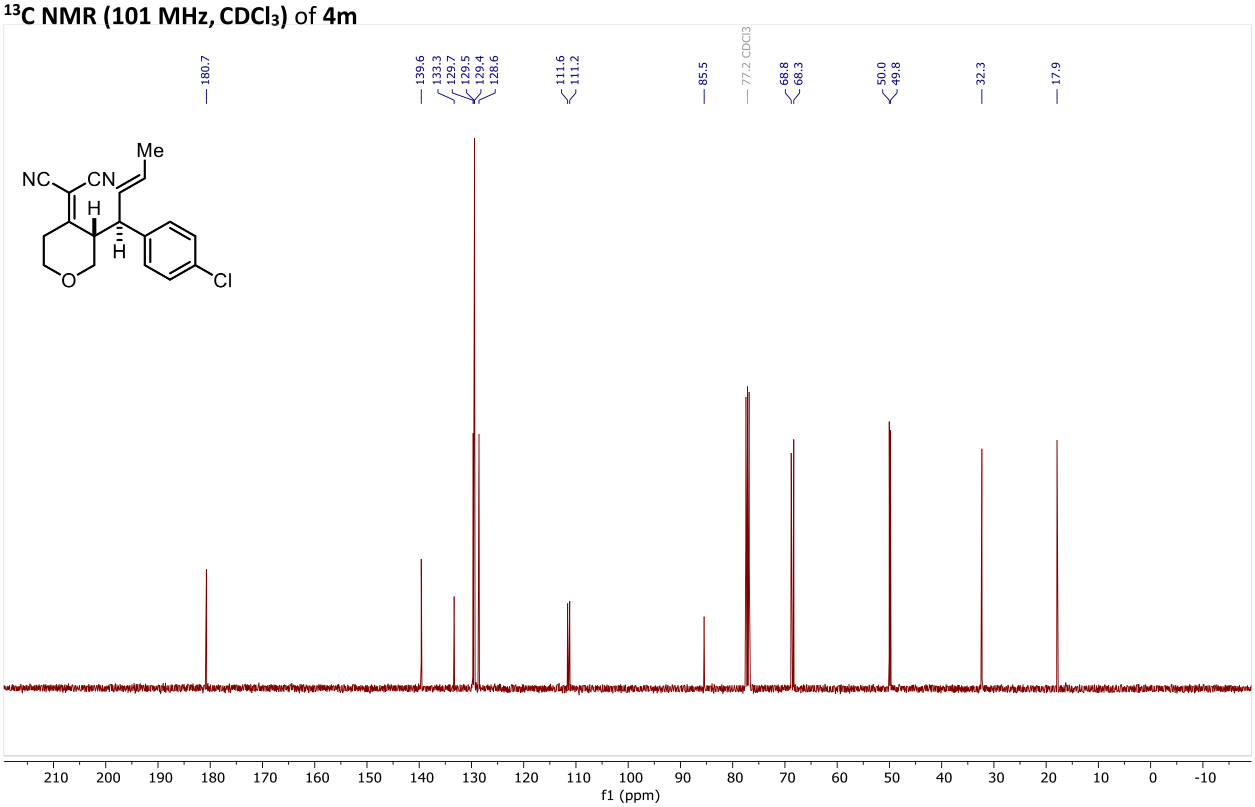

^{1}H NMR (400 MHz, CDCl$_3$) of 4n

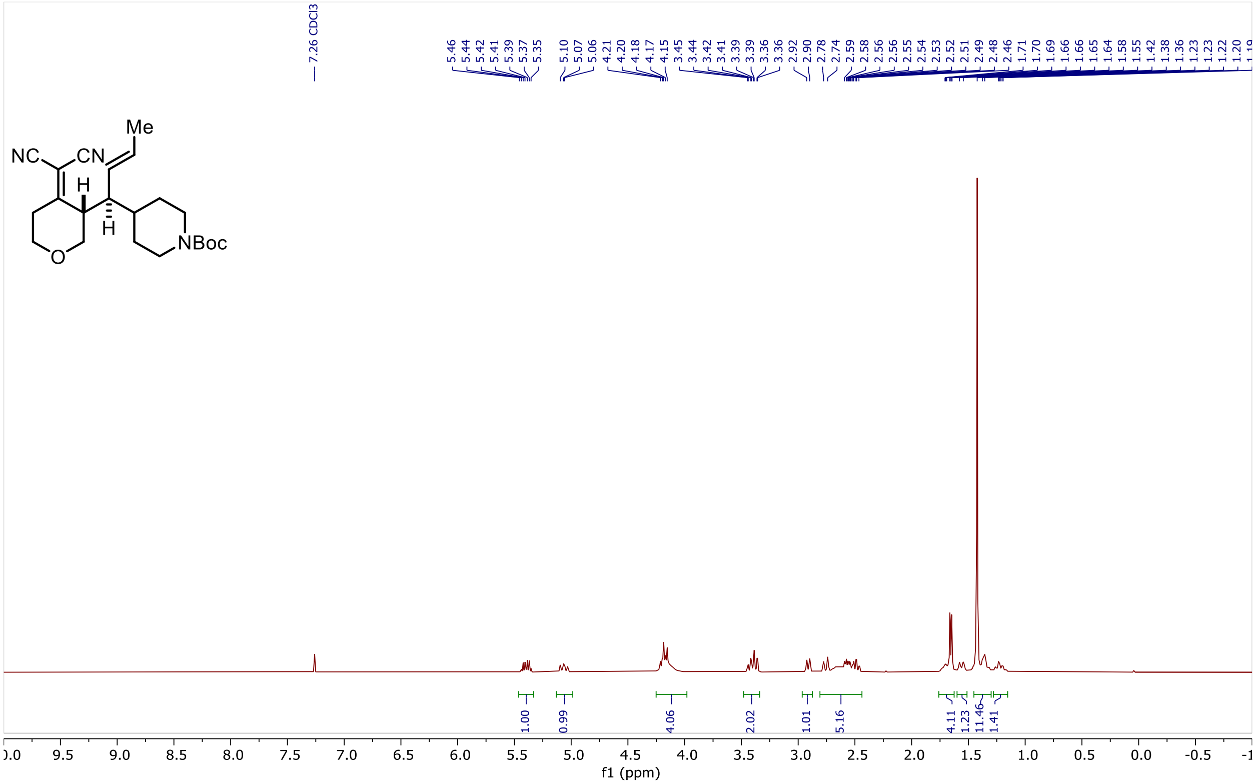

^{13}C NMR (101 MHz, CDCl$_3$) of 4n

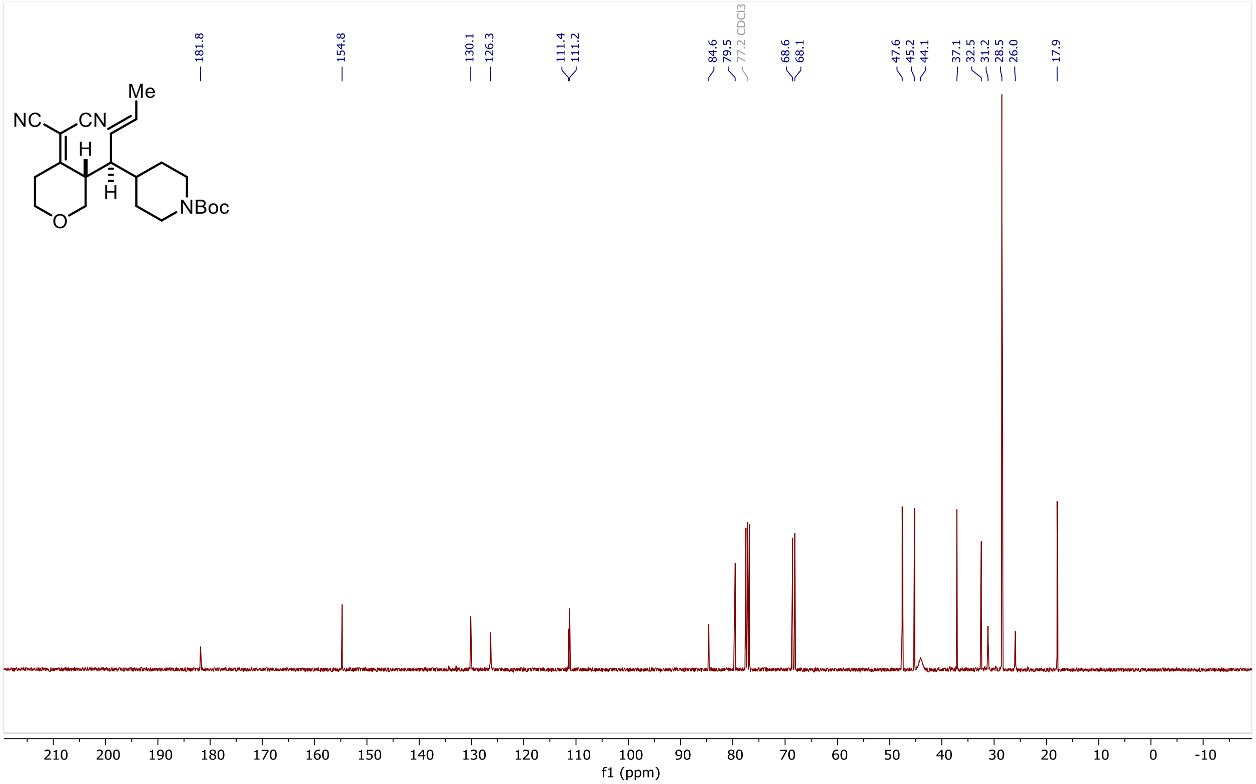

^{1}H NMR (400 MHz, CDCl$_3$) of **4o**

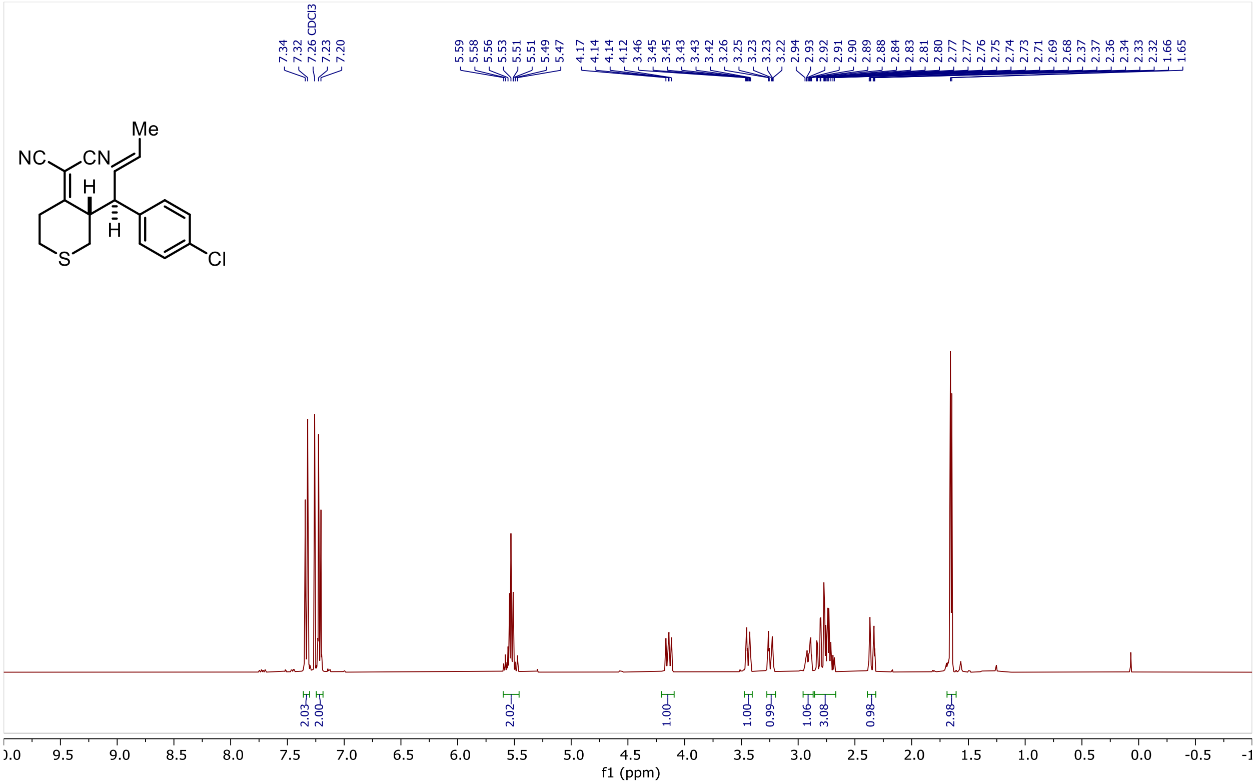

^{13}C NMR (101 MHz, CDCl$_3$) of **4o**

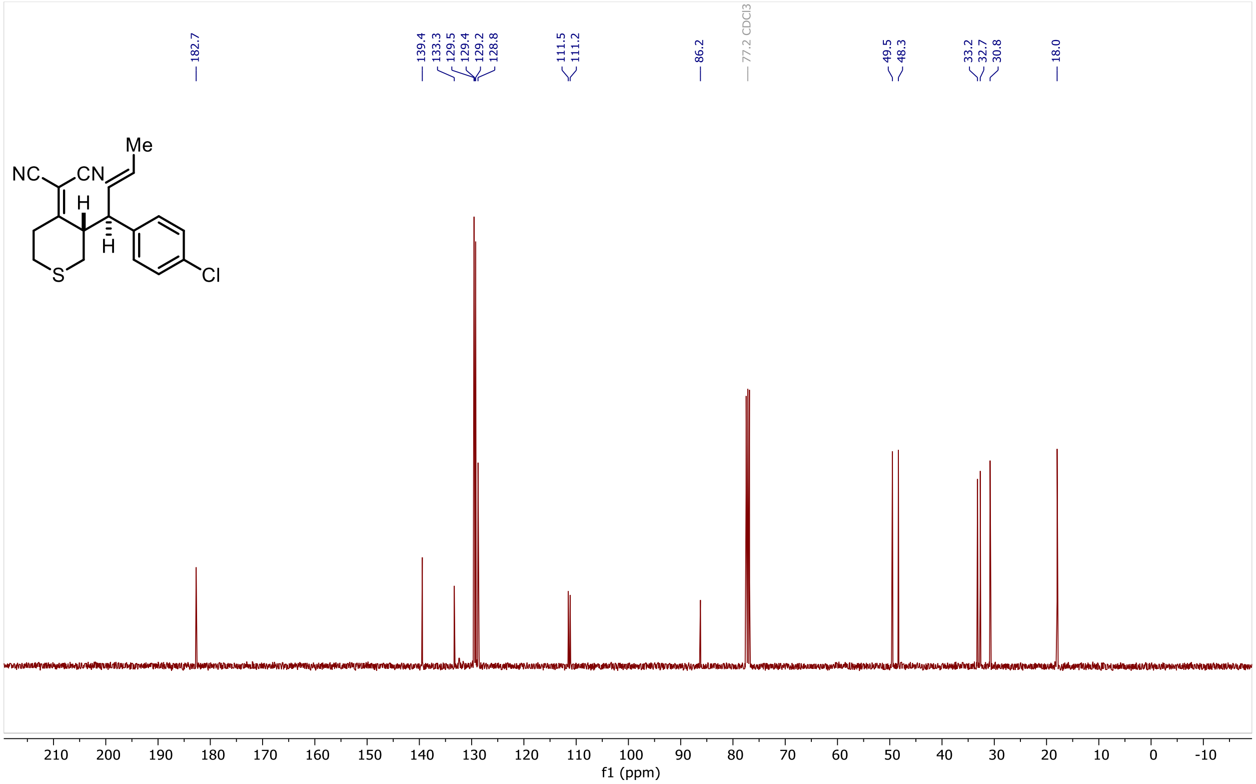

^{1}H NMR (400 MHz, CDCl$_3$) of **4p**

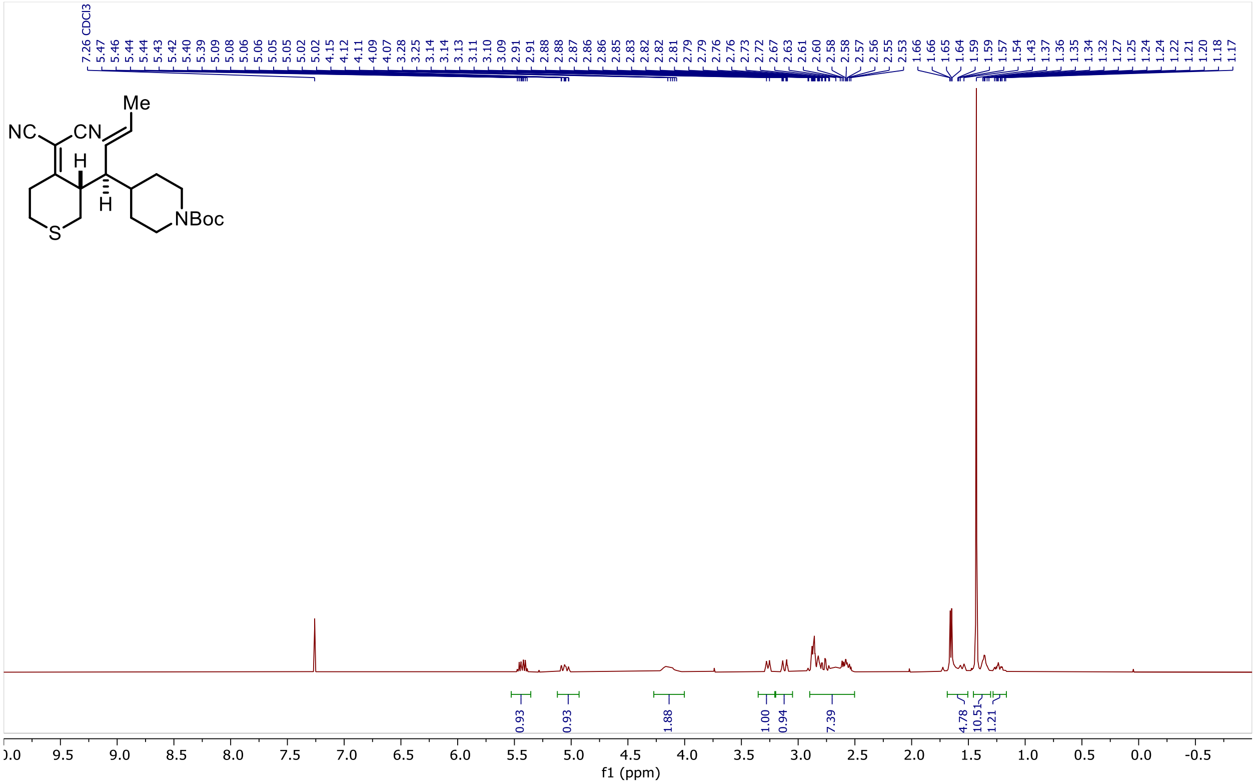

^{13}C NMR (101 MHz, CDCl$_3$) of **4p**

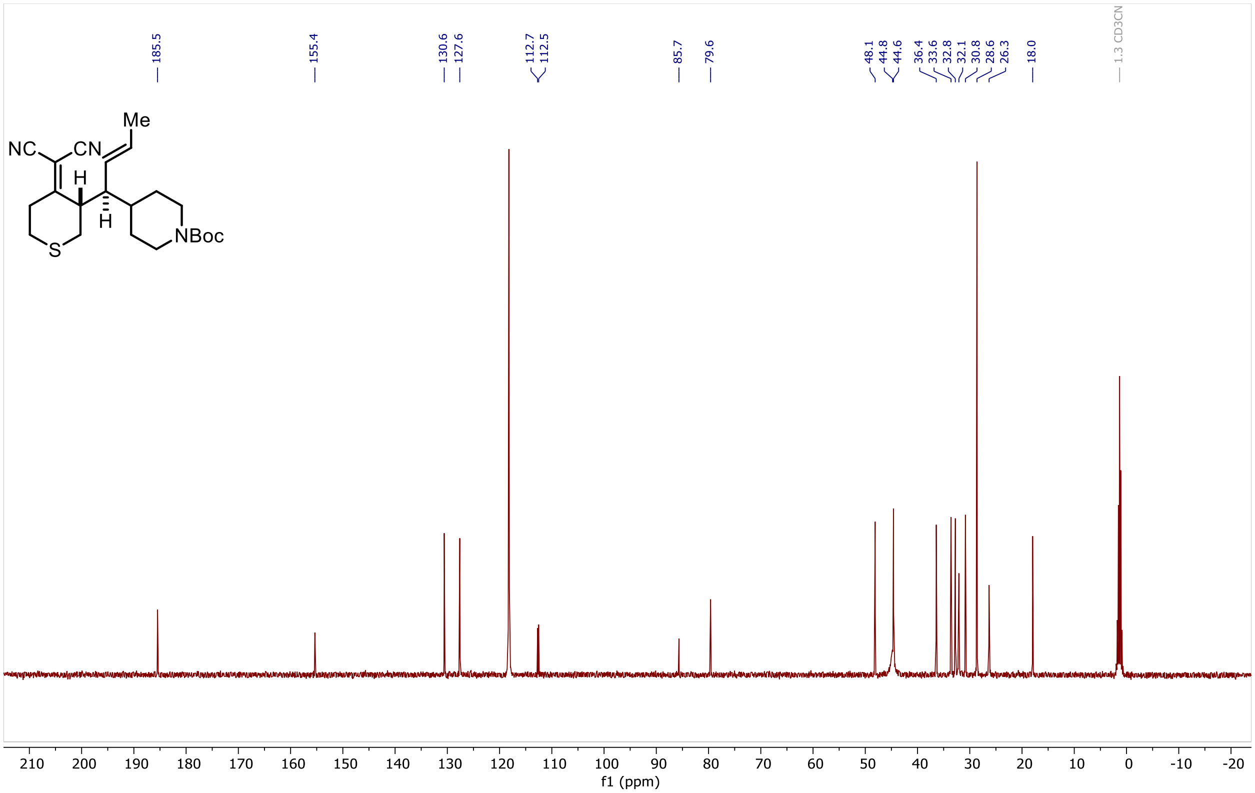

^{1}H NMR (400 MHz, CDCl$_3$) of 4q

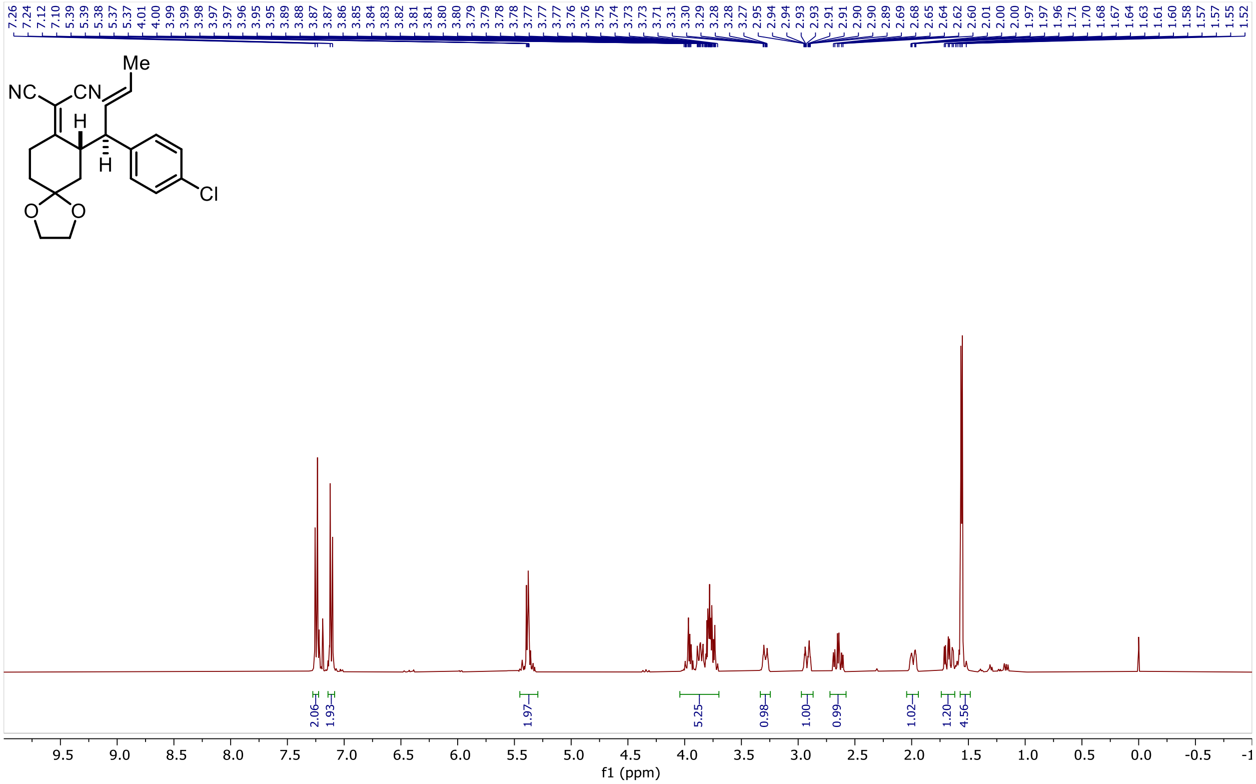

^{13}C NMR (101 MHz, CDCl$_3$) of 4q

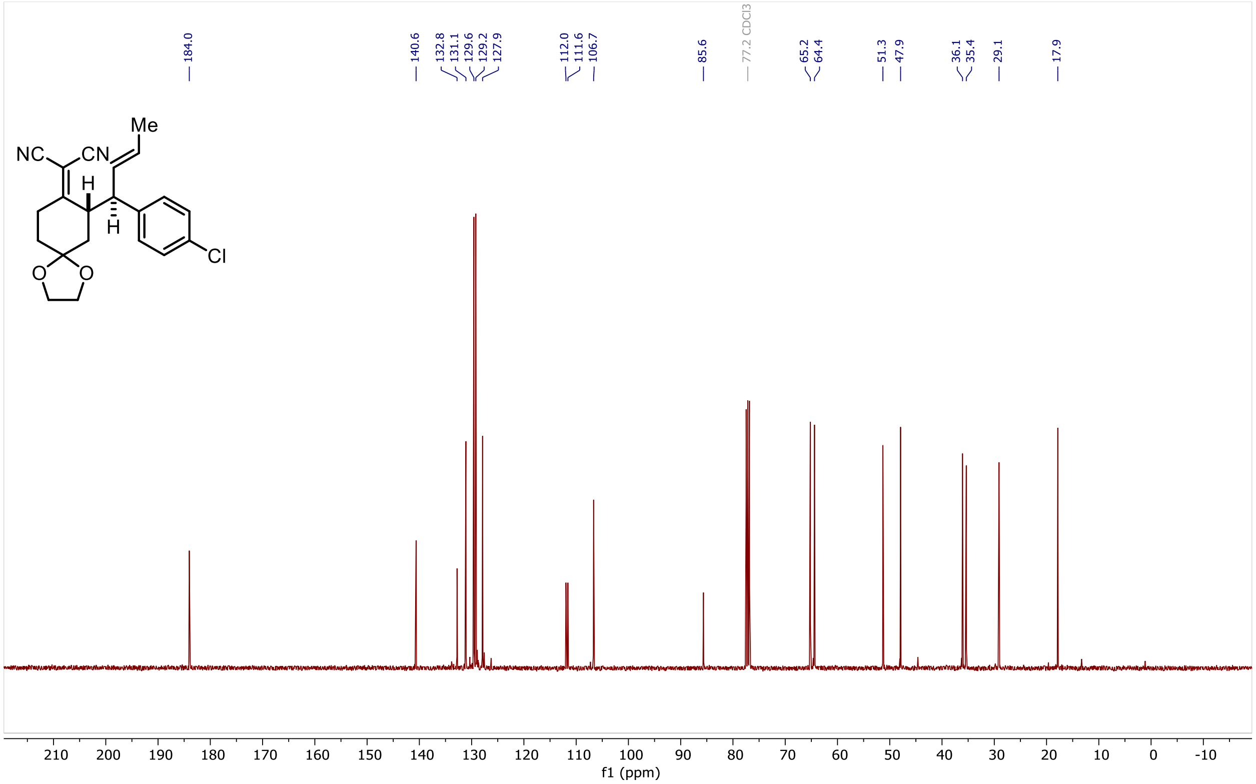

^{1}H NMR (400 MHz, CD_3CN) of 4r

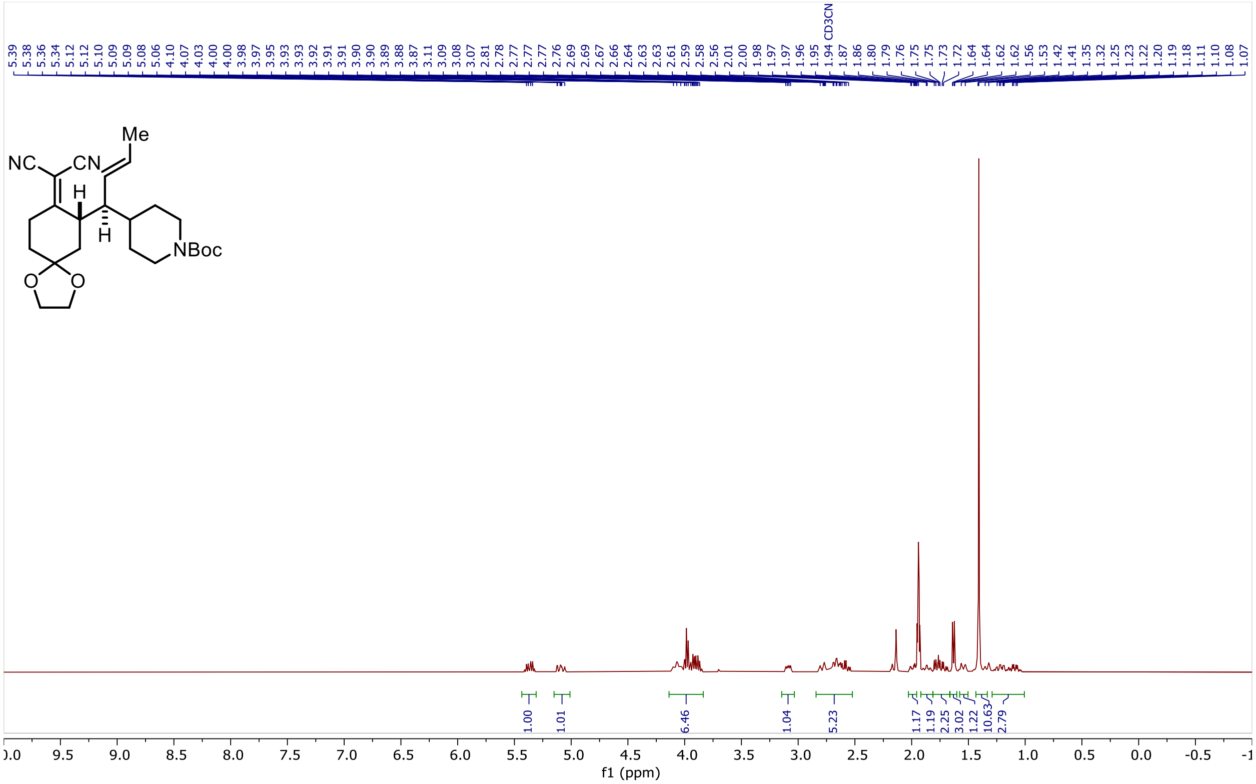

^{13}C NMR (101 MHz, CD_3CN) of 4r

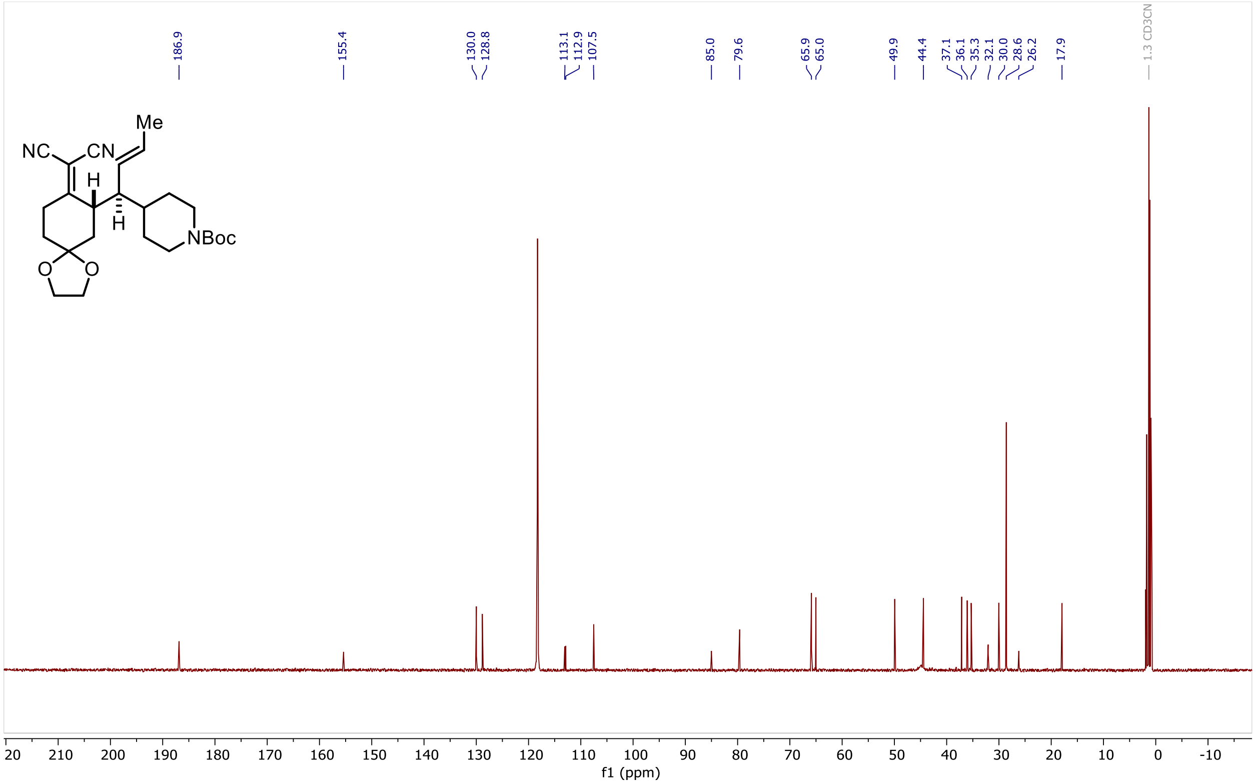

^{1}H NMR (400 MHz, CD_3CN) of 4u

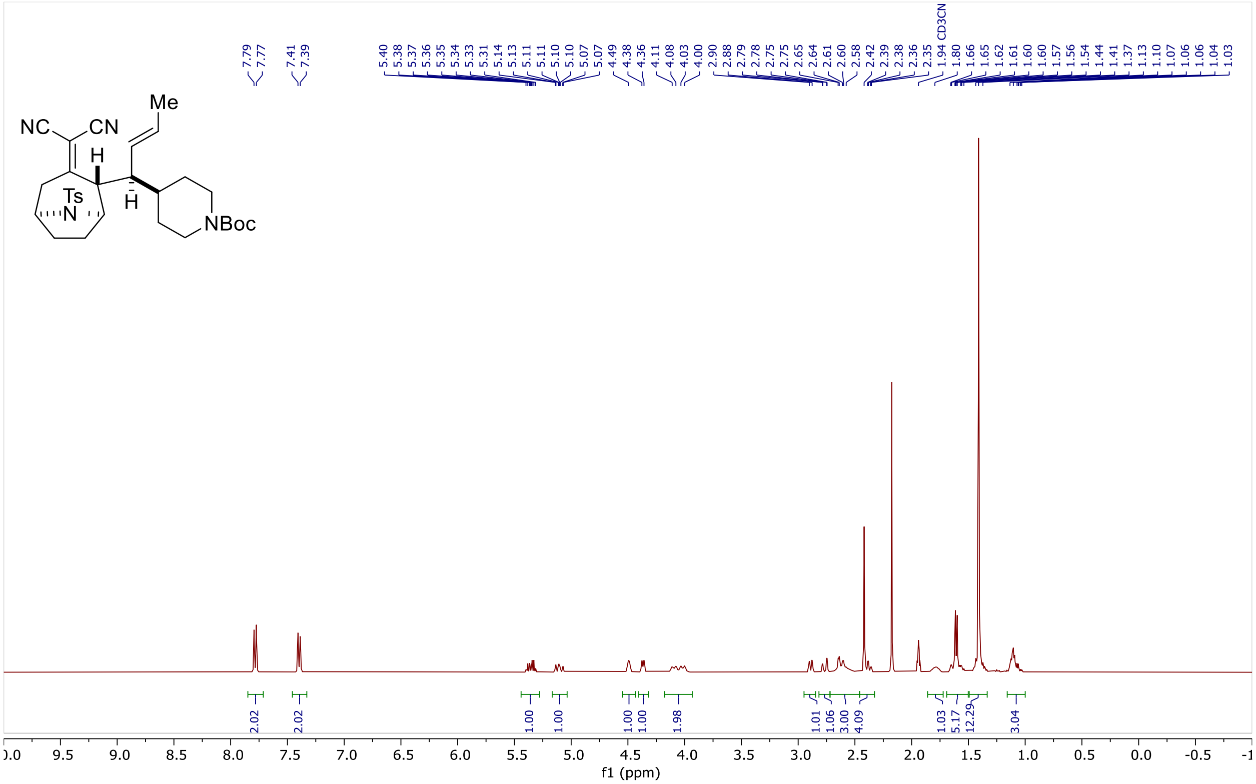

^{13}C NMR (101 MHz, CD_3CN) of 4u

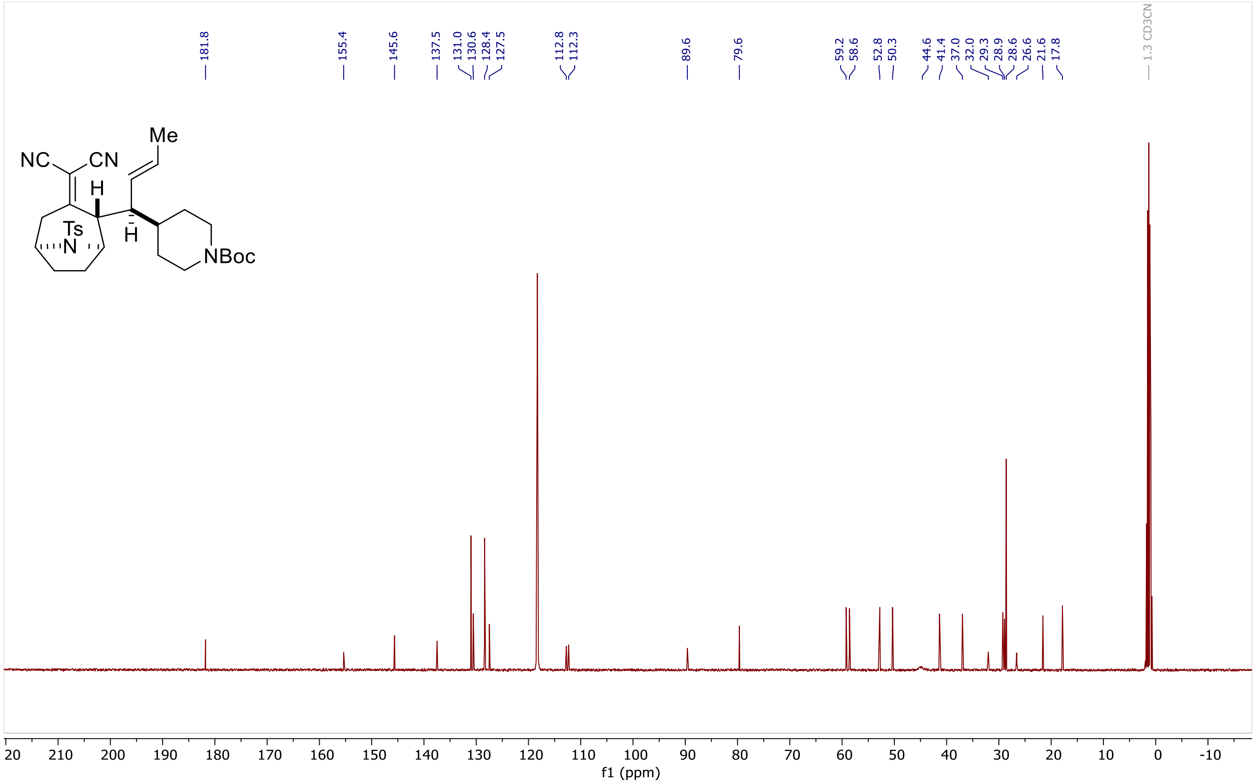

^{1}H NMR (400 MHz, CDCl$_3$) of 4v

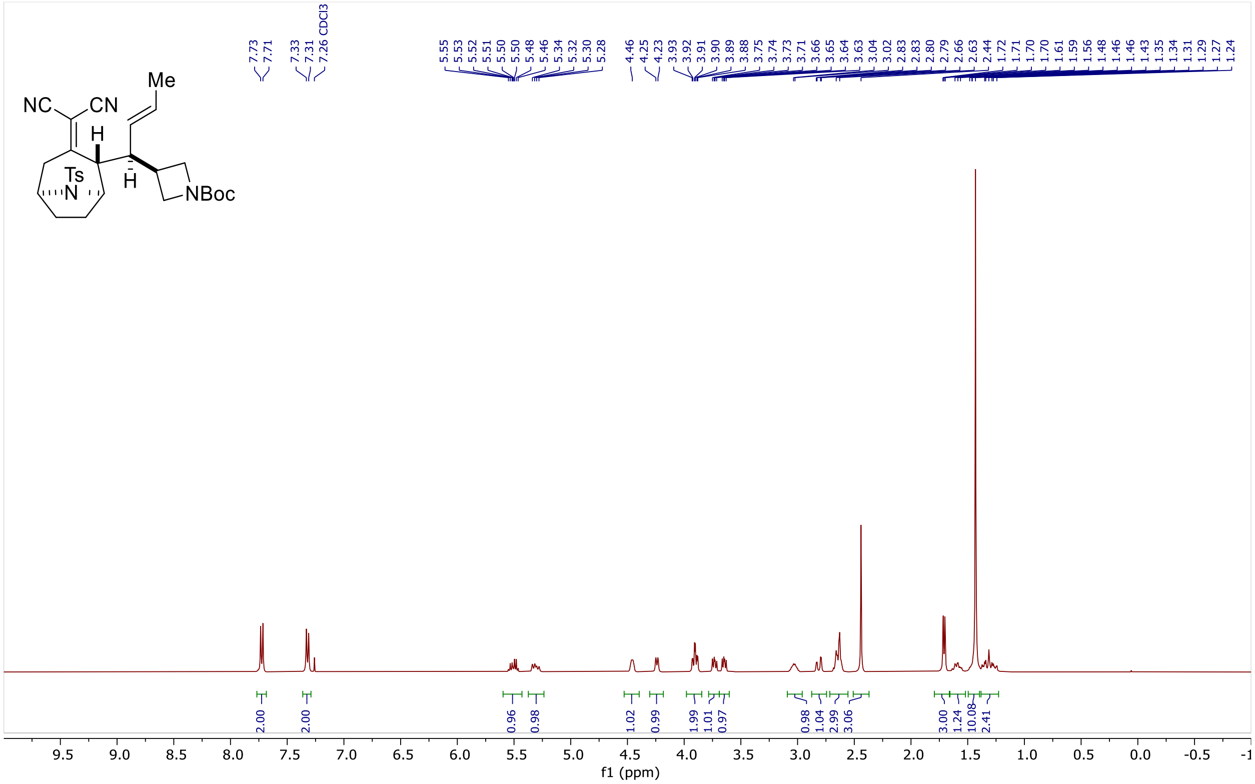

^{13}C NMR (101 MHz, CDCl$_3$) of 4v

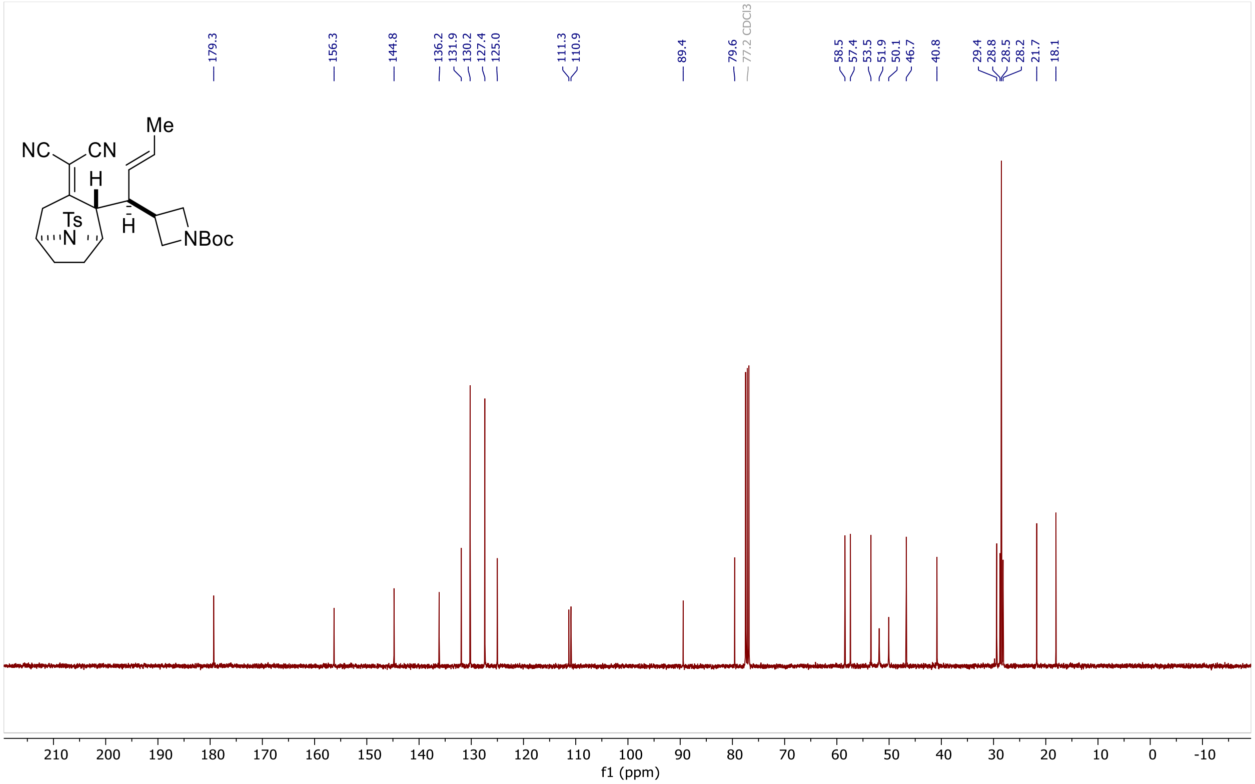

^{1}H NMR (400 MHz, CDCl$_3$) of **4w**

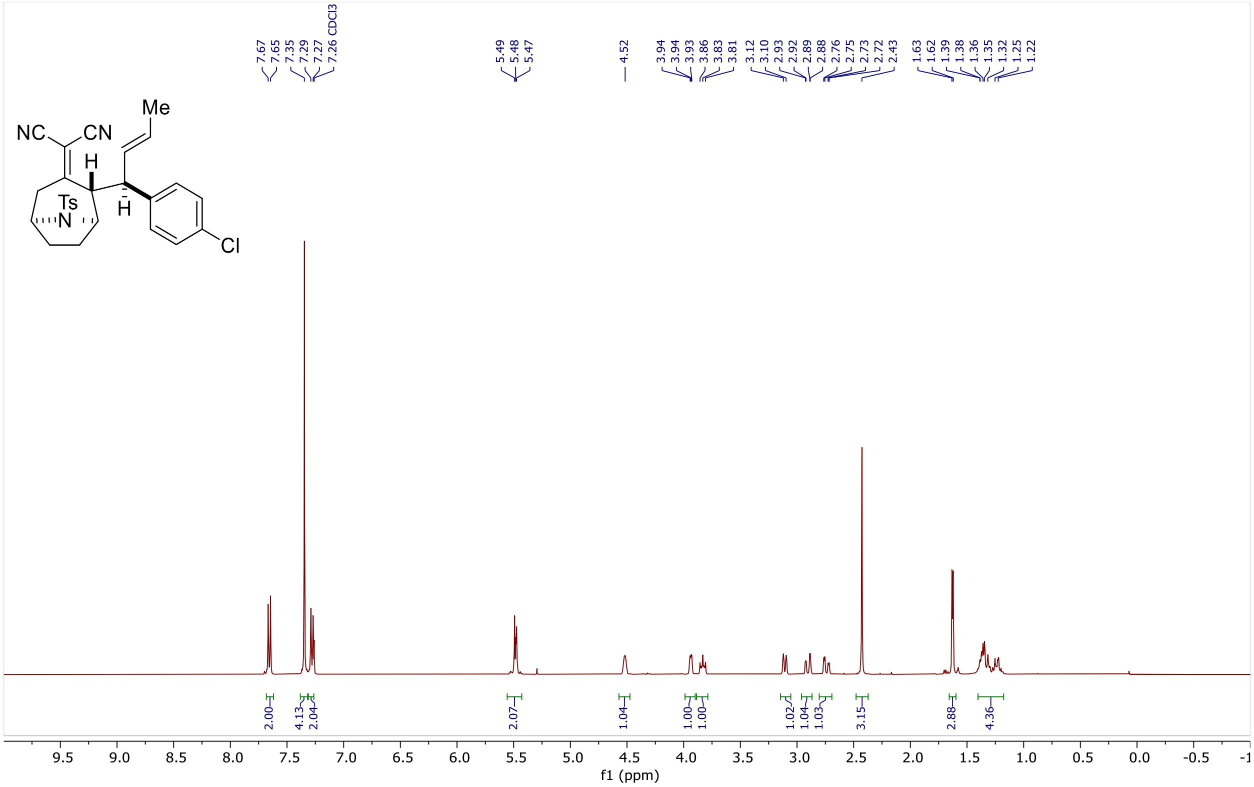

^{13}C NMR (101 MHz, CDCl$_3$) of **4w**

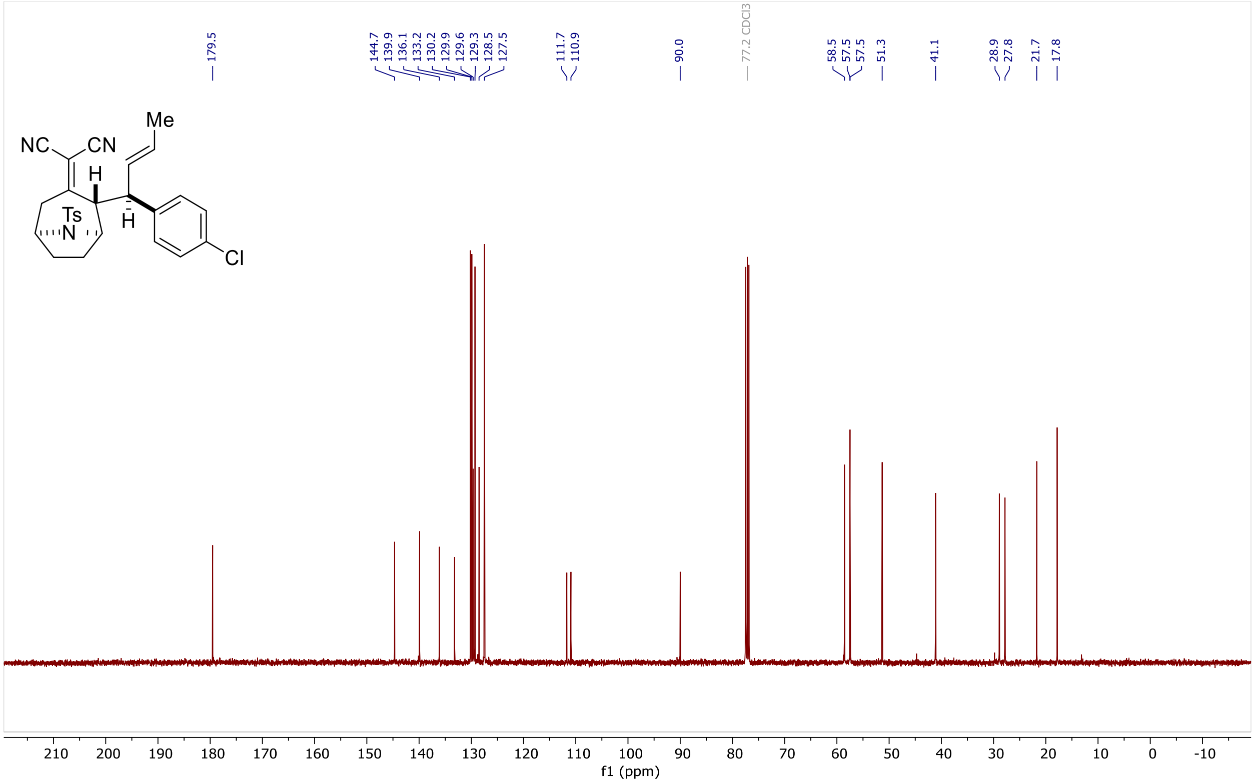

^{1}H NMR (400 MHz, CDCl$_3$) of 4x

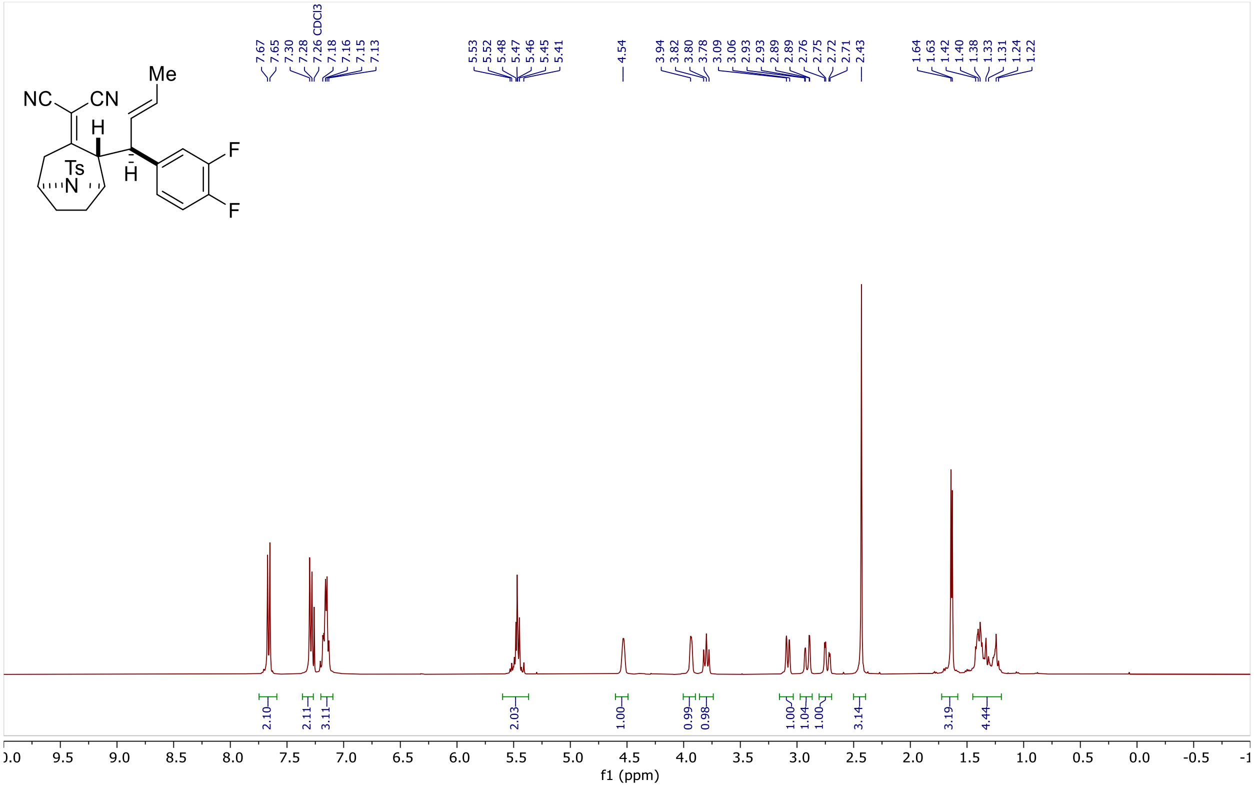

^{13}C NMR (101 MHz, CDCl$_3$) of 4x

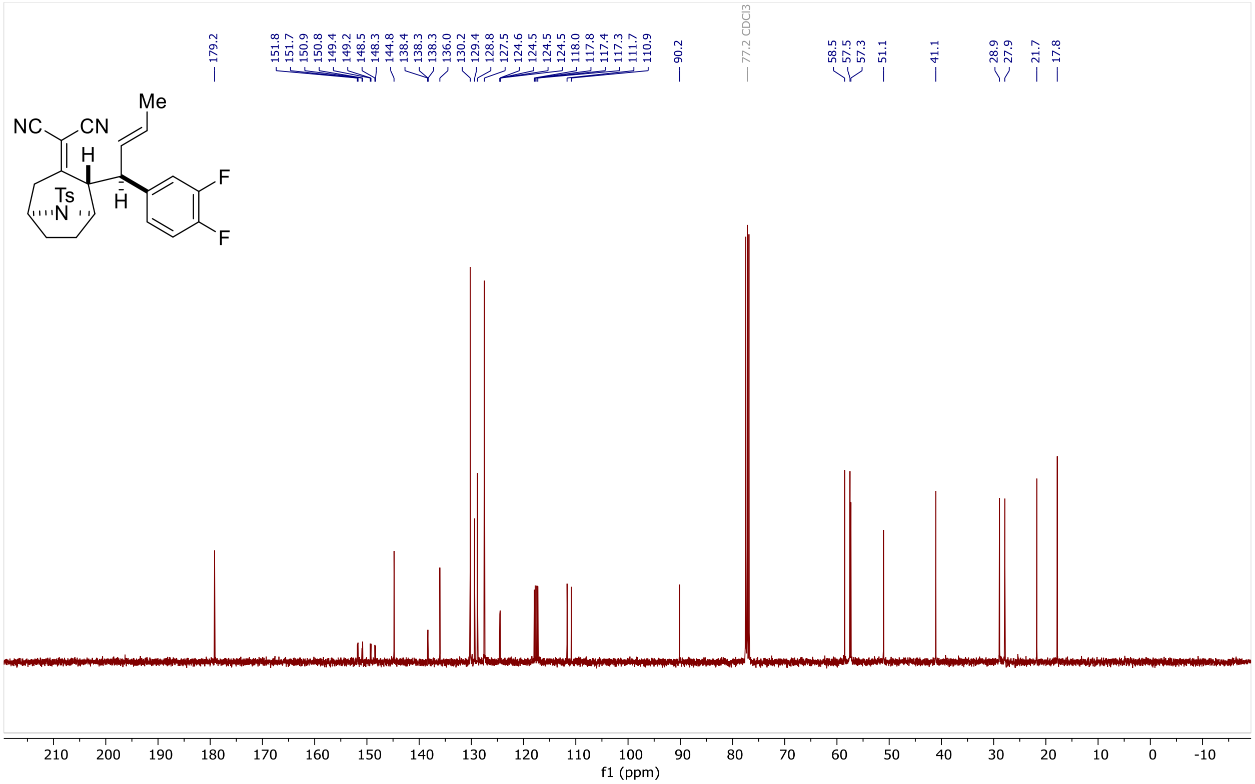

^{19}F NMR (377 MHz, CDCl$_3$) of **4x**

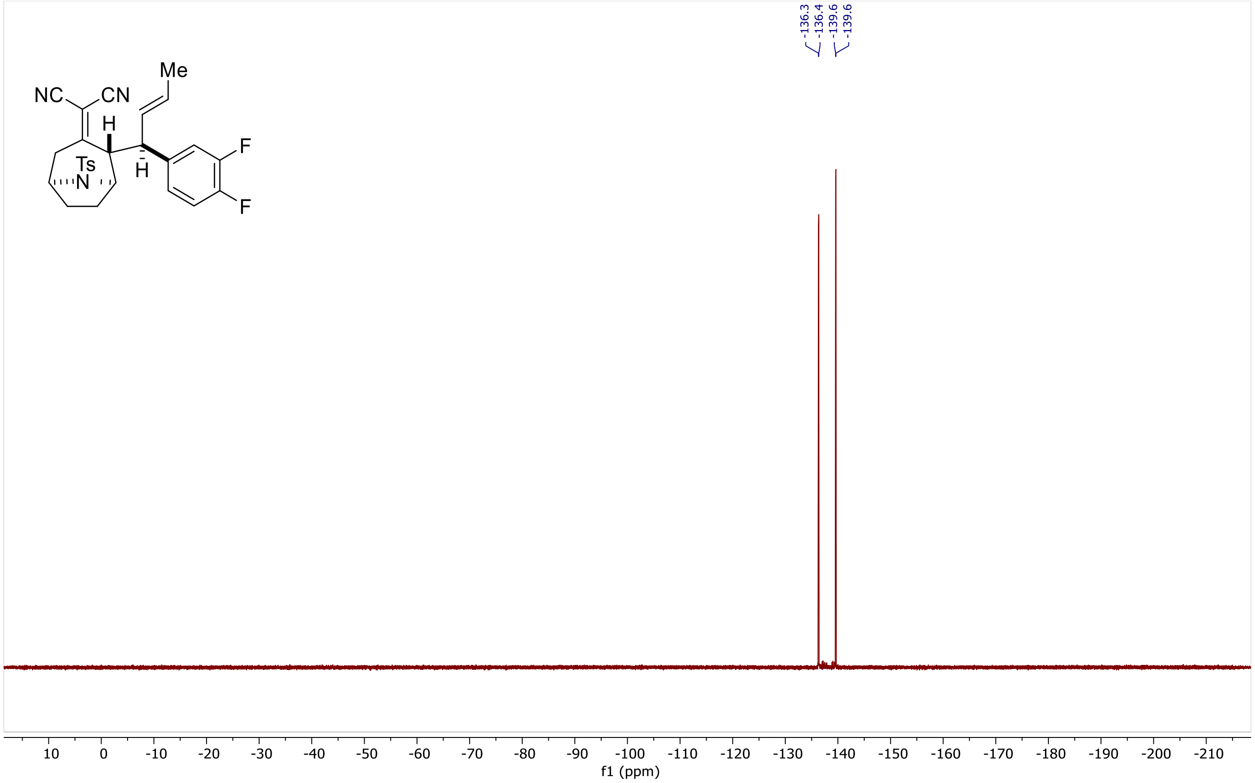

^{1}H NMR (600 MHz, CDCl$_3$) of **4y**

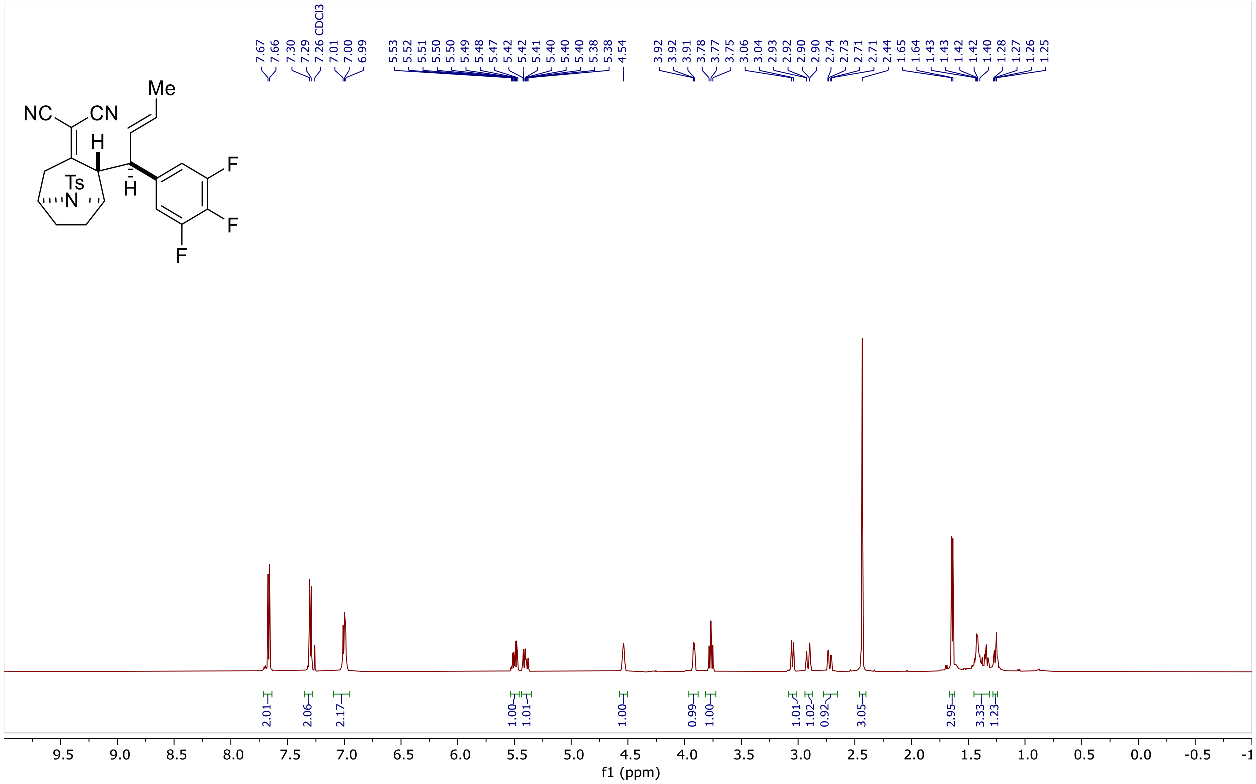

^{13}C NMR (151 MHz, $CDCl_3$) of **4y**

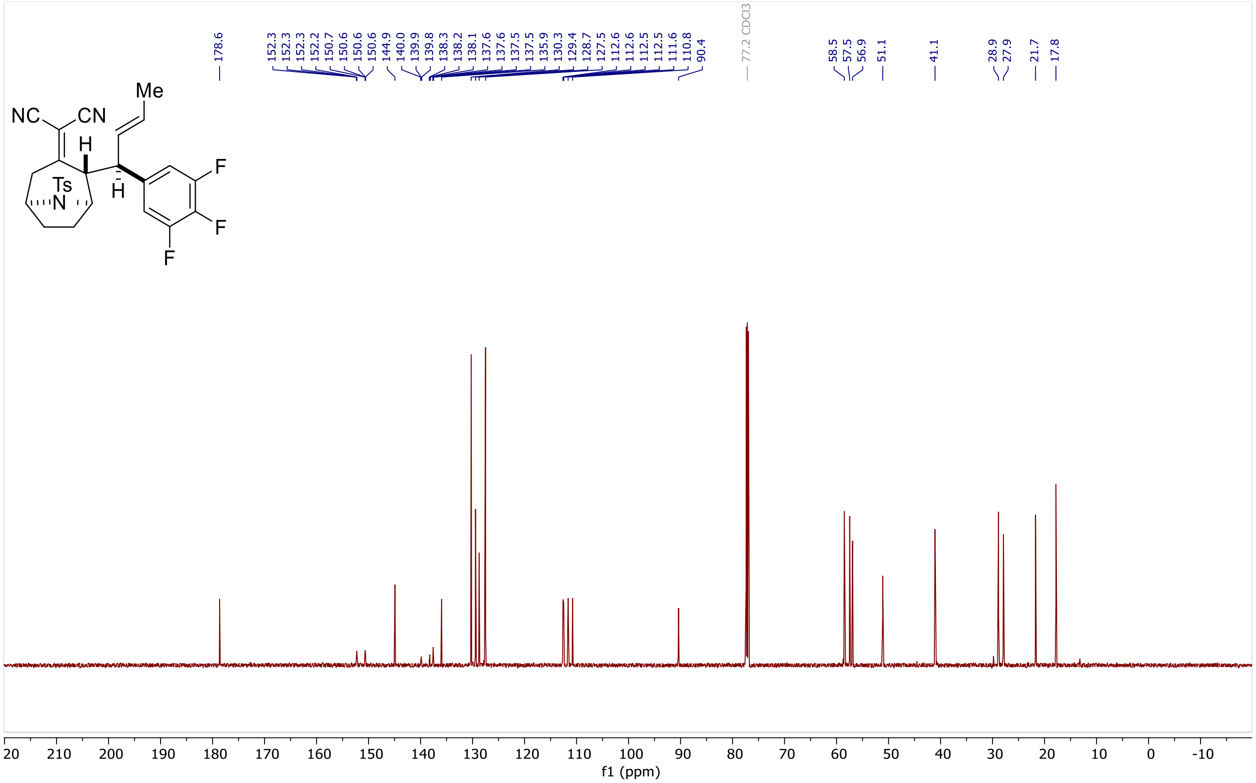

^{19}F NMR (377 MHz, $CDCl_3$) of **4y**

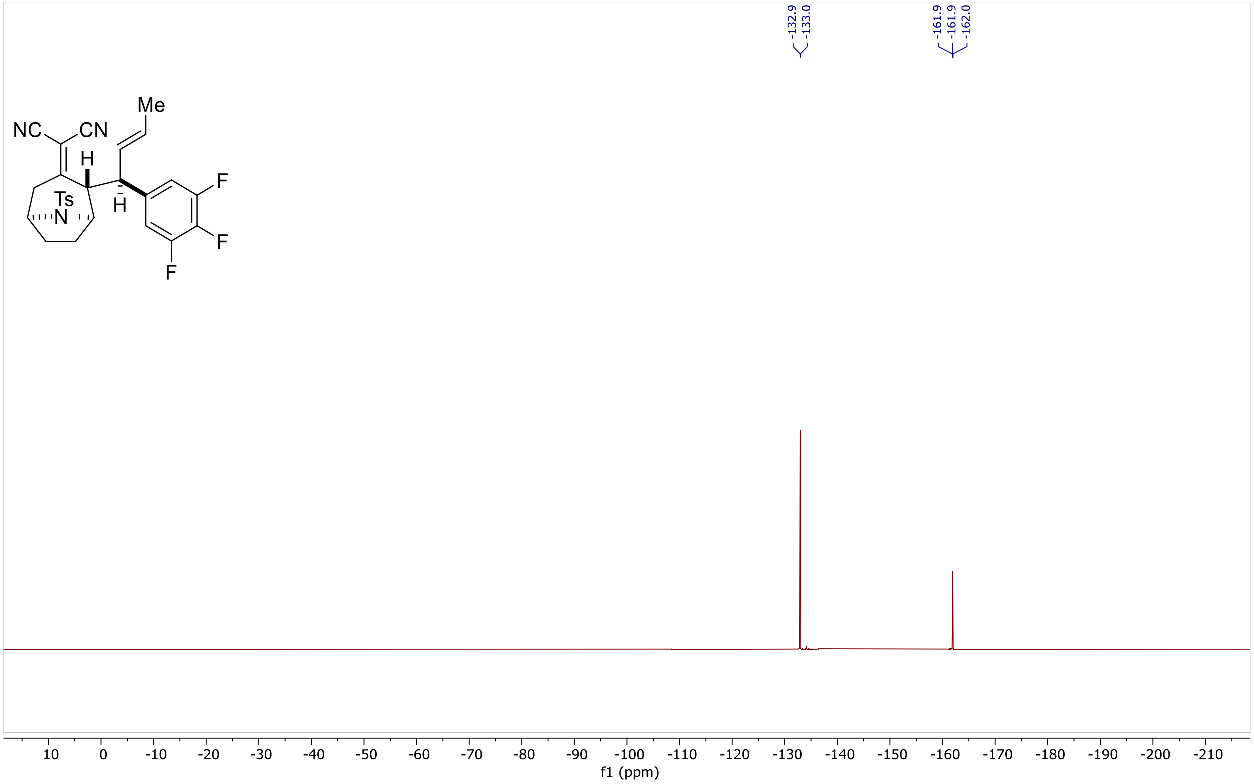

^{1}H NMR (400 MHz, CDCl$_3$) of **4z**

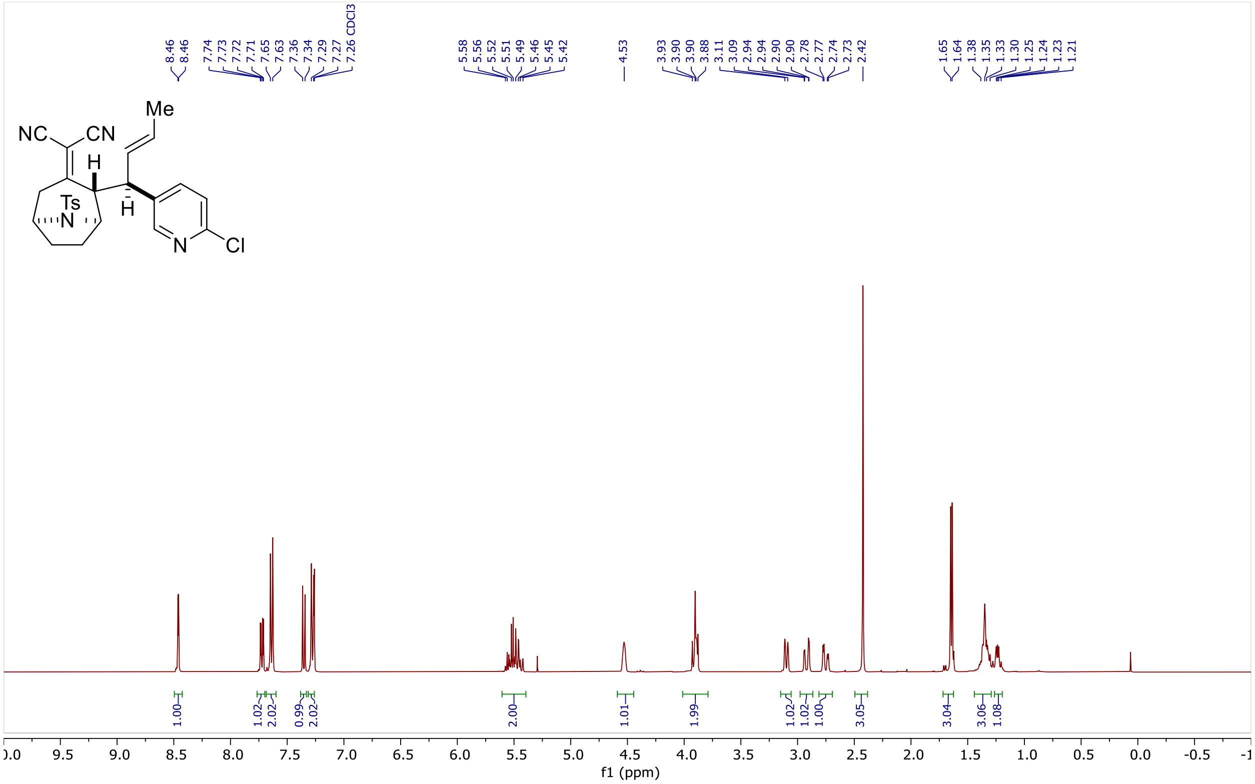

^{13}C NMR (101 MHz, CDCl$_3$) of **4z**

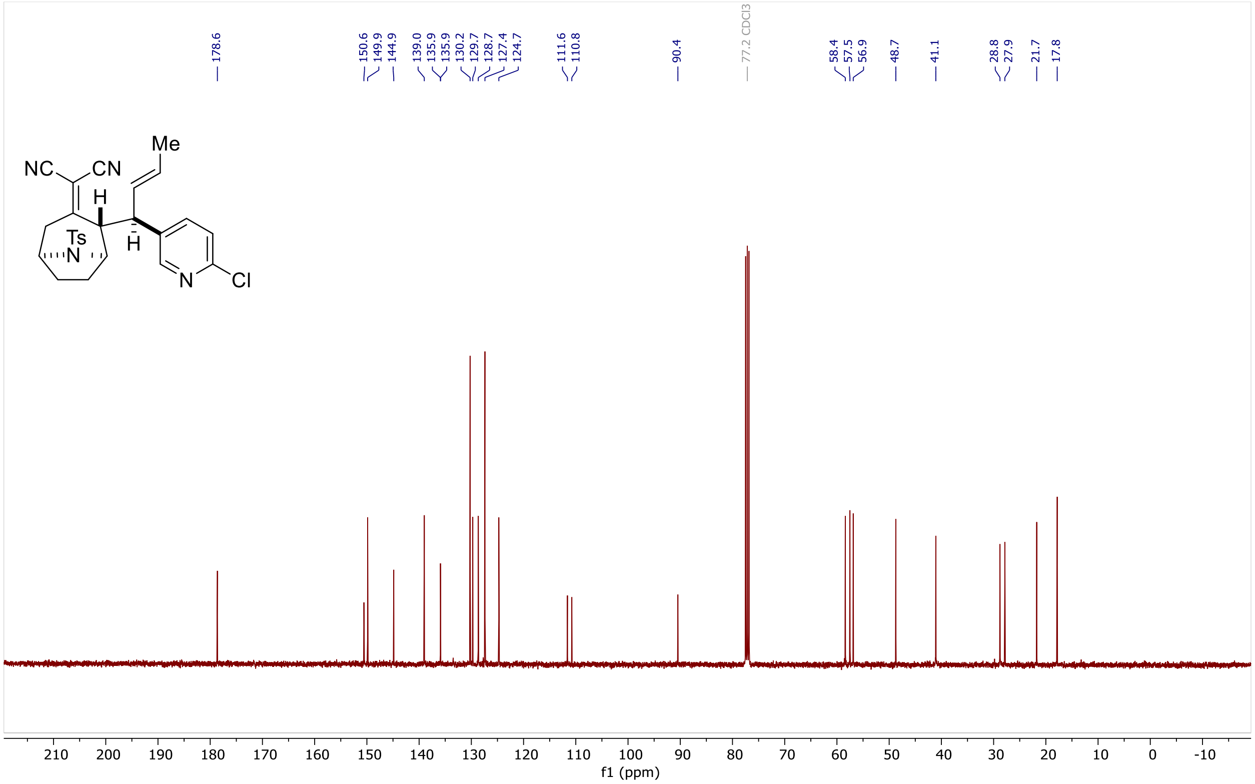

^{1}H NMR (400 MHz, CDCl₃) of 4aa

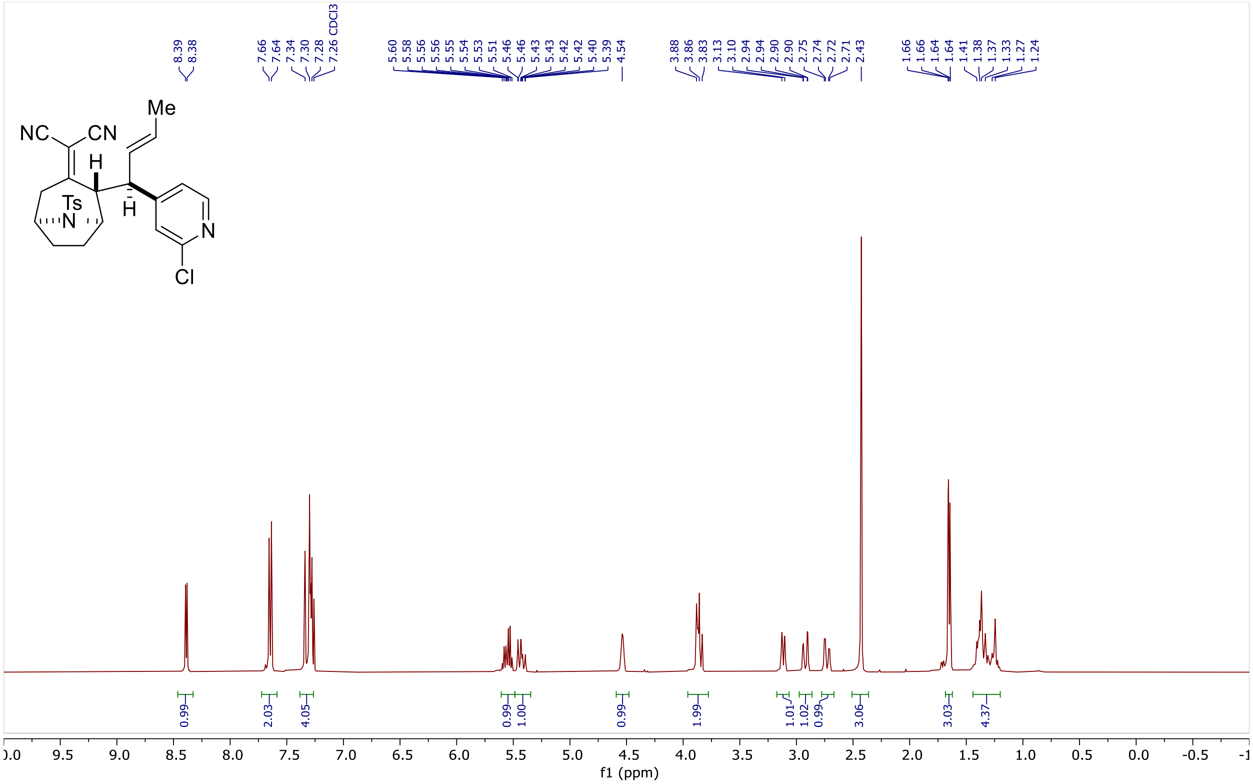

^{13}C NMR (101 MHz, CDCl₃) of 4aa

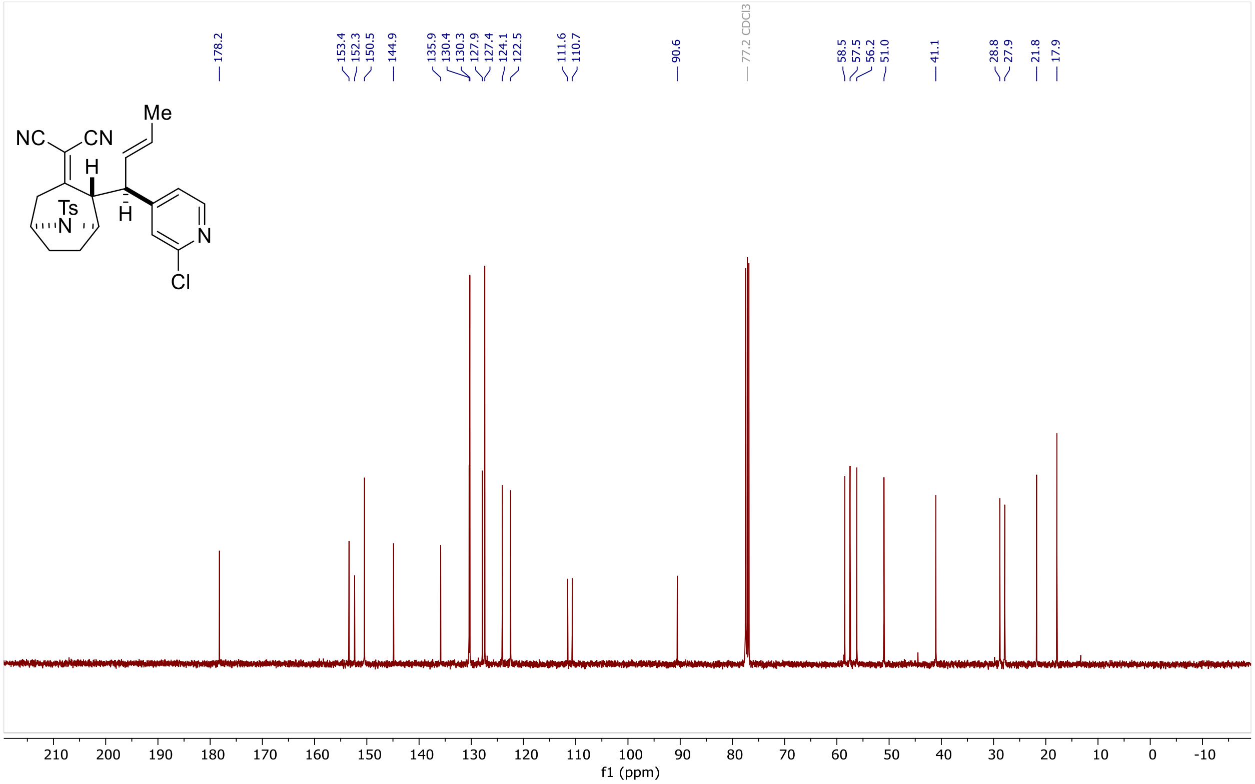

^{1}H NMR (400 MHz, $CDCl_3$) of 4bb

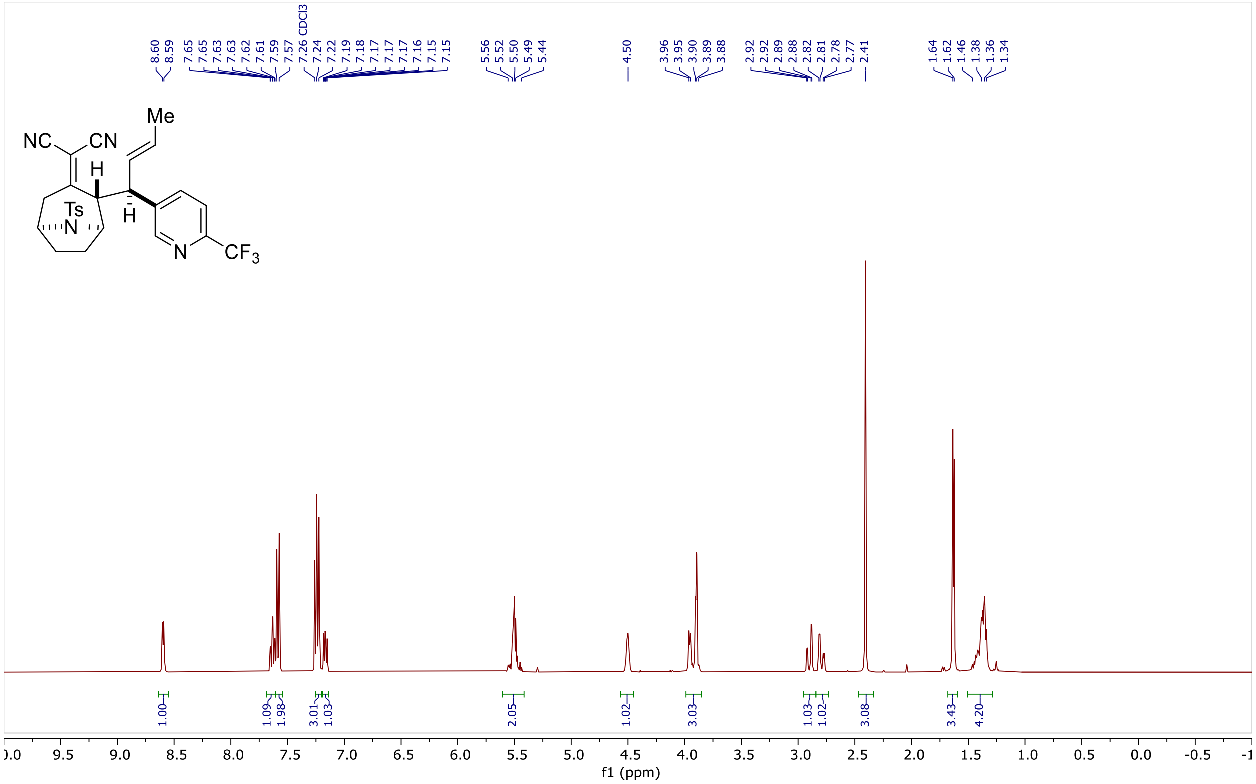

^{13}C NMR (101 MHz, $CDCl_3$) of 4bb

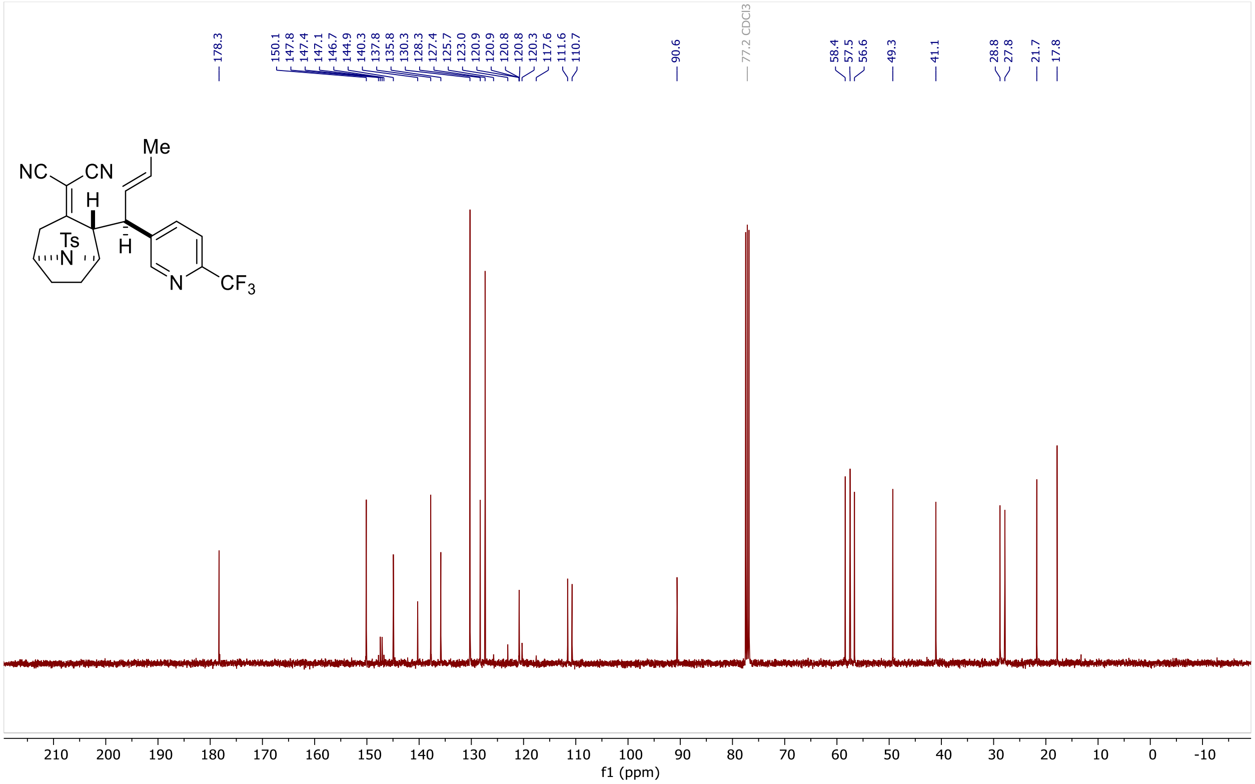

^{19}F NMR (377 MHz, CDCl$_3$) of **4bb**

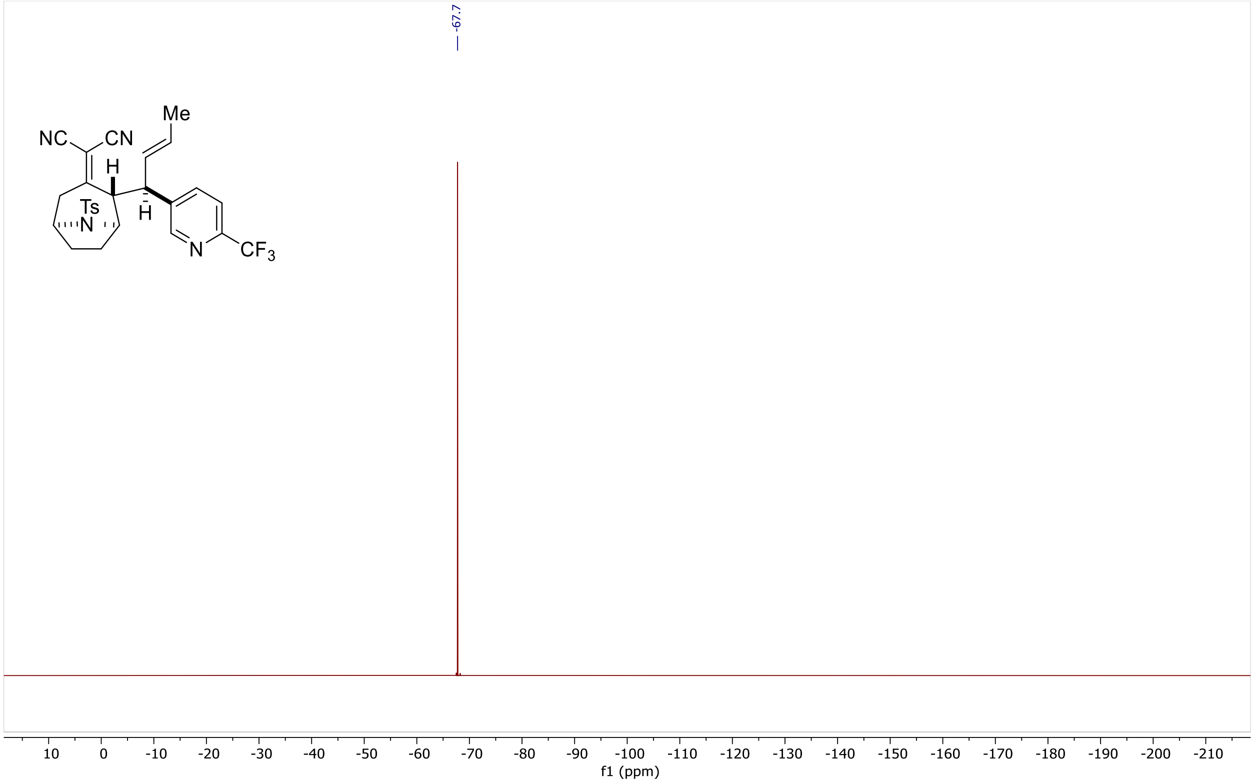

^{1}H NMR (400 MHz, CDCl$_3$) of **4cc**

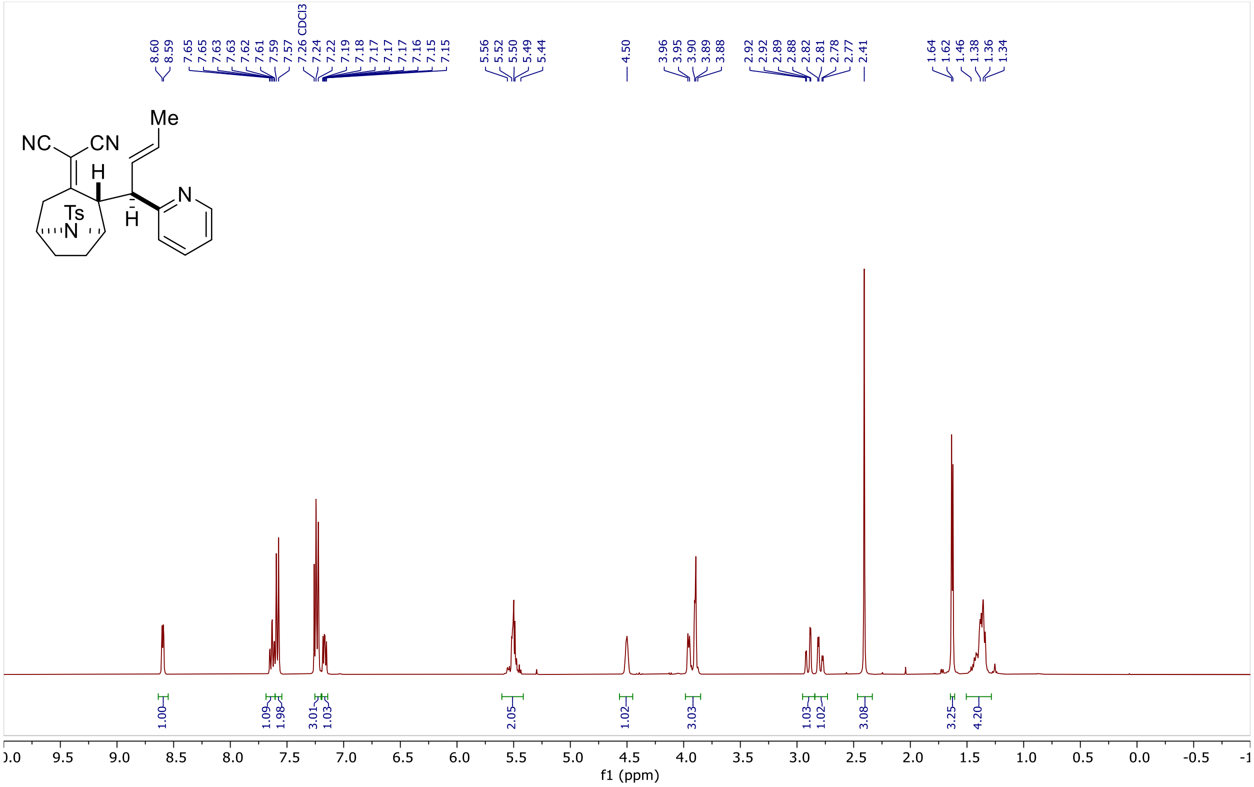

^{13}C NMR (101 MHz, CDCl$_3$) of 4cc

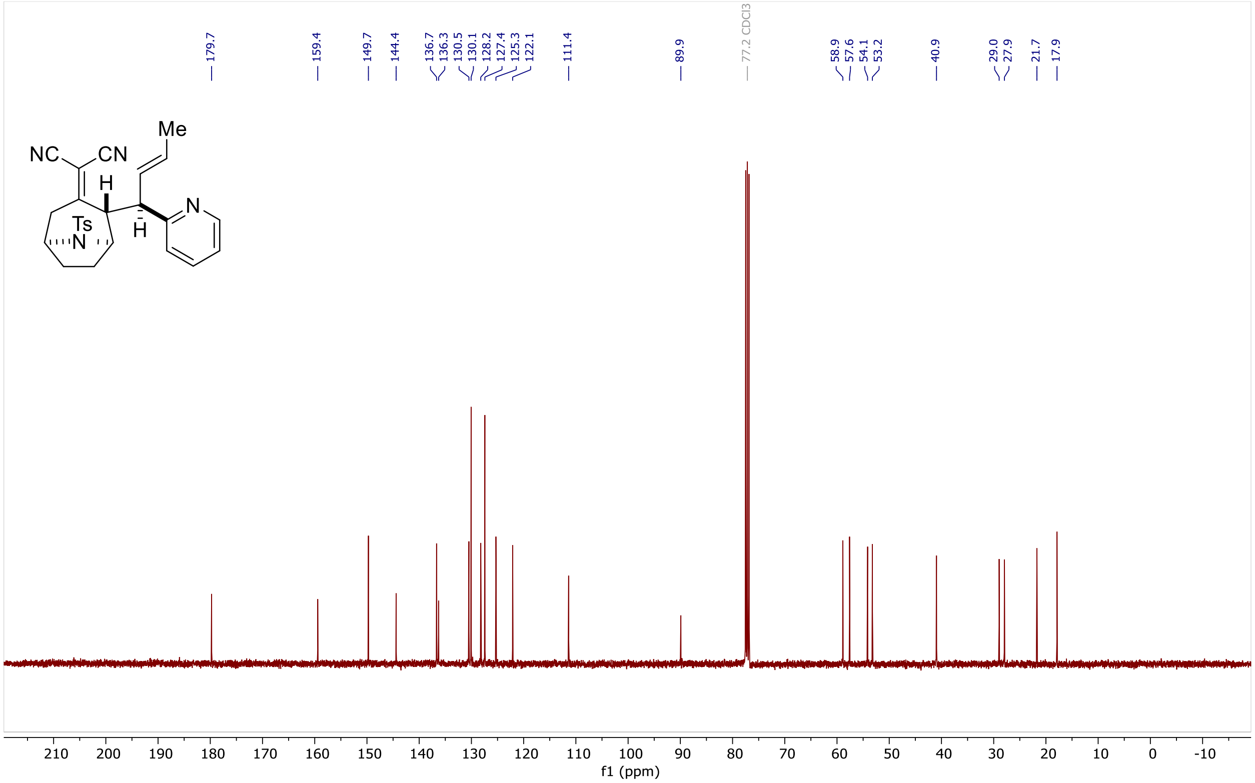

^{1}H NMR (400 MHz, DMSO-d_6, 354 K) of 4dd

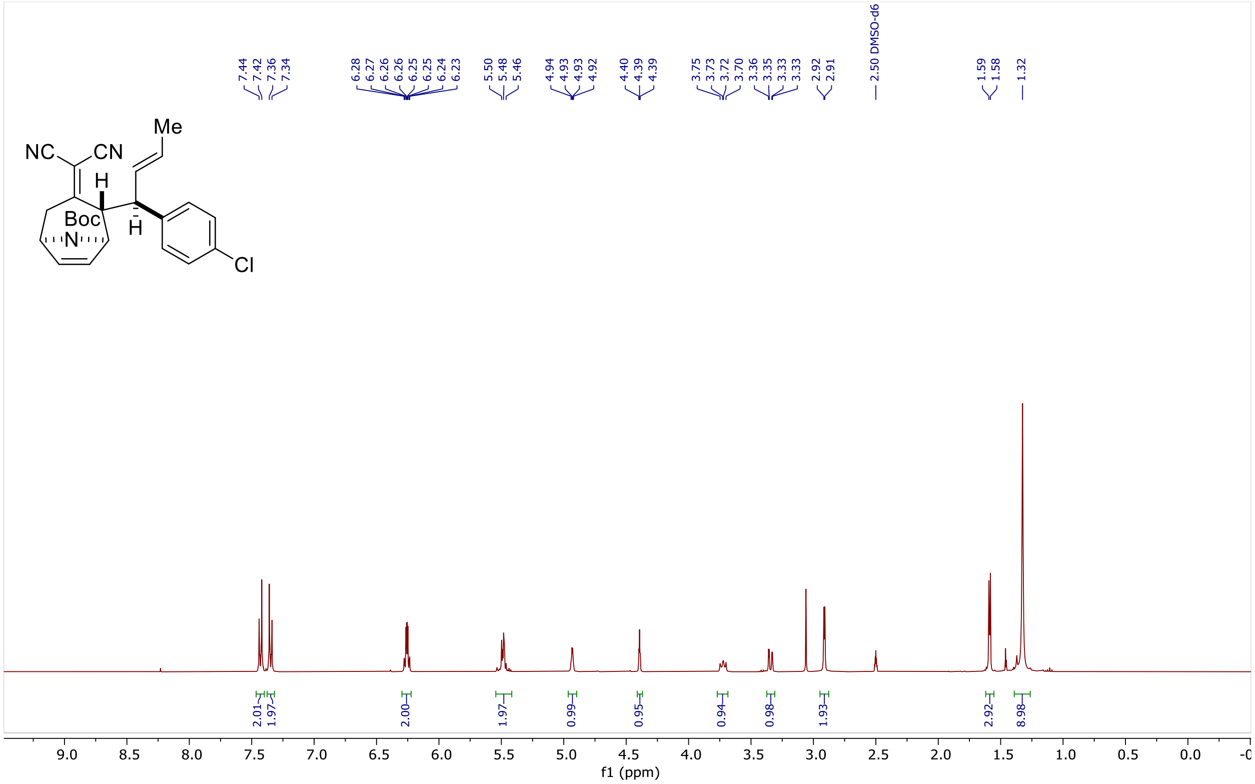

^{13}C NMR (101 MHz, DMSO-*d_6*, 354 K) of **4dd**

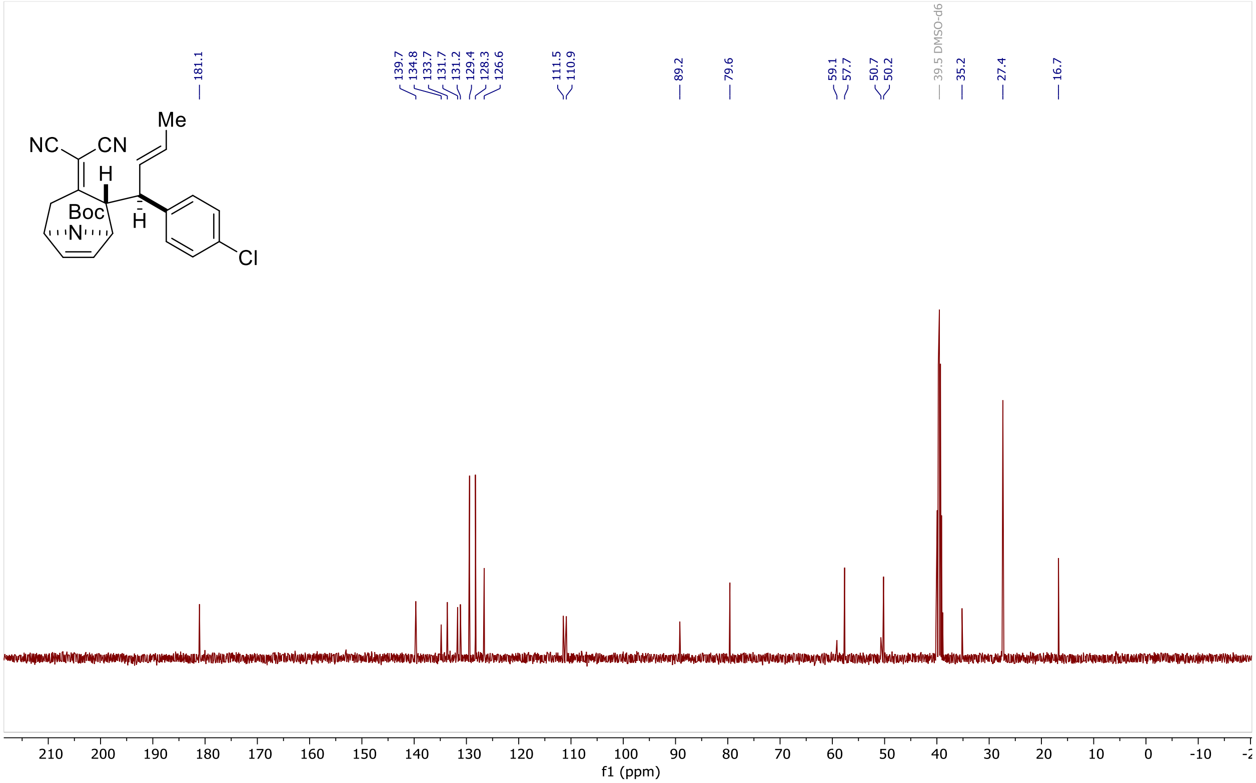

^{1}H NMR (600 MHz, CDCl$_3$) of **4ee**

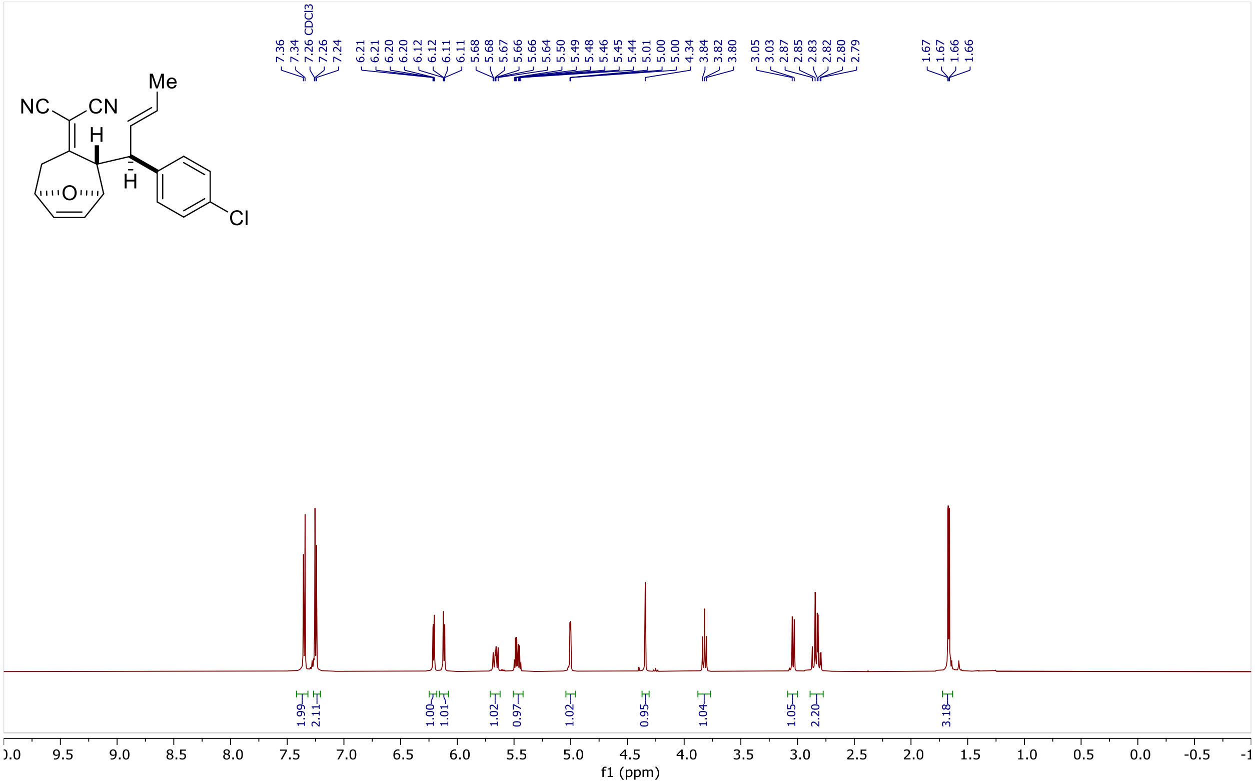

^{13}C NMR (101 MHz, CDCl$_3$) of **4ee**

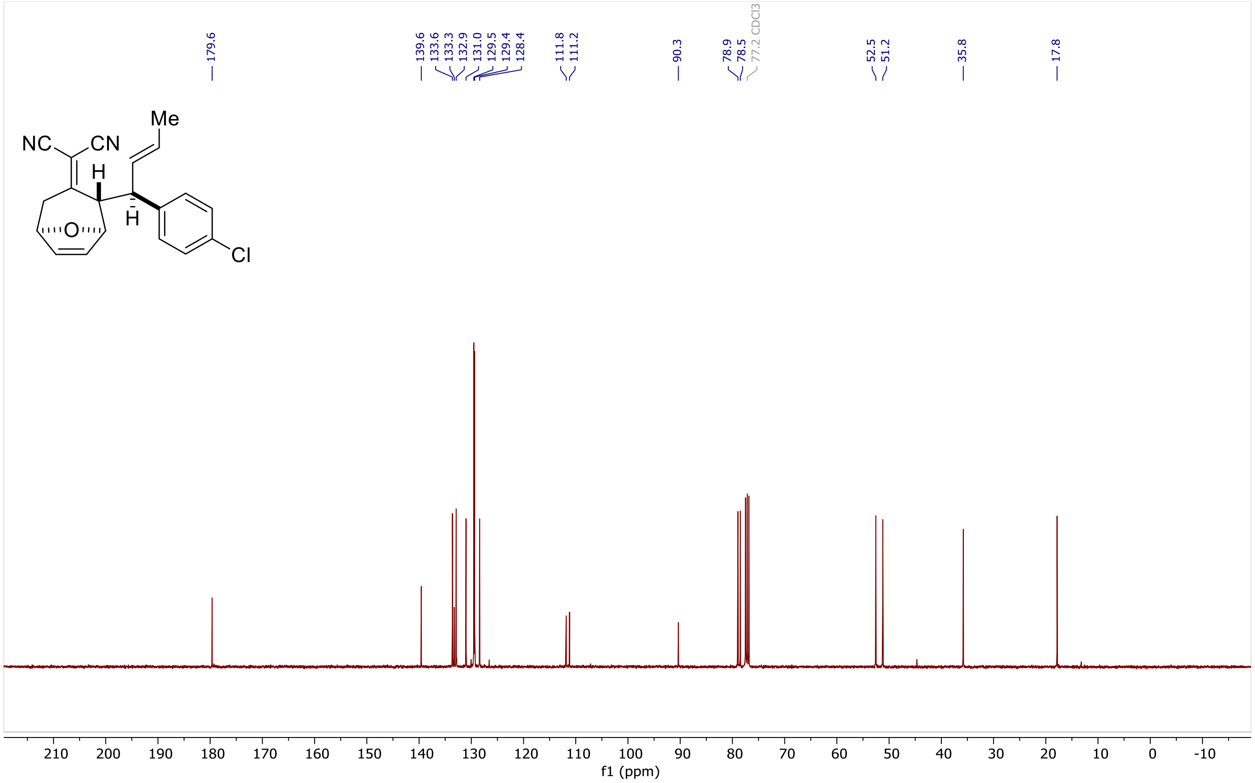

^{1}H NMR (400 MHz, CDCl$_3$) of **5a**

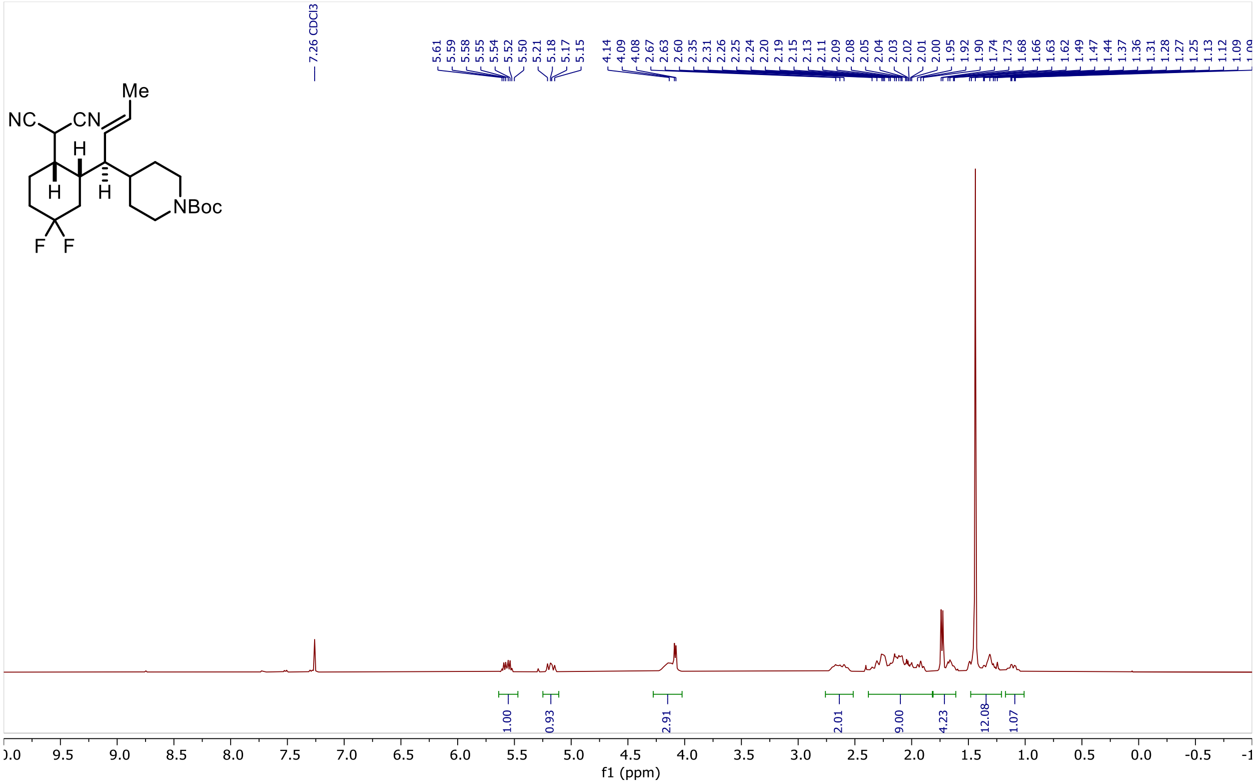

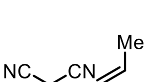

^{13}C NMR (101 MHz, $CDCl_3$) of **5a**

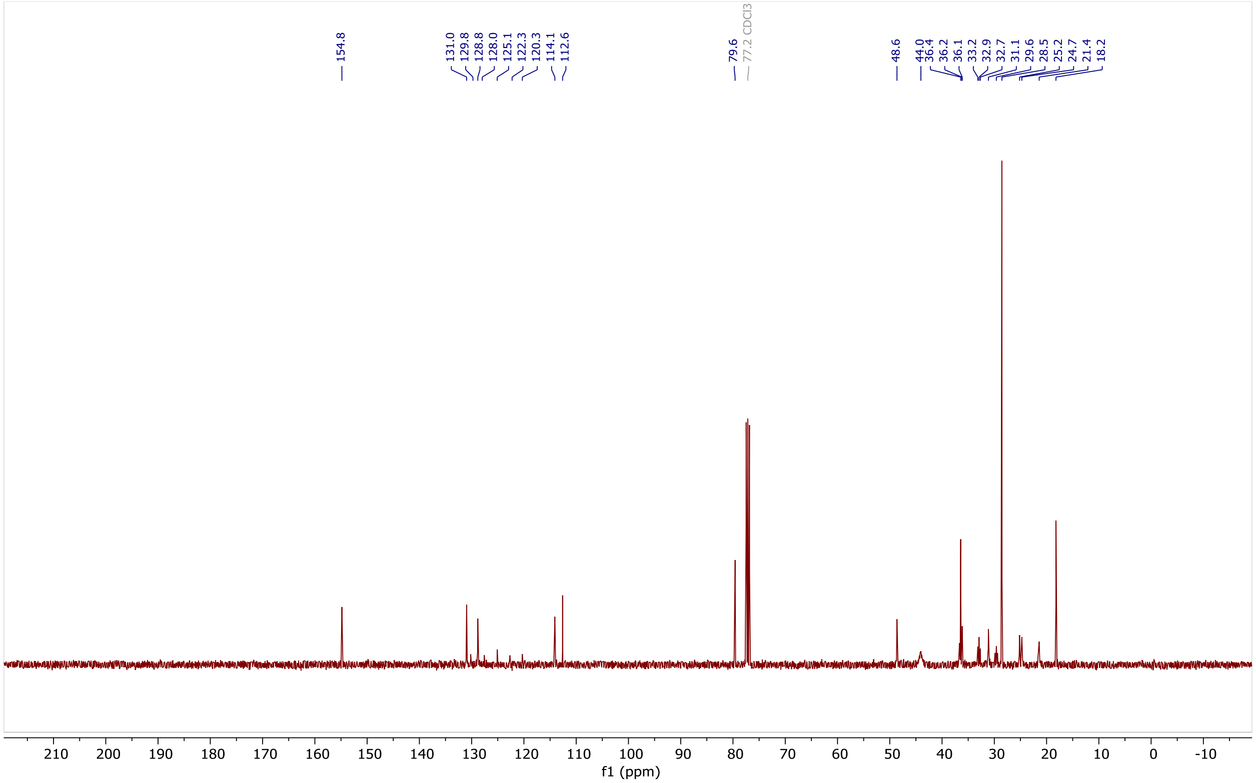

^{19}F NMR (377 MHz, $CDCl_3$) of **5a**

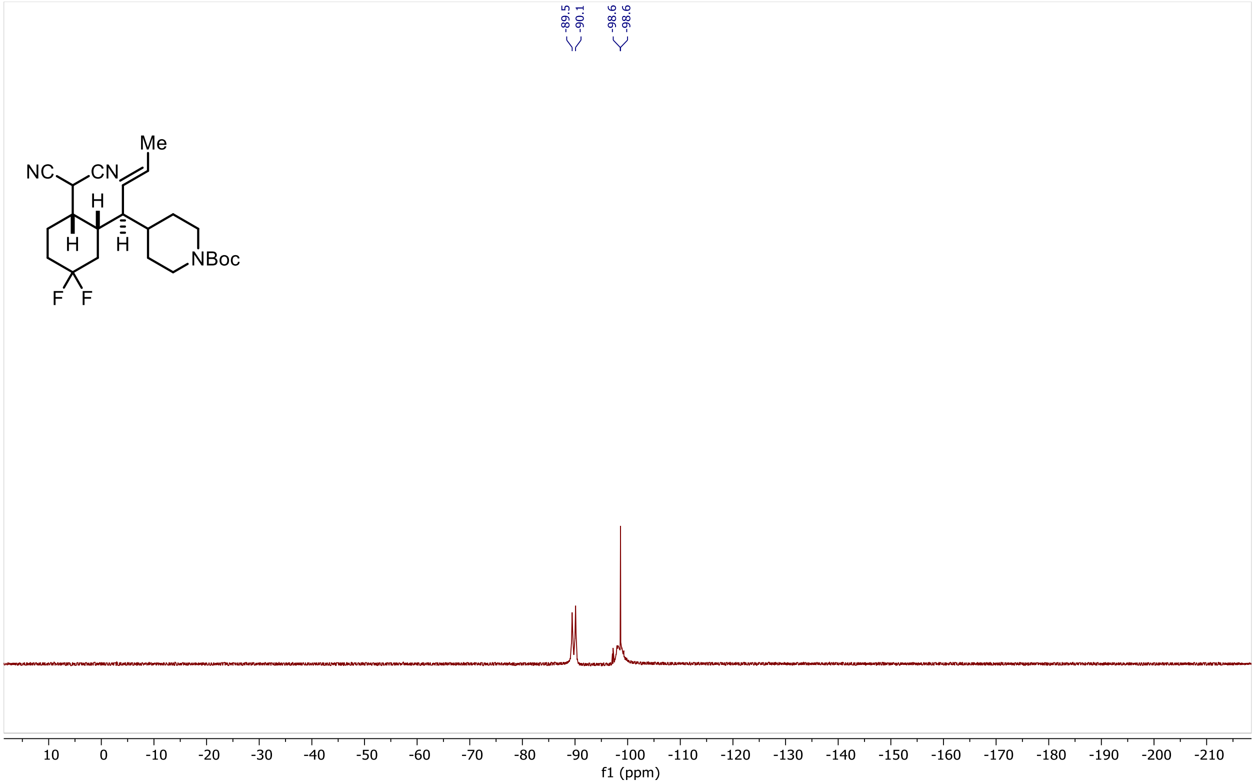

^{1}H NMR (400 MHz, CDCl$_3$) of 5b

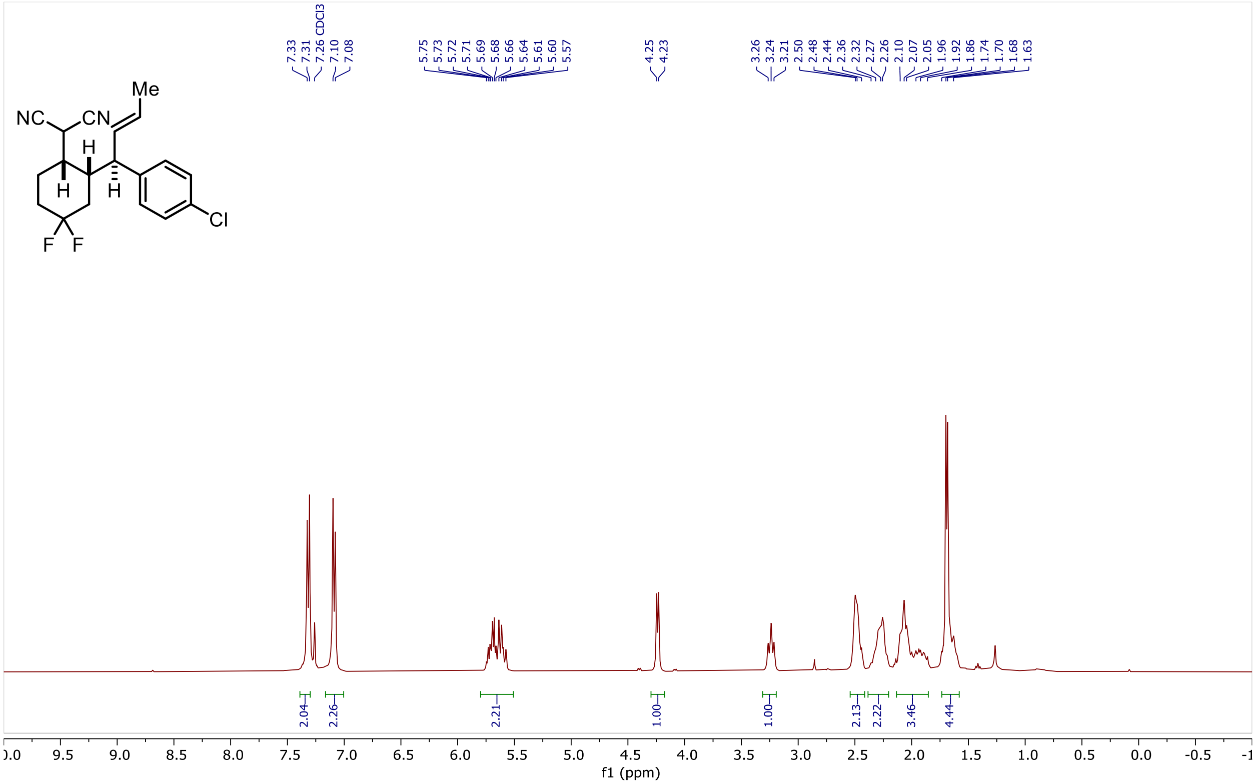

^{13}C NMR (101 MHz, CDCl$_3$) of 5b

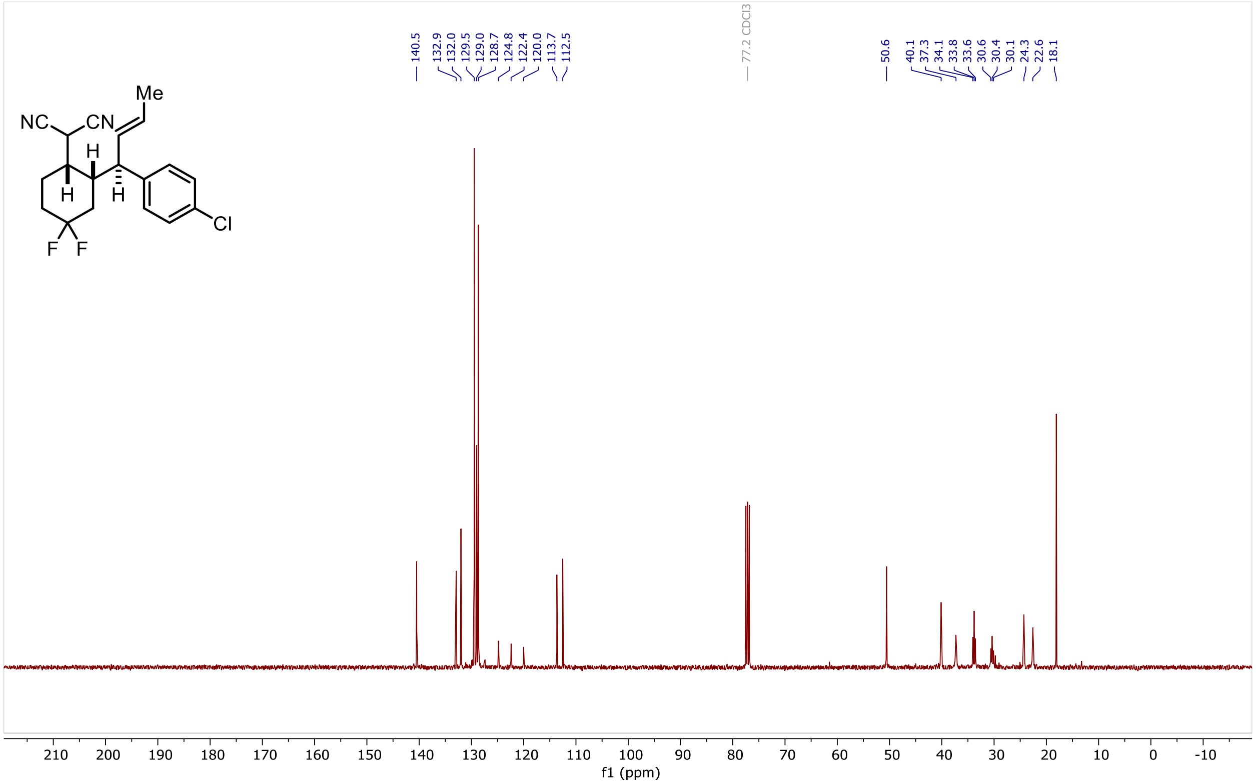

^{19}F NMR (377 MHz, $CDCl_3$) of 5b

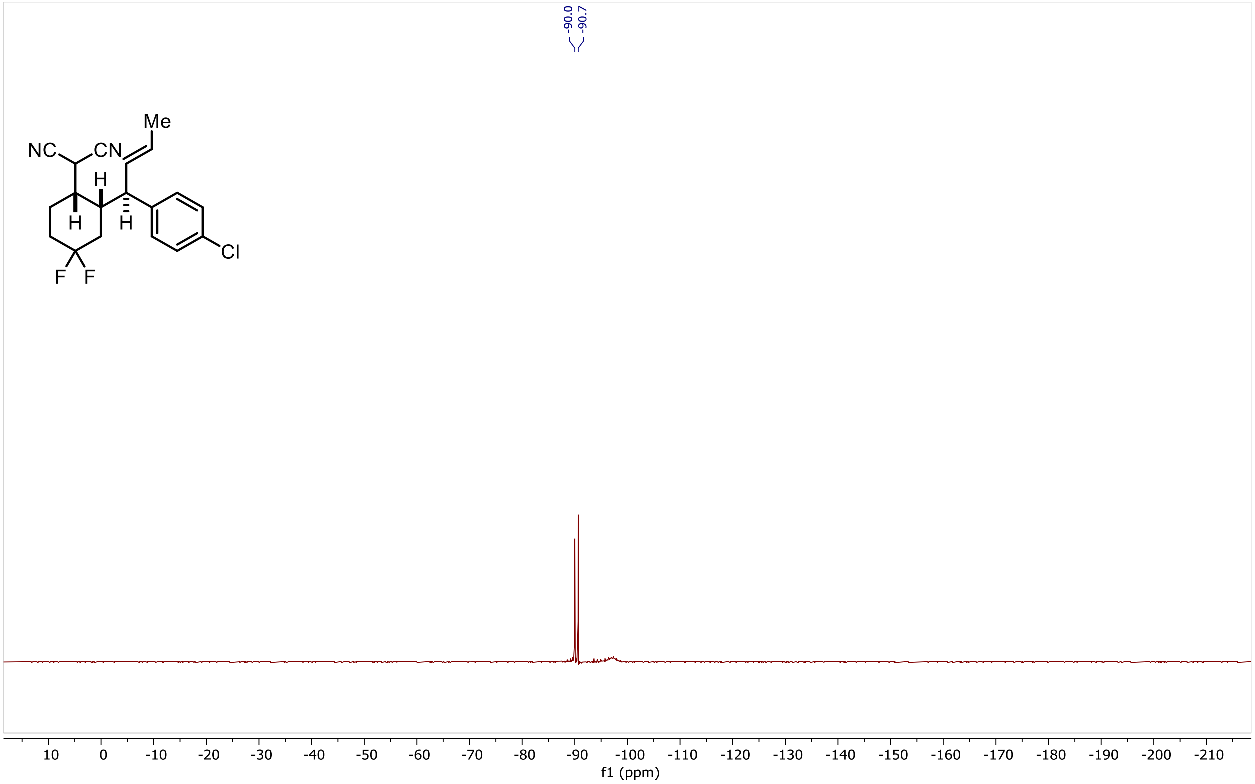

^{1}H NMR (400 MHz, CD_3CN) of 6ff

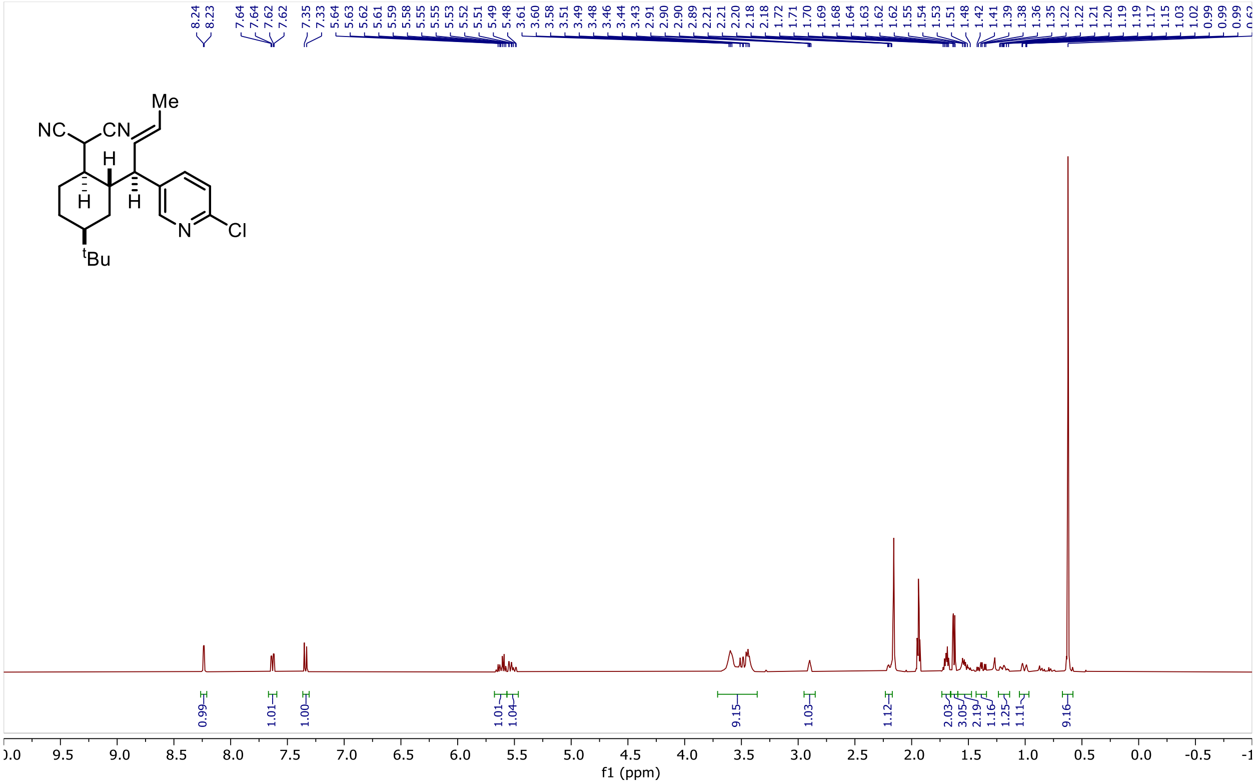

^{13}C NMR (101 MHz, CD_3CN) of 6ff

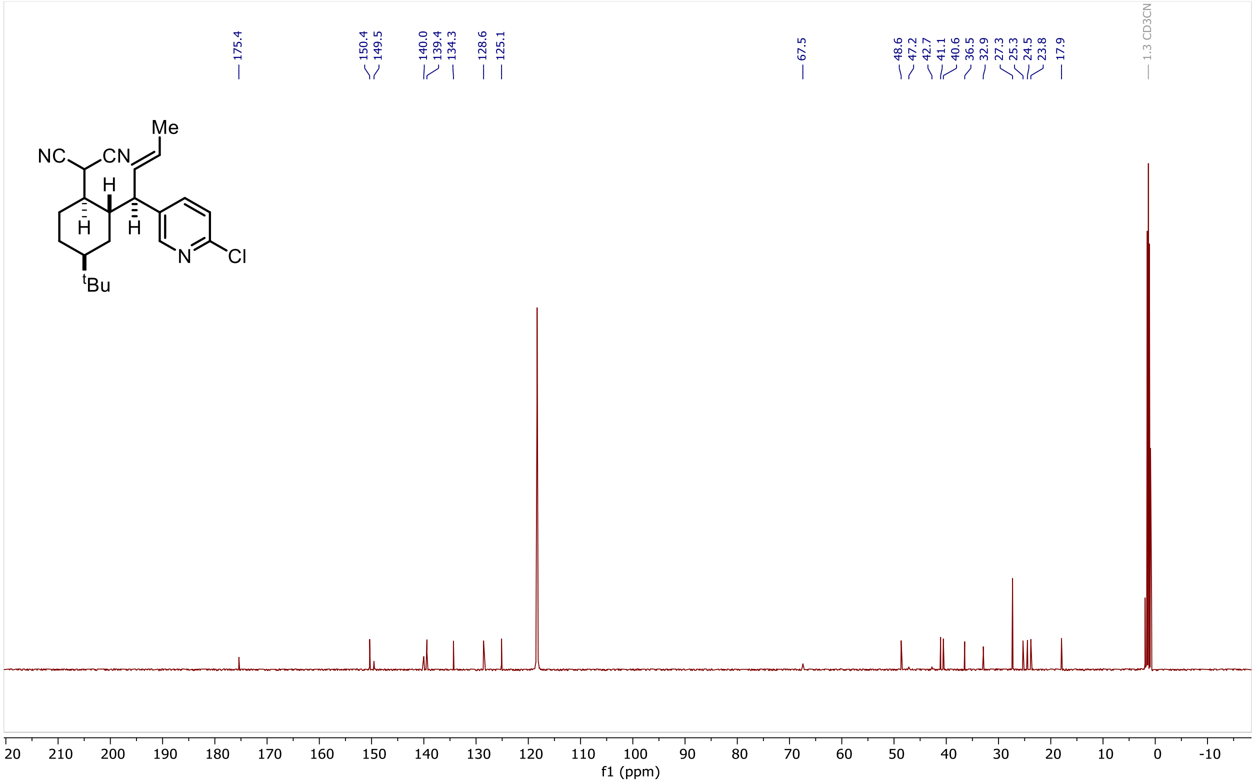

^{1}H NMR (400 MHz, CD_3CN) of SI-3

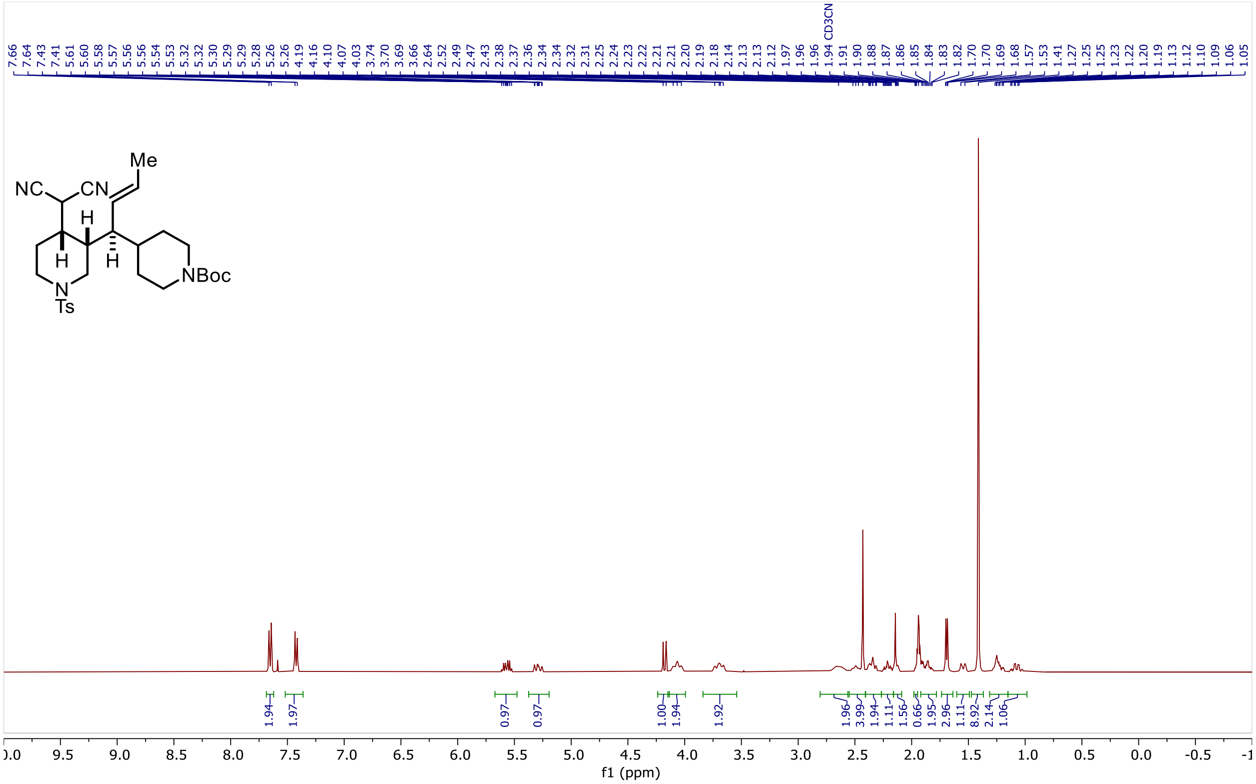

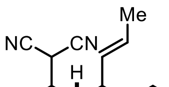

^{13}C NMR (101 MHz, CD_3CN) of **SI-3**

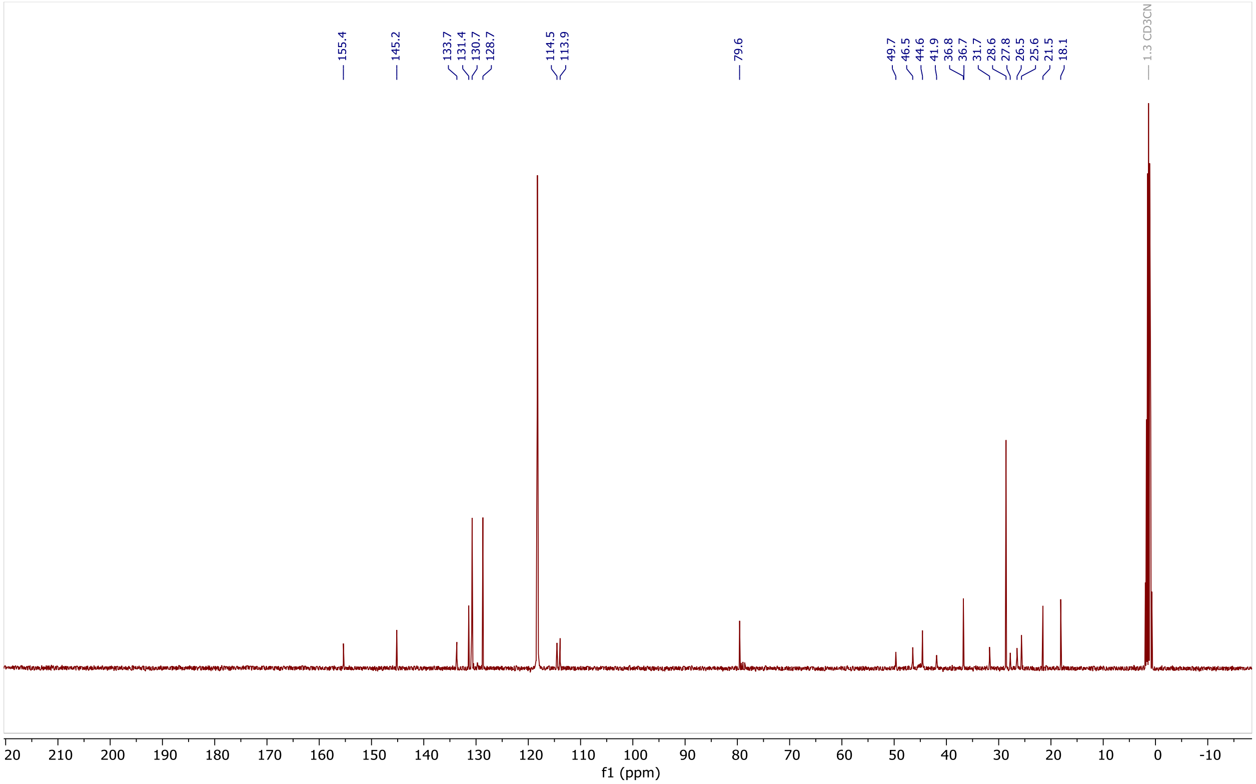

^{1}H NMR (400 MHz, $CDCl_3$) of **SI-4**

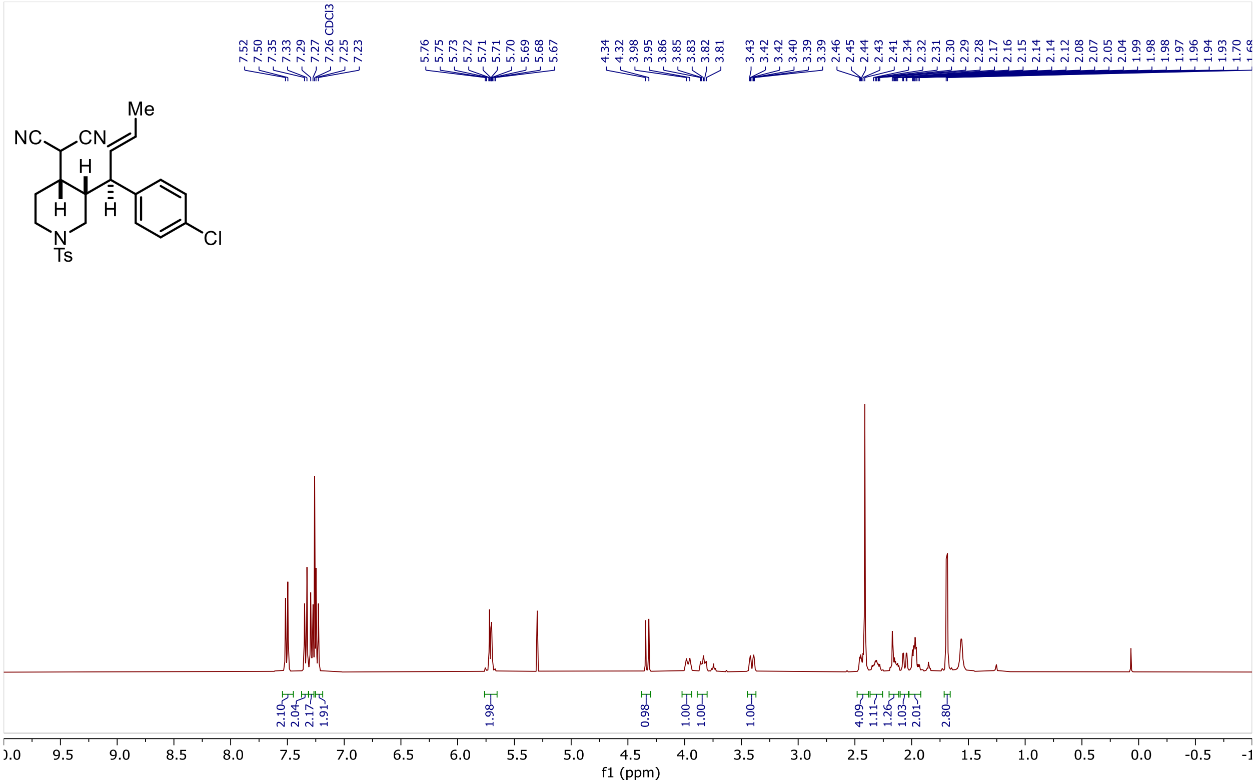

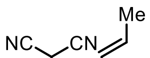

^{13}C NMR: (101 MHz, CDCl$_3$) of **SI-4**

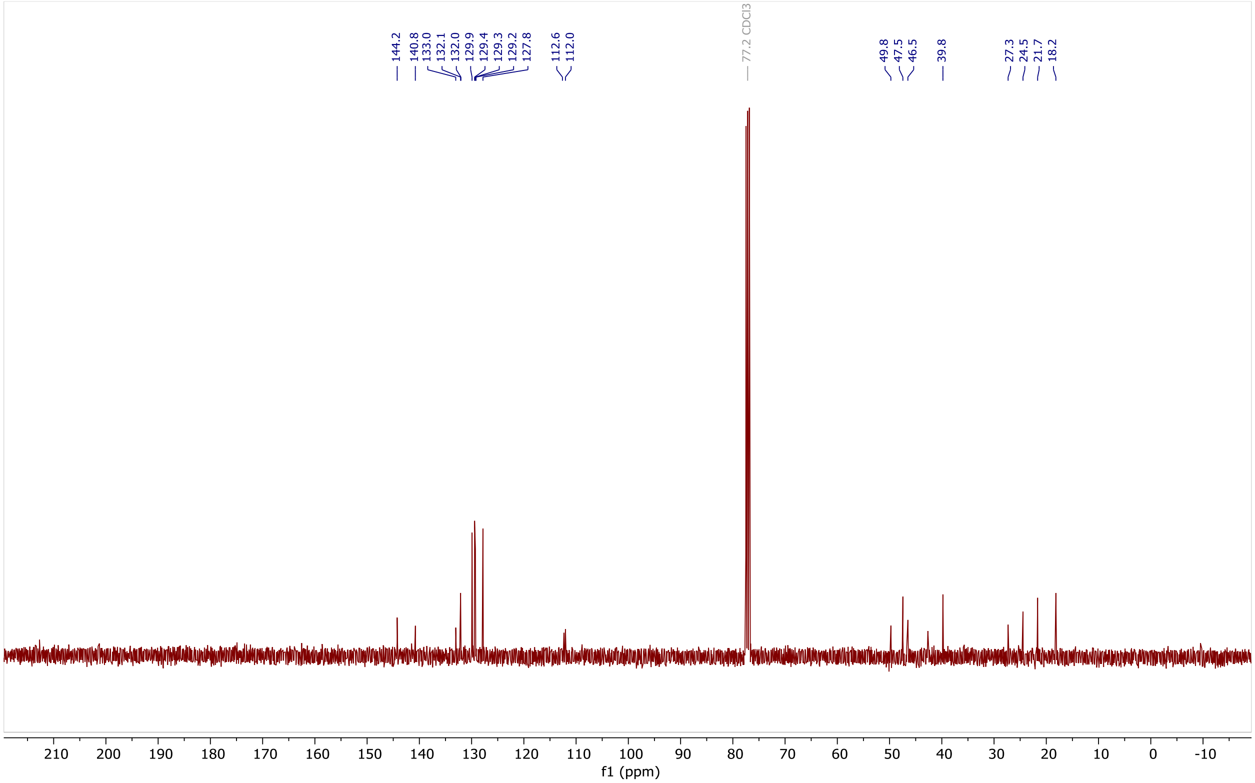

^{1}H NMR (400 MHz, CD$_3$CN) of **SI-5**

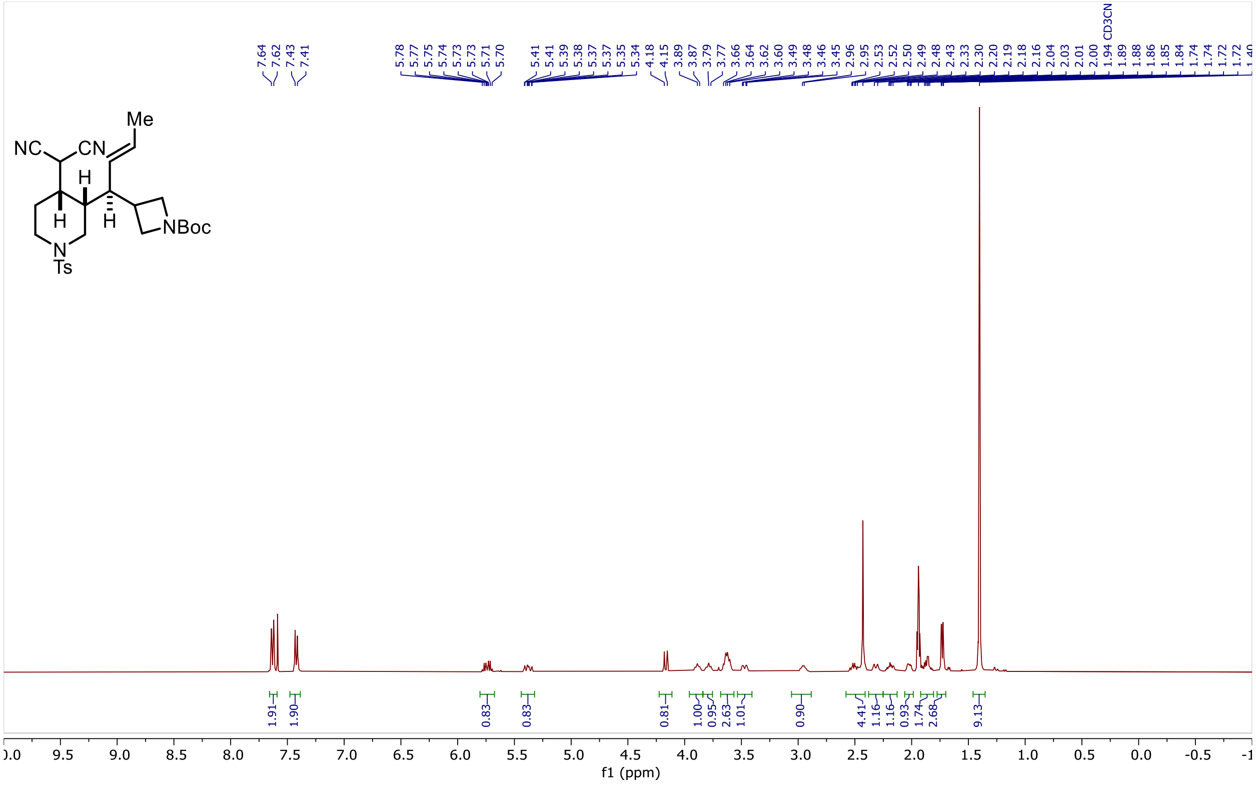

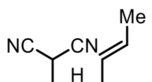

^{13}C NMR (101 MHz, CD_3CN) of SI-5

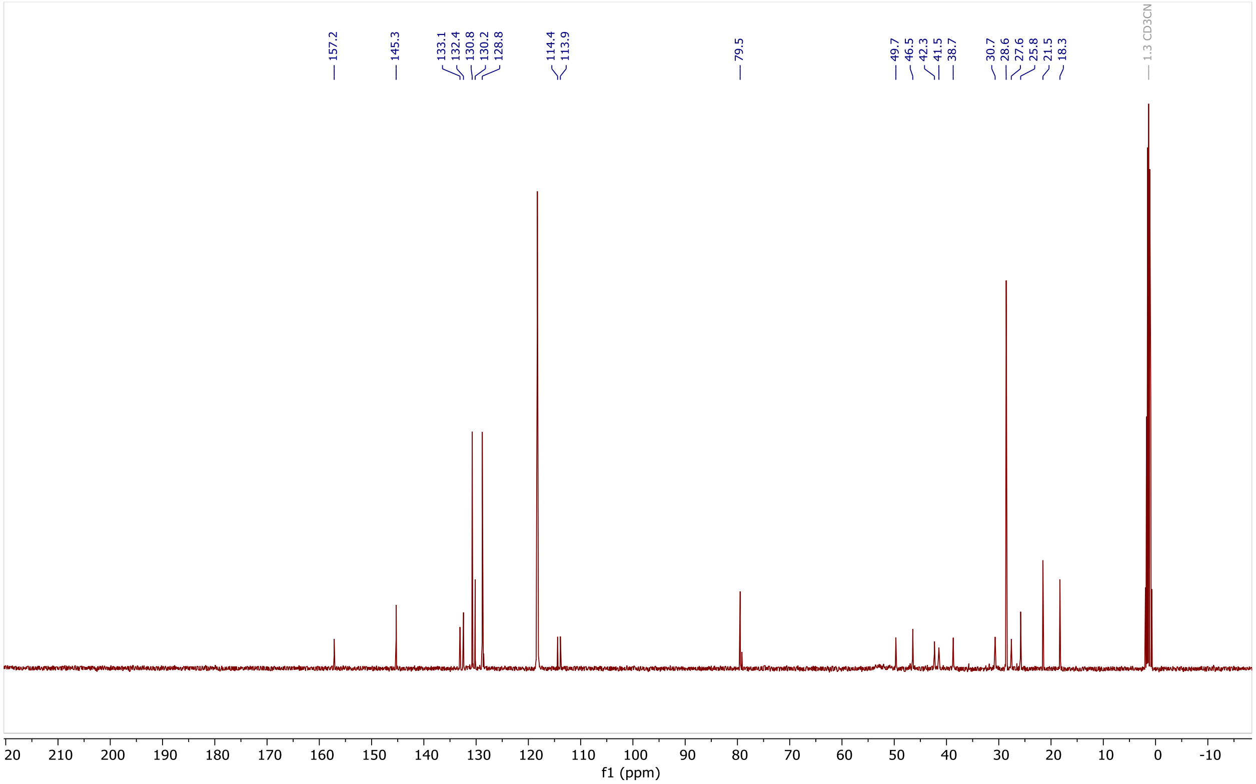

^{1}H NMR (400 MHz, CD_3CN) of SI-6

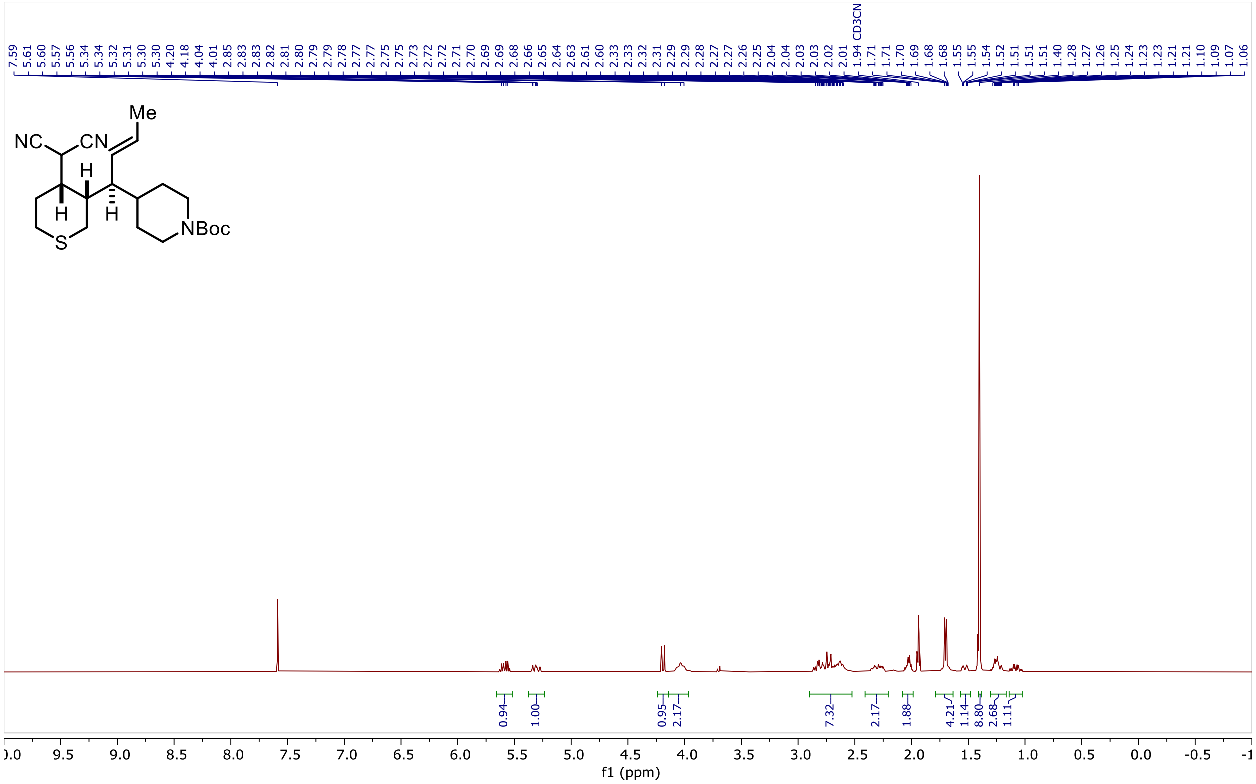

^{13}C NMR (101 MHz, CD$_3$CN) of SI-6

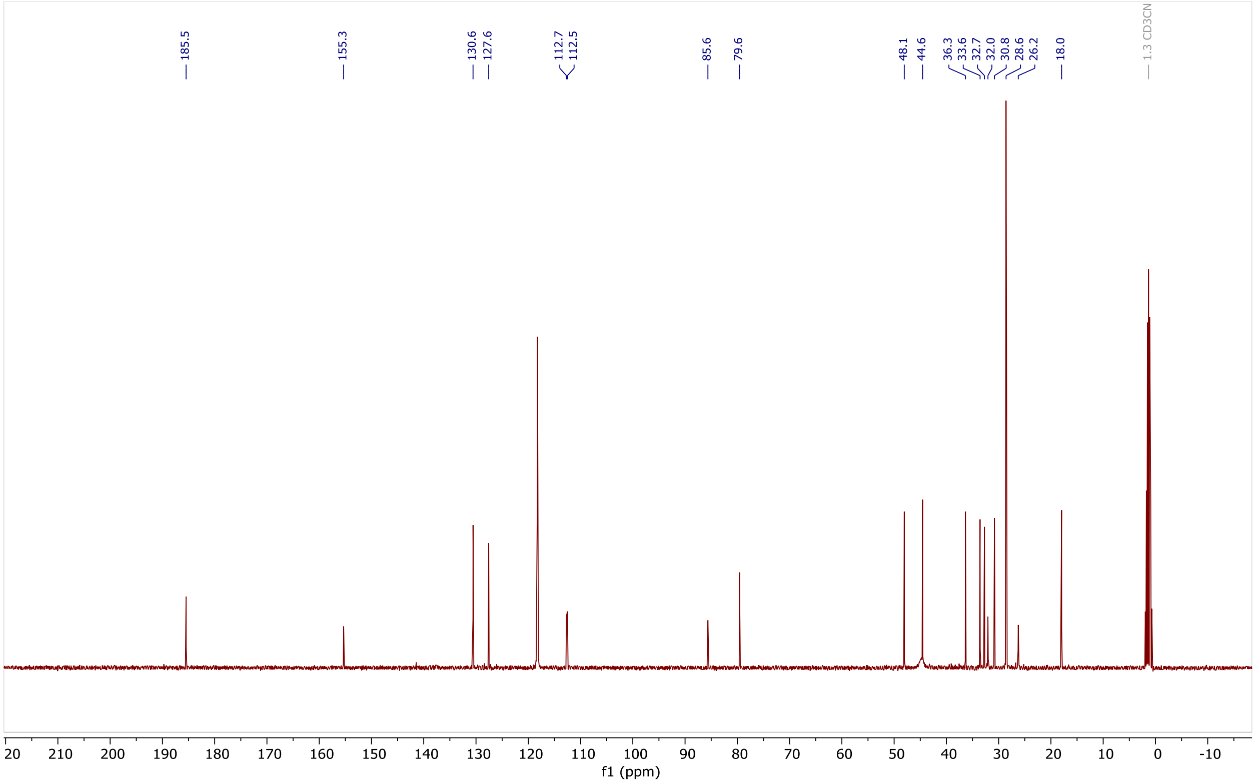

^{1}H NMR (500 MHz, DMSO-*d$_6$*, 354 K) of SI-7

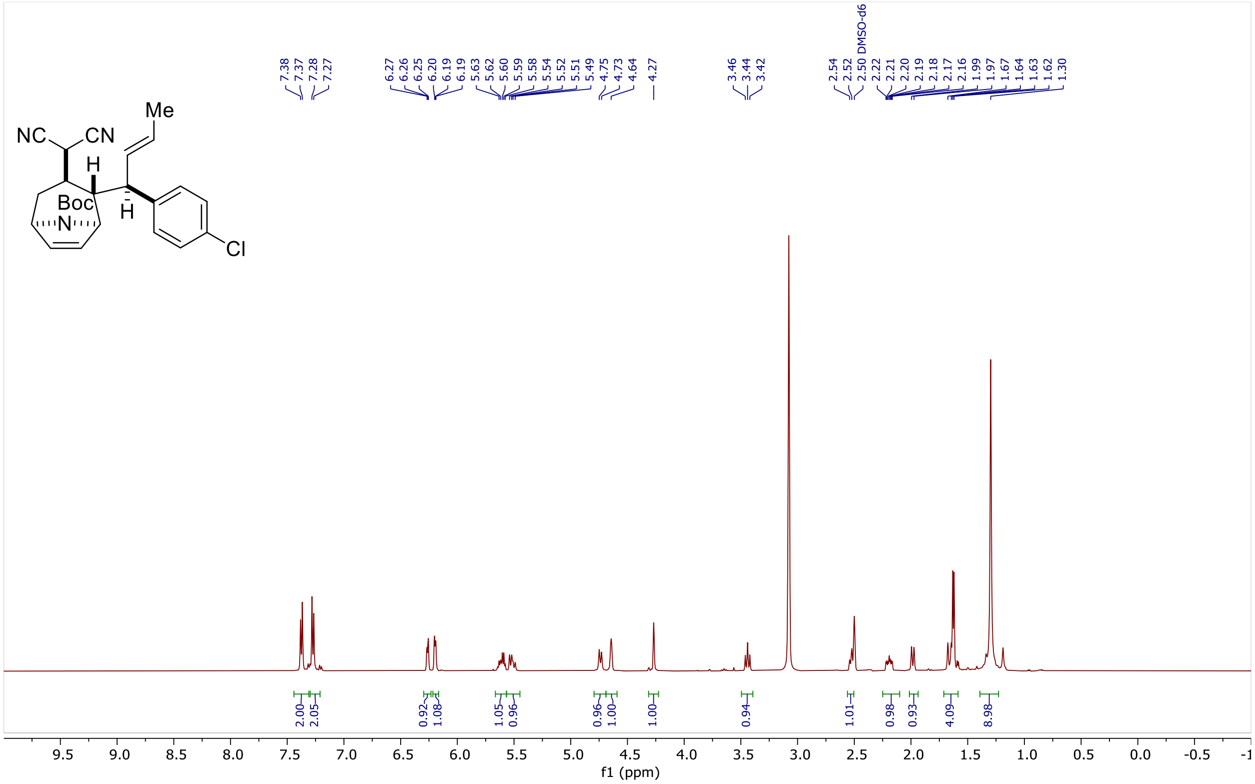

^{13}C NMR (126 MHz, DMSO-d_6, 354 K) of **SI-7**

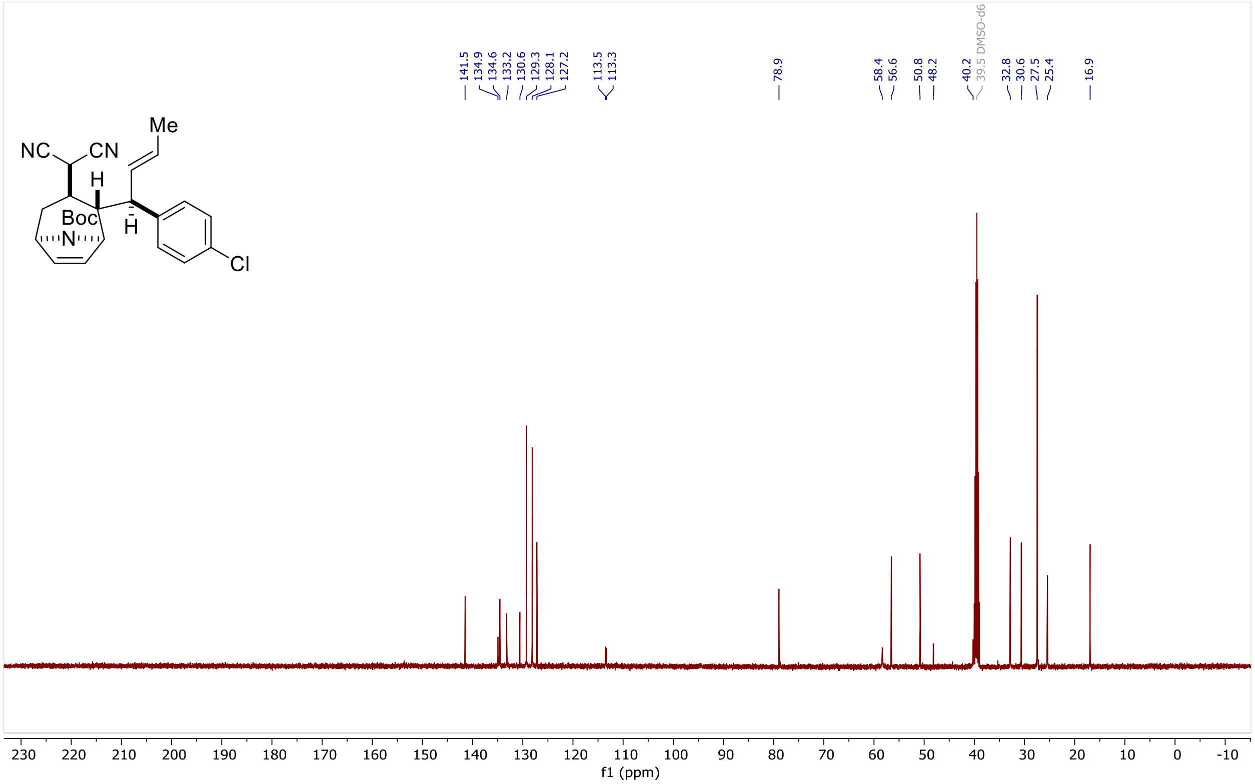

^{1}H NMR (400 MHz, CD_3CN) of **6a**

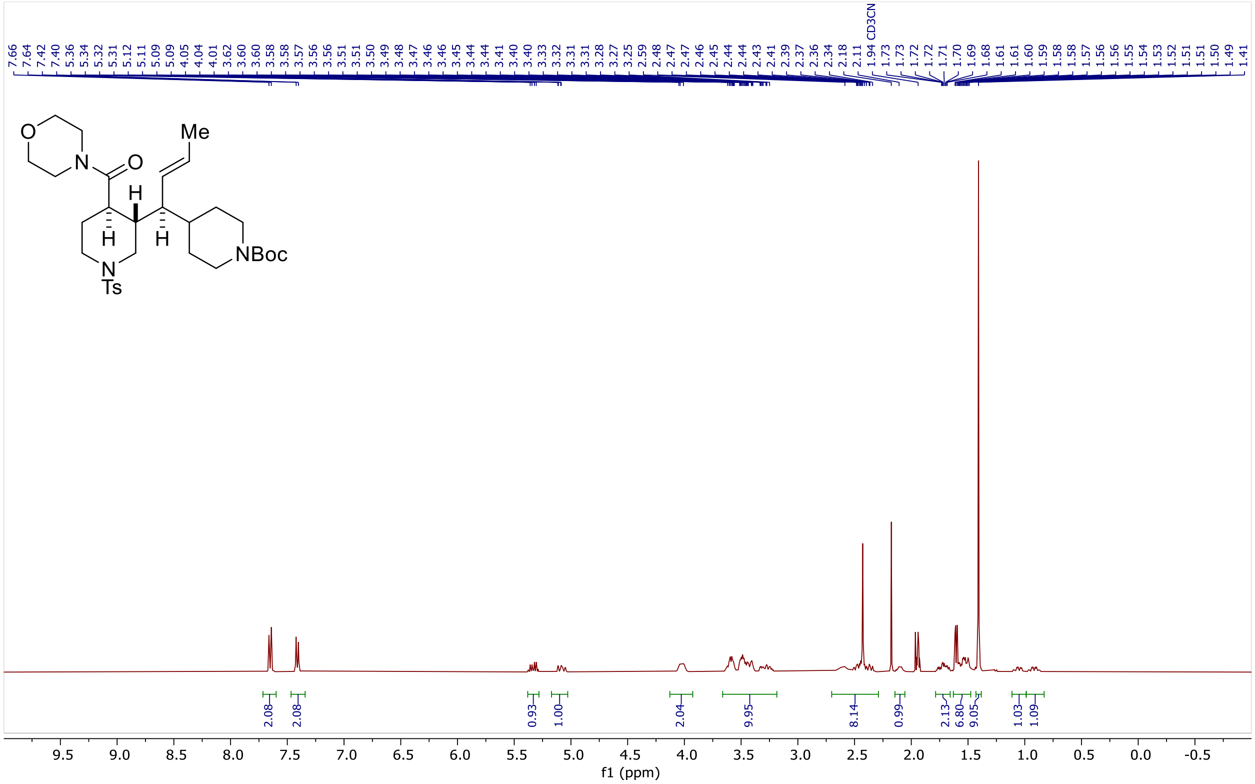

^{13}C NMR (101 MHz, CD_3CN) of 6a

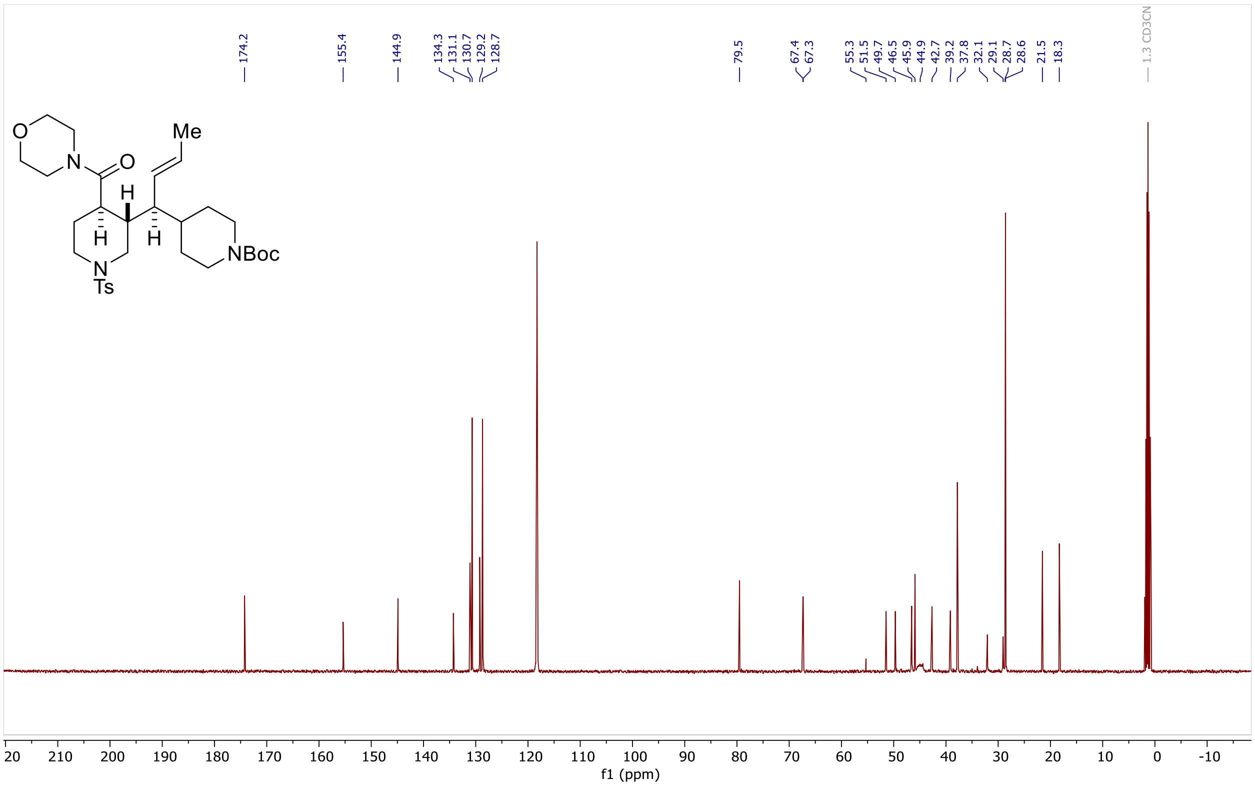

^{1}H NMR (400 MHz, $CDCl_3$) of 6b

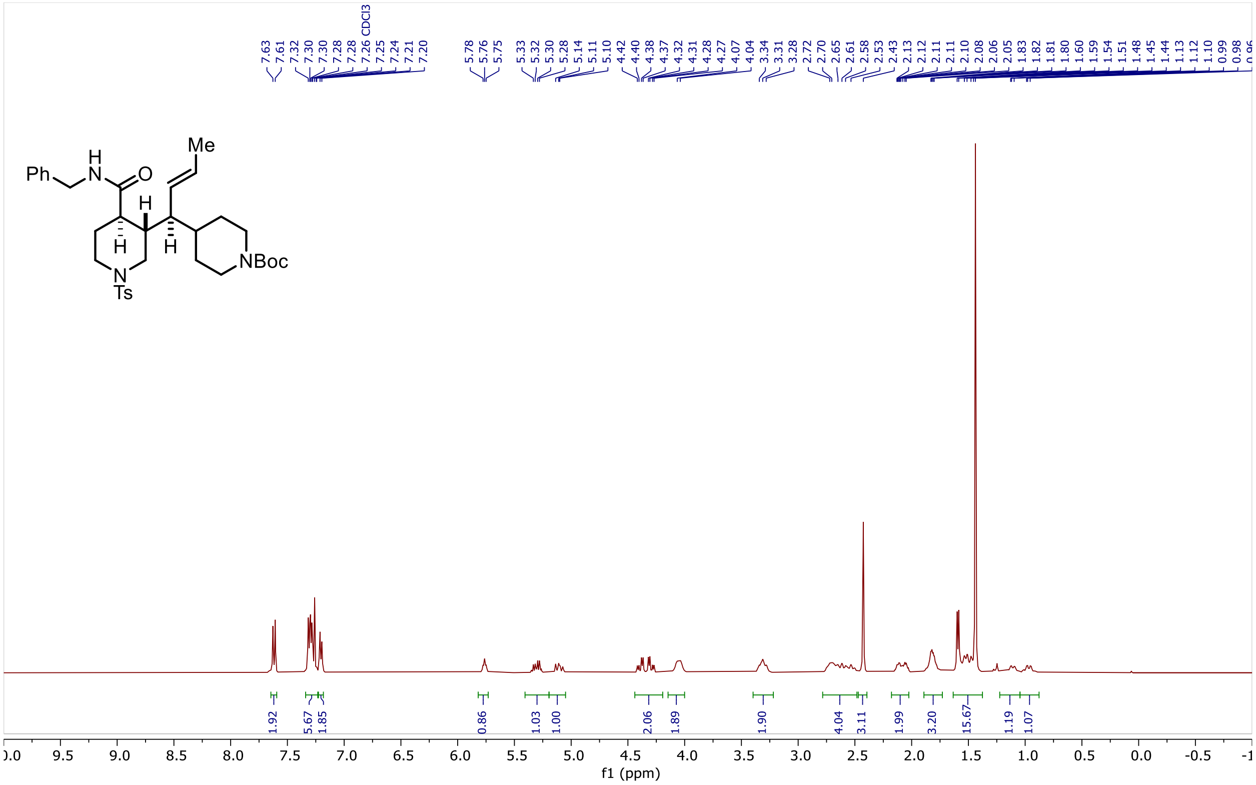

^{13}C NMR (101 MHz, CDCl$_3$) of 6b

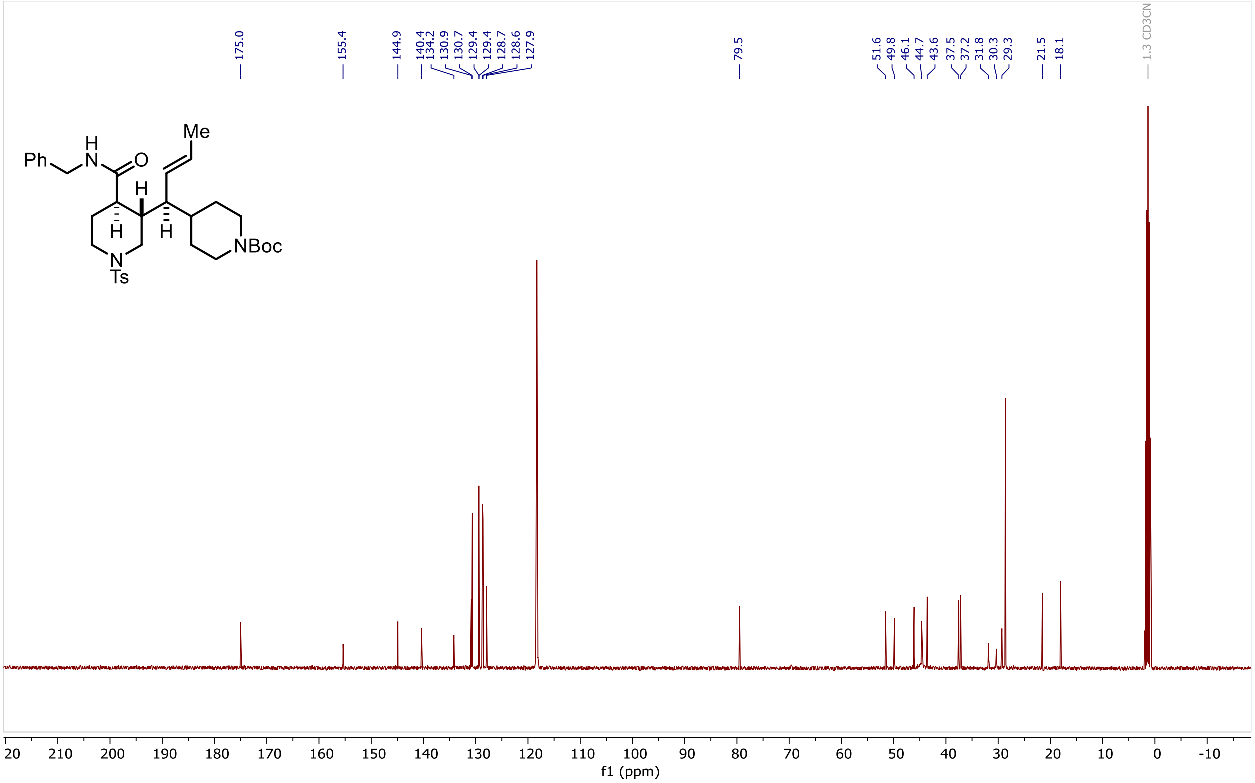

^{1}H NMR (600 MHz, CD$_3$CN) of 6c

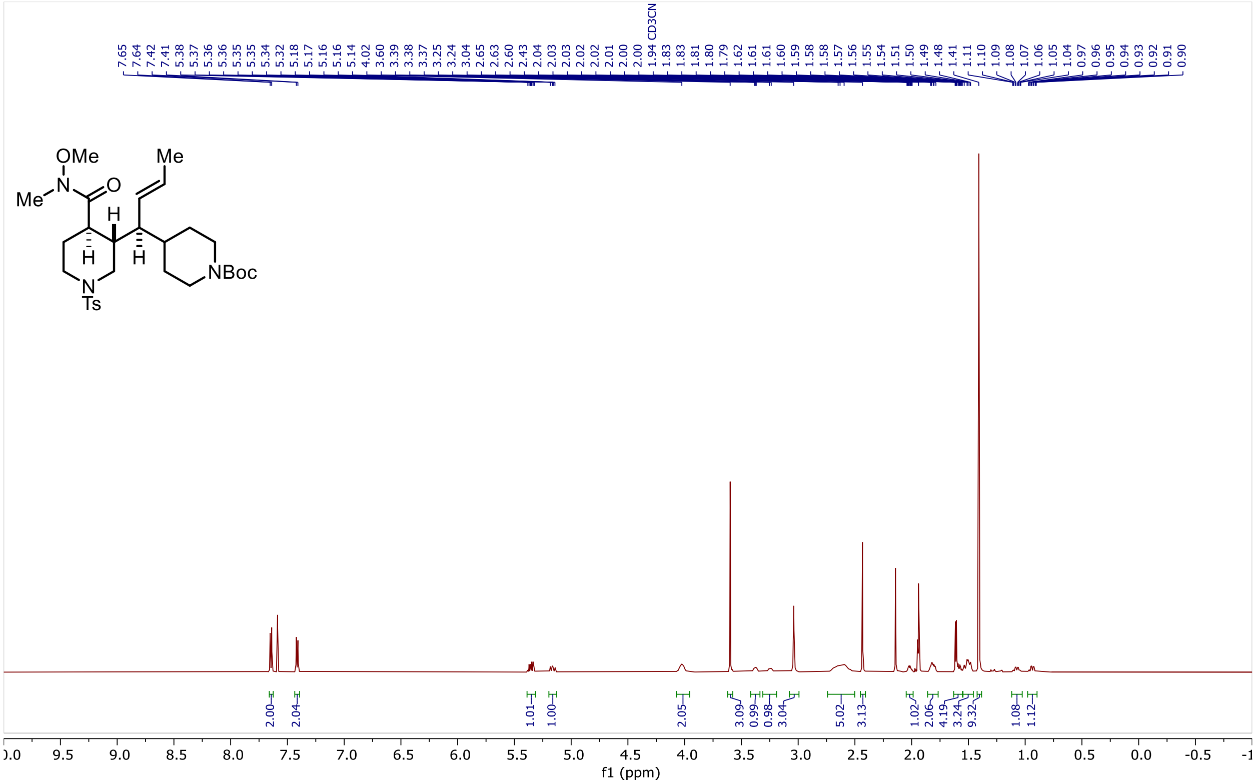

^{13}C NMR (151 MHz, CD_3CN) of **6c**

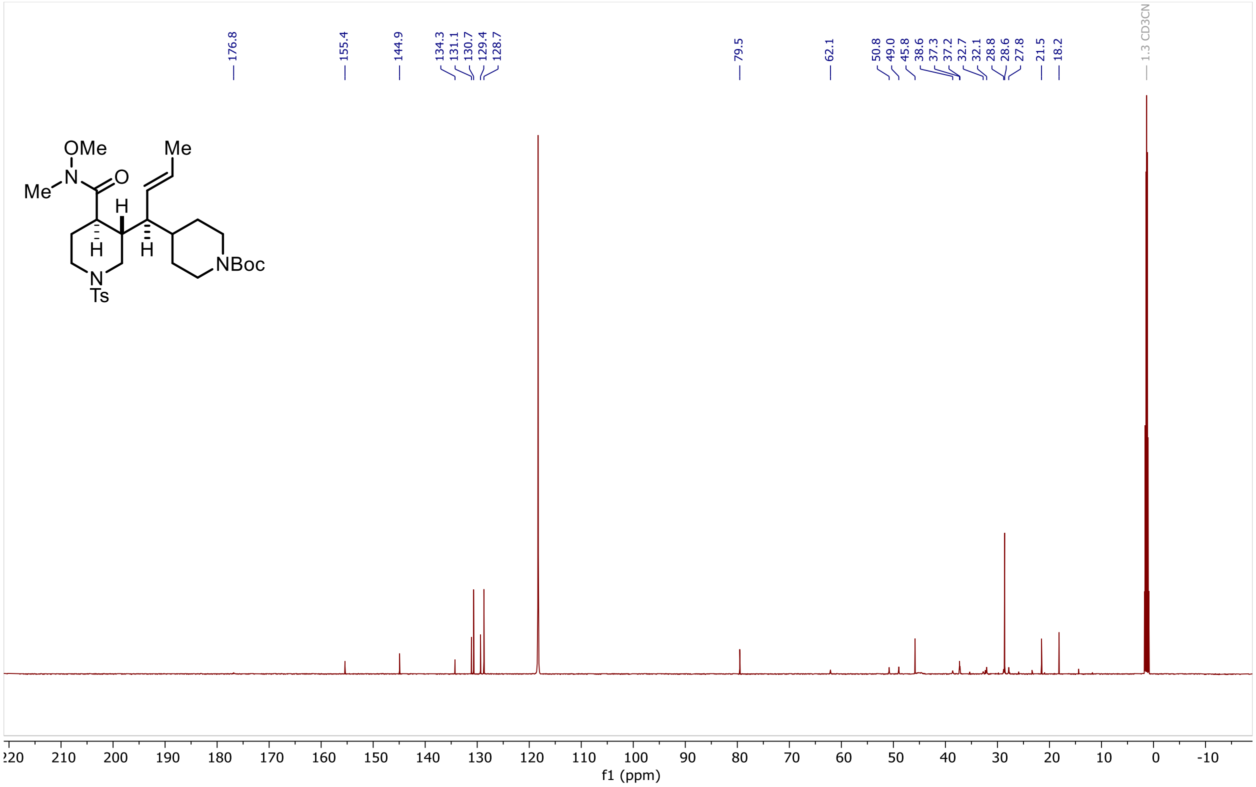

^{1}H NMR (400 MHz, $CDCl_3$) of **6d**

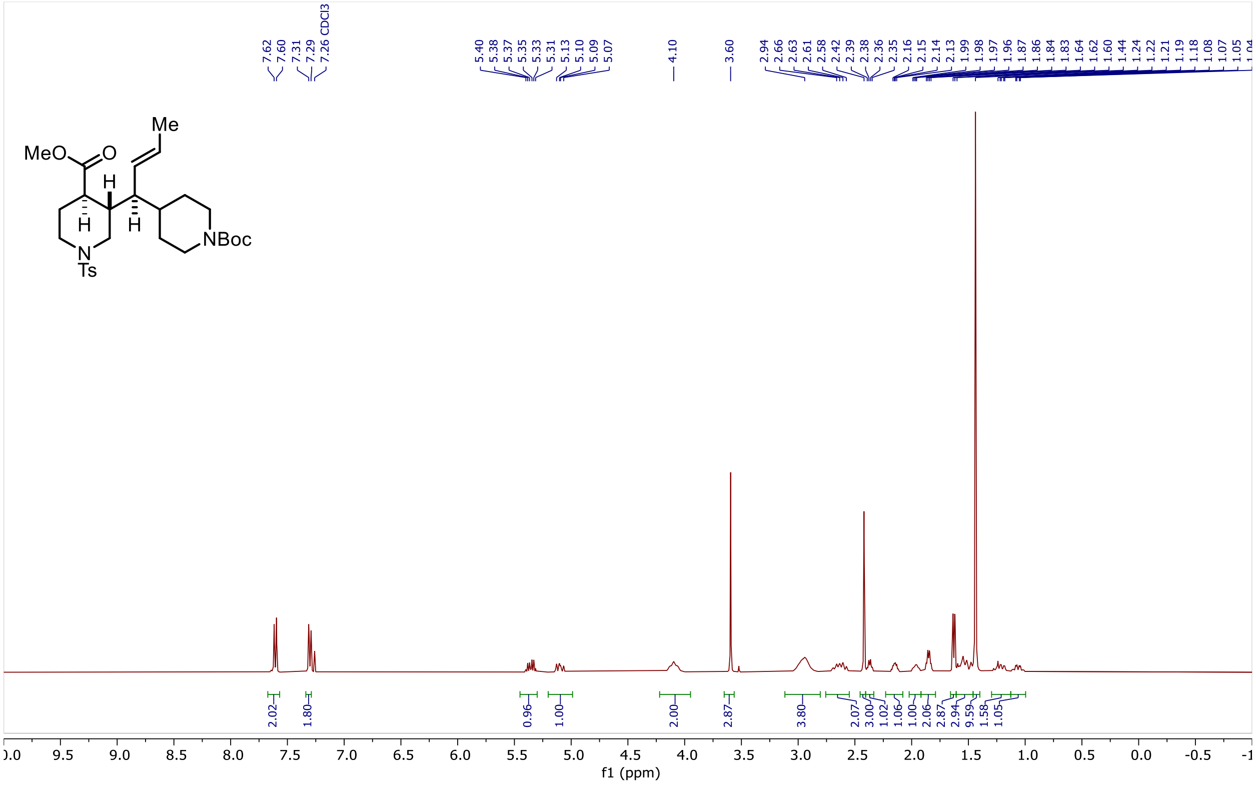

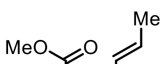

^{13}C NMR (101 MHz, CDCl$_3$) of 6d

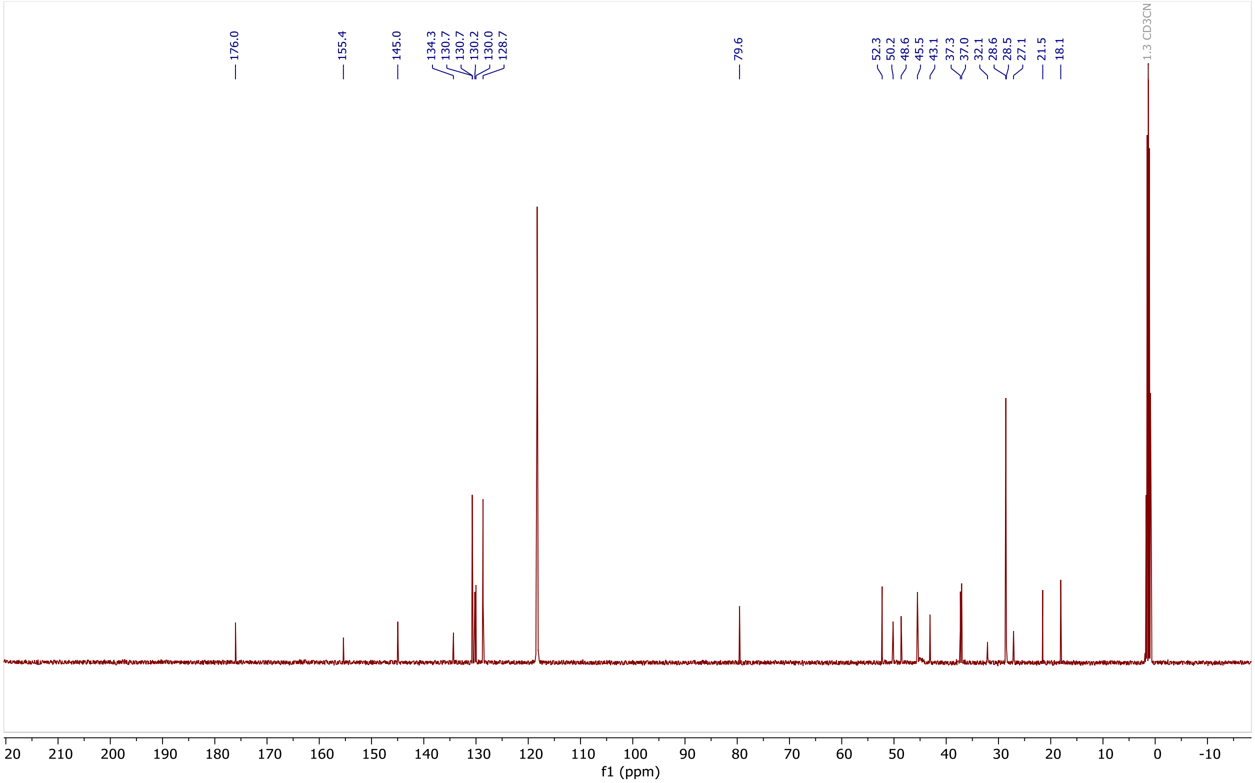

^{1}H NMR (400 MHz, CDCl$_3$) of 6e

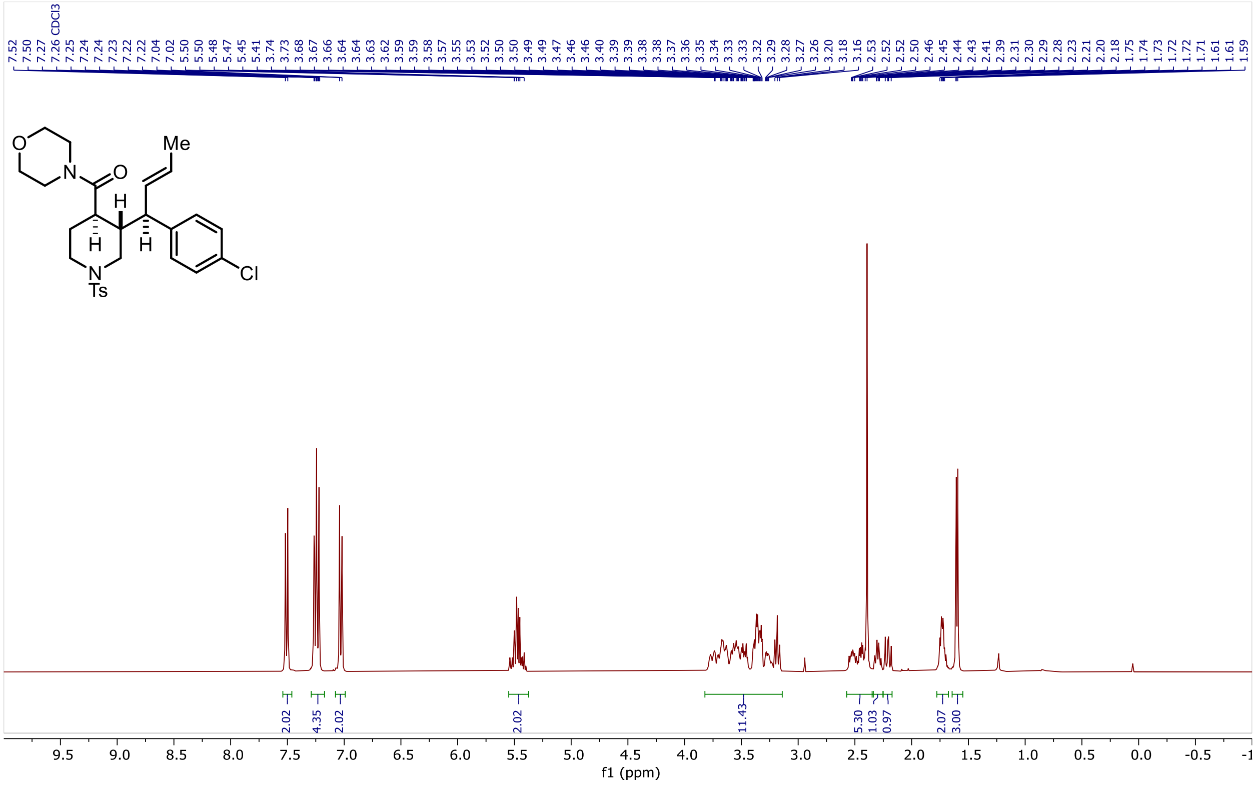

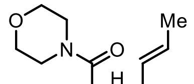

^{13}C NMR (101 MHz, $CDCl_3$) of 6e

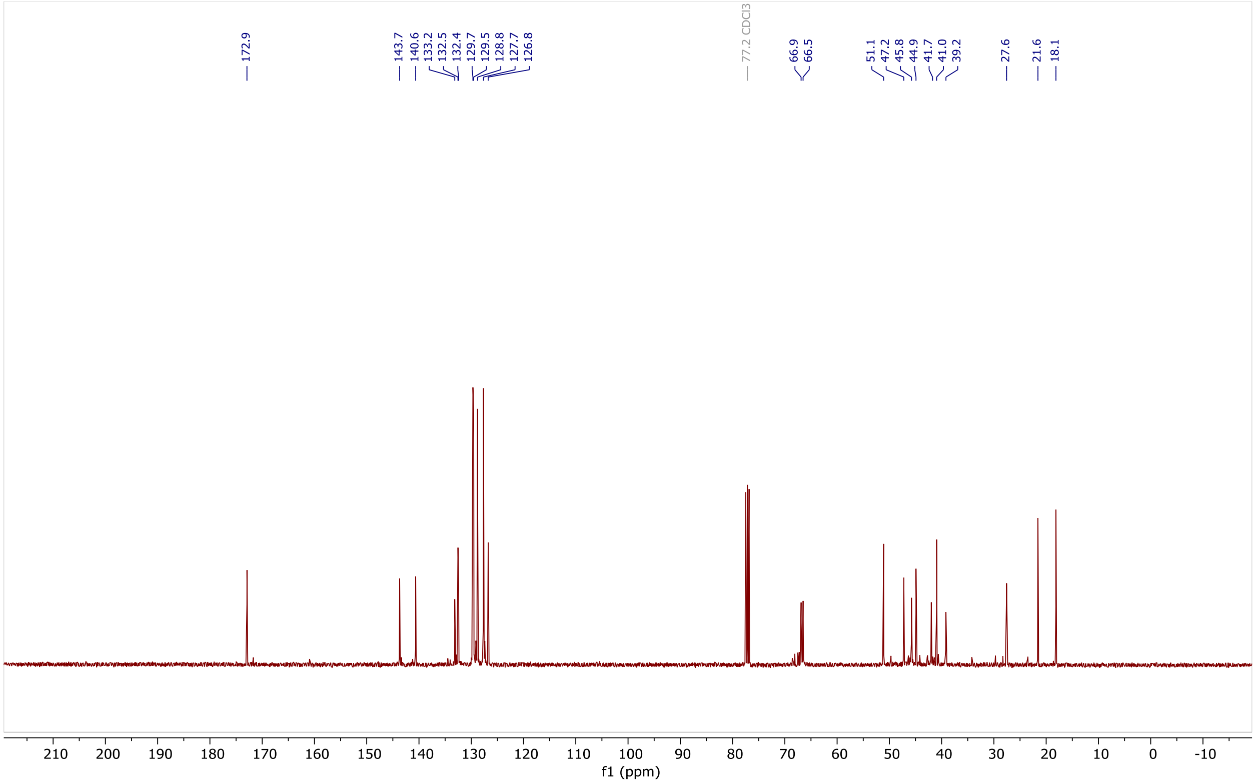

^{1}H NMR (400 MHz, $CDCl_3$) of 6f

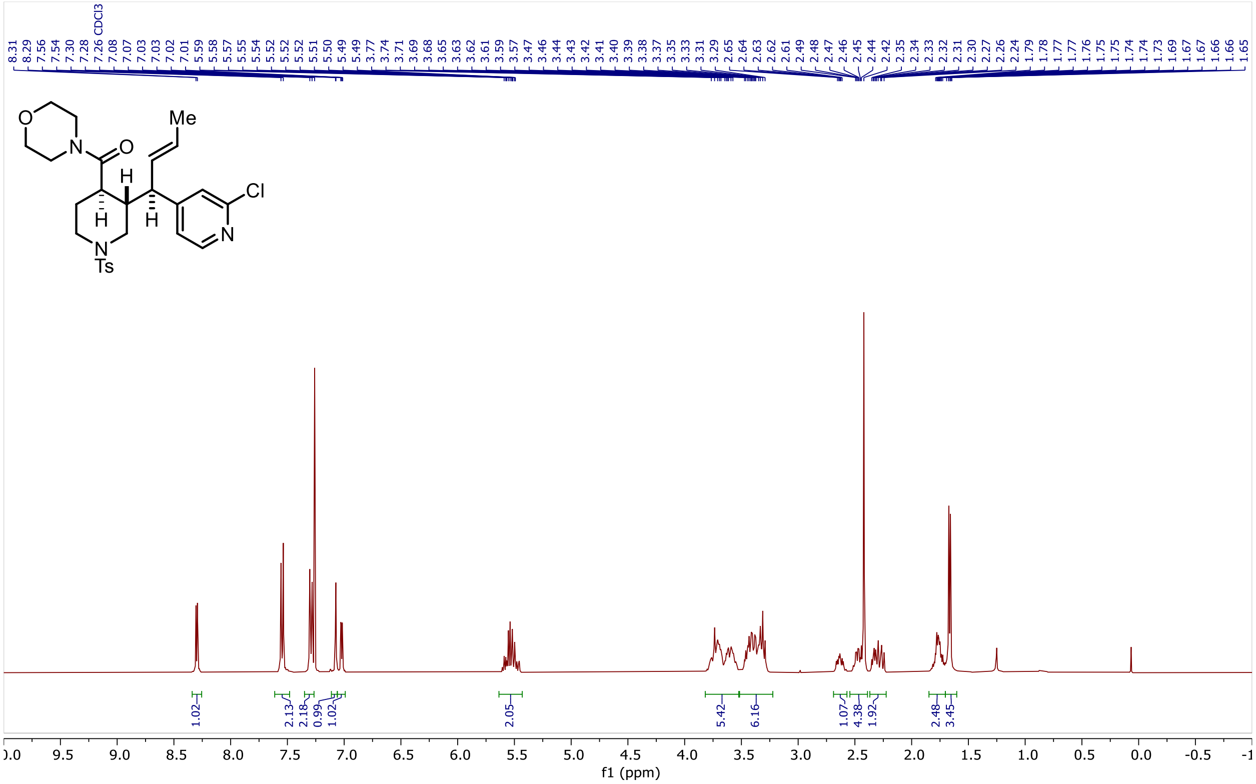

^{13}C NMR (101 MHz, CDCl$_3$) of 6f

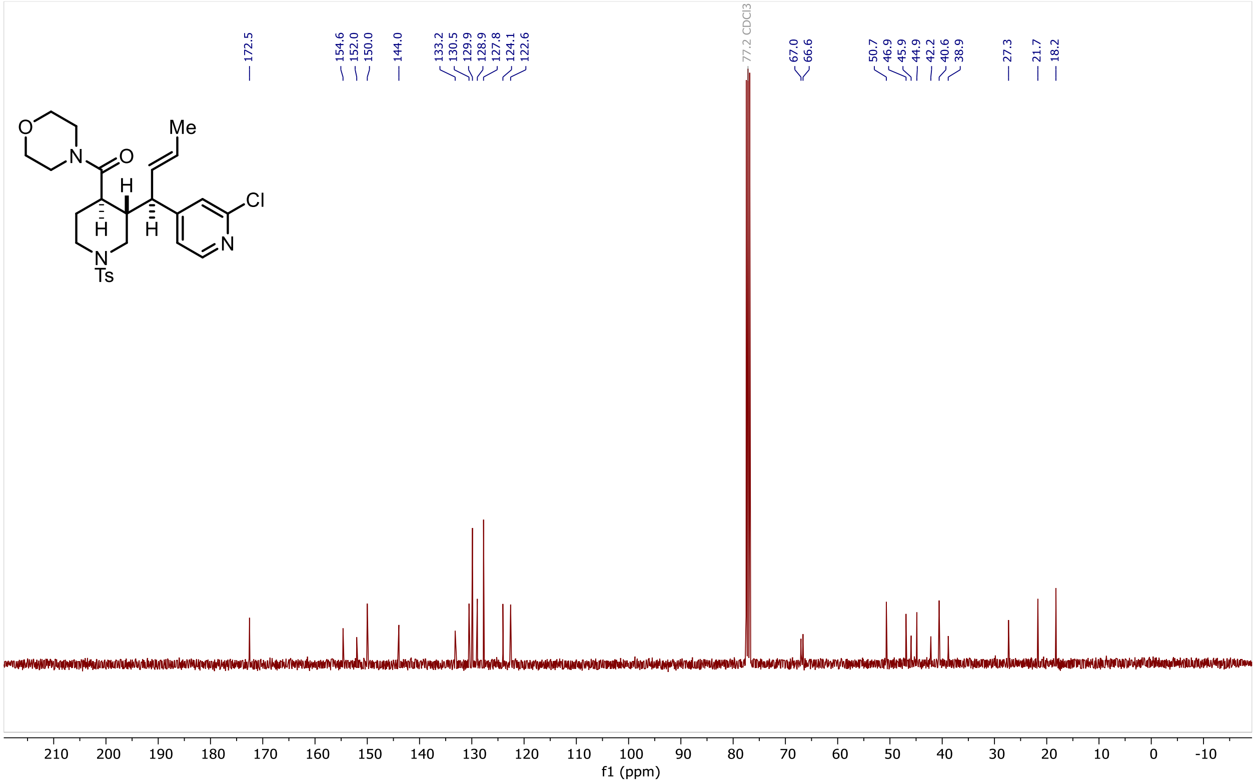

^{1}H NMR (400 MHz, CD$_3$CN) of 6g

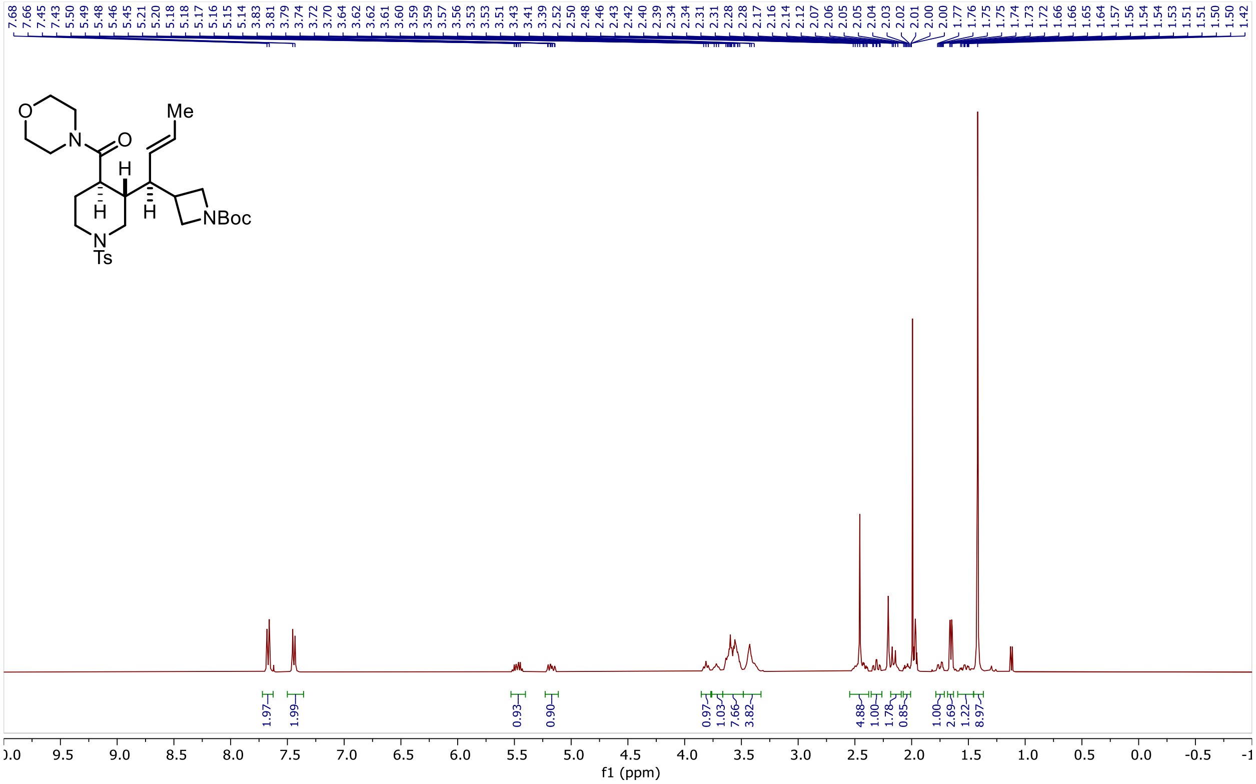

^{13}C NMR (101 MHz, CD₃CN) of **6g**

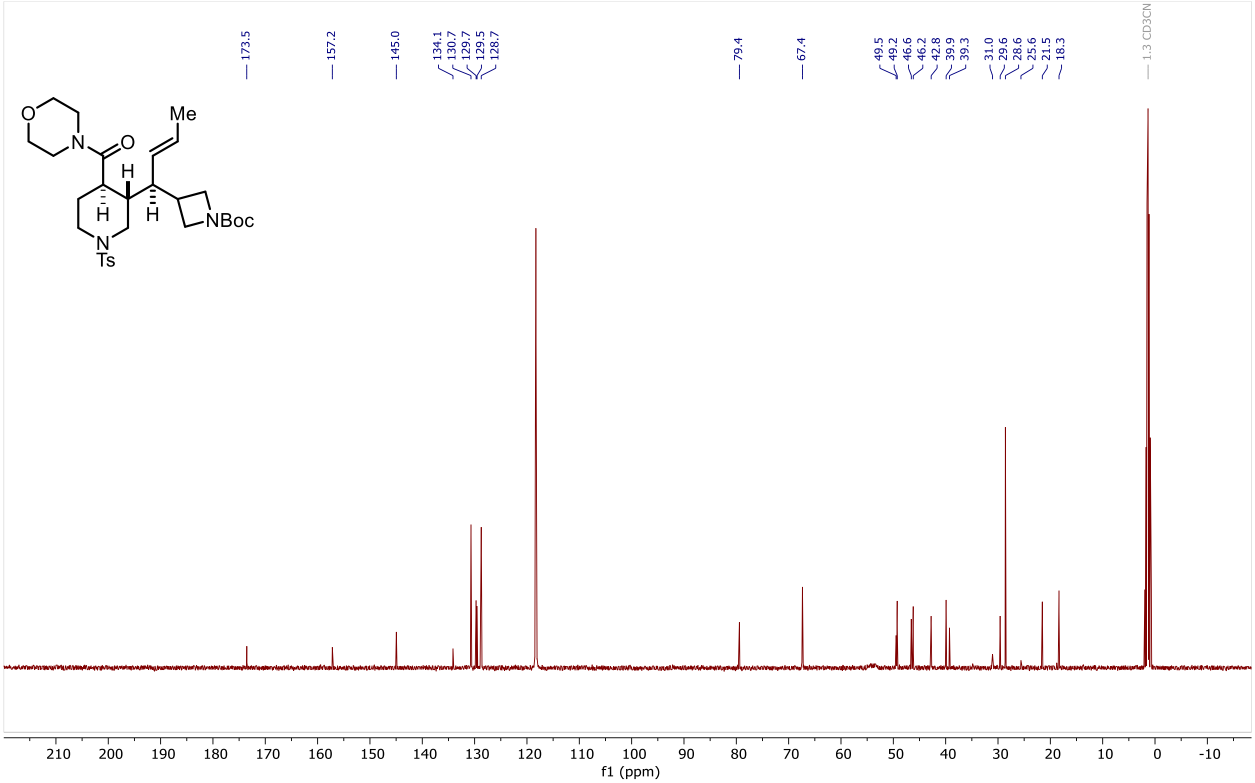

^{1}H NMR (400 MHz, CD₃CN) of **6h**

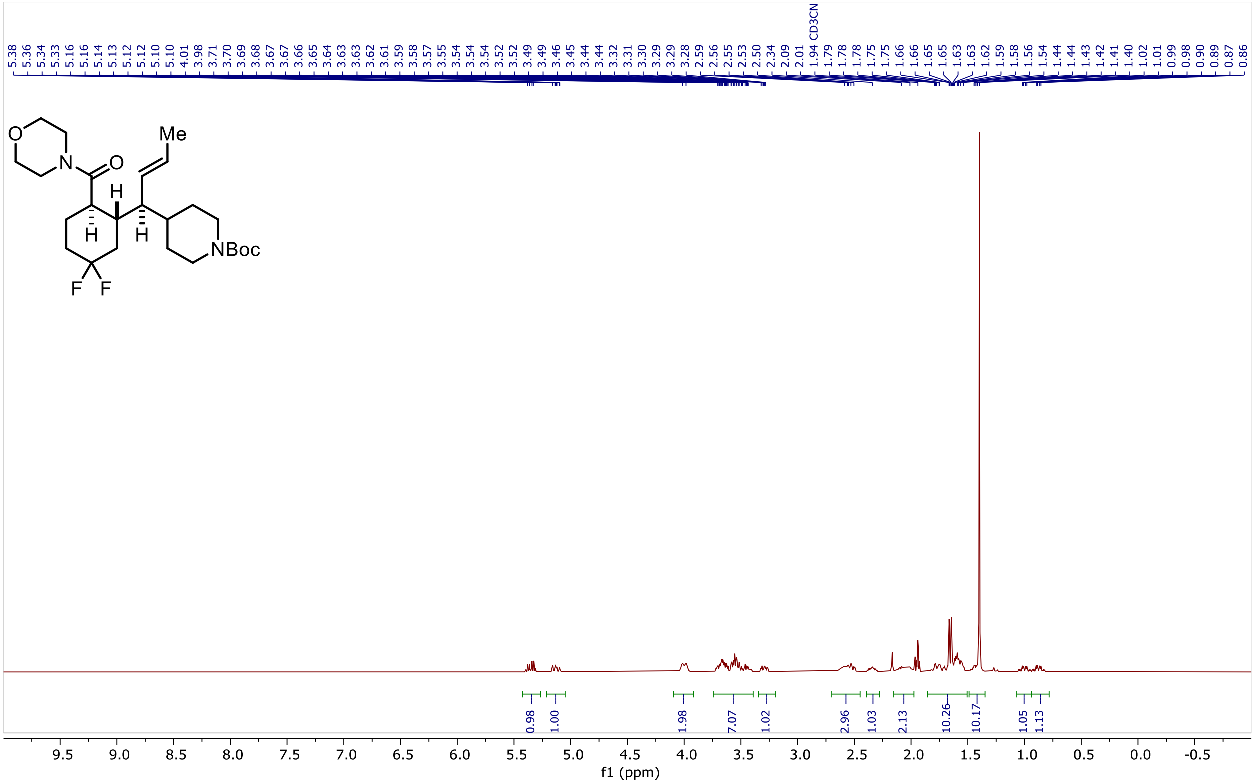

^{13}C NMR (101 MHz, CD_3CN) of 6h

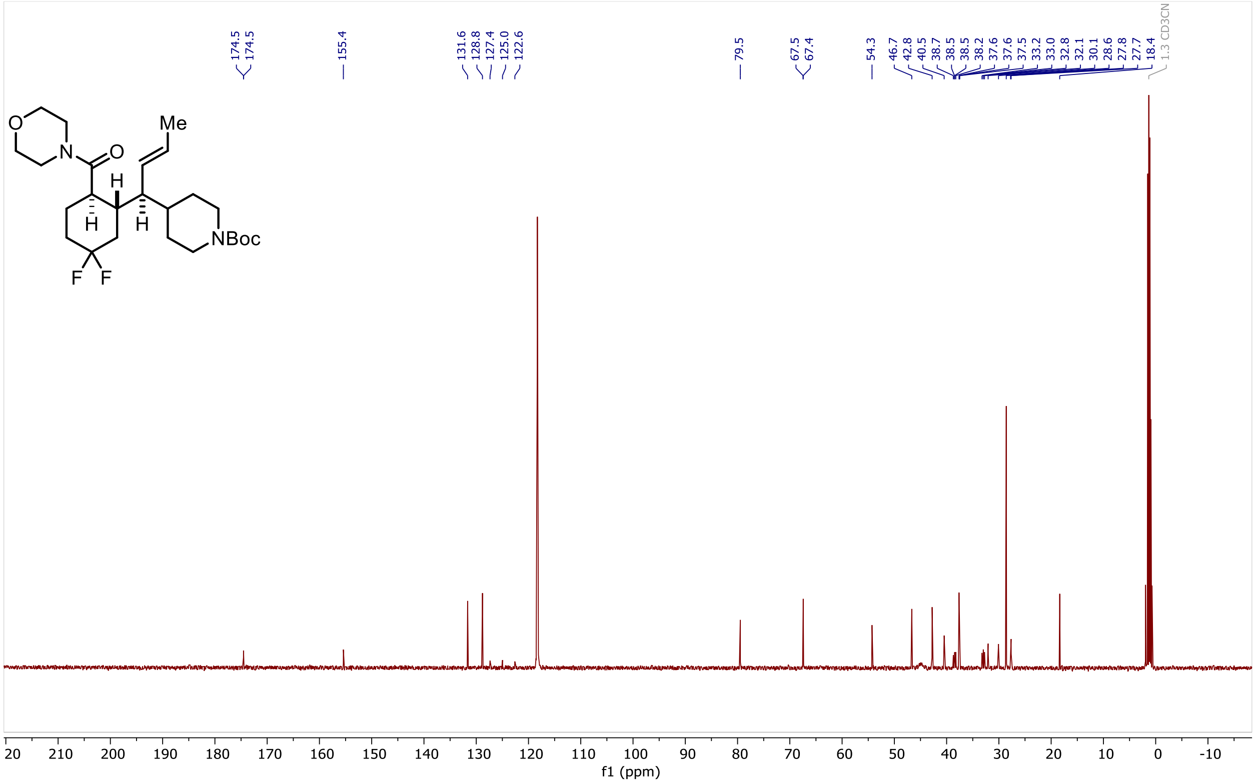

^{19}F NMR (377 MHz, $CDCl_3$) of 6h

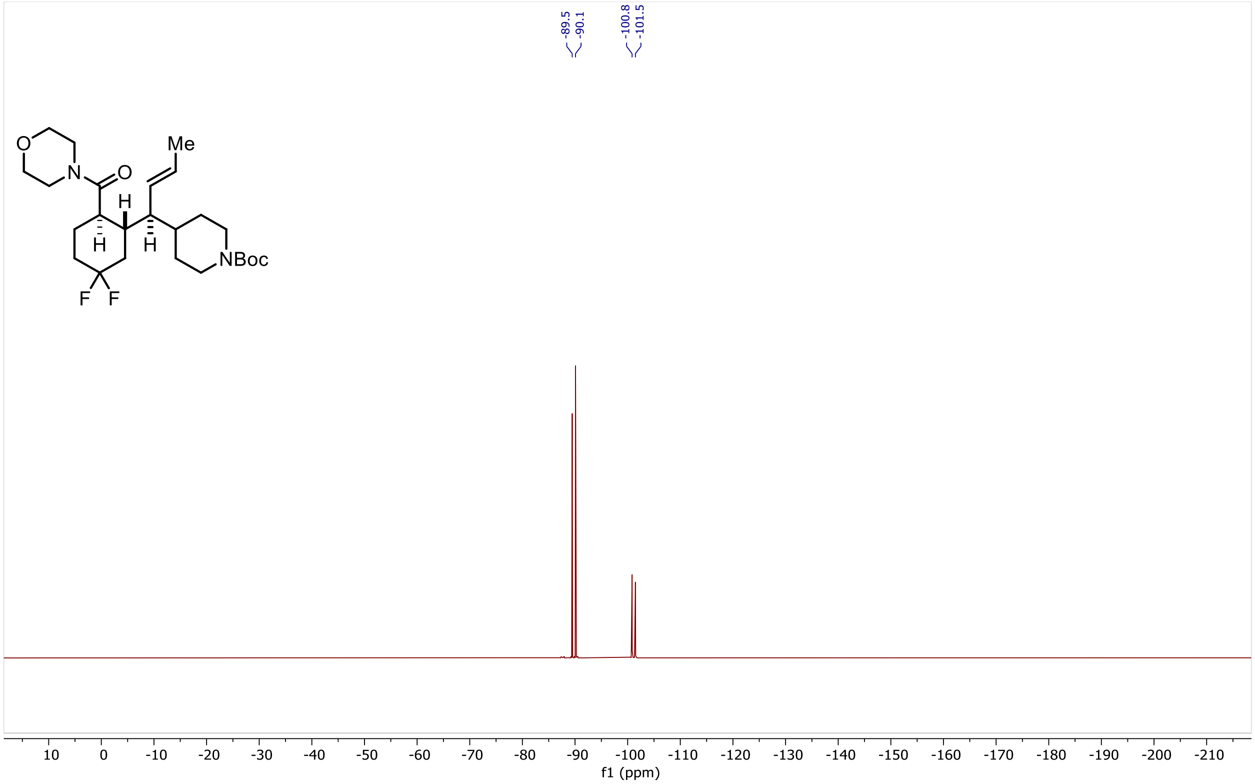

^{1}H NMR (400 MHz, CD_3CN) of 6i

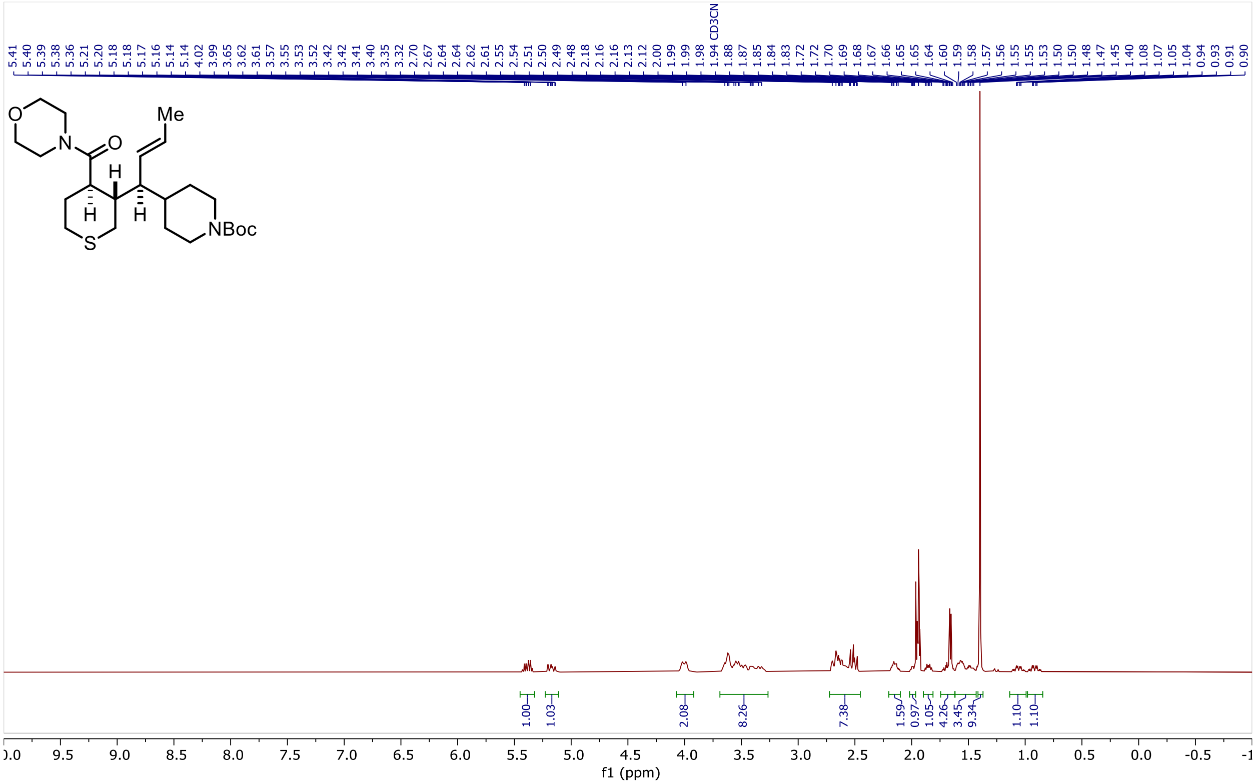

^{13}C NMR (101 MHz, CD_3CN) of 6i

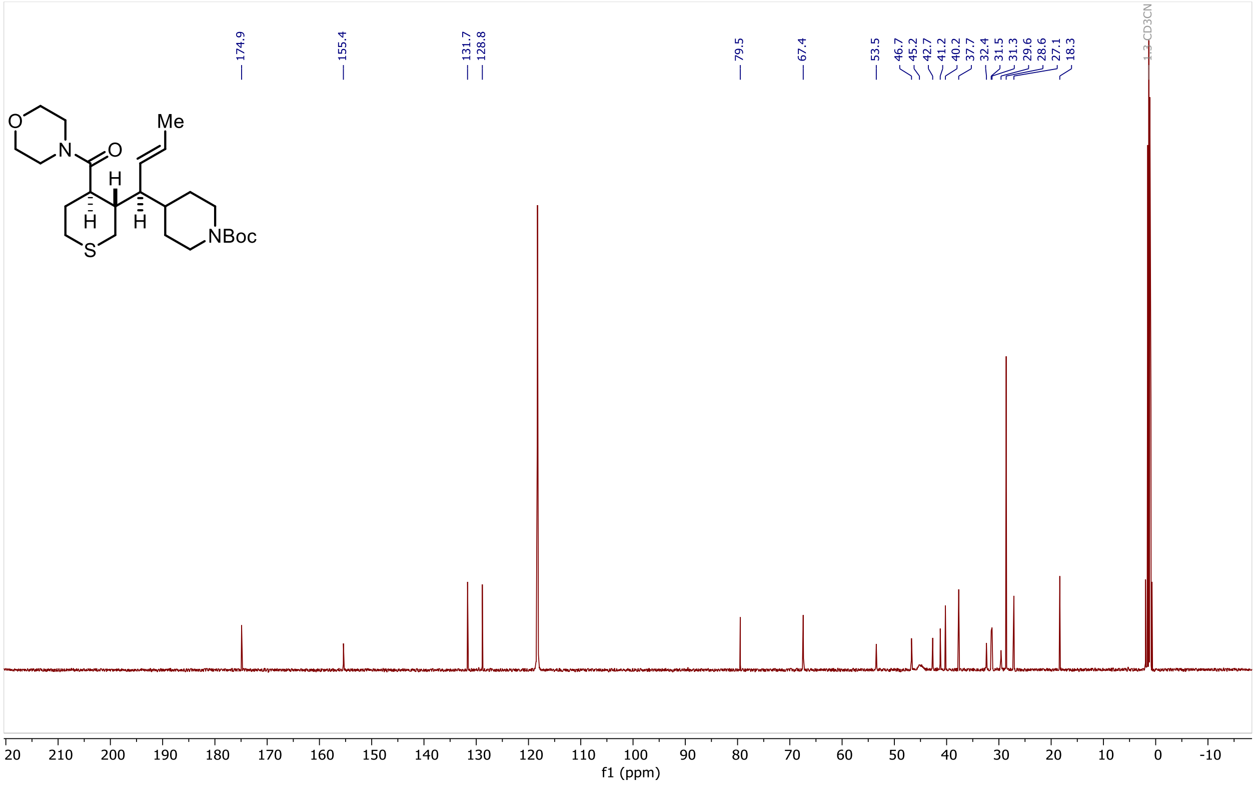

^{1}H NMR (600 MHz, DMSO-*d*$_6$, 353 K) of 6j

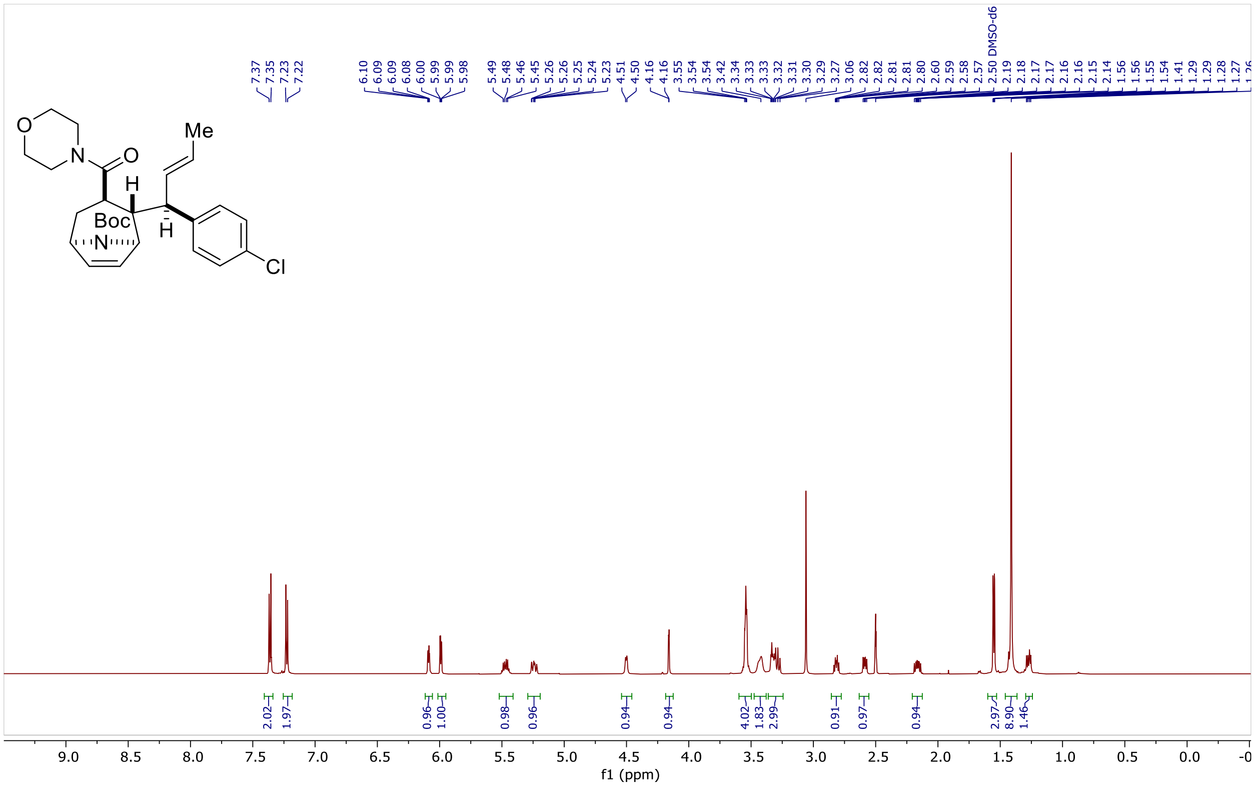

^{13}C NMR (151 MHz, DMSO- *d*$_6$, 353 K) of 6j

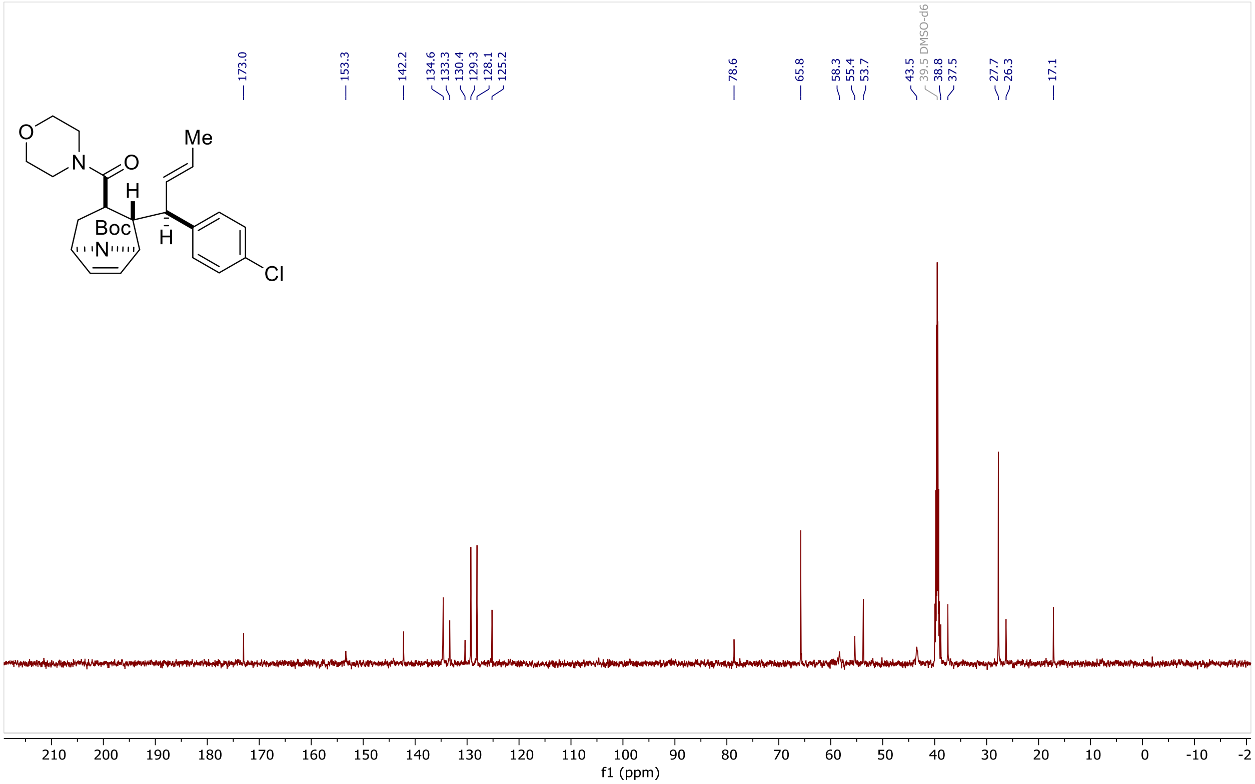

^{1}H NMR (500 MHz, DMSO-d_6, 373 K) of 9

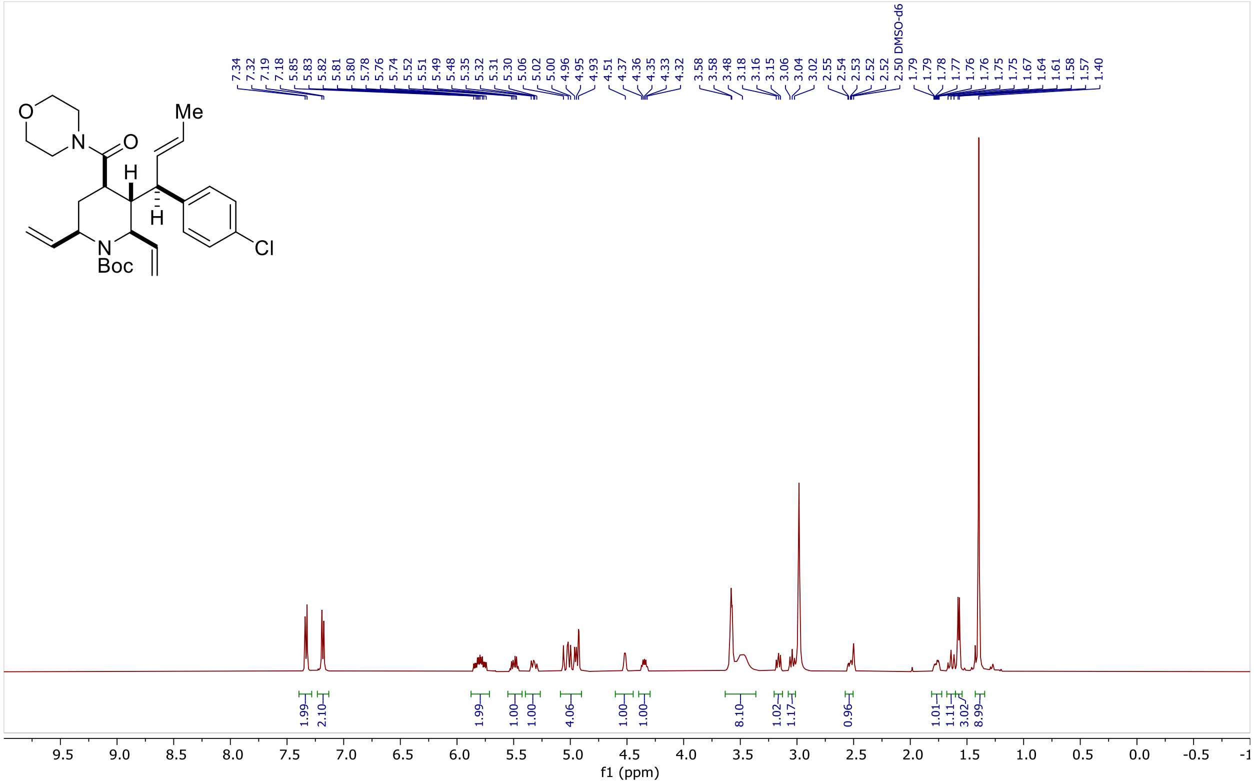

^{13}C NMR (126 MHz, DMSO-d_6, 373 K) of 9

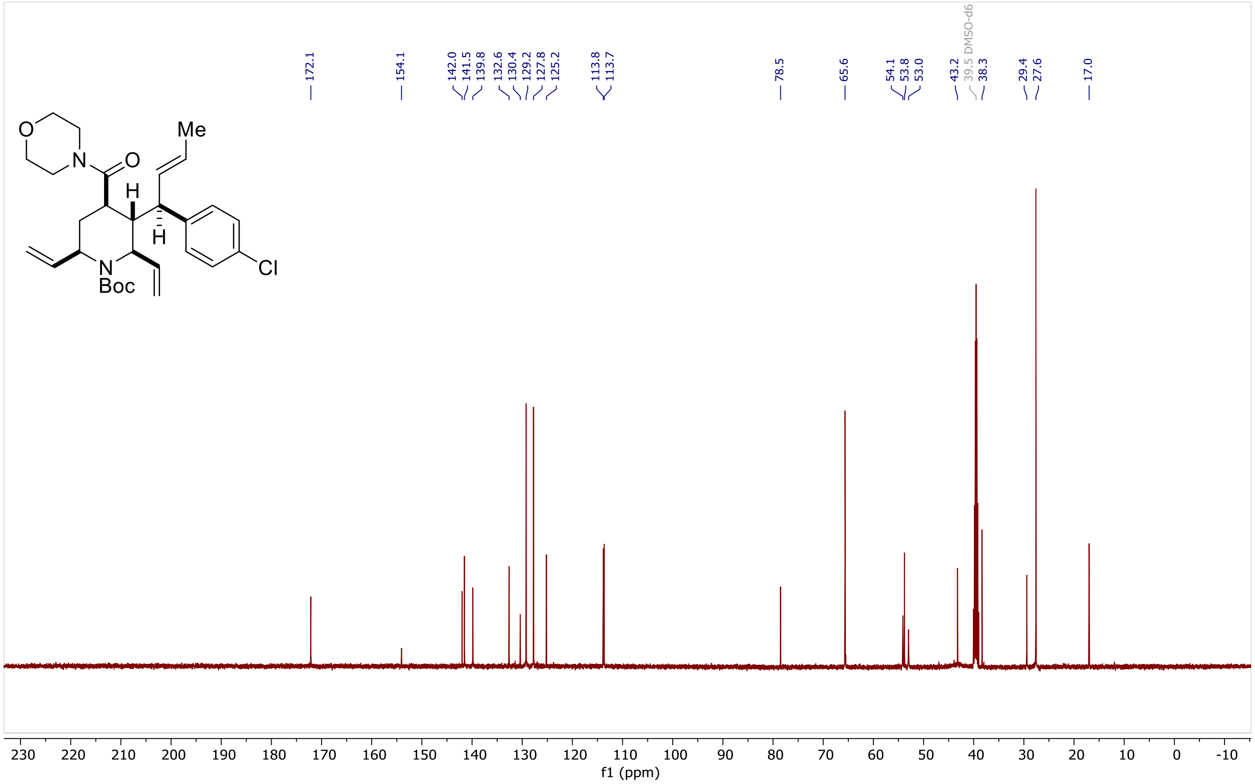

^{1}H NMR (600 MHz, CDCl$_3$) of **7**

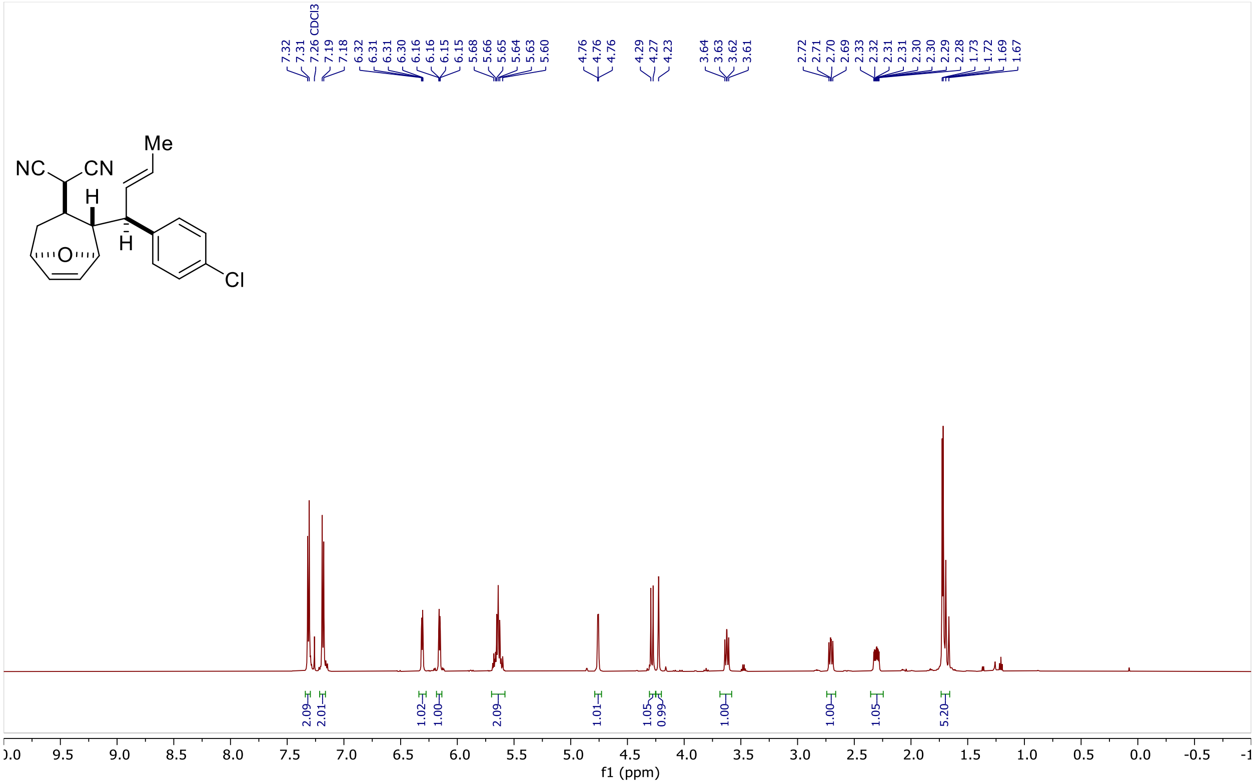

^{13}C NMR (151 MHz, CDCl$_3$) of **7**

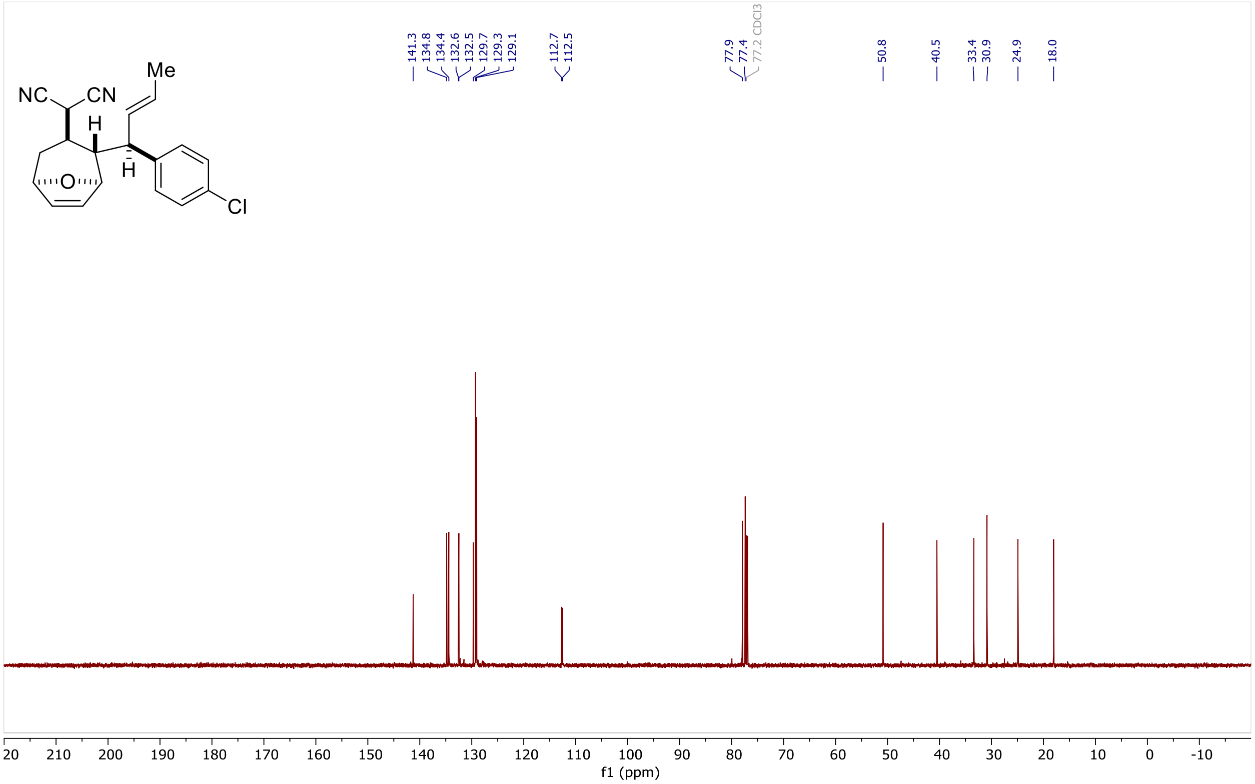

^{1}H NMR (400 MHz, CDCl$_3$) of 8

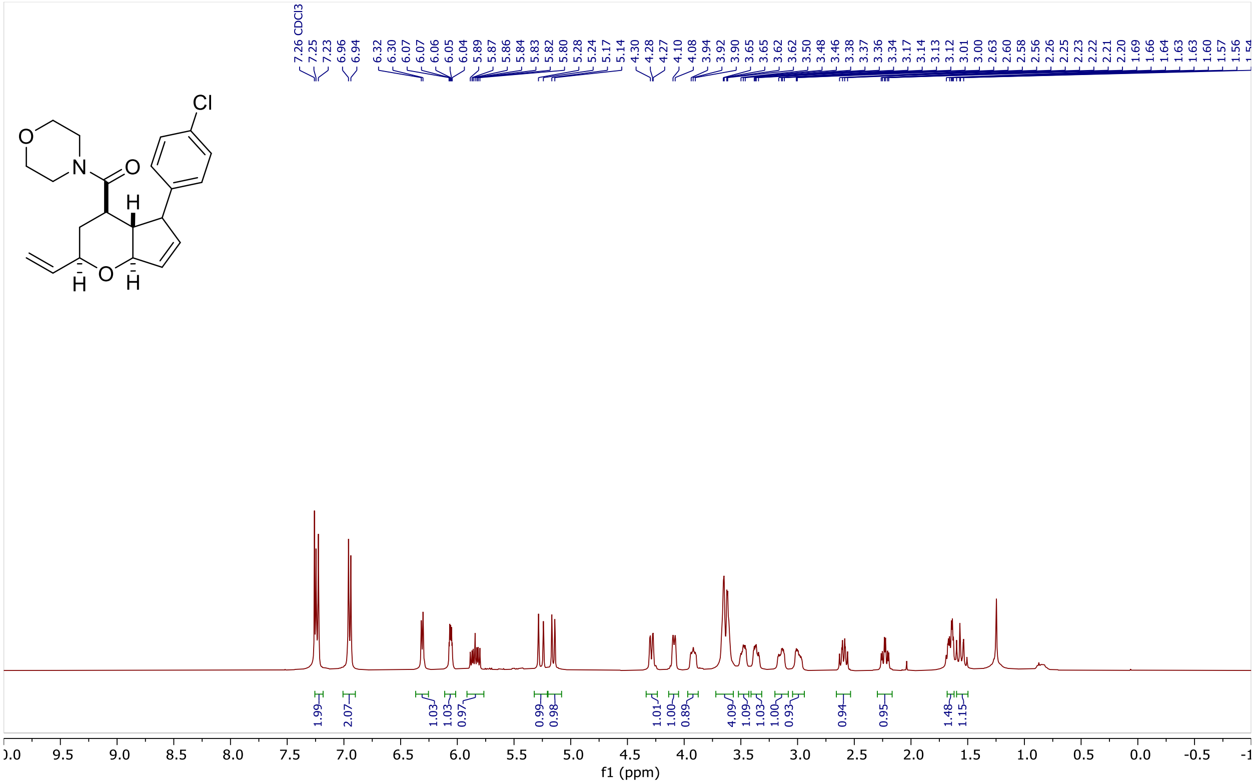

^{13}C NMR (151 MHz, CDCl$_3$) of 8

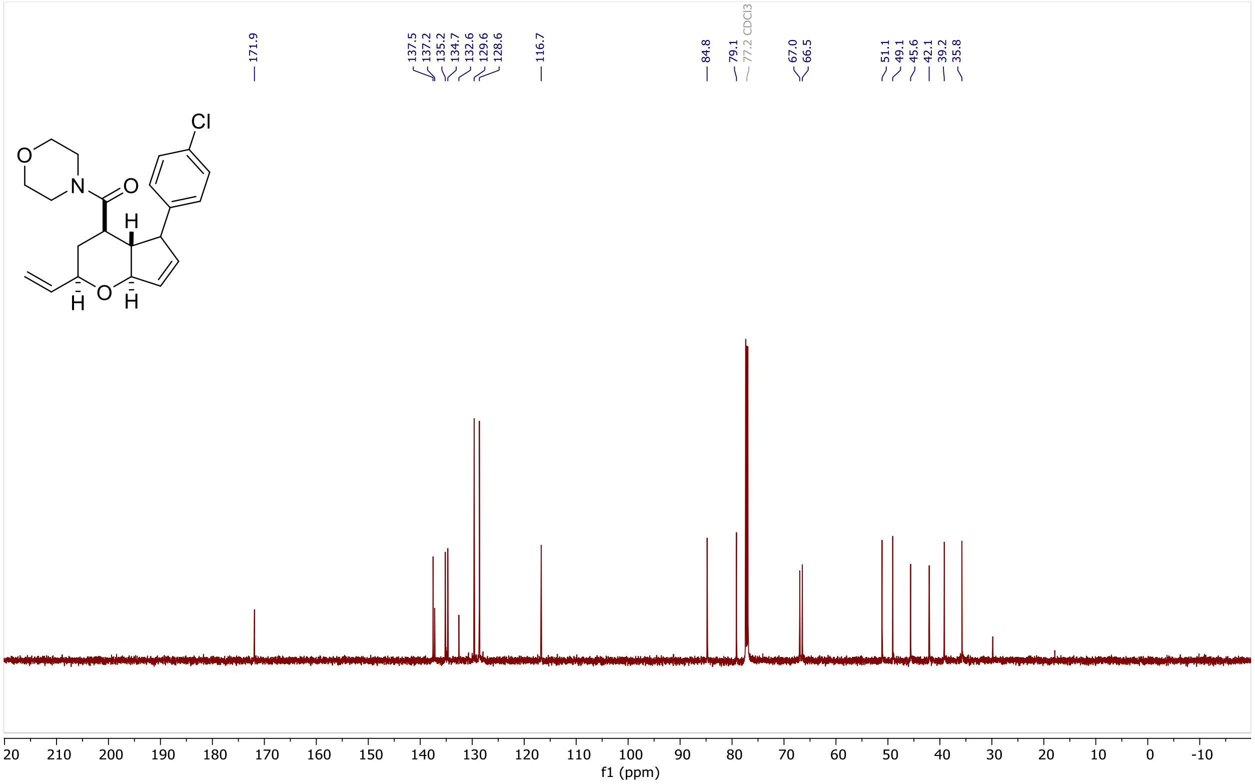

^{1}H NMR (400 MHz, CDCl$_3$) of 10b

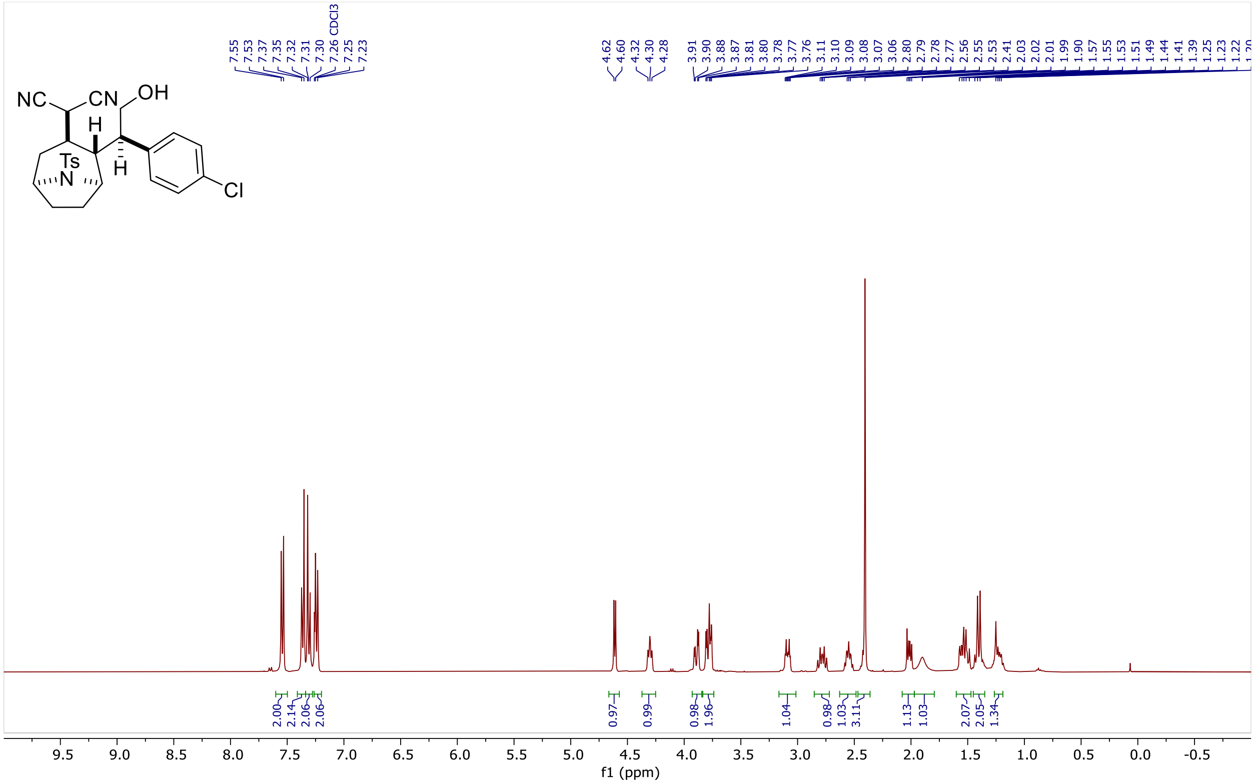

^{13}C NMR (101 MHz, CDCl$_3$) of 10b

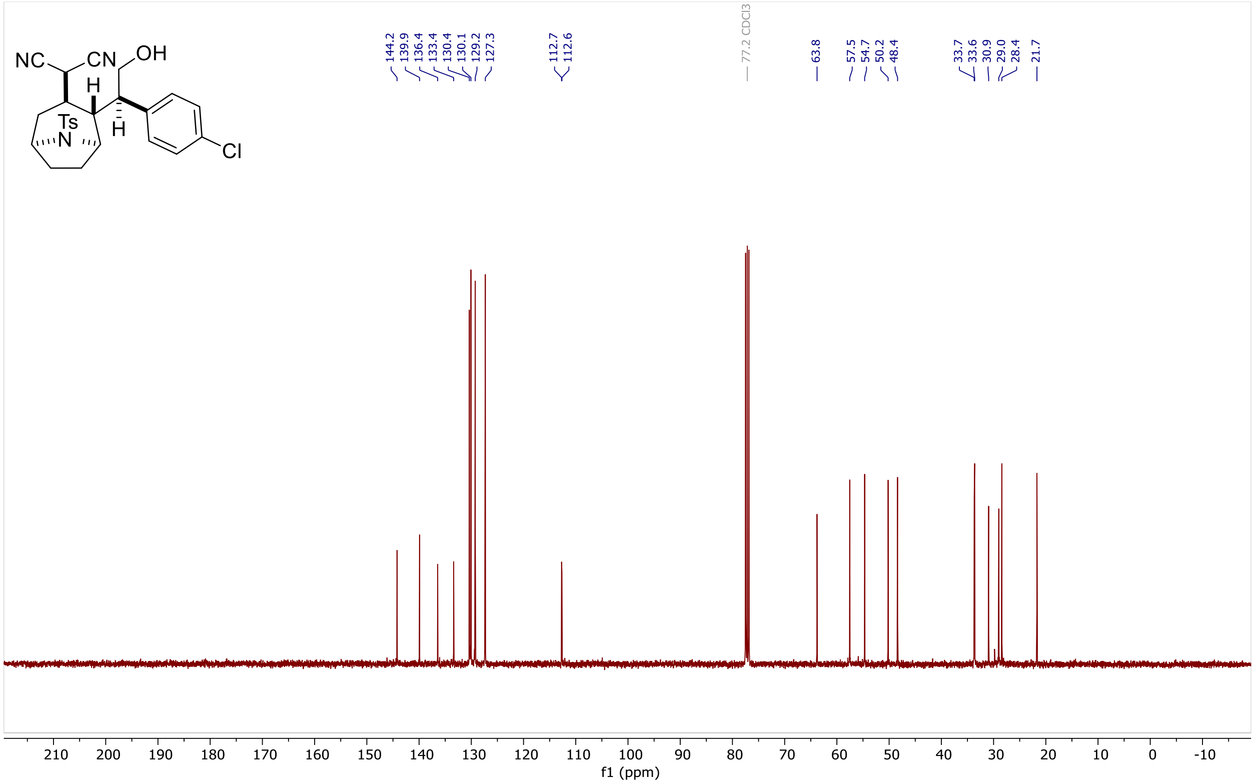

^{1}H NMR (400 MHz, CDCl$_3$) of **10c**

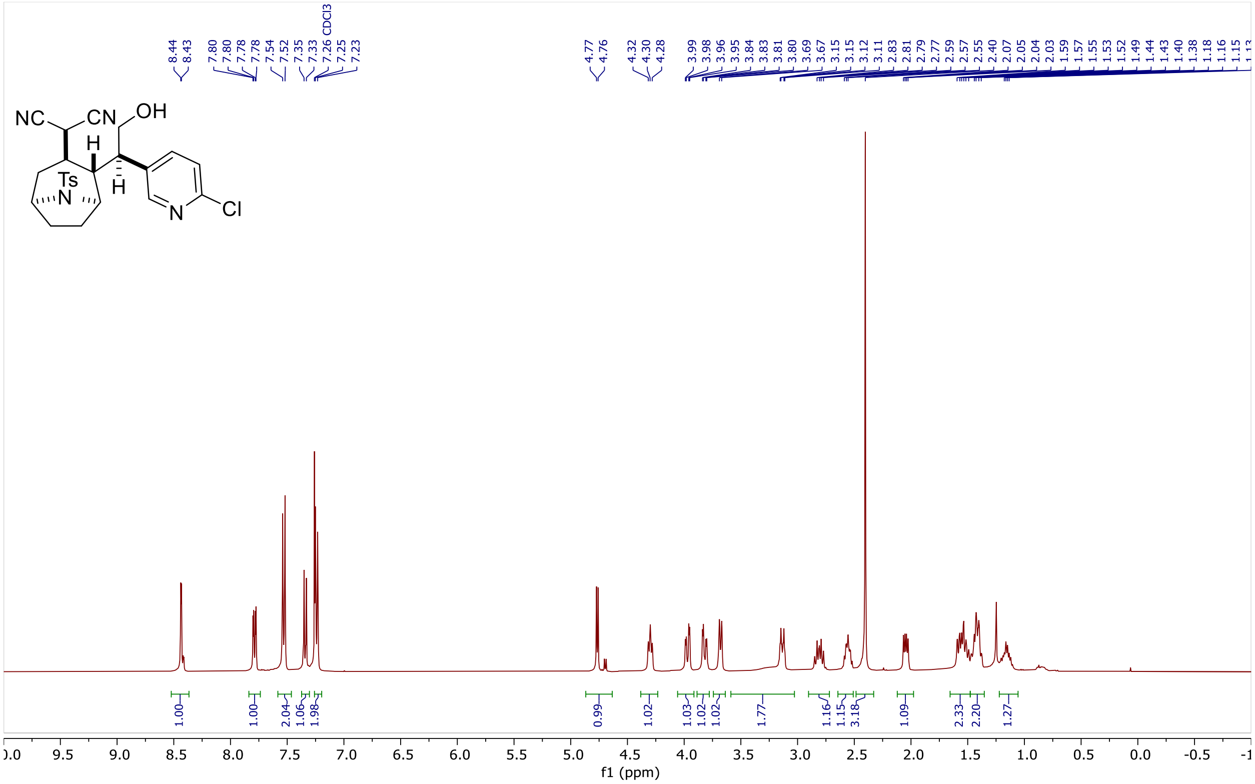

^{13}C NMR (101 MHz, CDCl$_3$) of **10c**

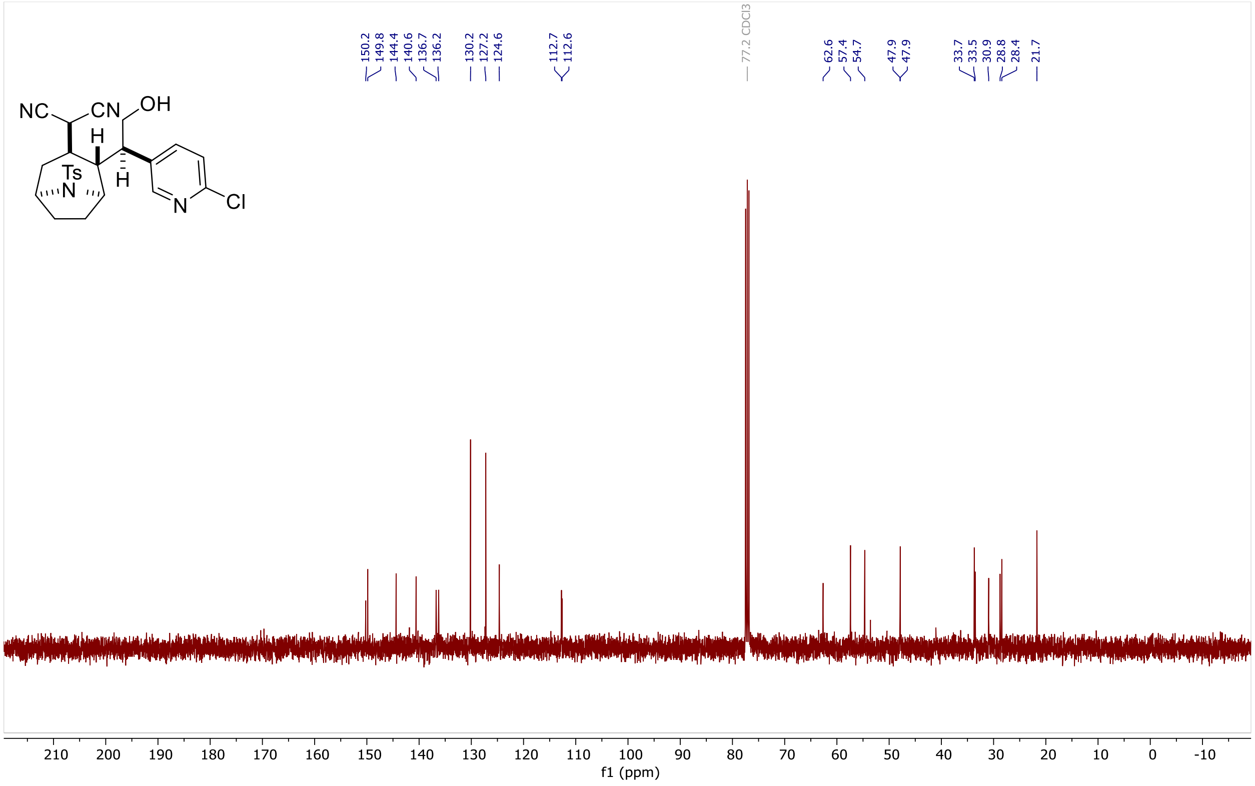

^{1}H NMR (400 MHz, CD_3CN) of 11a

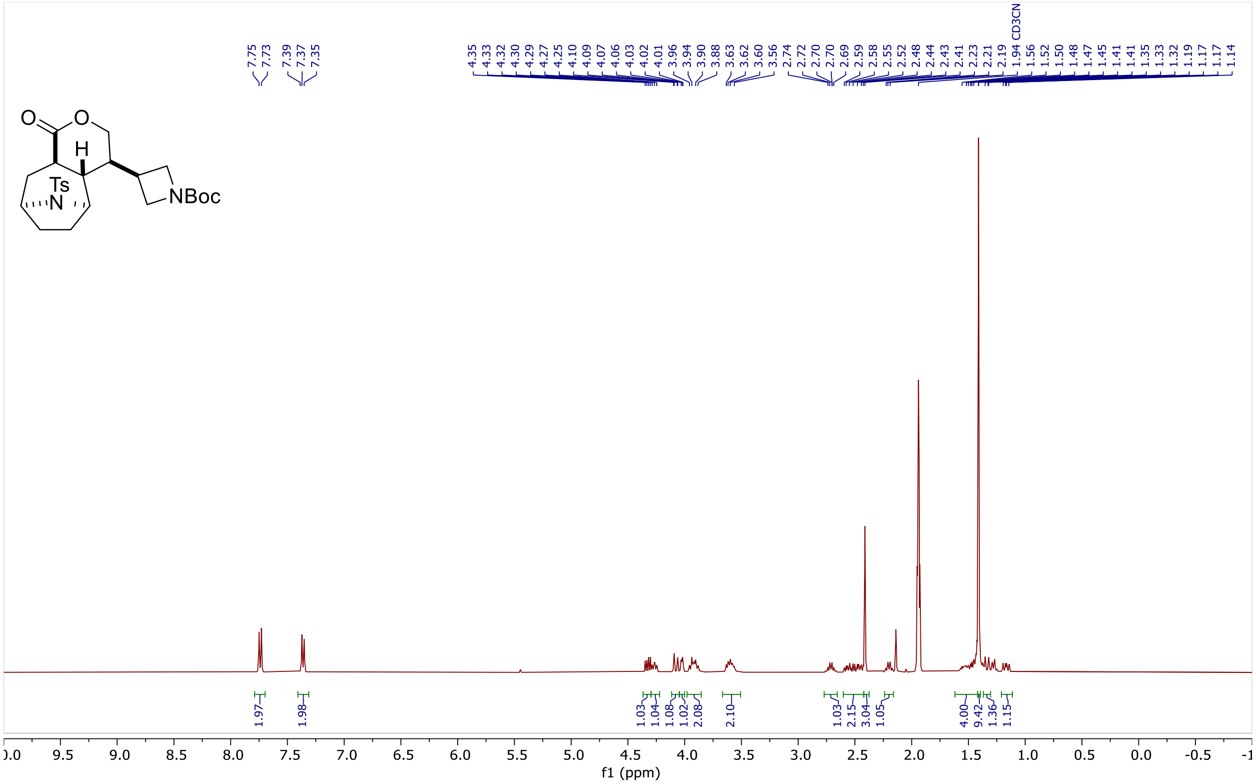

^{13}C NMR (101 MHz, CD_3CN) of 11a

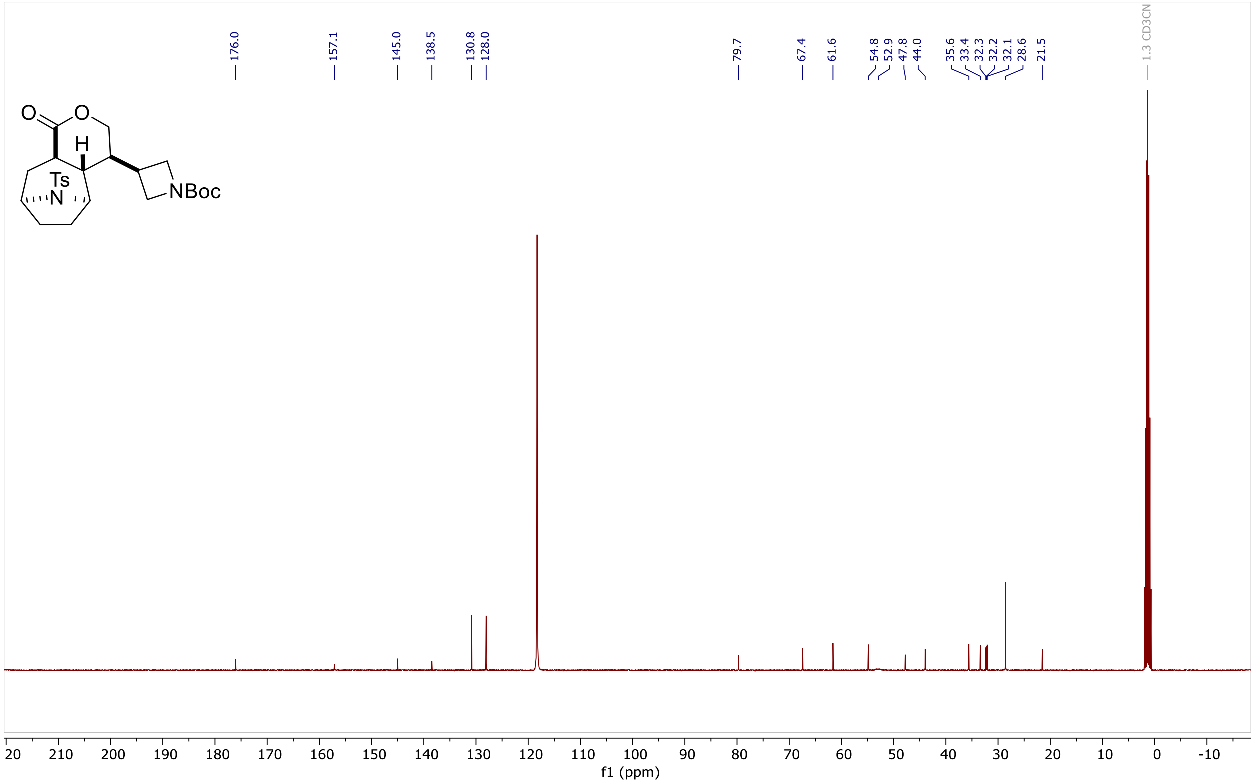

^{1}H NMR (400 MHz, $CDCl_3$) of **11b**

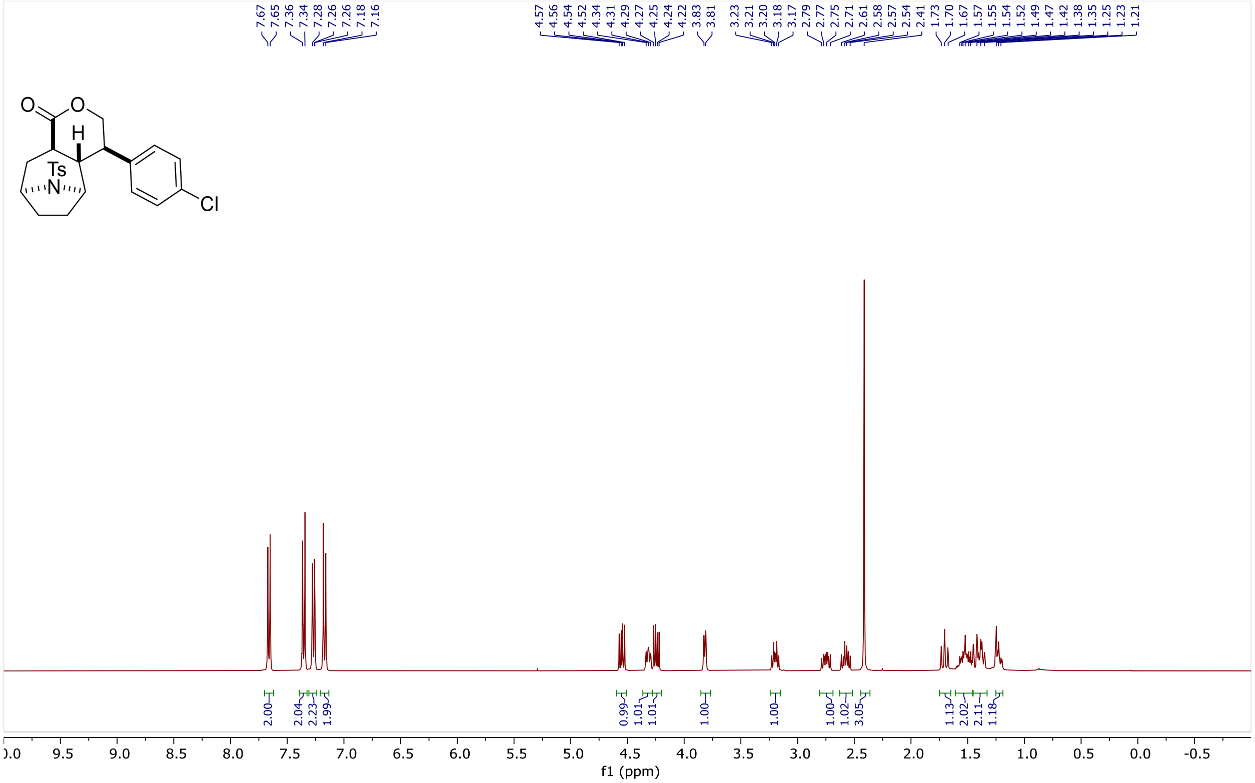

^{13}C NMR (151 MHz, $CDCl_3$) of **11b**

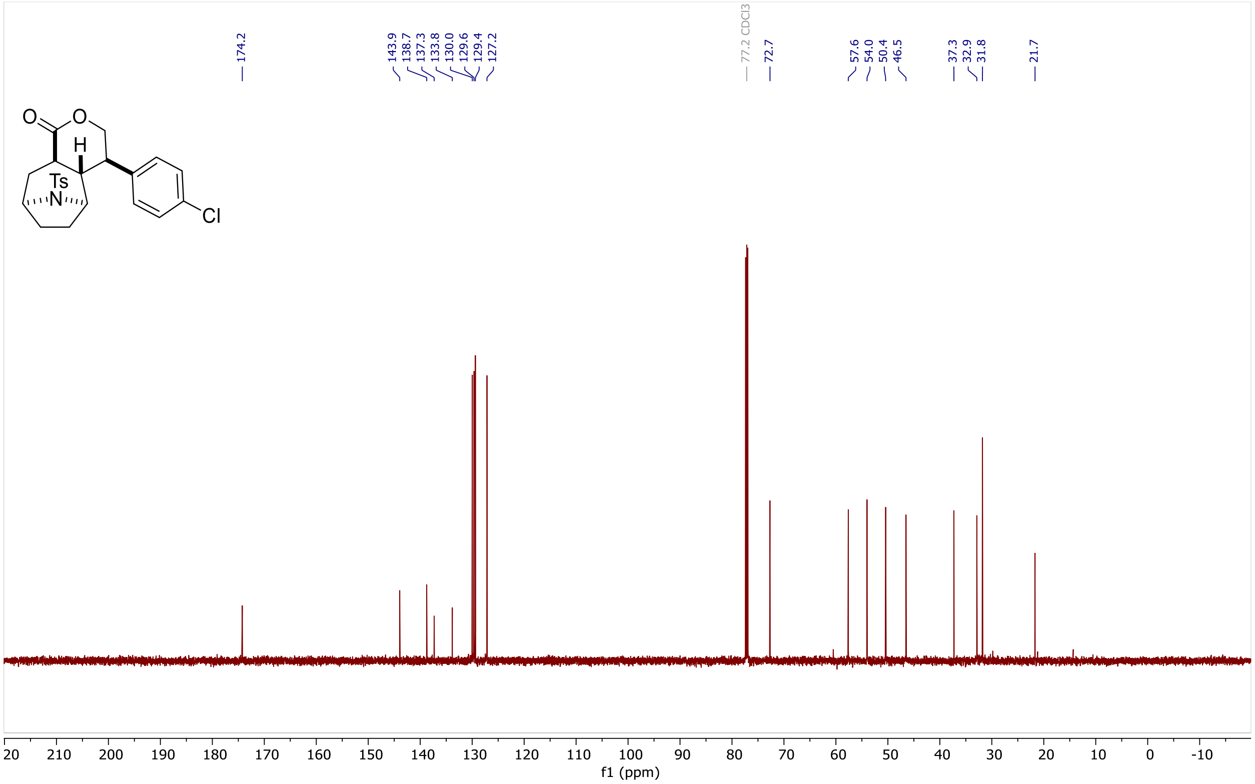

^{1}H NMR (400 MHz, CDCl$_3$) of 11c

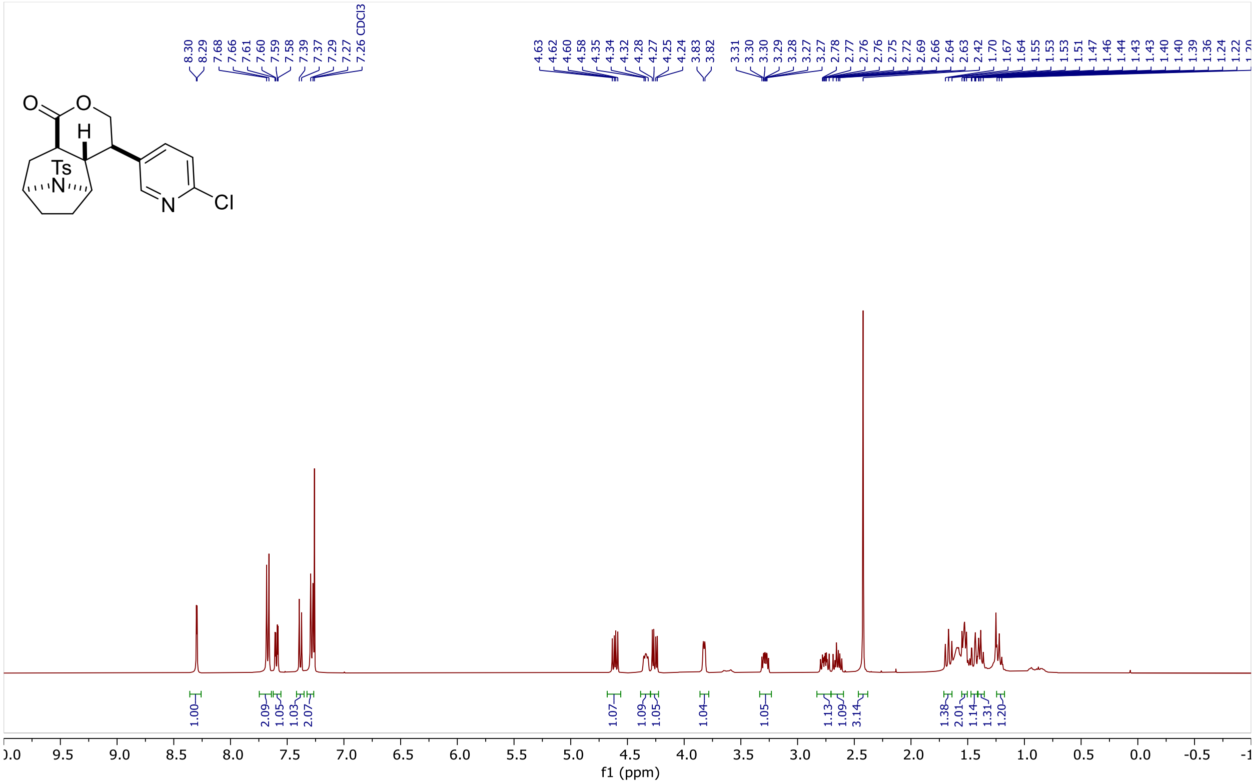

^{13}C NMR (101 MHz, CDCl$_3$) of 11c

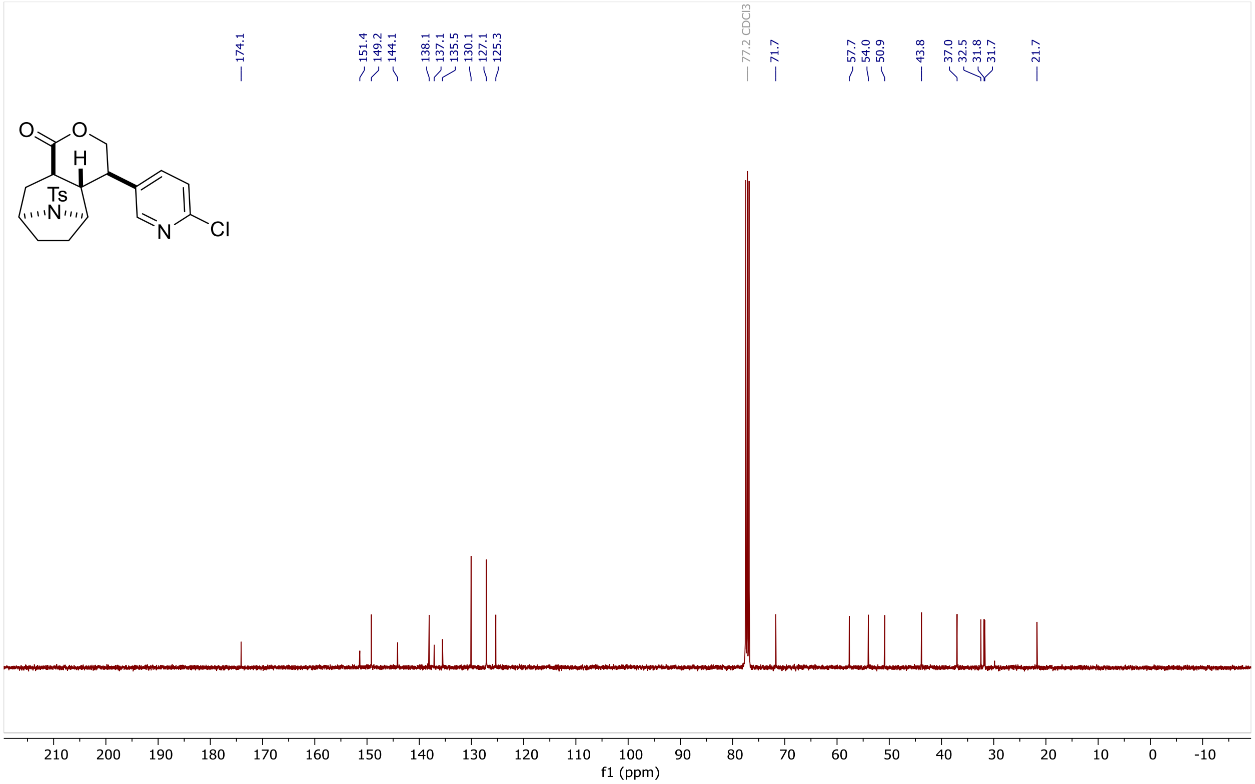

HPLC data

Compound 2a

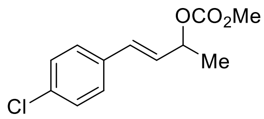

Chiral-racemic

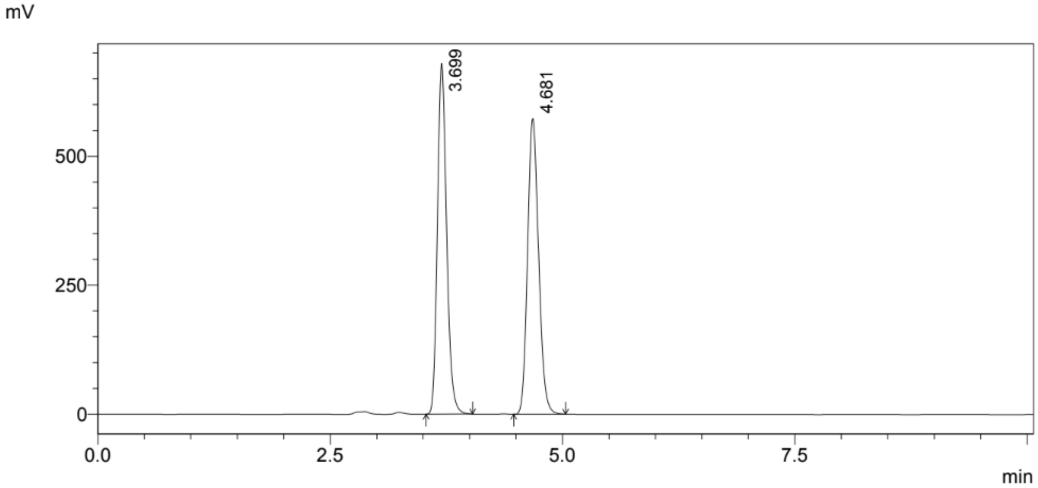

Detector A Channel 1 254nm

Peak#	Ret. Time	Area%
1	3.699	50.069
2	4.681	49.931
Total		100.000

Chiral
non-racemic

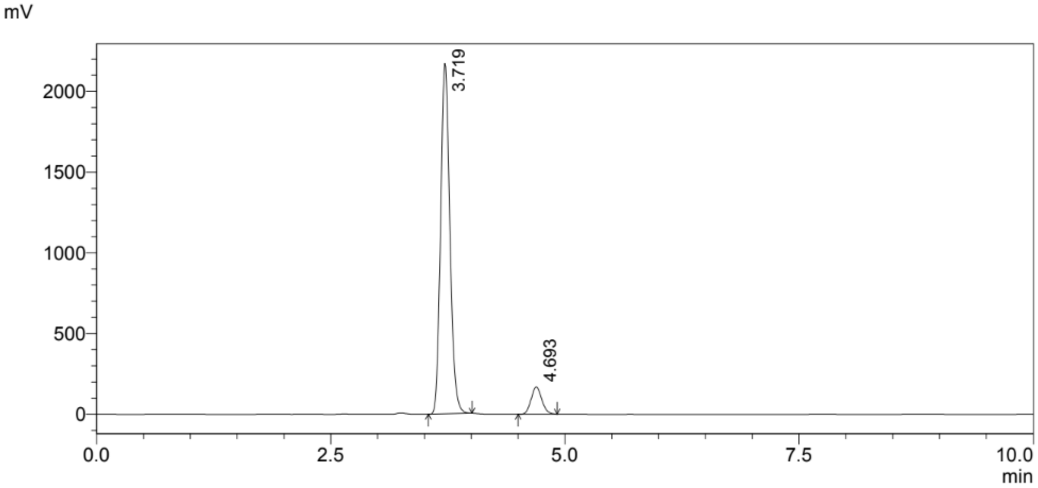

Detector A Channel 1 254nm

Peak#	Ret. Time	Area%
1	3.719	91.733
2	4.693	8.267
Total		100.000

Compound 4a

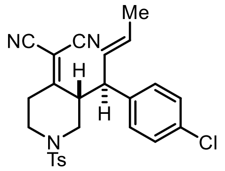

Chiral-racemic

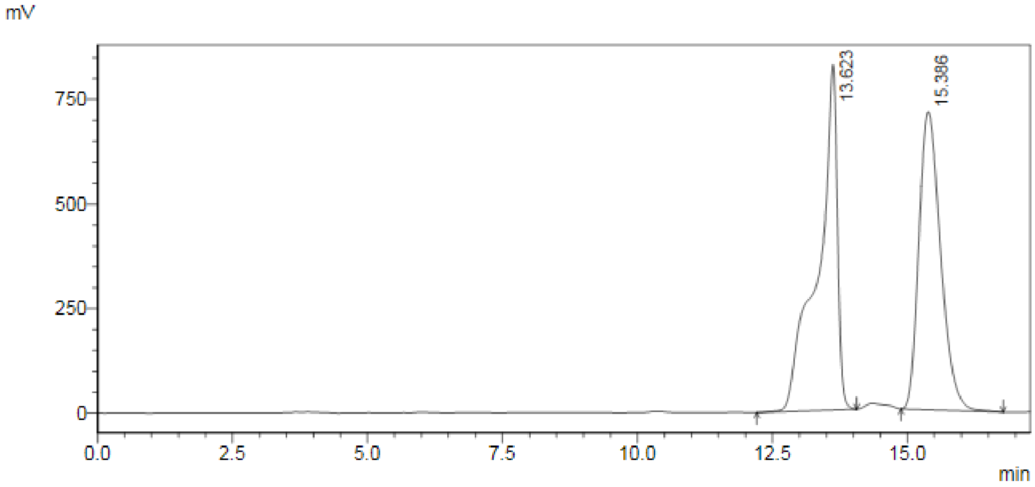

Detector A Channel 1 254nm

Peak#	Ret. Time	Area%
1	13.623	50.740
2	15.386	49.260
Total		100.000

Chiral
non-racemic
using
***rac*-2a**

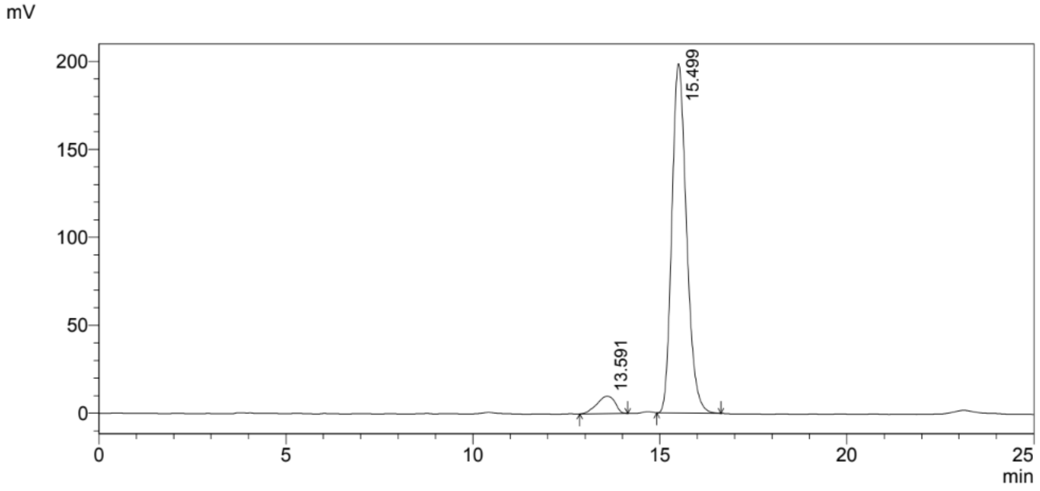

Detector A Channel 1 254nm

Peak#	Ret. Time	Area%
1	13.591	5.817
2	15.499	94.183
Total		100.000

Chiral non-racemic using ***rac*-2aAc** and DIPEA

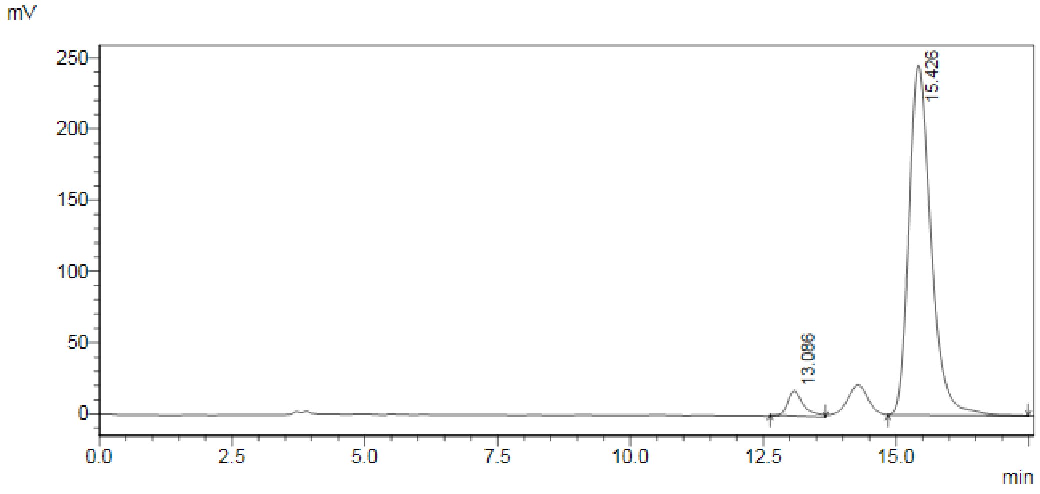

Detector A Channel 1 254nm

Peak#	Ret. Time	Area%
1	13.086	4.881
2	15.426	95.119
Total		100.000

Chiral non-racemic using ***rac*-2a-Ac** and K_3PO_4

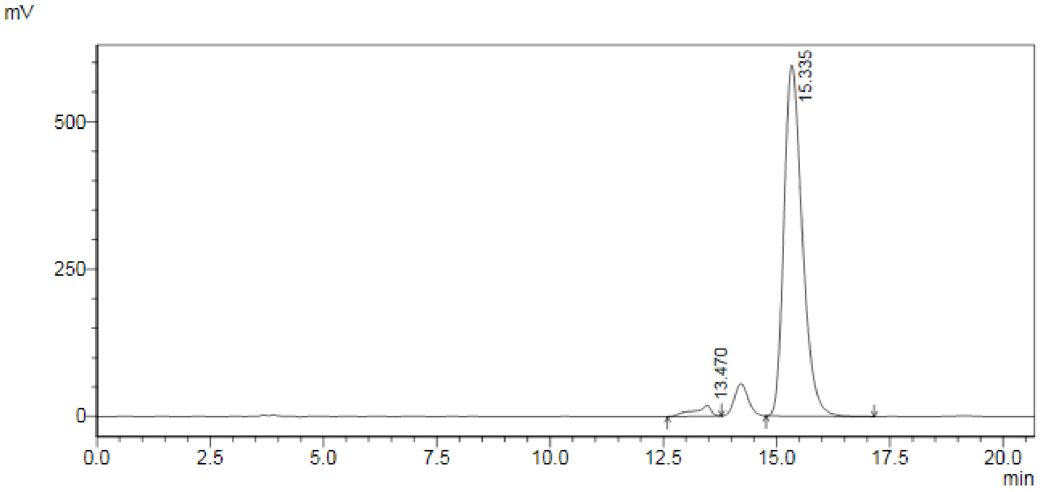

Detector A Channel 1 254nm

Peak#	Ret. Time	Area%
1	13.470	2.902
2	15.335	97.098
Total		100.000

Compound 4b

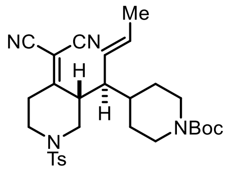

Chiral-racemic

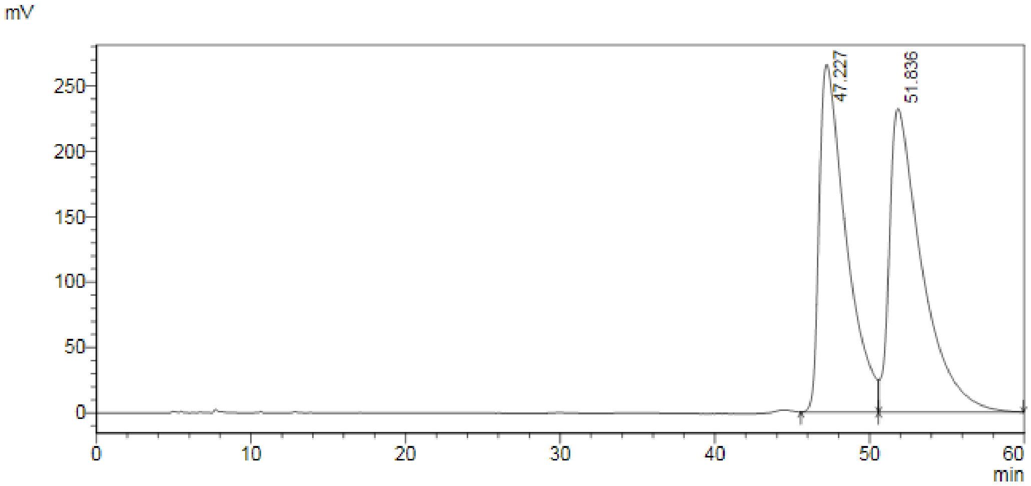

Detector A Channel 1 254nm

Peak#	Ret. Time	Area%
1	47.227	48.445
2	51.836	51.555
Total		100.000

Chiral non-racemic

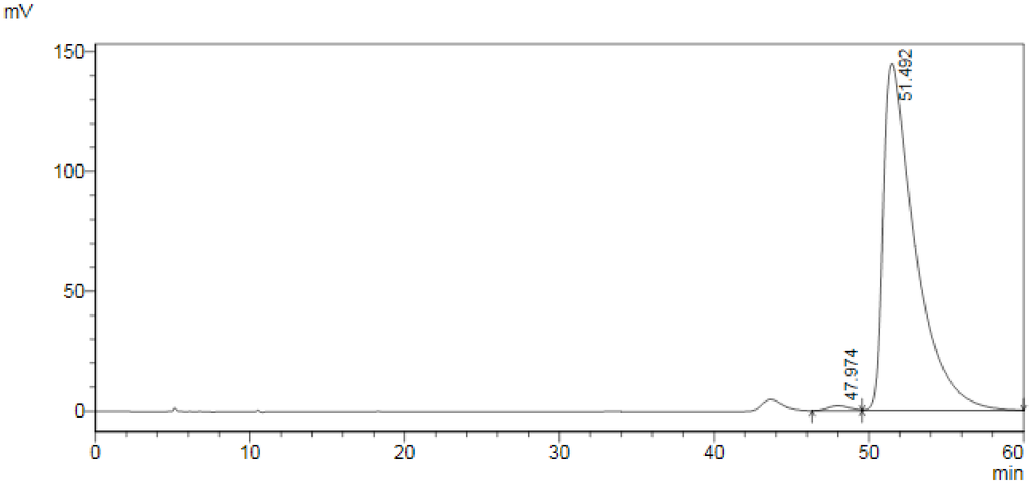

Detector A Channel 1 254nm

Peak#	Ret. Time	Area%
1	47.974	1.023
2	51.492	98.977
Total		100.000

Chiral
non-racemic,
3 mmol scale

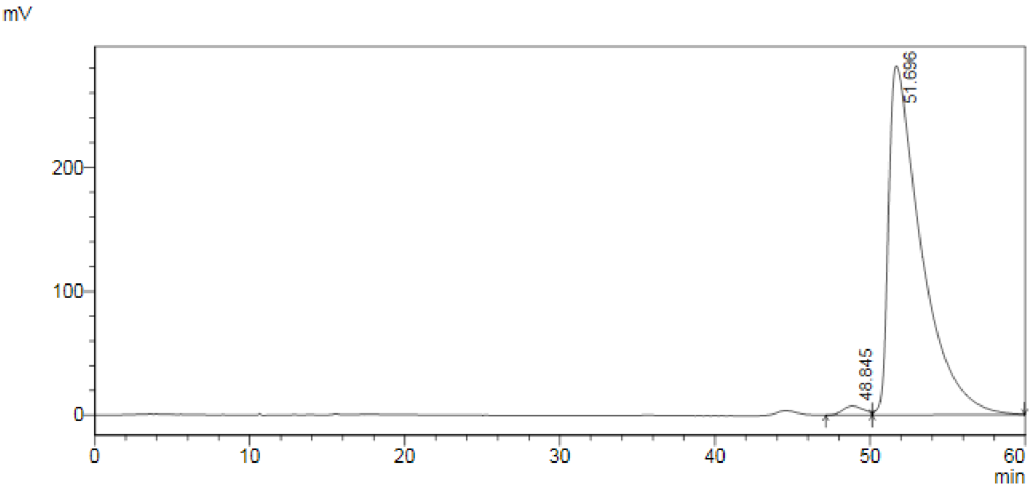

Detector A Channel 1 254nm

Peak#	Ret. Time	Area%
1	48.845	1.665
2	51.696	98.335
Total		100.000

Chiral
non-racemic,
3 mmol scale,
0.5 mol% Pd

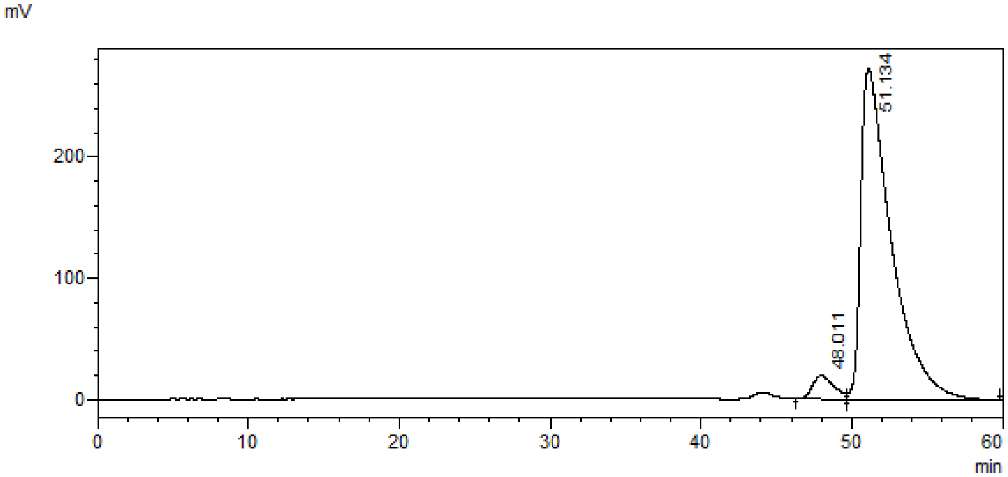

Detector A Channel 1 254nm

Peak#	Ret. Time	Area%
1	48.011	4.805
2	51.134	95.195
Total		100.000

Compound 4c

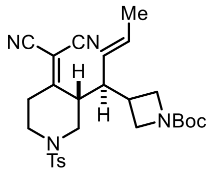

Chiral-racemic

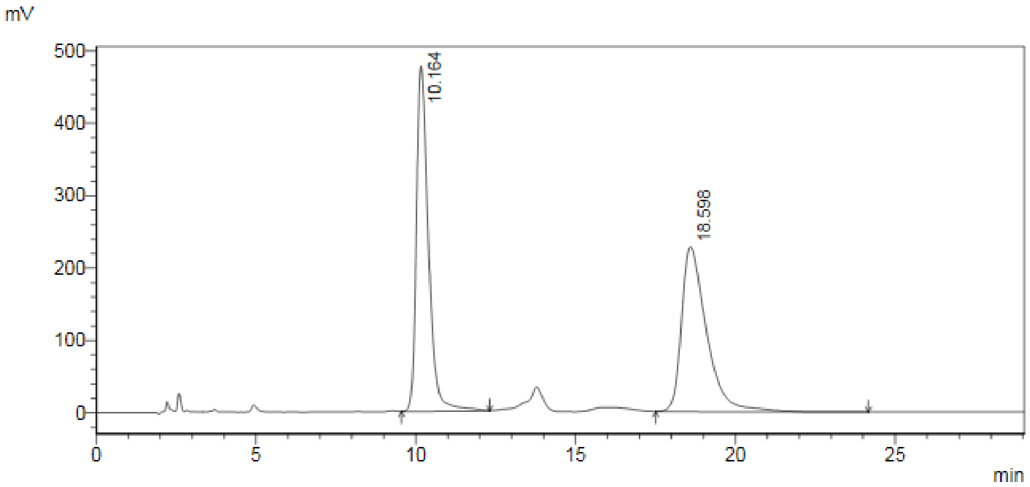

Detector A Channel 1 254nm

Peak#	Ret. Time	Area%
1	10.164	50.132
2	18.598	49.868
Total		100.000

Chiral
non-racemic

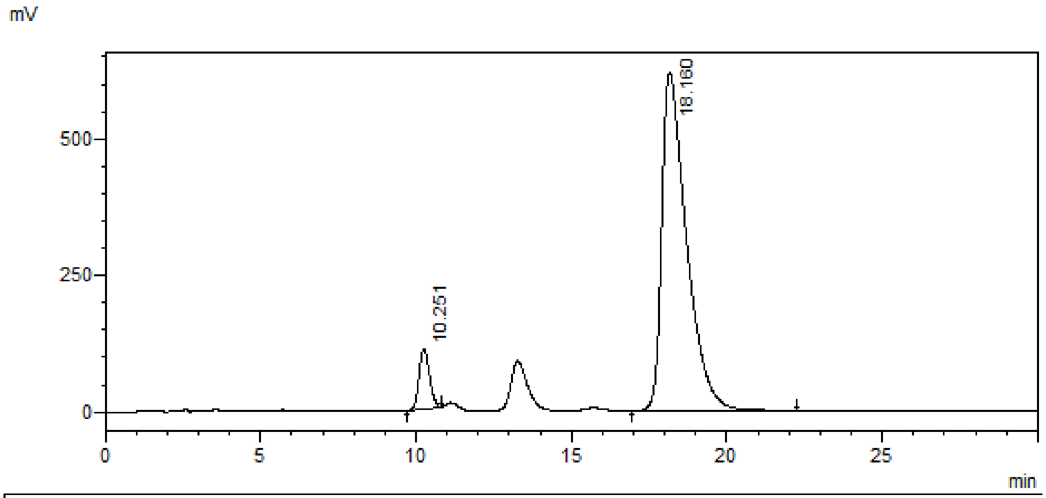

Detector A Channel 1 254nm

Peak#	Ret. Time	Area%
1	10.251	6.998
2	18.160	93.002
Total		100.000

Compound 4d

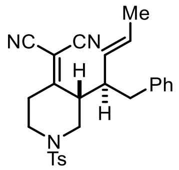

Chiral-racemic

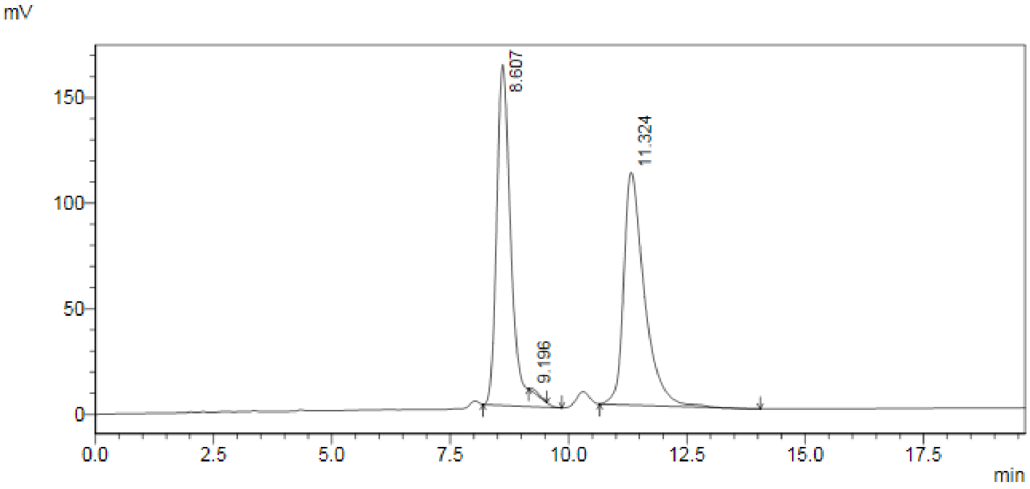

Detector A Channel 1 254nm

Peak#	Ret. Time	Area%
1	8.607	50.207
2	9.196	0.252
3	11.324	49.540
Total		100.000

Chiral
non-racemic

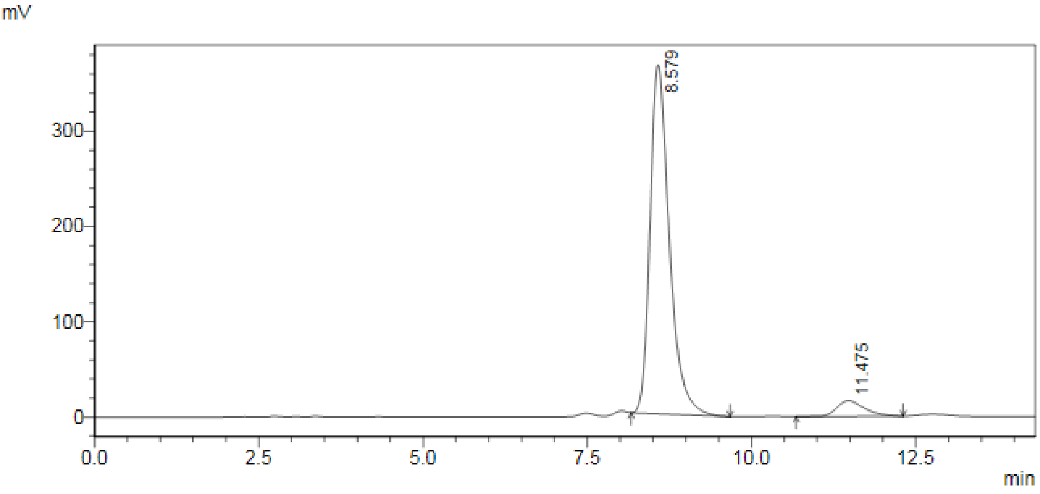

Detector A Channel 1 254nm

Peak#	Ret. Time	Area%
1	8.579	94.029
2	11.475	5.971
Total		100.000

Compound 4e

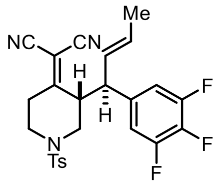

Chiral-racemic

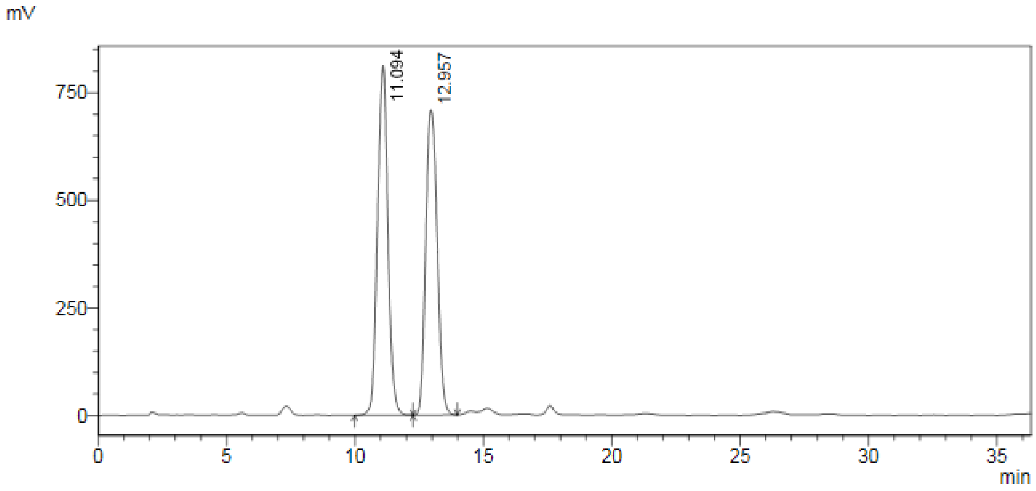

Detector A Channel 1 254nm

Peak#	Ret. Time	Area%
1	11.094	50.947
2	12.957	49.053
Total		100.000

Chiral
non-racemic

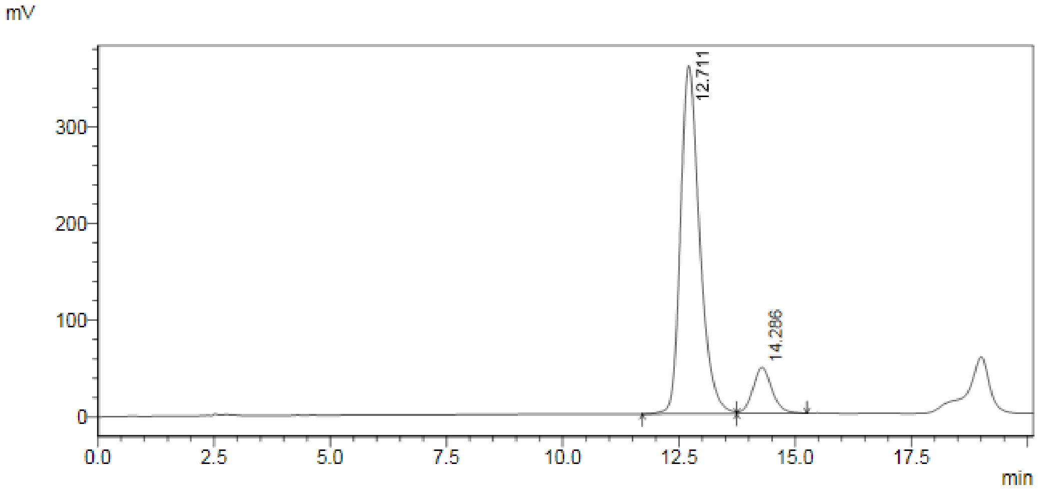

Detector A Channel 1 254nm

Peak#	Ret. Time	Area%
1	12.711	88.320
2	14.286	11.680
Total		100.000

Compound 4f

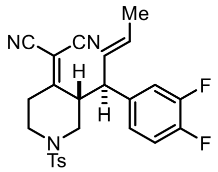

Chiral-racemic

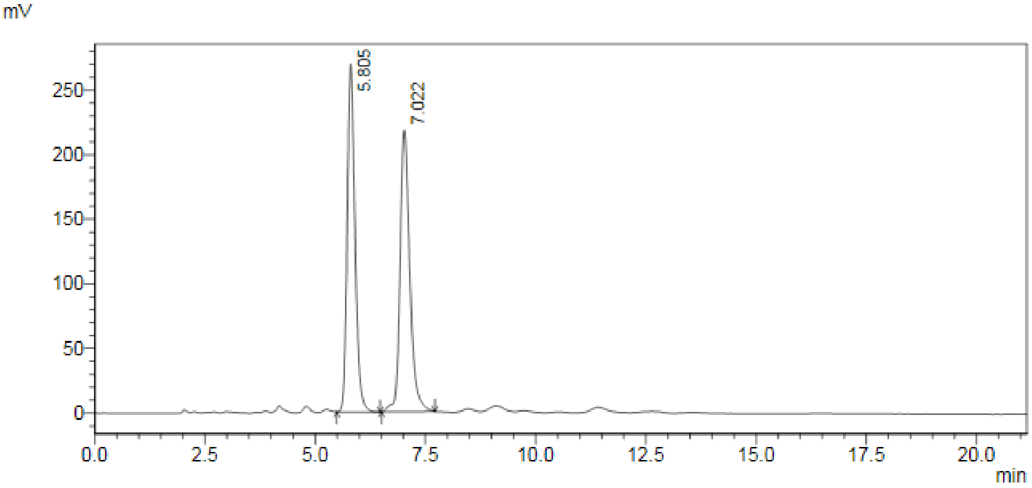

Detector A Channel 1 254nm

Peak#	Ret. Time	Area%
1	5.805	49.648
2	7.022	50.352
Total		100.000

Chiral
non-racemic

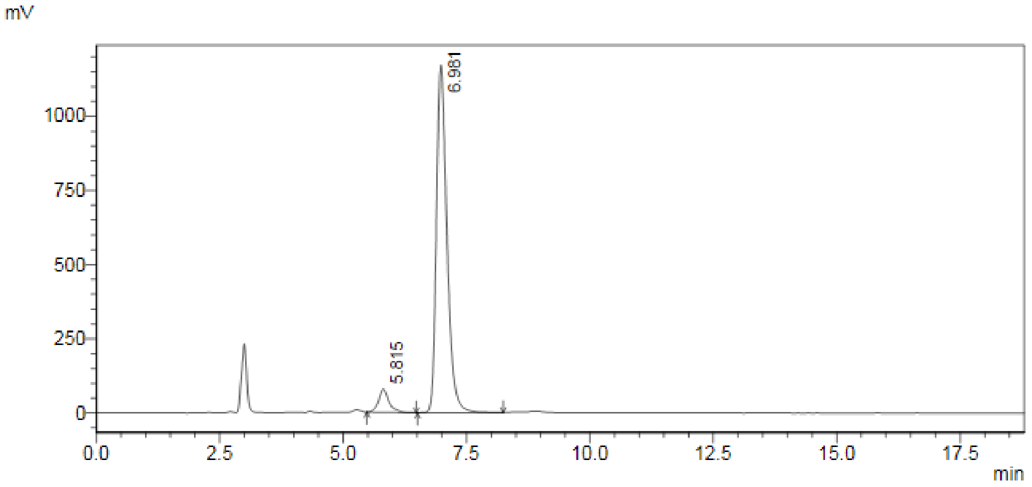

Detector A Channel 1 254nm

Peak#	Ret. Time	Area%
1	5.815	6.286
2	6.981	93.714
Total		100.000

Compound 4g

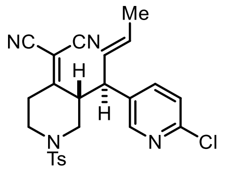

Chiral-racemic

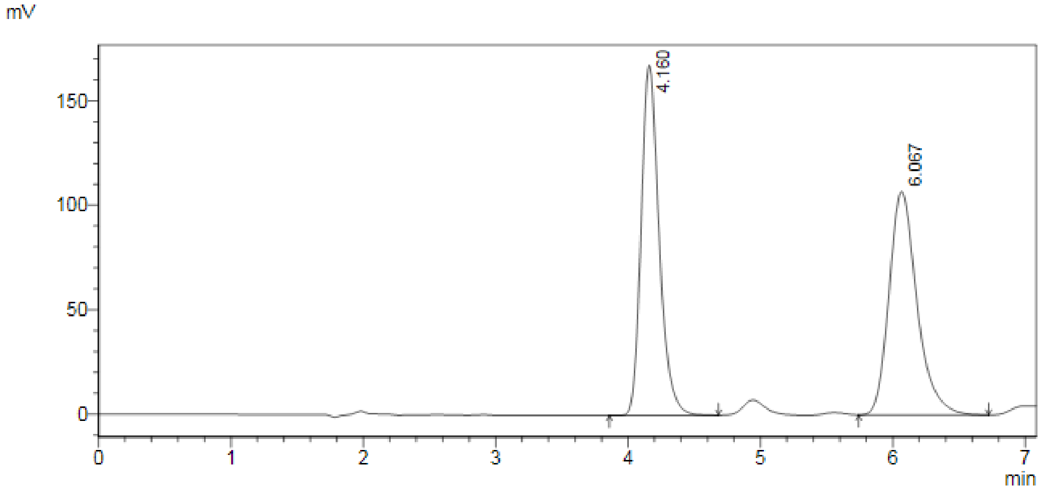

Detector A Channel 1 254nm

Peak#	Ret. Time	Area%
1	4.160	50.196
2	6.067	49.804
Total		100.000

Chiral
non-racemic

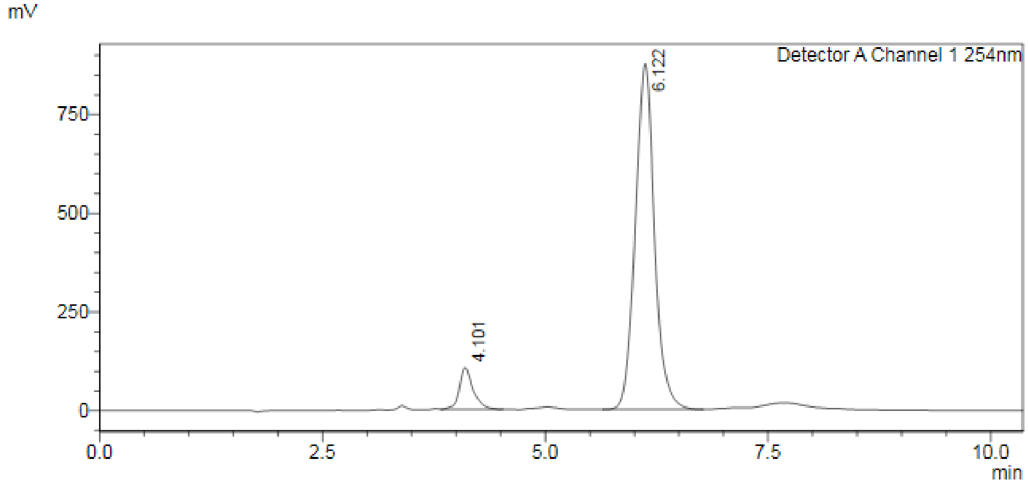

Compound 4h

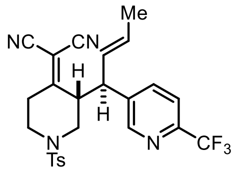

Chiral-racemic

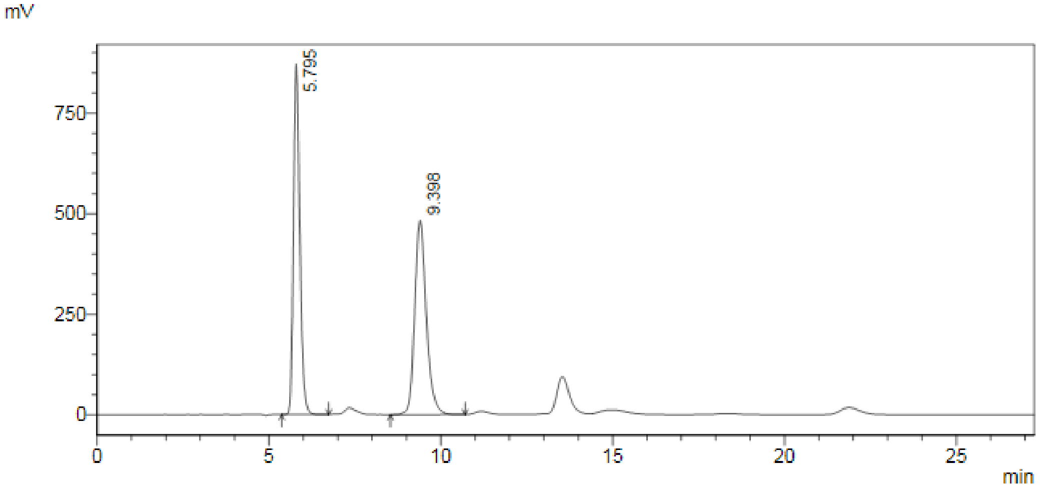

Detector A Channel 1 254nm

Peak#	Ret. Time	Area%
1	5.795	49.846
2	9.398	50.154
Total		100.000

Chiral
non-racemic

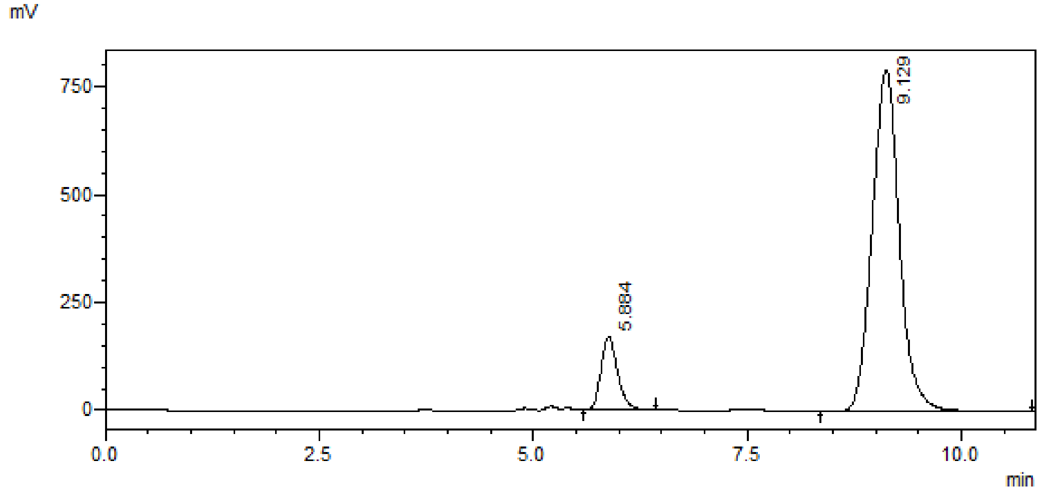

Detector A Channel 1 254nm

Peak#	Ret. Time	Area%
1	5.884	11.683
2	9.129	88.317
Total		100.000

Compound 4i

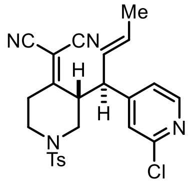

Chiral-racemic

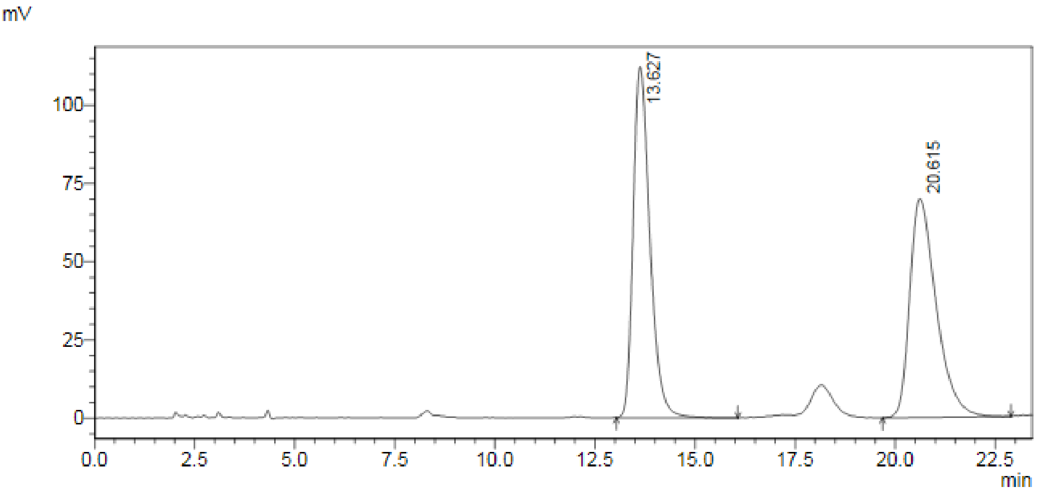

Detector A Channel 1 254nm

Peak#	Ret. Time	Area%
1	13.627	50.400
2	20.615	49.600
Total		100.000

Chiral
non-racemic
using ***rac*-2a**

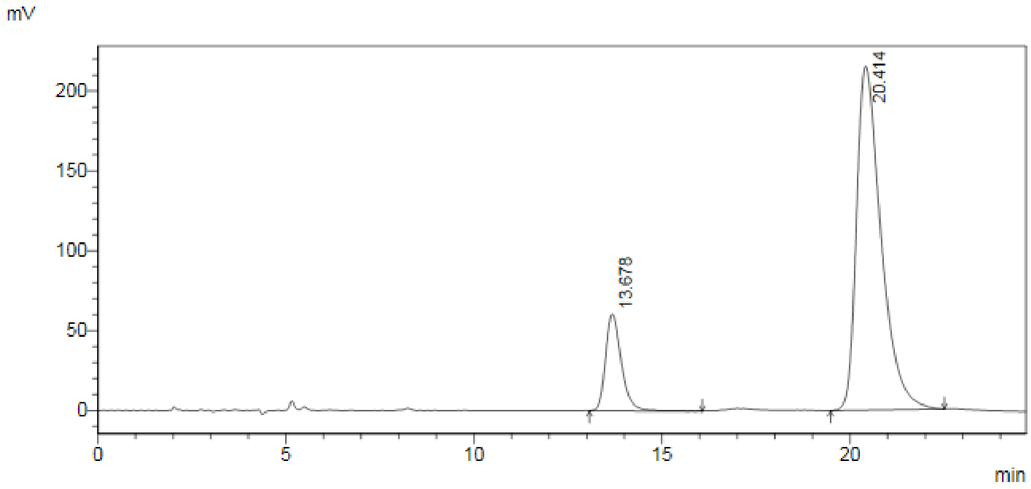

Detector A Channel 1 254nm

Peak#	Ret. Time	Area%
1	13.678	15.230
2	20.414	84.770
Total		100.000

Chiral
non-racemic
using ***rac*-2a-Ac**

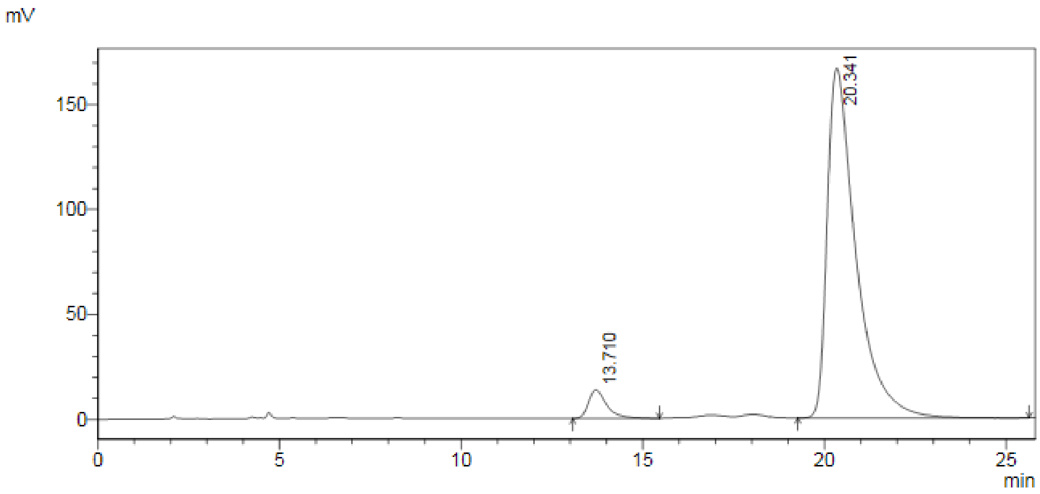

Detector A Channel 1 254nm

Peak#	Ret. Time	Area%
1	13.710	4.901
2	20.341	95.099
Total		100.000

Compound 4j

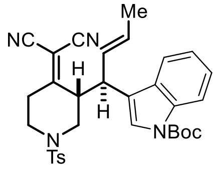

Chiral-racemic

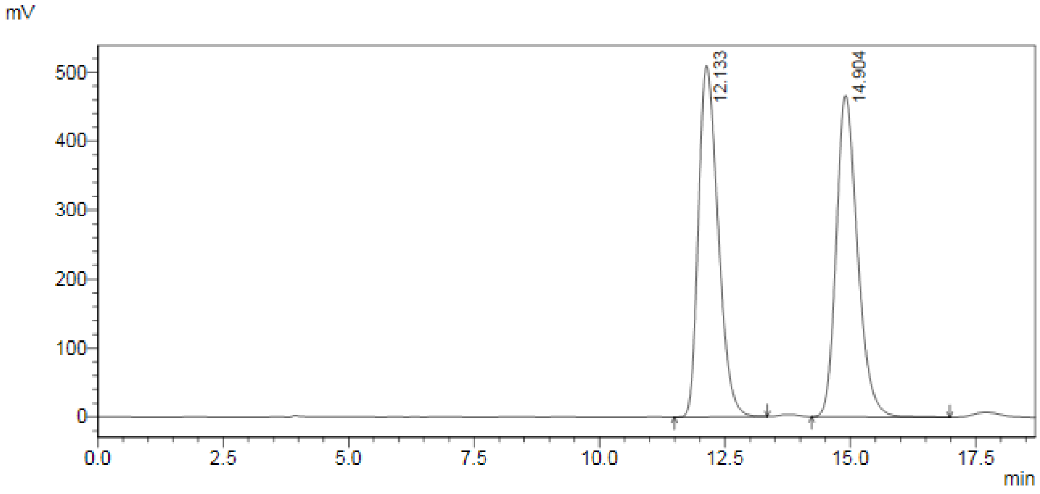

Detector A Channel 1 254nm

Peak#	Ret. Time	Area%
1	12.133	50.150
2	14.904	49.850
Total		100.000

Chiral
non-racemic

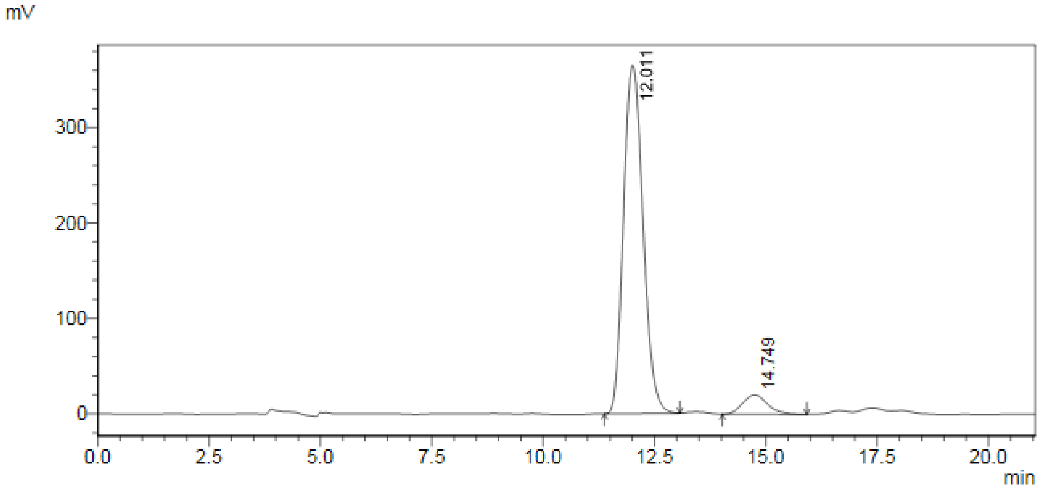

Detector A Channel 1 254nm

Peak#	Ret. Time	Area%
1	12.011	93.434
2	14.749	6.566
Total		100.000

Compound 4k

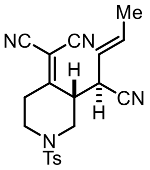

Chiral-racemic

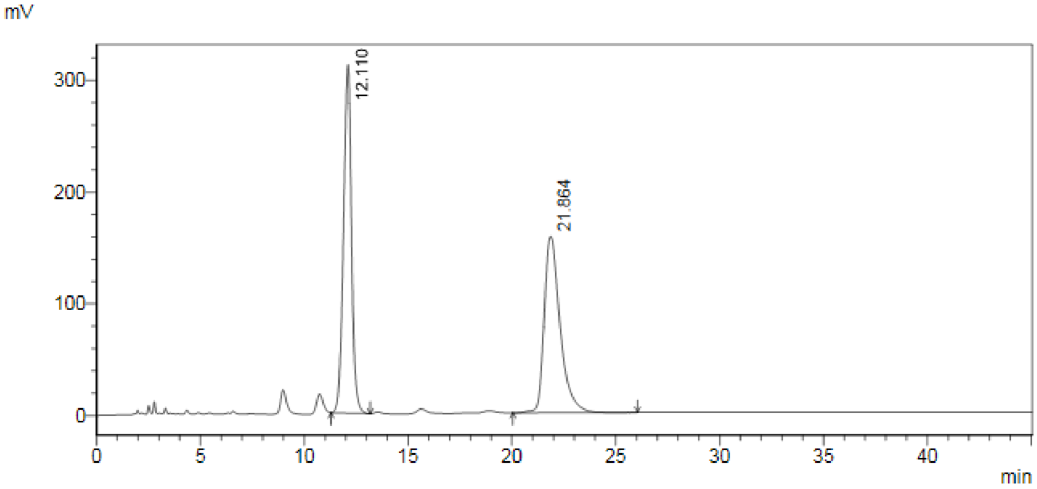

Detector A Channel 1 254nm

Peak#	Ret. Time	Area%
1	12.110	49.865
2	21.864	50.135
Total		100.000

Chiral
non-racemic

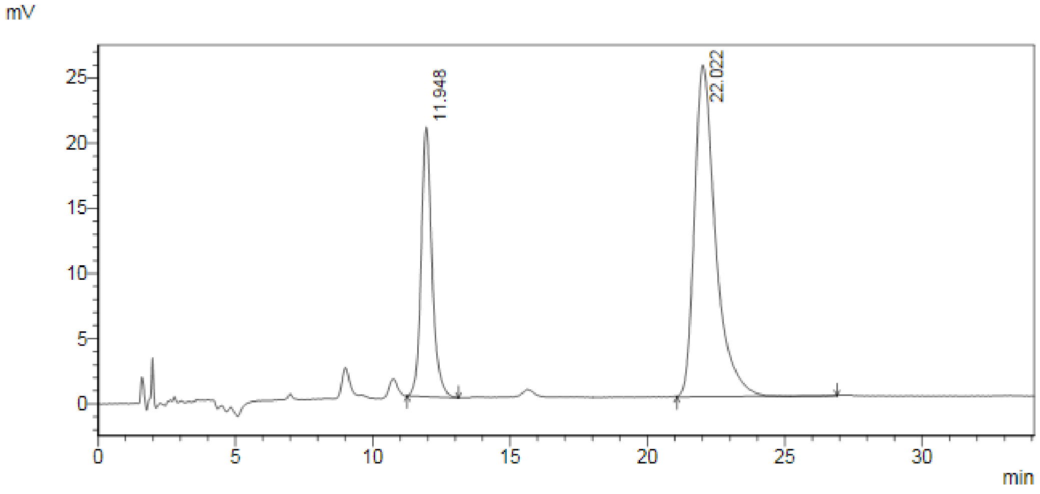

Detector A Channel 1 254nm

Peak#	Ret. Time	Area%
1	11.948	29.535
2	22.022	70.465
Total		100.000

Compound 4l

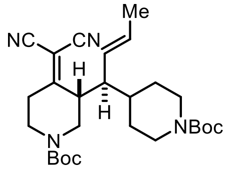

Chiral-racemic

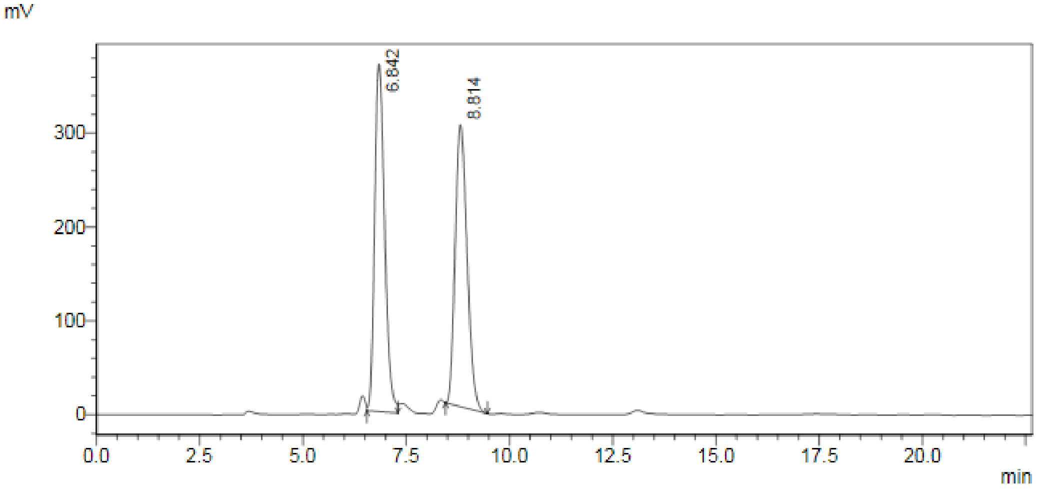

Detector A Channel 1 254nm

Peak#	Ret. Time	Area%
1	6.842	50.829
2	8.814	49.171
Total		100.000

Chiral
non-racemic

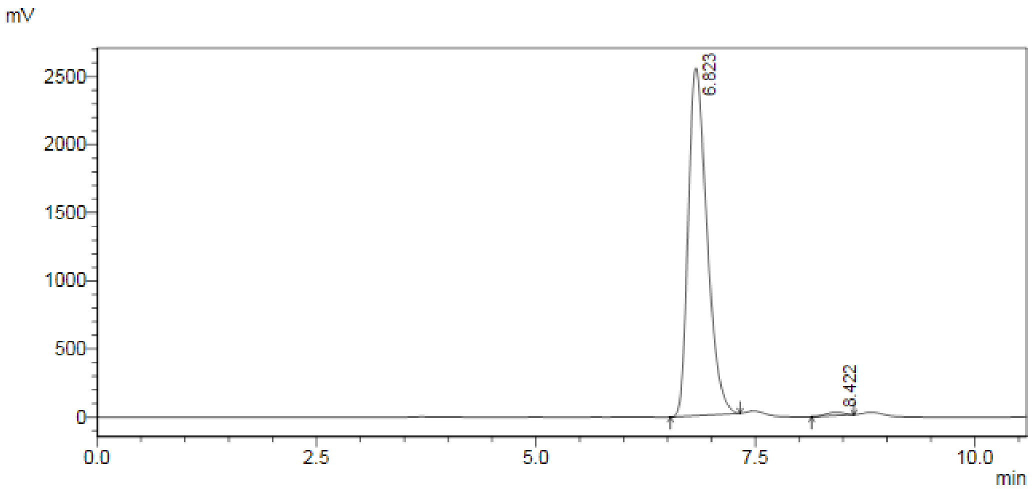

Detector A Channel 1 254nm

Peak#	Ret. Time	Area%
1	6.823	99.099
2	8.422	0.901
Total		100.000

Compound 4m

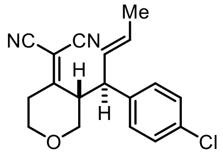

Chiral-racemic

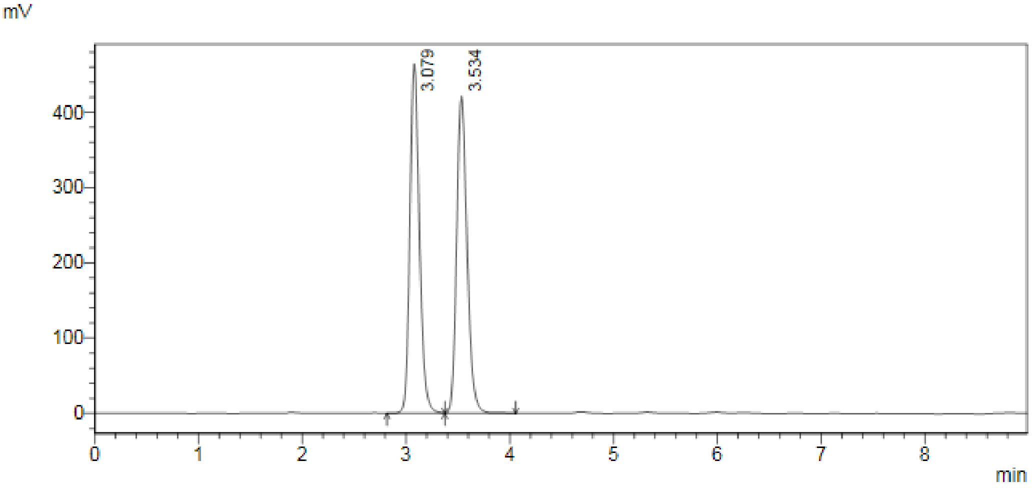

Detector A Channel 1 254nm

Peak#	Ret. Time	Area%
1	3.079	50.248
2	3.534	49.752
Total		100.000

Chiral
non-racemic

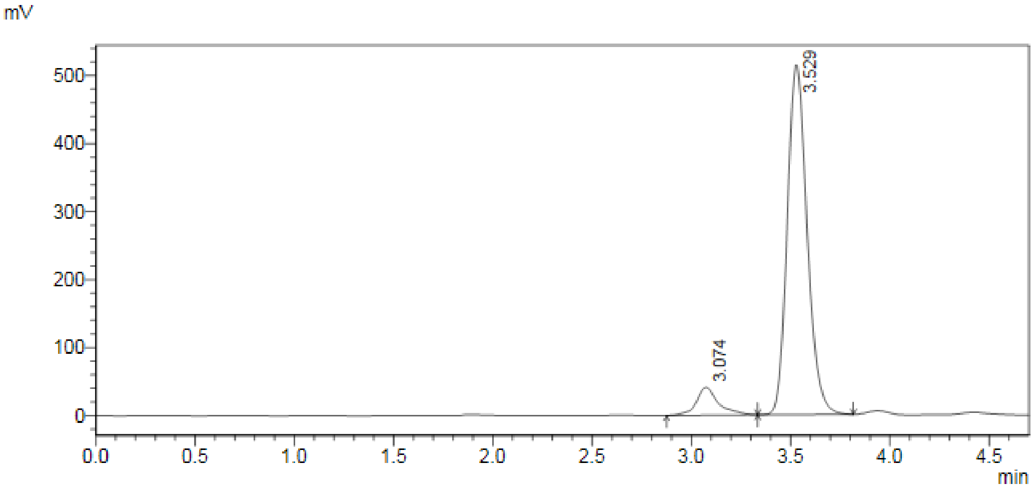

Detector A Channel 1 254nm

Peak#	Ret. Time	Area%
1	3.074	8.144
2	3.529	91.856
Total		100.000

Compound 4n

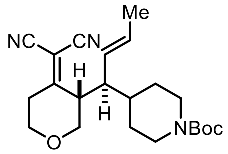

Chiral-racemic

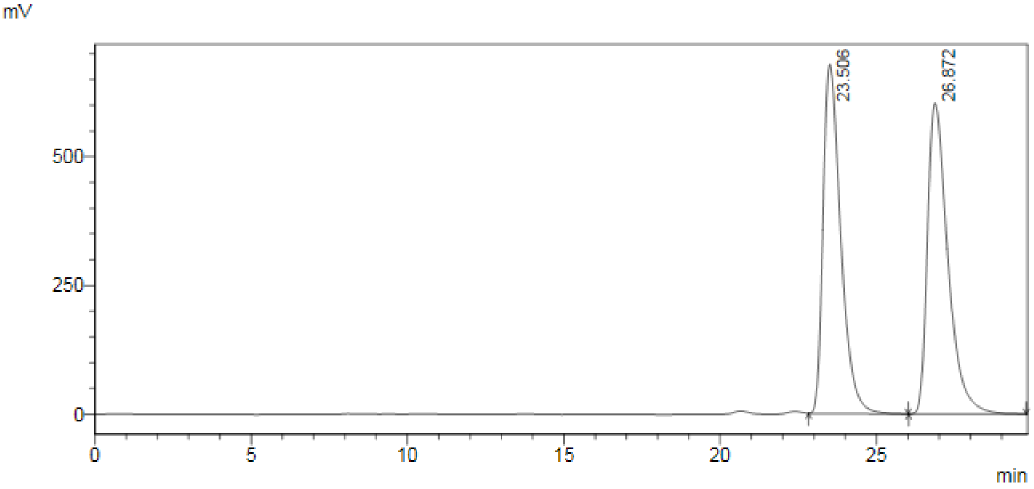

Detector A Channel 1 254nm

Peak#	Ret. Time	Area%
1	23.506	49.307
2	26.872	50.693
Total		100.000

Chiral
non-racemic

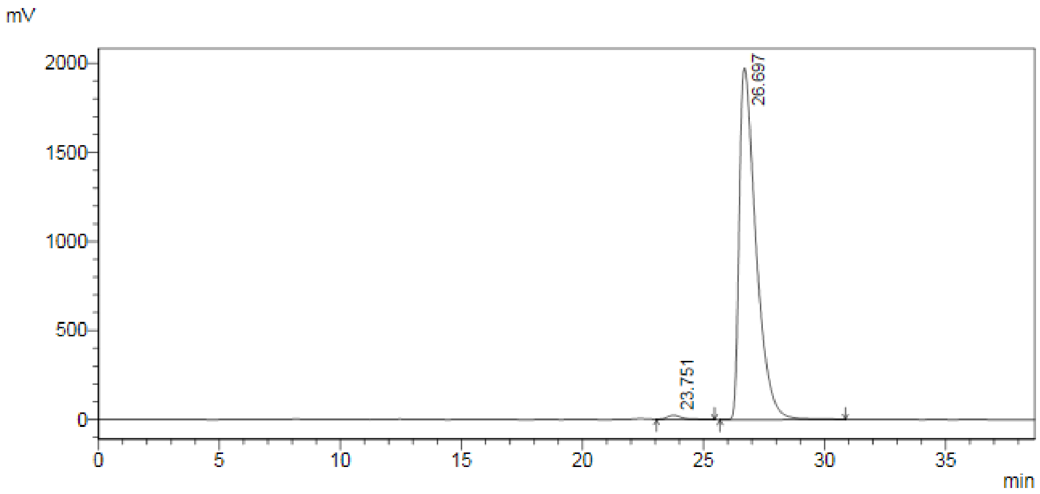

Detector A Channel 1 254nm

Peak#	Ret. Time	Area%
1	23.751	0.979
2	26.697	99.021
Total		100.000

Compound 4o

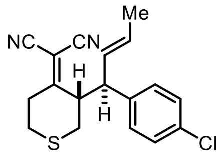

Chiral-racemic

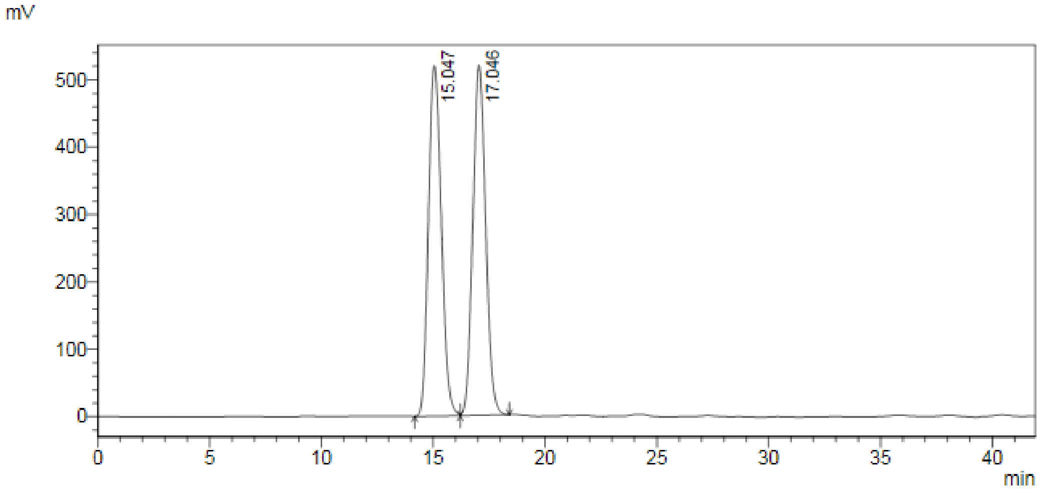

Detector A Channel 1 254nm

Peak#	Ret. Time	Area%
1	15.047	50.129
2	17.046	49.871
Total		100.000

Chiral
non-racemic

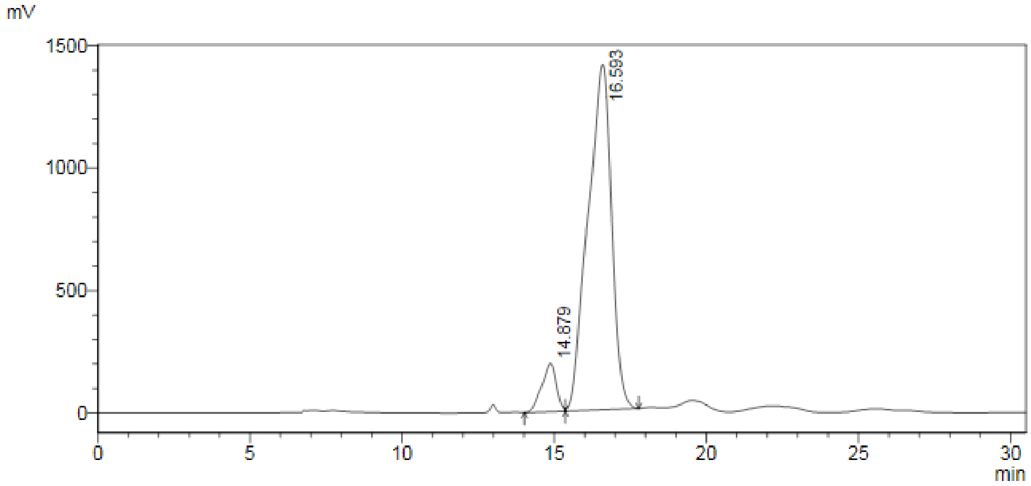

Detector A Channel 1 254nm

Peak#	Ret. Time	Area%
1	14.879	7.991
2	16.593	92.009
Total		100.000

Compound 4p

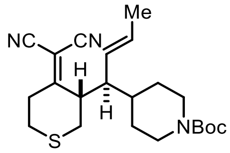

Chiral-racemic

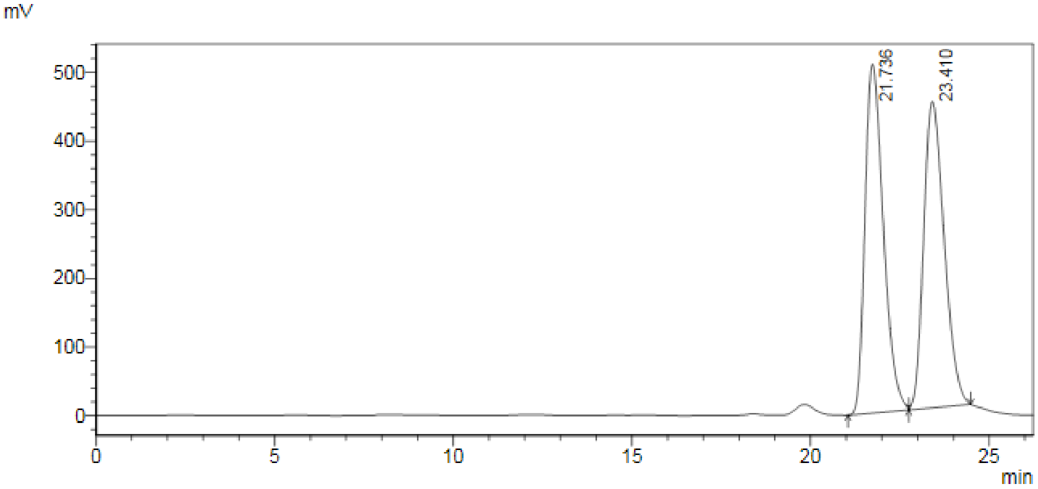

Detector A Channel 1 254nm

Peak#	Ret. Time	Area%
1	21.736	50.451
2	23.410	49.549
Total		100.000

Chiral
non-racemic

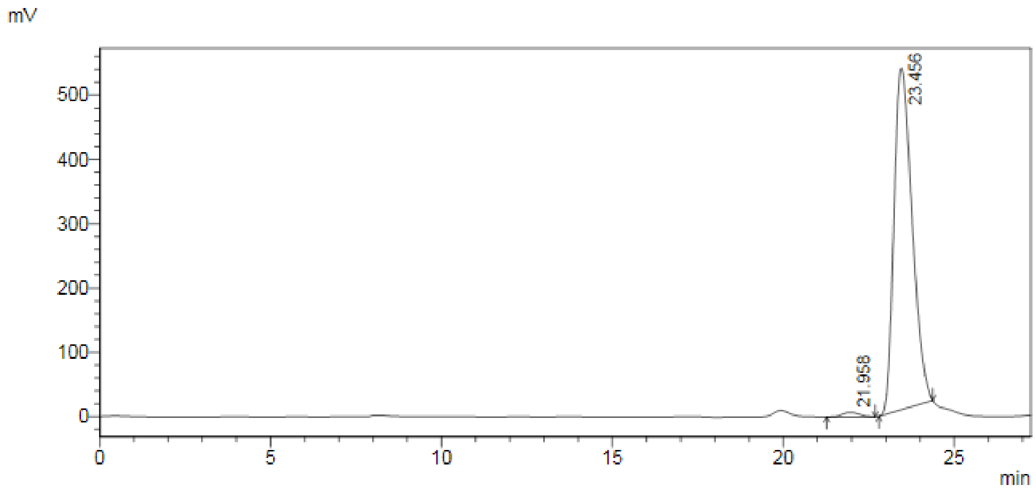

Detector A Channel 1 254nm

Peak#	Ret. Time	Area%
1	21.958	1.230
2	23.456	98.770
Total		100.000

Compound 4q

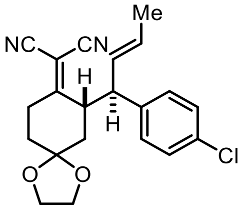

Chiral-racemic

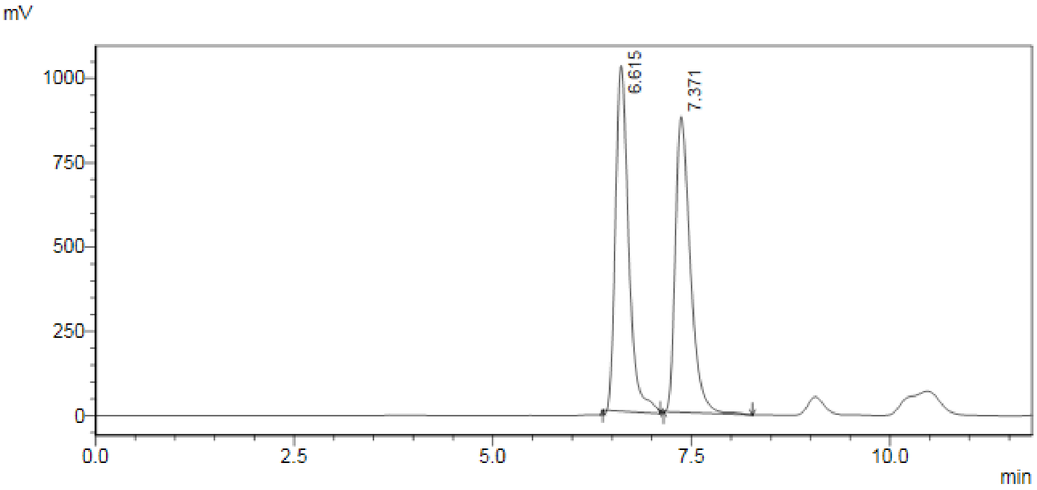

Detector A Channel 1 254nm

Peak#	Ret. Time	Area%
1	6.615	50.524
2	7.371	49.476
Total		100.000

Chiral
non-racemic

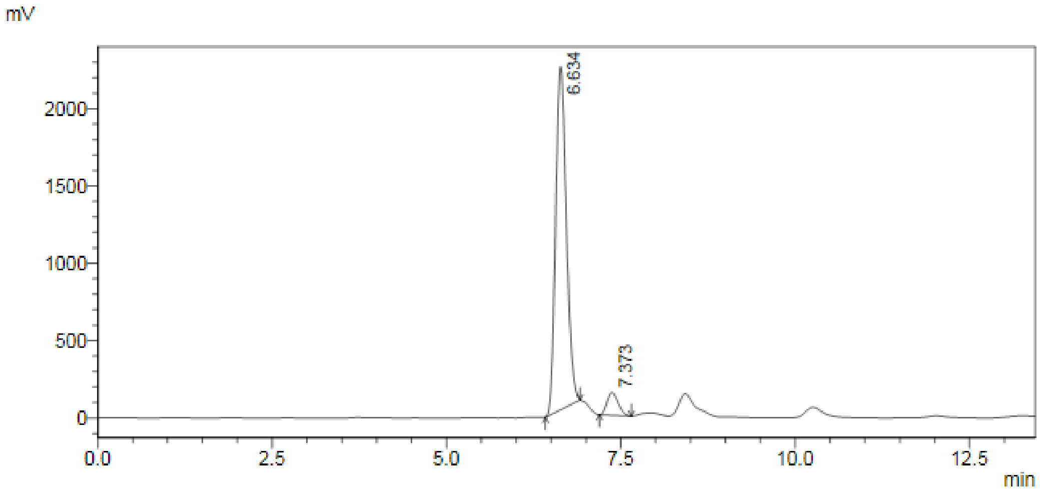

Detector A Channel 1 254nm

Peak#	Ret. Time	Area%
1	6.634	93.586
2	7.373	6.414
Total		100.000

Compound 4r

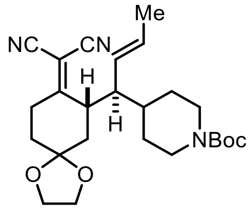

Chiral-racemic

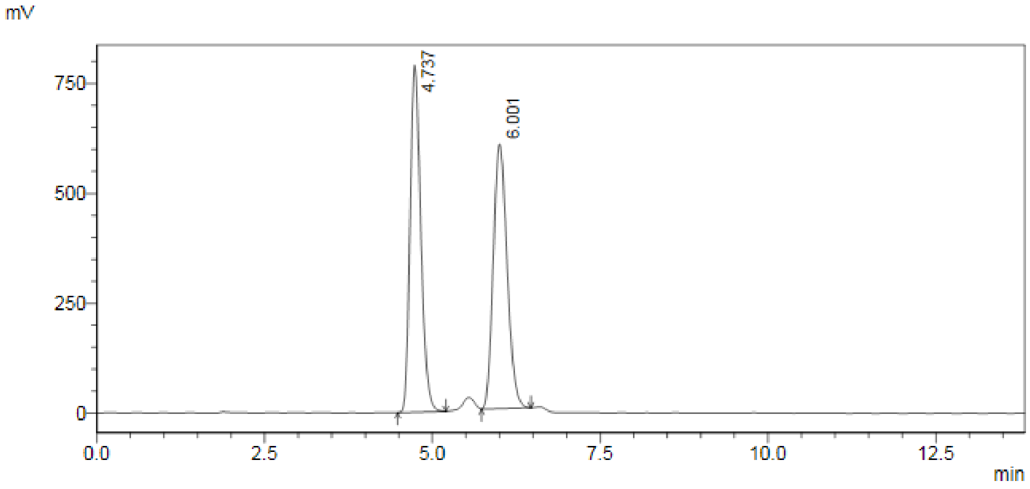

Detector A Channel 1 254nm

Peak#	Ret. Time	Area%
1	4.737	50.607
2	6.001	49.393
Total		100.000

Chiral
non-racemic

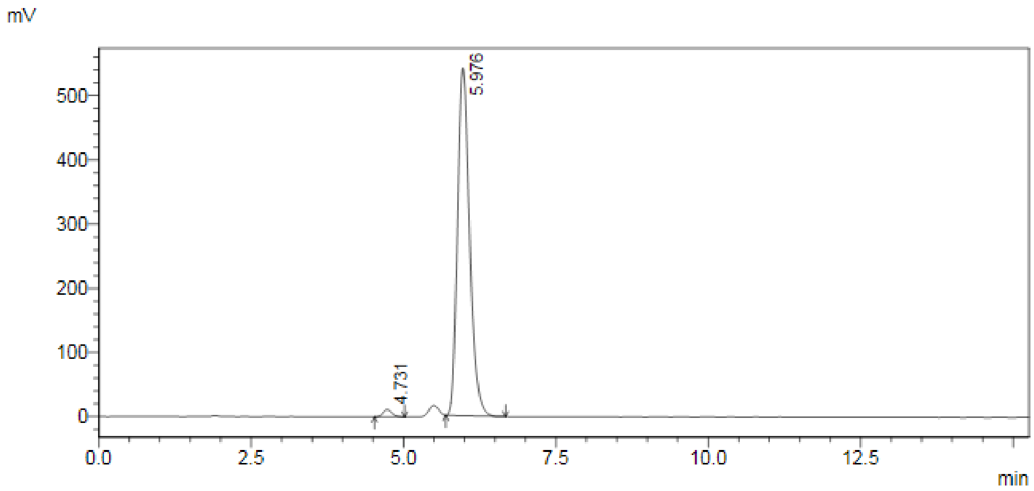

Detector A Channel 1 254nm

Peak#	Ret. Time	Area%
1	4.731	1.544
2	5.976	98.456
Total		100.000

Compound 4s

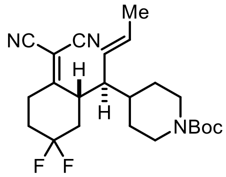

Chiral-racemic

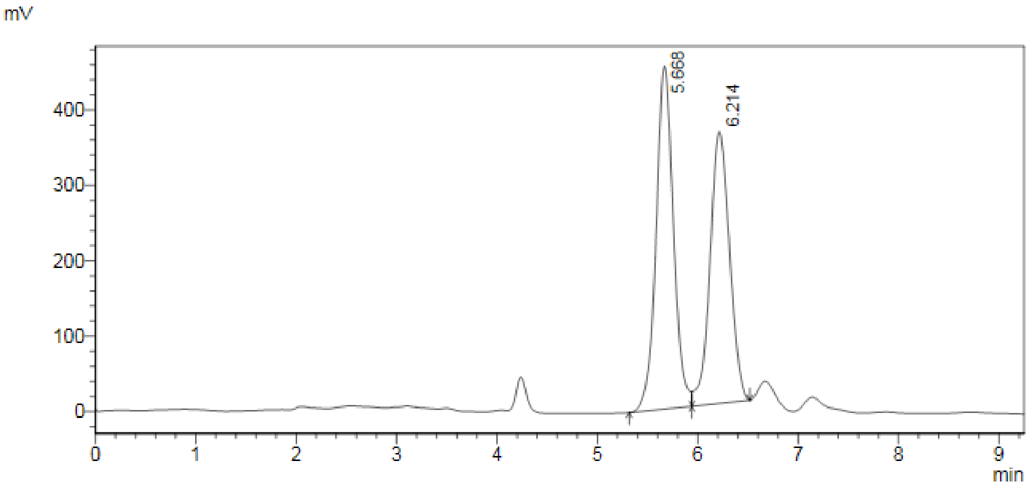

Detector A Channel 1 254nm

Peak#	Ret. Time	Area%
1	5.668	52.839
2	6.214	47.161
Total		100.000

Chiral
non-racemic

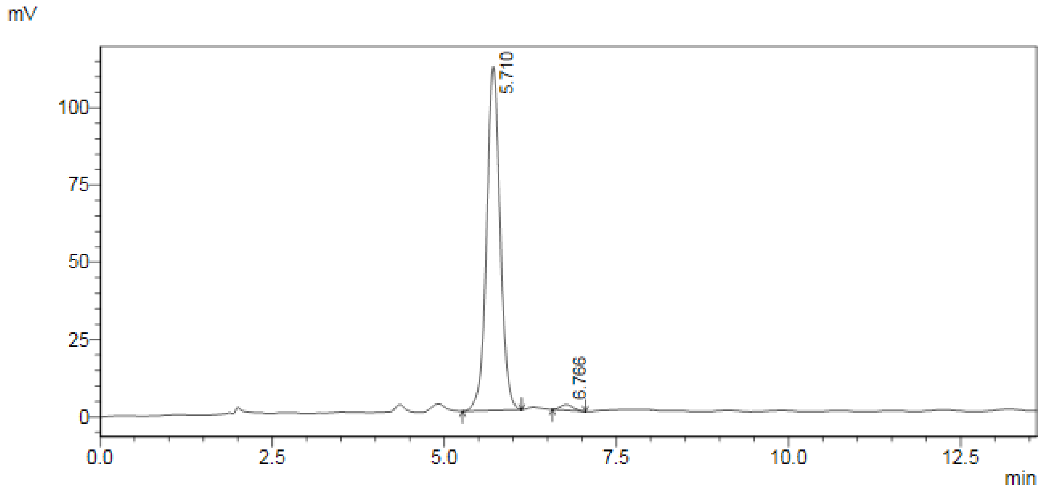

Detector A Channel 1 254nm

Peak#	Ret. Time	Area%
1	5.710	98.494
2	6.766	1.506
Total		100.000

Compound 4t

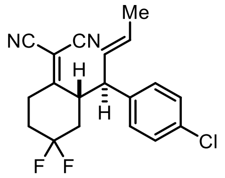

Chiral-racemic

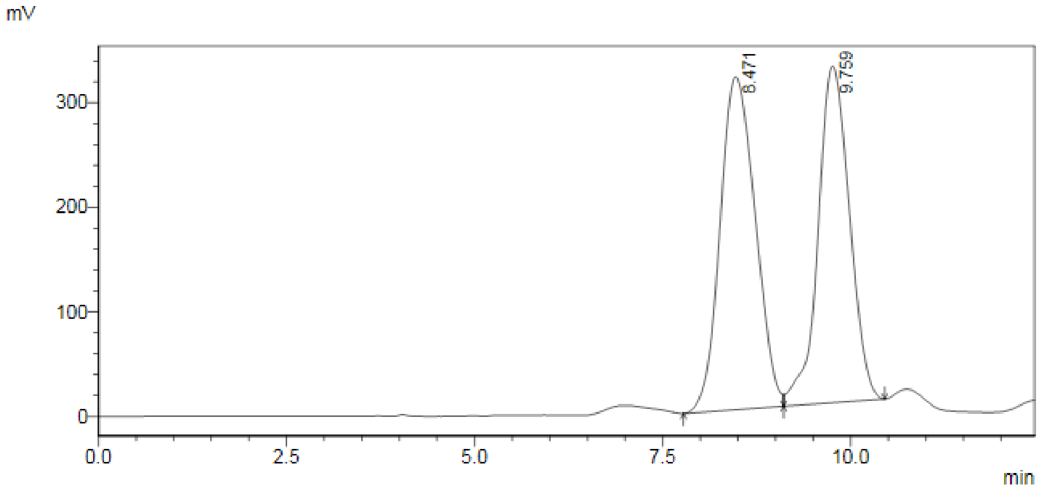

Detector A Channel 1 254nm

Peak#	Ret. Time	Area%
1	8.471	52.474
2	9.759	47.526
Total		100.000

Chiral
non-racemic

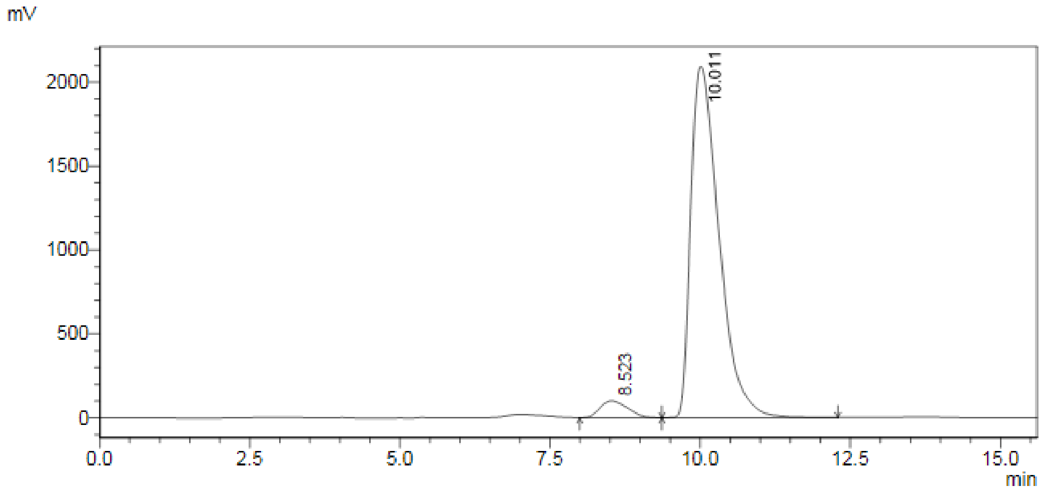

Detector A Channel 1 254nm

Peak#	Ret. Time	Area%
1	8.523	4.479
2	10.011	95.521
Total		100.000

Compound 4u

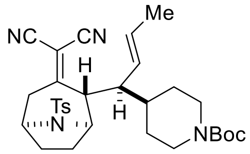

Chiral-
racemic

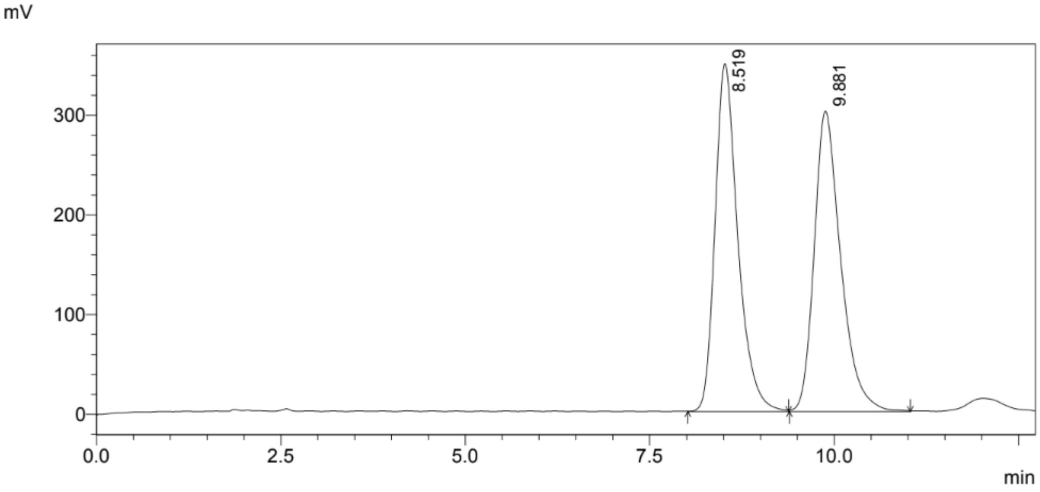

Detector A Channel 1 254nm

Peak#	Ret. Time	Area%
1	8.519	49.862
2	9.881	50.138
Total		100.000

Chiral
non-racemic

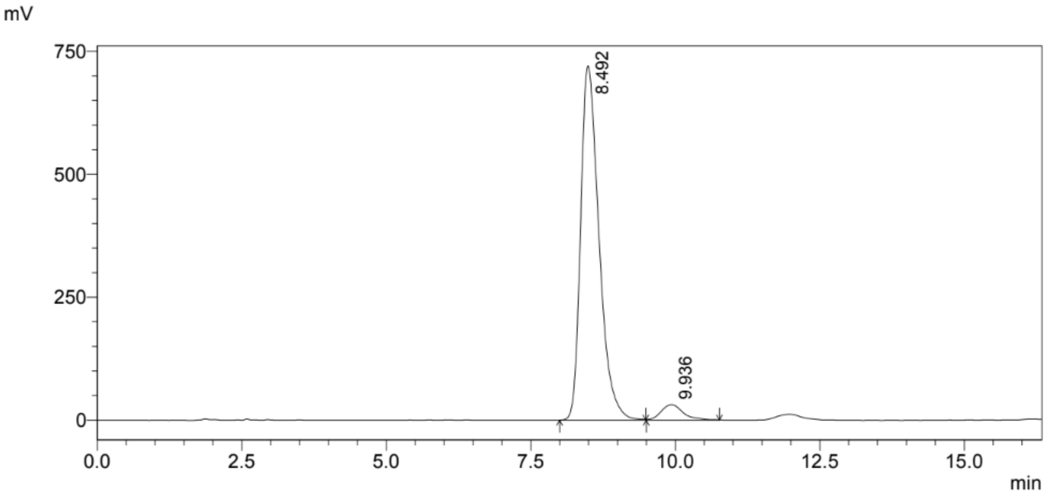

Detector A Channel 1 254nm

Peak#	Ret. Time	Area%
1	8.492	94.893
2	9.936	5.107
Total		100.000

Compound 4v

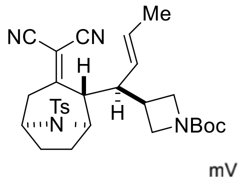

Chiral-racemic

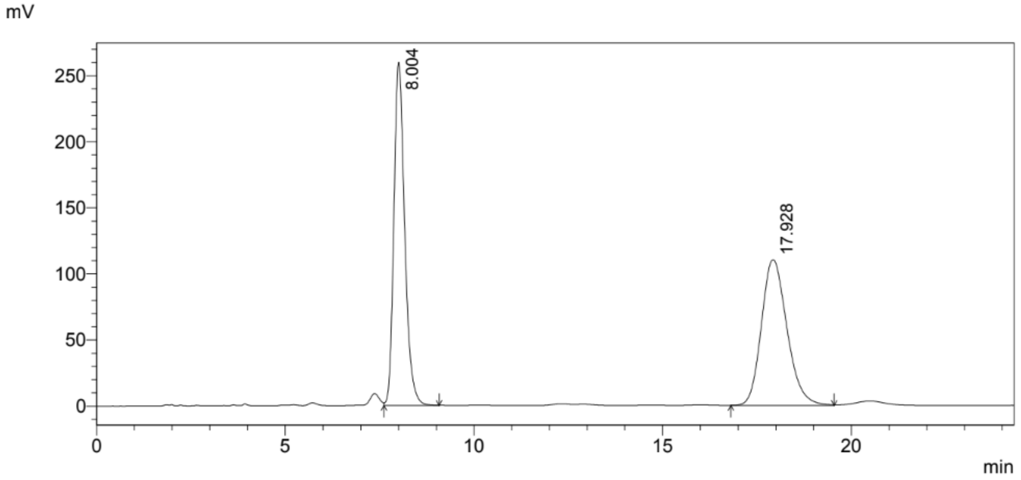

Detector A Channel 1 254nm

Peak#	Ret. Time	Area%
1	8.004	49.953
2	17.928	50.047
Total		100.000

Chiral
non-racemic

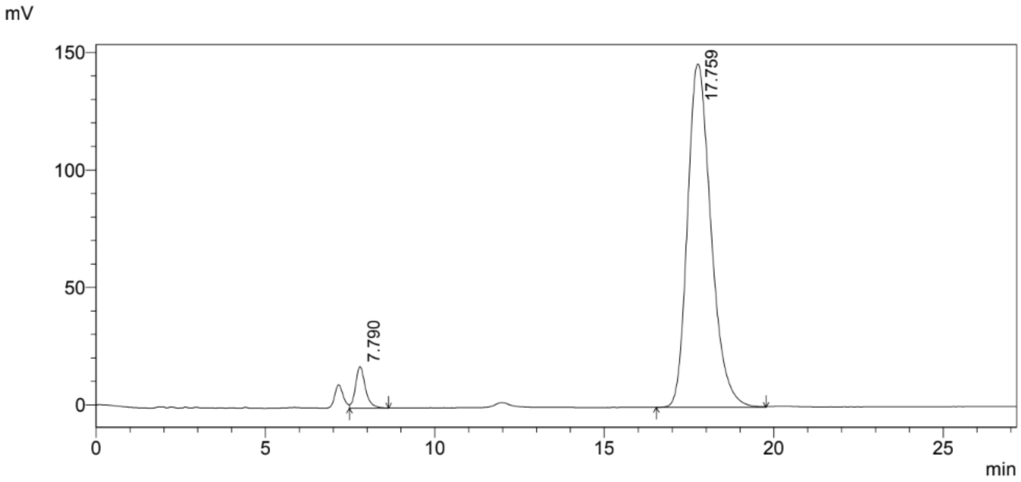

Detector A Channel 1 254nm

Peak#	Ret. Time	Area%
1	7.790	4.795
2	17.759	95.205
Total		100.000

Compound 4w

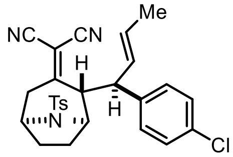

Chiral-racemic

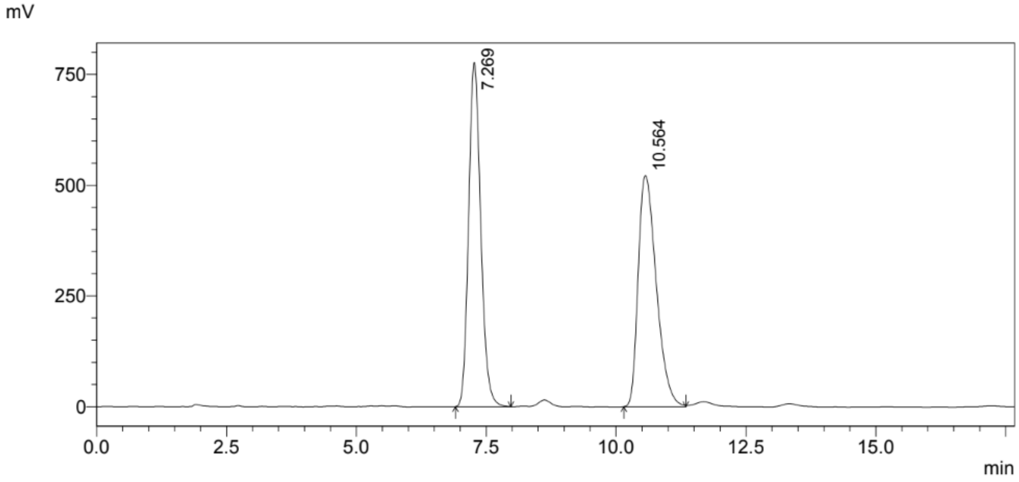

Detector A Channel 1 254nm

Peak#	Ret. Time	Area%
1	7.269	49.947
2	10.564	50.053
Total		100.000

Chiral
non-racemic

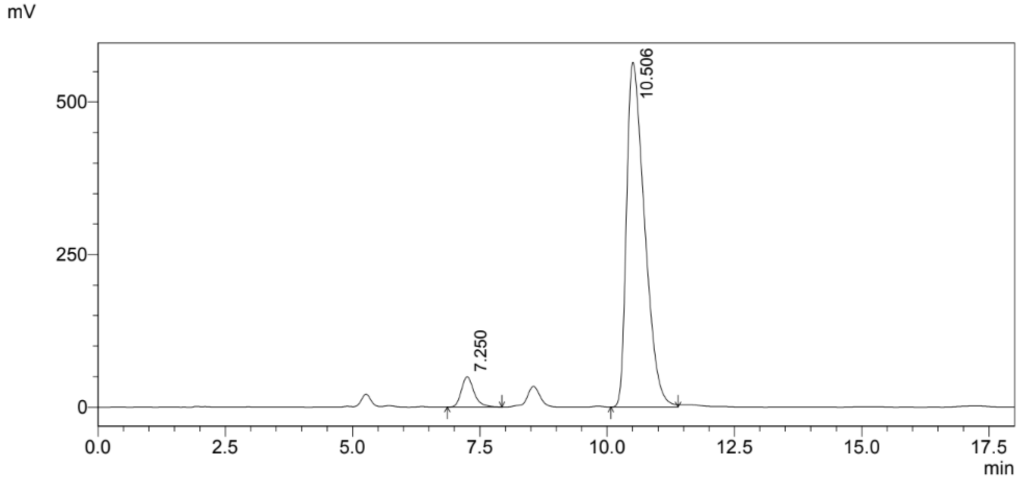

Detector A Channel 1 254nm

Peak#	Ret. Time	Area%
1	7.250	5.825
2	10.506	94.175
Total		100.000

Compound 4x

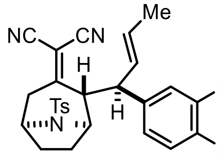

Chiral-racemic

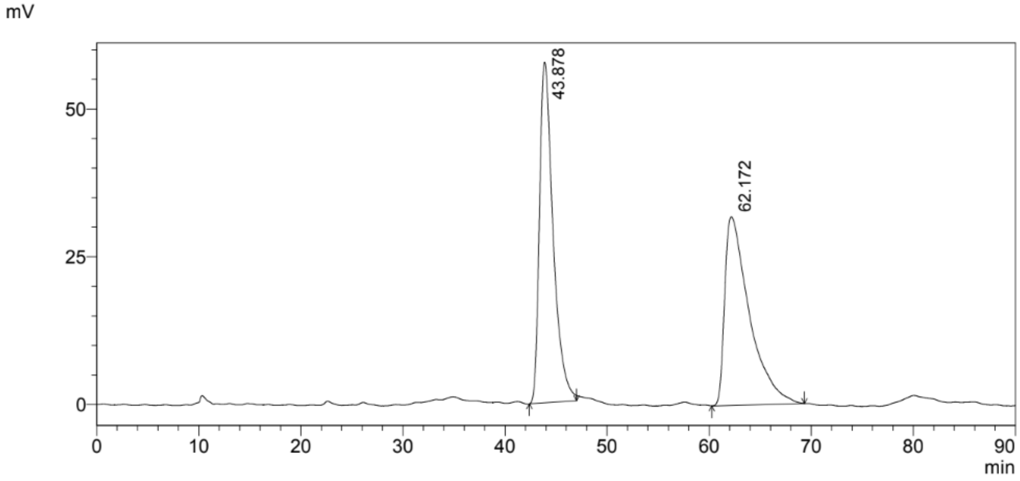

Detector A Channel 2 280nm

Peak#	Ret. Time	Area%
1	43.878	50.028
2	62.172	49.972
Total		100.000

Chiral
non-racemic

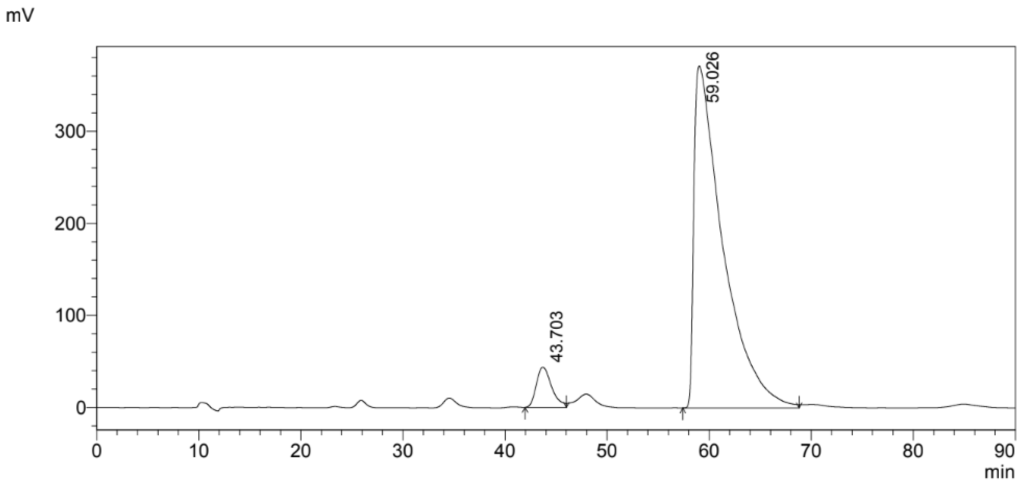

Detector A Channel 2 280nm

Peak#	Ret. Time	Area%
1	43.703	5.882
2	59.026	94.118
Total		100.000

Compound 4y

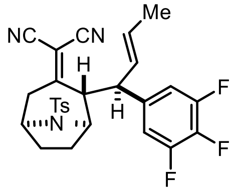

Chiral-racemic

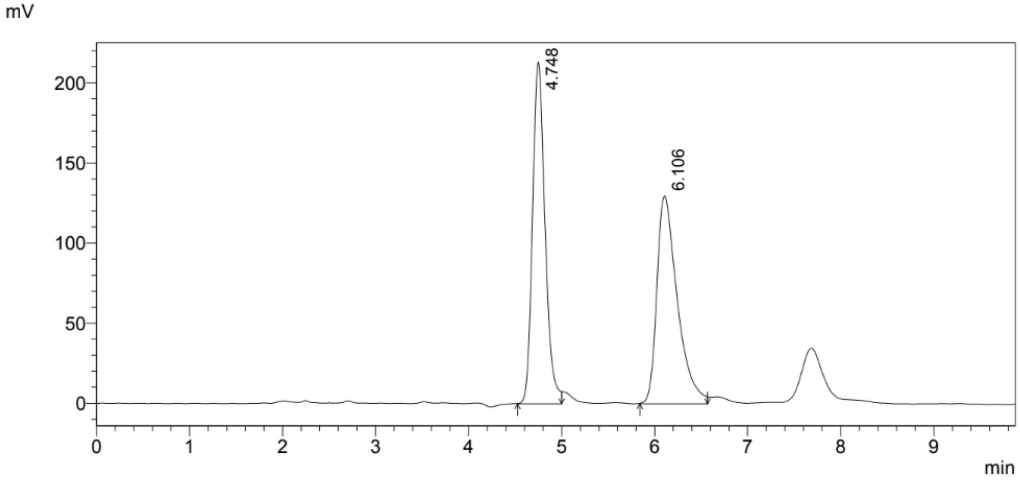

Detector A Channel 1 254nm

Peak#	Ret. Time	Area%
1	4.748	50.189
2	6.106	49.811
Total		100.000

Chiral
non-racemic

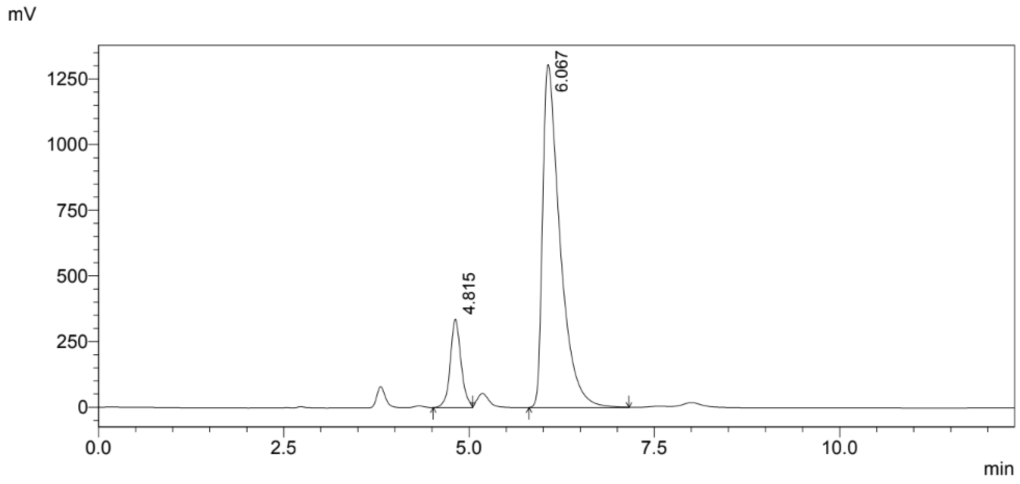

Detector A Channel 1 254nm

Peak#	Ret. Time	Area%
1	4.815	13.723
2	6.067	86.277
Total		100.000

Chiral
non-racemic,
single crystal

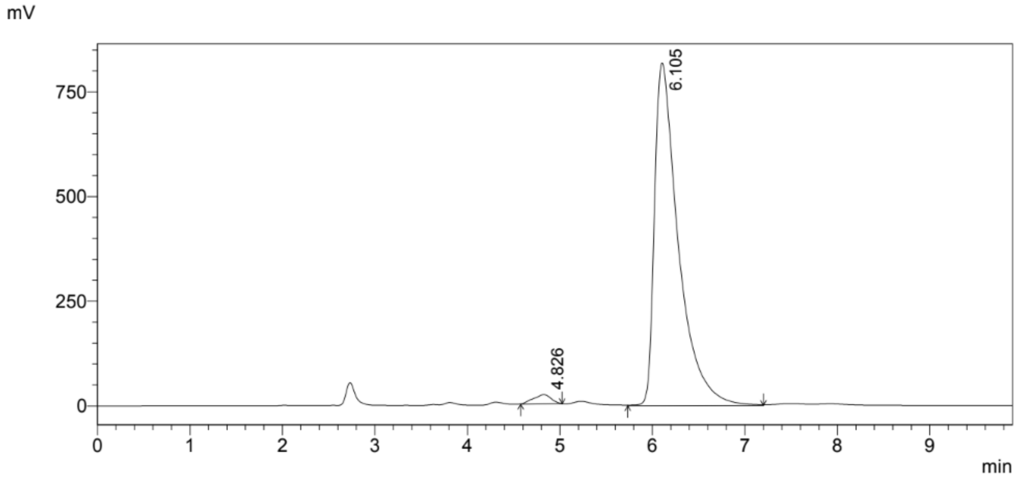

Detector A Channel 1 254nm

Peak#	Ret. Time	Area%
1	4.826	1.991
2	6.105	98.009
Total		100.000

Compound 4z

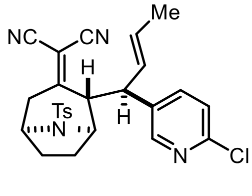

Chiral-racemic

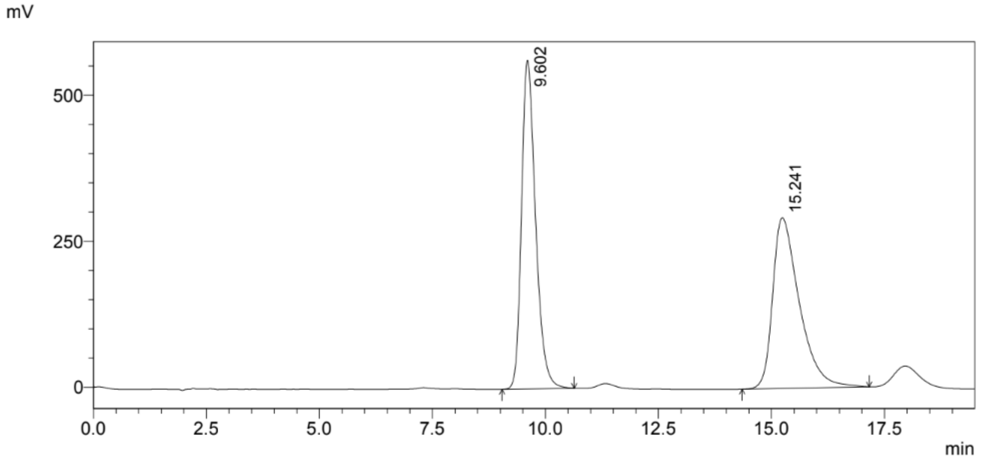

Detector A Channel 1 254nm

Peak#	Ret. Time	Area%
1	9.602	50.300
2	15.241	49.700
Total		100.000

Chiral
non-racemic

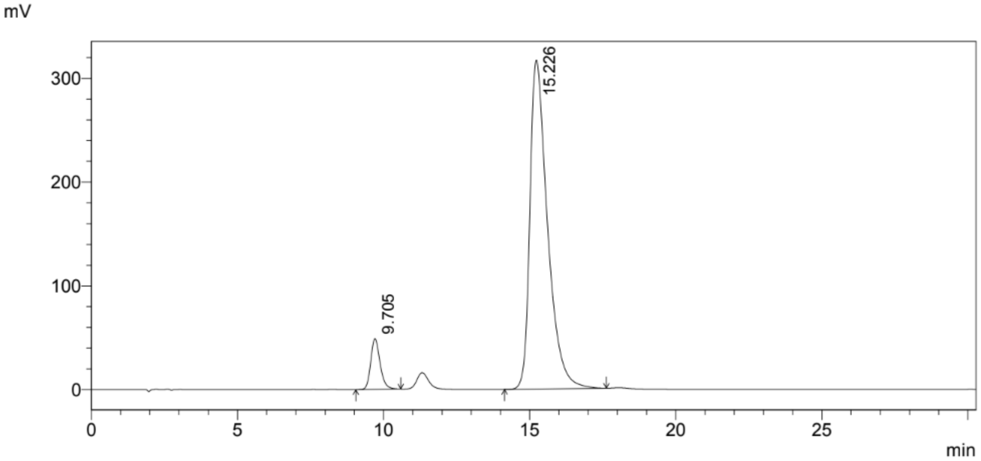

Detector A Channel 1 254nm

Peak#	Ret. Time	Area%
1	9.705	7.664
2	15.226	92.336
Total		100.000

Compound 4aa

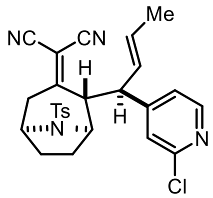

Chiral-racemic

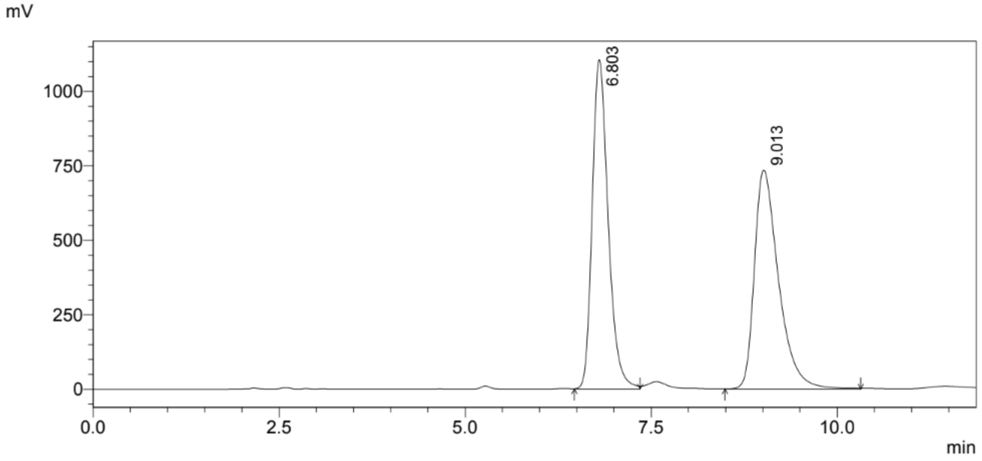

Detector A Channel 1 254nm

Peak#	Ret. Time	Area%
1	6.803	49.849
2	9.013	50.151
Total		100.000

Chiral
non-racemic

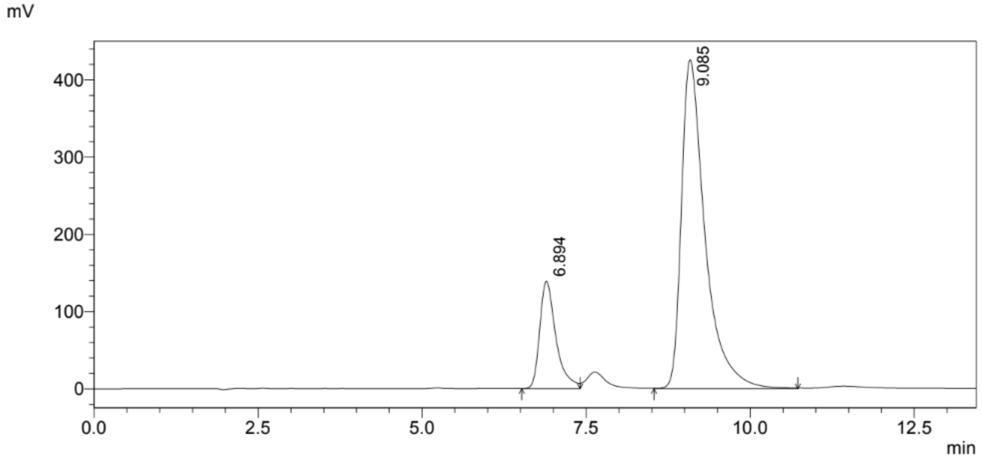

Detector A Channel 1 254nm

Peak#	Ret. Time	Area%
1	6.894	18.343
2	9.085	81.657
Total		100.000

Compound 4bb

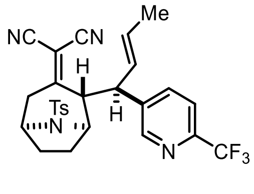

Chiral-racemic

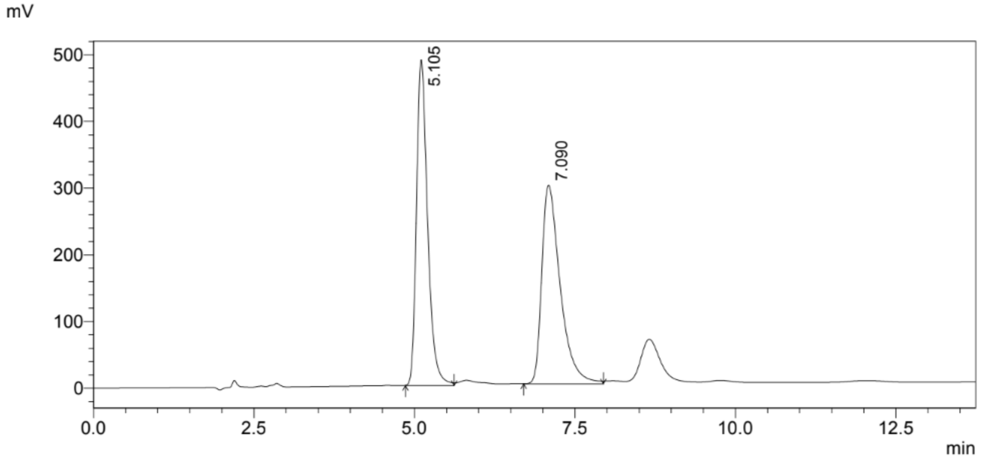

Detector A Channel 1 254nm

Peak#	Ret. Time	Area%
1	5.105	49.733
2	7.090	50.267
Total		100.000

Chiral
non-racemic

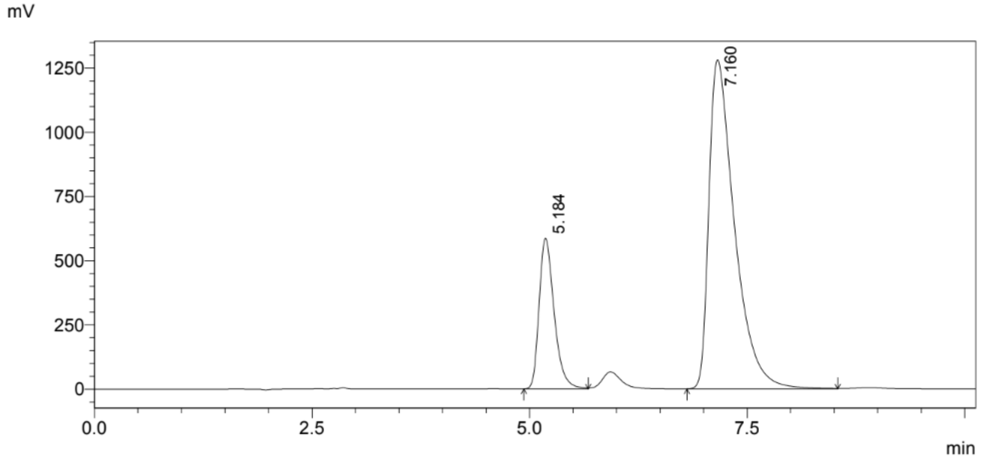

Detector A Channel 1 254nm

Peak#	Ret. Time	Area%
1	5.184	21.192
2	7.160	78.808
Total		100.000

Compound 4cc

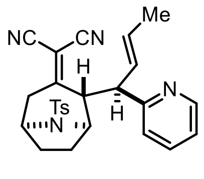

Chiral-racemic

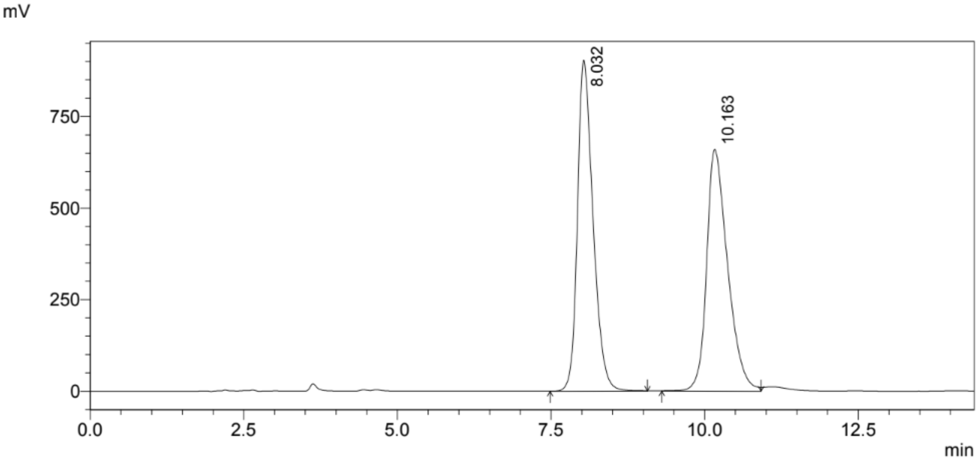

Detector A Channel 1 254nm

Peak#	Ret. Time	Area%
1	8.032	50.439
2	10.163	49.561
Total		100.000

Chiral
non-racemic

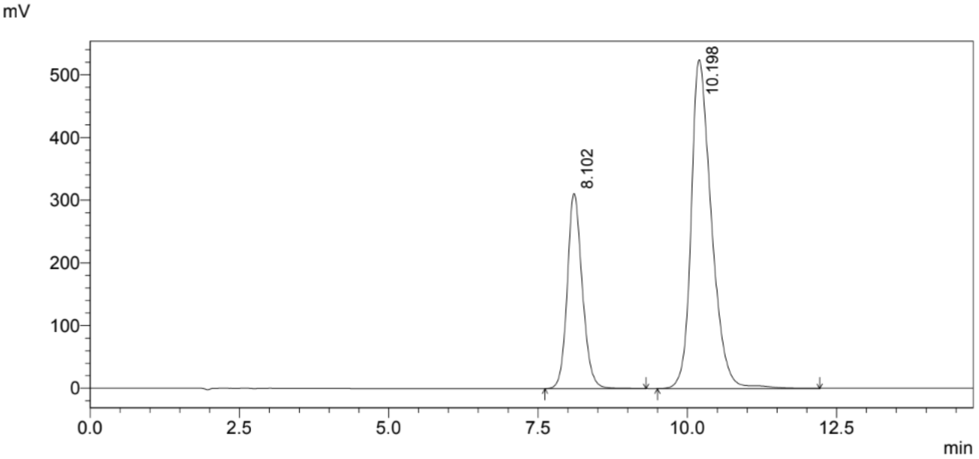

Detector A Channel 1 254nm

Peak#	Ret. Time	Area%
1	8.102	30.460
2	10.198	69.540
Total		100.000

Compound 4dd

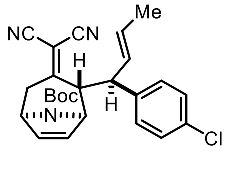

Chiral-racemic

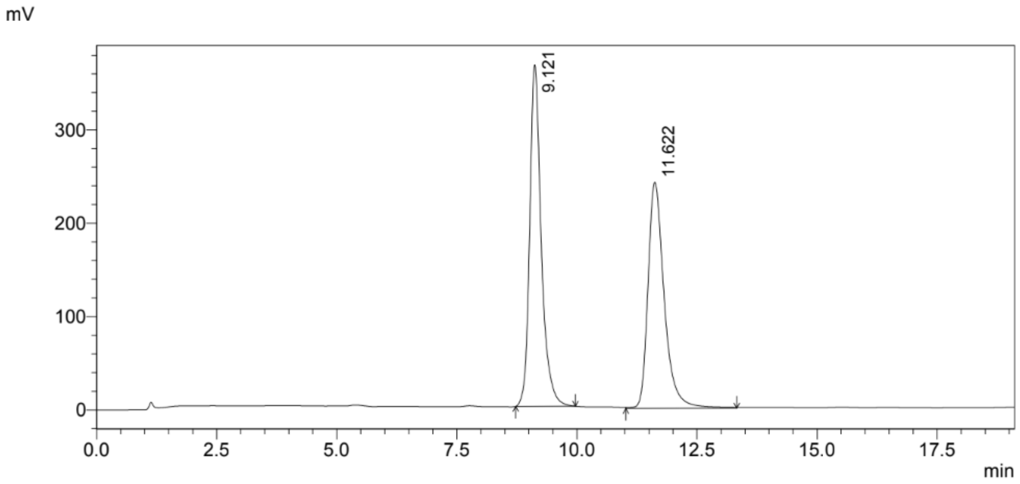

Detector A Channel 1 254nm

Peak#	Ret. Time	Area%
1	9.121	51.790
2	11.622	48.210
Total		100.000

Chiral
non-racemic

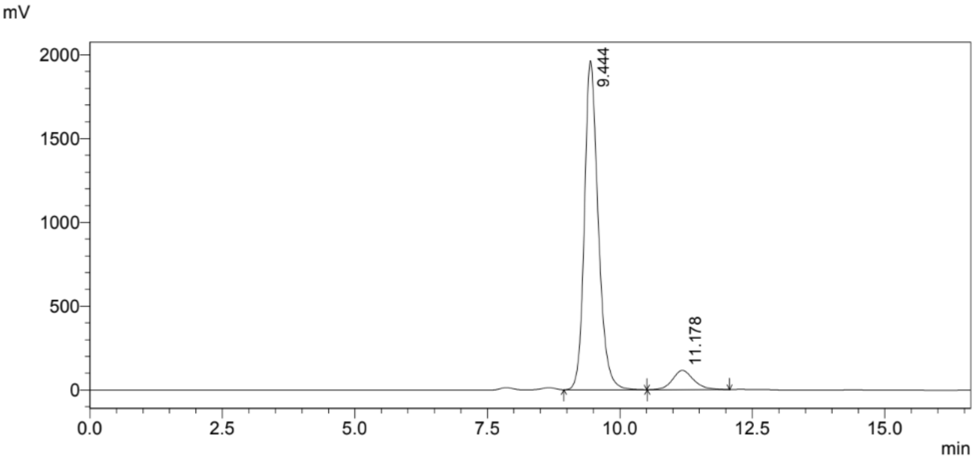

Detector A Channel 1 254nm

Peak#	Ret. Time	Area%
1	9.444	91.794
2	11.178	8.206
Total		100.000

Chiral
non-racemic,
7.4 mmol scale,
2 mol% Pd

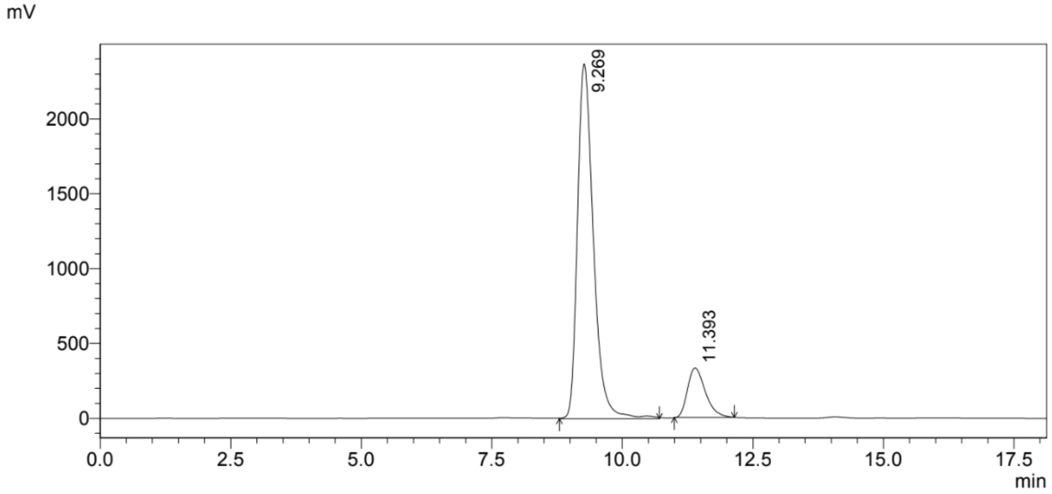

Detector A Channel 1 254nm

Peak#	Ret. Time	Area%
1	9.269	86.486
2	11.393	13.514
Total		100.000

Compound 4ee

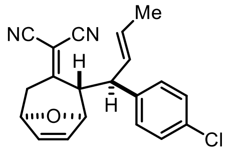

Chiral-racemic

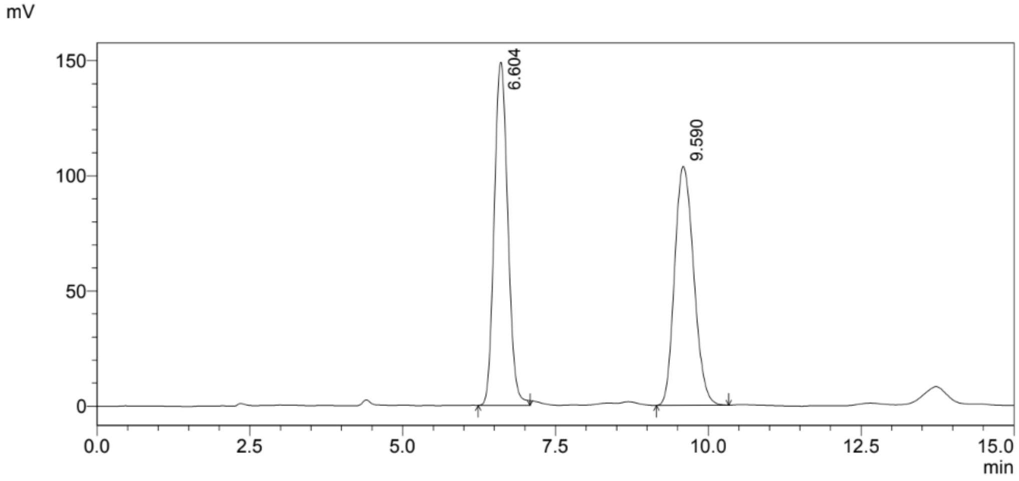

Detector A Channel 1 254nm

Peak#	Ret. Time	Area%
1	6.604	50.377
2	9.590	49.623
Total		100.000

Chiral
non-racemic

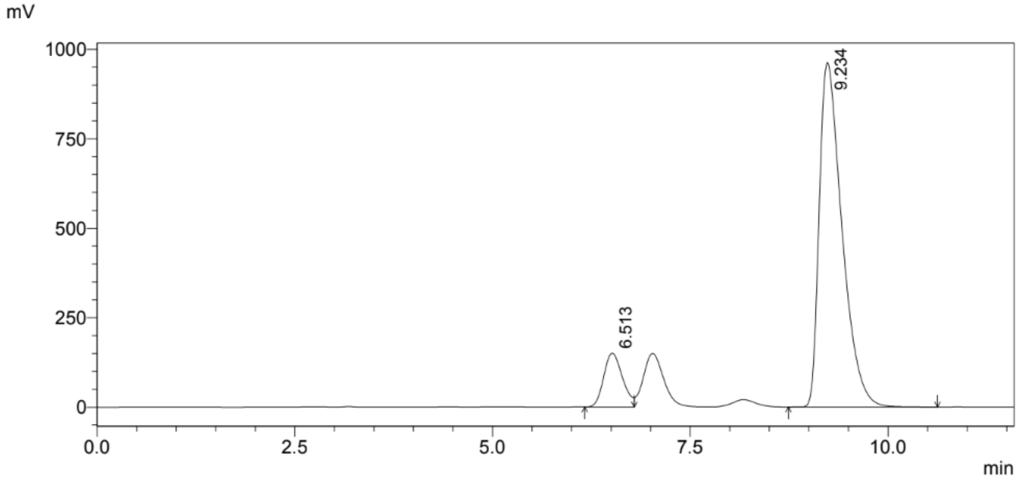

Detector A Channel 1 254nm

Peak#	Ret. Time	Area%
1	6.513	11.422
2	9.234	88.578
Total		100.000

Compound 6ff

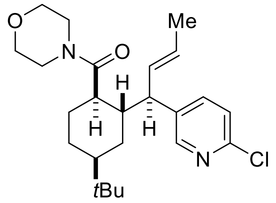

Chiral-racemic

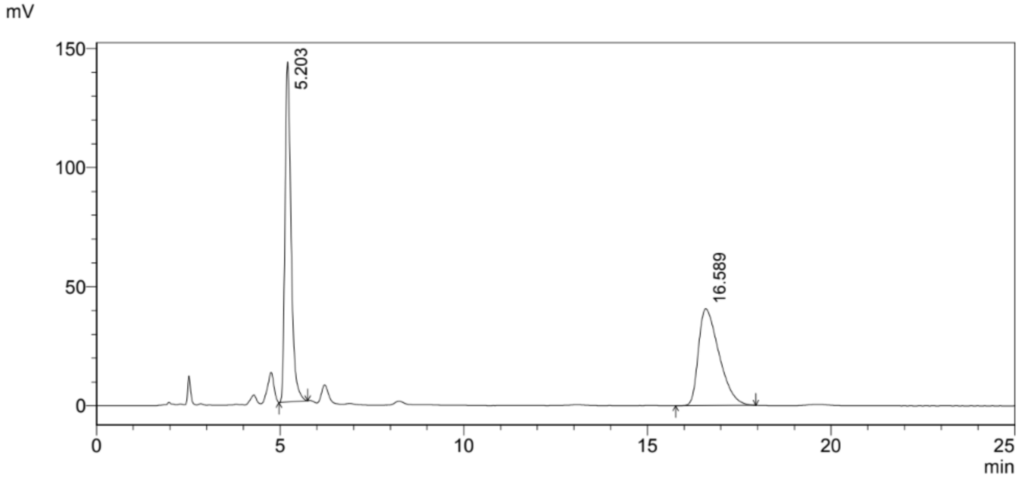

Detector A Channel 1 254nm

Peak#	Ret. Time	Area%
1	5.203	50.855
2	16.589	49.145
Total		100.000

Chiral
non-racemic

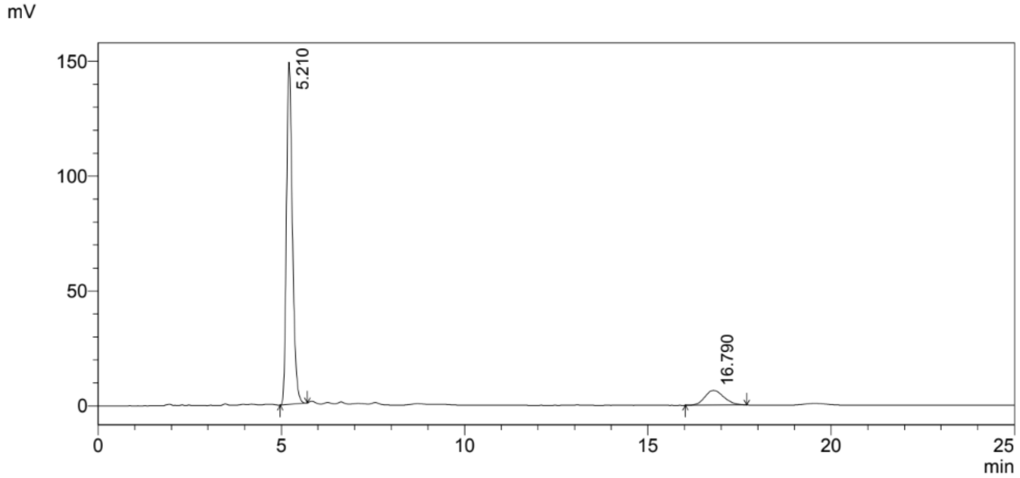

Detector A Channel 1 254nm

Peak#	Ret. Time	Area%
1	5.210	87.873
2	16.790	12.127
Total		100.000

Compound 11b

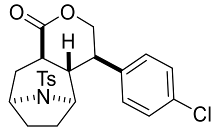

Chiral-racemic

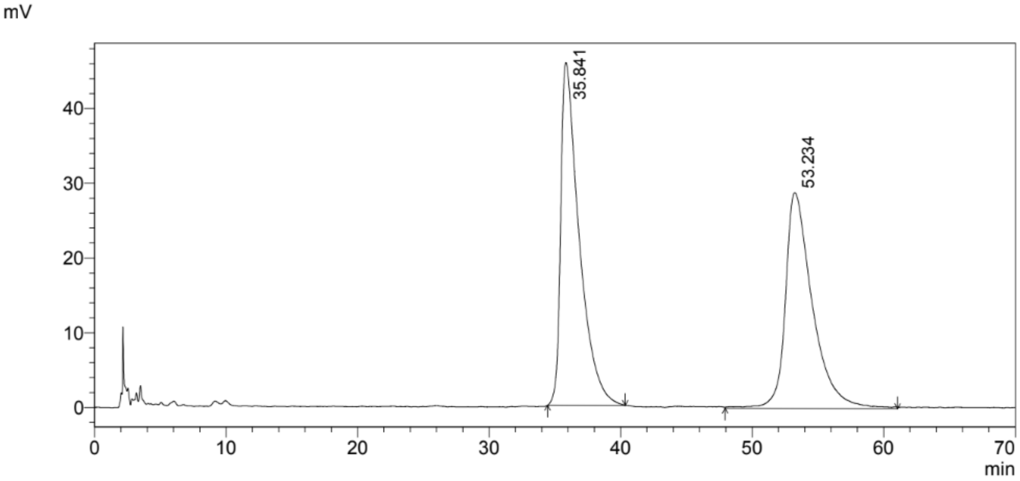

Detector A Channel 1 254nm

Peak#	Ret. Time	Area%
1	35.841	51.889
2	53.234	48.111
Total		100.000

Chiral
non-racemic

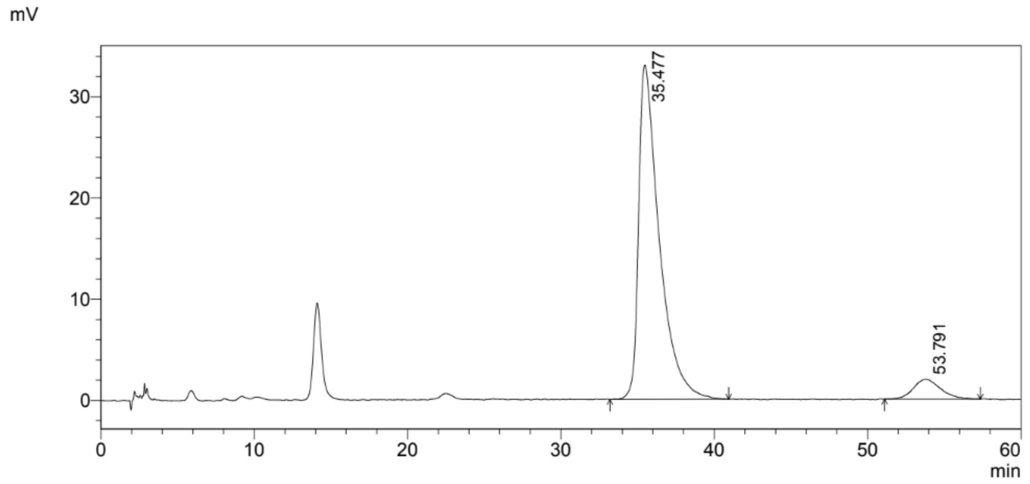

Detector A Channel 1 254nm

Peak#	Ret. Time	Area%
1	35.477	92.633
2	53.791	7.367
Total		100.000

Chiral
non-racemic,
single crystal

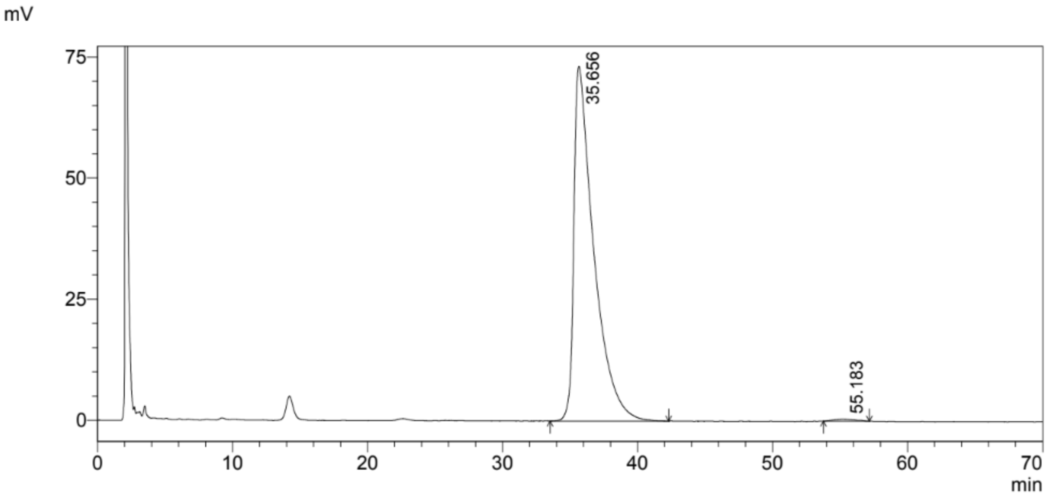

Detector A Channel 1 254nm

Peak#	Ret. Time	Area%
1	35.656	99.390
2	55.183	0.610
Total		100.000
